# Genome-wide identification and characterization of circular RNAs by high throughput sequencing in soybean

**DOI:** 10.1038/s41598-017-05922-9

**Published:** 2017-07-17

**Authors:** Wei Zhao, Yihui Cheng, Chi Zhang, Qingbo You, Xinjie Shen, Wei Guo, Yongqing Jiao

**Affiliations:** 1 Key laboratory of Biology and Genetic Improvement of Oil Crops, Ministry of Agriculture, Oil Crops Research Institute, Chinese Academy of Agricultural Science, Wuhan, China; 2 BGI-Wuhan, Wuhan, 430075 Hubei China

## Abstract

Circular RNAs (circRNAs) arise during pre-mRNA splicing, in which the 3′ and 5′ ends are linked to each other by a covalent bond. Soybean is an ancient tetraploid, which underwent two whole genome duplications. Most of soybean genes are paralogous genes with multiple copies. Although many circRNAs have been identified in animals and plants, little is known about soybean circRNAs, especially about circRNAs derived from paralogous genes. Here, we used deep sequencing technology coupled with RNase R enrichment strategy and bioinformatic approach to uncover circRNAs in soybean. A total of 5,372 circRNAs were identified, approximately 80% of which were paralogous circRNAs generated from paralogous genes. Despite high sequence homology, the paralogous genes could produce different paralogous circRNAs with different expression patterns. Two thousand and one hundred thirty four circRNAs were predicted to be 92 miRNAs target mimicry. CircRNAs and circRNA isoforms exhibited tissue-specific expression patterns in soybean. Based on the function of circRNA-host genes, the soybean circRNAs may participate in many biological processes such as developmental process, multi-organism process, and metabolic process. Our study not only provided a basis for research into the function of circRNAs in soybean but also new insights into the plant circRNA kingdom.

## Introduction

Circular RNAs (circRNAs) are a novel type of endogenous noncoding RNAs characterized by the presence of a covalent bond linking the 3′and 5′ ends^1^. Unlike linear RNAs terminated with 5′ caps and 3′ tails, circRNAs form covalently closed loop structures by back-spliced circularization without polyadenylated tails and 5′-3′ polarities^2^. Thus, circRNAs are resistant to RNase R, which is a strong 3′ to 5′ exoribonuclease that degrades linear RNAs efficiently^3^.

Although circRNAs had been observed for decades in eukaryotic cells, they were once disregarded as rare, some form of transcriptional noise or RT-PCR artifacts^4^. Until recent years, due to the high throughput sequencing technologies coupled with exonuclease-based enrichment strategies and new bioinformatic tools, such as CIRI^5^, KNIFE^6^ and UROBORUS^7^, the identification, biogenesis and function of circRNAs were widely reported in animals, such as human, mouse and *Drosophila* ^8–11^.

In humans, circularized exons are typically bracketed by long introns that highly contain complementary sequences such as ALU elements, and these short intronic inverted repeats could efficiently promote the production of circRNAs^12, 13^. However, study in *Schizosaccharomyces pombe* showed that circRNAs could also be generated through an exon-containing lariat precursor that lacked noticeable flanking intronic secondary structure^14^. Besides, RNA binding proteins, muscleblind (MBNL1), Adenosine deaminase 1 (ADAR1) and Quaking, could act as trans-factors to play important roles in the biogenesis of some circRNAs^15–17^. Thus, the biogenesis of circRNAs is still elusive, which need to be investigated further.

Recent studies had demonstrated that circRNAs could inhibit miRNA function by acting as miRNA sponge or decoys. For example, the circRNA, ciRS-7 (also termed CDR1as), contains more than 70 conventional miR-7 binding sites, and could increase the expression level of miR-7 target genes by strongly suppressing miR-7 activity in human^4^. Another circRNA in mouse, sex-determining region Y (Sry), which harbors 16 putative binding sites of miR-138, had been also regarded as miRNA sponge^16^. In addition, previous study had revealed that a subclass of circRNAs, EIciRNAs (exon-intron circRNAs), could interact with U1 snRNP to enhance transcription of the host-genes that they were derived from^18^. Furthermore, researches showed that the expression patterns of circRNAs exhibited developmental-specific, tissue-specific, and even cell type-specific in animals^11, 16, 19^. Thousands of circRNAs were enriched in neural tissues and progressively accumulated in adult CNS (Central Nervous System), which had been described as an aging biomarker in *Drosophila* ^11^. CircRNAs in mammals were highly enriched in synapses, and differentially expressed during neuronal differentiation^16^. CircRNAs were also found to be prevalent in many cancers and their expression level was closely related to clinical characteristics of tumor in human. Therefore, circRNAs could be putative disease biomarkers in cancer^20^. These findings indicate that circRNAs are important regulators and may represent another crucial layer of post-transcriptional control over gene expression.

Compared with the comprehensive study of circRNAs in animals, the systematic characterization of circRNAs in plants has received less attention^21^. Until recently, Ye *et al*. (2015) had identified 12,037 and 6,012 circRNAs in rice and *Arabidopsis thaliana*, respectively^22^. Lu *et al*. (2015) had also reported 2,354 circRNAs in *Oryza sativa* ^23^. Wang *et al*. (2017) had isolated 88 circRNAs in wheat^24^. Zuo *et al*. (2016) had found 854 circRNAs in tomato, of which 163 circRNAs showed chilling responsive expression^25^. In rice, some circRNAs were differential expression under Pi-sufficient and Pi-starvation conditions, suggesting that circRNAs may play a role in response to Pi starvation stress^22^. These findings imply that circRNAs are abundant in plants, and may have important function in response to abiotic stresses. Although some researches have been conducted, little is still known regarding circRNAs in plants. Further studies are still necessary.

Soybean (*Glycine max* L. Merr) is a leading oil and protein crop around the world. Soybean genome (approximately 1.1 Gb and 2n = 40) is an ancient tetraploid with a partially diploidized tetraploid. Soybean has undergone two whole genome duplication events approximately 59 and 13 million years ago, which cause that about 75% of the genes are paralogous genes with multiple copies^26^. To date, no studies have been conducted on soybean circRNAs, especially on the characteristics of circRNAs derived from paralogous genes. In current study, we systematically analyzed the circRNAs from different tissues of soybean using high-throughput sequencing technology and bioinformatic approaches. A total of 5,372 soybean circRNAs were identified and characterized. Besides that, the characteristics of circRNAs derived from paralogous genes were also investigated. Our results not only provided genome-wide profilings of circRNAs in soybean but also provided useful source and new insights into the plant circRNAs.

## Results

### RNA sequencing and identification of circRNAs in soybean

**Figure 1:**
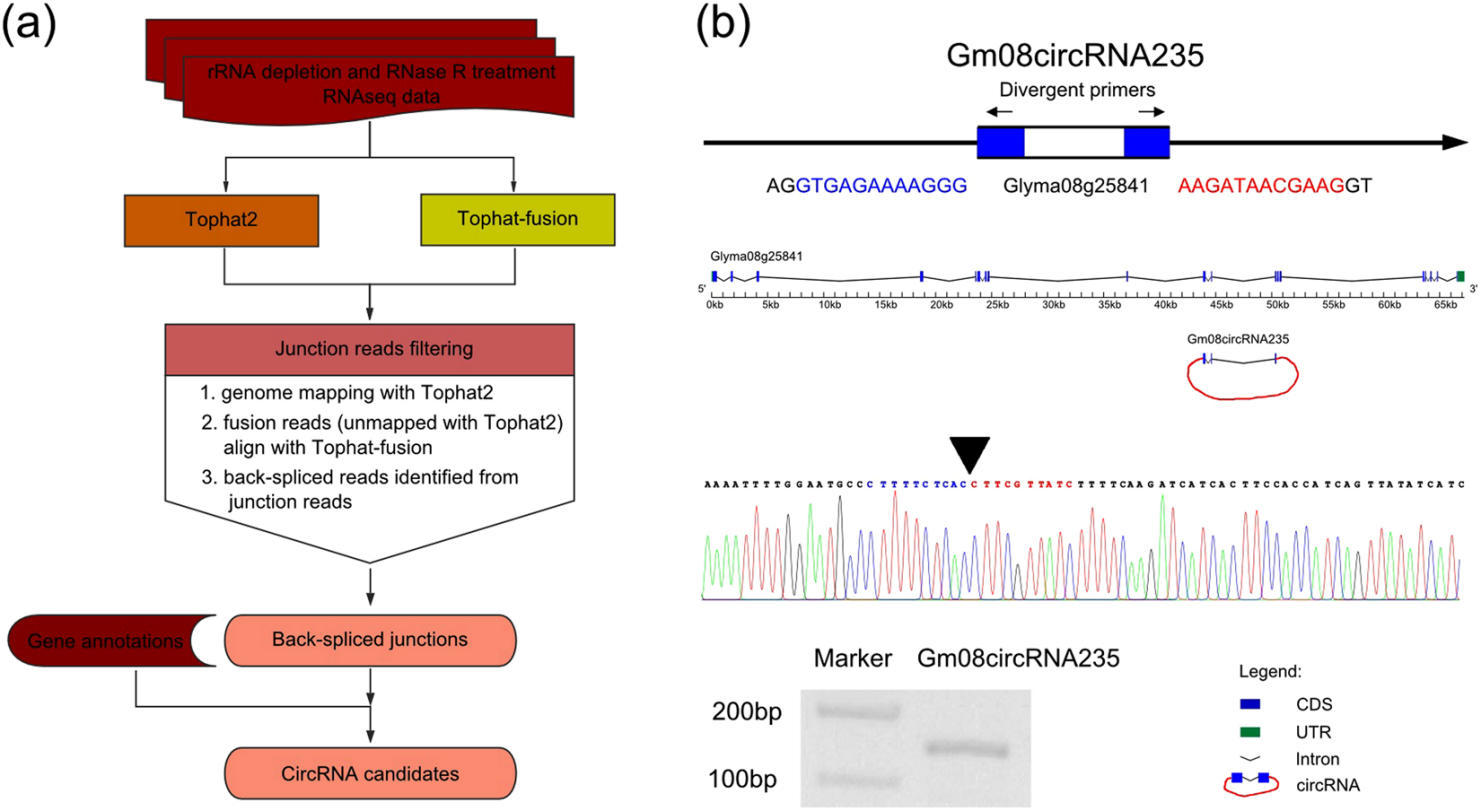
Pipline and validation method used for identification of circRNAs in soybean. (**a**) Pipline used for identification of circRNAs in soybean. All the steps were displayed in the form of the flowchart. (**b**) An example of validation strategy of circRNA. The upper part represented how the divergent primers were designed for PCR. In the middle, the parental gene structure and length scale were showed. Next part was the circRNA with back-spliced junction displayed by red trace line. The lower parts were the results of Sanger sequencing and agarose gel electrophoresis. The agarose gel electrophoresis image showed the expected size of PCR product, and Sanger sequencing were performed to confirm head-to-tail back-spliced site (black arrow). The flanking sequences of back-spliced site were marked by blue and red.

To explore soybean circRNAs on genome-wide level, we isolated RNAs from leaf, root and stem tissues of soybean (*Glycine max* L. Merr). After rRNA deleption and RNase R treatment, the remaining RNAs were used for library constructions, and then sequencing of the libraries were performed with an Illumina Hiseq 2500 analyzer. After trimming the adaptor sequences and filtering low-quality reads, a total of 61,610,358, 103,305,514 and 81,678,762 reads were generated from leaves, roots and stems, respectively (Supplementary Table S1). The clean reads with high quality were then subjected to an optimized pipline to identify circRNAs (Fig. 1a). After filtering the junction reads with non-canonical splice sites or cross genes alignments, a set of confident back-spliced junction reads including 2,911 from leaves, 6,899 from roots, and 7,416 from stems, were obtained for identification of circRNAs finally (Supplementary Table S2).

Based on the back-spliced junction reads, a total of 5,372 unique circRNAs were identified in soybean, of which, 776, 3,171 and 2,165 were from leaves, roots and stems, respectively (Table 1, Supplementary Table S3, Supplementary Dataset S1). Among the total circRNAs, 2,494 were exonic circRNAs that were generated from exons of single protein-coding genes, 2,581 were generated by introns, and 298 generated from intergenic regions (Table 1, Supplementary Table S3). The published tool CIRI was also used to analyze the sequencing data. By comparing the CIRI’s results with our predictions, we found that 1,058 of 5,372 circRNAs were commonly detected in both of two experiments, among which 836 were exonic circRNAs (Supplementary Table S3).

**Table 1:** Genome-wide identification of circRNAs in soybean.

Type of circRNA	Tissues			Total number
	Leaf	Root	Stem	
Exonic circRNA	515	1216	1274	2494
Intronic circRNA	201	1782	760	2581
Intergenic circRNA	60	173	131	297
Total number	776	3171	2165	5372

To confirm the predicted results, divergent primers were designed for ten randomly selected circRNAs to perform polymerase chain reaction (PCR) (Supplementary Table S4). The PCR amplified products were further analyzed by agarose gel electrophoresis and Sanger sequencing (Fig. 1b). The results showed that 70% circRNAs had bands of expected size and validated back-spliced junction sites (Supplementary Fig. S1).

### Properties of soybean circRNAs

**Figure 2:**
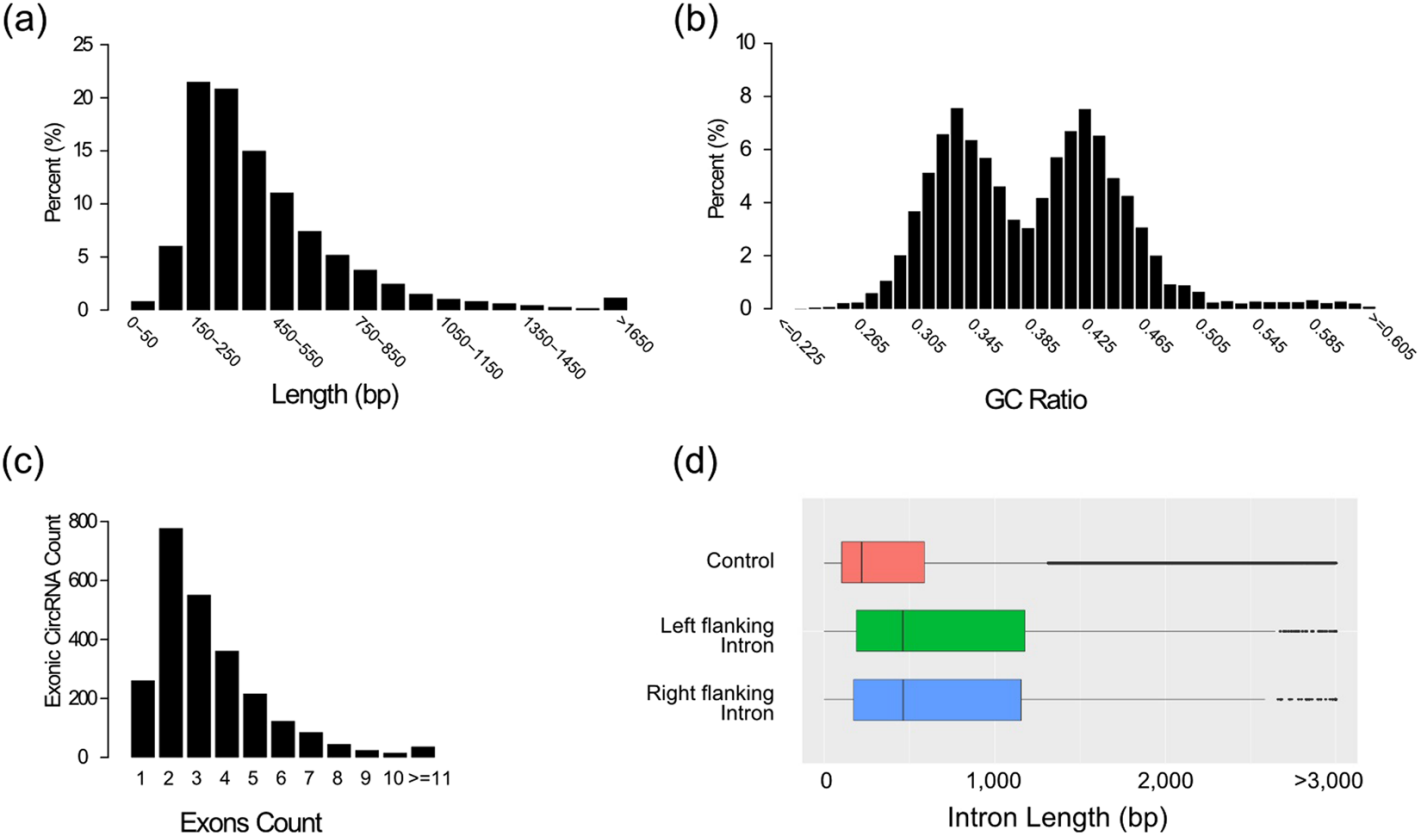
Sequence features of circRNAs in soybean. (**a**) Distribution of length of circRNAs in soybean. (**b**) Distribution of GC ratio of circRNAs in soybean. (**c**) Number of exonic circRNAs that contained different number of exons derived from parental genes in soybean. (**d**) Distribution of length of the flanking introns that bracketing exonic circRNAs in soybean.

To uncover unique features of circRNAs in soybean, we had investigated the properties of circRNAs identified from different tissues of soybean. The soybean circRNAs were mainly between 150–600 bp in length, and only a few were more than 2,000 bp (Fig. 2a). The mean length of exonic circRNAs, intronic circRNAs and intergenic circRNAs was 521 bp, 464 bp and 391 bp, respectively (Supplementary Fig. S2). The GC ratio of soybean circRNAs had a double peaks spanned from 0.3 to 0.4 and from 0.4 to 0.5 (Fig. 2b). Meanwhile, the GC ratio of intronic circRNAs, exonic circRNAs and intergenic circRNAs were mainly spanned from 0.3 to 0.4, 0.4 to 0.5 and 0.3 to 0.6, respectively (Supplementary Fig. S2).

Based on the gene structure annotations of soybean, 78.2% of exonic circRNAs harbored one to four exons derived from parental genes, whereas 56.3% of their parental genes had more than ten exons (Fig. 2c, Supplementary Table S3). We extracted the flanking intron sequences of exonic circRNAs from soybean genome sequence for further analysis. Compared with the average length of introns of soybean, the length of the flanking introns of exonic circRNAs was longer (Fig. 2d). In addition, the results of alignment between the right and the left flanking intron sequences of exonic circRNAs using blastn (v2.2.27) with e-value setting at 1e^−5^ revealed that only 2.7% of intronic sequences contained reverse complementary sequences in soybean.

**Figure 3:**
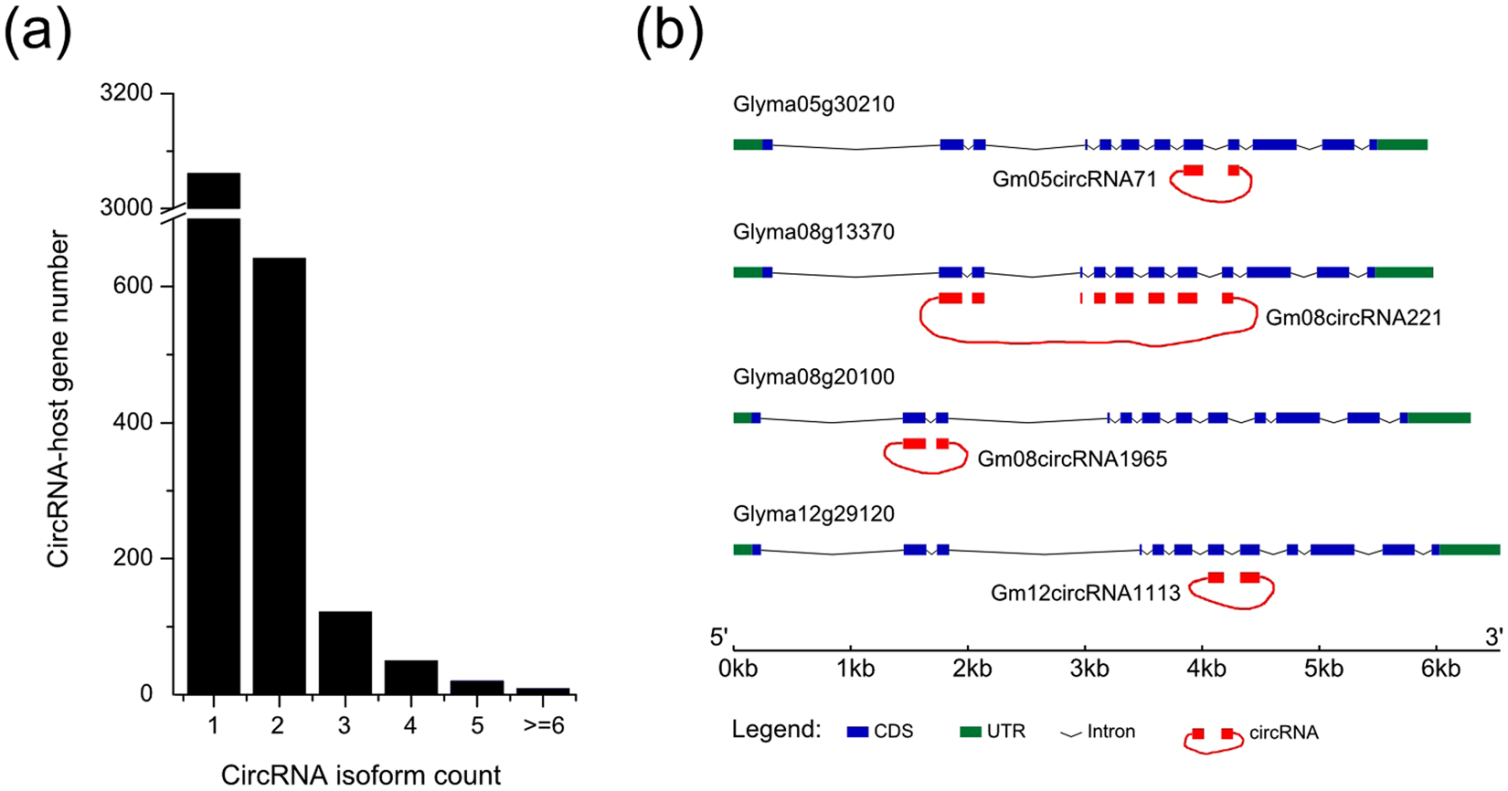
CircRNA-host gene and paralogous circRNAs in soybean. (**a**) Number of circRNA-host genes that generated different count of circRNAs in soybean. (**b**) A schematic plot of paralogous circRNAs. Paralogous circRNAs marked as red were showed below the structure chart of each paralogous gene.

Of the 3,904 circRNA-host genes, approximately 80% could generate only one form of circRNA, and few genes could generate more than six different isoforms of circRNAs (Fig. 3a, Supplementary Table S3). The circRNAs were also analyzed by use of the tool CIRI_AS and the results showed that 43 circRNAs exhibited alternative splicing (AS) events (Supplementary Table S5). The different isoforms of circRNAs were results of alternative back-spliced circularization, and circRNA-host genes with alternative circularization preferentially contained more exons than those genes with non-alternative circularization in soybean (Supplementary Fig. S3).

Soybean is a partially diploidized tetraploid, and has undergone two whole genome duplication events, which result in approximately 75% of soybean genes are multiple copies^26^. These multiple copies of genes are usually paralogous genes, which share homologous sequences, structural similarities and functional redundancy in soybean. Our results showed that the paralogous genes in soybean could produce different circRNAs, named as paralogous circRNAs in this study. For example, each of the paralogous genes (*Glyma05g30210*, *Glyma08g13370*, *Glyma08g20100* and *Glyma12g29120*) could generate one circRNA, and these paralogous circRNAs were originated from different exons of paralogous circRNA-host genes (Fig. 3b). In soybean, 4,451 (82.8% of total circRNAs) circRNAs were generated from the paralogous genes (Supplementary Table S6).

To explore whether circRNAs were conserved in different plant species, we further compared the circRNAs derived from the orthologous genes of *Arabidopsis thaliana*, *Oryza sativa* and soybean. The circRNAs sets of *Arabidopsis thaliana* and *Oryza sativa* were obtained from the PlantcircBase^27^. The orthologous gene set in *Arabidopsis thaliana*, *Oryza sativa* and soybean was generated by BioMart from EnsemblPlants database. Among the 8,362, 9,385 and 1,995 parental genes that produced exonic circRNAs in *Arabidopsis thaliana*, *Oryza sativa* and soybean, respectively, 685 gene pairs with high confidence between soybean and *Arabidopsis thaliana*, and 1,095 gene pairs with high confidence between soybean and *Oryza sativa* were orthologs (Supplementary Table S7). Furthermore, 551 gene pairs with high confidence among the three species were orthologs, which suggested that circRNAs exhibited conservation feature among plant kingdom.

### Expression patterns of soybean circRNAs

**Figure 4:**
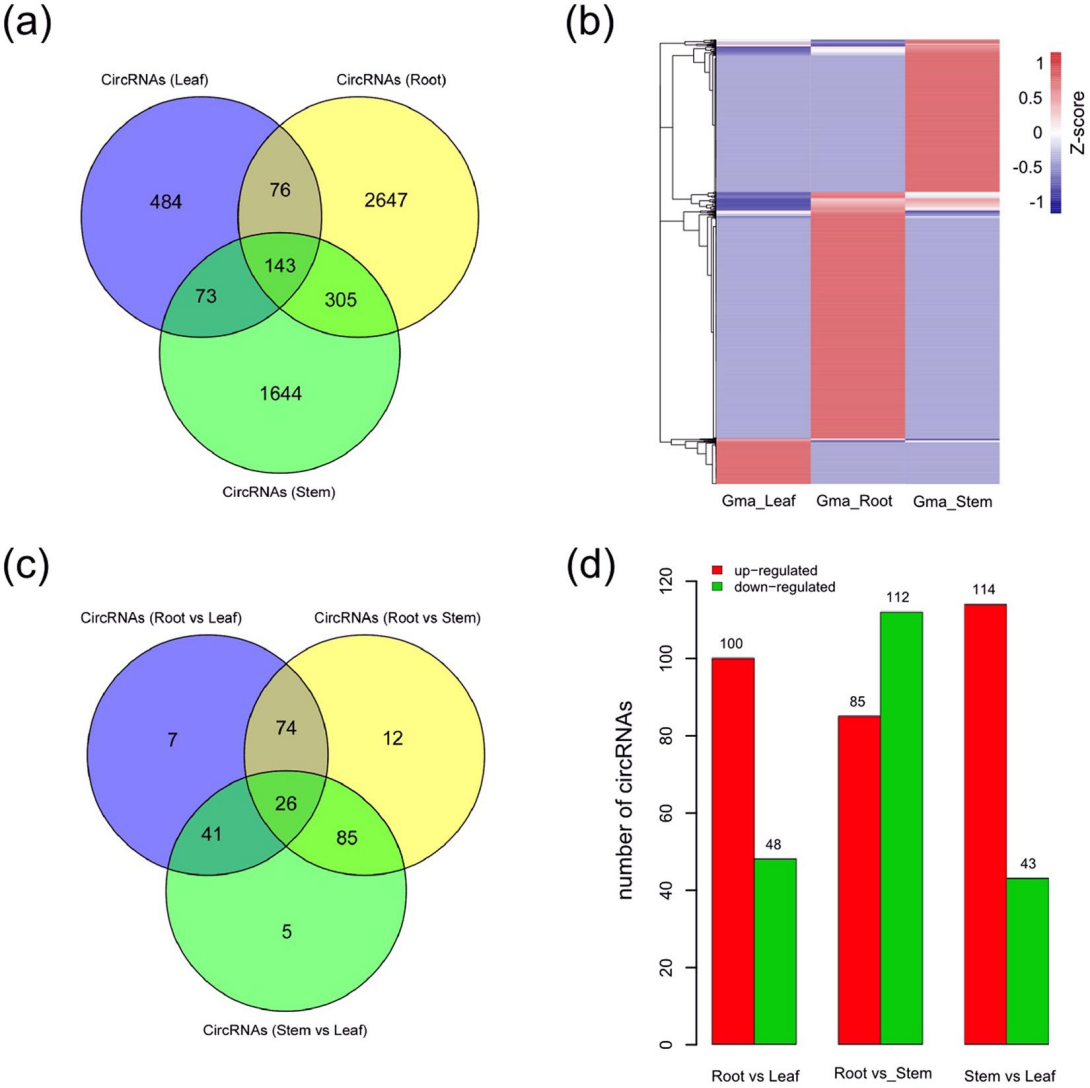
Tissue specific expression patterns of circRNAs in soybean. (**a**) Venn diagram showing the number of tissue-preferentially expressed circRNAs in each tissue of soybean. (**b**) Heatmap showing the expression patterns for all the circRNAs identified in soybean. Vertical columns represented different tissues of soybean. Horizontal rows represented circRNAs. Color scale representing Z-score was given at the left. (**c**) Venn diagram showing the number of differentially expressed circRNAs in each tissue. (**d**) Histogram showing differentially expressed circRNAs among tissues of soybean. The number of up-regulated (red) and down-regulated (green) circRNAs was displayed at the top of each bar.

To investigate the expression patterns of soybean circRNAs, the FPKM (fragments per kilobase of transcripts per million mapped reads) value were calculated for each circRNA using Cufflinks (V2.1.1) in different tissues of soybean. Approximately 62.4% (484) of leaf circRNAs, 83.5% (2,647) of root circRNAs and 72.2% (1,563) of stem circRNAs were tissue specific, whereas only 2.7% (143) of the total circRNAs were commonly expressed in all the three tissues of soybean (Fig. 4a). Meanwhile, hierarchical cluster analysis of circRNAs from leaf, root and stem also revealed that circRNAs exhibited specific expression patterns in different tissues of soybean (Fig. 4b). Similarly, the paralogous circRNAs of soybean were tissue-specific expression as well (Supplementary Fig. S4).

Among the circRNAs identified in soybean, a total of 250 were differentially expressed, 26 of which showed constitutive differential expression in different tissues (Fig. 4c). The intercomparison analysis showed that 148 circRNAs were differentially expressed between roots and leaves with 100 up-regulated and 48 down-regulated, 197 were differentially expressed between roots and stems with 85 up-regulated and 112 down-regulated, and  157 were differentially expressed between stems and leaves with 114 up-regulated and 43 down-regulated (Fig. 4d). These differentially expressed circRNAs may have specific functions in the tissue differentiation in soybean.

### Functional annotation of soybean circRNAs

To explore the putative function of soybean circRNAs, GO categories and KEGG pathway analyses were performed on the 3,904 circRNA-host genes. For the molecular function, the enriched GO terms included nucleotide binding, ATP binding, catalytic activity, protein binding, nucleic acid binding and mRNA processing (Table 2). For the biological process, the circRNA-host genes were mainly involved in developmental process, multi-organism process, reproduction, response to stimulus, metabolic process and cellular process (Supplementary Fig. S5). The KEGG pathway analysis showed that the circRNA-host genes were significantly enriched in seven pathways, including pathways for citrate cycle, aminoacyl-tRNA biosynthesis, pyrimidine metabolism, glycolysis/gluconeogenesis, glycerophospholipid metabolism, propanoate metabolism, and oxidative phosphorylation (Table 3).

**Table 2:** GO categories of circRNA-host genes in soybean.

GO ID^*^	Functional category	Gene Count	P-value
GO:0000166	nucleotide binding	485	1.16E-22
GO:0005524	ATP binding	584	6.40E-22
GO:0005488	binding	132	1.67E-13
GO:0006886	intracellular protein transport	77	2.76E-08
GO:0005515	protein binding	680	3.35E-08
GO:0008017	microtubule binding	45	2.83E-07
GO:0005737	cytoplasm	119	4.89E-07
GO:0004386	helicase activity	57	6.02E-07
GO:0000155	phosphorelay sensor kinase activity	19	7.53E-07
GO:0023014	signal transduction by protein phosphorylation	19	9.67E-07
GO:0016874	ligase activity	60	1.16E-06
GO:0015031	protein transport	64	1.86E-06
GO:0008536	Ran GTPase binding	14	2.57E-06
GO:0005874	microtubule	43	2.75E-06
GO:0003723	RNA binding	96	3.75E-06
GO:0017111	nucleoside-triphosphatase activity	84	4.32E-06
GO:0006397	mRNA processing	22	5.31E-06
GO:0036459	ubiquitinyl hydrolase activity	20	6.25E-06
GO:0003824	catalytic activity	273	7.67E-06
GO:0003676	nucleic acid binding	218	8.26E-06

^*^Top 20 GO terms with the threshold of P-value < 0.05 were listed.

**Table 3:** KEGG pathway enrichment of circRNA-host genes in soybean.

Pathway ID^*^	Description	Gene Count	P-value
ko00020	Citrate cycle (TCA cycle)	23	0.001
ko00970	Aminoacyl-tRNA biosynthesis	19	0.003
ko00240	Pyrimidine metabolism	36	0.006
ko00010	Glycolysis/Gluconeogenesis	37	0.016
ko00564	Glycerophospholipid metabolism	19	0.035
ko00640	Propanoate metabolism	11	0.041
ko00190	Oxidative phosphorylation	10	0.048

^*^Pathway with the threshold of P-value < 0.05 was listed.

**Figure 5:**
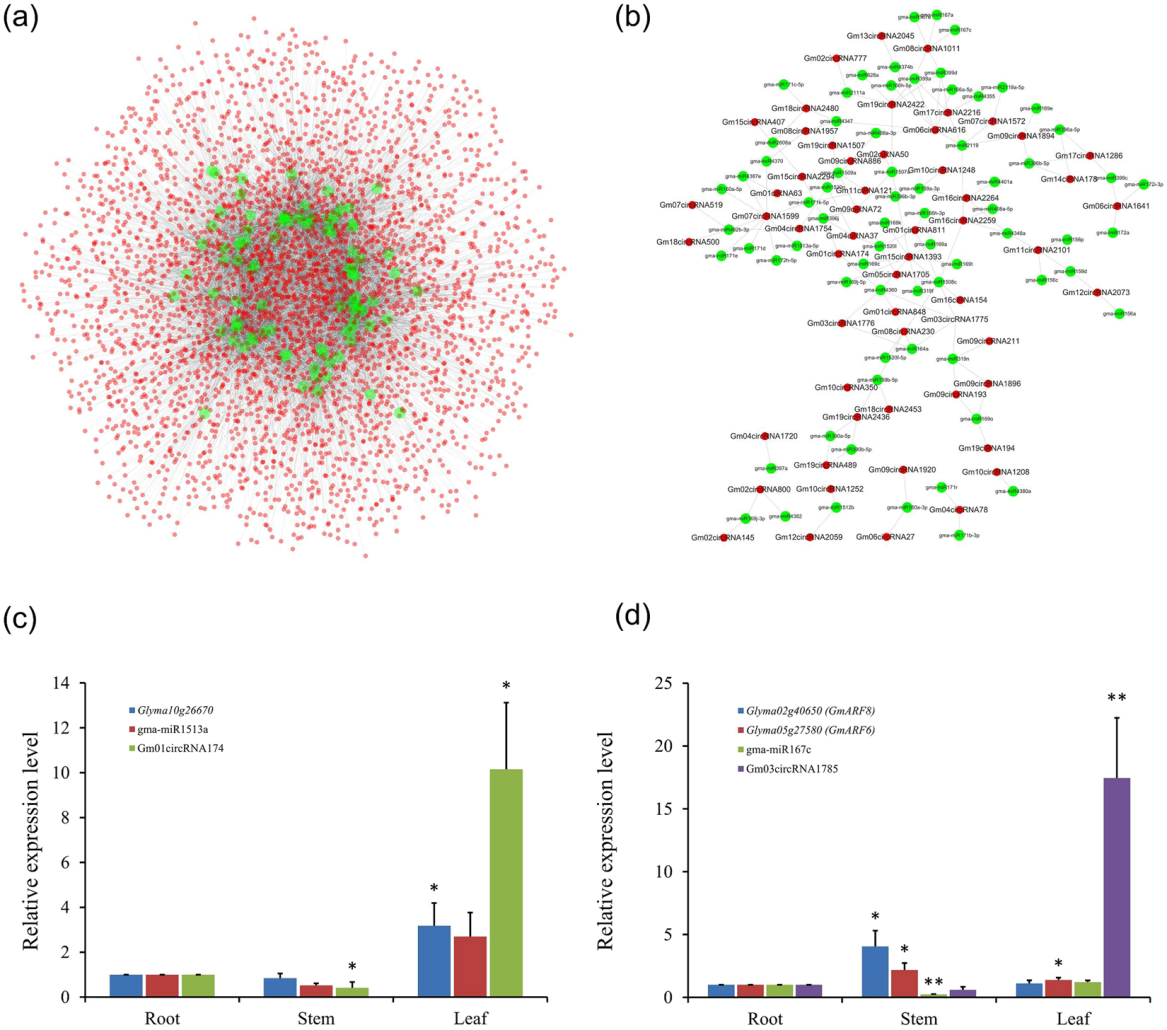
CircRNA-miRNA interaction network in soybean. (**a**) The panorama network consists of 2,134 circRNAs (red circle) and 92 miRNAs (green circle) in soybean. They were connected by 5,648 edges based on seed sequence pairing interactions. (**b**) A subnetwork comprising the 57 differentially expressed circRNAs (red circle) and their target miRNAs (green circle) in soybean. (**c**) Expression analysis of Gm01circRNA174, gma-miR1513a and *Glyma10g26670* in different tissues of soybean. (**d**) Expression analysis of Gm03circRNA1785, gma-miR167c and *GmARF6/GmARF8* in different tissues of soybean. All experiments were conducted three times. Error bars indicate SD. Stars above bar indicate the significant difference as follows: **(P < 0.01) and *(P < 0.05), comparing to that of root.

Recent studies have demonstrated that circRNAs could bind miRNAs to repress them from targeting mRNAs and therefore regulate gene expression^4, 9^. To uncover whether circRNAs in soybean could target miRNAs and further affect the post-transcriptional regulation of genes, the sequences of circRNAs were used to identify potential binding sites of miRNAs. In total, 2,134 (39.7%) circRNAs contained predicted binding sites for 92 miRNAs. Of these circRNAs, only 352 had two to six miRNA binding sites (Supplementary Table S8). Some well-known miRNAs such as miR156, miR172, miR160, miR398 and miR399 were all predicted to be targeted by specific circRNAs (Supplementary Table S8). Based on the interaction theoretically predicted by conserved seed-matching sequence between circRNAs and miRNAs, an entire circRNA-miRNA interaction network was delineated by Cytoscape (Fig. 5a). Furthermore, the part of the circRNA/miRNA interaction illustration was enlarged to display the differentially expressed circRNAs and their target miRNAs (Fig. 5b). The circRNA-miRNA interaction network showed that a single miRNA could be targeted by various circRNAs, whereas a single circRNA could also target different miRNAs. For example, 119 circRNAs were predicted to target gma-miR396j, 85 circRNAs could target gma-miR397a, and 44 circRNAs could bind gma-miR169a in soybean (Supplementary Fig. S6).

We conducted qRT-PCR to validate the interaction of circRNAs, miRNAs and target genes. Two circRNAs, Gm01circRNA174 and Gm03circRNA1785, were selected. Gm01circRNA174 was predicted to target gma-miR1513a, and gma-miR1513a was predicted to target *Glyma10g26670*, an F-box contained gene^28^. The qRT-PCR results showed that, in comparison with root tissue, the expression of gma-miR1513a was not significantly changed, while both Gm01circRNA174 and *Glyma10g26670* were significantly up-regulated in leaf tissue. These patterns suggested a potential regulating mechanism among Gm01circRNA174, gma-miR1513a and *Glyma10g26670*, in which up-regulation of Gm01circRNA174 might decrease the activity of gma-miR1513a and increase the expression of *Glyma10g26670* (Fig. 5c). However, such expression patterns were not identified in stem tissue (Fig. 5c). Gm03circRNA1785 was predicted to target gma-miR167c and gma-miR167c was predicted to target two auxin response factors, *GmARF6* (*Glyma05g27580*) and *GmARF8* (*Glyma02g40650*), which played important roles in nodulation and lateral root development in soybean^29^. The qRT-PCR results indicated that, in comparison with root, the expression of gma-miR167c was not significantly changed, while Gm03circRNA1785 and *GmARF6* were significantly up-regulated in leaf tissue (Fig. 5d), suggesting a potential regulating mechanism among these three genes too. Interestingly, for both of these two circRNAs, the positively correlated expression patterns between circRNA and mRNA were not identified in stem tissue, indicating the tissue specific features for the regulating mechanisms among circRNA, miRNA and mRNA.

## Discussion

In the past years, circRNAs were once considered to be RNA splicing errors, and regarded as some form of transcriptional noise or RT-PCR artifacts^4^. Recent studies had identified abundant of circRNAs in mammals, and demonstrated that natural circRNAs were important regulators in animals^9, 10, 30^. However, compared with animal circRNAs, the knowledge of plant circRNAs is still limited^22–25^.

In current study, we reported the first genome-wide identification and characterization of circRNAs in soybean, which is the leading oil and protein crop with partially diploidized tetraploid genome. In total, 5,372 unique circRNAs were identified from different tissues of soybean, including 776, 3,171 and 2,165 from leaves, roots and stems, respectively (Table 1). The circRNAs identified in soybean was less than that in *Oryza sativa* (12,037) and *Arabidopsis thaliana* (6,012)^22^. The reason might be that, in our study, we used an experimental strategy of high-throughput sequencing coupled with RNase R enrichment, while, in *Oryza sativa* and *Arabidopsis thaliana*, the bioinformatic method were mainly used^22^. Besides, since only three soybean tissues including leaf, root and stem, were analyzed, the actual number of circRNAs might be underestimated. Previous studies revealed that circRNA prediction tools, such as CIRI, find_circ and CIRCexplorer, yielded highly divergent results, and 16.8% circRNAs were observed between different prediction algorithms^31^. In our study, of all the circRNAs identified, approximately 19.7% (1,058) were also detected by CIRI, which could be regarded as more reliable circRNAs. Interestingly, there were 2,581 intronic circRNAs detected in soybean (Table 1), which was much more than 1 in *Arabidopsis thaliana* and 485 in *Oryza sativa* ^22^. This indicated that soybean introns could generate more circRNAs, which might be attributed to the large and duplicated genome and multiple copies of genes in soybean.

Previous studies had demonstrated that exonic circRNAs in animals were typically bracketed by long introns which highly contained reverse complementary sequences^1, 12^. However, unlike the animal circRNAs, most of plant circRNAs had limited repetitive and reverse complementary sequences in intronic sequences flanking exonic circRNAs. For example, the proportion of reverse complementary sequences was 6.2% and 0.3% in *Oryza sativa* and *Arabidopsis thaliana*, respectively^22^. Here, our study showed that the proportion in soybean was 2.7%, which was less than that in *Oryza sativa*, but more than *Arabidopsis thaliana*. These findings implied that plants might harbor different mechanisms of circRNA biogenesis from animals. Besides, soybean and *Arabidopsis thaliana* are dicot plants, while *Oryza sativa* is monocot plant. Thus, the biogenesis of circRNAs might diverse between dicot and monocot plants.

In *Oryza sativa*, 39.0% of circRNA parental genes had more than ten exons^23^. Similarly, our results showed that the proportion in soybean was approximately 56.3%, which suggested that circRNAs were preferentially originated from the genes with multiple exons in soybean and *Oryza sativa* (Supplementary Table S3). However, whether this phenomenon was prevailing in plants need to be investigated further.

The expression profiles of circRNAs in rice, tomato and *Arabidopsis thaliana* revealed that circRNAs exhibited developmental stage specific and abiotic stress responsive expression patterns^22, 25^. In our study, the majority of soybean circRNAs were specific expressed in different tissues (Fig. 4a,b). Moreover, a variety of circRNA isoforms generated by alternative circularization, were also tissue-preferentially expressed in soybean (Supplementary Fig. S3). Consequently, this might be indicative of important functionality of circRNAs and their isoforms during the tissue differentiation in plants.

Unlike *O. sativa* and *A. thaliana*, soybean has undergone two whole genome duplication events, which caused that 75% of soybean genes are paralogous genes with multiple copies^26^. Our data revealed that over 80% of soybean circRNAs were paralogous circRNAs generated from paralogous genes (Supplementary Table S6). Although the paralogous genes of soybean had high homology sequences, their paralogous circRNAs could vary a lot. Thus, new mechanisms of paralogous circRNAs biogenesis might be generated during the evolutionary process of soybean. Furthermore, these paralogous circRNAs showed different expression level or specific expression in different tissues, which suggested the paralogous circRNAs might play different function roles from each other in soybean.

CircRNA was reported to bind specific miRNAs to prohibit them from regulating their target genes^4, 30^. For example, the ciRS-7/CDR1as circRNA harbored more than 70 miR-7 binding sites, and Sry circRNA contained 16 putative binding sites for miR-138, both of which could act as miRNA sponge or decoys to regulate the expression of functional genes by competitive binding miRNAs. However, since that only few circRNAs contained a substantial number of miRNA binding sites, it was currently debated whether miRNA inhibition was a general feature of circRNAs^8^. In our data, although some putative binding sites of miRNAs had been identified in the sequences of circRNAs, no miRNA sponge for a single circRNA was observed in soybean (Supplementary Table S8). Notably, a number of circRNAs could target one common miRNA (Supplementary Fig. S6), thus we tentatively put forward that various circRNAs containing common miRNA binding sites might act as miRNA sponge together, to regulate the activity of target genes in soybean.

Some well-known miRNA families of MIR156^32, 33^, MIR172^34, 35^, MIR160^36^, MIR398^37, 38^, and MIR399^39^ were predicted to be targeted by certain soybean circRNAs. Besides, we noticed that some miRNAs, such as miR482, miR1512, and miR1515^40^, which were related to soybean nitrogen fixation, were the putative targets of circRNAs in soybean. Furthermore, the circRNA-miRNA interaction network showed that circRNAs were important members of ceRNAs (competing endogenous RNAs), and could competitively bind miRNAs (Fig. 5a). Thus, the circRNAs containing miRNA binding sites were potential post-transcriptional regulators, and might participate in diverse biology processes by interacting with miRNAs. We selected two circRNAs, Gm01circRNA174 and Gm03circRNA1785, and their corresponding miRNAs and mRNAs to detect their expression patterns in root, stem and leaf, respectively. The results showed that, in leaf tissue, expression of circRNA and mRNA showed positively correlated patterns, while in stem tissue, such patterns were not found, which indicated that the regulation mechanism among circRNA, miRNA and mRNA might be tissue specific. *GmARF6* (*Glyma05g27580*) and *GmARF8* (*Glyma02g40650*) were reported to be involved in nodulation and lateral root development in soybean^29^, which suggested that the regulating module among Gm03circRNA1785, gma-miR167c and *GmARF6* and *GmARF8* might play important roles in the activities of soybean life. This module are worthy of being studied in the future. However, there was still 60.3% of the soybean circRNAs were predicted to have no miRNA binding sites, which indicated that these circRNAs might have different functions from miRNA sponges in soybean.

In this study, we explored the abundant and characteristics of circRNAs from leaf, root and stem tissues of soybean using high-throughput sequencing technology. In addition to the identification of circRNAs, the features, expression patterns and their functions were also investigated. Noticeably, we characterized the paralogous circRNAs derived from paralogous genes in soybean for the first time. Nevertheless, it should be noted that only three tissues were included, which might lead to limitation of some properties of soybean circRNAs. Future studies about circRNAs from more tissues of various developmental stages or under abiotic/biotic stress will be helpful to solve the problems. Our study not only expanded the knowledge of circRNAs in plant kingdom, but also provided useful clues for understanding circRNAs in the evoluation of polyploidy.

## Methods

### Plant materials

The seeds of soybean (*Glycine max* L. Merr) were germinated at 28 °C in an incubator. The whole plant were grown in a 28 °C greenhouse under a 16:8 (light: dark) photoperiod. Stems, roots and mature leaves of soybean were collected at the trefoil stage, and then immediately frozen in liquid nitrogen and stored at −80 °C.

### Libraries construction and SBS sequencing

The total RNAs were isolated from the root, leaf and stem tissues using TRIZOL reagent (Invitrogen, CA, USA) following the manufacturer’s procedure. The total RNA concentration and purity were assayed with a NanoDrop ND-1000 spectrophotometer (NanoDrop Technologies, Wilmington, DE, USA). The RNA integrity was assessed on an Agilent 2100 Bioanalyzer Lab-on-Chip system (Agilent Technologies, Palo Alto, CA, USA). Approximately 10 µg of the total RNA was used to deplete ribosomal RNA according to the manuscript of the Epicentre Ribo-Zero Gold Kit (Illumina, San Diego, USA). The rRNA-depleted RNAs were further incubated at 37 °C for 1 hour in 16 μl reaction with 10U/μg RNase R (Epicentre, Madison, WI). The remaining RNAs were used as templates for the construction of cDNA libraries in accordance with the protocol for the mRNA-Seq sample preparation kit (Illumina, San Diego, USA). The clustering of samples was performed on a cBot Cluster Generation System using TruSeq PE Cluster Kit v3-cBot-HS (Illumia, San Diego, USA) according to the manufacturer’s instructions. And the paired-end sequencing was performed on the Illumina Hiseq2500 platform.

### Computational identification of circRNAs

Soybean genome sequence and gene annotations were downloaded from EnsemblPlants database (http://plants.ensembl.org/Glycine_max/Info/Index). The raw reads were filtered through initially trimming for adapter sequences and removing the low quality reads. Then, the clean reads with high quality were used to identify circRNAs using an optimized pipline described in Fig. 1a. Briefly, the clean reads from each sample were mapped onto soybean genome sequence using Tophat2 (v2.1.0)^41^. Then, the unmapped reads were extracted and further aligned with soybean reference sequence by Tophat-fusion software^42^. The junction reads with non-colinear ordering alignment on the same chromosome were regarded as candidate back-spliced junction reads. The candidate back-spliced junction reads were realigned against gene annotations of soybean to verify the splice sites. The junction reads with non-canonical splice sites or cross genes alignments were discarded, and the remaining confident back-spliced junction reads were used for identification of circRNAs. A candidate circRNA was called if it was supported by at least two unique back-spliced reads. Furthermore, CIRI software was used to detect circRNAs, and CIRI-AS was employed to detect alternative splicing events in circRNAs^43, 44^.

### Expression analysis and circRNA-miRNA interaction network construction

The transcriptome reads were mapped onto soybean genome by Tophat2 (v2.1.0) with the default settings^41^. Quantification of the expression level of circRNAs was performed using the FPKM (fragments per kilobase of transcripts per million mapped reads) algorithm. Differential expression of circRNAs was profiled with the DEseq R package^45^. P-values were adjusted using the Benjamini and Hochberg’s approach. CircRNAs with P-value ≤ 0.05 and |log_2_ (foldchange)| ≥ 1 were regarded as differential expression by default.

The miRanda^46^ and Targetscans (V7.0)^47^ were used to predict miRNA binding sites of soybean circRNAs upon the alignment against miRBase21.0 (http://www.mirbase.org/)^48^. Base on the interaction theoretically predicted by conserved seed-matching sequence between circRNAs and miRNAs, the graph of the circRNA-miRNA interaction network was visualized using Cytoscape 3.4.0^49^.

### GO categories and KEGG pathway analyses

A Gene Ontology (GO) enrichment analysis was used on the circRNA-host genes with the GOseq R packages based on the Wallenius non-central hyper-geometric distribution^50^. The KOBAS software was used to test the statistical enrichment of the circRNA-host genes in the KEGG pathways (http://www.genome.jp/kegg/)^51, 52^.

### Validation of circRNAs in soybean

To confirm the circRNAs predicted in soybean, a set of divergent primers were designed on the flanking sequences of head-to-tail splicing sites of circRNAs (Supplementary Table S3). Polymerase chain reactions (PCRs) were done using these divergent primers and cDNA templates. The PCR procedure was as following: 94 °C for 3 min, 35 cycles at 94 °C for 45 s, 58 °C for 35 s, and 72 °C for 30 s. The final step was at 72 °C for 10 min. PCR products were separated using AGE (agarose gel electrophoresis), and purified with QIAquick Gel Extraction Kit (Qiagen, CA, USA). Sanger sequencing were performed to further confirm the presence of the back-spliced junction sites.

### Quantitative real-time PCR

To confirm the predicted results, qRT-PCR detection was performed to evaluate the expression levels of circRNAs, miRNAs and target genes in different tissues of soybean using a SYBR Green PCR kit (GeneCopoeia, Inc. Rockville, MD, USA) with ViiA™ 7 Dx platform (ABI, USA). The amplified primers and internal controls were listed in Supplementary Table S3. The qRT-PCR procedure of circRNAs and target genes was as following: 95 °C for 30 s, 40 cycles at 95 °C for 5 s, 58 °C for 30 s and 72 °C for 30 s. The qRT-PCR procedure of miRNAs was as following: 95 °C for 10 min, 40 cycles at 95 °C for 10 s, 56 °C for 20 s and 72 °C for 20 s. After qRT-PCR amplification, the melting curve and amplification curve were examined in order to evaluate specific amplification. The relative expression levels were analyzed by 2^−ΔΔct^ method. U6 was used as the internal control for miRNAs. *SKIP* was used as the internal control for circRNAs and target genes. All the qRT-PCR reactions were assayed in triplicates.

## Electronic supplementary material

Supplementary Figures and Tables

Supplementary Dataset S1

Supplementary Table S3

Supplementary Table S5

Supplementary Table S6

Supplementary Table S7

Supplementary Table S8

## References

[1] Jeck WR (2013). Circular RNAs are abundant, conserved, and associated with ALU repeats. RNA.

[2] Chen LL, Yang L (2015). Regulation of circRNA biogenesis. RNA Biol..

[3] Suzuki H, Tsukahara T (2014). A View of Pre-mRNA Splicing from RNase R Resistant RNAs. Int. J. Mol. Sci..

[4] Memczak S (2013). Circular RNAs are a large class of animal RNAs with regulatory potency. Nature.

[5] Gao Y, Wang J, Zhao F (2015). CIRI: an efficient and unbiased algorithm for de novo circular RNA identification. Genome Biol..

[6] Szabo L (2015). Statistically based splicing detection reveals neural enrichment and tissue-specific induction of circular RNA during human fetal development. Genome Biol..

[7] Song X (2016). Circular RNA profile in gliomas revealed by identification tool UROBORUS. Nucleic Acids Res.

[8] Jeck WR, Sharpless NE (2014). Detecting and characterizing circular RNAs. Nat. Biotechnol..

[9] Fan X (2015). Single-cell RNA-seq transcriptome analysis of linear and circular RNAs in mouse preimplantation embryos. Genome Biol..

[10] Salzman J, Gawad C, Wang PL, Lacayo N, Brown PO (2012). Circular RNAs are the predominant transcript isoform from hundreds of human genes in diverse cell types. PLoS One.

[11] Westholm JO (2014). Genome-wide Analysis of *Drosophila* Circular RNAs Reveals Their Structural and Sequence Properties and Age-Dependent Neural Accumulation. Cell Rep..

[12] Zhang XO (2014). Complementary Sequence-Mediated Exon Circularization. Cell.

[13] Ivanov A (2015). Analysis of intron sequences reveals hallmarks of circular RNA biogenesis in animals. Cell Rep.

[14] Barrett SP, Wang PL, Salzman J (2015). Circular RNA biogenesis can proceed through an exon-containing lariat precursor. Elife.

[15] Ashwal-Fluss R (2014). CircRNA biogenesis competes with pre-mRNA splicing. Mol. Cell.

[16] Rybak-Wolf A (2015). Circular RNAs in the Mammalian Brain Are Highly Abundant, Conserved, and Dynamically Expressed. Mol. Cell.

[17] Conn SJ (2015). The RNA Binding Protein Quaking Regulates Formation of circRNAs. Cell.

[18] Li Z (2015). Exon-intron circular RNAs regulate transcription in the nucleus. Nat. Struct. Mol. Biol..

[19] Salzman J, Chen RE, Olsen MN, Wang PL, Brown PO (2013). Cell-Type Specific Features of Circular RNA Expression. PLoS Genet..

[20] Li Y (2015). Circular RNA is enriched and stable in exosomes: a promising biomarker for cancer diagnosis. Cell Res..

[21] Sablok G, Zhao H, Sun X (2016). Plant Circular RNAs (circRNAs): Transcriptional Regulation Beyond miRNAs in Plants. Mol. Plant.

[22] Ye CY, Chen L, Liu C, Zhu QH, Fan L (2015). Widespread noncoding circular RNAs in plants. New Phytol..

[23] Lu T (2015). Transcriptome-wide investigation of circular RNAs in rice. RNA.

[24] Wang, Y. *et al*. Identification of Circular RNAs and Their Targets in Leaves of *Triticum aestivum* L. under Dehydration Stress. *Front. Plant Sci*. **7**, 2024, 10.3389/fpls.2016.02024. eCollection (2017). 10.3389/fpls.2016.02024 PMC5215283 28105043

[25] Zuo J, Wang Q, Zhu B, Luo Y, Gao L (2016). Deciphering the roles of circRNAs on chilling injury in tomato. Biochem. Biophys. Res. Commun..

[26] Xu D (2010). Genome sequence of the palaeopolyploid soybean. Nature.

[27] Chu Q (2017). PlantcircBase: A Database for Plant Circular RNAs. Mol Plant.

[28] Kulcheski FR (2011). Identification of novel soybean microRNAs involved in abiotic and biotic stresses. BMC Genomics.

[29] Wang Y (2015). MicroRNA167-Directed Regulation of the Auxin Response Factors *GmARF8a* and *GmARF8b* Is Required for Soybean Nodulation and Lateral Root Development. Plant Physiol..

[30] Hansen TB (2013). Natural RNA circles function as efficient microRNA sponges. Nature.

[31] Hansen TB, Venø MT, Damgaard CK, Kjems J (2016). Comparison of circular RNA prediction tools. Nucleic Acids Res..

[32] Cui LG, Shan JX, Shi M, Gao JP, Lin HX (2014). The miR156-SPL9-DFR pathway coordinates the relationship between development and abiotic stress tolerance in plants. Plant J..

[33] Cao D (2015). GmmiR156b overexpression delays flowering time in soybean. Plant Mol. Biol..

[34] Yan Z (2013). miR172 Regulates Soybean Nodulation. Mol. Plant Microbe Interact..

[35] Wang Y (2014). Soybean miR172c targets the repressive AP2 transcription factor NNC1 to activate *ENOD40* expression and regulate nodule initiation. Plant Cell.

[36] Nizampatnam NR, Schreier SJ, Damodaran S, Adhikari S, Subramanian S (2015). microRNA160 dictates stage-specific auxin and cytokinin sensitivities and directs soybean nodule development. Plant J..

[37] Zhu C, Ding Y, Liu H (2011). MiR398 and plant stress responses. Physiol. Plant.

[38] Naya L (2014). Regulation of Copper Homeostasis and Biotic Interactions by MicroRNA 398b in Common Bean. PLoS One.

[39] Kuo HF, Chiou TJ (2011). The role of microRNAs in phosphorus deficiency signaling. Plant Physiol..

[40] Li H, Deng Y, Wu T, Subramanian S, Yu O (2010). Misexpression of miR482, miR1512, and miR1515 increases soybean nodulation. Plant Physiol..

[41] Kim D (2013). TopHat2: accurate alignment of transcriptomes in the presence of insertions, deletions and gene fusions. Genome Biol..

[42] Kim D, Salzberg SL (2011). TopHat-Fusion: an algorithm for discovery of novel fusion transcripts. Genome Biol..

[43] Gao Y, Zhang J, Zhao F (2017). Circular RNA identification based on multiple seed matching. Brief Bioinform bbx014.

[44] Gao Y (2016). Comprehensive identification of internal structure and alternative splicing events in circular RNAs. Nat Commun..

[45] Anders S, Huber W (2010). Differential expression analysis for sequence count data. Genome Biol..

[46] Enright AJ (2003). MicroRNA targets in *Drosophila*. Genome Biol.

[47] Lewis BP, Burge CB, Bartel DP (2005). Conserved seed pairing, often flanked by adenosines, indicates that thousands of human genes are microRNA targets. Cell.

[48] Kozomara A, Griffiths-Jones S (2014). miRBase: annotating high confidence microRNAs using deep sequencing data. Nucleic Acids Res.

[49] Shannon P (2003). Cytoscape: a software environment for integrated models of biomolecular interaction networks. Genome Res..

[50] Young MD, Wakefield MJ, Smyth GK, Oshlack A (2010). Gene ontology analysis for RNA-seq: accounting for selection bias. Genome Biol..

[51] Mao X, Cai T, Olyarchuk JG, Wei L (2005). Automated genome annotation and pathway identification using the KEGG orthology (KO) as a controlled vocabulary. Bioinformatics.

[52] Kanehisa M (2008). KEGG for linking genomes to life and the environment. Nucleic Acids Res.

