# Supplementary material for: Genome-wide identification and characterization of circular RNAs by high throughput sequencing in soybean
Source: Sci Rep. 2017 Jul 17;7:5636. doi: 10.1038/s41598-017-05922-9 (PMC5514102; doi:10.1038/s41598-017-05922-9)
Supplement: Supplementary file 1 — Supplementary Figures and Tables [file 41598_2017_5922_MOESM1_ESM.doc]

**Genome-wide identification and characterization of circular RNAs by high throughput sequencing in soybean**

Wei Zhao^1^

Email: hustzwqq@163.com

Yihui Cheng^1^

Email: chengyihui711@foxmail.com

Qingbo You^1^

Email: youqb2006@163.com

Chi Zhang^2^

Email: zhangchi@genomics.cn

Xinjie Shen^1^

Email: ylssxj@163.com

Wei Guo^1^,

Email: vivi1998@126.com

Yongqing Jiao^*, 1^

Email: jiaoyongqing@caas.cn

* **Correspondence:** Yongqing Jiao, E-mail: jiaoyongqing@caas.cn

*1. Key laboratory of Biology and Genetic Improvement of Oil Crops, Ministry of Agriculture, Oil Crops Research Institute, Chinese Academy of Agricultural Science, Wuhan, China.*

*2.* *BGI-Wuhan, Wuhan, 430075, Hubei, China.*

**Supplementary Information**

Additional supporting information may be found in the online version of this article.

**
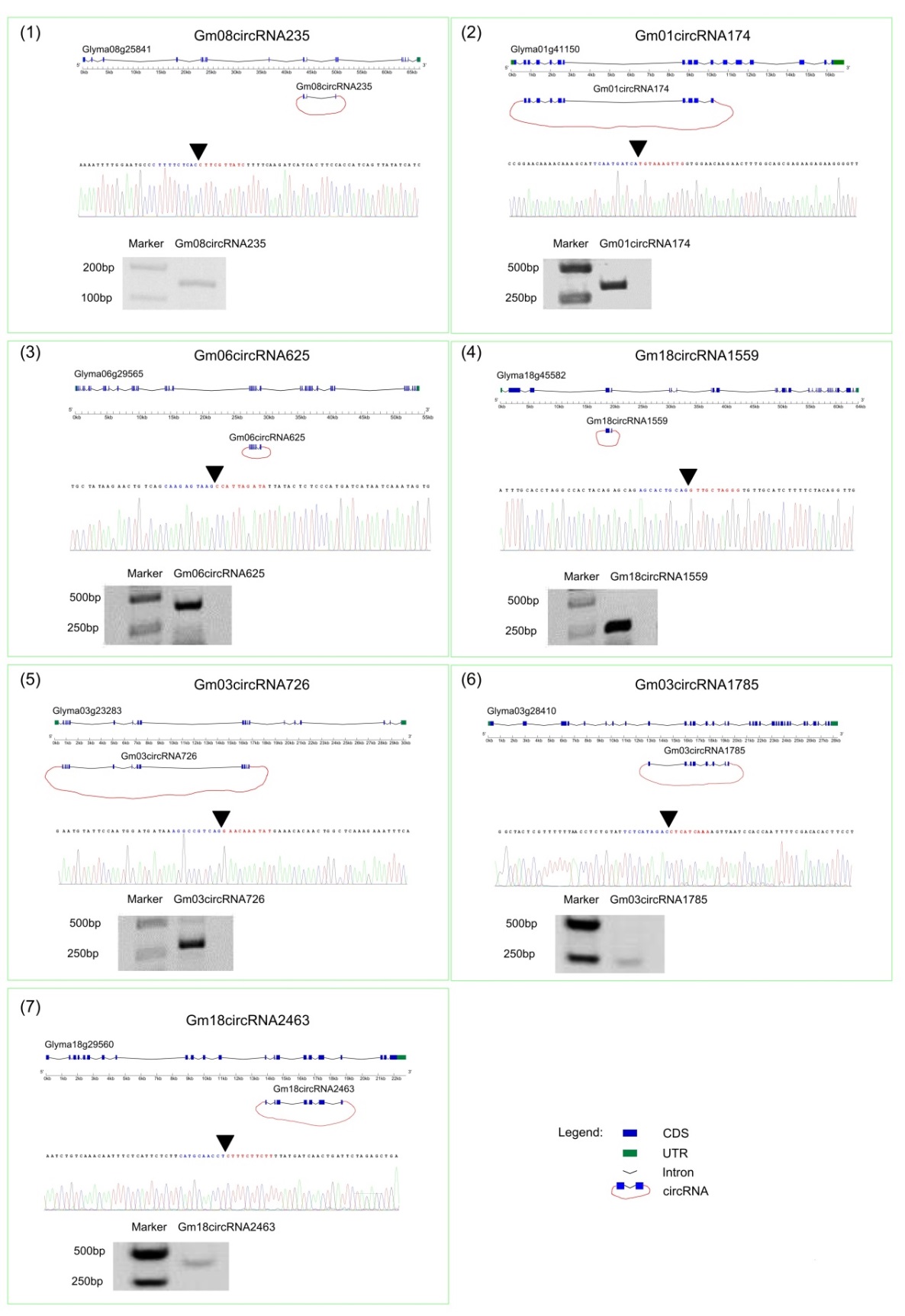
**

**Supplementary Figure S1.** Experimental analysis of circRNAs in soybean. The upper part represented the parental gene structure and length scale. In the middle, the circRNA with back-spliced junction displayed by red trace line were showed. The lower parts were the results of Sanger sequencing and agarose gel electrophoresis. The agarose gel electrophoresis image showed the expected size of PCR product, and Sanger sequencing were performed to confirm head-to-tail back-spliced site (black arrow). The flanking sequences of back-spliced site were marked by blue and red.

**
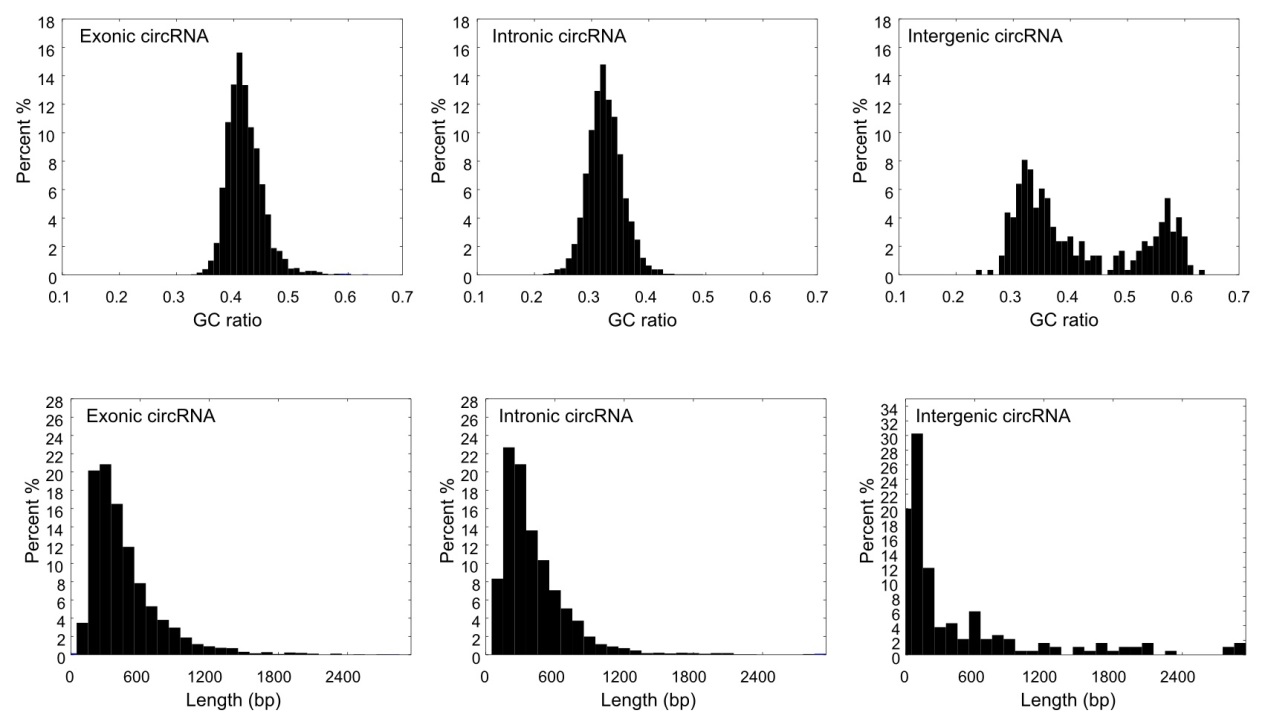
**

**Supplementary Figure S2.** Distribution of length and GC ratio of exonic circRNAs, intronic circRNAs and intergenic circRNAs in soybean.

**
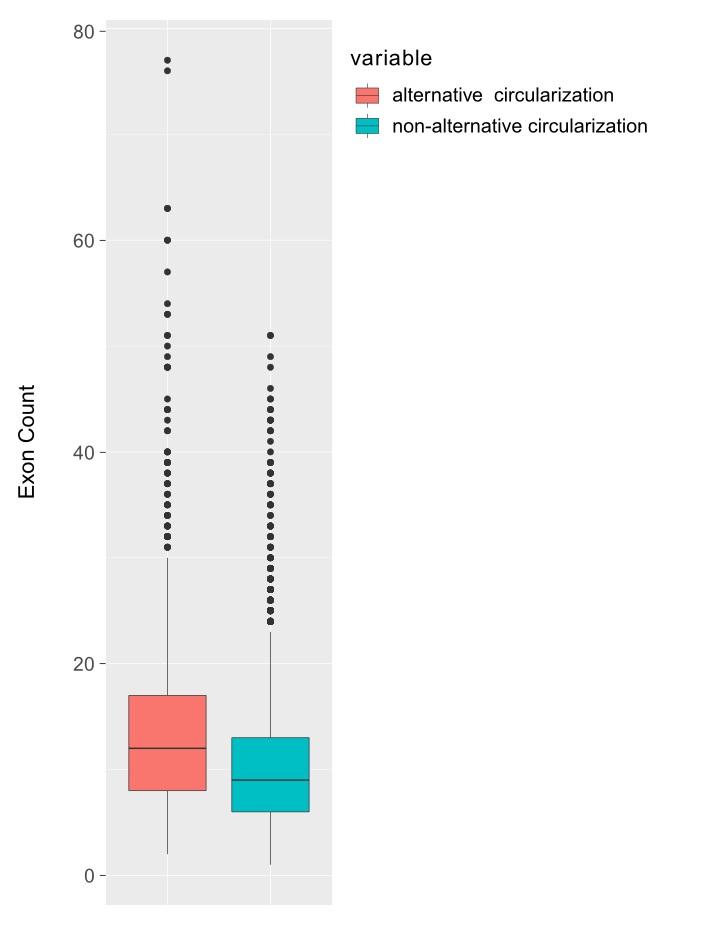
**

**Supplementary Figure S3.** Distribution of number of exons of circRNA-host genes with alternative circularization or non-alternative circularization. Red box represented the circRNA-host genes with alternative circularization. Cyan box represented the circRNA-host genes with non-alternative circularization.

**
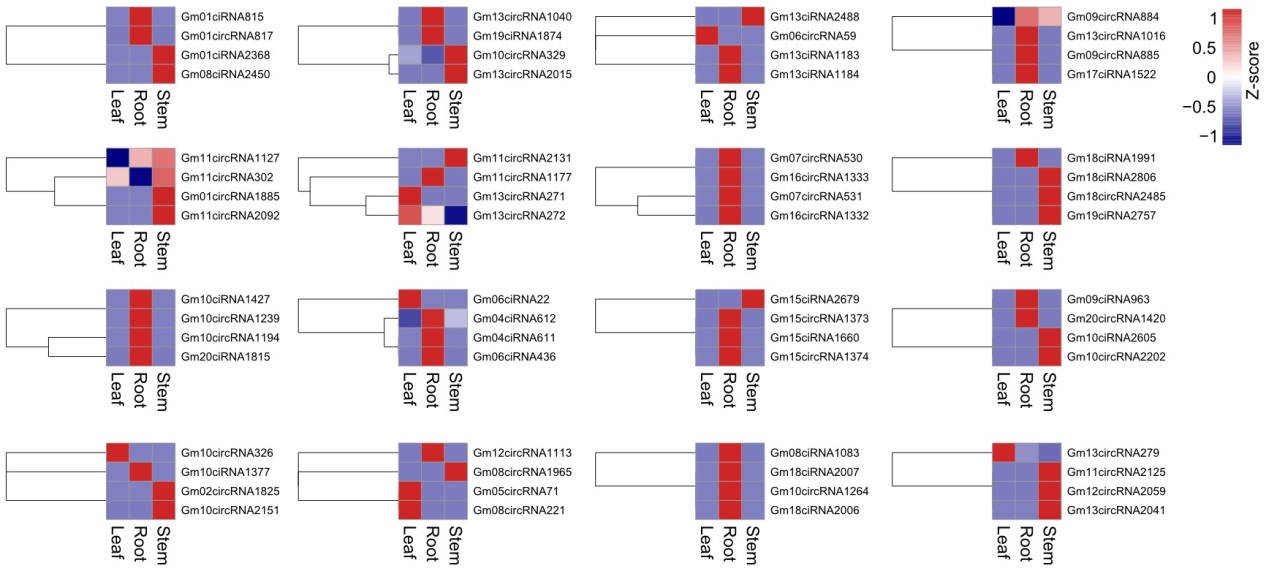
**

**Supplementary Figure S4.** Expression patterns of paralogous circRNAs in leaf, root and stem tissues of soybean. Sixteen groups of paralogous circRNAs were showed. Color scale representing Z-score was showed at the left.

**
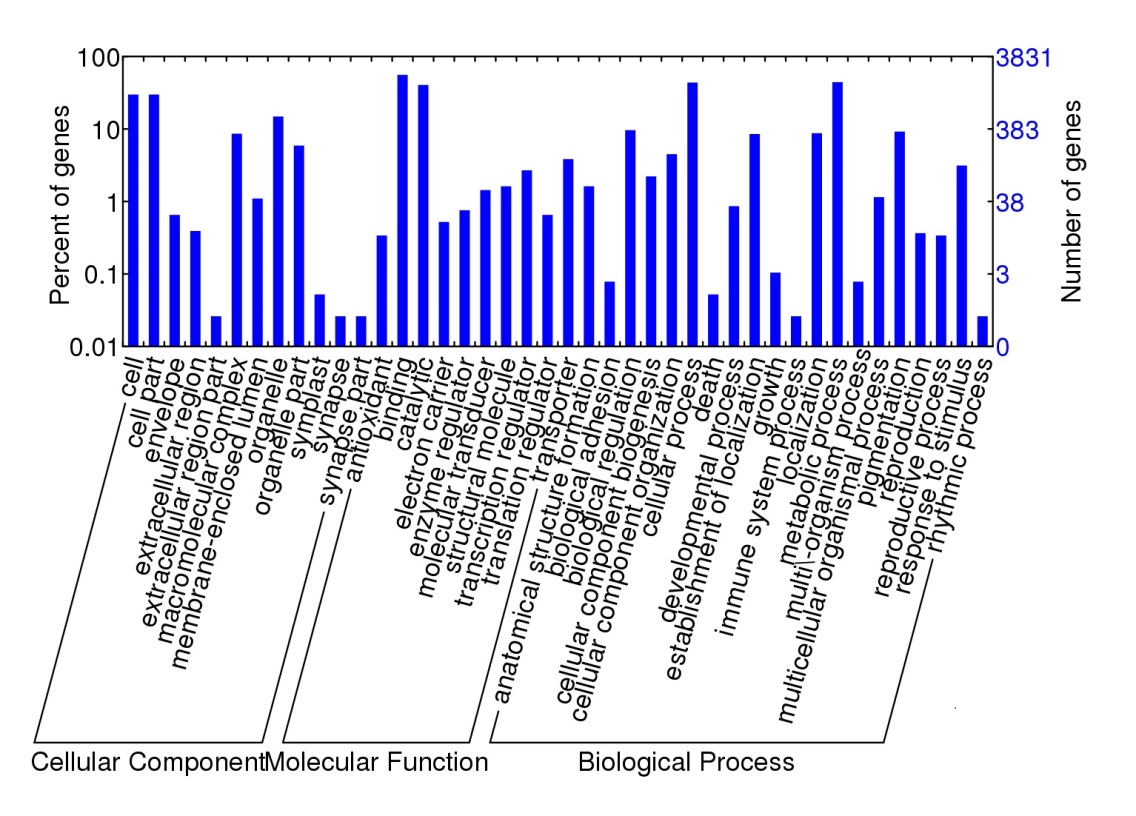
**

**Supplementary Figure S5.** Enrichment analysis of the Gene Ontology function of circRNA-host genes in soybean.

**
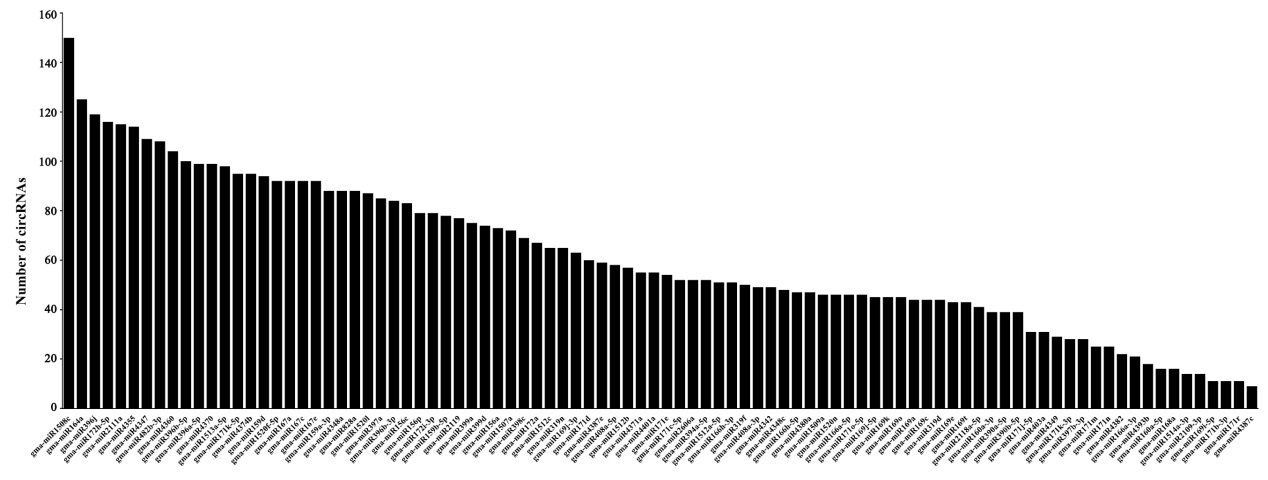
**

**Supplementary Figure S6.** Histogram showing the number of circRNAs that targeted each common miRNA in soybean. The horizontal axis represented miRNAs. The vertical axis represented the number of circRNAs that could target a common miRNA.

**Supplementary Dataset S1.** Sequences of predicted circRNAs in soybean.

*(Provided as separate MS word file)*

**Supplementary Table S1. Summary of RNA sequencing data analysis.**

| Sample | Gma_Leaf | Gma_Root | Gma_Stem |
| --- | --- | --- | --- |
| Raw reads | 61,764,152 | 103,468,250 | 81,809,128 |
| Clean reads | 61,610,358 | 103,305,514 | 81,678,762 |
| Q20 (%) | 99.36 | 99.49 | 99.52 |
| Q30 (%) | 93.33 | 94.38 | 94.42 |
| GC content (%) | 46 | 54 | 51 |

**Supplementary Table S2. Reads mapping statistic of RNA sequencing data.**

| Reads | Gma_Leaf | | Gma_Root | | Gma_Stem | |
| --- | --- | --- | --- | --- | --- | --- |
|  | Number of reads | % of total reads | Number of reads | % of total reads | Number of reads | % of total reads |
| Clean reads | 61,610,358 | 100 | 103,305,514 | 100 | 81,678,762 | 100 |
| Mapped reads | 24,776,642 | 40.22 | 65,432,342 | 63.34 | 47,157,415 | 57.74 |
| Unique Mapped reads | 24,007,001 | 38.97 | 58,113,516 | 56.25 | 45,811,727 | 56.09 |
| Non-splice reads | 23,820,438 | 38.66 | 57,156,492 | 55.33 | 45,058,263 | 55.17 |
| Splice reads | 186,563 | 0.3 | 957,024 | 0.93 | 753,464 | 0.92 |
| Unmapped reads | 36,833,716 | 59.78 | 37,873,172 | 36.66 | 34,521,347 | 42.26 |
| Candidate back-spliced junction reads | 5,961,769 | 9.68 | 788,538 | 0.76 | 4,656,853 | 5.7 |
| Confident back-spliced junction reads | 2,911 | 0 | 6,899 | 0.01 | 7,416 | 0.01 |

**Supplementary Table S3.** CircRNAs identified in soybean.

*(Provided as separate MS Excel file)*

**Supplementary Table S4.** Divergent primers for validation of candidate circRNAs and qRT-PCR.

| CircRNA ID | Forward Primer (5`>3`) | Reverse Primer (5`>3`) | PCR Product Size (bp) |
| --- | --- | --- | --- |
| Gm08circRNA235 | GGTAGTAGCTCATGGTGGTC | ACCCATACCCTCAAGCAGTT | 172 |
| Gm01circRNA174 | TGGACTGAAGAGAGTAGCTGTTTGC | TTTGAGAATCTGATGAACCACCTTG | 320 |
| Gm03circRNA726 | TGTTTCAGTGGGTTCGGCAT | TGTTCACGGAGGGAGAGGAT | 281 |
| Gm06circRNA625 | CAGGCCATGCCTCCATTTTTG | CTGTCCGCTCAATAGGTGTGT | 430 |
| Gm18circRNA1559 | GCAGAGGATGGGCTAAACTT | GTTTCTATGCCACCACATGC | 224 |
| Gm03circRNA1785 | CCAGGCTTCAAAGACCGCA | ATCCAGAATGGCCTGCGAAT | 485 |
| Gm18circRNA2463 | GCACTCAACTGGCAAAACCT | GCTGAAGATGCAGCACAAGT | 633 |
| Gm09circRNA1919 | AGAACTTGCTGGAGCTGTACCCTTG | AACATTGATAACAGCAGCACCATTT | 349 |
| Gm08circRNA1976 | GGTAGTAGCTCATGGTGGTC | ACCCATACCCTCAAGCAGTT | 387 |
| Gm19circRNA2436 | AGGGTTGTGCTGTTGAGG | GGAGGTAGTGACTTTCGG | 352 |
| For qRT-PCR |  |  |  |
| Gm01circRNA174 | GACAGGGTCCGATGTTCGTG | AACCCCTTCTCTTCTCGCTG |  |
| Gm03circRNA1785 | CCTTCACACAGCTTTTAACCACC | GGTTGTGTCTCCGACTTTCCT |  |
| *Glyma10g26670* | ATTGGTTGGTTGGTGGTGGT | TGGCAACCGATGTGTGGAAA |  |
| *Glyma08g27850 (GmARF6)* | ACTTCTACCCGATATGCGCC | CACAGGGGAGGGACAGTAGA |  |
| *Glyma02g40650 (GmARF8)* | CAGGGACATGAAGGAGGGGA | ATCAACTGGGGTGGCAAACT |  |
| gma-miR1513a | TGAGAGAAAGCCATGACTTAC |  |  |
| gma-miR167c | TGAAGCTGCCAGCATGATCTG |  |  |
| U6 | GGGGACATCCGATAAAATT | TGTGCGTGTCATCCTTGC |  |
| *SKIP* | GAGCCCAAGACATTGCGAGAG | CGGAAGCGAAGAACTGAACC |  |

**Supplementary Table S5.** Alternative splicing events of circRNAs in soybean.

*(Provided as separate MS Excel file)*

**Supplementary Table S6.** Paralogous genes and paralogous circRNAs in soybean.

*(Provided as separate MS Excel file)*

**Supplementary Table S7.** CircRNAs derived from orhtologous genes of soybean, *Arabidopsis thaliana* and *Oryza sativa*.

*(Provided as separate MS Excel file)*

**Supplementary Table S8.** The putative target miRNAs of circRNAs in soybean.

*(Provided as separate MS Excel file)*
