# Supplementary material for: Genome-wide identification and characterization of circular RNAs by high throughput sequencing in soybean
Source: Sci Rep. 2017 Jul 17;7:5636. doi: 10.1038/s41598-017-05922-9 (PMC5514102; doi:10.1038/s41598-017-05922-9)
Supplement: Supplementary file 2 — Supplementary Dataset S1 [file 41598_2017_5922_MOESM2_ESM.doc]

Supplementary Dataset S1 : Sequences of predicted circRNAs in soybean

>Gm07ciRNA1

GTGAATGAGCTAGCTTTGCAGTTGGAAAATATGTTAATTTAAAGAGGAGTGCTGGCAACACACTCTCTAGCTTACTCTTTCTTACACACATTTCTTATAGGTTGAAATATATTGAAAACTACAAAATCATGAGAGACTCACTGAAATCTATGATTTCTAACAAATTTCAGTTCAAAAATGTGTGTTTACTAGCATTTTTCTAATTTAAATACCTTTATCTTCTACATGAGCTTTTCAATAACCACGGGTTT

>Gm06ciRNA10

CATGCCTTTCACTTAAAGTTTAAACAATGCTTGATTTCCGTAACTTTTGACTTTAGATTATCACTAGCTTAATCATTAAGTGCAAAAAAATTACAATAAGTTACTTTTATACGCAAAGCAGCACACAATAATCCTACTAAACGCACACACTCTAGACTTCTCATATACAAAAAGAGAAGAGCCAGAAGAACATTGTCTCTCTAAGTTAAGTACCACTTTGA

>Gm13ciRNA100

GTGACTCTGAATCTCTTATTAACGAAATATTACTATTTACAAAGCTGTTTTGTTATATCTTCAAGTTCTCGTTCGTTTTGGCCATCTCTGTTTAGGATTAGGATTTCGTATTTTCATAATTCATCCTATAAAGTTCCTGTGTTGATAGATCTTTAGGCGATCTGCTTAATTTAGTTAATGAATTTATATATGATTCTTAGGCTGCTTCTTATATCCGTTAAAGTCAACTCAGCCTGTGTGAGTTTTGGTCTTAGCACTTGCAATCATGAAATTAATTATTTCATCTTACTAGGGTCTTGGGGCAACATTTTTCGTATAATTACTAGCATCAAACCAATTTTTCTAACTTTTTTCAATCTCTATCCCTGACTTCTTTGATTTATTAGAAATTAAATGAGGATAATAAGCTTTAAATTCTTGCTAAACTCCCTTTGTGTTAGTTTCATGAAAAGTAAGCTA

>Gm08ciRNA1000

CATAGGATCCTTCGTAAGAAGAACTTAACTAAAGTTATACAAATCGTATAAATTTCTCCTCCTTACGTCCCATTATAAGATAACAAGTGGCCCTATGACGGACTACGACAACTACGAAATCAATGGTTAAAAGATTTTTAAATCTAAGTTCTAATATATTGAAAGTTTTGTCAGTTCAACTCCGTTACTTAAAACCACCGTAAATTCGAGTTACAAAGAAATGTTGTATAACGGTGAACATCGTTCGTGGAAAAGAAGATAAGGAGGAATCTCATTGCAAAGGAAT

>Gm08ciRNA1001

GTATTTGTGCGTGGATGGCGGCTCTTATCTCTTATTTTTCGACTTTTCGGAAATATATTAGCTGATGAATTAGTAGTTGTTGTTTCTTTAGTACCTTTAGCAGTTCTTATACCTCTCATATTCCTTGGATTATTCTAAGTATGAAAATATCGAAATAAAATGACTCACGACGGGCTGTTAGCTCAGCGGTAGAGAATGCTCCTGATAATTGGTCGTCATGCTTGATACTAATTTTTTGAAAATTTACTGATTAGATTAAAAAAATTTAGTTAAAAATATCAAACAGGTAGTTAGGTTTGCAGTCTTTTATGATTCTTTTTTTTTTTGTTTTTCTTTTGATTCGTAGTTTGGTTTGCAGTCTGCTAACTAATAACT

>Gm08ciRNA1002

GTATGGAATATGGTGCAGTCTATTTAATTAGGATATGCTTTAAATTGGAAAAGGCAAATTAAGACAATATTTTTTAATTATCATGTACCAACCGATGCCATCGTACGTGGAGGGAAACATTAAGTTGGCTCAACGAAGTTTTCAGTAATTGTTATAGATGTATATATGATTCATTGCCTTATGCTTTCCTATCTGAACATAACTACATAAATGTCACACACACACACT

>Gm08ciRNA1003

CGTTCATTGGTAAAAGTTCCCTGATACGACATGCTTTAGAGACAGTTGTGTGGAAACTTTTTGAAAGTACAGAAAAAGAATGAAGATAATATAGAAAGAATATACCAGTAACTAAAGACTTTCAGCGGAACCAGTAAAAGAACCGAACGATTCGAATGTTACATGGTTTACTAATATTTAAATAACCTCTAAGATGGTCTAACAAATAT

>Gm08ciRNA1004

CATATATGAGATAATAATATGTATGTATGTGTGTAGAGAGACAGATATAGAGAGTTAACTGTGATCACATTCTGAACCGTGAGAACGTGTTAATGTTGAAATTATCCAAAAAAAAATAATTAGGTCAGAAGTCTGATAGTAGTAATAAATCTAGAACTTACGGTATAAAAACTTTTTAAACTTCTTTTAGTACAACAGTTTAACAGTAGTTCATCAACTACAAAATCAAAGAAACTTTTTATATAATTTACGATCAAACTTCTGTGAAAATAACTCACCTTTTGTTATAAACTAATACACTTTTAATACTCACGATTGAAACTTTTTAAATAAGTAACCACATACTAACTAATACTTAAACTATATGTGGTGACAGTCATCTAGGATTGGGTCATATATATCGGAGAATTTAATAACTTGAACACGAATAAAGGTAGGGATAAAAACATGATATTAACGATACGCTAAAACTAAACAAAACTTCAAATTTGTCGTACCACTATATGTAAGAAT

>Gm08ciRNA1005

GTAATTTCTTGGTAACATGTTGATGCCACATTAAATTACTTTCATGATCAACTTTTATATGAATATTAAGATTTATCGTCACTGCGGTCATCTGCTTACAAGTTCTTTTGTTGTGTTAATGCAATTCACTGTGTTATTTCTTTTTTTTTCTTTTGGTTCAGGAGCCAGGACGAAGCCTTTACTTCATTTCTGCAACTTGTAGATGCTAGGAATGAATATTTCAGTTATATGCATGCATGATGCATCTTTCATTTCTCTAATAATAAGCAGAAAAGAAACTTCTTGTTGGAAAGCTGCTTTAATTGTGGTCACTCTAAGTCTGCCTTCATTGAAGCCTCCAACACCTTCTTTGTGCTGGGTGTGTGTGGCTTTGTTTATTCCTGCATTGCTTAGTGATATGGCATAACTGCCTAAGTAGTGCTTATAAGGCATGTTTGGTTGCCATGATGCTATTTATAGGAAAATTTATATCTTTGCATCTTGATT

>Gm08ciRNA1006

GTACTACATGTTAGTATGTTGCCTGACATGTTAATTTGCTTGTTAATATAAATCTTCTATTTTATAATTGTGGTGATGTTTTGTTGGATTACTTGGTGGTTGAATATGGTTAAAAATGAATGGTGTTGCCAAATTTTTGTTTTTTGTTTTATTTCATGTGTATCCTTTGGGATGCTTCTGGCCTGCTACTTAAGAANNNNNNNNNNNNNNNNNNNNNNNNNNNNNNNNNNNNNNNNNNNNNNNNNNNNNNNNNNNNNNNNNNNNNNNNNNNNNNNNNNNNNNNNNNNNNNNNNNNNNNNNNNNNNNNNNNNNNNNNNNNNNNNNNNNNNNNNNNNNNNNNNNNNNNNNNNNNNNNNNNNNNNNNNNNNNNNNNNNNNNNNNNNNNNNNNNNNNNNNNNNNNNNNNNNNNNNNNNNNNNNNNNNNNNNNNNNNNNNNNNNNNNNNNNNNNNNNNNNNNNNNNNNNNNNNNNNNNNNNNNNNNNNNNNNNNNNNNNNNNNNNNNNNNNNNNNNNNNNNNNNNNNNNNNNNNNNNNNNNNNNNNNNNNNNNNNNNNNNNNNNNNNNNNNNNNNNNNNNNNNNNNNNNNNNNNNNNNNNNNNNNNNNNNNNNNNNNNNNNNNNNNNNNNNNGTCTTCCTCATGTATTATCCCGATTTACTATCTCTTTGTGCAAATTCCTATAGAGTGGAAGATAAGATTGAAAAATTATGTGAAATTCCTTTCACGTGGGATTGGAGATTGTAATAGGGTCATCTGGCTATTTGTGAGTTTGTTGTAAGGCTAGAAAAGAATGAGATCTTAGTTTTGGTGGTTTAGTTTCTAAGAACATTACTCTAGTGGCAAAATGGTTATGGTGGTTTCCCTTGGAACTTATCTATCTGTGGTACTGGATGATTTGTAGCATCTACTGTATTCACAGCAATGGCCGGGGTTTGGGCAAACATTATGGTGGCTTCCCTTTGAACTCATTCATGAGTTTTCAAGTCATAGTCTTACACCAAAGACATGCTGATGGTTTGGGCAAAAAGAAAATAAAGTCCAGCCCAAAAGTTTTTCATATACTCATACTAACATGACAACTTCTTTACTCTGATCTGAGACCTATTTTCACCCAAACTCAAACCTAATTTCACCTAACACGGGTCCTTTGAACCATCTCCCTTCCTTCCAGAGACTTTGGCTTTGAGATGGAGGGTGACTGAGAGTTCTTTCGAGTATATCATTTAACTATGTTGGCTTTCCCACAATATTTGATCAAGATCCACCATTGTTTGTTGACTTGGGTTCCTGTTATTTCATCTATTCAGAATTCCTTTGTTTTTATCCTGTTTTTTACTGCTGGTGTTGGGTTTTATATTTGCCATCATCTACTTGTTTCTGGGACATTTTTAGATTAAAAATGGCATCTAATGTTGGGTATATACTATATGTAATAGCTGTGATCACAACTCTATTGCTATTTGGAACCTTTTCTTTTTGTGAATTCGTTTTCCAGCAAATCAAATAGAATACAGGTTTGTGTCAGACCCCCCCCCCCCCCCCTCTCCCAACACAAAAAAACAGCAGCAGCCACATTAATCAATTCTTCCTTAATGATCAATCAATGAAAATGTTGATTCTTAACATTTGTCCTTGCTGCATTTCTTTTTGCTTACAGCCTGGAGAAGAACAATCTTAATCACAGAGATTAAAAAATGACTGTTAGTATAAATGGGAAACAGTTGTTATAATTTATAGCCAGCTACTTGTTAATCTGTCATTGAGTTTTATTTCTTTGATTAAAGGATATACTTTCATAATCATTTTTTCATTGTCATTCTGGAATTCCAATTCAAATGTTAATAATTTTGAATTGCATATTAGTTACTTGCATTTAGTCCAAATTTAGTGTTAATTCATTCTGTCCATTTTATTGTTGGCGACTATTTGTATTGATAAATGCAGTTCTTTTTACTCTTTATATGCATAAAATCGTACTTGCTTATGCTACAAAATGTTGCGGTCAATGTTGCTCTAATTGGTTCTGTTTTCTTATACTCATGTCAGTCCAATGATGAATTCCTTGTTATATTTTAATAATCTATGCTTATTATTCAATTAATTGTTTACT

>Gm08ciRNA1007

CACTTCATGAAATGAATGTGCACATAATTATTAGAGATTCTCACGAAAATTTAAAATCACTTGGCAATGAAGATTAGTATTTTAATCCCTAGAAGGTTTGGAGTTACAATTCGAACTCCATAAAAAACAACAACCGGGTCCACCTCATGTGATCACAGTTCACAATATAGTCAGGTAGTCAGGTCAACACTATAAATTAAAAACTAGTATGCTTAAAGTTCGGGAAATCAATTGAACAAGAATTACTAACTAGAGAAATAGATAA

>Gm08ciRNA1008

GTATTTCACATCCCTAACGATTTCGGTTTCTTGATTCGGAAATAAAACTTCACGGTTTATATTTACCCTGCGTTTATCCCTGATCCTTTTTATTTGTATTATTTGGATTCTATAGAATGTTACATTTTCTATGCGTCATTGCACTGTGCCTTTTCGAGTGTTGCTTAGGCTTTAGTTATGCTTATTTTTTTTGGTTTTTCATTCTGATTATTCTGAGAATGTGCGCTGACTTGAGGAGAATTAATGGTTTAAGTGCTATGAGCTGTAAAGCTTAATCATTGTTCATTACCTCAAAGTATATAATTTGTGCACACAAAAAAGGATTACAAATTTAGATCTAGTTACTCAAGAGCTTTTAGTGCAATTCTGAAGAATTGAAGTTTTACTCTGGTAACATTTGCTGATAGACGATTTAATGGGTATTAATTTTAAGTATGTATTTTTATTTAGACTTGGGACGCGGCTACTTATAAAGTTCAGCTTCTGTCCATCATAGAAAACTGACTCCTATTTTCCGTAGTTTAAGTTTTAACATTGCTTAGACAGACGATTATCGAAATGAACCTAATTTTAATTGGAGAAAAATACTAAATACACATGTCCGACTCAATGAATTACTGTGTTATTGATGATTGATGGTGGCATTTCTGCCTGGCTGGTTTAATTGTTGGATTCAATCATAAAGTGGGTCACACT

>Gm08ciRNA1009

GTATTTCACATCCCTAACGATTTCGGTTTCTTGATTCGGAAATAAAACTTCACGGTTTATATTTACCCTGCGTTTATCCCTGATCCTTTTTATTTGTATTATTTGGATTCTATAGAATGTTACATTTTCTATGCGTCATTGCACTGTGCCTTTTCGAGTGTTGCTTAGGCTTTAGTTATGCTTATTTTTTTTGGTTTTTCATTCTGATTATTCTGAGAATGTGCGCTGACTTGAGGAGAATTAATGGTTTAAGTGCTATGAGCTGTAAAGCTTAATCATTGTTCATTACCTCAAAGTATATAATTTGTGCACACAAAAAAGGATTACAAATTTAGATCTAGTTACTCAAGAGCTTTTAGTGCAATTCTGAAGAATTGAAGTTTTACTCTGGTAACATTTGCTGATAGACGATTTAATGGGTATTAATTTTAAGTATGTATTTTTATTTAGACTTGGGACGCGGCTACTTATAAAGTTCAGCTTCTGTCCATCATAGAAAACTGACTCCTATTTTCCGTAGTTTAAGTTTTAACATTGCTTAGACAGACGATTATCGAAATGAACCTAATTTTAATTGGAGAAAAATACTAAATACACATGTCCGACTCAATGAATTACTGTGTTATTGATGATTGATGGTGGCATTTCTGCCTGGCTGGTTTAATTGTTGGATTCAATCATAAAGTGGGTCACACTGA

>Gm13ciRNA101

GTATGAATTACTAATAACCTTTCACTTAGCTAGCCCTTTTGATTGTTCATCTTGATTGGAAAAACAAGCCAACACAATAACAAACTCAATAATAGAGTAATAGACAGTGGAAATAAATTCTCCCAAATTTGCAAAAACCTAGGAGCACCAATAAATTATAGAGCGCAAAATAGAAATTAAGAAAAATCAGAGACACGATTTGTTTAAAGTGAAAAACACCTCAATACAAGGGTAAAAACCATGGTTCGTCCAGATTCATATATCATCTT

>Gm08ciRNA1010

GTGAATATTGAATATAATTTCCGACGATCTATATGTTATATATTGGTGGCGTGGGGTTGATGGAGCCACCTCGACTGCGATTTGGGCTCTCTTGTTCTTAAATTTATTGAAATGCATGAACATGCTACTGCCAAATTAAGAATTGAAATGTTATGCCAATTGCTGCATCTGCT

>Gm08ciRNA1011

GTAAGATCTCTTTCTAAGATCATAGCCTCCTTGTTTGATTCTGTGTTTTTTGGTCTGTTTCTTGTTTGTGTAGCATGCTTAGAACTTGTGTGTCCTTCTTGGGTGGTTTGGTGAATGGTGATGTCACTTTTCAGTTTTGGTATTTCATCACATAAGATAAGGATCAGCCCGTCATAGTTGCTCCACCTAAAGAGTATCCTATCCCCCTCTTGTCAGGAGTAATGGTTGCAGTTAGCCAAGGAAGTTCATGTAGTTATAACTGTACCTCTGCATTTTTATTAAGCTGGAAATAAATTGAAGTGTGTTTTTTATATAAAGAAAAAGGTGAACTGCAGTGTTTATACTTGTTGGTT

>Gm08ciRNA1012

CATAACACATGATCATTTTAGTCATGAAGTAGACAATCACCATGAATACAATATATTCTTATAAATCAAGATTTGTTTATAGGTCCGAGACGTCATACAAGACATCCTCGAAACTGTTCCATATTATAGAAATACAGTTTTCAGTTTTTGAACTTTTATTACAGTGATTTTTCACAACAACCATACTACGACGTTTATAAAAACTGTAGAATTACTGAACAAAGCACAAAGACGTTTGTATATTAGTTCGTTACCTAAAACGAAATACCAACTGACAAAACATTTGTACAATGTACAAAGAAAGGAAGTTTGAGTAAATTAAC

>Gm08ciRNA1013

CAATCAAATTAATTGTGAAGTGGTAGCGGCAAGTTAAAAAATTAAAGCTAAGCTTGGCAAAAGAAATTGACATACGCGGCTTGTTCATTGCAACACTTCTTGTCTTAACTCGCAGTGGCAAAACAAATTCCTCTTTTATAACTAAGCCACAAAGTGAAACGCAAAACAAAAGCAAAGATCGATATGAAGCTTCACCGATATAATTGACACAAATCAAAAAAAAGAAGAAAAAGAATAAACGTAACACAAAATGACT

>Gm08ciRNA1014

CATTCACAGAGTTGATTTTTTAACAGGCAGAACGTTTATACAAAACATGGTGAAAGTTACGAACTCTATTATATGATGGGAGCACAATTAATCATACAACATAAGTCCAGTCAAACTTATTTAGGTTACAATTACGATTAGTGTTCCCTCAAACTTGATGATCGCTCATCGTGAAAAACCAAAACGAACAAAACATGAAATTTCGATCGACAACGAAAAGTTTGTGTAATTTACGCGAAATGTGAACTTATAAAAGGGGAATAAAACAAAAACACACAGACACTGGTATAGTGACATATACTTTCTCATAAAATCGACTACCTTACGCTACCCTAATTTACTCTTGGTGTTTTTTCGAAGAGGAGTGACGGGGAAAAGTTTTAAGTCTTGTTCAATTATAAAGTAGACATAGGAAGAAAAAGGGACAGTCTGCGTGAATTATAATAACTACTGGACGTAACTTTGAATTGTAAAGTGAAACCACGGTAGCTAGAACTAATTTTCGTCTAAAAGTCAAAAGTAAGTAAAAGTTTTAACTCTCCGATTGGTATTTTGTAAAATAGAATTGACTAGAACTTCGAATAATTACATATAAAGAAACTCGGTTAGTGATACTTATTACTTAAATCGAATCGGCAACGGGTGACACGACACGAAATCTTTTACAATCTGACCTCTACGATATGACAGACTCTTAGAAATAAAGAGTACAACAGTAAGATTTGGCTTTATGGCCAACACGGTTACTTCGTTGTTTAGTACAGACTATTTTAACATACGTCCCTAGACGGAACAAAAACGAAAGACAACTTATCAACGGTAGGTTAAGTAAGAAGAAT

>Gm08ciRNA1015

GTGATTCCCTTAATTTCTCTTAGTCTTAGGCGCCTACCTTAGATCTTCTCTCAGAACTCTTTAGCATGTTTACAAAAGTCTTAATTCTATCTTGTAGATTAAGTCACAGCACTGATGACTAATGTGTTGACGCGCTGGTTGTTCACTTACATTTCCAATTTATTATGATCCACTATGCTTTGAGTTGCTCAAAATGCATGAGAAGTAGTATCCAGGCTTATTACT

>Gm08ciRNA1016

CATTTGAATTTATGAATTAAGTAAATGTCAAGAAGGTACGAAATGACGCCATATCTGTTATAGATGTATCAAGAAAAGAAATGAATTGTTCAACAAATATGGTGAACGTAATAGGTTGAGATTACATCCGAAACAACAAACGTAATAAGTTGTTTTTTTTTGTACGAACGACAGTGAACGAATGTATATAATACACCTCCACGAAAGATTACTACCATGGTTGATTGATTTGGTAAAGAGTTGAAGATAACAACTATGTAACCTGAACTTTTAACCTGCTAAGAACAGGATTGCATCGAACGGATATAAATCGTACCTGACCAATCCCATTTAAGTCGTACTGTTCCGCGACTCAGTTTACCATAATTTACCGAAGGTATGTAAGTTTATATTCAAGTAAAACACCAACTGATACAACTCTTTCCAGGTGTTTTACTGATGACTTGCCAGTATAGATTATTTTAGGTCAGTCAAGAGAAACCTTGTAATTCCCCGTAAACCAACCCCTCTTCAAAAAGTCCGGGCCCTTAGTTATTAAGAACCCATACTTATTTTAGACAAACCAACCAGTATTTATAAGAACACTTATAAATTTTTCAAAAGGTCCTTAGTTTCACAATACACTACAAAATATCCATGAAATGTAATTTATTAAATTATTCTCATCGACACTCAGACACTATAAAAGTGATCTCATCATTTTTTTTAATTGAGCCTTTTCAATCTAAAGATGGAGGAGGTACTCTTTCAAAAGTACCCTTTGGATCGATTTTTGTAAGGAACCTTTGTGAGAAAGACTCCTTACAATACAAAAAAATCTTTTTCTATATGGTTTGTACCCTTTGATTCTAAGGGTCCTTTGATTTTTAAAGAGGGGATGGTTTGTGGAATTATTTTGGACACACCAGAGAGTTTTATAATGAGTCTAACAGACGGACGTTTATGTAAATAAAACTTTGACCTTGTTTATCAAGATTAAGAACATATCTACTAATTTATAAAGAACCACACTGTTCTTTATTTCTCTTTAAAACATCCCACAAGGTTCATTATCAGACATTGGCGTTATAAAACAATGACAGGCTCATACGTTGTCCATCCTATTTAATAAAACACAATAGATGTTATAACTATAGACAACTCTGAACTCTTCATCAAACTTACTAAATATAAACAGTGTTTTCATCACCTATAAACGGAAGTGTTCTGTTCATCACCTATAACGGAAAT

>Gm08ciRNA1017

CATTCGTACTAGAATAGAAGGAAAGTTATATCAACAAGAGAAGGGAAGTGTAACTAACTCAACGATTAATAATCAGAGAAAGGACACTAACGTAAGTAAAGGTCAGTAATAACAACATTGTTTAATTGTCCCGGAACCAAATAATACTTTACTAGTAAAAACAAAATCGTATGAACATTTCCTTGAGAAAAACGTATACTTCTTCTTACTTCCCCCCGAACGAGGTCATAGTATATACCTTTGTATTTCTTATTAATTATAAGGTAGGATTTTAAAACGTTGTGGATTTTAGTAGCACTTCCACTCGAGAGGTAATGTTTTTATTTAGATGTGAACCTTTGGACACTGAACTCTATATACTTTTTGTCGACTAACAATTTCAATACACGTCTGTTGTTTTATAAGTAAAAGAGAACTGTCGCCCAGAGATGAGACTTCTATGAGGTCGAAAGGGGGTGAGTAGGGAAGTGAGGTTTTGTTTTGACAATGCTTAAATACTACGAACTGACATATAATTTCACTTTGAAAAAACCCTACTTTGTGACTAAGGTTTCTAAATGCGAAAAGGGTGAATTGAAAGACCTGTGGAGAAAACGTACAAAATAACAGTCCCTAAACTAGGAGGTAACAGAATCCTTGTACTTTATATTAAAAAAGTTCTTTTTTCGTATTTTGTTGTACCCTGGGTGGTATAGGTTACGTCCCTGGTATTCAGGTAGTATCCTCAATGACCAAGGAGAAAATTTTTACCGTAAAACGTACAAGGATTCCTTTTTCTTCTTATAGGTTTAGGAATAACAACCACGGAAACATCAACTAGAACTGTTTAATTGACATCATTTTCTATAAGTTGTTAATTTCTAACTTACACTCCCCACACTTCTTTCACATGGACTGTGATTTCAGTTCCGATTATCCTATATCACTAGAATTATTACAAAACAAAATCCTCCATCAACACTTAAACTCTGTAAAATAAAACATGAAGACGTACCAACTATAATTTTTGACTATTTATTTATGTTAACACACACTTAGATATATATCGACCAAGAATTAAGTTGAAAAGGCCTACGATCCAGGTTATCGA

>Gm08ciRNA1018

CAGTCACTTATAAAAAAAGAGAGAAACGAGTGTAAAAGAAAGTTAAGTTGTATATTAAACTAGGGGCTTTCATTGTTACACAACGAAACTAGGGATTCCGATTTTGTTTAACCAGAGATTTACACAAATGTACGATTGATTAATTAAATGGGATAGTTCAACAGTTCAAGGATTGTTAAAAACCGCATGTCAGAATTTTTTTCATTAAAACTACATTTTATTAGATCATTAATAAAACATCTCTAAAAATTTTTTTCTTTTTCGATAAAATACAAACAAACGTTATTATTTTTAGTGAAGATTCTTTTTGTTAACAAAAATACTTTCTGACAAGTGATATGGAAGACTAAAATTAAGCTAAATTCTATAAAAAAATCGAAGACTAATAAAACTTTTCGTCTTTGTTTCCATACACATAATTTTTTTTCGATATTGTTTACCTGGGAAATAAGAGTGTTACCTGATTAAACATATGTGTTTTCGGAAATCCCTAGTTTCGGAAATACTAAACATATGTGGGAAATAAGAAAAGTACCATAGAGAGAAAGCAGTGTAGGAGATGTTTGAACTCTAAAGTAACAGTTTTATATTGTTTCACTGTGGAGTTATAAAGAAGAGTGCCAGGTCAAACTCGTAAATGAGT

>Gm08ciRNA1019

CACTCTTAAGTGTAATAATATAAAACATAGCAATTCTAAACCACATAAGAAACAAGTCCCTTACGACGACTCACGAGTGACGGAGGTGTACGTTATCGACTTTACTACAAAACGAAACTTGTGGTAATTACTCAAAAGTCGTAATACAACTTTTTAATTGTTTATTTCTGTGTTATTCTATTTTGGTTCTAAGCGGATACATCTTCAAAATTGACGTATTTTTATTTAACTCTTTGAAGATGATAACACTGGAACACAAACCAAATCAATTTATAATAAACGATTTTATCTCATAATTTTCAAGTACAGGTGTATTATACGGTAGAAAAGAAAATTGAAGATTCCGGTGAGAACAAAAATTAGTACTGATCTGTATAAATACACGTAGAAAAAAGCGACGTATTACCGATTGTTATAAAGACTCCATGTCCGTTCTCTACTTGTCAGTAGTAGAGGTATGAGAGGAAAAGAGAATGTGGAAGT

>Gm13ciRNA102

GTATGTCTGGTACCTTCTGATGTTATGTAGCATCTCCTACTGATGCGAGATCATTAGGGGGTTGGGGGAAGGGAGAGAGAAGAAGGGTTGCCTCATAAATTTTGTACTTTGTGATTATTCTGTTGGGTGAATTAGAGGATGTGAAATTAACTAGGTAAGGAAAAGATCTACTTTAATTTTCTGACATTTCTCAACCCCCTTATAGTCGATTAATGGGGTTGGATATCTGGAGGGCAGCTGGGTAGGTCCTATGGACTTGCTAAGTGAGAAGAGAATTAATTTCATACAGAACAGAAAAATCTTACCTTACTAACTAGGTTTATAGCTAGCTTCACTTTATTAGTTGAATTTTTTAGGTGCACAATAATTCTCAGCTTAAAAGAATTCAAGCTATATAGTTCTCTCCCTTAAAGTCGTTTGTGGAGCATGGATTGTTTCTTTGTGGTAGGAATAACTGATACAGAACTGGGTTACGTGGTAATACCGGCTTACTTGTTGGAATTGGGTTAATTGGCAACCAAGATCTTTGGGAAATTTGGAGAAATCACAGTTCACAGGCATTTATGGAATATGCTATAAATTAATAGATAAAACTTGTGATCATACGGAAGAGTTTAGATCTATAGATTTGCAAGTTGGGTAGACTAGATGTATTTTGTATTGTCAACTGCTTGGTACTCCTTGGACTAAATCTGATCCTGTATTGGGTCAATTGAAAAATTCATACTCAGTTTTATCATGAAT

>Gm08ciRNA1020

CAAAGGGGTAAAGGAGTTCTATAAACAGTTTCTTGACAAACTGGATTTTTGCATGCAACAACATAGTTCTATTTCGTGTTTTTACCAACGGTGGTACGTCCTCAGGAACCGCCAACTTTATAACATTTAAAACCACCCCCGTAATGGGGTAATGGTTGACGAACCAGGTAGGAACGAT

>Gm08ciRNA1021

GTATTTAAGCCTTATTTTCCCCCTTCGTTCTTGGGAAATATTTCCCATGCTTCTGTCACTAAACATTACATTTGCTTGGCCTTCCTTTTTTTTTTGTTGATTTTTTCTCCTTGTGTTGTTGTCTCCCTGGGGGTGGGGGAGTGTATAGCTTTAGCCTGTGTAATAACTGAGTAACCAGATCTTCTGTTTTGGAGACTAAGCTGAGTTTACTTGATGGTTTTCTGCATACCTCATATATCTTATAAACCTTCACTGTCACATAAAACCGTTAAGTTCACTTAAATTCTTTAGTTATGTCATCGTAATACCTATTTGCTGATTTTATCTAGAAGTAACATGAAGTAAAGAATCCAAGAAAGTTGAACAAAATCAATTGTGTTTTTCTTTGGTGCTACACTTTTCCCTGATCTTATTCTGTGCTTTCAGACCTTATTTTTGATTGACATGTTAA

>Gm08ciRNA1022

CATACAACTTTTAATAAATAAGTTAAGACCAACGTCAAAGGAAATGTAATTACTATCCCATCTTCGACTTTAATTCATTTGTCGGAAGTGACTTTCAAGGTCCAAATGATTTAATTAATCCAAGTCCTTTTACTTTAAGGAGAACTAGGGAAATTAATGAATAACTTGAGACGATGACAGCACGGTTTGGTATCTAGAGTCAACGCTGTCGATAACAGTTTGATACGTCATACATATACTAAATTGATTTTGAGGGACAAGAAAACTAGTGAATGAAACATTAATTACTAGACCCATGACTACGTTAGTGACATACAGACCAACCTCTTAGTGTAACAAAGTACTCATATAAAAATACTTACTATAGATCATTTTAATCGTTTCTGACATGAATACCGGTGTTGAAGGAAGTTGAACAAGGACTTAGTGAGTTAGTGAATAAATCCACTATTGTTTCGATAGACCAGTTAGACGACATTAACGGTTGTTAATTTTTAAACTTTAATAGAGGTTAAGAAACTTTGAGGAATGATGTATACGTACCAGACTTTTTTTTACGGAAATATATACCATAGGATTAAATGATAACTTAAACTTATAAGTAAATTCATGATTTTGAATAGAAT

>Gm08ciRNA1023

CACCCGTTACGATAGAGAAGACAAAATATAAAAGTAACGTGAACGTGAACGTACGTAACGTGTGACCATTGAACCAAAATTCCATGATTCTCTAATGAATCACCCGAATACCAAGGTTGAAGTATTATTATTATCATCCATAATAATAATAATAACTATAATAATAGCAACTACGATACTACGACAATAAGGAAAAAAGACGAATCCGAAAAAGAACAAAAAAAAGATGGGTGTGGAAAAGAAAAGAGTAGGTCGATCGTCCTTCCTGGGAATAATTCGTATCATTTGACCCGGATCTTAAAACAAGGACATCTGTTTTATAAAAAAGTCGACAATCAT

>Gm08ciRNA1024

CATCCACCGATACGGGTTAACAGTTAAACGGAGGACGGTACTTACTAGTCAGTTGAAAAACTCAAAATACTAAGTATTCTAATCTCTCCTACTTCTAGGAATATAAGTAATTAGAGAAACAAACGGTTAGACAACCCCAACACAGTCACCAATTCTACACCTGAAAGAGATTTGAGATATACGAATTATTAAGTTAGAACACGTTGTTTTGGAATAAAACAGTGTAGAGTACTTGCTACTCTGAGTCAAGGGATGAAGGAGGCAACACCAACTTTAAGAAATAAAT

>Gm08ciRNA1025

GTTTGTTTCTGTGATTGATCTTGGATTTACCAATTTTACTATACTGTGGTCATGTGATTTCAATTGGATTGGGCCAGTTATTTGAATAAATGCTGTATGTTGACTGAGTTCTGGTTTGATTTGAGGCAGTGTTCAAGGTTATATGTGGCACATATACATGAGTGGGATATACATGTATTTCCACATTAATTCCTCACGACCAGTATTATTTCCT

>Gm08ciRNA1026

GTACATAAAAATATATTTTTTTGATCACCTTGAGATTGGTGTAGAGTATCCATCAGTTTTGTTAATGACTAATCTTTATCGGAGAACCTGATTAACTACCTTTAGTAAAATCTTCCCTTCCAATCACGTTTTTTTCATTCATAGGATTCGAACCTAAGACCTTGCTTAAGGGAGTGAATCCACTACAGACCAGCCACTTGTTGTATACATAGATATATATGTGGCAGTCGAATTGGCATATCTAGTTATTAGAATTTTTCACAGGAAATTCTAAATGTTCTTCCATGTAATGGGGCATAGTAGCGTCAAGATGTGTATATGTCACCCTAGAGTCCTTTAATCCTTGCTCTACAAACATTGCATATAATTTTTTTTGTGTGCGTGCCAGTGTTTCAAAGTTAGACTATTAAAGCGAGCTTTCAAATTTGTTT

>Gm08ciRNA1027

GTATGACTTTTTATTCTTTCTTTGATTTAATAGTTTATTATTGTCAGTTAAAGACTCTAGCTGAAAAATGCAATGTATTAATCCAACGGTTTCTTCCAGATGGTAAAAACTAAATAACTTATAAATGGTTGCATTGGATGCATTTCTGCTTCTTGAATTTGACTTTACTTTGCCATAGGAAGTGGAGGATTTTAGTTACAGTTGATGGATCCCCATTTTGAATTCCTTTACTTTGCCATAGGATGTTTAGGGAACTACGACCGATTCTTATGAGTTGTTCAAACATATGTATATGCAATTATGCATACTTAATCTTGTTTCTG

>Gm08ciRNA1028

GTATTCAATTTCAACTTCCTTTCTTTTTATTTAGGGTTTTGATCGCCTCAATATACCTCAACATTGAACCTACTTTTTGGGAAATGCAAACCGACTTCTTTTGAACAAACACTCTAATTGTTGTTGCAACATTTTCATTAGTCCACAGTAGACGCCAGCCTTAACCATGAATGAGCGGTTAGAAAACTTACTTGTTCCTTATCCTATGTGAGGATTTTAGTGTTTTTTGTTTGGTAACCCAAGAATCCAGCTAGTTCCAGTTTACTTCATAGTTCGTAATTATTCGTGGTTTGCTGCTTGGGTTTGAATTGTTAGGAAGTCTCACATCTTCTGTCTCAATTTTCGGGGTGCAGCTTGTATATCTGTTGGTCAACTTCACTTAATGCTAATTGGTTTTAAGTTAAAATCTAACATGGTATCAGAACCTATAGTCCATCTTAGTTTTTGCCTATTCAAGTAAGTTAGCTTTGGTTTCTT

>Gm08ciRNA1029

GTATTCAATTTCAACTTCCTTTCTTTTTATTTAGGGTTTTGATCGCCTCAATATACCTCAACATTGAACCTACTTTTTGGGAAATGCAAACCGACTTCTTTTGAACAAACACTCTAATTGTTGTTGCAACATTTTCATTAGTCCACAGTAGACGCCAGCCTTAACCATGAATGAGCGGTTAGAAAACTTACTTGTTCCTTATCCTATGTGAGGATTTTAGTGTTTTTTGTTTGGTAACCCAAGAATCCAGCTAGTTCCAGTTTACTTCATAGTTCGTAATTATTCGTGGTTTGCTGCTTGGGTTTGAATTGTTAGGAAGTCTCACATCTTCTGTCTCAATTTTCGGGGTGCAGCTTGTATATCTGTTGGTCAACTTCACTTAATGCTAATTGGTTTTAAGTTAAAATCTAACATGGTATCAGAACCTATAGTCCATCTTAGTTTTTGCCTATTCAAGTAAGTTAGCTTTGGTTTCTTA

>Gm12ciRNA103

GTATTAATAACCATTGCGACCTCTGTTTATAAAAGTATTATAAGGAAATATAAATAATAATTGAAGGAATCTTCTTGCTGGACTGCTGTATGGGAACTAGGGAAAGGCTTAGGGTATGATGTAGGGGAAGAGTTTTTAGCTGTTCTGAGAGTGGGGAAAACATCATTGTAAGGTTTTTAGTTTCTCAACCTCTTTAAAAAGTTA

>Gm08ciRNA1030

GTATGGTAGAGACCTAAAAATCTAGAAATATTCGAGTTTCTGATTCTGGATCAAATTAGTCATAATAGAGATAAAAACGGATGCTTCTCTCTTTTCTTCTTTTTTTTTTTTATATTAAATATAGTTCAATATTTTATACTTGTGTGAAATTGTAATTGGTGAGGCTACATCTCTGTATTCTTTGCTAGTGGAAAAATTATTAACTTTGTCTCAAATCAATGGAAAAGAT

>Gm08ciRNA1031

GTATTTCAGCTCACCGATCAAATTCAACTTCTGGGTATTTCTGCATATTTAATTATGATTTGTGGGTCGTTGGCAATAAAACTTTTTTTTTTCTTTTTGTGGAGCTGGTGGACTCTGGCTTTATGTATTGTGAGGATTCTGAGGTTTATGATGTGCCTTCGGGTGTAAATGATTGTCAATTCAAATGGTACTTGGTAAATGTTGGGTTGAGGGAAGTTTTGAGGGGGTTTCTTCAATTGAAATGTTGTCTGAGCACTAGAAAGGATATTCATGTTTGGGATTTTTGTTTATTGTGGTTTTGAAGAACTGAAGACTGGCCTAGACAGTGCCACTTTTTTTGTTTCTACTTGAGTGGTAGGTTTGTGTTGTGTGTGTCTAGGCCAATCAATTTTTTTGAAGCTGAATTGTCCAGAAATAAGTTTATCTCGCATGAGTTTTGATTGTTTGTTTGTTCCATTAACTCTTAAAATAGGTAAATTGGTG

>Gm08ciRNA1032

GTTTTGGTTTTCAATTTCTTCCTGATTTTTTGCTATCTGTTCCTTTTTTTTAACTGACATGATATATTTTTCACCTATTATAGTGTAGTATTCTTAGCCATTTGATCATCTTTTTGTGTCCTCTTTTTACCGCAACTGTATAGATTGTGTAGAGCCAAGTGCACTGATGTGCAAGGTCTTCCCTGGAATACCTTAAAGCCAAAATGCAATGATTGTATTGCTTACGCATTAGATAGCTTAATAAAGGCTGCTCGTACACATTTCAAGAAATTTGCGCATCTACTGTTATTGTTTCAATTTTTTTTGTTGGCTTATTTTGTACAACAGTGTACT

>Gm08ciRNA1033

CATGAAAGTTTTACAATTCGAGAAGATAAATCGTTAAATATAAAGAGATAATGGATAAGAAAAAAAAGAAAAAAATACGAGGTTTGGGCAAGGAATAACTAAATACATGTAGCACTATAACATGGTATAAAGGGACAATACGGGGTAGTGAAAGGTTGACGACGAGTAGAACAATAGATTCTTCGTCAATAAACTCGATGTCTTCAAGGTTCTACGATTTCTAACCCGTATCATTTTCCTTATCTTATAATAAAGTCAGTGACTTTCAGATTCTAAGGTTTATATCTCTTACCATGTTCTTTATATAGAA

>Gm08ciRNA1034

GTGAGCTTCCTCTCTTGTCTCTTGTTACGGATGCTTCACCGTTCTCCACAATCTTAGATCTGGACCGGACTCGCTTCGATTTGATGTTTTCTTGTTGCCTTCTATCCTATTCGAATTTCAATTGCTTGCGTATCTGTGACTTCAAGCGCGAGCCTCCTAATTTTGCTTTGTTAGTTCATGCAATTTTGGTTTCCGTGGGATCTAATGTGGAATTGTGCATT

>Gm08ciRNA1035

GTGTTTTTCTCTATCAAATGAATCTTCATTTTCTGTGATTTCTTTAACATTTGGCCGAATTACATTTCTAAATATATTCATGTCCAAATCTATTTTATGAGTGTCCTGATGTACTGCTGAATATGACCATGCTTAAGAGGATTCAGAGATTTTAATAAGTGCCAAACTATGGAAAGCCCAAATACTCCAAAATTGGCAAAGTATTGATGTAGTACATTTATTTTACACCACACTAATGGTGAGCAGTTCTCAAATGTTAATCAATCTGTATTCCCCTTTTCTACCCTTTCATCCGAAATTAATTTGCTGCCTATAAAAATTAAACTATTTAAATATAACTATTATAACTGAATACTGTCTGTTTGGTAGAAGAAACAAACATTGTTGTGTGTGTCTAATGGGCTAATGGTTAAGGAGGGAATCCAAACCACTCCTTAAACTTTCAAAACCTGCAATTTTTTTTTTTGGGGGGGGGGCTTCTAAATGCCATACTGACCTTTGTAAATGGTCACTGATGATATCCTTAATTTTGTTCTTTTGCTGCCACCATGTAATAAGAAGATAAAGTTGGAAATTAAATTTGTATCCACTGCACTCCATTAGTCACTTTTGTGGCTGCCAAACAATGGTGAGTGGCTTTCTTCCATTACTTCATTAAAACTTCCCAAAATTGCAGTATTCCTTTCTACTCAATCCCATTATAAGCCATTCCGTTGTGAACACTCCATCCCATCCCCTTCAAAACTCCCAATCGTATTGGAGGCCTAAATGGTGATTCTGGTGCATGATTAGGATGTTGAATTTTTCTTTTATTCTTGTCACAATTAATAATGATTCTGGACACACTTGATGTTGATTGGTATAGGGTATCTCTTTTTTA

>Gm08ciRNA1036

GTTTGCTTAGAGTTATAAACACTTCTCTTCATTTCTTTCTGGTCCTTTTTTTTTGGAAAATTAAAGTTAGATAATTAAGTAACTTGTAGTCCAAGTTAGACTATGTGTGAAACTGACTTTGGAATTGCCGTGCTATCCTCAACAGTCTATAGCAAAATTTATA

>Gm08ciRNA1037

GTAACTTACATTGCTGGATTAATTTATTAGCAGCTTCCTAATTTTTTGTAATTGTAATTTTTGTTTGTGTTTGGTTTCTTGCAAATCCATTTTTCTATAAGTTTTTATTACTTCTTCTTCATTGTGTGAGTTTCATATACAAATTGATATTGTGATTGTGGTTATACTGTTATACATTTGCTCTTGGATAATAACATTGGAAGCAGTTTGCTTTTCCACCCTGCCACATGGGAAAAAAAACTCAATTTATTCTATAATAAAGCTCATAGCTAAATAATTAGTATGCCGGTTGTAGCCTTGAAAGAAGAAATGTGTGAATGACTGGAGTAAGTTGAAATCCATGGTGTGTAGCTATGTCCTAGCTGCTACATTATAAGGAGTACCATGTCATAAGGAGCTTAGTTTATCTTTGAACTGTAGGCTTCGTAAGTTCTGGCATGCTAAGTTCTTTCATGTTGGTATGGTGTATAGCAAAACTATTCAAACTGGGCAATAAGATTTCCATACGTGGCCAATGAATTTCTTTTCTGTCAATAATTATGAATTTGTTCTTTTTTTCCCCTATCTTGTCAGAACTT

>Gm08ciRNA1038

GTATGGTTGGCTGACATGTGCCATTGAATGTTTAGTAGAGTAAAATATTACGAGGAAATTCTGCTTTTACATCTCTATGATAGGATATTTTAACTGTTAGTTAATTGCATTATGGCACAGTACCAGTGCACTGCTCTTCTATGCTGACCCAGACGGGCTGTGAAGAAGAATAGGGATAGGAGTTGAATAAATTGTCAACAGATTTTGGTAACGAAACTTA

>Gm08ciRNA1039

GTAAGTATCATTTGCTTTATTATGCAAGAATGATGAGTATTGGTGGTTGAGATTTGATAAGGAAGATGGGGAATAATGATATGCATTCTAATCTGTTATCACATTTGAGAAAGAAAATACAACTAAAATACCCTTTAATTTACATACAACAATGGAATTCTGAGATAGTCATGAATAGATGATAGGATGAAGAGATACCCTTGAAAATACAGGCATTCCTTGCCAACCAAATCTACCCTAACCACAGAAACTGTCAACTGCCATAGAATCATGGCCTCCTTTAATACTTGAATTCAACTTTCAGTTTA

>Gm12ciRNA104

GTGCGTTTCAATTTTTATTACAGTTCATTCAATTTCAATTCTCTGTAGTTTGATGTCAATTCAACTACACTGTCACTTTTACTTCACGTGTTAGATTGTTGTGATGCCTTCGTTTTAGAGTGTTTTTATTTGTGTGATTTGGCTGAGGTTAGTTAATGAATTCATTATTACTAATATATCTTTTGAATTGAGTTCCCTCGGCAACCATAGGGATCTTCTTTGTTGCGGTGGCTTTGGCTGAATCTCATTTTTAA

>Gm08ciRNA1040

GTATCGTGCCACTGCCTTTCCGTTTATTATTGTAACATTTCAGAGCAATAGGGGTTATATGATTATCTTATTCCTGGTACTCTTGCATGTTTTTGTATTCTAATATTTCTCTGTAGTCTGTAAAAATTTGTGTTGTTGATATTTTTCTCTTGGGATAGTGAAACTTGAAAGTGAGGTTCAACCTAGCTTTTCATCTTTCTCGAAGTATTTAAATGTGTCTATTCTGTCTGTCTATGCATGTTCTGTGTGAAGATGCAATGGATGATTGTTGTTTTGTGGTGGCTTCTACATTTGTATGTAACAAGATGATTTTATTCAACTAAGTTATCAATAAAAGCTTTACAGGACTGTATAGTGCTGTACGCAAATATCAACTATTAATTTTTAAGGAGTTGATGTTTTTTTTTTTGAAGATCAATTTAGTTCTCTCTCTTAATTTCAGTTAGATACTCAATTCTAATTTGTGCTTCAATTTTTAAATGTAGACTCTAATTTTGTATTAGCCAGAATGCTGATTTAAAAATCCTTTCATAGAAAATCTGCCTTTTTATGATGTAATAATTTGTCAATTATCCTTCTAAAGTTGGTCTTTGTTAAGTTGGTTAAATAGAGAAGTTGTACAGTTGAGTGTACATCCCAGTGCCAATGTATGGGTGCCCCAGCTTACCAGTTAAAAGGAAATTTCAAATTTCGTCATTTCATATTCATCTGTCTCA

>Gm08ciRNA1041

CAATCTCAAAGAGGGAAAGTACAAAGGCTTAGACACCGAAAAAAAAAAAAAAAGTTAAAAATAAAATAAAACAATTATTTTTAAAATACGAGAAAAAAAAAATCCCGCTTTGAAATCCAAGTACGTAGAATCTAATTTCAACGAAACAGCTAGCACACCCTACATCTATCTCAGTTTAGCAAAAGCAAAAAAAAGAAGCAAAAGCAAACCGGTGGCTTTGGCGTAGCAAAAAAAGAAAGAAAGAGAAGAATCCAATTAAAACCTTTATGCCTCCTCCCCAAAGAAGGTATTTATTTACGAAGACCAATCTACTAAAGTAACTGCCACGCCTAAAGCTAATAATCATAAAAAAAAATAAAAAATAAAACACCGACCAGGGGATCGA

>Gm08ciRNA1042

GTATCCCTTTTCCTTCCCGTTAACTATGTTTACTCTTTCACCTCTCGCTCTCTTTCGCATTTCGTCGAGCACCTTCGCTGCACTTAATATGTCTACTTCACTACCTCTCTTTGGATCTGTTTTTTACTACCACATTTGGAGTTTCAAGCTACAAGTATATACTCTAAAAAACCTGTTCATTTTTTCCTTAAATTAAAAAATGTCTGCAATGATCCTTTTGCATTTGGGTGAGTTGTTAGATTGAGTACTTGTTCACAAGGCTTGTTGCTTTCGAGTTGATACCACGAACTTATTTTGATAAGCTAATATTGTGAAATTTGTTGAAAAAAGAATTTATAAGATCATAAGTCACTTTTATAAGTCATTAAATCAAAATAATTTCGGTACAATAAGCTTTCCTCATGGAAAATGATTACTGTAAATGATGCTTTTTTAAAGTCAGGGACTAAAACATCCTTCATATTGGTCCTTATCTTAACATTAGTAGAAAAGGCAGTGGAAAATGACCAGAGCAAATCATGAATTTCAAAGTTAGAGACCAGAGTGAAATTAAAAAGGCCAAGTAAGGACTAAAGTCTAAAATGACTGAAGCATACAATATGAAGACTAAAGTAAGGATTTACTCTTTTTTATTTTTTGACGAGGTAGGGGAGATTACTCTATTTGTTATACTGTTAATAGATGAAAAATATTCTT

>Gm08ciRNA1043

GTACGTTTTCTTCTCTCATTTCCCTCTAAATTCTGCCTATGATGAAGGCTTCTAAGCTTCAAAATGCTTAGACAAACCACCTTTTACTATTTCTTCAACACATCCTCTGTACTATCTTGTTAAGAATATGAAAAGTGGGGTCACAATTTCTGTGATTTTTGACAAATTTCAGCTTTTGATATTAGTGTATGTTAAAGAGAGTGTATTAAAGTGTGTGTTGCTAGTATTTTCTTTGTATGAATTGGCCTGCTATGAATTTTTTCTCCTCTTTGTAATCAGCATCATTTGGGGAAGATTTCCAACTTCCTTGGGGACATAAATACTT

>Gm08ciRNA1044

CATTGATGATTGTTGTTGATGTTAGAGAAAAGAGTCAAAAGACGGTAGTAAAAAAAGGGAAATACGTATGTATAATGTATTATGAACGTCAAAGTCAAGCTAGCGTACACTGAGCCTGAGAAGGTAAGAAGGGGGAGAAGTAATTAAAGGTGCAGAACAAGAAGTGGTTCAAAATAAATAAATATAATGTATCGTGGAATTATACAACTTCTGAACT

>Gm08ciRNA1045

GTGACAACTTGTTGCACTTATCTTTAGTTCCCAAACATGTTATGGTTTATTTGGTTTAAACAAGCATTGCTGATGCATTTATGCTCCCCATGGTAAAGATAATGCATTTTTGTTGTCATTTTGCTTAAAATTTGCATTGTTAAGAGGAGTTGCATAATTGTCCCTTTAATCGGCTATGGAACATGAGTCTATCTTTCTATTTAGTTTTTAAACCCTTTAGTAATGAATTGAGATTTTGTCTGGTTTGTTTGTTCTA

>Gm08ciRNA1046

CATAATGAACATATAAACATTATCTTACGAATTTACCATAAAACTACGAAACGAGATAAGGTACGGAACAATACTAAGAGTAAAGATCAATCACAATCCTATCAACCTGGTACTTCCAAAAATACACAAGAAAGAAGAACCTCTTTGTTTTCTTCTCCCGTTATTTCAATCAGTCTCTTTATAAGATTAGGTTAATGAAACGACGAAAGTAACACAAGGGGTCCGTTACTAAAGTACAAACTAGGGAACCGGAATCTAATTTAAAATTAGAACACTGGACAATTTCACGGACATGAAACTCCTAGTCATATGAACATCAAATAAATCAAACTATCGTTGATAACCTACGAACCCTAAACAAAACAACACGAGTTAGGTACGTTGAACAAAGGTAAACTTCATATCCTACGTAAAAACTCGTACGTTTCTCGTGGTAATATGACTTATAAACATAAACATAAGTATATCTTCGTTAAACCACTACGA

>Gm08ciRNA1047

GTTGGTTGGTATATTTGTTTGAGAGCAACAGATTGATTATCTTGTATGCATGTTCTATTGTTCTTTCATTAACTGAGGTCTGTTGTTAAATTACAATGTGTGCCTGGTCGTAACTCAACCTAATTAAACTTCTCACCGTGCTGAATCTTTCATCATTGTTAATCAAAACCTTTGCCTTGACTCTTCATGTATGGTCCAATCATCCTGTAAAATGGTAAGTGGTTCTATTTTATTTCACATGTAGTTCTGCTGCATATATTGTACTAAATAACGGTTCGTTGAATGTGAGGGCATAAACTTTAATGATTCTGCTACTTTAGTTATTTGATCTGTAAGATGGTTTGCCTGTAGCCAAGAAAAATCACGAAAAAAGTATGAATTTTGGAAGAACACCACTGTGGTCAAGGTGAGCAATAACAGCAAAAAAAACGAATGAATTGTTGAAGCCTCTCACGCTGACCATTGGATACCTGACCTTGATAAGTTTCAACACCTCCAAACACACCTAGTTCACTTTGCCACTGACAAGAAGAGAGATTTTTTTTGTTGTCAAAAATGACAAGGGGCAATGGTTTATGGCAAACTTCGAATATGAAAAATAGAACTTACTTATCATAACTTTTTCTTGAGTTTCACTTGTTAACTCTTGTGCAAATCTGATGAGTGATGATTGCTGAACTGCTCGGAGTCACAGTTCTGTTTTTGTTCATTTTCCTTGA

>Gm08ciRNA1048

GTGTGTGTTCTTTTATTGGTAATGATTGTTTGCTAATGAAATGTCCCCATGCTTTCTCCTAGTACGGGATTAAGATTTGTTCAGCTTCTTCTGCAAATAGAGGTGGTATCATAAATAACCTGTGGACAGACTGTTATGTAGTCTAAACCTGGGTATTAACATATAATCAATGTGTTTGATAATGATAAGTGGAGCTCTAAATGCTACTAAATTATGGTGTTGTTTTGTAGTTTGGACAAAATTTGTTAGGATGTATATGCATTTGTTCTATTAGTAAACT

>Gm08ciRNA1049

CAAGCACCATTGGGTAGTTCGATAATCCCAAAGGCACAAGGAATTGTGTGTGTGTGTATATATATATATATACACATATATAAAACTAAGCTAAGCCATGAATCCCAAAGAACGTCATGAATACAAACTAACTTAAACATATTATATTTAAATTAGAAAGTCAATAGTTAAAGATCGTCTTCCCCTAGTCGCATCGCACTAAAACATCTGGGGGAAAAAGACAACTGACTTAAATCCTTTTTAATATTTTTAACGAACGTAAGTCATAGTAAACGGAAAAGAACGAGAACTACTATATCCGAAGAAGGATAAAATAAAGGAAGTACGGGAAGCACGGTGATTACTTTAACAACAGGCAACTAAATGTAAGTTTACCTAAAACCGTAACTTAAGTGCATTCAAT

>Gm12ciRNA105

GTAGGATTTCTCCTTTTTCTCTTTTTTCCTGTGCAGAGGCAATTTTTCTTCATTGCACTTTTCAACTCTGTGTCCTTGTTTTTTAATTTCTGGGGTGCGTTTTTTTTTGGTGTTTGGAAATCCATTCATTCCATGTTGGAGTAACCGTTGAGAGGATAATAGTTGGGTTGTTTGTCTCGACTTTTTTGTTTTCCGAGGTGGCTTAAATTTTATGAAGAAAATCTTTGCTGTAGATTTTCTTAGGATTTTATTTCAGATTCACTTTGGATGTTGTCGATACTCTATATTGCACCCCCCTTCCCACTGTTATTAATTTATCACTCATTTTAAAAAAATATTTTGTTGAGTCAAATCAAATGGAACTCTATTTTTCCGACCTTACTTCTTGCTTTTGACGATTATGCTTTGTGGAGAACATAAGAAAAAATATATGGTTTCTCTTAAAATTTTTTTACCTGGAATTAAGAAAAATATCGTCTCTATAAACTGTTTTGAGATCGTGGGGGATTGGGACCTTTCTATTTTGTATGGGATATAGATTATTTTATGTGTTACTTGATAAAGTTTTTGTGATTCATATCAATTTCTTACTGTGCAATCGGTGCCAAATCTATTGTTCGGTGAAGAAACGGTAGCATAGTCAGTCTTGCTT

>Gm08ciRNA1050

GTAAGTAACATATCTTGCACAACATTTAAGTGTGATTCCTCAAAGAGAGAATGAGTAACATAGACAGTAAAAATGAGCAATGTATGAAAATGTGACTTTTACTCAAATACATTGTCCATTTTGAATCTACCTCGTGTTTTCTCATTGTAACTTGCAACTTTCATCATAAAAGTACAAAATATATTAAATTACATCATTTGCAAGCCACTGTACCTTTGCATTAATGTGCCAGATCCAATGACCAGTGCAAAAGTATAGTGTCTTGTGTTGCATTGTTACTTGCAAGACTATTATTACCTTTTGACTCTTAGAAGAAACAATGTTTAAGTTAAATACGGGTTACGGCATGTTACATCTTCCATCAGGTGATCAATCTATAATGCTCTTGCTT

>Gm08ciRNA1051

GTAAGTAACATATCTTGCACAACATTTAAGTGTGATTCCTCAAAGAGAGAATGAGTAACATAGACAGTAAAAATGAGCAATGTATGAAAATGTGACTTTTACTCAAATACATTGTCCATTTTGAATCTACCTCGTGTTTTCTCATTGTAACTTGCAACTTTCATCATAAAAGTACAAAATATATTAAATTACATCATTTGCAAGCCACTGTACCTTTGCATTAATGTGCCAGATCCAATGACCAGTGCAAAAGTATAGTGTCTTGTGTTGCATTGTTACTTGCAAGACTATTATTACCTTTTGACTCTTAGAAGAAACAATGTTTAAGTTAAATACGGGTTACGGCATGTTACATCTTCCATCAGGTGATCAATCTATAATGCTCTTGCTTA

>Gm08ciRNA1052

GTATTGTACACTCTTACCAACCTCATTAATCTTAATTGAGCCATATTTTTCTTTCTTTTCTTGCTACATGGAATGAAGCAAAGCTACTATGAAAGTAGTCCTGTGTTGGAAAAAATTACCACAAAAAAAAACTATTTATGTAGTACATGTTTAGATTGTTTTTACTTAAATAGTTTTTAGTTATGCATTTAAAAGAATGAGAAGAAAAAAACTACTAATCAGTGTAAATATTTGTTCAACATTTTGCATCATATTATTTTCAAGTACAAAGTTTGGAGATATTTTATTAAAATAGTCAGAGAACAATAACCCCCGCACACT

>Gm08ciRNA1053

GTTTTCTCGCAAACCCATCTTTTCTTTCGGTGTAAAATTTCGTTCTTTGTAACATTTTTTTGTCCTTTTTGTTTGGTTCTGCATGTGGGTCATGTCTGTGCAATGTCACTGCCGTGTGTGTGTATTTCGCCATCTCGTTCCCATTTACGTGTAATGTTTTTTTTGTTTCTGTTGTTGGTTTCGTTGTTTCTGGTGGTTATATGGGAAAGTGGCGGATCTTAGAAAAAAGGGTCATTTAAATTCAAACTGTGTTTTTTGTGTGTGGCTAACGTTGTTTTGTGTCGTGGTTACT

>Gm08ciRNA1054

CATTAAAGAGAAAAGACTCAACGACAAATTACAAAAATGAAGACTTAAAATAAAACCAAATTAATGAGACGCCCAGGGACTTGAAAATGGTTTAAAAATCAATACAGGGACGTGATGTATCAAAAAGAAACCAACAAAAGGTGAACGGATAATATAAAACATTAATACTTGTTGAACGTCACAAACCGATTCTAAAAGTGATTTTGTTTGTCCCTTTTGAAACTAAGGGTAACGTGAAAAGTGTGAAAAAGTCATCGTAAACATCCTCGAATAATAAGGAAAACGAGTTGAAGGTGACCGAAACTTGTTGTAGATTATATTTGTTTGGTAATTAATCAAACAATGAAAGGCATGGTACTAACCATCTTCTAGAGAGAATAGACATTCCAATCTCTGAGGAAAATATCTAGTTACATTTAAGGAACGAATTTGAATCAAAGACACAGTCGGTGGGGAAGGTGGGATTGGTTATTGGTACATATAAGAGTAAAACCTACCCTTTTTCACCCGTCAAACAGACTGATCACACTTTCGTTTTCGATAAGACACGATATCCATTGAACTCCATTAAAACAACTCAGCGA

>Gm08ciRNA1055

CACTAAGTAAACTGGTCATTATCGCGAAGGACTTGATTTTTCTTGTTTAAAACACTTAATTAAAGTTTTCCCCACACCACTAAAAAAACTATCTCCCCCCACCCTAGCTAACTACCCCTACACCCTAACTTCAAACCACTAAAACCCCATATAGTCACAAAAATAAAATTAGACTATCATCGTACCACGAGAGTTGTTAAGTCTAAACTAGACTTAAAATCATGTTAACATGTCACTGTACACAACACAAAAATCACCTGACTAAGTCCTCACAGACTCCTAAAGACCCATAAACTTTAAAACTTAGCGGATACACGTCTGCTCCTCAACTAGAAACCACGTAAGTTATACACTTAATTACTTCAGAAACCATAATAACCTCCCTAACTACCTCACCATCCTTATCCTAAAAATCTAGTGTGAAAATGTTTTTTAAACCTCCATGACACACAGCACCATTTTACTAACAACCTTTATTAGATTCAGGGTGTATACGATTTTTATTAGGGTGTAATCTTATATATTCACTCCCCGTTGGGAGTAGGGAACTCAATCGAAAACCTCAACTCAATCCGAAAAATGTAAAGACTAGGATCCTGGTGTGAAGACGATCAGGAGCGAAACTCCACTGTTTGTGGGTGGTAAACAAGTGCGGGGCTCGCGGGTCCTTCACTAACCTAAATAAACAGAAGATTCTTGCTTACAAACATGGAAAAGATGAGCAAGTACTGAACTGACGTACAATTTAAAGTATTTACAATAATTTCTAGGCAGAAGGGGATCATTTTTTTATCAAAGAAAAAGACCCATTAACAAGTCCCAAACGTAATATAATGTAGTAAACAACCAGCGCGACGACCCAGAAAAAGAGAGCAATAATAATAGTTACACTAGAAAAACGGAACCCCTACCACCTATATGAGAAAACTCCAAAAGT

>Gm08ciRNA1056

GTGCTCTCTCTCTCTCTCTTTCTTTCATACATGGAACCAGATCGAGTGCCTATGAATTCATATAGTCAGAGAATTGGAATCGTCAGATGAAGATCAGATGATTCACATCTCAAATGGTCCAATTTATTGAGTTAAAATCTGAGCTGTCCAATTTTCATCCGACAGTTCCAATTCCCTGGCATGAAAAAATCTGACTATAGGGAAACACACAAAGCATAGTGTTTTTTTTTTTTTTGGTTAAGAGAAAGGAATCGAACTTCAGTGATTTACAACAACTAACGCACAATCCCCCAAAGTTGTACAAATGTGCACATGGAACAAAATTTGGTGCAAATGAATTCATTGTAGTCAGGGAACTATGAAGATCCGATGACTCACATCTTGAATGCTCCAATTTACTAAGACAAACTCTGAGCCGTGTGATTTTCATCCGACATCTCTAATTCACTATGTGCTTCATTGCATGAAAAGTCTGACTGTAGGGAATCCAGATCCTAGAGTACACACAAGGCACAAGCATATACTATGATGCACAAAAAGAACAGAAGTGCATACCAGCATTCACACTGATCTTAACTGTAAACATTTTTAAGTTTCTA

>Gm08ciRNA1057

GTATCCTAGCACACATTATCTGTGCCGTATTAGTTTCATTACCATATGAGGCTATTTATGCAATATAAGGAGAGATTTTTAAAGAATTGAATTTTGAAAGATAAACAAGTAGTCTGCTGTTATGTGCTAATTGATATCTGAATTAGTTTGGGTTGTTATACATTTAACTTTTGAGAATTGAATCTGGATTCTTGTCTTTTTGCAGCTTATGCGCCTATCATTTCTATAATTCAGAAGATGTTTGTACTTTATGAACAATTCAATTCTATATGTTCATTCCTTTTTTATTTTATTTTATGGACATTTCATGCATTTCTTCAGGGCAGCCTAGAATTTCATCATGCTTGCATTGATAACAATAAACCTTGAATTTCCATGTTTTGCTACATCTTCAAATTTTTTTCCAT

>Gm08ciRNA1058

GTATGTATCTTGATTGAGTTGGTCGGAAAAGCCTGTATTGTTTATCTTAAAAGTTTATCAAAAGCATTTTTGTTTGCTGCATAGGTAATTCTCATGTTGCTCTTAGATTCTCAAGACTTTTTTCCCTGTTATGTGGCCTTTTTTCTAATGATCCAAAAGCAAGTTTGGAGAGTCCCCAAAACATGCATGTTGAAGTGAACTATACTCCTATTTATTACTGGTGCAGTAGTATCAGTTAATTTTTAAGCTTTTTCTTATCCTGTATTGCCCTTCTCTCCTATTCTTGTGTAATTGAGTTGTTAGAAGGGATAAGGCTACAATTTTTGTTCATTTCCTGTTTACCTACTATGTTTCCGTGGGTGCATGTCTTATATTCACACACTTCATAGCATTGTGCTTTGGGAAATGTTGCTTATGGAGCCTGCAAGTATTTGTTGAGCCTATTTTTTTACCAGCATTAAGAATCGGAAACACTAATATACTTGTCAAAAGTTGTATTATCTTATTGTTTTATTTTGGATTTTTA

>Gm08ciRNA1059

CAGTCACTTGAAAAAGCAATTAAAGGTGAAACGTTTCTTTTGGTTTTATCTCCGGTTTTAATGAGTACGTAAAGTTCAAAGTTTAAAGTTGATACAACATAAATGACGTTTACGACTTACCATGTTTTTTATTTTATTTAAAACGTGTACGATTTCGAGATCTAGTTTGTTACTAAACATTCAATTGAAACGAAACCCTTTACTTATCGCCGACCAAGATATGGCAAAACGA

>Gm12ciRNA106

CACTCCTCCTTCAAATCCTAAACACCACCATCGTTAATTAAATGCAAATACTTACCAACCCTTGAAATCAAGAAAACTAAACTCCATAAAAACAAACTACTACGAGACACTCACACTAACCAACGAGTACGCTATTGTGAAAGGGAAGACGAAGTCACCTAACGACCTTTAACAAGAATGACTAAATGTAGCTGACTGACTGAACAAAGAAAAATGACAATAACCTTACTCTTTAAACACCTGATAAAGTACAAGACTAAAGTTTAAGATCAAATTAAACCAACCAACTCT

>Gm08ciRNA1060

GTTAGTTCACTCAATTCTTGTGAGATGATGATACCTGCTACTTGACCAGTTTAATGTTAAAAGTTTTATAAAAATAATATTGAAAAAAACACTTTTATCTATTTGGGGTTGATTTGTTTATATAGCAAATAAGTTATGCACGGACAGTGTAAAACGTTTGTCGAATTTTACAATAATTCTGTTAAAAGTCATACTAATGGTGATTATTTATTGGCTGAGAGTAAAACTATACACTAACAGTACATGGTCATTAAACTGTTGTTTATATATATTGCAAATTTCTTTTACCCAGATGAAGTTGCTCTTTGAACAAGTAATCAAAATCAAAACAACTCTAAATCGGTAAAACGTTTTATTCATATGACTTTAATTTATTAATTTGATATTAATATGGTAAAAAAAAACTTTTAATATTGTGTGTACTATTATACTTGCTTTATGCATGCTCCTTCTCAAATAACCTTTTCCATTGCAAACTTGAATATGCAGCTGCGCTAATAATGAGTAGCTAAGGCCTGAGGTGCCATCAATGACTGATAATTGTTGTTTTAGACTCAGTTCTGATGTTGTGGTTATTTA

>Gm08ciRNA1061

GTTCATTCCTTCTTTCTACCATTATATGATTATTCATTTATCTATGCTTCTGCAGCTCCAATTGTGTGCACTTTGTTTCCGGTGCTGTTTTGAACAATTTGTTTCTGCTGTTTTTTTTATGCATTATTGATGTTTATGTTAACAGTGGTGCAATTAGGATTGAGAATATGGATTGTTGTA

>Gm08ciRNA1062

CATTCCTAGATATGGAGAAAATCTATTAATTACATAAGGTATAAACTACGTGTATTAAATAAGTTCCGTTTGTCTTTGAGTGGTATCAATATGTATGATAGAGGTAAGGTTCTGAGAGAAGAAATTTACGTGTAAAATATATAAATAAATCAATGAAACGAACAAGAACCCAACTTAGTCAACTTTCAACTTGAAATCCATCTTCCGTGAGGAACCCCAAAGACTTTTACATGGTGGGACTATCGTAAAACCAGAGAACGAGGGAAGATAGACTCTACGCTCGTTAACTTAAAAATAATAGAATACAATGAGTGTTTATCATAAGTTAATAGATTTTAACCTTCTTTTTTTTTTGACCTTAAAAATAGTATATAGGAAACATCTGTATTTCTAACCATTCGTCACTGTAATCTCCCATTATACAGTATACACGTAGTTGAGACTGTTCGAGGACGAAAAGTAAGTTGATTCCTGAATACAGAAT

>Gm08ciRNA1063

CATCCACTGAAATTAGTAGGTATTAAGACGAGAGTCACACAAACCAATGTTGGAAGGAGTTTTTATCATGAAGTTTTTTGTAACTACGTAAGACAAAGAAGAATCGGATCTAAACAAAATCCAAATCAATGAGTAAACTAGAGATATCAATGCTTTTTTAGGGAAAATCAGGGACATGCCGATATGAATTTGTAGGAGAAAATCAGGGATGTGATGTGTATGAAGAAAATTAAGGAATCAGGGATGTGTAGTAAAATTAAGATAGGGAAAATCAGGGACTCCTGATTTTCCCTAATCTTTACCATACACATCCTTATTTTTCCACTACAAAATTCATATCCCTGATTTTCCCTTTCAAAAACATTGATATCCCTGGATTAATCTTAATTTGGAAAAAAAATAACTTGAAGATCGAACGTATAAACAGACGATGTAATAAAAGGATCAGAGAGTCAAAAGACGTTGAGT

>Gm08ciRNA1064

CACTCTTATTACTAAAGAATATAGAACGTTACGACTTACGACAAAACTTGAAACAATTGATTTGATACTGGACAAACGACTAACAATAAGTCCCCTGTTCAACTCTTTGTACGAAAGCACAAAAGTTATAAAATTCAAAGGTTGAATGGTAATATAACCCTAAACTAACTTCCCTATAATAAATTCAACTCCAGATCTCAATAACGGAAAACATAAACTATTAAACCTTTCATCTATCACAACCACAACAGACCACGTACTAGAATTTAGAAAACCGAACGATTAGGAACCAATAAAAACAACATACTTCTAAGTAACATGTCAATAAAGATAGGGACAT

>Gm08ciRNA1065

GTCAGAAGCCCTCTCTCTTTCTCTTTCTGTCTTCCGATTTCGCTTAAAAATCCGTTTTTTATTTCCCGTGCATGGCTTTTTCTGAAATTAATTCTTACCCAGATGAATAAGTGCTTTGGAATTTGTTTCTAAGAACCCCTTGTTTTGTGGGTAGCTTCCTACTTTGTGGTGGAATTGGAGTAATCGATCTGGGGTATCTGCTATGAATCCCAGCTTTGTGATTGTGTGAAGAAAGTTTTAATGGGTGTTTGGAAACTCTCGATTCTTCTTTTTTCTTGTTTTGGGCTTTTAAAAGGCTTGAATTAGCGTATGCACACTTTGTTGTTTCAGTTTCTGAGTGAAATGAATCTTTTTTTTACTCACTGGTGCTTATTAGGTTGTTCAGTTTTGATTGGTAATGGAAAATTCTGAAGTTTGTTCATAAAGTATTAGTGGCGTGATAATTGTTAAGCTCTCAGAATGTAAATTTAGCTCTATTGTCTTGTGTTTGCGTTCTTTGAACCTTTTGATGTATTCTCTGATATTTTATCAAAGGTTTTCTTGTTTACTCACTCACTCTCCCTTTCTATCTCAATTTTTTTTATCCGTGCACATGAGCT

>Gm08ciRNA1066

CATGAAAAAGAAAAAAAACAGTCATTTAGAAGTGAACAGTTACTTTGTTATAACAAAGATTCTAAGATTTAACACCAGCGCCAGCGCTAAAGTACCGCTGGGAAATTAGTGTTGACACCAACGTTACGCCAGCGCTGACTTTAGTACCAACGATTATTAAAAAATTTTGTGTCACAGTGATACAAAAACTTCCCCACAAAAACTAAAAGGATCCACGTACAAACGGAAACAGTAAATATTTATAAGGAAAAAAAAAAGTTATATCTAAAGTTTCGACACCCAAACTACCACAACATCTAACCCTTAAGAAACACCCAAAAAATTCCAAAGAACAGAGCAACAAAAACCTACGACGTTCGAACGATCCTAAGTTAGGTACCCTAGAACATCAATTGGCTTAGATCAATTTTGAATATGTTACGAGCTCAAACCTGATATTGATTTGATTCATTGTGAAACCGGGAAAACAAAATCAGGAAAATGTACACTTTTCTCCTCCTTTTTGACTTTTCGTATCGTCAGGATTATTCACTATTACTTTTCAAAACAATATTGTATATTTCATAAACAAGTCTTAAAAGGATTTACCCAATACTCACATCAACCAACTCAAAATTCCAATATAACCGTTCAAGGACGAAAACAAACACTTTTACGATCGTATATACTTGA

>Gm08ciRNA1067

CATAAACAGTTGAACTGGGAAATAAAAAATGAGTAAACCCATAGGAGATTCTAGGAGTTAAACAAATCTAGGGGGTGGTAAAGACCCGATAAGCAATGTAATGTCATGTACTAGTTTATTTATCCAAAAAAATCATGAAACAACCCTAACCTATTTTTATGCTAAAACTAGGATAGGGTACATGGGGAAACACGGGGAAAATTCATAAAAATTAACGGGTAAAAGATAGAGAGAATAAAGCACATCAAGAACGAGAAATTAAAATCGTAACCTAACAAAAGAGAAGCAAAACAAGAAGGAAACTAGTGTCTACCATTATCGTTAAAAGAAAAGGGAAAGAGTTAACTTATGGGAAAACCACTCCCGAAAACCTTTTACTTAAAATATGAATAATGCCACATTTCCACCTACTTTCTCTTCCCCCCTTCACCACCAAAGGGAAAAAAAAGATGTTTAGTATTTTCGAAGATT

>Gm08ciRNA1068

CATATTGAAAAGAAATAAACGAAACGACAAAACTGAAAACAAAACGGAAAATTCGGAGTTAATTAAATTTACTTTCTCTTCATAATCTATAATGATGGCAAAGAAAGTAATAGAGATGGTTTTTTTTTGAGATTAATAGTCAACACAATTGTTCCATTGTAGGAATGCCATGGTTCTTTATATCTTAATTTACAATCTTAATTCGCATACATATTACTCACCATAATCGACAATGACTGAGTCCACTAATCATGACTCACTGAGATTAAGTCCCTTACCAAGGAGAACATTAGTAATGAAATATTACACGTATCATATTTACATATTTACACAACTCACT

>Gm08ciRNA1069

CAACTCAAAAGACTAAGAAATAAGAATCAGAAACCACTTGAATACGGTAGTGTAACAGACCATTTAACTTAAATCAAAGGTAAAGGAGGAGGTATAAATTATAAAGTAGTTGTATATATATACGTGAAAAAAAATATTCGTGTATGCACTTCGATAGAACGATCAAGGTTATGTCACGATTTGTATGAATACCAAATAGAAATTACGTAAGTATACAACTAATTTCTCTATCGTAATCTATTTAACTGTGTCCTTTGAAATGAACTCCTTTCACGGACACTCTAATAACATTTCGGAAGACTTATCAATTAGAGTTAAAAAAACGAACAATACCCTGTCAAACTTGAAGTAGTTCTAGATTAAACTCGTGACCGTAGACATGACATACTCAAGTTTACTTCAAGAACTAGAAATTATTCGGTTTTACAAACTGTATCTAAGTGCCAAAACATAATATTATTTGAAAACTAGAGGACACAAGACGTCTGCATTTCGACGAACTAGAAAAATTTAACAGTGACAGAAAATATATACATGTATTTATTTATTATCATATAACCTCGTACCAACAG

>Gm12ciRNA107

CATTCCTATACGAAAGAGAGACAGTTAGTAAAAAAATTTAATGTCTGTAATTATAATACTGATATACTCATAAAGTACAGACTAATGTTCAATACTCTATAATCAAACTGTAGTAATCAGTTTAGTAGAACCTCGAGAAACAGAAAATGTTAAAGACATACACAAAGGTATTTACATGTATCTACATATGAAAATAATAAAAGAGTGACATACGAAAAATACAGCGGAGACTATGATTACTTCGACTTTGATAATACAACATCTTAATGAACTAGAGTTCTGACGAACGTTTTTTTCTTTGTGCAGAAAAAATCAATCGACCGATTGTGAAGTGTAACGAAAACCTGAACGTAGCGAGAGACTTATAAAGATAAATTTACAGA

>Gm08ciRNA1070

GTACCCCCATCACATTATTCTCGCACCCTTTTGCGCAATTTTGAATTTTTCGCGGGTAATTCGACACCTCCTTTGTTGTGGGTTTTCGTTGCAATGCAATCACCCAGTTCGGTTCATGTGCAAACGATGAAACCCACTTTCAATATTACGTGAAAAAAAAATTATATTTGAGGGGAAAGAATGAAATCCGTTAATGGGGTGGTTGGAATATTTGAGCATGTTAAAAGGGTTGTGGCGGTTTGTAGGCTTTGATATTGTAATTGTTGTTGCTTTGTTGGTGTTATTACTACTGCTTGAGTGTGGCTGA

>Gm08ciRNA1071

GTAATAAAATCAAATAAAATCTCATCATCTTCCTTTGTCTATACCCTTTCTAGATACATATTTCTTTCTTTCTTTTTGTATTTGAATCTTTTTTTTTCTTCTCTCTGAATTCTCTTTTGTTTTCTAGTGCTGGTGTGTGATTTGAAGGAGGAATTTTAAAGTGGCTTGTGGTTTTAGAATTCTGTGACTAACGCGTTCTGGGTCACCGTGCCCTCCCTTGGGTTCCGCTGCTAGTGTTTTTTTGTGCAGTAAAACAGATAAAGTTGGTAGCTTTATTGGAATATGATGAAACTTGGCACTACCCTTTATTGCTCTTGCTAATTTTTAACCTTTGCTGGTTCTGGGTCTTCGAGTATTTCACTGGAAATTTTCAATTTTGGGGGGGCTTCTGCTTCTGGTTTTGTTAAATTCTGAGTTGCCTTTTGAGGGAACTCAAGTAATCAGAACTCAAGTAGTCAAGTTCAAGCTTCCTACTACTTCTCTCTCACGTTTTGGGGTTGAGTGTTGCCTTTCCTTCCTTCCTTCTTTTGCCTAAGTTGGTTTACACGCAGTGAAAGTTTGTTTCTATTTTTGGATCTGCTTAAGCCTAGGTTTTTGGGTGGTTTGTTTAAGTTTGTGTTTTGTTGAGCTAGGTTTTGGATTAGTGAATTTTGTGTTTTTCAGCTAGTACTAGGTTGCTCTGTTAGTAATTAGTATTTGATGTAGCACATGTTTCTTGCTGTGGTCTTGTGTCTTAGTTTTCTTAAACTTAAGGGTGAATTGACTT

>Gm08ciRNA1072

CACACAGAAAAGAAGAGTAGACGTAAAGACTAGCCAAAAACGAAACCATCTTTCCAATCTAAATAGACAAATATAACTTTTGACACGTAAATCTAACTAAGGTACCGAACTTAAAACCGATAGTAAGTCACTAAACCTATACCTAATAACTTAACACTTAAAAAAACACGAAACTCTACTTTTAAAACTGAGTTTTACACACAGGCAAAACTTAAAACAAGTTAGAGTTATAACCTTTAAACCTTAACCACTAAC

>Gm08ciRNA1073

GTAAGAATATTTATCATCTTTTTTCATCTTACCTTTACACTACAATACATAGATTGCACCAATTCATTAGCCATTCCTTACTGGACTAGAGGTCTTTTTTTCTCTTCCTGGGAAACATCAATTTCTGAGAATCTTTTAACACCTTACAAGGATTCTTCAATAGACATTAACAATTGCACTACTTTACATAAATGCTTCAGTGTTCAATGCCTGCTTGGTGTCTGCTGCCTATATTCCTCAATGTGGCTTCTAAACTCCTTTGAATTATCTATTTGATATTAA

>Gm08ciRNA1074

GTTTGTTTGATAATTAGTGTATATTCTTGTTTTTCCAGAAGGTTTTATGGTTTAAGTGTGTTGTTCTTGATCGGGAGAATATTGAAGTTTCATTATACTGTTTGTTTGTGCTCAAGCCTTGTTTCACTGGCTAATTTACTAGTTGATAAACGGATTTCAAATTTGATGTTCTTTAATCCGCTCTAGAATTCATTAGTTAGAAGTTAGTAACTTTGTCATGAGATTTGTGCAGTACTTTAAATGATCTTGTGCCTGACCAATTCAACAGTGAACTGA

>Gm08ciRNA1075

CATTCTTATAAATAGTAGAAAAAAGTAGAATGGAAATGTGATGTTATGTATCTAACGTGGTTAAGTAATCGGTAAGGAATGACCTGATCTCCAGAAAAAAGAGAAGGACCCTTTGAAGTTAAAGACTCTTAGAAAATTGTGGAATGTTCCTAAGAAGTTATCTGTAATTGTTAACGTGATGAAATGTATTTACGAAGTCACAAGTTACGGACGAACCACAGACGACGGATATAAGGAGTTACACCGAAGATTTGAGGAAACTTAATAGATAAACTATAATT

>Gm08ciRNA1076

CATCATTTAAAGACGATGTGGTAAACACGATGACAATCGTATAAAACTGATAAGTACGAAGAAAAAGTGGAACCAATATAAATGAAGTAACGTTCTGAACCCTTTACAATAAAAAAATTTTGTCGTTTCAATATTTATTTTAGAATATAAAAACATAAAATAAGGTAGATATAAATATATAAACTTGGGTGACTAGAATAAAAAATATTGAACGGAGGTGACTAAGTCCCAGAGACTAGGTGGTGGCAGGAATTATAACTTTGCATACCAGTAAGTAAAAAAATACCTTAATTAAGTAATTCCTAACAAAAAACAAAAATATCATTAAAATTAGTTCGATAGTAAAAGAAATAGTCTAAAACTACCAATTATAGATGGTAAATTACCTAAACACAACGA

>Gm08ciRNA1077

CATTAATAAAGTGAGAAAAAAAATTAATATTTTATAAAGCATAAAGATGAACAAGAACCACCACAAGCACACAACACGAAAGTTTCTTTCAACTAAATAAAACACTTTGCTACTTAGCGTAATGGTCATTACGTAAGTGGGAACCTATAAAAAAATTAGGGGTGGAATTCCAACATGATTTGGTTTAGAACCTGGAATGCTTTGAAAAGTTATGAAAACAAAGCAAGCATAAATAAAGTAACACCGTAAAATAAAAACAATAGCTCCATCGTGATCGACGTGGACTATTTTTAAATAACACAAAAATTAGTCGTTATTTTTAAATAATAACCTTCGTGTTGTATAATTAAGAGGAATATGCATCATGTTTTTCATAATTTTTTATCGTAAATTAAATTATAGAACCTTTGCATTTTACGAAATTTATAAAAGATGAAGTATACTAATCTTCCTCTCAATCACCCTTCGACCAAAACCCCCTAAATCTTAATAAGTATTTAGTGGTAGAGAGAAGATATACTCTAAATCTTCGTAACCTCCTCGGTGGACAGGGTTATAAGGAGACGGAAACCGAACGTTGTAAGAATTTTGAAAACAAACTGAAATTATAAGTACTTCTAATTTACTAAACAAGGATTCTTCGGTTAAAATGCACAAGAAAACCCGTTACTTAATCAGAACAAT

>Gm08ciRNA1078

CATTATAGAGAAAAATATAGAAAAAAGTTAGAGATGGTATGTGAATAAATACGTGGAATACGGTGACGAAACAAGAGATTGGAAATGAAAAAACCAACAACGAACATTGATAGTAAGTATACGAAGAACTACAGCACAGACCAAGACTTAAACATCAACGATGACTGTAGGAATCCGAACACACGGTCCCTAACCAAAGTTTCCCTTTAAGAATATGACTAGTAAGTAACGAGAGAATCAATCGAGAATAATACGAAAATACCTTAAAAAAACGAACGAGAAACATAAATTCTTTAACGTTTGCTACAAATATTTTCATTTAGTATTCCCGATAGTGAAAACTACAGAAAACAAACGATATCTTCACAAACTAAGAATAATAAATAGAAATGA

>Gm08ciRNA1079

ATATCCTTTGATCCGTTGAAATTTTACTGCTAATTTTTCACAAATTGTCGTTAATTTAGGGATAGGGTTGACTTTCTGTTAGAAAATTCGAGGAAAAAAATAAATCACATTTCACCCTCCGCTACCCCCATTTGTTGTAGATCTTTTTGGTTTTACCGCAATTAATAGTTTCATTCAGTTTGGATCCATGGAAACAAACACAAGAATAACACAATTTTAATAATTTTACCTTTTTTTGTCCTTTTCTTTTCAAATTTTGTTTCCTTTGCCACAATACAGACGGGGTATTCGTGGTTTGGTTTGTTAAGCCGTTTTGTTTGTTATTGTTTTCAATT

>Gm12ciRNA108

GTACCATGATCTTCACAAATCACATGCATATAATGTTTACATTTTTATAATAAGCCCAAATTTAGAAGTATTTGAAATTCTGTCAATGGTTTGATTTATGGGTGTGCTTAAGGCTTGCTTTATGCCAAAAATGATTTGTTGTGGGATGCCTGAATCCAAGAAGCAAATAATTGGATGTTTGTCT

>Gm08ciRNA1080

CATAATAAAAGTATGGGTAGAAGTACGACAATGTAGTTTGAAGATGAAAAATACCTGATATATACTTATCACAATTGAAAGACAGAAGTATTACGGTCAGATCAAGAAAAAAATATCTATTCAATAAACTTCATAGACACACATGAGAATCAGAAAAAAGAAACAGAAAGTATAGTAAGAGTGAGAGAAAAGGACACGTACCGACACCAAACCTTCCGGTCCCACGACGAAATTCAAAAACACCACTTTTTCTTTCTGTGAAAAAAAAGGACAACAAAAAGAACTATTAGGAAACTGTATTCAAATTTTAATACTATGGGTATAGAAATAACTACTTCCTTTGAGAAGTAAATTTTACGTTCGAAACGTTGGAATGTTACCCAAAACAGTACGTAAACGTCACAACCTAAAATACACATCAAAGTAACAATTAGAGAACGTCTCTATAAGGAGTACCGAAACTGTACTATAACGATGAATTAAACAGAAGTCATAATCTGTACTAGCTCAGTAAGGTAA

>Gm08ciRNA1081

CATTCAAAGTCTAAGATCAACGACGTTCTACCCAGAAAGATAATAATAAAGTCAAAGATTTAAGTTCGAATATAACAAATTCTACGAAGCTTATTCTTTAAAAGAAGAAAGAGTTTTGAACAGGTACTTATGACTGATAACCTTCAGTCCTTGAAACATACTTCTTGATGTTGTCTTCAACGACACCAAAAATCAGATCCAAAAACGTTGTAAATATAAACCTACTAAGGATAAAGTGAAAGTGTGTGTAAATTGACTTAAGTGAATATATGTATATATCTACACGTGTTTATAGATGTGTGTGTGTGTGTGTGTCCGTGCAGTTTCACATTCATACAAACTCTTTAATGTTTTTTGTCTATAAGAACCATATCGTTCAGTAGATCAAATCCACGCCACATAAATAGTTCACCAATTAATACAATTGATCACTGATAAGTAACTGGTAAGAAACTCACAACAATTGATGGACTCACCAATCATCAAGTACTTGATATTTATAAAAATGATTATAAAGTAACATTAAATGGGATAAGGATTAGAAGTTTAATCTATAACAGTTTAGTAATTTATTATTACAGGTTCACATTTAGACGTATATACTCGGTTTATACCTTAACCCTAGAACCAGCCAAGGTATCACCCGAAAGACTTCATAACTAGAACTTCGACCAAACAATAGAGTTAATACAAACTGTGGTAATCGACTACGATTCAACAGACTAGAAAGTAACGTACGATAAGT

>Gm08ciRNA1082

CATTACATCGAGTAAAGGAACCTAAACCTTAAATGAAGATGAGATCATGAGGAAGATTAATTGTTTTTTTTAGTATAAAGTACCACACGTACTTCCCCCATAACCAAGCCTTAGAACGTGTTAATTTAAACCACATATTTAATTGACATTCAAACTTAATCGTTAAGCTCATTAACCGTATACGGTTAGTCTAATATAACGTAATCGGAACAAATAAAGTATGTGTTCAATACATTCCCAGGCTTTTCTTTCCTTCTCTCACTTTCAGAGAACTTTCTTTCCTAAATCCTCGTGATTATGGGCGTGATAGTAACAGTACTTTTATACGTTAGATTTCGAAATTTTAAGGAGACATACAAT

>Gm08ciRNA1083

GTAGAGCTGCAGTCTCTTTTTGTTGCAGCTTCCTTTAATTGAGCAATTGGATATCTGAGATCATTCTTTTTTAATGCACTTTTGTATTATATTATTGTTAAAAATTGTTTTCAAATCAAAGAAATGTTTGCTTGTCTCTTACTTCTTGTAATATAATAAAATACGTTTCTTTGATTTGAAAAACAATTTTAATGCACTGTTGAAATTGCTTAATGCTTTGATTAAGCAATTTCAACAGTATTTGCCAAAAGATAAGTAATTTCAACAGTCTGATTATGGAAGTATAATCTGGTAGTGTAAATGTTGAGATTGCTGCAGTTTTTGTGATTTTTCATAATTCATAGATGCAATAGACTAGATGCTGCAATACCGGTAGTGGCAATGTTTATTTTGCTCGACTCAGGAAGCAATAACAGTGAATCTAGTTATTTTACAAAGAATGCAGTGCTTGTATTCTGCATGAATAAAGTTACAGTTTTACCTTCAAGGCAAAAGTTTCTACTGCTTGATTATTTTGTTATGACTGTGTCTT

>Gm08ciRNA1084

CGTTCTATGAAGGAATTGAAACCACGGGGGGAGGGGGAGAAACCCCGTGTAATGGTGACGAATACGTCTAATGACTCAAAATCGCAAATATTAAAGAGATTAATGAGATGTTTATTCATCTTCAAGAATAACTAAAATGAATTCCAGGAATAAACCCCTCCTTTCTCAGCTTTGGTTTGAGTTGAAGGTAAACAGTCAAGATAACGTCTGATATTGACTTCATTTGTTCTTACGGTGTCCTCAACACCCGAATCCTAAATAATCCGAACCCAATAATCCCTCGAAATGGATCAGAGACGACCTTCTCGAAACGACATAAACACCAAGTCTTAAGTTCGTAAAATAAAGAGTCACTATTATAACCGAATATAAAAGTTAAGTTCGTAAAATTAAAACCGAAGGGAAAACACGAGACTAGTACCAACAAAGTTCCTTCAGTGGTAGACGGACAAAACATATGTGGTACCTTATCAGCCCCTTCATGGACGGGAACTTAGGAGGGTTTTGAACAAACTACTAATTCGAACGACAAAGAATTGTGGTGACGAAAGACTGGTTGACAGATCTTTATGTTAAAATCAACGTACAACACAGTGAAAA

>Gm08ciRNA1085

CGTTCTATGAAGGAATTGAAACCACGGGGGGAGGGGGAGAAACCCCGTGTAATGGTGACGAATACGTCTAATGACTCAAAATCGCAAATATTAAAGAGATTAATGAGATGTTTATTCATCTTCAAGAATAACTAAAATGAATTCCAGGAATAAACCCCTCCTTTCTCAGCTTTGGTTTGAGTTGAAGGTAAACAGTCAAGATAACGTCTGATATTGACTTCATTTGTTCTTACGGTGTCCTCAACACCCGAATCCTAAATAATCCGAACCCAATAATCCCTCGAAATGGATCAGAGACGACCTTCTCGAAACGACATAAACACCAAGTCTTAAGTTCGTAAAATAAAGAGTCACTATTATAACCGAATATAAAAGTTAAGTTCGTAAAATTAAAACCGAAGGGAAAACACGAGACTAGTACCAACAAAGTTCCTTCAGTGGTAGACGGACAAAACATATGTGGTACCTTATCAGCCCCTTCATGGACGGGAACTTAGGAGGGTTTTGAACAAACTACTAATTCGAACGACAAAGAATTGTGGTGACGAAAGACTGGTTGACAGATCTTTATGTTAAAATCAACGTACAACACAGTGAAA

>Gm08ciRNA1086

CATGGTAGTTATAGAAAATAAATAAGACTTTAAAGAAGAAAGTTTGAATTGTCGGTCACACCGCCTGTCACCTGATGTTTGATACCCCGCACCTGTGTATTATAAAACAGACAGATATCACACAGAAAATTCACATAACTGGGTACAAACCGTATCTAAAAGAGTACCCTTTGTAGACCAAAAAAAGTTTTCGAGTGTGTACCTCGACCTTTTTCCTTCACGAACAAAGAAGTTTAATTTAACTTTATTTGTACGTGAATGTACATCAAAACAGTCAGATAAGTATTTTATACAAATCACATCATTTGATGTGATTCCGATATGAGGAATAAAGGATAAAAAGTACCATCGCCGTCGAGAAGCGTCGTAATACATATGACAACACAGACGAAATCCGGAACATAAGGACTTTCAAATTACTTATGACAATAATGTTCTTCTTTCTATAATTTTCCTTTTTATTTTATTAAAAAAAACCAGAGTATACAATTATTAAGAGATTAACAAGAATTTAACGA

>Gm13ciRNA1087

CATTATTATGATAATAATTATGTACGATCAAAATCAATTATGATATCCAAATACAACACTCTCTCCCCAACACAGATTGCACGCGGCCCCTCCCATCTTCGCATTTGGCCCAGGTGCTCTAGACCAGGACTCACACTCTGCCAGGTGAGTTACACTGGCCTTATTTACCCTCATTTTCTTACACCGAACGATAAAGTTTAGGGTTACGTTTTAGGAAGAGTTACCAAGTCTAAATAGTCGTCGGCGTCGTAATCTTATTAACGAGATCTATAAAAAAACCGCAGAAGAGGATCAAACGATGGTCTCACATCATCACGCTGCTCACAGTACAGATAGATCCAAAATCCTAGTAGTTATACTTAAATCACGATGTCATCGACGATGACACGGACGCAAATTAAGTAATTACTATTACTTTTATAATGATAAAAAAAATAACAATAACACACACCACACTCACTTACCCACGACCACCACAACAACTTCTAAGACT

>Gm13ciRNA1088

CATTCCTGTGTATAACCGTGATACCACACCTTACTACGAAGAGTGTTTACTGTAAAGAGAACTAACTGTGATAGTGAGACTATACGTATAACAACTTAAAGCCTTTGGTCGTTTGGTCTCAAAGAGTCTTTTATCGTAGCACAGGAAATAATTAATAACATGTTACGTGTAATGATTTTACAACATTCGGATACTGTTTAAATAAAGGTTCTCTTTCAAACCCTAAAGTCTAGGAACAACCATTCTATTAACGAAAAGACAGCAACACTTTCTTCACTAAGGCACAAAAACTTAATTATTAAAGACACAATTGTAAATCACATACCAATAACGTAATCAAGTTGAGTTGGGGAGGGGGGTTCATACCTAAGTCACACCACACAACAGTTCTTTGATAAAAAACTTTTACGTGCAGTATTCATAAAAACAAAACTTTCCGGTTTCTATTAATTATAATTATCGTCGTTTAAAGACAGTGTTCTACACTTCTCTAGTCGGTTTATTCCATTGTATGTAGTTCCTGTTACAGAGAACTATTTCTTTTTCGTCGTTCTTTTTTGAGCACCACTCCGTAGTATCGTGCACTACTATTCATATAGGTTTTCTTTTGATGCTACAAAACTCCGTCAAGCTACGGATTGTTGTTACTAAACCCATTTGTTCAATGAGAGTGTACAGTAAAACGAATTCTAACCTACTATTTCTCATACTTAGTCAATGTCTTTTTTCCTTCTCGTCTAGTACCTATCCATCTCACTGTTGAATGTTTCAAGTTTGGATGTGACACCGTATAACTTTAAAACCGGTGTGACGACCATCCTTTAACTAAACTGGGAACAATAATACTTAATACTTTTAGAACCCCAAAAAAATCACCAATGAACAAAACGTAGGTTTCATTTTCCATATGTATACAAAAACACCATTATGAAATTTTCTTTGTCATAAAACATGGTGTCAGGTGTAGATACGTTTCCGAGAAAAAAACACACACACACAACAGAGACAAAGGAAAAGGTAGGTTAGGGATCTTAAAGTTGGAAAACAGAAGTTTGTTTGTTTTTGTTTTACTACCTGGATCAAAACACTGAA

>Gm13ciRNA1089

CATTGAAAGGAATAAGAATAAAAAATAAAAGTAAGTAAAAAAACTTATGAAAATACAATAAACCTCTCCTCCCCTCCCACGTCTACGTTCTAAACTAAACCGATGTCTAAAACCTTGATTTTTAGACCATAGCCACTACAGTTTTCACAATTTTAAGGACCGGGTTAACTTTAATCTACCAAGAACTGCACAGGGAGGGAGTAGTCTTTTCCGTGAAAAATGAAAAAAATCCAGGTAAAGGACAAAAAAAAAAAATTATATGGATATACTTCTTTAAACATTCTATTTCGTGGTGAAGTGGGTTGTTTTCTTTGTACGTTAACTCCTTTAACTACCTTTGTATAAAAAAAAAAGTATTAAGACTAAAGCAAAAACCACAGGTACGTGGTATGAAATACATGGTAGGAAAACGA

>Gm12ciRNA109

GTAATTTATTTTTTTTCATTTTCCATATTGACTTGAGTTCTTAGATTGTGGGCTGTTTTGTTATATGCATTTCATAACCCTTGAATTTAATTGGTTTTCTTAAGCGCACAGAAACTACAAAAGTTGTGACACTTGATTTAGTTGTTGAATTA

>Gm13ciRNA1090

CATGAAAAAGAATAGAGAAGAGATTATTCAATTAAATTAAGTTTAAATTCTCAAATCGTCTATATATACCAGTTACAATTTCAAAAATGTGGTAACAAGTTATTTTTTTTTAATAAAAATCTTATAAAAAATTTTTTAATAATATTTTTAATAGTTTAAATAGTATACAGTTGTTTACAGATGGAGTTAATTATTTTAATTCAATACCATTTGTAGAAGTATGATTTTAAATTGAAAGAGTAACTTATATAAAAAAATTGTTCTTACGTACAACTAAGATTCAAATTAATGGGAAAACTATAACTTATAAGTACTACAGATTAAATATACACACAGTGAATTTATATACGAATGTGTGTACCGGTGTGTCCTCTCACCCCACTTTCTCTCTCCACGTGTCTCTAAATACTTAATACTACGTGAATGA

>Gm13ciRNA1091

GTTGGTTCATTTTATATTGTTTAGACTTTGTAGTCGGTGGGAATGTTAACTGAAATGTTATTAGAACATAGAATCTTCAACTCTATCAGATATTAACTTTTCAGGTTTCAAATCGCGGACAAATGCAGCTGATGCGGTTTCAATTACGACCGTAACCGCGGACAGTCAAAAACTTATGTTGCGGCCCAAATCAGTTTTTTAAAACCTTATAAATGCAACAAATAATGCGATTAAGAAAAACAACGTTGACCAATATTAATTCTTTAAATTTGTTTTTGTGTCTTTTTCAATGTTTCTTTCTTAAAACTT

>Gm13ciRNA1092

GTTAGTAAATATATAAATGTTTGTGTTACAATTATCCTATATACGTATTCTTTTATGCTTTTGTTTTGTTGAAAATCCTACATTGATTCGTGATATGACCAAAGCAGTGTATATATCTGGGGCAATCCTCATCTTAAAACCCGATTTATATAATTGAGTTAAACTCTGATACACGTTATAAGATGTTTATTTATAGCTCTCTTATTTTGCGATGA

>Gm13ciRNA1093

GTAACACAATAAAAAAGTCAAAGAATTGAAAAACATTGCCAAAATTTGCAGATCATGTCGGTAGCATGAGATATATATAGGTCAAAAGAATGAACTATAATCACAAGGCCTAATGACTGTTTGCTTTAGTTTGATTTTTATTTACATGTTTAGAAATTGAAAGTCCTTCAACTAATTAAAGTTTTTGAAGAAAAAAAACAAACAAACATTCTTTGAAGTTTGAATATAATCAAAACCAAATATGTCTCCCCATGTACATGTATATTCAGAGCTTATTATTTTTTATGGTATATACGATTGCTTCTGAATTTTATATATCTCTTATTTTTAACACTATATATTAGTTTTCTTTTTAATTATTTATTGCATGTATGGTTCTTATATCAAAGTATGACGTAATATTGATATTTACAGTTGCTTTGTTTCTTACATTTATGCACGCATGAAAAGGTTGGTCCAATTGAAAAACCTTTAGCTTTCAAGAATGATGGCTGTCACATAGTTGCGAAAATTCTTACAACTGTCATCTATTATTAATTATTGTTTGCATATGCCAATCCTACTCATGCACATATAATGGTCCAAATTCTTGATGTTTATCCAGCAATTTGGTGCGTGATTA

>Gm13ciRNA1094

GTACATATTGTTACATAATCTTCTGCCATGTTTAATTCATGATATCTGAAATCATATTGAAAATATGGTCATGACTGTCCATCCTTTCAATAAGTTTCATTCATAACTAATGATGTATTTGGGATGGGGCCAATTTAAAATACTTGAAATGGGTAGTTTCAGTCAAATTGGATGCACATATCATACATAGCCATCAGTTAAGTTGGGGTTGGGGTTCAAATATAGTGTCATTCTTATTTAGGGGTGAATGTTCATGCTAATAATTAATTTCTGAAATAAGAGGAGGGGGAAGAGCAGCAAAACCTTTCTCGCCGGTCTCTGGTGGAACTTTTTATTTTATTAGAAGAGGCCAAACCCTTACATTTAGTTCTACGTTTGAACAAAGTCCCATCAAAATCTGTCCAAATAAATTGGTTACATTGTTCAGGGGTGATTTTATCTGATAGAACTTTGAAATGGCAAATTACATTTGTGCAAATCATTGCAGCATGGGTTGATTTTATCTTCACTGGCTTATTTGATTCTTTTCTTTTGATGACATGTCTGACATTCATTGGATATAAAAATATCTCCAATGTTCAATCTTCAAGTTGCTAGAGTTGGTCTCGGGTGCATTGTCATAACAATAGTTCATTGTATTGTGCCTTCAGAATTGCTTGCTTGTAACTTAATTTTTTATGCACTGCTTTGAACCTAGCAATGGATATGGTACTCCAGAGAGATAACTCACCCTTTGGCTTGAATGTCATATTTGAGAATTATGGTCCACGTGTTATCTA

>Gm13ciRNA1095

GTGTGCATATACTTTTTTATTTTTTACTTTTAAGTTACTTCATGGTTGTGATGTCTGTATCTTTGAAATTTTAAACGTAATTTTAGTTATGTAATGCAATTTTGATATTTAGTAAAAAAAAGTAGAACTTGATTACGTAGTCATGTTCATATCTTTTGTGTATTTGGCACAACATAGTTGCCTATCATTACAAAGTTTAAGTTGTAAACAACCTCATGGTGTTTTTCAGTTGTTTCCAAAGCAGTTGCTTGAAGTTTGGGTTAGAATAAAAAAATGTTAATTTTAGTGTTGTGGAAGTGAGCTTGGAAACTACCGAACCTCCATTTTTTGCAAGGCACATGTTTCTGATGTGACCTATGGTTACAGGGCTTTTAGACTATCAAAATTTAACATTTTTAATTATATACTGGACTTTTGGGGTCATTTGTTATAAAATAAACTACATGTATGAAAGTTGAAAATGGATGAAACATCAAGTTGGTTATTTGAGATTTTCCCTATCACATATGATATTATATGCTTTGCATGTTAATTATTTTATTGAGTGGCTTGAGTGAGGGAAAATGGTGGGGACTGGGGAGTGTAAATACTTAATATAATTTTCATTTTGAATAAAGTGAGTGGACTGTGGGCTGTGATATGTAATTTTTTTTAAAATATAGTAATTTCCTTTTTTTTTTTTCAAAACTAGTGTATCCCCTCACTTTGCCTCAAATCCCTTGGATTTGACTGTTGGAGTTGGAGTGGTAGGATAGGGTTTGGATGAGGTGATGAGCCCTCTAAACCATTCCACTCATTGCTCATAACTTTTCAAAATATTTTTTGTATAATAATCCATCCCACATCATTGCTCCAAATGTATATTATAACTATAACTGTTGATTATTGCTGTGTTAGTACTTTTACCTAGGTGGCTAGGCTTATTAGCTAATTACCTGAAGTCTTCAACTAGTTTGGTTA

>Gm13ciRNA1096

CAATCTAAAAAAAAGGAAGTACAAACTACAAATACTAATTACAATCGGTTAGTTCCAAGACTTTTGACCCTAAAACCGACGTTGTGTTCCAGTTCCAAAATTTTAACGCCGGTGTCAGCGGAGTCAAAATTCCACTAGGAACTATACCATCCTTTAACACCAGTTACTACGGTGTTAACGACAATGGGACTTCAGTATCTGTGGGGTTTTTGGAATTACAACGTCAGTTTTAGCGCCAGTGTCTGACAAAAATTTTGGCACTAGTTCCAAAAACCCCAGAAACGCTGACGTTAATACAACACCAACGTATCCAGCATAAACAGTGTTAAAGGACATAATTCCTAGCGCTATTAGTGATGATGTAAAACTTTGGAAATCGTAACCCAAATTCCCTCTAGAAAATACCCCAAAATATATAATATAATACAAGATACAGA

>Gm13ciRNA1097

CACGAAACTTAAACGGTAAAAAGACCCAAAAAAAAGAGAGTTAACATACTTTTTTTTCTTGTTTAACCTAGGTACCGGAAGACCCCCTATCAATCAACTAACTATAGGTAATTTTCACCCCGGGGATGTACTAGTATCTTTTTTACCGCGTGCAATCGTTTCGTGCCAAATTGCACGCGGCCACGATTCAGTTCAGTGTATTTGACTACAACCCTCAAGAAAGTACCATGTATCTTTCGTACCGGCAGACCCCAACAACTAACTATAGGTAGTTTTCACCCCAGGTAAAGGTATGTATGTACTAGTAACATGTTTCATCACGTGCAATCGTTTTGTACCGAATTGTACGCGGCCCCAATTCAGTGTATCGACTACAACGTAACGTCAAGAAGTACCATACTTTTTATTTTTTTCTTGTATATCTTTGGTACCGGAAGACCCAAAAACCCAACTATAGGTAGGCAATTACACCCCAGGTATGTACTAGTTTCTTCGCCACGTACAATCGTTTCGTGACAAATTGTACGCGGCCCCTAATCAGTGTACCGACTACAAAAGATAAAAAAGTAGCTCAAAACATAAAAATACAAACAACCAATT

>Gm13ciRNA1098

GTAGAATGCTCTTTTTTTCTCGAGATCATTGATATATGTTCCTTTTGGGGTTGTCTTGTATCCCTCTTGCATTTTGACCCAGTTCCATTTCCATTGGTGAAAAACACGAATGCCATATGGTGTGTGTGTGTTGTGCAATCATTTGAGCTGGATCTTTTTCCAACTCTGCTACTTTGTTTGGTTATGGCATTGTTGATGTGTGATATTCACTCCGCCTTTTTTTCTTGATTATGTTGCATTTCTTGCTTCCTTTTTTGGTGTTGCATTTCTCACTTTCTTGTTAACAATCCAATTTGCATAGAAAACTGCTCAGGGTGATAGGATTATTTGGATGCGCCTTTAATGTTATCAGCTATTGTGAGTGTGTTAA

>Gm13ciRNA1099

GTACTCCCCTTCATACTTTCTATGATCAGTATTGTAATTCTCTACAAGCAGTTAAATCTGCATTCCACTAATTTTCAGCAGATACAGCTAAAGGGTTTTATAATAATTGAAATAAACAAGGGAGAAAAGAAAACCCAAGTCAGAAAATTTGTACAGAGGCAAACTTAGATTTCTAGATTTAATTGTGAAATGGTATTTAACCAAATGGGGTTTGTGATTTTGTTGTTACCTTTGATTATTGGCGTTATACTTTCCAGTTCCACTGTTTAGTAGTATAACTTGGTTATGACTTA

>Gm06ciRNA11

CATAAAAAGGTTAATAAGTAGAAAAGAGAGATTAATTAATATTTCTGTCTAGTACACACAAGTCCACACAATCGCACACACAAACTTGTATAACGCGGAGCTATATACTACACACTGGTCTTCTTTGTCTTCTTTTTATGTGCTACAATTTTGATCACAAACACTCAATTACACCCGTATATACAGTTCAGTAAGTCTTGGTGATCAATTGTATAATTCCACGGTAGACGAGTAGATCAAATTACGATCTTTTCATGTGTGTCACTGTTGACTCTTTTCTTTCTTTCGTCTATCATTTTCCTGCTTTTCACAATAGTTTAAGAAAAATGATCATTAATTATACGTACTATCCTTACTTCCTCTCGATAAATGATATAAATTATCTAATATATTAGTTAGTACGTCGGACCCTATTCTTTGTATTCTAACTATTTGTTTTTACCTAAAATCTATTAACGTCAACCCAACTTAACAATGAAAAGGTTATCTCGTGCAAGGATTGTTAAACTACTTTTAACTTTAATGAGACATGCTGTTAAAGTCGGTATATGCGAATGTTGA

>Gm12ciRNA110

CAGAGAGAGACTGAGAGAAGGAAAAAGCTAAAACAATGCTGCTCCGTGTGTGGTTTCATCTCTTTAACACTGGGTTAGATTAGTTAAATCAGAAACTTTAACATTTTCCGGTTCAAAGACTTTAACAACACTAAAGTCCTTAATTTGACAGTTAACTAACCTACGAGGATTAAGGTACTGATTAATCCCTTTAATGCTCAAGGTCAACTCCAAAATCCCTGATTAAGTCGCATAATTAGTAAATAAATCAATACCACACTATCTTTACATACGAGTTGAATAGAAGAATCGTGATTAGTAAAGAAATATATATAACTCTCTTCTTCTCCAACCAAAGTTACCAATCTACTAAAATAAGGATTCCAAGTAAAATCAGTATAAGTACTTTTAAGCAAAAAAAAGGGGAACCAAAACGTACCTAACCTAACTTACACTCAAGATGTCAAAGGACAAGTTCTTTGGCACAACAACGGCCTACGAGGTTAAAAAACCAAAGACGTTAGTGTATAGTCAAAGGGTGAT

>Gm13ciRNA1100

GTAATGTGAGATGATTTCACTTAATTCTACACCGAATATACTTGATGAAGCAAATGGCAAAACCTATTAGTTTAAATGGCTGCTTGCTAAGTTGTTAAGTGTCTGTTTCCCATTTGGATGAGGTTATTTTTAGCTTTGATCAAATAAGAAAGCTACCTAAAGCAAAGCAAACATTTTAAACTGTATGGTATGGTTATGGGCACTTATTGTTTAAACATTTTGTTTTGGTAATGGAGCATCATACATAGTTTATTGTGGTTTGCACATGTGATAGATGAAAAAACATAACAGTAATGAGCACTAGAAAAAGCATTTGTTATTCTTACTTTTTTTTTTTTTGCTTGGCATTTGTTATTCTTACTTAAGTGCTTATGGCTATACCAAATACTGAATCTGTCATCTGACTCAACAGAAAGCATACATATGCCAGGAAACCAATGCAAAATTAGAGTTGGTGTAATTAAATGACTGTTTCATTTTTCTAATATATATTATATTGTTTTCTTATGCTCA

>Gm13ciRNA1101

GTATTTATACTCCCATCAACCCCTTGAAATTCTCAGTCATCATGAAATTCAATACTATGTTGTGTAAGGGTTCTTGAACTAATATTTACTTCTGTTGTATAAAGTATTTAAAGTTTTCCGTAACTGTTTGAAAAGAGAAAATATTGAGTTTTATAAAAAGCAATTCATCAGGTTGAAGTTTTTCATCTCATTTTTATTCATATATCTTCTTATTTAGGCAGTAGCTAAAGTGCATGTCATATAGATGTTCTGCAAATAGAATTACTGAGAATTATTGGTGAACTCTTCTCATGATACTTGCCAAGATGATGAGTTGCGTTTCTGATGAAGGAAATATCTCAGCTGTCACCTATTAATGCCATTATGTGTTCAAAGAAAGGCATGTACCTTGGTTGTTAGTGTTTGCCTAGTGTTCTTCCTTGCCTGAAAAATAGAACTGTGGCTTTAAAAACATGGAAACGTATTGAAACAGTCTGTACAAAATTGTAATTGTTACATCTATTTTCATTTTACATTGGAAACGAGTTAAAAAAATTATTATTAAGGAGTATCGCATTAGGAGAGTATATGGTTGATCAGTTTTCCATAAATAGAACACCATTGTATCTCAATCATCATCATCATCATCTTTTATTCAATCAATTTGATTCTTTCTGTTATTGCATCTCTTGAAACTACTTGGCATTAGCCTATGATCTATCCCTCACATCCTCTAATTACTTACATATTGAAACTGTTGTATTCA

>Gm13ciRNA1102

CATTAAAAGACGAGGGACATAACACAGTACACGTCAGAACGTAGACGATTTGATCTCTAGTGAAAATAATTTTTTAATACGAACATATTATAAAAGAACGACCAACAAACAATGAACCTTATAAGTGTTCATCATTGTTTCATTAAATGTTGATAGCAGAGGGATTTTAATAATCTATCTGTTGTAAATGTTTAAAGCGACTTTGAGACTTGAATAGAAAATTAAAGTA

>Gm13ciRNA1103

GTCTGTATATCGATTAGGTGTCTATTAATGTAGCAATTAATACCTTAACTATCTTCATTTCTTTATAAGTTTATTTTGTTGTAGTGCATTTTCCCTTAGTTTGTTGGAATTGATTGTCTTTTAAAACTCCCATTTCCAAAAAATGATAAAAATTTAGGCATGGCTTTTTTCTTTATGAAGAGAAAAGGTTTAGTACTATAAGTGTGTGGGTCTATTGAAAAGTTTAATTAGTACCGAAATCATTTTGTATATTTGAACATGTATATGTAGTATGCTGTCATGAGCACTATATAACTCTCAACTGTTCTCGGGTGTGTACTT

>Gm13ciRNA1104

GTCTGTATATCGATTAGGTGTCTATTAATGTAGCAATTAATACCTTAACTATCTTCATTTCTTTATAAGTTTATTTTGTTGTAGTGCATTTTCCCTTAGTTTGTTGGAATTGATTGTCTTTTAAAACTCCCATTTCCAAAAAATGATAAAAATTTAGGCATGGCTTTTTTCTTTATGAAGAGAAAAGGTTTAGTACTATAAGTGTGTGGGTCTATTGAAAAGTTTAATTAGTACCGAAATCATTTTGTATATTTGAACATGTATATGTAGTATGCTGTCATGAGCACTATATAACTCTCAACTGTTCTCGGGTGTGTACTTA

>Gm13ciRNA1105

GTATTATGGACTTTTGATACTGGATGTTTCTTTTATAATTATTTTTTAAGAGAAATTTTTAGGGTGAAAGATCACTGTCCTATCCAGAGACAAGGTAGCCCAGGCTCAATAAGTGCCTAGAGCCTTAGAGGAGTACTTATAGATAATATACACACAACAGACTCCAATGACTTTTAAATGCTGACCAACTTAGTTGACAGCTTGACTTGGGTGTGCCAAGTGCCAACTATTCTTGACACATTTCTTTTATAGTTTACTGTCATTTCTGCAGCTTTATGTTTCAAATGAAAAACTTGCTTA

>Gm13ciRNA1106

CATATATGTAACTAAAAAAATTATTTTCTTTTAAATAATCAATCATATTTTTTCCCAACGTTTTCCCTTGGTATTATTAACAATCTCTACATATATTTTAGTAAGTACACAGAAGTAGATTGTTGAATTCGAAAACCCCATCCACCAAGTACTATATCATAGTCTTGGAGACTGGTTCACCAGGCCACAAACTAGGAACGTCGAGGTAAGGAAATTATTTTCCAACTTAAAGTTGTGTACCATCCACCCGAACACGTAATAGATGAGAAGATCCGAAGTTCGGACTTTCAGAGTATGTACTCCCCGACACAATCTCTACATTATTTTTTAGTAAATACAAATAAGTGGATTGTAGAATTCTAAAACTATATTAATCAATGTTACTTTGTTTTTAACGACAATTTCATAACCTAATAGAACAAGATGATCCGAGTTTTTCATTCTGGACCAGTTTATACAAGAACAATAGGTTATGTCACTTGTCTCTTGTAATCATTGAACATCTTTATGATTTCATAGTCATCTAGAATACCATACTATTAGAATAT

>Gm13ciRNA1107

GTGAAGTAAAGTTTTAAAAATCAACTTTATTTAACTGAAATTCGATCTTCAGAGGTTGTTTACACTTTATACTCAAGATAAGATTTGAATGTCCTAAAATGCATGACAAATCCAACAATAAAGAAAAGTTTATTTATAGATATTTTGCATATAAAAAACTTCATTTTTCTTTAAATGCTTGTTGACGTTTATATTATTTAATTTTAGTAATTAAAATTGGCAATTTTTAAAAATTTTAAGTAGATAATAATAATATGCCTGAATGAATATCTGATTGTCAATGTATTTTATTTCTTTAAGGTATTAAGAAAAAAAATGGGAATAACTGCCTTACTAATTTCTGGCTTTTATGAATAGTTTGTATGGTAAGAAAACACCCAACATCTCGATTGCATGGATACGAATTCTAGTGGGATAAAGCTTTTGCAGTTGCTGTATGGTTAGAAATAAGATGTTACTTGTAGAGGTGGACAATCATGATGGTCTCTTGGGGAAGCTAGGATTAGCTGTTATTCCATTTGATCTATACTGCATCATTTCTACACACAGTGTGACATGATCACTTTACTTGAAATCGAACAACCACATGCATTTAAGACATACCCAATATTAAATGTTCATCTGAAAATTGA

>Gm13ciRNA1108

GTGAGTAGTATTCCCTGTTTGGCTTTTTCCTTTTTTGAATAAAAAACCCTTTTTACTCTATTTCTTGTTATTCTCACGTTGATTTGAACTTAATTTGAATGGACCTATACGCTCCTTTATGGTTGCAACATATCTAGTCTCAATTAGACTCTGATTAAATTGAAAGTTGAATCAGTGGACAATATGGAACTGTAATTATGATATTTTATTTATAAATTGTATTACATCATTTACGTGCAAAAAAGTCCTTATATACTAAAGCTATTTAACTTAAATTCAAGGTTTAAAATTGTGGTCGTGGTTGCAGTTTTGTCACGGATAGACCTTGAAAACCTAGACGTGGCAGCAGCCAAAATCGCCATCATAGACTGTTTTTAAAATCTCTCTTCACTAATGAAATTGAACCAAATCCAAACTCTCACTTGGACAATCACGTTTATTCTAGTGTGTGTTTGAAAAAAATTATTTG

>Gm13ciRNA1109

CATCCTTAGAGAAAGACAGACTCCCCGATCTAGTTCCCTCTATCTTAAATCAAATTACATAAGGACGAAAATAAACAAGTTTACTACTATTGTGATGGGCTGAGAAGATTTATATATTAATGCAAGATAAGAACAAGTTAAGGAGAGTGTTTTATTAACATTAACAAT

>Gm12ciRNA111

GTTCGTTGTTTCTGGACATTATTTCTTGAGCAGAAAATCATTTCTAGATTCAAATTCTTATGGAAGCATGCATGATATAGTTAACATGTTTCACTTTATAATTTGAAAGATCAAGTTGAATCTGTTAGAAGTATACGAAGTTCTGTGGTATAGTCTTTATTCTTGATACGCTAGAGGAGAAGGTGAGGACTTGCAGTTTGTTTGTACAATCATTCTCGATAAGCTTGCCAATTAGACACGCATTTTCTTATCACAATTAAAGATCCCGTTTTTCAAGGGCTGGTGGTGGGATCCTGGTGGACTTACAAATTATTGCATTCATGTCCTTGAACTTTTTGTTTCTCTCAAATTGTAAATTGTAATTGA

>Gm13ciRNA1110

CATGCAGAAGGAAGGGATTGGGAAGTGTGTGAGAGAGACATAGACATGCGTGGCAAAGCGAGTATAAATAAATAAGTAAGTAAGGAGTGTTAGTCTTATTGTTATTATTAATGAGCGCGTTGCACAAAGAGAAGCGCGCTAAAAAGCGGTTTGCTGCTCAGCACAATAAGTTAAACTAAAAGAAGGCAGCTCACCACCACGGTGACTGCCCCAAATAAAAGAGAAGAAAAGAAAAAAGAAAACAACAGCAAAACTAGACTCGCTCACTCGCAAAGCGCGATAAAACGCTAAAGATCACGTCAAACCCCAAAACTAAAATCCCGAAACGCCACACCTAGGCTTGATCCCAC

>Gm13ciRNA1111

GTGTTTTCTTGTTTTCTGCTATTTCCTCTGTCTCATCATACATTTGCATATTGCTTGTCCATAATCTGTCTGATCTTTGCAAGAGATGACTCGCTTATAGGAGTTTGTAGCTTTTAAATAAGATGGTTATCTTTGGTTTTGTAATAATATGAATTCTCTGTAGGAATGGTTAATGATTAATTTCCGATCCTTTTTTAACATTGCTTTGATGTATCATGGCTTCCAAGACTCGAGTTAGATTTCTGTTGTCCCTGTCTTCATTAAGATGCATCACATTCTGTAGCTAGGGATAGGGGCTCACATTTCTCCATAACCTATTTGTAGGGTATTTGTTATTTCTTGCCTGAATTGGTATTGGTTTTGTAGCCTTTGAAATGCCTCTGATTGACATTCCTTTCTTGAAGTCTTGGACTATCAATAGTCAAATATGGCCTTTGTATTTGGGGATTTGTGGACATCTATTTATCTCTTATTATTACCATTTATGCATGATACAATCAGCGTGTCCTGTGGAATGGTCATTAAGAGTGCTTGTATTCGTGATTTCTTATAAATTCTCATGGATGAGTAAATTA

>Gm13ciRNA1112

GTATGAATTGTATTTTATTTCTCAATTAACCCTAAATCGTTCTGAAATTTAATTGAAATGTATTCTGTTAGAACTCAGAAGGAAGCCAATTTGTGAAATCGTTGGTTTGGAATAAGTTTTTCTTTCTTTTTTTCTTTTTATGAGCTTTTGTCTAGGTTCGATGTTTCGAATGCATTTAATTGATCAACTTTTATTGGATACTTATGTAAAAACGGATCTTAATGTGACGTCGTAGTTTCTAAATTTTCCTTCTTATTTGGAGAGCAAAAAAACAATCTCAACCGACAGCGATGCAAAATGAGGTGTTTTGCCTTTGGATGAGTCCTAACTGTTCAGATTTTTCTCCTTGAAATGCTACGGGTTGTTCTCCTAAAGAAATGTTTTTGTCTGGTGGATTGCTCTATAACACGACCTGTAACATATGTGTTCCTAAATGGACTTGCATGTTTTCTATACGAGTTTTCCT

>Gm13ciRNA1113

GTACACTCTCTTATCTCTATTCATCTTTGTTTTTTTGTTAGCAAAGCAAAACTATTGTTTCTCGGGAAATTTAAGATAATTCAATTGGATAACCTGAACGAAAATCTCAATTTTGTGCTGTTGTCGGAGTGACTCAGAAAAAAATGCAATGCACGATTCTAATAGCATTATTTATGATACATTTTCTGTTGTTGAAGGTAGCGTAGGTTA

>Gm13ciRNA1114

CGTTCTCGAAACTAAAATATTAAGATAATAAAGTGGAGTTCTAAAGCCAGAAGACTTAAGACGAAAAAAAAAAGAGAAAATTTACAAACTCTATCGGTCAACCAGTATAAACTAGGTAGATCGGTGGGGTGGACAACCCAATTAAAAATAAAAAAATCAAAAAAATCAAAACGATCTTAATGAAAAGTAAAACTATCAGACGGAAATGAAACCTTTTTCGAGCGGTTTTTACTCTGACAAAGATTATCCAAATCTGAGTGATGTGAAAATGAACGGATGAAATCTCTGTTCAAGTGAACTCTTCTTGTAATAGAGGAGGAAACGAATACTACCTATACGACTGGGAATAGCACACGGAGTATTATAAAGTTAAATCGAATGAACAGGAAAACGGATTCGA

>Gm13ciRNA1115

CATAGCATAGCTAGAGAGTAGAGTAGAGTAGAAGACGTAGAAGTAAGGGTTAAAGCCTTTAAGGCGAAAAGTAAGAATGTAGAAAAAAGACGGGGGAGAAAGGTATATATATATATTTATATATATTAGTTGAAGTTGTGAAACGACTTACAAAACGCTAAAGCCTTAGAAGTAACTGTAAATCTACGTTTTATGAACAACAAAGTTAGCCAACTCAAGCAATGATAAAAAGAAATTCCTATACTCTTACAGTTAAAAAAAAAATAAATTACGAATAACTTCCACGTGTTTGGAACAAAAATCAAATTAAATATATTTTTAAGTTATTATTAAATAGAATAGCAGCACAATATAGTTACTTATACTATCACTATTTTTATTTTTACCAATTTTAAATGACCTACAGACACATTTTTATAATCATATATATTTGATTTGAGATTTAAGACGATAGAGTAAAAAAAAATAAGTATTGTGGTAAATATACTTTAAGTCAAGTGAGACTTTTAAAAGGTCAAACCCTTTACTGA

>Gm13ciRNA1116

CATACAAAGACCAAATTGTGGAAAACTAGTAGTATTCCAAAGTATTGGAGTAATCCGAGTCAAATAACTATTATATGCGCAAGGCTAAATCAATACAATACAACTATACACTACAGACCAACTTAGGTTTAAGCAAGTCAACAGAAAGTCTAAGGTACTAGACTAACAAATACGATATACAAAAGGCTTAAATCAATGCTAAGGACTAAATTAGAAAGAGGAGTAATTTCATTCCGAAACACCAAATTAAAAAAATTAACTCCATAATCCCGAACATCAAATTAAAGAAGAAGAAGAAGAAGAAGGTATAAACTCCATAACCCCAAACAAAAAAATAAGATATCAGCTATATGTATACATGGACCAAAAC

>Gm13ciRNA1117

CAAGCAGAGACAAAGTAGAGAAAAGTAGTAGCGTCGGATTAATCGGAATCAAATAACTAAATCGAAACCAGAAAGTGGTACCAATGTAAAAATGCAAAGATAGAAAGCTAAACACTAAAAAATGCAATTATACACATATGGCCAAAACAGCAACAACTTAGGCTTTGAAAGGTAAGCTGAAAGACTAAGGCATAGAGCAAACAGTGTACGTTTTAGAAAGGGCTAAAAATTAGACCGATAGACTAAACTAAACTAAAACTCCTTAATCCCAAGCACCAAACTAAACTAAGACACTAAGCGAACAATATACATTTTATAAGGGCCAAGGATTAAAATCCCAGACGCTAAATAAAAGAAAATAGTGAAAACTCCTTAGTCCCTAGCACCAGACTGAATAACACGTAATAAATCAACATACGACGTTTTAGAAAGGAATAAAAAATTAATCCCAAATCCCAAACGCTAAACAGTAAAAACCCTTAATACCAAACACCAAATAACTAATACGTTAAGCCCAAACGAAAGAAAGGCCAAAAATTAGTCTCAAGGA

>Gm13ciRNA1118

GTAAGTTTTGGTCATATAATCCCATTGTGTATTCCATTTTTTACGCTTTATTGCAGTTATGGAAATACCTATTACCATTGTTGGAAAACTAAAACCATTAGGCTCTTGAGAATTTGCTTTTGCATTTGTGTGATAGATTTTCTGCTGCTATTAGTTTGTTTCCTTACACCTTCCTAATCTAAATATAATCATTCAATGCATTCATTGATCTTCCCAACATATCTCAGTAATTAATCATAGTTTATTTTGTTGTAAACTTGTGATATTATTAGCCTAGTTAGTAACTTTCTTCGATTTTGGAAAGCTTTTTCCTTCCATATGTTGAAGCCTTTTAACTCTTCAACTAAGCTTCTTGTGCCCAAACTACTTACATCTTATCCCTAGGTTAACCTTATCTGGTTGCTTATTTTTTCACTTTGGAGGTGTTGGGCATACTTTTTATTATATTTTTTTTAAGTTACCCAGATAAGACTATATACATTATTGGCCTATTGCTTCAGTACTG

>Gm13ciRNA1119

CATACATGATATAGAGACTATAAGTCACTATACGAAGGTTGGTCGTTATCTTTTTTCTTACACGTGATCACTTGTGATATATGAAAAAGAGACAGTCCTATCATTTTTCTATTGAAGAACGTCAAGTAGATAACAAGTCAAAATGTGGGAACAATCTTTTTTACCAGTATCATGAAAAACTATTACTCTCGTAGTCGTACAATTAGAGGACCCTTCTATTCTCGCTAATCCTGTACAACCTACGTAATCAAAGACTGTAAAAACCCGATCACATGTAGGTATTAAGACACGATAGAGCTAATTTGGTCAGTTTTGAGTTTTCATTATCGTCTCCTTCAAACCGTTTCTTACGGACTGGACTACCATGGCAAAACTAGTTAAACCTCGTCTTTCGTTACGTCTCCGTAAGAAAACGTCTTTATATACTTTGTTATTAACTTGAACCGTTAAGTACGATCTGACAGTCTATATACTAACATGACATAAGAAACAGTATATGCCAACTCCTTCAGATAAAGGTAACTTTCTTCTCTTCGATTTACAAAATGTACACGAATCCAGTCCAGGACAGTAAACTAAAGTTAAAACTGTCCGCAGTACGAACAATGGTTTAATCTCGTCATATATAAACTTTAGAATTTTGAACTCATAGTTTTACATTTGAAACTGATTATTGTAGAAAATACCTCCTATCCTCTGTAGCTTCAAAGTTCAACCACTTGTATCTAAAGATTCGAGAAGTTCTGACCTAGGAAAAGGTCCGAGATAGAAAGGTAAGATAACTGTGGATAGGAATTTTATTTAGTAGGAAAATACAAAAAAAAAAGTAGGTTAAGATTAATAAGTTCCAGATAAATAAATCACAGTCGTAAAAAGTTGTTTACCAATAAAGACTGAACATTGTGA

>Gm12ciRNA112

CATGAAGATGAAGAGAATAGTTGGTAACCTGTTTTTTTAGTTTTATGTAAAGAAATTCATAACAAATACAATAAAAAATTAGTCTTAATCTCAAAGTACAGTGATTAAGTGTGCTAATTTTATAATATATTTAATGACTGTATTTTGATGCTGCGACTAACTTCATGTTGTGTGTCGTATATTTTTTGACATAGGTTCAAAAAAAGGAGTTAATAATATCAGAGAATATACGTAAAAACAAAACAGTACAATACCAGTATCTGAT

>Gm13ciRNA1120

GTCATGTTTTTCTTATTGTCGGTATTACCAAACAACGATTTAATACGTGTCTTTTGTGTTTTTTTTCTTCTTTTGTTTTTTCCCGACAATTTGGCAACTTTAAGGAAGCTAACTTAGTTGTAATCATCGAAACATGCCGTTGCCAGTGAAATTGACCCTTTTGTTTTTTCTTTCCATCATTGCAGAAAAAATTAATTTTTGTTTTGTTTGA

>Gm13ciRNA1121

CATGTGTGTATGTATGGGATGGGGAAAGCGATAAACTAAATTGGTAACAATACACAACGAAGATATCAAGATAATATCACTCAAGGGGCAAAATTCCAACCCCGTAAGTGAGAACCTTCGTATATCAACACTTTAACGGTCAAAATTTGTCGTAGTACAAATAAAGAGACGTAACACAATGGACAGACGACCATTTAGGGAAATAAGATATAAAATTTGGGATTTTAATAGTAAAAAAATCAAAGACTTCACGATTCATCAAAAAATAAGAACCTAAATGACCTGAAAGATATAGATTAAACACAGGGAACTTGAATCCCAAATGAGGAAGTAACTGAATAAAATAATCGAAATCAAAATCCCAAAATAACGTGTTAAAAACGACATCAAAAAAGAACGAAAACAAAGAGGACACGAAT

>Gm13ciRNA1122

CATACTGCGACCAGCCACGGTAAATTAAAAACACGAGGAGCACAAAAACCAAACGTACAAAACACAAACTACATTACGACGATTTCCAAAGGTTCACTTTCCTTACCTTTTATAAAATAGAAGAATACGACGAACTCGTTACTTTGAACGTACCAAGAAAATGGGAACTTAAAATGGGTAGTCTTAACCCTCGATAAAGTATAATCGAACAAGCACCCCTTTAACTTTAAAAGTGGTACCTCGGTACTATGGATACCGGTAACTGGAAACAACCTTAAAGGATTAACTAAGAACTTACT

>Gm13ciRNA1123

CATTCCCGGTTCACGGAACTAGAAGGACAGTGACGTAATTGAGAAAGAGTAAGTCAGTAGGTTTATGTACATACTACAAATACCATTATAATACCACACAACTAACTTCTAAAAGTACCATGATTAACATTGAGAAATTATAAAAGAAAGAACAAAAGTAATACAACGAAACGAAAACCCAAACGGAAATCCTTGAAGTAAAGCCATCGTGTACTAAGAAATTTTCTATCCAATTTAAGGGATATCCACTGTAAGTAAATCCTTAGTAAGAGATATACTTTTTTTGAGTTCAAAATCAGTGAGATACAGGAATAATCATGTGTAACCAGGAACAATTATTACCGTATATCCCTGATTTTGAATGCTTTGTGTCATTGTTCCTAGTTACACATGATTATTCCCACATATCCCTGATTTCGAATTAAAAAAAAAGTATGTTGCTAACTAAAGATTTACTCAGTAGACATCCCTGGTTTACTAATAAAATTGGGATTTTCTATAAATCTGAGACTTACATAATAAAGGTTACACGACGAACAATAGAATCAACTGAATCAATACTTAGCTGTTACGATTTTACTGCTCAAAGTAAAGACTCAGGTCATGACACTACACTCATATTATAAAGTTACATGTGAAAGGATACGAATACACTTTAAGGACAAATATATACAAAGCAGGTACGAACCTATAAAAGTTGGAACGTAGATATGAACAAGTTAAAGAAAACCAACTGTACGAAACGTGACGTAACAATGACAGTATGTCGACGTAATCTCCCAAAAAAAAATTTTTTTTTAATAGAACAAATTAATGAACAAAGAATAACGCTAAGTACGGATGACTAGAGTAAAATAATTATACACATTGAAGAGGGGATTCACACCCACCAGTCGACAAGTATATACGTAAGTGCATAGATAACGTCTAAGTACATATACGTACATGGAAATAAACCCCTACTAAATAACATAAATTAAGGAAATGGATCTGTTACAGTTTCTCTACCAGTTTCAACTGAGGGATTCACACCCACCGGTCGACAAGTATATACGTAAGTACACAGATAATGTGTACGTACACACGCGTACATGACAATAAAACCCTACTAAATAACATAAATTAAGAAAAGGGACCTTCTCTACTTTCAACTGAGTCATCTCTATAACTGAAC

>Gm13ciRNA1124

GTGAGTCCACTTCCTTCTTTCCCTTTTAACATTGTTACCTGTTTTACTTTTTACATTGAATATTTATATGACTATGTTTGAGGTTCTTACAGCCAATTTTTTATTTTTTTTATTTAAGTATCCCTATTTCTGTGTCTTTTTCTATATGAGCAAATCATTTGGCTAGCATTTCTTATACGTTTTGGTGTCTGGGGAGAACCCAATTCATGCTGTTCAGCACAAGTCCTGAAGTGGAGTATTTCAACGGAATCTCCTCTGGTTCAAACTTCGCTGATGTATCTGCAAGAGACTCCGATGCTCAAGTTTGTGTATGAATGATACAAATAGAGAATAGAATTTAGAGTACCTCATATACCTTATTGGGTCTCTATTTATACCATTCTTGATTTGGTTATCTGGGCATTCTGACATGAGGTAACATGGGAAATGCCTCCTTGGACAAATCTTACTGGAATTGCTACCTCTTACAGATTCTTGGCCATGATTTCATGCTTTGCATCAGTATCGATGTTCATCTACCACTATAAGGTTCTAGAAATATGCCTTTTGAGGCATAGTTATTGGATTTTGGTTATTGAGCCAGCGCATTGGCTTAATTTGCTAGACTGAGCCAAAATTTTCCAAAGAGGGATCCAGAATAGATGCTAAGACCTGTAGGTTGAAGGAGTAGAGGATGGTCAAGTTGTTAGAAAAACTGTACAATTACAAGTAGATGGAATGTTACAAGGTTGAAAACTTGGAATGAAAACTAATTTGATACTGGGATTATGTGGCAGCACAGAATTAGGAGAAGGTAGAAATAAAATGTAGTCATTTTCAGGCTATGGTGTAATTTTGGTTGAAACTGCCTTTCAGTCTGCTTGCGCTCTATTTTCAATCTTCCCAACTTTACAATTTAAATTAAGTGAAGAACTCTGCAATCCAGTTGGTG

>Gm13ciRNA1125

GTTTTAGTTCATTGTTATGATTAATTGAATTTGTGAGGTGTTTTATTTGTGAATTCTCATCAATCATGTAGCTGGAGCTTTTCGGATGTAACATAGAGTAATTGTTGGGAGCTAAGATAATTTATTTAATCGAATTGAAGAGTTCTGTTATAAATTTTGAACAATGAGAAAGAGTAGAGAAATAAATCCAACTTCACCATTGTAACTTCATAGAATGTGCAGTTTCAAAATTAATG

>Gm13ciRNA1126

GTAAGTTGCATGATAATCTTGACTGGACAAAAATTTTATACCATGAGTAATAAATTAGTTTGAGGTGAGATTTTGCACAACAGTAAGGTTTTGCTATCTTGTGATTTGGTGGTCATGGGTTGTAGTCATAGAAACCTCTTCTAAAGGTTTTCTACAACCAACCTTCTCCAAACCCTGAACTAGACTGTTGTTA

>Gm13ciRNA1127

CATTATAATAACTTGGGTCTTACGTTTCAAATGACGCTATCCAGGGAAAAATGATAATAAAGTCACAAGGAGCCATAGAAAAGTTTGCTAAATATAACACGGAAAAGTAAACCCAATACTCAAACATAAATATATAAAAATTACAAGTACTTGGTAAATAAAATCAGTTATGACACTAAAGGACGTCAACTTTAATTAAAACAACTTAACTATACGAGATTTGGTTTCACACCGAGATCCGTATTTTTCCTATAAGTTGGAAGATAAAACGTACT

>Gm13ciRNA1128

CATACAAAAAAAGAAGAGAGAAAGACGAAGTTAAAAAGACCCAAGCGAATCACAAACTAAACTAAAGGGATAAAGGAAAGAAGGAAAACGGACTGATCTTTTAAACACCTTGTCTCATCCTTTTCCCGTATTGAAACAAGGCGTTCTAAGACACAAAAGATAGTTGGACCGTTGTTTTTTAACAACTTTAAAGCTACACTTGGCGAAGAAAAGAGGAATCCAAAAGTACAGAGGCTACGTTGTCTCTAACCTTCAAATTGTGAAAAAGGAAGTTTAACAAACAACTGGCTTTTCAAGATTGTTTTTTTGTTTTAATTAAAATAGCAAATCCAAACATTAAATATATAAAGTGTAACCAAACCAGAATTGTTGTGCACTTTTTCCCTAATGAACGAACTCACTTAACGACCTCTACGAACTAACAAAACTACCCTACCCTGACACCGAACAAAATACTACTTTGGTACAGACATATCTAAAAGTTAAAAACCTTGTTCCTCCTCAAAAGTCAAGTGACGTAACAAAATA

>Gm13ciRNA1129

CATTCCTTAAAATAGAAATTCGAGAGAACAATAATCAACAGTTTTCGACTTCATTGGATGTACCTTCTTTAACCGAAAACAACGAGACTGTCATGTCTTATAAAAGGACTCTAAACAACACCAAAAGACACGTACATATACATACTGACCCACCTTTTACTAATGTATTTTACTGACTAAAACCTTCGTATTAAAACTTTTGGTAGATAAATAACTTATCTTTAGTACTTACATAAGGCAACTAAAAGAAATACAGT

>Gm12ciRNA113

CATTCAAAGTCGGTGTGATGGTCTTTGCGTACGTATATATCACCTACGGTGTTAAACGAAAAAAGTACTCCGAGAATTCAATAAGGAAAAGGTGAATTGGTGAACAAGAAGACTCCCACCCTGGCCGTATTTTATTCTCGGTTGAAACAAGACATCAAGATTAAATAGAAGTTGTGAACAAGT

>Gm13ciRNA1130

GTGAATCTGTTAAGCTTCTTAAACTCTAAATTTTTGCCTAACTCAACTCTAAAACATGCGCTAGCTTGGGAATTCCGATGCTCATTCTTTGAATTCCAAGCTACAACGACGATTTTGATAGTTTTGTTTTGAGCTCTCCCAATTGGTATCCAGAAGTCCACTAACTTGTCAATTCACCTATATACACTGTTTTGACTTTTCATCAATACGCGGGATATATCTAACAACATCCTACTAGTGCTGCCACTCAGTATGTCAAATGTGGAATAAATTACTAATTTAGACCCTCACTTCTAAATAATCTATCCAGTATTAAGTGTCCATCAGTTTAGTCCTTAAACTGATATATTTTACTACGGTTTAGAACCAATTTGAAGAATTTTTCAATGGAGACTACTTTGCATTATTTTTATTACTTGGAAGATTGCTTACTGTAAATATAATTAAGGAAAGAGGATAATACTTTAGGGATTAAATTAAGTGTTTATTCTAGAAAGTAGAAATGTGGATAAGAAATATGCAATCTGTATGAATTTATATATATACCACTGAAATTTTGGGGTATTGA

>Gm13ciRNA1131

CATACACATAATACGTAATGTAGTGACGATTTATACCAAATAAACGAAGTACTAGACACAAGTAGAATAGAAAAAATATACCTGTACTTTAAAAAGTCACATAATAAGTGAACACCTGACCCTTACACTCTATACTTAGAAAATACTTAAATAATAGACAAACGGATACAAGAAACAACGTTCCAACTACTTTGTAATGTTAATGTTTAGTGTAAGTACTTAATAATACAAAGGGAGTGAAGACCTCTAACACCGTACTGGCACTGGAATACCGGTTTATAGACCTATCGTTACAATTGTTTTCAGGTGGTTCTTACAAAATTACTAATAGTTTTAATAATGTTTTTAGCTCTAATAAAATAGATTAAGACTTCGATGTCATAAACAAACGTGATATAAACGTGCTGTTAAAGACGTCAAGAGTACATTAGGAAGTCAGCAGAAATCAAGAGTTGAAGAGTACATTTAGTGGTGACTCGTACACACCAAAAATAAAACTAAGGTATAACCAACTAA

>Gm13ciRNA1132

CACTCGGGTGGAAAAACAGGCGCTAACAAACAATACAAAATCAAGAAGTAAAAGCAATAAATTAAATCTTCGAACAAGCCAATAACAGATAAACACCTGCAATAAGTCTCGAACCCCCCCACCCCCAATAAAACATCAGGGACTCCCAACAAAGGAATCAACCTAGACTCGAATAATTATATTGTAGACGCAATAGCTCAATTTAAATTCTAAATATAAGTAATGAATGCGAACTAATTAATAACAAGAGAGAAAAAACATTTTTTTATCTGTTCTACAAATATTTCTCTTCACACAAACTACAGTCTAATCAACCTTTCCCCTAGAAT

>Gm13ciRNA1133

CGTTCAAGATATAACAAAAAACGATAAGAGGGATAATTGATTAAACCTTACAAGTAGATATGAAAATCGTTTCGTACTCAAACAAACGAACAAAAAAGAAAAAACCCATTATATTGTTACGGGGTTTTCCCTCGATCTATCCCTTCCCTTTCAACTGTTCACCCCC

>Gm13ciRNA1134

CACGAAGAGTCACCGTAAAGAGGAAAACACACACACACACACACACACACAAAAATCCCCCACGTACACTATATGTACCTTCACCGGTTTGGTTTTGATTCCATCTTGGTGATAAATCAACGTAACTATTTTGACAACTGTCAAAGAACTGGTAATCTAGTCTATAGACTGGTTTTTGGAACACTATCTTAAGTCACATATAGCACCCCAAGTACAATGGATAATGTCTCTCAAAGAAGTAGTTACATATTATTGACTTGATTACCGAAGTATAATAAGAGATACAATGTTACACCATCAAGTAGACCAATTTTATTTCACAAGAATTAAAAACAACAACAATTCTTTTGTACTAGGA

>Gm13ciRNA1135

CATGAAACTATATAGAAATACAACTCACTCTTTTAACAACAGTCTATAACAACACGAATTCCCACACAAACCTTTAGACAAACCTCGGAACATTGACCTTCAATGATGAAAGGTTACGTCGATTACATCTTCCTTACCCCGACCAGTTAATCGTAAGACAACTTGAGTAGATTTATCAAATTTTCCTAGAAGTCAAGTAGTTACAATTTCGTTTTATTGAATATTAAAACCCATAACTTGATCAGACTATTAACTGTTCACAAATTAACGTGCAACATATAGTAGTACATTATCTTCATATAGTGGTTAGAAGATCATAATATCATTTGCCTTACTTAAACTTAAACTCTTGAAACACAGAGTTTGAAGAACTAGAACATTGGAAAATTCAACCACCATGTACGTTAAACTAGAAAGACGTTAAGAAAGGGAACATCACATTTATTATGACCATTTAGATAGTAGGTATATTTTGTCTCTTTCTTTTACGTACGGAATTACGGTAAGATTGTGTTGAGAATAGATCAAGGATAAGTCACCCGTATTCTACACGTAACTCTATATGAAAAAGAAACTTTTGTAACCAAAGTAAAACCGACTCTAGGACAACTACAATAACACGGGACTTATTAAAAGACATAGACTTCAATACCCATTACAAAAATTAATCACGAAGGTTATTTCAACTGGTGGACGTGGAACCACCATTATTTTGAATTTTTGTAAACATTGACACTCTAATTAACACCTTCAGGTTATTAAAACATTTTGACAACGTACGGTTTGACTACCACACCTCAATCCGATTACGAAACTTACAACATGTATTGTGAGTTTGAAAAACGGTCACTTACGAGTTAAAAAACAATCGTTAGTGTATATACGAAATGTCTAAGAACTGTAAATATTAATCACGACGTAAACCAACAGAGGAAT

>Gm13ciRNA1136

GTGCTTTTTAATGAATTTTGGCTCCCTAATCAGTATTCAATTAAATGAGATTTTGGAATTCGTTAAAATGTTCACTCCCTCAAGTCATCATGAAACACACTAATCAAAATGCATTTATCTGTCCAAACCCTTGTGTTGAGTGATTATCCTCTTAATATATATGATTTCCATCCTGCTTATTACTTATCCAAAATCTAGAACATGTCTCAAATATTGGCCATGCTACGGAATTGGTGTTGCATTTTTTTCCTATCAGAGCTCAACTCAGAATTATTTTTTACATCATCAAATGATCAGAATGTAATGTCAGGGTGGGAAGAAGCATGATTTGTTTGTGACCTTAATAATGATCTCTTTTCTTCATTCATCTAGGAACAGATAATAGTTCACTGTAGTTGTGTATTTGTGTGGATAAACTGGACACATTATTCATGCATAGTGTGCATGCTTCATCATTATCCAAGAATGATCTGTGTATCA

>Gm13ciRNA1137

GTATTGTAAAGGACATTTATTTCCAGTTTGTATTATTGGCTGAAGATGTTCTTTTCCCTCTCTTATATTGTGTTCTTTTAAAAGCTAATGTTGGTATTTTTGCTAAAAAATAATTTAGACAACCAAATCTGTTCATTTATATTTTATACTGTTATTTACTATTTAGTGTTTAAAATCATTGGTGATGAGTTTATGAGGTGCCATCTTGATTTTGAAATACAAATAAAACTAATGAGTTCTCAGAACAGTGGTGCTCAATTTGATGAAATCTGGTTTGTTTATATACTAGGGGGCTAATATATTCTGAATTGTATATCAGTGGTGTTTGTTTATGTTAATTTCCACCCCAAATTAATCAGGATTATGCTTTTTTCACTTGTAAACTGTGATTTCTGTGGTATTCTTTCTCCACAAAAAGTTATAGTTGATCAGGAACTAACTTCAATAGGCTTTGCAATGTTCCGTGATCCTGCTGACAGTATTTATTCTTTTATGGATACTCTCAATTGGAACATCTTCTGAGTATTCTT

>Gm13ciRNA1138

GTAATTTTAAATGACAAAACATAGATGCACTGCTTCAAATGTTTTCGGTACTGTTTTTAATTCAAGTTGAAATTTCACCTTAATAACTTAATGCAAACTTCCATTATGTCAATTATCACGAGAAAGTCCAATTCTAATCGGAGAACAACACTAAAAACTCTTAAGAAATTTTATCTACATTATATACACTATTTTAAATTCCTTTGTTCTCAGCATGATGACCTGTGAATGTGATATCATCATA

>Gm13ciRNA1139

CATACCAAACAGAAATGTTTCCTTAGAGTACACTGAAGATATTATAAAATAAACATTACACCAACGCGGATAAAAAGGTTGAGTTTAGTTCACCCTCGTAAACTATTATCATAACAAGTACTGAAGTACATTCATATAAAAGAACCATTCGATAAAAACTTCAGGTGATAA

>Gm12ciRNA114

CACCCGTGTTTTGTGTAATGTGTCTTGAAAACAACTGGTAAAATTTACGTATACGTGTAATGGTGGAAGAGGTCGAGACTAAAGAAAGTATCTCGTTGTGTTCAAAACAAAACAACGTAAGTGACCAAATTGTGAAAATAGTCAATCTAACAACGTAAATGACCAAAGTGAGAAAGTTGGTAGAGTGGGTGTCAAAAATAATACGGTACGTTTACATTTTACAAGGGTAAATTTTCACATGTATAGTCATAAAAAGAGGAGTTTATTTCTAACGTGAATTCAACATCATGGTTTTTGTACGACATCTTCCTAAGCACTATGTACAACGTTTTGAAACAGATTGAAGACGGGTCTAATAATAAATCCTTAAAGTTTACCTTTGTAACAAAGAAAGAAAGAAAGAATGTTCTTTAACTTTGCAACAAAGAAAAAATATACTTTTCATGCTTTAAAGCTTGGTTCATTGATAGAAAACATTAATAATGATGGAAAGATCGAAACTTGTGCAACAAAGT

>Gm13ciRNA1140

GTGAGTGTTCCTATGCTGCCTTCAGCTTTTATTCAACTTGTCGGTTCAAAAAATGTCAAATATACTTGTTATAACACTTGCATATTAATGTACAAGTTTTTTCACGTGATCTATAATAACATAGAAGATTAATCTAATTCATGTGTTCCCAACACTTCATGCTAATTCCTTTATTATATGGTTCTTTCATGCCTAACTGTTAGTTGACTAGATTAAAAGATGAAGGTAGCCTGCTACCTGTCAATTTATATACCGTGAGCCTTGCCGTCATACTGAACTATTTAATAGAAATTACTCTCTTTATATTTTAATGCATTAGCAGTTAACAAAGTTTTCACCTGTAGTTCTTAACCAGATACATTCTGCCACAAGTATGATACATTAAGAATGATCTAAAATTTTCTTA

>Gm13ciRNA1141

GTAATTCGAGAAAACATTCTCTAACAGTCAAGTATTTGATCAATTGTCAATAAATATTGATTTTCCAAGTTGAAACTGGTCACGATGTCACCTGAATGCAATACCGAATGTTTTGGCTCATGAATCCTGACTGGTCCATATCATCTTCAGTGGATGATTATAGCCGGTTTTATTTGGATTTTGCTGTCAGTTTTGTATAACTTATTATGACTCAAATGGCCCACATCCTTTATTTATTGGAAGAGAATTCAATTTTTTTGCCTTTTGATGCTGACTATGTATGGGATAATCTTATTATCAATGGGAACTGAGATTAGACATGGTATTATAAAATCTTATTTCAAGCTAGAATATGATTGTGCTGTCATTTTAATTTAGATAATTTCCTCAAATTCTATCTCAAGACTTA

>Gm13ciRNA1142

CATGTAAGAAGAAACAACGTGAACATACGGTTATCAAACGACGTAATCACTTATCCACTCCTGCTCAGAAAATCAGGTCGGAACTAAAGGATTATAATAAATGTTGACTATTAATTGTAGTACGAGGTTTGTTATCTTTACGGGGGGAAGGATTACCACACAAACCTTGCCACCTCTCTTTCACTCCCTCCTCATTTTCCAAACTTTAAACTTAACTTTCTTTTAACCTTCCTTTCTTTTAAAACTTAAAAAAAAGGATCATCAGTTCAAAAGAGGAGTAAAAGGAAGGTAAAAGAGGTTTGTTTGTTTTCTTTTTGTAACAATAAACACAAAAGAAAGGAGTTGGTTTGTATGATATTCTAAAAAATCCCACCTTTTGCTACTTTAAAACTCAACAATTTTGACATCCTTGACTAGATATAATCTTAAAGTAGACGAACACTGTACATTCGTCTAAACTGTAGACACCATGACCAACTCACCAACAACGAAAACAGACTTGAGTACCGCTAAATCGAAAGTAATGACGACAACGTTCTTAGTAAACGAACGGAACTGGTTGAGTAAAACCAAACTGACTTCAATGAGTACAAACAGGAAAGGAGGATTTTTTATTTCTTGGTTCTTGTTCGAGAACAATTGTTTATAAAACATATAATAAATGTTATTTATTGGGATAATTTTATAGATTGAT

>Gm13ciRNA1143

CATGCACAAACACAGAGGATAAAACGAAAACTAGAGCCGAGACACACCAAATGATACTTGCCAATGTTAAGTCAAGCAACTTAAAAAGAACACAACACACAATTAACTTGAGTTAAACACACTCTAATACCCCAGACGTAACCTGCGACACTAGCTTACAAAGGACTATACCCCCATAAAAT

>Gm13ciRNA1144

CATGCACAAACACAGAGGATAAAACGAAAACTAGAGCCGAGACACACCAAATGATACTTGCCAATGTTAAGTCAAGCAACTTAAAAAGAACACAACACACAATTAACTTGAGTTAAACACACTCTAATACCCCAGACGTAACCTGCGACACTAGCTTACAAAGGACTATACCCCCATAAAATA

>Gm13ciRNA1145

GTGTGTCTTTTTAGGTCTTCTAGTTGCAGCCTTTGGTCACTCATCACATGTTGTCAGAGATAGCATGGCTAAAAATAAAATGATTTGAGTGCAAATATGAACATAAATCGATATATAAATAGTTTCCCAGTGTTTCATATTATACTAAGTTTAATATCATCAATAACAATGATATTGTTTGGCTCATGTTAACTTTGATGTGTGG

>Gm13ciRNA1146

CAAACTTAAACGAGATGGAAAGATTCTATTACCGGTCAAAACGTGGTACCGTACTGTTGGAGACATAAAAGTCAAATAGTAGTAAACAAATACAACGTCTTATCTGGACCAATTAAAATCGTTAAATTGGAAAGAGGGTCTTTTTTACTTATTATTTCTTTGTAGTCCGGTGCCCACCCCATAGATTATCACTCGTTTTACTCTACTTATATTACATTGTCTTACTTCAATTATAAGGCAACATACCTATAAAAACGATACCTCGTCCCTCCTTTTTCAAGGTAAAGATAAGGGTTCCCTCCCTCCCATATTCCAAAGTAAGGTAAAGTAGTATAGTAAAATTTGTTACCTTGTAACCTTGGATAATAGTAAGACAATGTCTTTTGTTTTTTCATACCACCACTACTATAATAGTTTACATACTCTTTCTAAATCTCTCAAAAGGGGATGAATAGGGTTTCGGGTCAAGGAAGTCTCTTACGTTTCGAGGAGATGGGTCGAATGTCGACTTTCTCTTCATTATCTTACGATTTGGAGGAAAGAGGGAACGGTGATGGAAGAATATAAGAGAAATATACACTTACTTCTCACAAAACTTGACCTTGTTACCCAGTTTACATTTGTATATAGAAAGAGAGACTAAACTTACCATTTCGACCCCTTTAAACCAAGTAGAAAAAAATTCGTGCCACTGTGGTCGTGTGAGAGACGAACTGAACGAAGAATTATCGAGTAAGTTTATAAAACTATAGGCTACGAAGTCTTTAGAACAGGAATTTAAATTATGACAGCGTTCGAA

>Gm13ciRNA1147

GTCAGTACTATTATTATCATCATACCACTCTCCTTCTTTTCCTTCCACAGTTCATGCATTCATTTTTCACGCATTTAGAATTTTGAGTTGTCCCATCTTCATTCAATGGTGGTTATAATTGGACCATGCACTTAAAAAAGGCAACTTCCAAAGGTTTATAATTTTTTTCAGTTATACTTTATTTTATTATTTTAATGGTTAGGATTTCATGCTGTCACCTTTGTGGCTGCAGAAAATCAGAAAATCCACACTTGTTTATAAATGAACTACTGATTGTGTGAATGTGTGAAAAATTGACTGCACTGAATCTGCTTCTCCTTGTTTTTCTCCCCTTTCCCTTGTTGTCAGCTACAATGGTGTTTGTTAGACAGTTGTCTTATGTTAAGTGATGTCTTTTGAAAAGTGCATGATCTTCCATGTTCA

>Gm13ciRNA1148

GTATGAAATTTTATTAAAGCCAGCCAAAAATGTAAAATCCCTCAGAATCCGAATAAATTAGACTTTTAGTGCCATTAAGCTGTGTTTGGACCTGTCCTTAAAATGCTTGTGTAACTATTATTGGTTCCTAAACAAGTAGAGTATTTTATTCTCTAATACTCATAATAAAGACTAATCAAACTTAATTAGGAGAAAGTAGAGTTAAACTATGCTGTTTTTTCTTGGTTTGCT

>Gm13ciRNA1149

CATTATTAATTGGATATCAAGACAAAGGAAATTGACACGAAACTGGACCTGCTAAATAAACGAATGACGAAACAAAGAATCGAATACGTAACCCTTGTGGACTACAACTGACATTAATGACGACCCGGACAATTTACACAACATACGCGTGTGTATACAAACACGATCTTTGAGTAGTTTGTTTAATTACTTTTATATTAAAAATTACCAGTGTCAACTCATCAAACTCGAATTGGTCTAGTATATTAAACACACGTAAAAAAATAACCATTACACACACACATATATATATATATATATATATATATATAATTAAACACGATCAAATATAAAGACTAATGATTTAAACTTCTGTAGTCTTCTAGAGGTATTACTCGTACGTTTTGACACCATCGTAGTTTAATTCTATTTAACAGACGTAGAAAACTAAATTATTTCGTAACTAACAGTTAGGTAAATGCTCAAATTAGTCCTACCTTCTAGAAAAAAAAAAAAAAAAATTTCACAACGAAAATACGATCAAGTATAAAGAATGGGGAACACAAAA

>Gm12ciRNA115

GTATCCTTTGATAATCTATAATATTGTCTCCTTTAAGCCTGTCGTGGTTGGTTTGATCCAGAAATAATTACTACCTCCGGTCTCTATTATAAGCAACAAAAAAACACTTATTTTTTGGTCTCAAATATAAGTAAAAGTTAACTTTGTCTTATTTAATGGGACTATCTTCAAAATGTCCTTCATTTAATAGGGCCAAAGACTTTTTTATGTTTGATATCAAGTTCCAATGAAGGGTAGTTTAGGAAAAAAAATTTACTTTAGTTTTGGGATTAACACAATTTAATGAAATTAACTAATTTCTTAATAAGTGTGAAATCATTTTTTTTTTTTTTTGCTTATAATAGAAACCGGAGGGAGTATGTATCTTCCTTCACACCCACACAAATTTCAAACTTATATATTTTCATTATGTTCACTAAACATGGCATTTTTTCTCGGGGGTGAAGGTGGGGTTTGGTCAACATCTAGGTGTGAATTTAATTCTGGAATATATGTTGTTTCTGAAATGGAATTATTTGTTCTTGGAGTATCATTCTT

>Gm13ciRNA1150

CATTTCTCGTAGATCTTGAGTCTACTTATCACGTGAATTAAAACAAAGGTTAAAACCAAACCACTACAACGACTTAAGACTGTACCAGAACTACAGACCTAAACTAAAAACTGTTACAACTCGACAAGAATACAAGAGGTAACGAGGAAAAGAGAATACCTCCTACAACTACAATAACACAACGAGAAACCTCAAAATTGCATCCTTCTCAAACCGAGATTTTTTAATGAAAAACAACTACAACAAACCAAAAAAATTACGTTTGTAGCTTTTCATCATTACACCTAAAATCTGGAAAAAAAACCCCAAAACCTAATTTTAATTTTTCGGAAGGTAGTTTTACAGATTTTCAATAAAGAGGACCGATGAACGGACCGTTTATA

>Gm13ciRNA1151

CATAAGTAAGGAAACGAAAAGTTAATTCTAGGTCTGCCGGAGCAAAACGGGTTATGGATGGAAGGAGATCTTAGAACTACTACAATTAACGTTAAGAGATATTAGAAGTGTAATCAAAGAGAAGTATAAGGAACGCTTAAACATTACTTGTAAACATACTCAAGTCTAATTCCTTGGAAACAAAT

>Gm13ciRNA1152

GTAATGTTCATTTTTTTTTGTTGATAAGATATCATGGATGAAACATGACATTGATATGATATACTGGTTGTTTGTGAGTTTTCCATGATTTTGTCAGGATAGTTTTCTTTGTTTTTTATTCAACATTTGATATGATTTCCTAATAAAGGCAAACAGTTTGTCTGTTACAGCTTGTAAATTAAATATGTCATGACTAGATTTATGATCCGCAACTTGGTCAAAAGGTTTCAACATGGCCTTTTAGCTCCCAATGTTTGGTTTGGATACCCAAAATGTAGATGTTTCAGAGTTCGCTCCCTGTCAAACTATATCATTTCCTGTTTTCCAGTAAGAATATATAGAAGACTTTAAACTGGTGATTAGTTTTATTATGTATCTACTGCTGAGCTGAACGATATGATGCTTAAAATATTTGTACATCCTTCGAGAAGGTACTGTAATATCAACTATCAATTTAAGTTGGTATTAGAGTTCGTTGATATCTAGCAATCAGCTGGTTTCAAAGCTATGCAATTGTTTGGATTTTCACAGTTTCCCCGCCAATTCATGTTATACTTAATTTACTTCCAGTGTGAAATCTATGAAGACTCGTGCATTTGAATATTTGTGACTGTACTTATGCATGGTTAAACTGTTGACTTGAAAACTTCATATTTGATT

>Gm13ciRNA1153

GTACATGAGTTCACTTACTCACTTAATGGTCTACAGTTTCACTGCACTAGATTTTATTAGTATAAATATGCTCTCTGGAAGGGTAATTATATTAAACTAGTGTACCACATTGCAGTATATTTCTTTCCTTCCTCTCCCAACCTTTCATTTGAATCCTTCATTGAGTTTCATGTAGTTATTAGAATCTTACAATTCATGATTTGAATCGTACGATTCAGCTAGCTATCGATTCATATCATAAGATGAATCTCAAATCATTTTGAATCTTACAATACATGAATGAAACGGTGCAATTTCGTATCATCGATACAAATCACACCATACAACAATTCATTGTTTTTCTCTTTTTAAACTTTTCTTTCACCAATTCTCATGAAAGAAGTTGAAAATTCATCTAAGCTTAAATTGTTAAATGAAAAGGAATTGAAGGAGCTCTTTTGAACCTAACCTATTAGATTACTTCACTACTTTATTTGTCACTTACTCATTCTATGCATGCACTATATTTGGCCATTCCATTGCTTTGTCTCCATTCTTAAAATAGAGGCTTCACAAACTTCTAATTTTGTTTTATGGGACTTATGATTTTATCTATTTCTAGTTTAGAATAATTACATTGAAAACTATATATCATCTATTTTTTTTTTTTTGATCAGCAAAGATAAATGTATATATAATTCTGAGTACCAAAGGTACAAAATTACAGTGTTATGATAGACCGTACAGTTGGTTCCAGTTATCAGACTTAATGTAGTCTCGATAGGAACAAAGTCAGGCCACATACATGAGTGTAAATCTACTGTTAGTCTATGTTTCTAGCCTCAGCTAATAAACAAACCCTGCTCTAAGATTACTAGACCATTGATTAAAATGAGTTGCAAAATCCTTCTCCAAACTTCTAAGCCATGTCCAGAGTAATAAAGCTGCCTCATCCATCAATTTGTTGCTGTTGAAGGTGTCATCGGAGAAAACTATATATCATCTATTAGATGTATTTAAAGTTATTTTCATTTTCTCTTAATCTTAGGATTTGATATAAATTATGATTCATGATTCAAAAATTTGCATGGCAATTCTCAATTTTAATCTTGATATGATAACCTTCATCCCATCAAGATAGTGAGGTGTTCTGATATAATTCTACTTTTGGAATCACCAAATAAGTTCTTTAAAAAGTTAATAAGTTAGATTAGTAACTATCACACACATAGAAGAGATGAGTGGGAGTGGATGGTAGATCCAAGTGGGAGATACACGGCGCAAACAGATTACAAATGGATGAGGGAGACAGCAGTTGAGGGGATTCAGGATCAGTCTTTTGAGGAGCTGTGGAAGTTGAAGGTGCCACTTAAATTTGGAGTCTTTGCATGGAGGCTATTTAGAGATAGACTGCCTACTGAAGTAAACTTACATAGGAGACAAGTAGAGCTCATGGATAGGAGCTGCCCATTTTGTACAAGTATGGAAGAGGAAGCAGGACATATGTTCTTCCACTGCAGTAAAGTCATACCTATCTGGTGGGAAACATATTCCCGGGTGAATAATTCTGCAGACTTACCAAATGATCTAAGACAGCATTTCCTACATCATGGATCTATATTGAATCAGGGGACTAGGGCTAATAGATGGAAGTGTTGGTGGTTGGCCGTGACTTGGACTATTTGGCAGCAAAGGAACAAGATGATATTCTCCAGTGAATCATTTGACAGTAACAAAGTGATGGATGAGGCAGCTTTCCTGTTGTGGACATGGCTGAGGCATATGGAGAAAGACTTTTCAATGCATTTCAATCAATGGACTAGTAACCTTAGAGCAGGGATTTTGTATCAGTAAAGATTAACAACATAGAATGTCACTACATTTTACTAATATGTTCTGCCCTGGCTGTGGTTCCAATCTAGACTCCATTCAGTCTGAGTCGGAACCATTACTCTGGGTTCTTAAACATATGTATTTTAGTACCTCTGGTACTCTTTTAGTATATATAAAGTGTTATCTTTGCTGATAAAAAAGATTAGTAACTATTTTGGTAGGTTGATATCCTTGTTCTGACCTTTGCTTACCATTATGGAAGCTTACTGAAAACACAAATTACTTTGATTTTTTGACTT

>Gm13ciRNA1154

GTATTCTAGTAGCCCTTTTGTTGGAGGTAGGGATAGTAAATAGTATATGAATGCAATCAATGCACAGTTTTTGTGTTGAGATTCTCTTAGAAATTAATTACGGTTTAACAGGCATTGTGCATGCTTCTTAATTTGTGCACAGCTTGCATGCATGGTGTACATTTTCAGGAGACTCTCTTTAATCAGAAGTTTTGAACCTAGTTTACTTTATCACTGAACTTGAACTGGGCAAAGATCCATTTTGAAAAACCAAATTTGGAAAAAACAGGTTTAAAAACCAGTTCGAAACTGGTTCAGTTTTCAGTACTGGTTTATTAAAGTGGTTTGATTCAGAACCAGTTCGCTGAAGTTGTTCAGTTTGGTTCAATTTTAAAAAAAAAACTTTAAAAAATTGAATCATGCATGGAGCTAGTTGGAGTTTGGCATAAGGCACTTACT

>Gm13ciRNA1155

CATTTAAAGAGAATGGTGTTTTTAGTACAAACTAAGTAAACAATTTACGAAGTAGAGATAAACATCTTAATATTTCTGAAAAAAAAAAGGGGTACCCGGATCCCAATCAGGATTCAGGTCATCTGTATATCTCAAATAACTCTTTTTAGGTTCGGGGTAAAGCCAATCTCTAGTTTGGTTATATCTCATATATTCACACCCCCCCCCCCCCCCCCCCGTTGGGAGTGGGATTCTCGATCAAAAACCCCAACTCAATCTGGGTTTGGGTGTAAAATTCTCTCAGTAAATCACCAGTCCTATTTTTTTCTATCCTGCTTATTCGATAGTATAGGACACAAAAATTACATAACAAACCACTCGACTGTTCTATCCTATTCGAATAGGACAGTACAAGTAATCAAGTTTTTAATAGGTCTTCTCCCTCGATCCAATCCTTGTCCATCCTAACCTATCTCTTTTAAAGGTGGAAAGTCCGAAAGTGGAACGATGATGCGTTAACAAAACGAGGTGGTGGTGGTGATGGTGTTAACAACGATGCTACATGGTCGTAACAACGGTGGTGTTGGTAACGATGACAACGGTAGTGATGATAACGACGACGGTAGTGATCGTACATAGTACTAACGAAGATATTAACAATGGAGATGA

>Gm13ciRNA1156

GTTCGTTTTCAGCTTCCATTCATTCTCTCTTTTCTTGTTGCTGAATGTGCTCCAATTTTTGTGACTCATTTGGTTCTTCTTCTACACTAATTTTTTGGATGTCAATGTTCTGTTTCGAAAAAGAGAGATTTTGTGCTAGAAACACACTTCGCACTATCATTATAACGGCAACGGTGTAACAGGTTATTAATAATTCATAAAAATATAAAATAAAGAAAAATTCTTAATTCATTGTTGCTAACCTCGTTTATTCAGGGAATCAGGGTAGTGCAAGTTTTCATCTTTCTCTTTTACCTTAATCAGTTTTGTGCTCAAGTTTTCTGTATGAGTTTTGGGACTAAATAGAATGCAGAAGTTTGTAGTTAATGTTGTCTTCAATTAATTTTGATTTGAATGCGATGCTCCCTTTGAACATCATATAT

>Gm13ciRNA1157

GTAATATTATTGTTTCTTCTTTTAATAAAAAAAACTTTACAAATTATGAATTCATTTTTGGCAACCTTTGATTTGTTGGCGTTGCTACGTTCTTTTGGCCATAACTCATCAACAACTTTGCATGCATACATAATAACAAGTTGATTCATATTTTTTGTTCTTATTAAAAGAAATTACAAGGGGTGATTTTTGTCTGCTAAGTTGTAAAAAATATGTATAATAGGATTGGGGTGATGTGAGCCGTTAGATCTTGTGAGAAATCGACGGTTGATATGTATGTATATTGCGGTATAATGA

>Gm13ciRNA1158

CATTTCACAGAAGAGAGACATTTACAAGATATCTCTAAATAAAAAAGGAATTCTTATTTAATGTTCAGATTGGGTAAATATGGACACTAGAAAAGACCACGGACGAAGGATGGAGATTCAAACTTAAATAGTTTAAACAAACAAACTTCCACTGATGTAATTGTCTCGAACGACTCGTTTTTTTTTTTTTTTTGTAATTGTCTCGAATAGACAAAATACTTTAAAAGAAACCTTTGAATGGACATTAGGACATATATCAAACATAAAACAAACGTCGTTCATAAATAGATTTAGTAGGTAAGTCTCAAAATACACACATCGTTTTATCCTGAAAACTATTTTTACACAAAAGAAGATCAGTAAGAAAAACTTTTTAGAATTTTTTAATCGTGTACGTAGAATTCTCCAATAGATCCATAGACGATACTAGAGTGAAGAGCAAGTTTAAACTTTTGAAACAAAACGTATAAATACGACAAAAAACTACCTCCTGGGTAACTTCCTCTAATAAAAACCTATATGTTTAACACTACCAAAGA

>Gm13ciRNA1159

CATATATAGAACCTAAGTAAAGTAAAACAAAGTTTTACAAGAACCGTAAAACGGAACGGAAAAAAATAAGGAACATAAAGGGAGAACAACAGTTTAACTCAGAAAGTATAAGTGTCATAGGAACTGATCATAAAGAAGAACTTGTGTACATAACTCAAGTGTCAATTATTGATTAAATAACGTATCCCAAAGTTCAGTGGAGTTTCATCAATCATAAATGTAAAGGAACACACACTTATGTAAAATACAAAGCTCATAGTATATTTCTCGAAAAGATAAAATGTGGAAGAGATGGAGTTTTCTTTCTATGACTACTATCCAAAGATTAACGTGACTAATAACGACGTACGTACAATGAATATTTATTCTACCTCTTATTCATGAAAACAGCAACCGTCAGGTTTTATAAATCTTTAGATCAACGAGAAGTCTCAATACTCGACCAATTCTCCAAATTTCTTCTTTGACTAAGACCCGGACATAGCAGACTTCGTGGTTACTCGTCAATTCGACGTGTCGACTTCTTTAAAACATCAACTAAAACTCTACTGTTTGCATCTGTAACCTAAGGTATATTACGAAAACTCATAATACAGAAATAAGTTTCATCACAGTAGTATACGGATGTAACAATCAAATACGGTCTGACACGGTCTGGTTTAACAAGTTCAGTTGTTTAATGAGTATATTAAATCTAATTACACATGAAAATCGAGGAAAATAATACACAAGACTTAAAGA

>Gm12ciRNA116

CAAGCATAGGTAAAGGAGCAATTCTAGGGTTAAAGTTAAAGTAACTAGCTACGGTTTTTGGTTTCCTTGAGCTTTAGGCGAGCACGACGGAACGAAACAACTAAAAAAATCATAGCGCACTGGGTTCTCGAACTGACTTGTTTAATGACGATAAAGGCAAATTCCAGCTAAGGTTTTGCCAAAGAAAAAGTGAAATGTAAGAAATTAAAGTCACTACGACTTAGCCCCCAAAGCTTAGGGAGATAAAAAACCAAAACTAGAAAGAACCGCAGCTTAACATCAAGACCTTAAAGGAAGCAAGACACAAACTTAAACTATCAATACTTATAAAACCA

>Gm13ciRNA1160

CATGAAGTAAAATGAGACCTGATTACCTAAGGATAAAGAAAAATACCAATATATTCAAGCAAACAAATCGATGTACTGAAAACATCTTCGTCTAAACACAATATAAAATAACTAGTGTTCGAGATAGATCATGCTCTCCTATCTAAAAAAGTAAAAGACTTTTAGATAAAATACTACGACACCGGACACCGATACTGAACATACATGAAGGATTCGACCTCCTAATAACGGAACGTTTATCAACTAAGTATACGACGAGACGACAACCGGTTCATAACCTTAAATCCTTCTTAACCACTACGTAAGGATCATCGTTTTACGTTTTCGTTAATTAGAACAAAACTTTAACTCAAAATGCAATGT

>Gm13ciRNA1161

CATGTTTTTCTGAAGAATACGCCCCAAGTATAGTCATAGATGTATAAATATATAAAAAAACGTAAGTATTAAAGTACATCCTAGCTTTGTTACATTTAAACTGTTCCTATGAAAAGTAAACAAATAAAAAGGACCAAATAGTTCATTTTACTGTACCTCTAATAATTACTAGTACCTCTAATAATTCGTACGTCTATGAACACAAAGGATCACAGTAAAGATTAAAGGTACCGTAGTAAAAACACGTAAATCTTAATCGTGAACAATGGATTCGGATT

>Gm13ciRNA1162

GTTCTCTTCCATCTATTTTTTAATTACTTTGATACTTTCATAGGCTTTGCTGCGTTGGTTATTGTTCCTACTCCATTGGAAGTTTGTAATCGATAATAGCTTAATCGCCGGGAAAGATAGATTCATAGGAAATTGATTCTGAATGTTAGTTCAAACAATATTGACGTGAAACTAGGGTTTTCGTACTTGAATAGAGCTCGTTGCGTTTCAGGGCCTTTGGTTGAGAGTGAGAGAATCTCTAGGGCTTTGCTTGGTGAAAGGGAATTGGAGGGAAAAGATTGGAGAATGATCTGTGTATTCGGACTAGTTATTCTTCCGTTGCAAATTTACTTTTTTCGGTTGAAGTTCTTAAACTGGACCAATTACTGTAATTATTGCATTGAGTCTCATTGTTGAGTGTTTTGGAAGGTTTATAGGTGATTAGATCATGTCTTGCAGCAGATCATTGTTTCCTTTTAATCATAGATTGAATTTTTCTGGATGATATTTGAAATATAGTCATTAGTGCACAAATT

>Gm13ciRNA1163

GTAGGATCCACTATTATCAATTTCAAGAATCTTTTACTTTTTAATTTAAACATGATCTATCTGTTATAATTATAGATTTAGCTCTAGTGTGCTTGTGAATTTGTACTTATAAAGGCTGTGTGATGCCATCACTTGTCCCCTGCTTATGCCAATACCGAAATTGGGGAAAAACTAATAGACTTGAAAACAGAGTTCCGGCACATATAATGAAGTATATAGTGCAATAACACTCCAGGTTTGCATGAGAAGAAATCTTTTTTTCTACCATTGCTCATTATTTTTCCTAGTGATTGGATTTATATACTTATTTTTCAATAGTAAATAGATGAGATAATATTTCAAAGAATATGGGTATTAGCTGTAACATGCGCACTTGCCATCCCTTTAGTCTGAATGAAATATATTGAGATAGATGTTTTATTCTTTATTTCCTGTTAGCCTTATTCTGA

>Gm13ciRNA1164

CAATCAAAGTAACGGTAATGTGAACGTCGTTAACGATAGCAATAGTAATAGTACAGGCACTAATGACTAATAATTACGTTTTATTACTGGTATTATCACTATTGTAGTGTGAAAAAATGTGGGAAGTGCTTAAGTGCAAAGACTACGTTTTATAACTCCTTATTAATTAAGACACAAGGCAGGTTGTGAACAATAAACGTACTACCGTCACTACCAAAAAGGATTAGAACAATCTTTTCTTTTTTTATTCTCTCTTTCTTTCTTGTCCAAACTCAAAAAATCTCTACAAGAATTACATACTTACTTGTTTAAGCAAAGATGAGACTGACTCCATAGGAAGGTTCTATACGATTATGACTTCACCAAATAACCTAGTACGAGTGCCTGATTAACTTTTTATAAATCAGTTATGGAGCTGAGACAAGAAAGGACTCTTTGAAAAACCGGAGACCCCTAATCCAAAAACACCTGTTAAGGTTACTTCGAGTCTTCTAGACTTACGTTCAATACTTTAGACACCCTTTGGTTTATCGTTACCCCAAGTGAAAATTCAAAACGGTACATTGGACACATAAGATCTTATGAAAACGTGACCAAGTGAAAACTAAAGAACAAATAATTAACAAAAAGAATTAGA

>Gm13ciRNA1165

GTATATATGTATCTCTCTTATTCCACTTTATATAATGGAGTATGGACTAATGGAGTGTCCATTTTCTGTAAATCACCATATTCTTTTTTCATGGCAACACAACTTCCAAAAGTTACTAGTTATTGCAGTTCCTGGTTGGATGCTACATTCGTGTATATATTTTTGTTTCATTTTTTGTCTTGTCTTGGGATAAAACTTGACTTTCTGA

>Gm13ciRNA1166

CATTTATGTACAGTACTAAAAGTATCTGAAAACGTTATTGGCTAAATGAACCATCAGAACCACTAAGAAAACTTATTTTTAATCTATAACACGAGTAGTTGTATAGTACCTATAAATGCGGTGGTCAAAGACCGTTAGTTAATAACAAGTTTATTTCATACTTTACAGCTAAAATGAGCAGACACAACGGTTTAGAAAATTTACATTTTGGATAAACTTGAGTCTTTCAAACGATTTTTGTTCGGAGTACGTATACCTAAATCAAAGAGGTTCATCCGTACCCCTAATTGAAAAATACATTGTCAACTCAACACCTTAATGAGTACCAAAGTAATATCTCTCTCAAAGCCAACAGTCTTTAAAAACCTCTTTAAATACCAACGGTAGTTTGAGATCGATAACTAGAAGAGAGTAGTGTATAGAAATCACTTCTTTCTTGTAAAACTATCGGGAGAGATTTATATACTACCGTATACGTTAGGTGTATAAACAACATAATAATATACGAAT

>Gm13ciRNA1167

GTAATTCTGCAGCTGCTTGCCTAGTAATAGATAGAAAACTGGAAAAATTGAATTGTTGTTGATTGCTTCTGATATGAAGATATATATACGGCAACATGTAATAGAATATTGTTACATAGGAAGACAGATAAGATAAGATAAAACACATTCTTAGCTAACAGATAAAAGTCCTAAGAATTCAAAAGTCAAAAGAAAGATAATGCTGTCTTCAGTTCCAGATAATTTCTGATTTGATAAACTCATTCCTTAACTGTAATATGTTTAATCAGTGTTATAAATTGCTTTTTGACTGGAATTGTTTCCTAATGAAAGGGATGGTTCTATATATTCTGCTTGAACTGTCTATTTCTTTGTATTGTTTGATAAATGAATGCCGAGTCTACTGGTATTTCTAATAAGAACTCTGATTTGTCAAGTTGATGGATATGTCTATAGTTGCAGCTTAATCTTAAACAAAATTGTGCCAGTGTTGAAAGCTGGCTTGTTATGCCAGTAATCTTGTGCAGAGAAATAAGAGGCCACTTGAGATGATTTCATACTATTTTTGCCATAGAGGAAATACAGACATGTATTTTTTTTAAATGGTCCTTGACGCCTCATATACCTGGGATATTGTGATCCAGGCAAAGAACCAGTACTCCCTCATTCAGGGTTCCTCTGTTTCAATTATCAAGGATGTTTTCTCTTTACATTTATTTGTGAAATGGATCTTATTTTCTGTTGTGCATGGGTGCGGAGGGCTCATTAAATGTTTTATTGTATCATCTACTCT

>Gm13ciRNA1168

GTAATTCTGCAGCTGCTTGCCTAGTAATAGATAGAAAACTGGAAAAATTGAATTGTTGTTGATTGCTTCTGATATGAAGATATATATACGGCAACATGTAATAGAATATTGTTACATAGGAAGACAGATAAGATAAGATAAAACACATTCTTAGCTAACAGATAAAAGTCCTAAGAATTCAAAAGTCAAAAGAAAGATAATGCTGTCTTCAGTTCCAGATAATTTCTGATTTGATAAACTCATTCCTTAACTGTAATATGTTTAATCAGTGTTATAAATTGCTTTTTGACTGGAATTGTTTCCTAATGAAAGGGATGGTTCTATATATTCTGCTTGAACTGTCTATTTCTTTGTATTGTTTGATAAATGAATGCCGAGTCTACTGGTATTTCTAATAAGAACTCTGATTTGTCAAGTTGATGGATATGTCTATAGTTGCAGCTTAATCTTAAACAAAATTGTGCCAGTGTTGAAAGCTGGCTTGTTATGCCAGTAATCTTGTGCAGAGAAATAAGAGGCCACTTGAGATGATTTCATACTATTTTTGCCATAGAGGAAATACAGACATGTATTTTTTTTAAATGGTCCTTGACGCCTCATATACCTGGGATATTGTGATCCAGGCAAAGAACCAGTACTCCCTCATTCAGGGTTCCTCTGTTTCAATTATCAAGGATGTTTTCTCTTTACATTTATTTGTGAAATGGATCTTATTTTCTGTTGTGCATGGGTGCGGAGGGCTCATTAAATGTTTTATTGTATCATCTACTCTGTATTCT

>Gm13ciRNA1169

GTAAGTGATTCTATCCTTTCTTTGTTTCACTTGAATCCTGTTTCATTTGTATCAGTTCTTAATTTCTTATATTCTTTTAGCTGCCCTGTTGTTTGAATTTGTCAGATGTTCCCGAGGGGAATTGTGTGGCTTTATAGGCTTATTGCACTTGGTTTTTATCTCCACATCACCCAAGCTTTTTGTATTTTTAGTTGCATGTAAAATTGTGAAACTTAAGATGTGCTTGTAATTTATAATTAGATTGAGGATTAAATTGCTTGAAGTTGTTGTGTTTACTTGTATCATTGATGACATAATGCACGAGAAGGT

>Gm12ciRNA117

CATGCTTGACCGTAAGTAAAACGTATGGTAAAGGGATAACTGCTATATAAAGAATTCATTTCGTCGTCTTATAATCGATCCGAACCCAAAATCGGTAGAAATCTTCGTTCATTATGACACAGATTTGTTGATAATAAAACTCGAAGCTGGGTTGAGAAAAACATTCGAAATTTGGTTGTATTTAAAATTAACTTGAAATTTACTTTCCAACACATACCCCGGTTACCTGACCTTATATCTACTGATCAACGTGAAACAGTAAGAACGACCTAGAACCTGAACATAGTACCACATGAATGCTCAAACATTTAGAACTTTATCACTAACACTTTACGAGACAAGAGAACCTTAATGAAGGTTTATTACTAATTACTTCGTATACCTAACTAATCTTCAAATCCTGTCCGTGTTTTATATCTGAACACGAAAATGACTGAACTCAAGACATGGTCTTAAATTAACCCAAGGAAAAAACCCTACCCTTGTTTTGTTTATTCTTTGGTTACACAGATAAATAGGTAATATACAGTGGTTCAGAGGAGAGCTAGTGAAGAAAAATTACAGGTAAATCAAATGTTTAAATCCAATCCCAATCCCAGTCCCAATCCCGGTCACAGTTACAAAACCCGTTGGAAGAAACCAAAGGTTTCGTTTTATGGAGAAACATTTTCCACCAAGACTTAGACTTAAAGAACAGTCCTTGTTACAAAATAGTATACCTTTAAGTATACATAATTCTATAGTCTGTTTATTTGACATAGAGATAATACAACCGTTTAAAACAACCAGT

>Gm13ciRNA1170

CATTCGTGAAATAAAATAAAGGGTAAATGTGGAGGAATACAATTTATGGGAAACCGTTAAACTTAATACAACCTTTCGGTGTATTCAAACTAATAAAGACTAAACAATATTAACGCCGGAGAAACCCTCGAAAGAATTAACATCGTAGTTACTCCACACAAAGAACAAATCAGGAAATAACAAATCTTTATACCGGGCTTATTACGAATATATACTGAACTATTAAGAATAGATTCAGTTAAAACATTTTAAATTAATCTATAAATAGAGTTTAAAATACTATAATCTTGAATAGAATTTAAGTTATAACTATAGTGGACATAACAGTGTGAGATCCGAGTAATGGATCAACACTCCTTCACACAATCTTTCAAAGGAATTATTGCCACTGTTGATTGGACGGAATTAACACTGGAGACACCCCAGACCAATGGAATCAATCTTGGAGGGTTTGATCAAAACTGAACTCGATTCTGGATTCGAGTTTAAGAAGCTTGAAGGAAGTAACAACAAAAGAAAGGTACCGGTCTCCGGTTATAAACTTTAGATAAACATACCAAATCTAAGTATTAAAT

>Gm13ciRNA1171

GTAAGCCTTTTTTCCAAACCCTCCCTTACTAGTGTGTTATACAGTTTAATTTTGTGCAACTTAGACAATTTGTTATTAACACCAATAGGCAATAGTGTGAGAGTTTGTGAAAAAACAAAAAGATTTTTTTTTAAAATGAAACTAATATGTGAAAATGTCACCTTTGGTTACTTTACATTCATTCGTATTTATTAAATTATTACTATAAAGAATATTCATTTGAGAATGCTAA

>Gm13ciRNA1172

CATTTCTACTCCAAGTCGACGGATTAACCTGAAACGATGTTTTAACCATATATCCCTTCCACTTATTTCGTACGGGTAACTGTAACACAAAATAGGAACTTTCGTTGACACATTCGTACAGGTATACGTAAACTAGTAATTTGTGATAAACGAGTAACTCAACAAATAGTCGAAAGGAAAACTTCGATACCGATTCATACGACTCTACTACGAGTACGATCATATTCATAAGTAGAACTTGTAATATTATTAGATACATAAAAAAATCCGTCATCGTTAAATGTCATATCAAAAGAACAACTTTAATGAGAAAGTACGAACCATTTTAATAAATTAATAAATCAAAGGTAGAAGCCTCAATATAACGTACACCTACAAGGCACATCACGAAGTTTTGGGTTTAAATCGAAAAATAATCGAA

>Gm13ciRNA1173

GTAAAATTCTCCTTCCATCGCTGCTTCCTTTTCCTTTTCGCGGATTCCCAAATCTACATATTCCTCTTCCTGCACCCCCGTTTGTGTTCCCCGATTCGCTTCTCGCCTTTTGCACGTGCGAATTAGGGTTTCAATTCGCGTTTGCCATTTCATGAGGCCAGATCTAGTTGTATTGCATGCATTGGTATTGCGTAGCTCGCTGGTTTTGTTTACTGTTATTTAGGTTAAATGCAGGCGATGTGTAATTTTCTGTAGCCATGTAATCGATGATTGATGTACCTTTTTTTTTTATAAAAAAAATCGAAATTAAGTTTTGTTTACATGGTATCGTTTTTAGGATCAAAAGATTGAACCAGTGAAAATTGGGCATTTGGTTGTTCTTGTATTCTCAGTAAACTGGAAATTTTAGTGAGCACAAATTGAAGAAGGTTTGCTAATGTAGATGGGAACCGTCTTTTAATGTAGTGTGTTTGGGTGAGTTTTTAAAAAATGATTTTGACTTTGCAAAATTGATTTTCAAGCAATGTGATTTATGTTTCATGTTTCGATGTTTTTATTTTAAAAGCAGGATACGATTTGTTATCCAATGAAGTTATCTATAATTGCTTCAATGCAATCAATTATGGACTCGTGGATCATTTTTTTTAACATAAAACTAAACATATGAATATTTACTTAAAATCATCTTAGCTAGTATTTCAACTAAACATGCCATTAGGAACTTTACAGTGCACAGCGTTTTTTATTTCTTATTTTTGTTTGATTTGGGCTTCGTTATTTTTGATGAATTGTCACTTGGATGCTGAGATATTCTCTAGATGTACGATATTTTGGCTCCTTGCTGGTA

>Gm13ciRNA1174

GTATATTTGTCTTTCATCTTTTACTCTAGTTGATGTATATTTAAAGAGACCTATTCATTTCCTAAAGAAATGTAGTAAACATCAATCAGCTTTCCAGTATCAACCTGTGTTTCACTTCTATAAGATGTAATGATACATGCAGAGAAATAGAGATATGCATGCATGCAGATTACCAGGTCTGAAAATAGTTTTTAAATTTAATTTTTATGTATCTTAATCACTTTGCTGGAATATTTTTTGTTAAGAAGATTGGAGAAAAGAATGTTGTTCAAGTGGTCACAAATAGTGAAAGTAAATATGTTATAGCGGAGAATAAACATGAAAATTGTTTGTGGATGCCGACTCAGTCTGTATATGCTAGTCTATCTGGATCTTAGTTTTGATTACTAGAGCTACCTGCCCCCTGGTGTGGCCATAATGTACTGTAAATCACTGAAAAATATTAACCTGCTCTTGATTGTGAACAGTATTGTTAT

>Gm13ciRNA1175

CATTAACTAAGACACAAAGGCAGAAACAGTTTCTTCAATTGATTTAGTCAACTTAATATATAAAACGTCGGTGTAGAGGTTTTTCCTAGGAAACTTACAAAAACCATAAATATTTTGTAACCTATATACTAATTCTCTTTTTCCCAATATGTGACAAAATGTTATAGTAGGTTAGTGTTGGATGGTACATACCATTCTAACAACTGAAAATACCATTAATAAAATTTTCAGTATAGTTGTCACTAAATACTAATCTACTGCCACATTTTAATTCACGTACTGGTAATTTGAGACACTAATTACGAATTGGTAAAATGAGATTTATTAATGGGCGACTCGAAAATCTATGAAACTCGACTTTGACTAAAGACATGTCGACTACAACGATGGGTAAATCGTATGTGTTTGTTGTTAATATATCAGAGATGAGAGTTCACCACATTAATAAATCTCTCGAGTGGAGTAATTAGAATAAACTTTTTTAACAGTTGATACTTCATTTCTACCTTAATGATACTTGAATAACTTATAAGTAAAACTTGTTGGACCTGACGGAACT

>Gm13ciRNA1176

GTACCTTTTGAACCATCAACCACCACTCATTGGTTTCTTATCTTGAAAAATAATTTTGGCTTAATTCCAGCTTTTGTCTCTTATTAGCGTCATTTTGTGAATTTGGTTTATCTACTTTTTTCTTTTCTTTTCAAATTTGCTCCCCTCACTATATTAATTATATAATTTTGGTATATTCGATGATTTGACATTCAATATTTAACAAAAATGTTAATGTGACATGAATGTTGACGGGACAATTAAAATATTAAAAGATTGATCAAAGTTGTACAATTAAAATATTGAAAAATCAAATTTGCCAAAAAAATAAAATAAATGACAGACCAAATTCAGCCAACTGTTTTAAGTTTCATTACTTTAATTAAGAGACTAGTATGAAATTTCAATAAAGATAGAAGCACAATCCTTTGATACATATTGAAGGAAAGGTAGTGGACTAATAGTATTACTTATGAGATCCAT

>Gm13ciRNA1177

GTATTTCATTGCTATTTTCATCTCTTCGCTCTCTAACCGAAGATCCAATCTCCTTCGTTCTTTCCGAGCATTTTCCGAATTCACAATCACATAGAAATGCTCCGTTTCGTTCCGTGTTTTTTTTCAACTACGAATTTTAAATCGCGGTGTAGGGCTTTTAGTTGTTTTTATCAACAAGTTATTGTTATGGCTTCTGATATTAGTATTGTCAATAGTTGTTGATGTTAATCATCGAAATGAAACGAAATCCTAACAATTAAGCTCAGTAACACTTCAATTTCAATGTATTATGGTGATCATTTTAAGAGGTCCATTGGCAACTGCAAAAATAGCCTCAACCGTGTTCACTGTTGTGACTAAGCAGAAACAGTAATATACAATAGATGTAGGCTATGATTGATCTGAATGTACGTGCGCGTGTGTGACCTTACATT

>Gm13ciRNA1178

GTATGTCATTAGTTTGCATGCTTAACATCAAATTAGTCTGTTTGGATGTAGAAATTTTTTACTCAAATATTACCTGATGTCGATCCCTAGAATTCTGAATAATCCTAGGATGATAATTGGTTCCAATCCACTTTGGTCCCACCCGCCAGAACAAATTAGACTGTAGACTGTTGTACCAAAAGGCTATTTTTGAGGGTTCTGTTTCCATTCCAACCACTTTGACTGTGGAAGATACATCCTCTGTCCATCTAGAATTTTCATTTTGTAACTGAGGGTGGTGCCATTAGGCTTGAAAAATTAATCACATTTTCCCTTTATGTCATTGCGTCTTATTACATTCCAACTTGCTTCAAGTTTAATGCATTTGCTGTCAATAACCCTCCTTTTTTTATTTTTGTTGA

>Gm13ciRNA1179

GTAAGCTCTGATTTCTGTTGGTGGGCTCGATTTCTCTCTGCCATACACTGTCTACACTTGACCGCCAGTTCACTAATGTTTGAATATACTTACAAATTTACAATGCATTTAGCATAAGAATTAATGGTTGTGACTCAATGTTGTATACTAGTTTGCCACTCTCTTCTCACCTAGTGGGGGATAACGGCTTGGCTTGTTGTTGTTATTATTCAATTTTTGTTCATCATCATATATAATTTA

>Gm11ciRNA118

GTTTGCTGCTTTAATCAACATATATTGATATGTGACTTGAGTATAGATGGCTCATGATGAATGGTTTATGGAAAGGACATGGATTGACTAATAATTACACTTAATGAGAAGGAAATGTCTTGATGGATCTCTATATGTGATAGTTAAGCTTACCAACTAGCTTCCTACACGCAATATTGCTCTCTCCGTATCCAGATCCTGCTGTTGTGCTATCTTTTCTTTTGTACTGTATTTTAAATTTTAAATTACCTAGTCGGTTGCT

>Gm13ciRNA1180

TATAGGAAAATCAGCAAATTATATTTAAACAGACATCAAACGAAAAAACATACCAAACAAACGAACTAAAAGGGGAATTTCAAATTTTGCTACATTTTTAACCAAATAATTACTAATTAGGTCATTCTTTACATATATTAGATTCAAAACACAAAACGGAATCGATCGATAATACTACAAAAGATCTATACACACGATTGTATTATCTCTTTTGTAATCAAGACGATACTAAAATCACTAAACATGAACTATACCTTAATACTTACGAGAGTATGGAAATCAACAAGAACTTGACCTTTAAAATGACGTTTTAATAAGGACTTCTAACACTCTATAATCAAAAATAAACGAAAGTAAATAACAAAACTAACTAACCTAGTCGATATTCAATAATATGACCAGAGGAAAAATCAAAGGAA

>Gm13ciRNA1181

CATTGTTAGAAAGAAGAACGTAGGAACTAGTAAACGCAAAATATTAAACTAATAAATCATATGTTATTAGAATTGTCGTGTAGACTAAAACCAAAATATTATCATTTCTTTACACCAGAAAGTTTTACCCCAAAAGAATTGTTTATACGTGTCCCTAACCCGTC

>Gm13ciRNA1182

GTTCGCCTCTAGCTCGTCTGGCTATCTTTAGATTCAAGTTGGGAAAGAATTTTTCAAATTTCAATACACAATTGCGAATTTCAATGTACAAAAATAATAAAAAAAGAAGAATTTGTTGCTTCGGAAATAAAAGTTCTTGACTTAAATTCAATCATACACCCTAATTTGGTGGTTGAACAGCGATAGTTATTGTAAACTTTTTTGTATGTCTTTCATTTTTACGGATAAAAAAAAACATACACTTTAAATTTCGGTGGATTTGATTATGCTGTGTGAGGCTGA

>Gm13ciRNA1183

GTTTGGACTTGTAGGAAATTTGACTTATAGGTGCTAAGAAAAGATAAAAGGAGACATGTAAATATTCTTAATTCGTCAATGGAATTACTGGACATGCTTGCTTGTTGGTACTTGGTAGTGTTTTTCACATGAAACCTGAAACCCTGGCTTGGGTTGCTTTAGAATAATTTAGTTTCTATTATTGAACTTTTTTTCTAATTTGGTTACTTTATCTGTTA

>Gm13ciRNA1184

GTTTGGACTTGTAGGAAATTTGACTTATAGGTGCTAAGAAAAGATAAAAGGAGACATGTAAATATTCTTAATTCGTCAATGGAATTACTGGACATGCTTGCTTGTTGGTACTTGGTAGTGTTTTTCACATGAAACCTGAAACCCTGGCTTGGGTTGCTTTAGAATAATTTAGTTTCTATTATTGAACTTTTTTTCTAATTTGGTTACTTTATCT

>Gm13ciRNA1185

GTATCAACTCACCACTTTGACTTATCTCTTGTTTGGCACATAAAATCCGCTTTTGCCAGATCCGAGGGGAATAAAAATGGTAGTGTCATGACTTGGGAACAAGAGTAGAACAACCATTTTTATTCCAATATTTTGGTTTGCTCAAGAATGGACTTGTACTTTCATTCTTGTCCAGTTGCCTATGCTGTTTTCATGCTTAGTACATGAAATAAGCATTGAATGGAATCTTAAGTATAATTTATAACTTTATTACTAGTATAGATAAATAAGGAAAGAAAACAAAAATGAATTAATCCAGTTTGCAAAGTATGACAATTTTGACATTTCTGGGAGGGAATGGGGAATTCCCTATGGGATCAATCAAGGTGTATAATGTAATCATTATTCCATTTTTTCTTTTTTTGCCAAACTAAGGAGCCCCCATTCCTGTGGAATGATCAATCTGTGCTCTAATTATGCTCTGAATAATTATGTTTTTATTACCCTATAATCTTTGTGTTAGTTTGGGTGCTTTTTTCCTTCTTCAATTACTGTTATTATATTGCTAAATTGGGAAGTCTCCATGGCTTGTCAGTTGTCTCAAATGATTGA

>Gm13ciRNA1186

CACTCGTTCAAGAAAACAATTAATTAATGACAGAGGAGAAATACACCACTCTTTACGTTTCCTCCTACTTCTTTCTTATTACTACTCTGAACTATCCTAGACAGGAATAGGGAACAAAGCGAAAACGTAAAATCCTTTTCAGTTGCGACGAATTACAAATAATCAAAATCAACCTACTTCGTTAGATCTAACAATTCGAAAAACAACGAACTGAGATACCGGTCTAACAAACACGAAACACAAGTAAAAAGTATACAT

>Gm13ciRNA1187

CACTCAGTAACGGCGAAGATGAAGATCCCAAAGAAAAAAAGAAGGGGGGGTTTAAGTACAAACCGCGTGAGAAACGATTGTACGCGGAAGAGTACTTGAATAATTAGAGAAAATGACGAACTGACTACGATTACAGAATGAAAAAATATAAATACGCGTTTACAACGACCATCAATATCTTGAAACTAGAATGGATAGTTGGATTCTAATATTCGATTTTAAACGTCAATTGTATATATTTATTAGTAGGCCTTCAGCTTGAACAAAGATTTTAATCTTCACTTGAAATATCGAACACTTTTCAAGTGAACGACATTTGAAAACATCACGAAAAAAATTATAGATAAACAGCTATACTTTCTTTTTACACATTACAAACTTGGTGTGGCGTAACGTACCATAATACAGAACTAACATGGAAATCATCGAAAAACACGTGTACGAA

>Gm13ciRNA1188

CATAACTATATATATATATGCGAAGAGCGTTATAAGGAGCGATAAAGCACAATCACGGCAAGGGAGTGACAATACTTGCTTTGTTGACTATTAACTAGATTACTAATCTAAACTAGAGCACTATTACGTTTATGCCGAACGGATTACTCATTAATCCTTTGGATGTAAACCTGTAAACAAGCACGAGACGAAGGGAACGATAGTAGGTGATAATACTACTTCATCTGCGTTTGGACCTTGTTTCTCTGACTACTTAAACTTTGACGAAAGAAACGCAAAAGTCAACGAGGAATCTAAAGTAGCCACTTCTAATTTACGAGGACATATCCCAAATAAGGCATCTGACAAGGCAATAATTGAAATGTGAGTAAGTTGAAAAACACGGTTCAGATACTACTAGTTCTAACTCAAATAATGTATTCATCTTGGACACGACTCTATTCGACGCCACATCTTAGTATTCACACGTCAAAGCGATCAGTGTTAATTTACGTACTTTTACTATTCTTAAACGTACCTTTTTACCCTTTGTTTTCACTTGTTTATAACAAAACAAAAGTTCGTAATTGAAAACTTTAGGTTGTTTGACAACAATATCTATAATACTCTATAAACCTTTAAGGTCAATTCATGAAATAAAAAAGACTATGTCTGCAAATAAAAAATTTAAGTTCGTTATTCTCCCGTAATGTGACTTATATACGA

>Gm13ciRNA1189

CATCCTATTAATTAATTGTACGTGGTACGATCTATATAACGGACTAATTTTAAAATTTTAGTGTTAGGAGAAAGGAAAAGAGAGAAACGAAGCACACCAAAATGAAAACTCTCACACCAACTATAACCAAAAATTAAGAAGGAAGGTGTTCTAGTTAAATTTCTTACTTATTACTTTACTAGGTATATACACACACATATTATAGACACAAAGGTTAACTTAAGAGCTTCGACTCTTAAACTACAATTCCGTTTAATAAAATATTGAAAGGGTAGAAGTAGAAACACTTAATGGGAAGTTAGCACATAGTTGTTTTACGTTCTTAACTTTTAAATCCACACTATATATATAAAAAACGCTCACACTAATACTCACAAAAAAAAATAAAAAATATAAGTTAACAAAGATGGATATTAAACGTAGTATTTGAGAGGAAAAGACTGATATATACACGCGTGCGTACGTAGTAAGTGCCATGTGTGAATCTAAGTCGGCCCCTTTAAAATAAACCGTGAAACCGGACTTTAGTTGATAAATTAAGTAGGTGTGAGT

>Gm11ciRNA119

CAAGCAGAAAGCTAAGAAAAGGTGAAAGTAAACGAAAATAAGTTATTAGATTACATTAAGCCTGATCTAGTGAAAATAATTTTGTGGTTATTTATTTAAGATTCAAGTAAAAGACATTAAGCCTGATTCTAAGTCAATAGCTGCTATGGATGT

>Gm13ciRNA1190

GTTTCTCACAGCCACTTGAATTTTACTGTGGTTTTATTATTTTTCACTTCAAATTCTGACCCAAACGAGGACTGTAAAATTCGACTGTTGATCAATTTACGTGGAATTGCTTGAATTTGTGGTGGATGACTAGGGAGCACTCTAATTCTGCTGAATTTTGTATCAGATAACGCTGCATTTGTTCAAACCTTAATACTATTTACGTACTCTTAATTTTGTCTCAAGTCATTCGTTATTTGTGCGCAATCTGTTTCAACTACATTTTTCTTCTTTGCAATTTTGCATATTGATTTAATGTTTTGTTTCTAGAGTCTGGACATCCTTAAACTACAACACAAAGGGAATAAACATGTGTTTATGCCTGATAACCTAAGGTTTAAGGCTGAATGTAACCATAAAATTTACTGTGCGAACTGTTGTGCTTACTTTGTTATAGTGTTGGACGCTTCCTTCCAACTGAATCTTATCGCATCT

>Gm13ciRNA1191

GTAGATTCCCATCCCTTATTCGCTCTCCATCCCCCTCCCCCTAAAGAAATATAATCATTTAATCACTTTTTGTAGTGCTGTTTGTCCTGTGCTATAATTGTCTTATTTGATTTAGGCCTTGTTTGGATAAACTTCTTCATAATCTCTTATAGGAGAAAAAAATGAAGGAAAAGAAATAAGTTTCTCCTATAAGCTAAAAGTGGCGTATGCATACATTAAAAACTTGTTTCTGGAGATGCTAATATGAGAGAACTTCTACAAATTAGCCTATGCATAAGCTAATTTTAGATTATGGAATAATTTTATTTCATTTTTCCTTATTTTCATCTATTTATGGAGAAGTTTATCCAAATAGGATCCTAATCTAATGCAATCTTAAGATTTTCTTATCTGTTGTTTTAAGGGAGTAATTTTTCTCT

>Gm13ciRNA1192

CATTAAAGAGAAGAGACGGAGACGATAAGAGTAAGCAGGTACTAAAGGGAAAATTAATATGCTTACTTCTTTAATTACTCTAATAAACTACGAAAATGAAGATTAAACTTTGCGCATAACTAAATCAAACCCCTTAAACAAAGGAAGAGTCGACCCAACAACTAAATCCCAAACCTAGGCTAGATAAGAAACCGGCACGATAAATAAATTGAAAATAACGACCACAAATTCTACAAG

>Gm13ciRNA1193

GTAATATATACATGATCCATAAATATAATCCATGATCTTCAGTTGCTTTTCTGATGTTCCCTTAATCGCTAATGAAATGGAATGTGCACTATCCCACTTGCAATCACTTTATTATTTGTTATTTATTTATGTTCTTCTATTATGTTATGTCCCACCTTGTATCTGCCTACTTTGCTTTTGCCTGCCCTTTAATTCTTTCTCTTTACTGCATGAATCTCAACCATAAATTAAAATATTAATACAAAAATAAACTCGAAACGAATGCTGAGGGATTCAATTGTACTTATTTTACAACTAGGGAGATCAACATGGGCTTCTTAACTTTCCTTTCTGAAATTTCATGAGTATGCACGCACACTTCTCGAATAATAGTAATAATATGATTATATTAAAATATTCCTTTACTTTAGTCTCCAACCTCCCACTTTGTTTGTGAACGCTTACT

>Gm13ciRNA1194

GTATTTCTTTCTATCCTCCTCTCTTCTCTCCACTTAGTTACTTTTTATTTTCTGAAAAACTCATGATCTCGAGAATTTCAATGCCTGATTCGCTGATCTTCACTTCCTAATGTTTTCATCCATCGGATTTAGAAAACCCATTAATTTTTTTTTGCATTTTTTGGCATTAATATCACGAATTTTTTATGGGTGAAATTCCAAGACATGGTTGGAAATGGTTTGCGTGTGTCTAGAATCCAATGTTGATGTGACTGTGTTCTTTTCAGTCAAATTGGGAACATTCTTTTCGTAGTGTTACGGATTATTATGGCTTTGGCATGCTTTTCATGGAAGCATATATGCCTTGTACAATTTATGAGAATTTA

>Gm13ciRNA1195

CAACCAGAAGTAAGTAGTATAGATTAACCCGTTAGTACGAGTGTAGGAATAAAGATGTACAACAGATTATTATATTTTTTCCTTTAACCACAGTAGGAACGAACATGTAGTCGTGGTTAGATATTCGTTAAATTTTCAAAAAACAATAAATGTAGGGTGAAGTTCAAGGAAAAACTGATGTACAGTACAGGAAAAACCAAACAAAACGAACTCATAGTACTGTCCTACTGTGGAACTGACACAACCAGAAAAACTGTGACCAACAACAACGAAATCTATTTGAAAAGATAATGACACTGTGTAAATCTCTTTAGACATAAACGATTACTTTCGTTTACCCTAGTAAGAACAACATCGACCTGTAACTGATGCTCAAGAACACATTTGCGTCATAAAAAAGAGTCGAAAAGAGAGATCGATT

>Gm13ciRNA1196

CATAAATTTAATAGAGAAAAAAACAAGAAAGAAAAGGAAATGAGAAAAACCATTGACCTGTGACCCGGTACACAGGGTTGGAATCTTTGTTAACTGATCAACATACATCGTTCATTGGACTTTGTTAACTTACGCTTATGTTTACACTCATGAATTAAACTATTTATCGTACTAGTTCTTAACACACGTATGAAAGTTGTTCATGTATTGTGTGGTTTGAAGTTAAAGTACTTTAGAAAATGACTTACAACAGGTACCAAGGTCACAATAAGTAAGATCCTTATTACTTGTCCAGATCTTAACACTTTTTGCTTAAGGACTTATTATAGGAACGGTGACGTAGGAGGGTTCCCACGCTTCAGGTAAAGAGTATAA

>Gm13ciRNA1197

CAAACATGAAAAAAAATTTGAAGTAAAGGAGAGATTAGAACACACTAGAACAAGAAACACATTACTTGGTTGTTCAAGAACGATAAAGAATTTTCCTTTTCATATTGTTCTACAACGGTAAGAACACGGCACAATCTGACAATCCGTAGGGTGTTAA

>Gm13ciRNA1198

GTAAGCACATTGGTGATATTTCCTTATGTATATTGTAGTGATTAAGGAAATGCGCTTCACTAACGGCCCATCCCGTCTCTCCCCCCCCCCCTCCCCGAACTGTTTTTAATCCTTCTATTATATGAATTAGCTATGCAGCCTTGAAATTAATTTCTAGCTCTCAATCAAAAGGATATGATCATTACCAACTTACCATTGCTCTTTGAAAATTATAAATATTTCAAAGGGAAGATTAATATAAATGAATGTGCTTTGACGAACTCTAGTCTTCTGGAGACACATTGCAACAATGTCAAAGGCTTAATTCTACATCTCAGACTAATTCTAGAATTATTGGTATAAGGCTTCCTAAACTTGTCATGAATTGATAGAATATTAGTACTCTGTCTCATTAAATGGCATTGGTAGAGGATCCTCACCATCCCTCACTTCCTGCATATATACATAAACTTCTAACCTTTTAATCATTTGAAGTTTGAGCCATGGAAATTGAGAAAACAAATGAGTGTGATTATACATGGAGGTATGCATATTGCTTTATTGCTCGACTGAAATCTTCTATTATCAAGTTGTAAACAGTGGTATATATTGTTGCAGTTTATAATTTGCCAGTGAGAGTTTGTAATTCCGCTTCTATACATGTCTTA

>Gm13ciRNA1199

GTTTTCCTCTTCCCTTGTTTTGTTTCCATGTGAATGCAATTGTGATTGGTATTTCTCTACCTATTCTTTAAAACACGGCTTAAATTTAATCTATGTTTTTCTAGGCGTCATTTTTTATACCTCCATTGTTTATAAAAGGGTTAATATATCTGGAGTTGATTCATGATTATTGATGGGCCCATAGAATTAAAAGTAAAACTATTGTCTTAATGGTCAAGAAAATACATTAGAATCTTTGTTCATGGAATAATTTTCATTGTAAGCCTGTGTTTGATCGGTTAGCTCCACGTTTAATTTAATGTCAATTGCTGTAATAACCTTAATTTTTGGTGGTTGTTGGACGTCGTTTCAGTGCTTGAAGATTATAGGCATTTCTTTGCCGTCATTGTGCCAAGGGAATTTTTACCTAGTGTGTTAATTGAAGCATTTTGCTTATTTGCTAATAAGAGGTTTCTATCCCATAATCCAATCCGTTCCTTTCTTTTTTCACTAGTTTCGGTAGTGTTAATTCATTTGTTTGTCAAATGTTAATGCCTTATGGGTATCAAACTTTAAACAAATTTTATTTTGGTAGGGAAATTCTAATCATGCCATGCAAATTCTTGGTCGTATCATTTTCTAATATTTTGCAGATGACAGTCTTACATGATATTCCATGATG

>Gm06ciRNA12

GTATGATTCTGTTCTTGTTCATTGTATGCTTTGTGTCTAGATCAGACAACAACAGTTACCTAGTTTGCATCAGATAATGGAGGAAATCTTGCATACTCCAGAGTATTTCACGTAGAAAATTAGAATAAGACGGTGGCACAGAAATTGAATCTCTGCTGTCCGATTAACATTTGTTGGGACTATATGGGCTTGGTTCCTAACAACGTAAAAACAATCTGGACTGGAATACCCTCAGAGTATGCAAGAATCTCCAGATATTTTATCAATGTGGTCGTTTTATGTTATTTTGTATATTGTGGCCTTCCCCCTTTCCTTTCCTTTCCTTTTTTTCCCCTTGATGCTTTCCTACCGTCGATGGTAGCTATCTGCATACCCAACTGAAAAGGGGGGAATAAAAGACCAGGAGACTTA

>Gm11ciRNA120

CAGGAATCAGCAATGGTGAATGGTGAAAAACTTACGAAAGACTCAAACAGACGGAACGGAACGGAACGTTTACACATATTTTAGAGTCGAATAAAAGGAACAAAGTACAATTGTTACAACTAATGAATATGAAAAATCAACTTCCTATAGAAGTCATCAATGATCGATGTGAAAACCAAAATTCACATTAATATTATACTAGTAAACACACATGCTGTTTCCTACCCTTCTTAGAAACCACAAAACCATCGATATGAGACATTATACTAGTAATATTAACGTAAAACTACCTACATCATATTGACAGATACTAGCCCACTATAAATTATTAACTATATATAATGTACGGAATGAGATCTGGAAGTAACGTATTAATTATCACACCAACCTAAGTGCCACTACTTAGGTTTTAATGTTACCGTTGTACTTTTTTAACCTTCGTTAAGACATAACGAAACGGAAGTTGTACCTCTTAGACTTAACTAAAACAATTTTCACAATGCTATTTACCACAAACATTAATACGGTTCGAGAATAAAACAAAAGAAACTTACTATCTAGTCAGGAGCTCCAACAGGACACCGAATAAAGTTAAATAATGTCTGTGGTAAGATCAACGTCTATTCTTGATAAAAGGTCAATTAACCGTTTGGACAACCCACTTTCACCTTGATAAACCTGATAAGTACTACTATCACTATCAGGACAACCAACATAAAAATAGTGATTACTTAACAACTAAACTCACAGAATTAAACTCACAGACGAAATGAAAATCTGGATAAAAAAGGAAGAGTCAAGAATGTTTAAAATAACTAAAGAGTGAGTAATAAAAAAGTAGTACAACATTACAAGTTATACCATAC

>Gm13ciRNA1200

GTAATGTACACATAGAAAATTGTTTATCACTCCTATTTGTTAGCAAGCAACTTTTCTGATATTTTGTTTCCTTGGGGGATGCTGGCATAGGTAAAAATTCCCCCTTCATAGAGACTAATTTTGTATGCAATCCTTTGTTTAAACTCTCTGTTAGAATAAGCAGAGTATGTTGATAGATAAAGAGCAAAGTTATGTTATTATTTAAAATTCAATTGTATTTTGTTTAAGGGTGCACTTTTCACAAACATGTAAGACAAGGATCACCAGTATAATTTTTAAAATTTATTTTAAATACAATTGGTTATTATTGGAGTATAAATTACAATTTGTCTATCTTAGGAGGTATGAAGTCAGCTAACTGGGAAACATAACAAACCACAAACATTTCATTTTTTTTTCTTGCATGAACACCAGTAGTACTTAGTAATTTCTATTTCCTTATCCTTTTCATTCCAACATTTTTCTAGGTGCAACTAAGTTTTCTCTGCAACCTTTGTACACTTGATTCTATGATCCCAGATATAATGTATAATTTCTAACACTTAGATTTGGCTGATGAATTGATTGAGTCAGCACACTCAAAGCCACTAACAGTTTGCACTAAGACTTGTTGAATTGCTTTTCTAGTTTAATACTTTAAATTTAGTTTCAGTGCAGTGCCTTTATCACAGGGATGAATCAAATATATGATTTCA

>Gm13ciRNA1201

GTTTTTTTTTTGTCTCTCCAATGCTACTTTATATTTATTAATCAAACCCCTTGGCGATTTTTGCGAGGCACTGAAAGGAGGATATGGTGTTTCCAATGTCTGATTATAAATTAGTATACTTTGCTTGCTTGGGATTTAGAAATTTGAACACTTTATTTTCTATTTTTCAACAATTAGGACGTGCCTGCCTTGGTGTGGGCTTAGATTTCTTTGATTTGTATGATCTAGCTTACATTTAGGATTGTGTGCTAAAGTGAAAGCTACATGGTTTTGATGATGGGTCTGATTTTGGCTCATGTTTCTGCACCCTTTGTA

>Gm13ciRNA1202

CAATTCATTGTCGCGTAACTAGGTACGTGATCAGCTAACATAACAACTGTTAAACAATGGTTTAATTTAAAGTGAACAGTACTAAAGAATTACGTATATATATGTGTAATGAATTAACTAAAAAGTTCTTATTTTTTACGTTGCTCTGGGTACTTGCTTAGTTTTGAGTTTTCTGTGCACAAAGTTAAGTAGAACAGTCTGTCTTGACTAGTAATAACAACTTCGTATATGAATATTGAATTAATCATTTAATTCAAAACCTGTAACAAGAAAAGTGGGAAAAGATCGTCGTTGAGGTAGCGGCCAAAAAGACGTACTTCTAACTAACTAATAATATGTGTATTTTTTCACACGCGTTGAAAGCAAATGAATTCAATCTTAGTGAAATATGAACTTCAATCTTGGGGGTTCTTAAAATAGAAGTTTTTCCACGTAAACTCTATTGATTTAGAATTAGATTGTCGCATGTATAAGGAAATTTGTGGAATAGTTACCTAACATAAGAGAGTTACGACTAATATATACTTGTTCAAATCTATTATATTAATTGGGTTTGTATACGTTTTAGTTGTAGAGTTTATAAAAGTAGTATATCTAAACTATTCGTATTTGATATTTATATATATATGTTGTCGTGGTATATATAGATCCTTTATTAACTTAATATAAAT

>Gm13ciRNA1203

GTTGGTATTCTTTTCAATATATTATTCATCTGGCATACATAATGAATTCTGCAAAGTGCTGTTGAAAGTTATTTATGCACATCACGTGCAACATCACATGATATGGTTGCAGTATCATTTATTATATTGGTCCTCCTCTGTCATCTGACTTGAAATTTTATACATAATAGTAATACTTTCCAATTTGTCATCATCTTTGTGTGCTTTTGAATGTGCTCA

>Gm13ciRNA1204

CATACATTTTAACGATGATACGTAAAAAGACACAGAAAATAGAAACCAGTTAACTACCAAGATTCCACTAGTTTAAACACTAGGATGGATGATGGAACCTAGTATGTCTTCCATCTGTGTTTAACACACTAGTTTAGTACCTTATATTCTAGGGGAGAATAAAAAATTAAATAAAAAACCATTAACTAGGGGGAGAATAAATTTTTGATATAAAGTTTGTATATATCCATCGTATTGAAACTTTAACAATGACTGCAACCTTATTGAGAATAAGTTTTATGTAATATCTACATTCAGATTCGGGACAGACGGGTTGTTTTCTTAGTCTACCAGATTCGGGAGATTATTTTTGGTTCCAGTGTTTCAATTTCTATGTTTAGTGTTGTAATTTTGTGATTTTCAACTCCACTAACCGGAACTTATAAATAAAACACGTTCATAGAAACACTTTTCAATGGTAGAAAAAACCGGAACTTACGAG

>Gm13ciRNA1205

GTATGCCACACGACCTATTCAAAATCATGTTACTGTTACCTGAGAATGGTTCCTTCAGGACGAAAATTGTGATATATATATATATATATATACACACACACACACACACACACACTAATTGTAATGCCTGTTTGTGGGTATGCGCTGCCTCTAGGTTAGATTTTGGTGGGTTCAAGTTTCGATTTTAACATATTGATGGCTCAAAGTTGAAATGGTTTTATTGAATTACCGGCATCTTGTTGTTTCAATGCATTATAATTGGTTTTAAAGTTGAGCCCTGATGGAGTCTTTGTTCCTATTTTCCTCTTCGTGATTTATTCCGCCCATGTTTCTTTGTGTTGAAGATGTATGAAGTATCTTCTGTAATCTATTATAATTCCTGGCAGTGTTGGCATTTTTGTTGCTCTATTTATAATAGGAATTGTTTTTTAAAATTTTCAAATGGATTAGATATACATGTAAGGACTGCTTATGAAAGCACTGCTAATCAATTTAAATTGATTAATCAGAGAATTGAATATGATAGCATTGGTTATAGTTAGAAGAGATTAAGAAAAGCAACATAAACTAATAATTTGTATTAGTGTGGAAATCATGTAGAAGCTGCTGGTAAGAATTGTCAACATTATCCTACGTGTGTGTATTGGGCCACTCCTGTTTTCGAGTAGCTTTTCCTTGACGAGTTTAGGATTTTTGTAGAAGTTACTCCT

>Gm12ciRNA1206

GTGGGCTCTCATTTTATGCTTGTCTCAGAGTCACAGTTGTGCATATCCTGTTTTGGTGCAAGGCTAACTCCAATTGAGCTGATTAAAAATGCAATCATGTTTGCTATCGCAATGAAAATTGATTTAAACTGATTCTGTATAGAAGTAATTTTGATGGTAATTATAAGTGATAATGCTATGTTGTGCTTAAATTGTTAATTGTGTCATTTTCTGTTTTATAGTTTGAATCATAACATAAAACAGATTATGCTATGATATATTTTCCTCTGTTCACTCTTTCTGATTTTATTGAAGTTTTGCTTATATGCTATGATATAATGCAATCAAGTTTATTTGCACAAGAATTTGTGGTTATGTGGATATTAGCTGTTGGGACTAGTTCTTTCCTGTCATTGTTGTTTTTCTACATATATCCAAAGTGACAATACATGGCCAAGTTGAGCAAGATTTTCATGTTGAAATACTTTGTTCTTCAAGTCATAATCTAATTTATTTTTCCTTTTTTAAAGGATTAAATAACAGTGATCAAATTATGTATGAATTTTATATTTGACATTTTCCTGTGTAAAGAATCTTCTGTCATTGTATCTATACTTCTCGAGTCTT

>Gm12ciRNA1207

CATACGAATAGAAGATAAGAGAGGGAGAGAGAGAGATATATCGTAGTATTTAAGATAAGAACTTAATAATAATATATAGATATGGTCGGTCATCGATGGATCTAGAGTACAGATTTACTTCATTATATATAGACTATAATGTACAAAATTAAAGAAAGTGATCCTTTTGATCGGAGAGTAATGCGATAGACCGAAACAGTTCAAGTAGAAGGAAGAACACGA

>Gm12ciRNA1208

CATACATAAGAAACTCCAGTACTGATAAGAAGTGTAAGTCGTACTTTGTTCTGTGGTATACTCTAAACTTAATCATTTTCTACTGTAACTTTTTGTTTCTTTCTTTATTTTCTTTTCTTTTCTTTTCTTTTCCTCACCGAAACGTCATATAGTAGACACTGTACGTTCCGTTATTGTAAAGTCCAAGGTGGGTTATTCATCCAAAAAAGATGTATTGGAGACTTAAATGACCTACCACCCTGGCCTCTTATAAAGTACAAATTGTTGATGTTAGGTAGAGTGGAACTTTAGACACTTGGATCGTAAGACGTACCATAATACAGCCGTAGATCTACTATGCCGGGCTTGGACGACGGAGGAACCATAATTTTCTCCATTCGGCGTAAAGATTTCAGTTCTTGCCCGAATACCCGA

>Gm12ciRNA1209

GTAGGATTTCTCCTTTTTCTCTTTTTTCCTGTGCAGAGGCAATTTTTCTTCATTGCACTTTTCAACTCTGTGTCCTTGTTTTTTAATTTCTGGGGTGCGTTTTTTTTTGGTGTTTGGAAATCCATTCATTCCATGTTGGAGTAACCGTTGAGAGGATAATAGTTGGGTTGTTTGTCTCGACTTTTTTGTTTTCCGAGGTGGCTTAAATTTTATGAAGAAAATCTTTGCTGTAGATTTTCTTAGGATTTTATTTCAGATTCACTTTGGATGTTGTCGATACTCTATATTGCACCCCCCTTCCCACTGTTATTAATTTATCACTCATTTTAAAAAAATATTTTGTTGAGTCAAATCAAATGGAACTCTATTTTTCCGACCTTACTTCTTGCTTTTGACGATTATGCTTTGTGGAGAACATAAGAAAAAATATATGGTTTCTCTTAAAATTTTTTTACCTGGAATTAAGAAAAATATCGTCTCTATAAACTGTTTTGAGATCGTGGGGGATTGGGACCTTTCTATTTTGTATGGGATATAGATTATTTTATGTGTTACTTGATAAAGTTTTTGTGATTCATATCAATTTCTTACTGTGCAATCGGTGCCAAATCTATTGTTCGGTGAAGAAACGGTAGCATAGTCAGTCTTGCTTA

>Gm11ciRNA121

GTACGCCTTGTGTTCTGTTAAAGATCTCACATTAACTACTCATATCATCATAACAACAAGTAGGAGACACTTCTTATTTTATCAACTAATCTTTTTGAGTTTAGTTAGACCAAAATTTAAACTTTAAGATAGTATTAGAGTTTATTATACTAAATTTTAAGATGTGTAGTTTCCTCTTTATACATTTTGTGCAATTTGGACCAGATCTCCAATTTACTAAATTTTAAGATGTGTAGTCTCAGATCTGAAGAGTGACATAAGGCATTGATATGCTTAATTGACTTTAACTTCAGTCGAGGTTTTTTAGTTGACTTCAACATCATGGAATTCATATCTTGAGTGGCCACGCGTCACTATTGAGGTGGATTTGCTCATTTGGTTAGTTTTAATTTGTCCCTTCTTTTATCGAAGGGAAAAGAAAAGAACAAATTATAAATCCTTCTTTTAGAATCCGTGTGGAGTATGGACTAAGTAATCCAACAAGATATTAGCAAAAGTCATGAAATGTTAATAAAATCAAAGGGTTCGGTTGCGGTTGGAATGTATCATGCTTTGGTCGTGATGTTTATTGATTATGTCTGTGTCACAGCAAGTTAATTACTAGTATATGTAAAATGTTAATGGTGTGAATACAATATATTAAGAATACTGGGATCT

>Gm12ciRNA1210

CATTCTTTTGATGATAACGAAGTGAACAGTAAGAAAAGTTCACTGCTGTGTTCATAGAAAAAATCGTGTATGCGACAGAATTAAACGTCAACGGATTATATCATCACAGTATTCAATTCTTTCAGTTTGATCACATATGATAAAAAACCAATTTGTCAGGTTGTGGTTCCGGTGATTTTCTTAAAAGTTCATCTCTTCAACCC

>Gm12ciRNA1211

CAAGTAAAGTAAGATGTAAAAAAGTACATAAAGTATTCATTTCCAACTATTTTTTAACAAAGACAATTATTAATTTAAAGTCATGATTGGATTGTTAAGTTGGAATTGAAGTTATATAATTAGTAAACATGAGTTAGAGAATAATGTACGTCTTACACAATACAAAATCAATCGTAAAGAAAAAATTCAACTTTCACAAACTCTAGGGTATAGATGATATCTATACCAGTTTGATCACATATATTTACTTTCTGTTGAGTTGAAGAATATTCAATCAACATAATCAACTCAATCTGGGTTTGTTTTAAATATTCCGTTCAAATAAAATAATTAACTAAAGTTTTATCTAGTCAAAAGAACAACTAACGAAAAT

>Gm12ciRNA1212

CAAACTAATGAACTAAGTCTTAGGACAGAGAAAACAGACACTGGAAAGGTTGTACACCGGTTAGACAACTCAGCGTATGGGCTGACTTCTAACTTAACAGTTCGACTTAAATTGATAATTGATAAATGTCTCTTCAATAAGTAAACAAAATAGTAAGACACGTTTCCTAATCCATATGTGTGATACTAACTGGTAAACAGTCAGTAATTTATTTGAAGTCACGGATTCGTGTACGTTTACTATCAAGGA

>Gm12ciRNA1213

CACTACTATCAGTAAATGAGCGTACGTCGAAATACCCAATAGTGGATAGCTAGAATTACTGTATTAATGTCGGTAGGGAGTTATATCAAAACAAAGAACACTTGAACTAAGTCACGAGACACCTACCAAAAACAGTAAAAAGAAAAAGAATAGTACACATATTTCTCATTTTCTAATGGA

>Gm12ciRNA1214

GTATTGAAAAATATTTTTGATCACTTGCAACCTGTGTTATTCATTCATGCCTTCATGCAATTTTGTACTTGATATCTTGAATGTTAAAGTTTTTTGGGGGGCGAGGATCTATTTGAACTTCGGTAGCAAGATGTGTCTGGATTTACTGCCTGACATATGTTGCTCCACTATTCCTTTACCTCTTGAAGGGGGGTTTTCAAAATGCAATGTTAGTAAGTGATTA

>Gm12ciRNA1215

GTATTTTTTTTAATTTGTTGTTAATGCAATTTTCGCTTGTTTCTTAGATTTGTTCATATTGATTGCAGATTGTATGTGTTGTTGTTGCTTTGACGCGTGTTCTGTTTTGTGAGCCAACATGGGCTAGCTGGCTACCGCATTATTTGATCTGTCATTGGTTGTGTTTTATTTGTTTTATACTAAGATTTAAGCGTAGTATAACTGCATTTCCTGTTTTGGTTTGTGGCTTCATTCCCTGCTGCTTCATTACCCAAGACTAAATGCGTTTATGGTTCTGCTAGTTTTTGGAGAAGTAATTGTTAATTTTTGTTAGCTTCAGGAGAGAACTTTTAAAGAAAGAGCTATTGTTTTCCTAAATGTTACCTTGACTTGGGCCAATCATGAGTTTGAACACGTGATTTTCTTTGTGCTGTTTTGTTGGCTATTGTTTTGGATAGTTTTGTTGGATTTGTGATTCTGTTTCAGGGTATTTCTATTTTCTAAAATGCATGTGGAAACTGATTATCTCTTGAGAAAACCAAAAGACAATATTCTCTCACATCTTGACGTGATATTTAGTTGCTTGTATGTTATAAAAATATAAACTGTTGATTTTCCATCAATTATTGTCAATTTCATATTTCATCCTTCATATTTGAGATTCTAATTTCTTTCAGATTTTAAAGTATTTTTCCTTGATAATTTCCCTCTATGTTTGTTAGTTTAGGGGGATAATATTCATTCAACATTAAAATTAAAAGTAAACTTAGTTTTGGTGAATTTTTAGGACAGAAAGTACTGTGTATCTGGTTAGGATTCAAGAGCTGGCATCTTGTAGGACATATACATGTAATTGTTGTAGTTGAAAATTCAGATTTCTTATTTTCAGTTTGCTTTGTGGCAATGCCCTTGGACACATGTTTACTATACTTTTGTTATGCATTTCAAGTAGTTTAATCCTTGTTTACTCATTTTGAATTGGTACATTTCAAGTAGCATTTGTACCTTGTCCTACGCCTGATAGTCTGAAATTTGATGTCAGTTTGATAATGTTATTGTTGTGCACTTTTTTTGTCTTCAATTTAAGCCTCCCTTTGTAATGCACACTCATACTTACACTTGCATGCATGCAAGAATATTATGATTGTCAATGCCTGAGCACTATATGAGTAATGCTTTATCACAAAAATAAATTATATATCAAATAATATATA

>Gm12ciRNA1216

CAGACAAACAACGTCCGTAGTGTAGACTAGAGTGTGCTAAGCCTAAGCCTTAAAAGAGCTAGGGCAAGACTAGACACTAGAGACTAGACAAACGCAATGAACTAGAATACTATGAACACAATGATATACTAAGAAAATAATGAGTGCGGTTATCCATGCAAGTAGAAGAAGGAAAGTGGTTTCCGCTTCTAGCTAGCCGAAGATAAGCCAAGACCAAAACAAAAAAGCACGACAAAACGAAATCTAGA

>Gm12ciRNA1217

GTGAGTGCGGTGTGGTGGTTGGGTTGGGTTGGGCGTTAAATAAATAATGCTTTTCCGGTTTCGGTCATATGTTCGCATCAATGCATGGTTCCTGCTAGTGATGCTTTTGTCCTGTTCCTCTGTCTTTTTTTGGCGGCTATCTTGTCTTAATCTCTACATAGCAGATTCTTTCATTTGCTTTGCGTTTTTTTTTTTCTCTCCGGTGATTAGTTCTGTTGCTCGATCCATTTGTTTCACTGCATGTGTGTGGTTAGACACTTAAACTTTTTGTTTTTCTGCGGCAAATTGGTGGATTGTCTTCGATATGGTGTAATTTACTGTTCAATAAAAAAACAAAAAAACAGGGTATCAATCGGATTACAGAAAGAGACACTGTTTTCTGCAGATTTAGCCACCGATTTTTTTGATATATTCGAGGACTTTCTAGATTTATCTCCTTCACTCCCTCTTATCCTCTTTTTGTCTCTTCTTTCTTCAGGTTCGGACCTTTATTATTATTATTATTATTATTATTATTATTATTATTATTATTCGTTTCTTCGGTTCATCTATTATCTTTTGGTTTCCTGAATCCATTTCTTTCTACGGTTATCTTATCTTATTGGCTACCATCTTCCCTATCTTCCTCAACTGGGTTCTGCGTATTGTTTTAAGTTTAGGCTTGTTTCATTTTAGTATTTGATTGCTAATTTTATATTTCTGTATTCATTTGTTTATTTATTTATTTATTACTTTGTTTCATTCACGATTCTAGATGAGCTCTAAGGTTAGTGAGGGGGTTATTTCCTTTATGTTAGATTTAATCCCCTTCCCCTTTATCATGCT

>Gm12ciRNA1218

GCATGACATTATTTCACCAACCAATTGTGTGTGTGTGTGTTGCTGTACTCTTTGTGTTCATTTCTGGTTGTCTTTGAACATGCCCTAGTTCTTATGCTTTTCAAATATGCAAGCAAGGCTTGATTCTTAATGATTTTTTTTATTCCTTTTGAAGGTATCCATTACCCATTTGGAATAGTATAATTTACAGCAGTTATACACAAATGTATTTGTTTCAGAGTATCTGGTGTTACTGTTAAGTCATTAAATATGTATATGTATCAAATACATACTACTGTTCATTGCATGCTTTTATGATTTTTGTTGAGTTTAATCATGAACCTATTTAGTAATTCCT

>Gm12ciRNA1219

CATTACAAAAGATAGAGTAATGGAACGGTAAAACAAACCCACATTACATCTCAACATAAACCTCGGAACCTAACTGATCGGACCTGTAGAATTTTTACTTACACAAAGACTACTCCTACATTTACCTTCCGAAATTATTCGTATTTGTTACTATAAAAGTAGAAGGAGAAACGAGAGGACCAAAACCGATCCAAGAGGCAGACGACTGACCAAGAATTCACTGGGTTTTAGAGGACTCGTACAACATCATTCTTAAATTGAATAAACAAGAC

>Gm11ciRNA122

CATCATCGTAAAAGAGGGTGGGAATGTGAAGAGGGAGTCTTTATACACGAAATATAACTCCAAACTCAACCATAGGATCGATAGAAGGAAAATTTCGTGGTTAACACGTGGTAAATCAACAGTGAACCAGGTTTACCGAACGTCGTACGAGCACACAAACAAACTCCACAACAATACCATGTATCAAGACAAGAATAGAGAAAGACATAACATGATTTCCTAA

>Gm12ciRNA1220

CATACAATGTTTGACTGGAGAGAGTGAGTGAGGAGACAGAATGTGGTCCACGTGTTCAAAGTTTTTTTTTTCTAAAACAATCTTTTGTTTTGTCTTTTTAGTAAATAATGAAAATAAAATCGTGGAGGACGTATTATAACCCTCTACCTTCAATTATTGCGTCTGTTTTGGATCTATGTCAAGGAATTTATGAATCAAGAGATTAATTTTAACTACAAAGTGTAATCATTAATTAATAATATATATTTCTTTTATTCTATAAAACCATACATATAAAAATATAAATAAATTAAGAATATTGATGTAAGTACTGAATCATAAAATAAGGATATTAATAGAAGTGTTTGGTAAATTATTAAAAATAATAGAAAGAAAAATAAATTTTTTATACTTTTTTGTCAATCCGCGAAGTGAATAAATATATTATTTCTAACGTAATTTGTACTCTAGTATCTAATTATTCCACTTTGTGATTAAAACTATCCTCTTAATATTTTTTTTTTTTTTCTTAGAATATTTACTTAGATTCAAGACAGGATTATCATATACGGTACTACGGTATTACTTAATATCTGAGTAAAAGAA

>Gm12ciRNA1221

GTAGATATGCTTCCCTTTACTTTGCTGATTTTTTTCAAGATTCCTAAGAAATTCTTCGGTTTCTTGCGGAAGCAGATGATTATTCATCTGCTCGTAGTGAACGCATGTGCTTGTGCCGATTGATTTGTTTGCTTTTTGGATATCTGGATTTTGTTTTGTTATTTGTTCCTTCTGCTTTAATTTCTAAATGTCATTTGAATATATAGGTATTCGAGTGCCGTCGTTTTTTTCATTTTTTTGTTTATCTTTGTTTTAAAGGATTGATTCTTTAGCTTATAGTTCAGAATTCAGATCCTCTTCTGAGGAACATCCGGTATGAATAGCAAGGTTTTAAGTGGCAGTCACAGTTTTGTCGTGATCTTGTCCTTGCAGGAATTTGACGTCAATAAACTTGATGTTGCCATCAAAATTGTGGTTTGTAAAACTTTGTTGAATAGATGTTTTGGAACAATGATTTTAGCTTTAGGTGTTATGATCAAGTGGAGAAGGACTTGGATTTATATCTTAAAATATAATTAAAAAATGATTGAAATCCTAGCCTGATTTGGTGAGATGGATTGCTTCTTAGTTAAAAATTCAGATTATCAGTAAGTGGTGGATGTTGAGTAAATTTTTTTGTCATGTTTGAAGGTTTTTTGAATTGGATGGATGGTTGTTTAATTATGAGCAAGGTTTTAAATTGCTGTTGTGGTTGCGGTTTTGTTGTAATCTTTTGACATTGCTGAAAATTGTGGACAAATGTCAGTCATGCGGCGTGACTGCAATTCATGTTGCAAAAATCACGGTTGTAGGTCATTTTTTAAAACCTTGATTATGGATCCTTGTTTGTATTATAGCATTACTA

>Gm12ciRNA1222

GTATAATTTTACTGTTCTCTTCATTGCAATCATTCACAGACTGGGATTAATTTGATGGTTTATTCTCAATTGTTCTAAATCAGTCACCAATGTAGAGCAGCCAGCTGCATCATGCTGTTCAACATGACATGAATGATGCTATCATGTGCATAGTTTTTCTGTGTTTTTAGATGCGTTGTAATTTAATTAA

>Gm12ciRNA1223

GTAATTAAAGCATTTTTCAGTTTGTAGTTTATATGCTGTACTGTAGTGTTATTTCTACGTGCCTTGTTTGATCTGTTAATGATGGTTTTTGATTGCCACATTCAGGTATTTAGTTGAATGCGTGATGCATGCAATGGGGCATGCTCTTCACTTGCATCAACTATTGGTTTAATTATTTCAGTACATTGATTTTGTTCACTAACTGACTTCTTTTTCTGTCTACCCATTTCTAAATTGATGCTTGAATGCTTTATCAGTTGTCCATCATTTCTCTCA

>Gm12ciRNA1224

GTTTGAGAACCATGCAGAAATTAAACTATAAGCTAGCTACCTGCTGATCATCACTCATATTTTTAATTACTCTTTTCCTACCCTATATATAGTACTATTAATTAGTTCTTCGTCTTATTTAATTTTTTATTATTTGTGAAAATCTAATACAGCGCTCAATCAATCAAGTTAGACTCTGTAGACACCTTATTTTGTTAATTAGTTACTTCGAATTTTGTTGCTT

>Gm12ciRNA1225

GTGAGTCATCAACTCTCAATTCCTAACCCTAATTTCCCAATCCAATACCATATCTTCTTAGATCCCTAATCCGTTCTCAAATTTCGTTTATTATTTATTAATATTCTCACCGTCCAATATTCCCCCCCCCCCCCTTTCTTTTTCTAATCTTCAGAGCTTTCTCTTCGTTCTCGTTGCATATTCGATTTCGATTGTCAATTTTTATGGTTGTTTTTATTGTATATCTATATATTCTCTCTGTTCTGATTGATGTTTTAGTTGATCTGTGGTGTCTCGGGTTCGCCGTACTTCAATGCATCTATGATTTTTTTTTAAGTGATTTGGTGTTATTATTGATCAATCAAATGTTGCCAGAGTCGAAACACTGAGGGGCGGGGGTTATTTGGATTTTTGTCTAGATTTCCTGATGCTATTATTGATTATGATTATTATTCATTCACATAGTTTCCGATGCACTTTACTTTTTTTCATCGGTCACAATATTCCTTAGGTGAATGCTTTACAATCGAGAGAGACTGTGAACTTGTTGTGTCGGGTGTATGATAACTAGCTGTATTATTCATCCACATAGTTTCCGATGCACCCTTTTTCACCGGTCACAATATTCCTTAGGTGAATGCTTTACAATCTTAAGAGACTCTGAACTTGTTGTGTCGAGAGCCGTGTATGATAACTAGCTGTATTGAGGGGTATATCATA

>Gm12ciRNA1226

GTTAAGTTTTGTTCCTCTGTATTTTGCTTTCTATAATCTCTACCGTGCTTTATTTTTTTCAGCTCTTTACATCTTCACTCAAGCTTAGCGCATGTAATGACGTAAAAAGTTCATATTACTAAATTTCACTTGCCCCCAAAAGAACACAAGATATAATACACTCTTAAAATTGGGGATTTTTAATTCAAAACAGTAACTGAAAATGTTCTATCTGCTAGTTAAGAGTGGACCTACTGTTGCCGTTGTCCATGTGTACCCCTTCTGTAA

>Gm12ciRNA1227

GTGGCCTTTTTGAATTTTATTCTTGACCCGTTTCTTTTTTAAGAGTGTTTCCAGATCTGTGCATTCTGAGCTGTGGTTTTGCGTTTTGCATTAATTTGGAATCTGTCGGTGACATGATGTGGCTAATTGGATTTAGGTTGTGATTAATGATTGCCTTGTTATTATACGATGTCGACTTTGGACATTTTCACTTGCGTTTGGATTGTTTGTTAGAGGAGGTTGACGTGTTGTGATTTTGTTCTTTTGATGAATTTGGAGTTGGATTTAGGTTGTGGTTGCATTCCTTATTGGAATTTTTTTTTTCTGGTTTTGTATTGGGAGCAAGGGTTGGTTGGTTGTTTGTTGTTTCTTTCTTCTGATGGGATTGAGCT

>Gm12ciRNA1228

CATTGAAGAACAAAAGAGAGAAGGAAAGGGAAGCATAGGTAGTAAAGGAAAGATAAAAGAAGTTTAGGCTAGCTAAAGAGATGTGAAGCATAGCAGTAGCGGAGTGATCACTTGTGAGTATAGCGCTTTAGAATGCAAGATCGGAATGTGAAGATATTGTAAGCAATGAGATAATAAAGTTAAAACGTTTTAATAATGCATTTACTTCCAACAATTATGTACTAATACACAGGCACACAAAGTTAAGACATGATAAGAAAATTACAGGCTAACTTTAAAGCAAAGGAAT

>Gm12ciRNA1229

GTACGGATTCTGATTTTTGTCTGGTCAATTTTTCATGTATTTACTAGCATAAAATAATCATGTCCGCATCTGTAATTTAACTGTTTGGTTTCATTTTTTAAGAATAAAGATCAGTATTTGCTACTCATGTGTCATCACAGTGGCTGTGATTTGGACATTTGTTTATTGTGGCCTAAAACTCTGGAAATTACAATATGCTTGTCATCAATAACACATGACTAC

>Gm11ciRNA123

GTGTGCATCTTTAATCCACGCTATTTTCTGCACTTCTTCTTGTTCTTTTTTCCTGTATATTTTACTTTTTTTATGTTATATCTGCTGAATTAAAATACAAAAATCTGAGTATTTTTAATTAATAAAACTTACTTCTCTTCAGTGAAATCATGAGTGGTGTGATGACTTCATTATGCTGACCT

>Gm12ciRNA1230

CACTGTAAAAACGTAAAGACAAAGGTAATCTATAGAAATCTAACAAAGGTCTGGGTTTTTATAAGAAAATACCAGAAGTAAAACACAGAACGTGTCCACTGTACAAACGTAAAGACAAAGGTAATCTATAGAAGTCTAGCAAAGGTCTGGGTTTTTATAAGAAAATACCAGAAGTAAAACACAAATTGACTTTAGTAATAAAACTACGATTTATTACCCTAATATCTCGATGACTGAAAATATCTTAGTACGTATATAATCTGATTAGTCATATACGATGTCCTTCACATAAATCTATGTTCCACTAGAGACGTTATATATACACGATAACTAAGTAATAGTGTGAATGATAGATAATTATACTCTAAGAC

>Gm12ciRNA1231

GTAAATCCTATTCCTATTCTTTTCCCTGCAAAATGATGTCATTGATGTCTATAGCTTGTCTAAGTAACTTTCTTGTTCATTTCTTGTTGGAGACTGATTCAAATGTCTAAGTGGCCTTACTATTAATAATCTTACTGGTTCAGAACAGTGAACATCCTTTGAACATTAGCAGTATATCTAATATGAGCCTTGATATAAATTATTTGACGATTTATTTCTTGTAAATTTTAATGATTATTTTGTACAACTAAGACATCTACTGGAATTTACAAGATTTCTGGTGCATTAATGTTAAGACATGCTTGTGTGTGCCTGTTGTGATGCTCTGGCCTAAAACTTAATCTTGTGACTTGTTAATTGATATACTGTACAAATCCAATCAGTATTCTGATTCCCATATCAATGTAATATTAGTCTCTCAATTCAGTTTTTTGTACTTTCTTCCTACATTCTTTAGCCTCTTTCTTGTATCTTTATCTGCAAGTCTGCAACTGATTCTTCAGCCACTTACAAAGAATGATATGA

>Gm12ciRNA1232

GTAAGTACAATGCCTATTATTACTTAATTTGTTGTCATTCTCCTCCTGTGGTATGATTTTAACATTCTATACTCTAATGAAACATCAAGAGTTTAGGTTTATCTGCCAAACTTAAGATTGTACTCTGCAATTAACATTTATAGGAAACCTCAACAATTTTAGGGCTCAGAACTATGGAAGTTGTCACGTTAACTAATTGGTTTGCTA

>Gm12ciRNA1233

CATTCACTATATAAGAGTTACAAGACAAAGTGGACGGATCGAGAAATCATTAAATATCAATATGAATCGAAAACGAGTTAGAGTTACACCAACATAAAAGTCTGTTAACGGTAACAGGGTAATAAAATAATGTTATAATGGTACATGTTTGTGGAATATATACCAAATGTGTAAAATATTTTTTTCCAAACCTTTGACGTTACATAGAACAGAAGATTAAAAGATGTCGACGGAAAGTGAAGGACAAGAGTCAGTATAGTCGAACACGAAGTACAAAATAACTTTATAAAGGGCTATCGTTCGTGTACGAAGGTCGTAAAACAGGTTTACGATGGAGAT

>Gm12ciRNA1234

CACTCTGAAAGAAAGAAGGTATTTTTTAACCACCGTTTTACGATTGAACTGTTGAGATCTCTACCGATCCAAACACCGTTTAGATTGACTAACATTCCTAGTCTATATCACCTTCTGAAACCTGACCTATAAGAAAACACCGTCGAAATTACAGTACTTCAAGTATTGTCA

>Gm12ciRNA1235

CACCCCCTTGTAAATCAATGAACATAGTATATGGGACGTTAAATATTAATAAAAATCGTGTTTTTGGTAGCAAGGCAACGAGGGTAGGTCGGTTAAGATTATACTTGGGCTAAAAGAATAAAACGTTAAAGAAAGAAAGTTTTGGTATCTTAATAAGAAAATAATAAATAACAGTCTAAAAGAA

>Gm12ciRNA1236

CATTCGTGGGTAAGGGGAAGAATTAACTACGTAATTTCAATTTCATAAATTACATTAATACATTGAAAATATCACAATAAAATTTTAATTTTTCGCTACTTCAAGTTAATGTTATTTAACTGATCTCTCCCTTATCACGACGTGTTACCCAGACTTATTTCGACATTTTAACGGACGAAAAAAGGGATACCACCAACTCGATAGTATCTGAACCCAAAGTGTTTTTGTTTTGGGTACCTTTTCCTTTACCTTAGAACCTGTTAAGTACCCAGAAGTCGTATTATACATCCATGTAAACAAACACAATGGTACGGCTAGTAAATTTAAAACGTACGTCATGTGTAGTGTTCAACGTTCTGAAAAATTAACATGATATTTTATTTTGAACTTAGGTTCCAAAATTTATAACCTGACGGCACCGTTTGTGACAGGGTTAAACGCAATCTTTTGGAACTACAGTGCCAGGTGCAATTCTACGTTTGACAAAAAAATTTTGGAATAAATTTAGTTTAAGTTGTGTTAACTAAACTAATAT

>Gm12ciRNA1237

CATTCTTAAAAGTTAGTGTAAAGGTAAAGAAATAAACAAACTAGTTTAAAATGAAACGTTAAAACTTCAATCAAACACAACCAATCATCTCTCAACACTAACGGTACTTCTTGAGTAGTGTGGTCCAGTTCGACACTATCTTTTTTTTTTTTCACTAGTCACATCCGTTTTCAAGTTTAATCATTTATCATTATTAATCATTATTTAATACCGTATACTAATTGGTATACCAATATACCTCCGTTGACACTGCACCGAAACTTTGGTTGATAACTCATCTTGTCTTAAAAATCGTTAGAACTGTACCAAACAGTTAATCGTCGCATTGTCAACCTAATGGATCGGAAACTCGTAAAATCTACATTTTAAATGTGTTACCATAAAGAAGTTATACTTCTTTGTTTACTGATCAGTTTACACGGTTGACTAACGTAAAAAGACAAGATTTAAGATAGAGGGACGAAAAAAGGTAAGACGACAAATAAATAAATAAGAAATTAAACTAACTCACCTTAGAACTCTCGAAGTGAGTATACCTACTGTACACCTTAGAACTCTTTGTACAGAAACGGTCCAAACCGATATACTGTTCTGAATGTACGAGTATAGACTAAAACGTGCTCAACTTTTATCCGATCGGTGACAAACCAAACGTTTTATGTGTAAATTTAATCATATACTGACTACAAAAAACGGTACGAAAAGCTTAACGTATTAAGACAGTACAAATCCAGACACAGACTTTCCGTTGTCTCCCCTAATTAATGCATTTACCCATATAAATTTTTTATACGGCATTGCTCATAAAAAATTTTTTTAATGGTTTTACCTAGTTAACATGAAATTATATTAACATATAATTTTATACTAAAATGAAAATTAAATAAATTAAAAAAAGGTCATTCTTGACTTTAGCACACTATTTTATGCTAAAGTTAATAAAAATTATTATTTTTTATTTGTACAACTTTAGCACTCAACCTTATGCTAAAGTTGTAATTTAATTTTTTTTTTTTGACTTTAGCATATTATCATATCCAAAGTCACATTTTAAAAAATCTTTTAATTTTAATATGAAACTTTATGGTAAAGAAAAAGAAATTTCACTTTAACATGAATTTTCATATTAAATAGGTAAAAATATTAAAAAAATTTTAGATAGGTAATACCATTAAAAACCACATTTAAATGGGTAATACCACGTTCTTGTGTTAAATAGGTAGAAACATTAAAAAAAATTTTAGATGGGTAATACGATTAAAAGTCACATTTAAAAGGGTAATACCACGTTCTTCCGTTAAATGGTAAAAGGATTAAAAAAATTTTAGATAGGTAATACCATTAAAAGTCACATTTAAATGGGTAATATCACGTTCTCGGATTGTCTTCAGAAATCTGAAGATTGTCACAGTGAAATTGATCGGAAGTTTATTCTTGTACAGAAAGGGTCCAAACCGTTAGACTCTTCTGAATGTACGAGAATAGACTAAAACGTGTTCAACTTTATTCGGTCGTTGAGAAACCAAACGTTTTATGTGTAAATTTAATCATGTACTGACTACAAAAAAACGGTGGGAAAAACTTAACGTATTAAGACAGTACAAATCCAGACAGACTTTCCGTAGTCTTGAATTCAGAAATCTGAAGATTGTCACCGTGAAATTGATCGGAAGTTTATTCTTGTGATCTATAAAACGTTACACGTTGTATACCGTTTCTTAAAAGACAATGGGTCGGTTAAAACCAGTCCTATTTTTATAAGTAACGGATTCAATTTAGACTTTGTTGACGAACTAAAAGAGGAGTTTTGTCAAGTAGTGTGGTTGGTATCGTACCATGAAGGAAGCTGTGGGCTATTTTGACAAATATAAAGTAATGACTTATTCTAAGAATCTCGTGGTATAATAACTAACAGTTCGAAGGTAAAAGAGGTCTTCCCAT

>Gm12ciRNA1238

GTATGTGTTTAGGAATCTATTTTCCATCATTTGTCACTCACGATAAATGCTTTGCTGCCTGACTTTCATTTGGCTTTGATGATATCATATGTTTCTATTTTATGGAAATTCAATAGTTTAGGTCGTTAGAAATTTCTTAATACAAGAAATTTCAAAGGATTTTTTTTAACTGCAGTTACATGATGCAAATGATATTGTTTCTCTATTTTAAATTACTTTCGCTTTTATTGAGTGGATTCTCTATATCTTGTGCTTTTTTTACCTTCAAGTTTATGGTTAAATAATTACTGGACTCCATTTCATTATGCATAATCCTAGGTTCCAAACTCACGTATACCAGTCTACTGTAGACTGTTGATCTCAATTTTATAAATTGATATTAATACTAGTATTGTGTGTTAATAAAGGTATATACAAGGTATCTGA

>Gm12ciRNA1239

CATATATATTAAAGACATAAACTAAATAGAATCTTCTTTTACCGAGAAAAATAAATGATAACTAATTATAACTTTAGAGGAAAGCTTTTTCGAAAGGTCCCAAACCTAAAAAAGATTCAATACAGTATGAGATTTTTTCTCGTATACATGTCGAAATCAAGATATATAAAAAGTAATACACTACGTAATATAAACAAGTAAGACGAAAGGATAAAAAGACCACCGTTTAAATTTCGATATCTATAGTAAGTCATTTGATACTGTTGGGTGATATATAACTAAAACCGCAGACTTCATCCTTAGTTGGAATTCTAAGAAAGTATTATTTATACCAAACACACTCGACGACTCTCGAGTAATTACGAAGACGTTAAAATAGTAACACTACGATAAAGACGGTAATTAAACAATTTTCTTTTTCACCATATCCAAACCACCTTGAAGAACAATATTTTGTTTTTATTCTTTTTCTATACCTCCTTGAATGTTTCTTATAACACAAGAAAATTAAGATAAACCAATATGATTTTCGTATATTATTCTCATGAAAGGTAGGTGAACTGGTTTCTTGGACCTGGTAAAGTAGGGTCAGAGTCTATAAGAGAAAGTATGAAGACTACAAGAAAACCTATGTCAAACTTCGAAAACTTTCCCAAGAATCACCAATTTGAAAGTTGATCTTATAGTTTAAGCAGGTCGCTCGTATCGTCGGCCACCAAGAGTAAACTGAAACCGTACTTGAAATATTAACTAAACTATACGAATTTGTTACTACGGTGGGAATCCCCTTTCCTGAGTAGA

>Gm11ciRNA124

GTGTGCATCTTTAATCCACGCTATTTTCTGCACTTCTTCTTGTTCTTTTTTCCTGTATATTTTACTTTTTTTATGTTATATCTGCTGAATTAAAATACAAAAATCTGAGTATTTTTAATTAATAAAACTTACTTCTCTTCAGTGAAATCATGAGTGGTGTGATGACTTCATTATGCTGACC

>Gm12ciRNA1240

CATCAATTTGTTAAAATCGGTAATTTCAATTGAACAGTGGTAAATGGGCTCCGAAATCGTGGGAATAAGAACTGACTAAGTTCGTGGTAAGTCTTTTACCAGACTAAAAAATGTTCATTTAGAATACAATTTACTTATTCCGTACTGAGTTGGTATTAAACTCGTGTCAAAAGATATATAGAGACTAATCAAAACTTTAAATACCCACATGAAGAAGTAGGTCAACACTTAAAAAAAATGGAATTCGTCGTGCCGATGAGACTTATTATAAACTGCATACTCAAACGCGAATTAAAGATAAAATGATACACTAAATGTTAAAAGAAAACTTATAAACCTACAAGTAATAGAT

>Gm12ciRNA1241

GTGAGTGTCTGTTTCCTTTCATTTGTTAGTCTGTGGTTGTCATTTGATGTTGCTTGCCAGTCAGTGTTGTTTTGCTCCGAATTTGTATGTGGAAAATATATATACTGGCTTGGGCCATGGAGGAAATGAAAATTTCATTATGATTGGTTTTGGATGATCAATGCATTTCTTTTATTTAGTTGCTCTGAACACACTTCTAACACTGGCTGTTATATATTGTTCAAATCAAAATATTTGGGGAATCAAATTGATTTTAAAATTTGATTAATGTCTGATAGCAGTAGTAAATGATTGACATGAATTCTTTTCCCATCCACTAGAAGTATAGGATCCAATAAATCAAAAAGTATAATTGTGTGCATGTCCTGTTCTATAGTATCTGTAATGGGCTTGTAGTCATTTATAATATGTTTTTTTATTTGATACT

>Gm12ciRNA1242

GTAAGTCTTCCATTACCTTTCTTTTGTGTTTTTTGTCCAGCTCTTTCAATGAGGCATGCCTTTGATATTGTGTTAATTGTTATTGACCCTTTCTCCTACAAGCCTAAGCAGTCTTACAAATGGTTTTATATCTAGCAATTTCTTAACTCAGAAACGTTCTTTGAGGGATAAAAGTTCACTTAATTTTGCATAAGTAGGCTTTCATTATTTTCCATGTTTTCTTATTTGAAATGATCA

>Gm12ciRNA1243

GTGAGCATACATTTAGAACCATGCATTACTTTTTTCTTGTTAGAGGAAAACAAATTATATATTTTATATCATGCTTAATGTAAGACTAAAATGTGTTCCATTAGTATAGTTTAGTTATAGTTGATGTTAGTTAAGTCAGTTAAAAACAGTAAGAATATTATGATATTCTCGTCTGTATCTGTATTGGTATAAAGGTTTTAGAAAACAAAAAGTAAAAACTATGCATAAAGCAATTAAGAGCATTCAAAGTGTTCATAAAGTGAAATGTAAGAAATGGATGCGACATGAGACAGTTACCATTTGCTTCCTTGTTAACTTTCTTTCTATTTTCTGA

>Gm12ciRNA1244

CATACATCGAACGGTCCGGGGGTGGGGGTTAGAAACCTGACAAAATAGTACACTTGGACAATTTCTCACACTAGTAACCTATAAAGATGTCTAACTTATACGGTTCATTCGTATGACGAAACCTTATACGTTGTTAAAAAAGTTTCATTTTGTTTAAAATCCTGAACCCACATTACGGTTCGTACGAGTTATAAAAAAATAAAAACATGTCTCAACATTAACCTTTCCCTAATCAAACGAAAAATACTATAGTTAGAAAATTCGTTACGTATCTATACATAGAGAAAAAGTATAACCATCAAGTGGATGTCCATGAATTGAAAAAGAAAGAATGTCTTATCTCGAAGTAAGGGGACAAAAAAGGTGGGGATGAGGTTAGGAGGAATTACGACACAAACCAACTCCCCCCTCCCTCTCTTATTTAACACACCCAGGGTGTGGAGGATGTGAGATGAAATTAATTTTTTAAAGAGAAGTTAAAGGTAGGAGAGTTGGTTTGTGTCGTATTAACAAACTTTTTGTAATGTGGTACAAGGGGAAGTTCCCTCCAACGACATTTTCAATTTTTTCGTCCCTTCAAACACATTAAAGGACTTTGGAGTTCCACCAATTACATTAAATGAGTACGAAAAACCCCACACATCAAATGAAATGGTAATTCGAAAAGTCAACCAGACGATTTAAAAAAACACCCAACTTGCTTAAAATGAGTAATCATCGAAAAAGTCTTTTCCAAGAGACTACATTTAGGACTACTGTAAATATTGAAACCCCCAAGACTTCACTTACGGGACGACAATACAATAACTTAACATATTGTAATCTACTCCACTACTCTAATCTAAACTACAAGTATTACAGTCACTCTAGGA

>Gm12ciRNA1245

GTGAGTATTCACACTTCACTGACACGAATCAATGTCTTTTACCGACTACTTCATTTATAATGCCCCCGATCTATATTATTGTGTTTTTAGTTTGTTTGGTGTAATTTACCCTAATTTCTGCATTCACCTCTGTCGGTGTTGGGTAATGAGAATCAAAGCGAAATCATCATTTCACTTGATCATATTCATGATTATTGCCACATGCATGCCAAGCTTGGATTCA

>Gm12ciRNA1246

GTAAGTGAAGCATCTATCCAGGTTTATGTTACTTCAGCCAGCATTTGCTTGTTTGCCAAGTGATGCTTATGCACTCAGCTTTATGCCTTCCAGACGGATATGTTAAATGAGATTGAATGTAGCTACATTCTTTGAGTTGGAAATTTTCTATTCTGAAAGCTTGACATTGGCCATGCTAATTTTTTTTTGGAAGGCAAAAGATATATAAATTATTAATAATAGAAAAACTAGTACCAGTGGCCATGCTAATGATTGCTAATCTTGTGTTTGTCCTCCAAAATTTGTTTTCTGGGACATAAAAGATGTAATTGATTAAAAAGCTCAATTTAGTATCCAATTTTTGTTTGTGTTTAGATTTATTGGCTCTTTATCACCTTGCATTTTGTCGCAAATTATCTGA

>Gm12ciRNA1247

CATACGTAACTAAAAGTGCACCGACTACACTACTAAAAAAGACGACAGAGTGAACAACACTAAAATTTACCTTCCAGGACATCTAAGAAATAGATACTATATTTAATACAGATAGTTTATAATCGGTACTTGAACAACTTAGGAAAATCAAATGGGTCATCCACCCATCCGGAGACGTATATCAAAAATCTCGTTGAAGAGAAACCACAGACGAAATCTTTTTCCTAATTTCCTAACGGTACCTTCCCAACGAAGCTCTATGTTATTATATAAGGGTCGACGACATGTAGTTGTACCAACTAAACACTTACGGTCTAGGTTAGCAAAGTAAGAACATTTACAATTAACTAGAAAGAATAATATAGAACAACTCTTTAACAGGTGGGACGTAGGGCAGAGACGTAATAGTAAAGTAAAATACCACCAACGTAAAGACGATTACGTTGACTACGTTAGACTTGATTTCGACAGTAACTACAATGAAAATATGAAAGTTTAATTTAAACTAGAAAAAACACACAGATATACTATAGACGCTGGCACTCTACAATGTATACAGTCACACAAAGTGGAAGCCAGGACCTTAAATAAGT

>Gm12ciRNA1248

CATACAGAAAGAAGAAGTCCACACCTCAAGAACTTTAAAACTTACATTTACTTATTATGAAGTGTATGAATTTGAACAGTTCTTTAACTACGAAATACTACCCTCCCTTGTTGGAAGACAAAACACGATAAACGTACGTATAAATGAGAAAAGAGCCGTAACAACAAACATGAAAATTTTTATATAATTCTGACAACATGAAAGACAAATAGACGAGTTAACACAT

>Gm12ciRNA1249

CATCCACGTACCTTATCTGTTTTAAACACAAAGTTGAAAAATTGATGTTAGTTGAAGAAAGAACGGTTAAAAATATTTTTACCCCTCTCTTTCGACCTGAGACATCTACTTTTTATTAACGGGTTTATTGTACAAAACGAAATCGAAGGACCTCTCACGGGTTGTAGGGAAATACGAGGAACTCTGAAATAAAAAATGAGTTGTATGAAGTTAAAATTCAAATACCACATGATAGATATAAGAACCAACTTAGAGTGGTGAATTAAGTAGCCAACCTCTAACGTTCAGTAGAGTTGTATTCCCTACTACCGTAAAGGTAGTGTGAACTTGGTACATCTTCTGGATAAAAGGA

>Gm11ciRNA125

GTATCCTGTTATTTGCACTTATACATAAACCACATGCATCTCTGTACGAGTTATTGACAATTAATGATTAGTTTCTTTTACAATAAAGCCTATAGAACGTTTGGGCCAATATAAAAACTTTTACTTTTACTTTCACATTTTCTATCTCGTAACATGTAGGGCTAATGTCTAATGGATTACCAAATACCAAATACACTCCAAGTCCCTCGTAGGTTTTGGTGACCATTTAAATAACTTTCTTCTATCAATGCTATCAATGCCACATCCCCTGGTTTCAGTGGTTTGCTGCATGTTTCCTT

>Gm12ciRNA1250

CATACAAAGGCAAAGGTAGTGAATACGAAGAAAAAAAAAAGACTGCAATCCATTAGTATTGGTTGAGAAAGGTTTTCGTCTCTCTTTTTCCCCACTTCCTATTCTCCAAACTTATACAGATTTTTTACGCGTCACCCCCCCCCCGCCCCCCCCCCCCCCCCAATGGCGAAAGGTTTGTTTTTTTCAAAAACTTCCGGGGTTNNNNNNNNNNNNNNNNNNNNNNNNNNNNNNNNNNNNNNNNNNNNNNNNNNNNNNNNNNNNNNNNNNNNNNNNNNNNNNNNNNNNNNNNNNNNNNNNNNNNNNNNNNNNNNNNNNNNNNNNNNNNNNNNNNNNNNNNNNNNNNNNNNNNNNNNNNNNNNNNNNNNNNNNNNNNNNNNNNNNNNNNNNNNNNNNNNNNNNNNNNNNNNNNNNNNNNNNNNNNNNNNNNNNNNNNNNNNNNNNNNNNNNNNNNNNNNNNNNNNNNNNNNNNNNNNNNNNNNNNNNNNNNNNNNNNNNNNNNNNNNNNNNNNNNNNNNNNNNNNNNNNNNNNNNNNNNNNNNNNNNNNNNNNNNNNNNNNNNNNNNNNNNNNNNNNNNNNNNNNNNNNNNNNNNNNNNNNNNNNNNNNNNNNNNNNNNNNNNNNNNNNNNNNNNNNNNNNNNNNNNNNNNNNNNNNNNNNNNNNNNNNNNNNNNNNNNNNNNNNNNNNNNNNNNNNNNNNNNNNNNNNNNNNNNNNNNNNNNNNNNNNNNNNNNNNNNNNNNNNNNNNNNNNNNNNNNNNNNNNNNNNNNNNNNNNNNNNNNNNNNNNNNNNNNNNNNNNNNNNNNNNNNNNNNNNNNNNNNNNNNNNNNNNNNNNNNNNNNNNNNNNNNNNNNNNNNNNNNNNNNNNNNNNNNNNNNNNNNNNNNNNNNNNNNNNNNNNNNNNNNNNNNNNNNNNNNNNNNNNNNNNNNNNNNNNNNNNNNNNNNNNNNNNNNNNNNNNNNNNNNNNNNNNNNNNNNNNNNNNNNNNNNNNNNNNNNNNNNNNNNNNNNNNNNNNNNNNNNNNNNNNNNNNNNNNNNNNNNNNNNNNNNNNNNNNNNNNNNNNNNNNNNNNNNNNNNNNNNNNNNNNNNNNNNNNNNNNNNNNNNNNNNNNNNNNNNNNNNNNNNNNNNNNNNNNNNNNNNNNNNNNNNNNNNNNNNNNNNNNNNNNNNNNNNNNNNNNNNNNNNNNNNNNNNNNNNNNNNNNNNNNNNNNNNNNNNNNNNNNNNNNNNNNNNNNNNNNNNNNNNNNNNNNNNNNNNNNNNNNNNNNNNNNNNNNNNNNNNNNNNNNNNNNNNNNNNNNNNNNNNNNNNNNNNNNNNNNNNNNNNNNNNNNNNNNNNNNNNNNNNNNNNNNNNNNNNNNNNNNNNNNNNNNNNNNNNNNNNNNNNNNNNNNNNNNNNNNNNNNNNNNNNNNNNNNNNNNNNNNNNNNNNNNNNNNNNNNNNNNNNNNNNNNNNNNNNNNNNNNNNNNNNNNNNNNNNNNNNNNNNNNNNNNNNNNNNNNNNNNNNNNNNNNNNNNNNNNNNNNNNNNNNNNNNNNNNNNNNNNNNNNNNNNNNNNNNNNNNNNNNNNNNNNNNNNNNNNNNNNNNNNNNNNNNNNNNNNNNNNNNNNNNNNNNNNNNNNGGCAAAGGCTATGTTATTACTAAAACTCTCAGGTATTGACGACTTTTCAACTTAAACCCTAATCCAAGGAGACGGAAAAATAAACAACGACGACAGTATTAACTTTCAGGAGTACCAGAAAATAAAACCATTAGAAGTTGAGGAAGTTGAACCACTAGGAGAAT

>Gm12ciRNA1251

GTGAGTAGTCCACTCTTAAAATTGAATAAAATTGTTGCATCTAAGCCTATTTTACTGTATTTTTGTTTTTGAAGTTATGATTTCAAATTTTTTGCTCTATTTTTATGGGCTTAATAACCCTGTGCTGCATGTGGAATTGTCTTTTTTTTTTCTTCAATACTTGCAATAGGTGATTTAGTTGAACCTTCCCATCAAGACT

>Gm12ciRNA1252

CATTGTGGCAAAGCTAATGAGGCGACGAACGAGAGTTAAAGGGGGGAGAAAATAAGGCGCGAAACAGCAAAAACAAATCCCTAAGACGAACAAAGAAGGCTCTAAGAATTAAACTAGACTAAGGAGCGCTCAATAACAAACTAAAAAATACAAACGACACAAGGCTAGGAAAAAAGACACTCAACTAAGCTAACAAAACAAAAAAAACCTCGCCGAAAAAAACAAAACACAGCTAAAGT

>Gm12ciRNA1253

GTAGATGTCATGTTTTCTTGTCTTTTGATTATTATTGGTTTATATTTTCATACATTTGAGATTTACATATAAAAAGGATGACCCAATGCATGATGGGGCTCCCTTAGAGTGAGGTATGGGGAGGGTAGATGTGCGGTGCCTATATAAAATCAAATATCCTGGACTTTATTTTCCTTTGATATACCATGGTTTTTTTGTTGCCATACCATTGATCTTATAATTTCATCCATTATCACATTCTGTTAAAGATAGATATGTGGGGGCTCTATTATAAAAGGGCAAATCATCTCTTTAGAAGACAATATTACAAAGGTCAAACAATATCATGGGGCTCCTGGACTTTATTTTCCATTGGAATATTATGTTTTTTTGCCGCCATGTCATTGGTCACATTTCTTGTATGATCTATCTCAATCTAGTAGAGAAAAGGAATTATATTCTTCTTGCTTCAATTTTTGGGTGTATTTA

>Gm12ciRNA1254

GTAATTTGCTAGCTCAGTTTGGCCTGGTTTAATTTTAATGCATGTTGTTGATTTTATTTGCCAGAATTTTGGTTGGAATATGTGGTGTTTGTAATTTTGTTCCGTTATTTTATCTCTGATGATGAAAGATAAGATTCTAGTTCTTTAGAATAGCTCGTTCTTCTTGCTAATGCTTCGTTGCTG

>Gm12ciRNA1255

CAAAGGGGGAAACAAAAGAAAAGTTACAAATTAAAAAATACTTGACCTAGGACGATCATGGGTTTATACGTTTGTACAACTACTTAGGAACTTTCTGAGCTGTTTCAAGATCCAGTATAAAAAATCTCTCGATCTAAACGTATAGTTGCCTGCAGAAGGATCCATTTGACATTGTATACTACTTTAAGAAGTGTACTAAACCCCAGACACCGACGAACAAAAGAAACTAAACAGTACACAAAAATAAACCTCATTCGAACGACAATAACAGAGATTCAGGAC

>Gm12ciRNA1256

CATTACCTTATATAAGAAAGATCATGAGAACACCTTATTACAACATACAAGGTACGTCACAACTATACCAGAATATCTTTAATTAGAGAACACTAGTATTCTTTAGAGATAGAACCTACTGGTGGAATCTAGTATTTTATGACTTTCAATTTAATTATACGGTATCGAAATATCTAATCTAAACGTACTGTAAGTGTGTATAACCCGTTAGTGAAAAATTACAAGACTCAGACTTACTTTTACTAAAACCTTCTAACTACATTTTGACAAGAATAAAAAATAAAAAAATTTTAAAATAACGATAGATAAGAAACGTATTTGAATTGTGTCACTTATTATGCTACTTCAGATCACGAAATCGGTCACAATAATTTAAACACGGGGGACAGTCAAGGACTTGGGGAACTAACTCTTAGTTTACTGACGTTAATAAAATGCACTAAACTTATTCCTCAAGAGTAAGAGTTATACGTGGGAGATAACTACGTCTCCACCTGTAGTTTTAATGAATATTTTTTTGTACTTAACTATTATCTTACTCGTCACGATGAATAGTTGCCGGAAAGTGCAAAATTAAAATTAAAACTTCAAGCTACAAACTACTATGTACTCCTCGTAAATACAATGATCAAACACAAAGAGCAGTAATGTACGACTAGTACCGTACACCTATTACAAGGA

>Gm12ciRNA1257

CAACACACCGAACGAAATATTAGGTACTAGTACGTGAAGACTACACCACTAATATTTCCCACAAAGTGTAAGGATAACATAATACTATAGTAGATATAAATACATATCTTGCATACAATACACATGGACGTACACTTGTTTACCTACTATATAGTTAGTAGGTACGAACACTTTAAAAGTATTCCTTAACCATTAGAGACGAATCAGGTACGATACGTAGAATGTGGAAATTTGATAAATTGTAGTTAAATGAACAAAAAAGTGAAGAGTATATGTATTTGTTATCGAGGAGAAATGACATATCACGGTAACTAGTTTTGACTTTACTGTCTAAGCATGACGTTAAATTCGTATAAACTTAGAGGATCCTAGTTTAGTTTAACAGACAAAAAGGTGAGAACATACTTCTGTTTCTAGTATAAGAAGTAACATACAAACAGAAAGTAATTTTGACTGTAGTCCACTAACCCTAGATTTAACATTTACATTTAACTTGTGCTCGGAGGTCCCTTTATAAAATACCTTTAAGATTGCACACTGAAAAGAAACTGTTTAGTTAACTCCAAACTAACTCAGAATTTCAACAGAGTAAAGAACTGATCTATACTTCCTTAAATGCTGAAGGACCTAGACATACTACAACTTTTATCTCGGATAAATACAT

>Gm12ciRNA1258

CATTTATCGAAAGAGAAAGAGAGAAAGAGAGAGGGAGAGACACGAGGAGAGAACGAGCGAACGAAAAGCACGTAGAAAGACACACAGGGGTCCGTAGACGTACCGTACTTGTTCAGGTAAACAATTACATACAGACGTACACATAACGTAAAGGAATCAAGTACTTAAAGTCACTAGTAATAAACAATTACAACGAGGACGTAATGG

>Gm12ciRNA1259

GTAAGATAAGACCCTAGTAACAGAATTGGCTGCATTTTATAGCATGAATTGCACAAATCAACATTCCATCCTGTTGGACTCCAATCCAAGTTGAGAGGAGGATTGCTTTCAATGAGCATTGGAAAGTAGGATTTGGCTAAAACAAACACACTCTAGGCAATTTTAGCATAAGCTCCGACCATTTGCACTATTTCAGAGCCTTTTGCAAAATTATGCTGGTAGTCTCAGATTACTTGGAAATTAGACTATAAATTTGTCTCAGGATAGATTTTTTTTGCTTCTCAATAGATATATATTTCCCTGTTGTACAATGAACCTTGAGATGGTAAATCCTGTCTGCTTAAATTTGCATTTGGGTTTGGCAACTTTGCGGTGGTTGA

>Gm11ciRNA126

GTAATTTCATTTAGTAATTATTTGTCGCTTTTTCAATAGCTGACGTAGTTTTTCTTTTTTTGAAAATGTTTATTAATAATTAATTAACTAATCTGCGGAAACTCTTAGCTGTTAAAACGTGTTTTACAACAGAGGAATATGATTGTTTTGAGGAAAATGAATAAGTTGGGGCGAATGTCAATTAGGGGACCAGTTTTTGGGTGCGGTATCACCATAGACATGGTTGTCACGTTTTTTGTTTTTCAATGTCGTGTTTAAATTTTATTGCACCGTCCAGATTCCGCATATAGTGTCTTCTTATGTTGTCAATGTTTTT

>Gm12ciRNA1260

GTACCCCCTCGGGCCTTCTCCCTCTGTCTTCCTTTCAAATGGTTGTTACCATTTGGTGAAAAACTGCTCAATAAATCTGATAATGTGGCTGTTTTCATGAGACTGTATTTCTTTTTCAAGCTTACCTGTATCAGTTATAGCTTTATATGATATGTGATAAGTAGAATGACTACTACTGATGTAGTAACAAAAATGATTTTATACCTTGGAGCTGCTGGTTTGGTTAGACATCATTGTCTATTCCAATAATCTTTCTCATTTTTATATGTGGGTAGAAATTGATGTAACAGTAATAGTGCTACAGAGTTATGAGCTTCTGAGAAATTTGTCTGGATTCTGGAGTACTTTCATGAAAATTTCTGGAGGATTCCTTTCTACACAACCTAATTAGTTAGGGCTCATTCATTAACGTTTTTGTTAAGTTATGATTGGCATCAAACCACAACCATTTTACATCAATAATTTTTCACTATCTATTGTTTATTCAGTTGGGGGGCAAGTATTTTGGTAAACGCCTGGAAATGCTTCCCCTCATTAATCGCATGCTATAGAGATACATAAGTTTTGAATCATGTGAGATGTTCT

>Gm12ciRNA1261

CAATAAAAAAATGGAAGTTACATACACAACCAACCATATTACAATAAAGTGGATCTCTCTCTGTAACTTTTTGGTAGTTGTATACACCTTTGATGATCAACAGATTAAACACGTTCTTTCCATTTACTTATTAGATACTGATAACAGATACTATTGATCATAAGGACACTGACAAAGTTCACTTTCGGTAAAATAATTTTTCACTATTCGTAATTTATAAATAATGAACTTCAAGACAAATAAACGTCTTTAAACCAGTGTACTCACCAGTAGTTTCTTGTACTTGGAAAACAAAAACTATGACGTCTAACGTACACATTTAAATTCATAGAACGTACGGTTTAAGGGGTTTTCGTAACTTACTTAGAGATCCTGAGGAACACGTAGTATTTTATCTGTCGTAGTTTGAAAACACGAATGATAAGGTGAACTCAAATACTCAGTAAATAGTACAGTAACAAAAAA

>Gm12ciRNA1262

CATTCAATGAATCAAAATTCATTACGAAAGGGAATGATCGAAAAAAAAAATCACATGTTACTGCCGTGAGCCCAATCTTGAATCTTGGAGCACGTTTGATAAGTCAGTGATCCGACTGAAATCACCCAGAAAGGAACCGATCAAACGTAAACATATCATAAAACGAAGGAAAACT

>Gm12ciRNA1263

CATGGAAAACTCGGTTGTGAGTAACCAAAGAATAGAACTTTTGTTAAATCCGAATTAACGTCAAAAACAAATCCGAATTAACGTCAAAAACAGAAAATAATAACAGTAAAACATTTAAACCAAGTAGATCAAAAAAAGAAAAGTTTAAACGGGGGGAGTGATATAATTAACAAATTAAAACTATATAAGCAACTAAACTATAAATTATAAATTATAAATTATTTTGTACAACTACACTGTTACTGTACACTTATAAAAACAATTTATAACTTACATCTAACTGGTTTCAACATGTTAATTTTATCACTTTTTAGTTTAAACGGTCTCTTTTTTTTACTCTCTGGTTTAAGTCGGTTGACAAATTCAATAACTTTAATGTAGTTAATTCTCCGATCATACTTTAAAGTTATTTTTAACTTCGGGAAACTATATATAACTTCCTTTTCATTACCTGATTATCATAATGAATACTCTAGGTA

>Gm12ciRNA1264

GTAAGTAGTTGCACTTTCTCCCTTGCCTACCTCTTAGATTCTTTGAATGTGACTTTAGGCCTAACTCAATCCCAAAAGCTAACTCATTGGTTAAGGTTTGTCCCTCACTTATATAATCCATCTTGGTTTTATCTCTAGTCAATATGGGACTTGGATTTTTCCCAATACTATCCTCTCTATCTTGGTAGAGTAATGATATTTGTCCTGCTGGGTTATGTCCTTGCCTACCCTCTCTATACGTGTAATTAACCATGTGTCCAGGTGGATGTTGGGTGGACATTAGTCTATTTCCATCTTGATATTCGCTGCTTGGTTATAGTTTTGTCTTTTGCTTGCTTGCTT

>Gm12ciRNA1265

CATTAGTGAGAATAAGAATTATAAGGAGTACAAAATAGGTACTAAGGTTAAAATAATAGGTTAAAGAACAAAATACACAAAATTAATCAAAATAATTCACAATATAATATAACGATAGCAAACGAGATTGTCTTATTTATTTTTTCAACGTAGAAACAATACTTATTATATTGCCCATACTACTTATCGAGTTAGTTATAGAATTAAAATCAAAAATATAAATTGAACCATAATATACTATTTTTTATTCGGGCTTACTCGATACACCTCCGATGACTTCCACGTAAGAGGTGTACCAACCCACATAACCCGA

>Gm12ciRNA1266

GTATGCATGCATGACCAAGTAATCGTGATATATTTATTTAGTTTCATACTTTCCTTTAAGCATTTCCTCGATTAGTTTTGTTCACATATATGAACCAGTTTTCAAGAACAAAGTTACCCCTTCTGATCCTCATGCACCCCTCTTCAAAAGCATATATTAATTTAACTTAGTAAAAAATCATGTTGTTTTTTGTATCTAACTACACTGTACACCCCCTCCTCTCTCTCTCTCTTGAAACCTTGTAGTTGTACCAAACAAAATGGTGCAGAACTCATGAACTACCTATCTATTACCTCCCTATTTGAAAGATCTAATGAAACAGCATGTCCTTTGATAATTTCCCTCTTCTTTTTCTTCTTATTTATTTAATGTTCTTTGTTATTTTTCTTATGTGTGTATGTTTGTGTGTGTGTGCGTGGAGCGTATTAGATGATCAAGAACATGAAAAAAACTGTAATTAATCTTCATTATATGATCATGCATATAGCTAGATAGAATAAACAGTATATACACCACTAATAATGGGATTTTTTTTTTACCATAACATCTGACTATAAATCATATAAATTTATTTATTTATACAATAATTATCTTAAAAATCATAATATAATGTATAAAAGTTAAACTCAAATAATTAAATTAATTATTTTTTCTCACATTCACATCATCAAAATGGAAAGATGTTTTTTTTTTTTCATTTTTCATTTCTTTAGCATCTCCACATATTTGGTACGGTGTTTACACGCAGCTGCTATA

>Gm12ciRNA1267

CATACAACCGTAGCAAGAACGTAAGAAAAAAAGGGAGAGAGAAGGTAGAGAATTTCCTGAAAATAATTTCTTATTACGAAGAAAATAATTTAGTTTAGTTTGGGTTTGAGGTTGACACAGTTTGACCTTTTATTAAGTGTACGTCAGTATTGAGTTTCTAATACATGAAGGACATAAACAGTTACTTTACATAAATAGTGATTTATTACGAATACGATTTTGAACTTTCGGGTACTTCCGTGACTGTACGAAGTTTCACGTTTGGTTCAAAAGACTTTATACATACTGACTTCGATGTAATGTCAACGA

>Gm12ciRNA1268

GTTTGTTATTTCTTCATAGACATAGTAAAGCAGTAATCAGTTGTCATTTAAAGGGATGCTTGAATAAATCATGATATTGCGCATCAAAATAATGTTTGAATATTTAAAGGGAATTTTTTCTCTCGGAAGAATTACATTACAATTTATGATATGAGAATTAACATACTTTCATTTTAGGATGATTAACCAATGCCGACTACAATTTAGGATATGCTAGTTGACATATTTTCGGTTTTGGATGATTAACTAAGCAAGTGATTAAGCACATTAATGCATGGTTTAACACTTGACTCAATTAATCATTCTTTTGGTTATTTCATCCAAAATTGTAATTCGCTTGTGTTCCTCCTAGAAATTACTTTCATAACTGTTGGAAAATAGGAGCTTTTTAGAAAATAAAGAGCATTTTTTTATCATTGCTTCTCTTTCCTTGATATTTTGTATAGCTAGGTTCACTGCATCTTAGGAAGTATTGATAACTTAATTGCTCTCTTGTAAATTGTAGTTGGAGATTGATTATCTTTGTAATACTTTGTGAATATAAATATAATAGATCATTCAGCTGAGTGGTGAAATACACTCAAAACACATCCCGAAAAACCTTGTTTCTCATTTTGTCATGATAATCAAGCTCGCGGTTGTCTTGTGGCGACATAATATTCCAACTT

>Gm12ciRNA1269

GTAAAAGTCTCATTTGATTCTACTGTTACTCTAGATTACTAATCATATGCAAGGTTTTAAAAGATGGTTTGCGACCATGATTTTAGTCACCACATCAAAGTTTTTGGGATCTGTGAACTGCAATTGTGACCGCATTTGTGTACAATTTCCCACAATATCAAAAATCGCAACAAAACCATGACTGCAATTTATAATCTTGATCATATGATTGCATTGCCATTTAATGAAGTAGCTACTGGTGAACTGCTTCTT

>Gm11ciRNA127

CATTCATAAAAAAAAAAGAAGTTACGGAATTAAAACCTCAGCTTTTGTTCGTAGGACCAAACAAACTATCAGATTTAGTTCAAAATAAAAAAGGTGTAGTTCTGCAGAAAGTGATGATCGACATATGTTTGATGTAACTGATAAAGTCGGAAGTACAAGGAGAAAGTTACGAAATCTTTGTTACCTTATTCCAACGAGATTTAATTGAACAAAAATTTCAGTCAAAACAATAAGTTAAACTTCACGTTGTTCTAGAATTTGGTAACTTTAAATACCTACTCATGTTGGAATGGATGAGTGAAATTTCTACTATATCTTCTTAAAGAAGAAGAAAAAAAGAACCGTAAGTACAAAGGATTCTTATCAAACTATGGAATGGTAGAACTTTTTTAACTCGAGTTTCTGACCATATCACT

>Gm12ciRNA1270

CGTTACATGATACTACAATGTTAGTAAAATCGACTTCGGTAAACGGAAAAAGAAAATGAAGACAATCGTATGATCTGATCTGACTAATCGTATACATCGTCTACAATTAATCTTGAATCTTATAGCTAAAATCAATAACTTATTGATAATTAATCGAAACCCAATAAATCAAGAGACAATCAAGTATATAGATCAGAAACAAAACTATTCGAATCAACCCGAATAGACACCGTTATTCATTAATGAGCACTTCGTAAAAACAAGGTAGACTTACTCGTATAATTTTGTCGAAAACTCCGGGTACATATGTCAATTCGTCAATACCCGAACCGAAATTTTCGATCTTTAACTTCGATATGTTTGTTAGGAAACACTTTCGAATTTTGTCGATAACGTCATAAACAAAAGTAGGTTCGTGAGTAGTCAAATAAAATAGATTTTAGAGTACTAACGTAATTGATGAATAACTTCACTACCAGGTTTGACGCAAATAGTATAAAATCTAACAGATGAGTTTACTTATGAATAAATTCGAACAAATAGTTCCATTTTAAAATTACACCAGTACCATATTCGTACAGTTCCTGGTTGAGAGGCTTTTTAAATTCGACATACCATAACCGGGTTAGTAACAAATATTGAGATTATACGACGGAGTGTATTCTCGGGAACCCGGACTTTACATCTGCTACTTGTATGGTGTAATGGAACACAACTAAATTTTACTACTTATCCCGTCCGTTCCTAACAATTGAGACCATAAAACAGTTCCTAGTTGATAGGATTTTCGAATTACCACCGGGTTCTTACCAAAATATAGAGGTTACAAGTCATCGATCACAACAGAAGGAGGTAGACGAGACGAAAGAAGGTAATCACAAGAAAAAGAAGTGATCTTGAATATCGAAAGAGACCGGATAATGAAGTCATAGTAGACAAAGACATCACCTGATATCATATAATTGACCACTTAAGTACGAAATAGGACTTTGGTTACGTAAAAGAGTATTAAACAGGGATCGAACTGTTAATTGTCTGAG

>Gm12ciRNA1271

CAAGGGGTGAATGTAAAGAGTGAAATTGACATTGATTTAAGTACAACATAATAACCGATCTGCTGGGAACGGTAACGTGTTACGGTTAAGGGTGAGTAAGGCACATAGGTAAACGAAAGTTTGGGAAATAATACAGTGAAATACCTACGTTCCATTACAAAAAAACATTAACTCTACCTAACTAGAAGCGATAGCACGCACAGATGGCGACGACAAGGAAGACCTGGCTGATTATATGACATGTAAATTCTTGTTGATTCCGTACTCCGTAGTTATGGCACAATTCTGAATTCTTGGTTAACAGAGTTTCCGAATGTGACAATTCACAATTCGATACTTCGTACCTAAGTACTTAATAAACACATTAACAGAGTTTTAGAAAAATTTGGTTGGAAGATATAAAAAAGTTCACATAATCAACACATTTACACTGTCGAATTCACAAGTTCGGTTGTTTTTCATTAAAAACTTGGTCTGTGAATTATTCTTCACAGTTTTTCCACAGGTCCACACGGCCACAGTCTTTGCAGAGACTGTGCCGCTGTGAAACTTGTTCTCTCTAAAAGTACGAAGTATCCACAATTCACTTCGGTACACTATACTTAGTTACTGAGATAATGTGGAGAATAGTTATATATAGTTTACTGAGAAGCTTTTAAAGTGGTTCGACAAACTTAAGAGAATGATATTAAATTGTGACCAAAAAGTAACGAAGTATGTAAAATTCGAGATCTTAATGAAGTAGATGATAACTTATGTACGTGACTACTACTGAAAGTATTGAAAAGTAAATTACAATTATTCAGAAGTAAAAAAATTTGGTAACTTACATTAACACTATTTACAAGCTTAATTAGAGTCGATATTTAAAATGTAACCTCACACACAGTGTCACCTTTTAAACTAAAACACAAAAGGTTTACCTGTCATTCAGCTTAGGGTTACATCTTACACGACTTAAG

>Gm12ciRNA1272

CACTATTAGAAAGTGTAATGAATCTGAGTTCCAAAATTTAACGGTGTTACCAACATCAAAAATACTATAATGCCCTTTACACCGCGGTTTTAGAACTACGGGGGTACATCGTATTGTGGGCTCCACTACGGGTTTTTCAGAACTATGACGGCGGTTTTAGAACGAAACTGAATTAATAAAGGGGGAATTAAGAAAGATCTAAATTAACGTTTAAAAACGGGGGTTCAAAACGTTACAACGTTTAAAACGGAGGGTTAAATGTTTTATAGCGTACAATACGAGGGTGCATCTCTTCAGCGGTGTTAATTATATAAAGTACCGCCAAAAAGGAAATTTGACGGTGTTGATTCTGTTAAATATTAAAAAAAGTAATTAACACCGCCAAAAAGATGTACACCCGTTTTAAACGTTGTAACATTTGGAAACCCCCGTTTTTGACGTTAATTCGGAAGAAAAATAATACTGTGAAGAAAAAATATTTTCTTTAAGACTGTTTATTTATTACAATCACGTTCCAAAATTTAACACCAACACCAAAGTAGTACAAAGAATTATAATAGCCCGTTTACACCAACAACGTCAGTTTTAACGTCAACACAACACCAACACTTATGGCGTTTTTGGAACTGTACAGACTATAGCACCAAAACCTGACAAAGAGTTTCGGAACACGGTCAATCACCGTAGATACATAAAGATTTACAATGAAAGTATCAAAGCATTAACTTTTTTATAAAAACCGAGAAACTTTAAGAACGACTTCAAAGTAAAACGTGGTTAGTGACAACTATGTCTAATGAATAACCCAACTATCAATTGGACAGTGTGAGTAGGAAAAGATAAAGTGGCAACACTTTAAATTCTAACCGAGT

>Gm11ciRNA1273

CATACGTTCGTATTATCTCCGTAATATCACGAAAGAACAATAAAGAAAGGAGAACAAAAGACACACCGGATCGGATTCTTCCTAAATACTAGGAGGACTAAACATGAGAGACCAAAGAATTACTTATGAAAATACTTTTCCTTTTTTTTTTCTTCTTTCTGAACAACATAGACAAGACAAATCAAATGAAAGAAGGACGTGTAATAACCTTAAACACGCGTCCGGGACATATACTCACTCCTTCTCACGCAATAATCCGAAATCGAACTCACCGAACAAACAAGCTTTTACGGAAACGTGAAACCTGAGTAACGAGAGAACGACTATATAGAGAACACAGATTGGGTGGCTACACCGTGAACAAGAGGTTCTGATGGGGAGATCAGGATGACCAAACTGGGTCAACACTGAACAAAAATTTAAAGACGAACCCAAATAAGAAGTTCAAACCCGGAGCCACCGGAATCCAGAACAAAAGTAAATAACGGATCCAAACACGAGGAAAAAGAACTTAAAATCTCCGGTGTTAACACAAAAGTAAATGTTACGGACAAAAAAATATTTTAAAAATCCGAAAAAAAAACCAAACCCGAAATCCATAAGGGAACCTATATCCGAGACTAGGTATAATCTCAAACACACCCCCCAGGATCAGGTTGTCACTCCTTTCTCACACAATCATCCACAACCTCTAGGGTGTAGTTGATCTCTATACCGGTTAATTATAATCTCGCATAGGATCGTTCATAACAACCCGGATAACACAGTGAGCGATAACCCGACGATAACTTTCTACAGGTCAGGACGTTTGAAGTACAAGTTCTACAGGTGAGGAGCCGCACTTCCCTCACACAACCTCTAGGACGTAACTGAACACTATTTCGGTTTTATAACATATATTCACCCCCGTTGGGGGTGGAATATTCGGCTAAAACATCTAAACTCAATCCGAGTTTGGGTGTAAGATTCTCCTCCGAAATCGAACTCGCTGGACAAACAGGTTTTTACGGAATCGTGAAACCTGAGTAACGGGAAAACGAATATATAAACAGATTGGGTGGTCACACCATGAACAAGAGGATGTGTGTAATTATGATCATAAACTAAATTCCTAGAATGATCAAACTAAAGTAATAGAACGTTACTCAAAGTCTCAAACTCAACACTTTGACTTCTTATAATTCAAAAGACAAAGTAATCAACCTACAACAACTTCATGTCAGTCAAGGTATACAATGA

>Gm11ciRNA1274

GTAAGAGCTAGTTAAAACTTTATAAATGAACCATAATACCCTATTAATGATTCATGAGATCTCTCGTTTAATTATGTGGATGAAAGCCATGTCTGATTCTGTATGTTATAGTTTTCAGCATTTATAAAGCTGTAAATTGATAAATGTGTTCCGTTTTGTTGTTTTAACTTTTAATGATATCTGAATTACTACATGATGTTGACTTCTTGATAATTTAAGTTGGTAGAAATGGAAATGATTAGTTTAGGTTTAATTGCACATTTGCACTTTCGGTCCCTCTAATTTGGACTACCTCTGATTTTGGTCTCCCAAGTTTTTTTAAGCTAATTAAGTCCTTTTATGTTTAAAATTAAGCAAATTTGATACCCTTGTTGATTTTCTTCTACATAAAGCCATCGAATTATTAATTTTAATTCAAATATTTTGGGGTTTATGTTATAGTTGAAATAGGAAATAATCAAGTTTAGAAGTAAAAAAATTATTGGAAACTAGATAAATTTGAAAGGTTTTACAAGATGTGAAATTAGGGTTTGAAGAGGGGGAATTTGATGTTTTAGTTGGTGAATTTTAAGCAGAAAACTGAAAAATAAAGAAGCTAAATTTTAAAAATGGTAGTTTGAGGTGCCACCCCACTTTCTAATCAATGTGTAAAATTGAGTAAAAAAAGTGAGAAATTGTGGGTTTTTGGTATTAGAAAATCGACAAAGGAGGGATCAAATTTGCTAAATTTTAAAAATCATAGGGGACTAAAATAACTCAGTCGAAACTAAAGGGAGGGAATGCAATTAAGCCATTAGTTTATAAGCATTGTTTTACTGACTGTGGTATGTTTGGGTTACTTCAAAATTTGAAATTGCTGATAGAATGATGCACCATGTACTTCAGCATTGTGCTCAAAGTACATTAAATTAACAAATCAAAGTATACTCTTCCGGCATACTGTTTTTTTCTGAGATGCATAATTTCTAGCA

>Gm11ciRNA1275

GTCGATTTTTTTTTCTATGATCCTCCACTTCACTAATCAAACTATTGTTTGCTAATTACGCATCTGAATTAGGATTTATTTGCCACTAATCAATCCTAATCCATCAATATCAGTGATTGTTTAGTTCTGTTTTTTTTTCTTTTCGCATGACGGCTTTCGGAGTTGGAACGGCGACGTTTGAGTCAGGGTCAATGCAAAGCCCCAATTTAGATTATTATTAATTTATTATTGGTAATTAAATATTAAATATAGTCAGAATGTCATTTTCTTTATATATTCACTTAGGCACTGATATTTCACGCAAAAAGTAGTAAATTTTCTCGATTTTGTGATCCTCCTACTTGGTTCCGAGAAAGGTGATGTCAAGAAAATGTTGAAATATAGTATTTCTTTCTAGCCAGACAAGATTGAAATTTTATTACAATTGAATTTTCTTCTTCTTCTTACTGGGTGTGTGGGTTTTGGTAGCTTTGTGAGGGATATGGAATTTGTAGGTTCTATGCTA

>Gm11ciRNA1276

CATCGTACATTAAGTATACTCACCTAAACGGAACAAGGACTTAAACTAATTTCCTAACATAGACTATATAGAATTGTGACTACAAAATACAACAAAATTAACAAAAACCAGTTAATTCGTATGTTAAGTTAATAAAAACCTCTAAGATGTTTAAAAATTAGCTGAAAGTTCGTCACATTGTAACTCATAATCGAAAAGTATCAAAAAGATAAGAATCAAAAACGGTCAACCATACGTCAACCTCGATACATAACTTAAACTATCGAACCTATTCATCTGTAGTTTCGTAGTAAAAATAAATATAAAATGGGAACAACCTACGACTCAAACGACCCATTAAGTCCCATCCATGAACGTAAATTGTAACACGTATATTATTTAATTATAAAACTGAGACAACGAAAATACTATGAACAGTATAGAAGATTCTATGTGAAACCTATACAGTTCTAAGACACCCTGGAAGTTTTCTACAGAAAAACCTTTTTAGACACGATGTAATATATTTATTTTATTTTAATTTGAAGAGGAAGTCTGTCGTTCTTACAGAGATAATAACAACTACAAAAAAACATAATAAAAAATCTATAAGTAACTAAGTTAAAATTACAAATAATGTAATAAATTCATTAACTAAACCTTAGGTGAAACCCAAGCTTTTTGAATGATGATAATGGTTTACTAAGTCACAAGATTAACTATAATAAAAATATTAAATACTTACTCACCTCCGAACCCGA

>Gm11ciRNA1277

GTAAGTAAGACAACAACAAAATTTCTATTTCATCATTGAATACAAAAATAACAAAAAGAACATTCTCCCATTAAACATGTGTAGTGTATCCAACCTTAGTATGGAATATTGAGACACCAGAGATACTATGTAAACATCAGCCTTGGCACACTATGGGGTATGGAGGCAACAAACAGAACAGAAAAAAAAAACTATATGATCACTGAGATAAGCGTAGCCCATTCTAATCATTATATACTTCTGC

>Gm11ciRNA1278

CAAACAGGGAAAAGAAAGGAAGCGAAGATTTAAAGGGGATTGTTAAGTAAGTTATAAAAAGCTTAGAAAAAAAAAAAGCGAAGCTTAACAATAATAAATCAAGTTAGAAGGACAATAACACGGAGAAAAAAAAAAAAGTAAAGAATATTAAAAGGTGACGAAAAGCTAAAGCGAAAGAGGCTTTTTACGTTAAATACTTTGAGTCTCAACTAAATTATCGAGAAACAAGTATTTTACAACCCTAACAGTTTCCTAAAAACGACACGTAATAGGCCTCTTTACATGCACCTCTAATAACTCCCAAGGAACTAAGGTACTTGTTTCATCTAAATAGATAACTATGAGCTCCCAACACGTAAAACACGACACGACACGA

>Gm11ciRNA1279

GTACTTACTATGAGCTTCTTAATTTTTTTCCCTTTACACCCAATTATTGCTGGTCTTCTTTATTTTCCCTGTTTTGGTCCTTTAATTCAGATCTGTTATTCTCTGAAGATGAAGGAAAAATTATTTTGATCTTATAGCTTTCTCAATCGTTCATGGTTTTTGGTAGTGAATCTTTACTTCCTTGCTATAGAAGCCACCTTTAACCAACAAATTAAATTCATACTGTGCTTTTTGACTTTTTGGCTAAACAGATTATAAGTTTTAGGTATGTTGCATGGGTCATTTTA

>Gm11ciRNA128

CATCCGGATTTAGTTTAGTTGAAATCAAAATCTATTCAAAATAAAATAGGAAAAGAAAGAACGAAACGCTCACCAAAACCAAAACCAAAACCAGGCAGGGGTGAAGGGTGGATTGCCGGTCGCGGTGGCCGGGTGGGTGGTGGATATCCCTTGTTAGTTACACCTGGTTGCAAATTGATACCTTAGGGATCCTGTTAGTACACGGTGAAATCAATGTCATTGCAAGATTACACAAATGATATATTATTACATATTCCAGTCTATAAAGTACTGGGTTATAATTAAAATAATAATACAAATAATACACCGGAGTCGATTCACCTTCATTAATGATTAATATATTCCAGTTCCAAAAGTCAGTTTCCTTTTTTTTTATCTATTCCAGTTCTAACTTTACTTACAAGTACGTAGTATAAACGTATTAAAAGATAGTGGCTTATGA

>Gm11ciRNA1280

GTAAGAAAATATTTTTCTCCCTGATTGTAATTTGTGTACAACTTTCACGATTTCATGATTATTCAAAGAAAGAGTTCCAAAGGGTTTACAATTTAAATTATGGTTTTAATGACAATCATTTGGCGTTAATTGTTCGATAGTAAATAAGAACTGAAGATTCCAATTATACGGAAGTGGGATCTGAATGATGGGATTCGAAATTACACATGATGAATCTTTATTTCCCCTTTTTGAATTCGTAAACAAGTTTTTGATTCGTA

>Gm11ciRNA1281

CAAAATAAAGAACTTGAACTTAGGTTAGATAAAACAAACGTTACCACATATTAATTCAAGTTATCAAATCGACGTACAAGTTGACTTCCAACCTATTACTGGTTTTAGTAATTGATAAATATCTCTCCTGACGAATCAAACACGATAGTAAGACACGTTTTATGATCATATGTCGATCCCACACAGAAAACTGATACTTAACCACCAAACATAGATTAGTGCACCTTTATCATCCAAAAATTGGGGTGGGGGAGATGGGGGGACAAAAAATAAAAGATACCTGGTCATACCTCGACATGTAGA

>Gm11ciRNA1282

GTAAATAAAATCATCTCTTTCTTCTTCCAATTGCTTTGCCCCTTAACTCTGCCATCGCATAAAGTTCGGCTCTTTTGCGTTTCTGCTGATGAGCGTTTCTTCTTTTCATTTCTTCTCTGTGGAAGTGAATGTTTGATTGATTCTCTGGTTCAAGTGTCATTTCGCTTCGCTTTTCACGGTTTGCCCTATTTTTGTTTTTTTATGTCCTGCTGTGCTGTGTGTAGCTGTAGTCCAATTACTTTAAGTTACTGTTTATTTCCTGTTTTCTCTCGTGTGGTTTACTACGCACATTTCATACCTGTCTGCATTGTATATTGTTTTCAAGTATGTGAAGCCACATTCTTTTTGAAGAGTTCGGAGTAATTATCATAATATATCTAGGAATGATTTTTACAATAATGAAAGTCATTGATACGACATTCCATGACTGATTGACAATACTGTTGTTGTATTCTATTTTATTTTTTTTTGAATTGGGAGTTTTGTTGAACATTAACTTTTTTTGCAACATCCAATAAATTGCTCCTCTACTATACTGTTTTGAAAATTCCATGCTGTGATGCATGATCTGTGTCAATACTTAAGTTAGTCTGAAATCTGCGAGACTGAGTTACAGAATCCAAGGTTTGATTGCATTTAATTTATTTGAGCGTTACCGCGTGTTTATGATTCACCGTGTGTGCTACTATAAGTGTGCATTGATGGCAAAGCTTATGCGTTATCAACATGATTGACCAGAAGCCTTTTCAGAATGCAGAGTTGTATTTGTTAAATAGATTTGCTTCTATAACTGTGTTTCTGTGTTTAATTGAATACAGAACT

>Gm11ciRNA1283

CATTCAAGGAGTGGTATAACTATAAATCACAAAGACAACAGGACGATACAAAATTTAAATTATCCAAACCAGGAGGAACGACGATACGGAAATTAAAACTACAATTTTTTTTAAGTCAAAACAAACGAGTTCCTTTATTTTATACGTACCAACACACGGATCTACGACAGTCATGTAATTAGACATTTGAGATAAACACTTGATACATTTCGACGACTTTAATCTCTCTACGGTTACCGATTATGAAACTAGTTTCTGATTAATAATCGAGTTGGACAATACGTTATCCTTTTGCGAAGATTTAAGTAACGGTATTAAAAATAGTTTCTTATTTTTGGTTAAACTACATATCAGGATCAAAAGGACCCTGTTTCTTGTACACAAGAAAACAATCTTTCTAAAATATACCCAGAACACTGACGGTTCCAGGGAGATATGAAACAGAGTAGAAACACAAAATCAGAGATATGGATGTTTAAGATACAAAATCAAGGATATACCATTAAAAAATAAAAATATATAAAGCCAGTAACAAAAAAAGGACCTCCACGTTTTGAGGATGTTTTACCTCCATATTCCTGTTTGTATTTTTTAACGAACACATCTCTAATTTTGTATCCCAAAGGTGCGTGAACCTCGTCAACAAAAGACCGTAATAGTAGGGTGAAGGGATCGA

>Gm11ciRNA1284

GTAGGTCTTCTGTAACTTTGGACAACTGTAGTCTTTTCCTGTCTCACAATGTAATATAAGTTCTCGTATAGCAGTTTGCTTAGGGTTACCTTTCTTCTTGTGTTAAGGTAAGGTGTTTCTCTTTTGATCAGATTACCAATGACATTGAATATAACAAAGCTTCTAAGCTAGGAATTCAGATGCTAGCCATAGATTTGGAGTTTAGACTTGTATGATTACTTGGATAGTAAGGATTTTGCAGTTCAAAAAAAAAAAAAGGATTAAATGAAGTTTAATTTCCATCTTCTTTCATGGTTCCTCGTGTTACTCTTTAATAGTGATTCTTCCAACTGTTGGAGCTGAAATTACTAAGTCGACAGACCAGGGAATTCAGTGTTTAATCTTAACCCAATACTTTCCTCTGTGAGGAATTCTTACATGCCTTAAGAGACACAAATGTGCTCTGTATAAGTA

>Gm11ciRNA1285

GTAGGTCTTCTGTAACTTTGGACAACTGTAGTCTTTTCCTGTCTCACAATGTAATATAAGTTCTCGTATAGCAGTTTGCTTAGGGTTACCTTTCTTCTTGTGTTAAGGTAAGGTGTTTCTCTTTTGATCAGATTACCAATGACATTGAATATAACAAAGCTTCTAAGCTAGGAATTCAGATGCTAGCCATAGATTTGGAGTTTAGACTTGTATGATTACTTGGATAGTAAGGATTTTGCAGTTCAAAAAAAAAAAAAGGATTAAATGAAGTTTAATTTCCATCTTCTTTCATGGTTCCTCGTGTTACTCTTTAATAGTGATTCTTCCAACTGTTGGAGCTGAAATTACTAAGTCGACAGACCAGGGAATTCAGTGTTTAATCTTAACCCAATACTTTCCTCTGTGAGGAATTCTTACATGCCTTAAGAGACACAAATGTGCTCTGTATAAGT

>Gm11ciRNA1286

GTATGTCCTGAGAATCCTATTCCTTCCAATGTCCCCCAGTATTGGTGCATGTTGATCTATATTAGTGATTTAGTGAATGTCCACATTCTGTAGTTCAGTATCATCATTTGTTCAAGAGCTAAGACTATTCCTGTGGCATGTATCTGTACAACTATTTGTTCTAACATCCACGCCTAAAAATGAATGATGATGTCAAAAGGTTTAGTCCAGTGCAAAAATAGAAACAAACTTTTGAATAAGCTTCACTTTATGAGCT

>Gm11ciRNA1287

CAAACACATAAAAAAATGGTTAAACAATGAAAAGTATAAAATCTAACGGCTGCTGTAACATTTTTAAATCCCGTTTAATCCCAGCACGAAGTTTACACTGTTAATTAAAAAAGAATAAATACACCTAAAATTAAAAATAAATAAAATTTAAGTAAGCTCCAAAAAACGCTAATAACAGACGTTACGCACTCACAACTTTAACCCCTTTTTTAACAGTGTCAACGTTAACGCCTCAAGTTAAACTTTGGAAACACAAAATATAAAAAATACACTTAAAAATAAATAGCATAAACTCACAAAGACTACCCGAAACAAAACTAAAACACAAAACCTAATAGAAACAAATTACCTTATTAAGCAAAAGAAAAACAAACTTACAAAGCAATAACTAACAACTACTCCTACAAAATCTGATGCCTTAAGTACATATCAAGATGACAAGATGGAAAACGGACACAAAGATTGAATCTTCTGTCAGATGAGAAGTTCTCTCCATCAAGGTAACATACCGAAAAACACATACTTACACAAACTTATTTGATGGTTAAAACAGAGACTTCATAATGACAAAGAAGATTCATCAAAGATTTCGTTATTTTGATAAACTCATTAAAAGATTCATAACTAATAACTTATTAGAAGTGGGTCATGGATACAATTACATAAAATCATTTCAAACCTCGGGGTAAACTTTCTAGAAAATTATAAGCTCCGTGATAAATCATAACTAAAATTACATAAACTCCGTGATAAAGAGTTACATTCAATCAGATACCACAGAAACGACGTTAACTTCCGTAAGTTTTACGTAGTACCTAGTGAACACACTAAACAATGAGCACACAGATACACGTACTAGGAAACAATCTCTCCGGTCACACATAGATACACGTACTAGGAAACCATATCTCCGATTACAGGTACTTTCTTAACGAGATTATAACAAAATCATGGGTATTA

>Gm11ciRNA1288

GTTCTTTAACTATCCTCCCTCTCTTCATCTGTTACTGTGTTTCCTCACCTCTCTTCCGTTTCTATTACTTGAACATATGTTTTGAACCTTTCTATAAATTTCGCAGCTGATTTTATGCAAATCTAACATACAGTATTATTTTGTTTATTCTTGTTTTTGCATCGTCTCATGGACACACTATTTCATTTCCTACCAATTTTATTTACTCTTTAATCTATCGGTGGTAACCCCCTACCCTGTTAATTTGAAAACAATTGCTATTCTTTATGTTAGTTTTTGTTCCAGCGAAATCCCTTTTTGTCAGACAAGAATTTTTCGTGTCTATGACCACTGCCTTTGCTCCTCCTTGCATCCTATCCAGTTGCAATAATTCAGTTTCAAAACGTGAATACATTTCAAAGGACAATCACTTTTTTTCCCCCAAGTCACACCGACTTTTGAATGTGATTTGTCCGCTGCTAATATTTTAACGATTTTTTTTTACTTTTCTTTCCTTATTGCGTCCAGCTTTCTCCTCCTCTGGAAAGTCTGTGTTCCCATTAGTTTATTCTTTATATTTTGATGTTTGGAAGAGAATTCGGGAGACAAGGCAATTTTGCTAA

>Gm11ciRNA1289

GTATGTCTGAATTCTGATAGCTTTTTCGTCTTTGCTGCTATGAGCATGCACGAATTGTCGTTCCCCTTGTATTTGGAGTATTGGGGGTAGATGAGGGTTTAGAGTTCTGTTTTCTGGCCTGTGGTTCTCAACGATTCTGTTTTAATTGTTACCGCTCATCACTTATTCAATTTTGAAGGTACTTGATATTAATTAAATTGTATCCACATAATTATATATAGCTTGTTGCCTATTACGTAAGTCGTTTGTGTTCTTGTAATGATTATTAGGGTATGTTTGGTTTTAAAAATTAAAATTCCGGCTTCATTGAGTTCTTGTAATGATTGCAAAAGTGCCATGCTATTTTTATCTTGTTTCTATTTTTAGTTCTTGTAATGTTGCTTTACTAATTCATATATATTTTTGACCTTATTTGTGCTCAGCTTTTTGTCTTGTAATTAGTATTCTACTGATATTATCTGCCTAGTATACTGATGATAAATTGATAATCATTGTACTTCTTTCTTCTCCTTTATT

>Gm11ciRNA129

CACTCAGTAGTTGAGAGTTAAGGACTGGGATTAAAGGGTTAGGTTATGGTAAAGAGGAATCTAGGGATTAGAGAAGACTTTAAAGCAAATTAATAAATTATAAGAGTGGCAGGTTATAATAAAAAGGGGAAAAAGAAAAAAATATTAGAAACTTCGAGAGAAAAGCCAAGAGCGACGTATAAGCTAAAGCTAACAGTTAAAATACCTACGAAATAACATATAGATATGCTAAGAGAGACAAGACTAACTACTAAATCAACTAGACACCACAGAGTCCAAGCGGCATGAAGTTACGTAGATACTAAAAAATAAAAATTTAAAAACACTTCACTAAACCACAATAATAACTAATTAGTTTACAACGATCTCAGCTTTCTGACTACCCTCAAAATAAACCTAAACATAGATCTAAGAGACTACGATAATAATAATAATAATAATAAAATTACTAATACTAATAATAAGAACAAAAATTACAACTTTAAGTGTATAGGAGGGTACGTGAAAAAAAGAGGACAGTGTTATAAGGAATTCACTTACAAAATGTTAGAATTAGAGACACTTGAACAACGTAGCTCCCCCACGTACTATTGGTCGACACATCTCCCCATCTATTATTAAAAGTGAGAACGTCGACACCAAAACAAGTAAAAAGAACTCATACGACCTATT

>Gm11ciRNA1290

CAGGCTTAGATTAACAACGAGGGTGAATAATGTTAATTATAATAGATGAATTACCTTTTTGGTTCTGGTCGCTCGATCTAGAGATAATAAAATAAGATAAGTTAAGTAGAATGACACGGCAGTGGTTGCAAGTTAGGTTACTTGTGGTAGATGAGATAATAAAATAAGAAAAGTTAAGTTAGGTTTCTTTCTTTATCTAACTCGATCTAGAGCACTCACAAG

>Gm11ciRNA1291

CAATTACTAAAAAAAGGTTTAATGACATACGAAAAAAAACCCCTTTTTAACGACATATGAAACACTGAACACTTTTACGACGTAATTAACAAAGTAAAGTAGGTGTATTAAGGTAAACCATTTACGTAATTATATAATGAAGTAAATGAGTCATAACCTAATGACACGGTTCACACGTACAGGAACATTTCGACAACGTGTAAGGGAACAAACAAAGTCAATTAAC

>Gm11ciRNA1292

GTATGTGTTGTGGTCAATGGATTGTGTGAGGATGCATAGGTTACATTCTATGCGCCCATGACCCTTGATTGGATTTGAATTAAATTTTTGTTTGGATAAATGACAATCAGTCTATTAGAGAAGTTAAATGAGAGAGCTTCTATAGAAGTTGAAGTGTACAAGTTGATTTTCACTTATAGGAAAAACTTAATTCATTTTACCTTTATCTTTTTCTCCTATAATTGCTTTTTGAAAAATTTATTCACACGGGGCCTAAAGGTATTTGTTTCTAGTTGATGTGCTTCGTGACAATTGGCTTGTGCATTTTGTGAATGTTTTCAAGTTTTGGATAACTATGTG

>Gm11ciRNA1293

GTTCGCGATTTCTCCTTTTCTCTTACGATCTGATCTCTTGTCAATGCCAATCTCGATAACCGAATCCTAATCCTTTAAATATTTAGCTTTATCTTTATACTCACTTAGGGTTATGTTAATTCGTGCCATCAACGACTCGTGACTTTTGTTTTTACAGTTATTTATTGCAAAAATAGCGTAGAGATGCTGAATTAGATTCCGATAGGGTTTTTGAGACGTGAAAGGGGTAAAACACGGTGAAAATTGATTTGGACAAGAAGTTTATAGAGGAGAAGTGTGATATAGAGTGAGTATTGAGCT

>Gm11ciRNA1294

CATTCTCTTTGAGGTATAAGTTAATAAGTACACATACAGTACCTAGGACTACAGTATAACGCAATATACACCCCGACACGTAAAGTATACATCAGTATCAGACCAAGGATAAGTCTGACAAGACAGACTGATACCTTGGTAAACATACCGAAGTGATGGGTACATAATTCATGGAGACCATGAAGTTAATATATATATATATATATATATATATAAAAAAAAAAAAAACGACTATTTTTTTTTACACCCCGACACGTAACTATTTATGACCTTTGAATAATAAAATCGAATTTAGTAGTTAGAAGTCACAATGACTTTTTGGTATCCATGAACAATTGATAGACAAATATAACTATAGTTAGTAATTAGAAAATACATTGACTTTTAGTGTGGATGAACAATTGACTTTTAGTAGTTATAAACCACATTGACTTTTAGTGTAGATTAGATCGTAGTTTTACGAAAAGGAGAGGATTATCCTTTTTTTCCAGAACCATAAACCATTATTTTTAAGATGTAGTTCGTAAACACATATTCCGTTTCTATTTCTTTAGGTCAATCATAAGATTAGATCGTAGTATAATGATATACGGTATTAACAAACTATATTTTTAATAGATCCCTACATTCTATTCTGGTAAATATACAGAAGTGGTAATCGTATTTGAAAATCCTGACACAGTAATTAGATGATAGGGTTTTCGAATTCGACAATCCACTTTTGTGTACTGACCGAAATATAATGAAGAGATTGTTATTAACACTAAAATTCAATCTAAATT

>Gm11ciRNA1295

CAGGAATCAGCAATGGTGAATGGTGAAAAACTTACGAAAGACTCAAACAGACGGAACGGAACGGAACGTTTACACATATTTTAGAGTCGAATAAAAGGAACAAAGTACAATTGTTACAACTAATGAATATGAAAAATCAACTTCCTATAGAAGTCATCAATGATCGATGTGAAAACCAAAATTCACATTAATATTATACTAGTAAACACACATGCTGTTTCCTACCCTTCTTAGAAACCACAAAACCATCGATATGAGACATTATACTAGTAATATTAACGTAAAACTACCTACATCATATTGACAGATACTAGCCCACTATAAATTATTAACTATATATAATGTACGGAATGAGATCTGGAAGTAACGTATTAATTATCACACCAACCTAAGTGCCACTACTTAGGTTTTAATGTTACCGTTGTACTTTTTTAACCTTCGTTAAGACATAACGAAACGGAAGTTGTACCTCTTAGACTTAACTAAAACAATTTTCACAATGCTATTTACCACAAACATTAATACGGTTCGAGAATAAAACAAAAGAAACTTACTATCTAGTCAGGAGCTCCAACAGGACACCGAATAAAGTTAAATAATGTCTGTGGTAAGATCAACGTCTATTCTTGATAAAAGGTCAATTAACCGTTTGGACAACCCACTTTCACCTTGATAAACCTGATAAGTACTACTATCACTATCAGGACAACCAACATAAAAATAGTGATTACTTAACAACTAAACTCACAGAATTAAACTCACAGACGAAATGAAAATCTGGATAAAAAAGGAAGAGTCAAGAATGTTTAAAATAACTAAAGAGTGAGTAATAAAAAAGTAGTACAACATTACAAGTTATACCATACT

>Gm11ciRNA1296

GTAAGCCAGACTGCATACCCTTCTCCTCCTTTCTTGTTTGTTGTCCACTGTCACTTGTTTCTACATGTTCTCTCTGTTTGTTTTAAATTTAAAAATCATCAACAATTTCAAACTGAAGATTGACATTTTATATTTGTAACTAAATACCAAATTGCTTTCTTGTAGTTACTACATAGGAGAAAACTGAGGGTTTTCTTACACTGCCTTCAGTTTTCTTCCTCTTACAATTTCTGTATTTTTATTGACTGAACTGACCTTTATAGCAGCCATTTCAGAGTGTTGGCTTAAATTATCTGTTTGCTTTCTTAAATCTCATTTCTTGCCACTGGCATACTTTACCTTTCTCTCTCTCTCTCTCTCTTTAAATTACT

>Gm11ciRNA1297

GTATCATCAAACATTTGATCTTACGTATTTCACTGTAAAATTGATTTATTTATTATTAAATATGAACTTCTGTGGGAGACTGAGAGTGTAACATTACATCACTAATTCCTCTTCAAGCTTTCCTTAGATGCGTCCTGCTTTGTTTTGACTTTATTTTCTGTTGGTAGATAAATGAAGTGTTAGTGACTTGACACATTTTAA

>Gm11ciRNA1298

CACTCAGGAGAAACTAAAAGTATTTAAAGTAAACACTACCTACCGTCGGAAGGTTCACTACAGATTGAAAACAATATTAAACGACGGACTATAGACCCTATAAATTGTTTAAACTTATAAACGTTATAAATTTACACAAAATATGAAACTATGCTGACGTTTTGGGTTTCTTGTGAATGGTTAATAGTCAAGACAGGATATAACAACAACAGGTGTCTTAATAGGATAATCCTTATCGACGTAGAACGTAACTATAAACCCGTAGTACACTACATCTTAACAAAAGATCCTACTAAACATCAAAAGACCGAATTAATTCACGTTGACTTAGCGGTTGATTCTAATAGGTCCAGTCTGAATCGATTCGTCTTACATCGAATCTTTCATTTGACGACGGTTGAACCACGTCTTAAAATATCTACAACATATAATATTATAAACAATCGTAAATCCTTATGAGCGTAGGACCGAACTTTTTTAAGAGTGAACCGTACCTGAAGGTTTTCCCTCTTTGACTTTATGAGCTACAGTAACCCGTGTTTCAAACGAAGGGAAGAGGAAACACTAACAGAATATATCCCCCACAGTAGATAAAAAAAAAAGAAAACTTTTTGCAGTAGATAACCTATACTTGATGTACTAAGAGTCGTTTACAAACGGGAGTCCCCCAATAACTAAATTCACAACTTTGGCACTTCGTACCAACTCTGAACTCCGTAAAGGTAATATCCGTTAAACCAACAAATTAATAGACCTGATGTAAGTTCACATCTTAACGTCCCAAAGATTCGGTAATCATTATCGGACTTGACTCTTAGTTTTACTAGAACTCCTCCAACCTCACGTTCCTTCGGTTTTAGAGATTAACAGACAAAACAATTATACATATTAACCTATATTACTACCTCAACCACGATCCCCGTAGTGACCGTGGGATCGTGTCCTAAGTAACTTCAGGAACACAGTGTATACTCTAGCTACTATTATTGAACTAGGACTTAATTACGAATCCACCGGACAAGTTATCGAGGAGAGACTTCACCACCAAAAGTAAAGGGAAATTTACAGTGTTCAATGTATACAGTACGGTCAAAGACAGTAATCTACCAGAACGATCTTATTTCCGGTTTATTGTGGTTTGCTATGTTCGACCACACAATCTAACTTTAACAACCAATTGCAGATTATCGGACTAGTGAACAGTTATTGTAAGTTTCCTAAAGAACACTTACGAACCTAACCCTAGAAACGTAGGAATAGTTATTTTATAAAACAATGTAAAATTCTTCAAAGGTTTCCAATTTAAATTCTCGACCTTTTTTTCCCTGTAACCCTGGAATGAAAGTTGTAATCGCGTTAGAGTATTGTAAAGTCCTGTAGTGTCAGTTCGTTATTCCACACAATAAATTCCGACGGTGTACAAAAGGAACCGAAGAAAAGAAATAGTTGAAAACGATTGACGTTTAAAAAAGACCGAAGACGTTAAACATCAGACCCTATCGAAAGAGAAATGTAAATGACAAAATTTTTTATCAATGTTTTTACAGTGTAACCACAAAAATGACAATGTCTTTTGTGCCATTTTTATTGTACCGTAAAACATTAATAGTTCTTTGTATTTGTCTGTTACATAACGGTTTGGTCAAACTGGGATTTTAAAAGAGGCCTTCAGTACCTCAAATACAACCGAGTATAGTCCGTTTAAAAACACGAACAATGAACACTACATCGTAGTTATCTCTCACGTACTTAAGTACATAGGAGTTTTGATAATAATCCTGTAAATATAGGCATACTGATA

>Gm11ciRNA1299

GTGTGCATCTTTAATCCACGCTATTTTCTGCACTTCTTCTTGTTCTTTTTTCCTGTATATTTTACTTTTTTTATGTTATATCTGCTGAATTAAAATACAAAAATCTGAGTATTTTTAATTAATAAAACTTACTTCTCTTCAGTGAAATCATGAGTGGTGTGATGACTTCATTATGCTGACCTTCTTTTCCATTGTATGCTTATG

>Gm06ciRNA13

GTAAGCCAGCTTTAATAAGTGGGCTTGTTTCATTCTTCTTTTATTCTCATTTTGTGGCACTTGATGGTTTTAAATTGTTTTTGTGGTTGCACCACAATTACTGAAATTGTGGAACATGTGGGCTAATGTGGTTGCAATTGTAGTCAGGATATAGTTGCAAAAAACT

>Gm11ciRNA130

CAGTCTTTAGTCTTATTTCTAAGGGACGAGAACACGAAACAAAAAGAAAGAAAAAAAAGTTGACTAAAATTAATTGAGTTTGAGTTTAAGGTTCTGTTATAATCGTAAGTGAGTAATGACAAGCAGGACAGTCTAATAGTAACAGACTGACTGGTAGTCAATTACAGACCGACCAAGATGTTTAAAGTGCGAGGTTACAGACCACGAATTGAATATAACTTCTAGGTTATAACTAAACATTATATTTATTTTATTACATATATTCATCTCCTTTTAAGAACTTAAACTCAATTCAAATTTGACAATGTTTCAAAACACGGAAAGACCTAAATAAACGCGAAATTTTAACCACGAATACAATATTACATAAGT

>Gm11ciRNA1300

GTATCCTGTTATTTGCACTTATACATAAACCACATGCATCTCTGTACGAGTTATTGACAATTAATGATTAGTTTCTTTTACAATAAAGCCTATAGAACGTTTGGGCCAATATAAAAACTTTTACTTTTACTTTCACATTTTCTATCTCGTAACATGTAGGGCTAATGTCTAATGGATTACCAAATACCAAATACACTCCAAGTCCCTCGTAGGTTTTGGTGACCATTTAAATAACTTTCTTCTATCAATGCTATCAATGCCACATCCCCTGGTTTCAGTGGTTTGCTGCATGTTTCCTTA

>Gm11ciRNA1301

GTTTGTAAATTAAATTGATCCCTCAATCTGTTTTTCTCTTTGGCATTTTGGTTCTCTTGATGTTTCTATTTTCTTGACATCTGAGTTTTCTTTTTGGCTGGTGATGGAAGTTAAAATTCAAAGACAATCACTGTAGCAGGCTTAAGTTGGCTTTAATTAAGGATCATAATTGGCGACCTTGACATCAAGAAAATTCATATTTGAACTATTAGTATATATGATGTGACTCGTTTCTATGTTAAGGGAAGCCAAATATAACAAATAGGAT

>Gm11ciRNA1302

TATAGGAAATGGTAATAATGAAATAAGAAACCACAAGTAAGGAGGACTATGTGATTTGAAACCCATGACTTATTACGTATCCAAACTAAGACGTTGATAAGTAGTAGGTATCCACTTTAAATACGAATACTTATGTGTCATCAAATCACATGATTCGGTAAACCCCACGAATCGACTTACCTACGATATTAGAACCCTCAAGATACCTCCTTATGTACAATTTTTTAATCAAAATAAATATTTATAAGACAGAATAAAACAGAACACGTAGATATATAATAACGACCCTAATGTAAAAATAAAATAAATGAAACAACAATTTAAATACTTACGTCGTACACTATACGACGTGACAAAACGAAAATTGTTTTTAACCTGTACGCTTTTTATAAACATTTGTCTAACTTTAATATTGTTTCTACCCAATTTGTTATTTACCCATCCACGTGTAATCAACCACTAACCGAATAGACGTACACACACACACACACGTGTACACGTATTCGTAGATGGTTTCCGTACTAGTTCCAAGGAA

>Gm11ciRNA1303

GTAATTATTATAATTATCCATACTCAATTCCCTTTTTTTCTTAGATTCTCTTACTATTTCCAATATTCGTCGCTTCTCGGGTTTCAAATTGAGAGGATAATAAGTTTAGGTCGAAGAGATGGTAATTTTGCTTGTTGTGGTTATCAGATGTTGAAAGTCATATCGTGTGGTTTAATTCAATAATAATAGCTCAGCTTATTCGACGATTGAAGTCGATGATGCTATTGGCGTCCCGTTTGTGGTTTGCGTTTTGTGATTCTGTGTTTACT

>Gm11ciRNA1304

CATTCACCGTTCACAACGCCTTAGGCAAAAAGAAGAGTATAATAAACAACTAATACGCAGCAAGTACTATCCTTTGAAAACGGCTGACAAGATAATTTTTATTATTTATACAAAAACATGGTTATCAATCACCCAACACACAGACAATTCGAACAAAGATTTAAGACTAAATTTACTAAAATAAAATATAAACGTTCACCTCGCTACGTGTTAGAACAACTATAATAATAAAGGACGATTACCACGTAGTAACTCTAACTATAAATAATAAAGTAGTACTGACCACCACTTTTTATGAACCGAACGAATTTTTGGTTAACGCGCATTGAAAAGAGTGACTACGGAACCGTATACCAAAACACCAAGGCTTGGAGCAAGCGTGATTAAAAATCACTCTTCCCAAATTACAAGACTTCAAAACGGCACGAATTCAAAAGTAAGAAAGTACAAATTATACGAACACTCGACAATTATAAT

>Gm11ciRNA1305

GTGAGTCACTGATTTTGGTGTGTGTGTTATTGATTGGTGTTATTTTTCTTTTGTATTTTGCTTGTGTCTGGTGTGATCTGTTGCTGTTATTTCATTGCTATTTTTTCGGGGAATGTAATGTAGCAATTTCAGTGATGATTGATGAGTTAAATTTGAATCTGGGGAGGTTAAGAATTG

>Gm11ciRNA1306

CATACCCTTGTATTATAATACACAAATAAAAGGATAATGGGTCAACTTAAAATACTTTAATCACTTTTAAACTTACAACTTATTATTCTTGTTTCTTGTATTATTTAACACCAGCTAAGTTGACAACCAATGAACAGTGATTAGTCTCAGTCCGACTCAATGTATTCGTCGTTCTTAAACTTCAAAGAAACGGAATAGAAAACTGAACCATCCCTACTTTATATACACGTAACGAATCAAATTATATCATGACGAAAGTTTCAAAGATTCAACTGTAATTTCAACAACTTTTTGAGTTTATCTCTCTCTGGCCCAGTTCGTATGTCATAACTTCAATCGCCTACTGAAAGGAAAAATTTAATTAATCAGCTGTAACTGCAAACCTTGTATCCTTTTTTTTAGTACTTAAGTCCTATTCACGACTCATTATTAGTTAAAGTAATTTATATTTCTGTTTACCAATCAACTCAACACTGAAGTCTCAATAATGATGGTAGGTTTAGAGTTTAAAAAGTTACCTAACTCGACTCGAAATCTATAAATTAACCCAAATTGAACTCGCAATCGTTCAGCGCCAGCGATATCAACAAGGAGATAGAGAACTTCCCGAGAAAGAAATAACTTTTGTAATTTAATTGATTCATTTTTTTATAAAGAACAATACACTAGTTACATTTTTTATGAAGTTTAGATGATATCATTCGAATCACCACTTTTTAAATTCATCAAACATAAT

>Gm11ciRNA1307

CAATCGAAAACATAAACGAAAACGGTAAAACGCAAAATTTCGTACGAATACAACAAATATGAAACGTAGTTCCAGAAACGGAAACGGTCACACCATTTGATTTAAGAGACTCGAAGAAATTCTGTATCATCATCAACTTATAAAACAGTACGAGATGCAACGAAAACGATACAGATCTTAAAACAAAACCACCAACTGACAACAAATTACACCATTATTATAAACAGTGTAGCGAAAGAAGAGTACGTTGTTCACGTATCATGATGGCATGTCTTACCTATAA

>Gm11ciRNA1308

CATAACTCACACTTAAAACGACGTTAAGACAAAACTTAATTACACAAGCACAGACCAACGAGGAACCTAATAAGGTTCAGAGAATGCAACTTTATGGTTACTTAAAATATGCCGCAAAATGATGATTGAACGAAAATATTATCAAGTGTAGTGAAGTTTTAATTAAAGTATGTTTTAGTAAAAACGGTTGTGATAAAGTTTGTGTGTGAATCTAAGAAAACGAAGAATCCGTCTGTAGTACAAAACATTCGGCACGACT

>Gm11ciRNA1309

CATTAAAGACTAAGAGATTGAGAAACAAAACAAGCACAAACACATTTAACTGTTAAAGATCAAGTAATACGTTAATTAGTGATGAACGTTACAAGGTGCAACAATCACAAACTAAGCATACCTACATCGATTATCGTTTGTTGAATCTAAATTAACTCAATAACGGCTCGAATATTAGCGGAAAAGACTAATCCTGTCCTATCCCGATTAGACCTTCAATTTCCTTACCACTTAAACCTAAAACATCAACCTTCCTCGTCCTCAAACCCAAAAGATTCCAACATTACATCACAACCCCTATAATTTTCATCACACAGACCGAATATCTTTGGTGTGCTCTTTTTCCAAAGATTTGCCTTCTTTTAGTTCCCTCCTCCTAAACTTCATCTCTTTTGGGGTGTGAAGTAAAACTCCAAAGGTAACCGTAATACGGGTGAAAAACTAAGTATTCGCCCTCTATCTAAAAGACTTAATGAGTTCTTTTGACGTCTATACTACCTGAAATTCCGAAGTTTAAACACCAAAAATTCCAAATCAAACCTCACTTTTTTACTTTCGTTACCTATTTACCTTTTGTACTTGTCTCAAAAATAACCGTTGAGTGTTGTAGGAATTTAACGGTCTATGAACTACACTAAATAAGATACATTCTTACCCAAACACATAACAACAGCTTATAAGGGAAGTACACAAATGTAATACATGAAGTCTTTTAGTCTTTTGGTAAAT

>Gm11ciRNA131

CACTCCTTTTAATGGAGGTTAATATAGTCCTTAGGTTATGTTATTTTTTTAATAATCTCTGGGTTTTATTTTTTACTAGTAAATACTCCCTAGGTTTTGTATAAAATCGGTTAAGTTTTTAAACCTAACGTAGGTACTGACTTGAAGTTTAGATT

>Gm11ciRNA1310

CATGAACGAACTACACTGTTCGACATTGTCGACAATGACAAAAGGGAAACACGAAAAATCAAAAATAAGTCGTCAAAAAAATACGGTTAGTCAAGAATACTATTATTATTGAAAATGGACTACGAAGACTATTAGTCAATGGGACGAGTGATATATTCAGAGAGTCTCCAGACTAATTTAATTGATAATTGTTATACTAATAAACAGGTACAAAAGAGAGGTGTTTTTGTTTTGTTTCTCTAATTAGGAAGACGTAGAAACCGGGAAGTTAATTCAAAGAGAGGTAAACTATAAAGTTTAGTTTTAAAACAAACAACGTGAGGGTTTACCCTGCTTTTTTGTTTGGATGTACCGTGTAGTAGACGAAGCAAATATAGGAAAAAATAACTTCAACGAAACTACAAACATACACTAATAACAACAAAATGTATAAGAAAACATGGAAGAATAATAAGATTGTTAATATTAATCTCGTCAATAACGGAAAATGGAAGAAAGGGAAAACAAAAACAGAGACGTAACTTCTACAGAAAAGGAGGACAAACAGGTATAGGGAACACAAGGAGAGAATTACCAAATGAAACTTTTTTAACGAACACAAAAATCCGAAGCGATAAATAAAACCGAAGATGAGTTAGTTTAACA

>Gm11ciRNA1311

CACACATGAAAATCCTTGAATTAACTAAAAATAAAAAGGGATGACTTTACGATCTCCGTGTTAAGAAACGGCACACATCAACCTTATTTATAAGGTTTAGTAGCTGAACGTACGGAAGTATACAAATAAAAAGGAGACAGGGAGCCCTTCTAATTAAGTACAACTACGTAAAAGATAACTAGAGGATACAGGTAAAAAGGGTAACGTTACGACGTAGAAAATACTACTGAAAGGAGAAACTATGAATTAACATGACCGTTGGAACCACTGTCGTGTGTTGAAGACTCTAAAAGGAAAGAACAGATAAATAAATAACAACCTAAGGAATGGAAGGATTATACCAA

>Gm11ciRNA1312

GTACTAAAATCTTTATCTTTTTCAAATAATGTGCTATCAAGGATTGCCTTCACTATTAGAGTTTGCTAATGGTACTCCTCATAATATGTCCTGATTTATACTCAGACTTTGCACTTGTTTGGTTTGCTAACTACAATGTGTCATGAGATCATAAGTCAGAACTCAGAATTATTTGGTTTTCTTGTTATTTGATTATTTTTGCCTAACTCCATGGTTCATTCAAAATTCTTGCCAAACACTACTTCATAGTTCATACTAACTGCGATTTGGCAGTGTATTAATTTTAGAGTTCTACCTTATATCCATTTCCGTAACGAAGACTACTATGAAATTGTCCTTTGCGGGAGATTGTTAGATGGTCTAGGACGGAGAGGTCTGTAAAATAATCAGCAGCTTTCTAGGAGAAGTCATGTTTGCATGGTTTTAAGCTATAACTGATGTGATTTGTGTGATTTGGTGTGGGTTTTTAGTTATCTAAAGCAAGAGAATCCATTTACTCCTGGAGCATACAAGAGCCTCTCTCTTAATTTGATGAGTTTTTCAGATCTCATGCATATACAATATTAAGAAAGGCAGCTGATCAGAATTTTCATTTAAAAGAAAATCTCAAGTGTTTCTTTTTGTTTCCCACTTAAGTCACTTATTTGCTTACTCTTTCT

>Gm11ciRNA1313

CATTCTATGAACGAATAAGTAAGTTCAAAAGTAAAAAGAAAAGGAAAAACTTACTGCTTGACTCTCTCGACCATACTAGGTAAACTGGAAACGTACCGTGGGTATAATAGGAATAAATTTATCCGTCTATACTATAAAATCTCATAAATCTATATATTGTTTGTCTTTGGATAAGTTGAACGAGAATCAAGAAGCAAAACAAAAAGCTACATTGAGAAACAAGGACACTCACATGTTTATAATATAAAATTAACCTTCCAAGTTTATAATCCAAAGGGACCTTTCCTTCCATGTAAACGAATACTCAAATCCTAGACTTATTGAAAGAGAAACGGTCAACGATTTATAAATACAACCCTCCGTATTTATTATTCGTTACTGTTCTTAAAAAAATTATAAGAAAAGAAAAGAAAAGAGTTGGTGAGTCATATCTTGGGTTCTAATTGGAAACTTTGACAACAGATATATTTAAACGGTTTTACCAAAAACTTCAAAAAAAGGCTATCACTGTATAAAGGACTTGAGAACATAAAATGAATATACACTCTTCGTCGATAGTCTATTTCTTGTTTCATCAAGTTTTTACTTGATTTTAACACAATAGATATGTTTTATTAACTAGTGTGACAAAAGGAGGGAAGAGGAA

>Gm11ciRNA1314

CATACATATTACCAATCGTTGAAATTATACAACGAACTTAAACTTAGGGCTAGATTTCACCACAACGGTCTAAGACGTAGGACCATGGTATCCCCATTATTTTGAAAATAAATACGCACAAACTCATCAATACAATCCTAGTCAACTCTGATTCTTATGAACTCACAGAGTGTGTGAATGTGTGTCGACTTTACATAAATATATAACTCAATTGTATTTTCGCCATAGAGATTGTCTACCGATGTCGTCTCTCTTTTATGTTGTTTTATATTTTTTGATGTATATATTTAGATTGTTCTAGGTAGACATTCCATACTCTGAACACAGGGTGTATCCTTCATACCCTAAGATCACACCCCAAATATTCCGGAACCCTAGAGGTTGATGGTGGCGATCGAAAACACCACACAAAGAGGGTTCCGAAAATCAAGACGATATGACGTACAAATTGCAAGTTACTGTAGGACCGTCCAACAGTATTGTTGGTGGACAACCAAAAAAGACAATATGTCAATGTCTAAGACAATCTAACAATCCCTTCCATGTTAATTGAAATAAACACATCTGAGAAATCCACATTTACTAAGTAAAAACATTAGTTAAATCAAGTCTGAATTGAACATATAAGAGATAAACAACAAACAAGAGATGGCATAAAAACAAAGTAAAAGATACTAAAGGTATGATGTTACTTAAGAAAGTTGACCAAAGAAGTTTAAACGACATGAAAATTTTTTTTAACTACTCAGTAAAGTGTCCACTAAGGTCCATATATACTTACACACTTGTATGTAAGACAAACAT

>Gm11ciRNA1315

CATACTGTAACATCTAGACCATAGAAATAACTGTAAAAAGGGGAAATTACTCGGTGTTTAATGTTGAGGTTCACAATAGTTCAAACAACAGAAAAGGGAGAGTATAGAACTCCCACCGGACAAGAAGTATATCTGACACAGAGACAACCCCCGCCGTGATTCTTTTATTTAAGGGAACTGTGTAAATAAATCCAAAAAACTGAAAATCTAAAAATAAATAGACATCATGTTCGATATTTTGTAAAACAGACACACCTGTAGTACATTGAGGGAGAGGAAATTTTTATTCCTTAAGGTGAAAAACT

>Gm11ciRNA1316

CATTATTATTGATTGTGTTACAAACCTAGACCATATGAACACGCACAACAAGACACTCCACATTAGGAACAATAAAAATAATAATAATATCTAGTAACAATAAATGGATCCGTGTAGGTTTATACTACTAAGTAAAACCTATAAAAGTTCGGATTTAATCCGATACAACAAGAATAAAAAAAATAAATAGTTAAGAGAACTTTAATTATAACTTTGTCAGTGTATATAATGTACGGCTACTAACAACTACTGGCATTACAAGCACAACAGCTTATACGGAAACGGAGTAACGTTTCATCGACGTAAAAACAACATACTATTAAAATTATAAGTTACGGGACAAG

>Gm11ciRNA1317

GTGAGTGGGTGGTGAAACTTTGCCAGTTTGGCCTGCATTTGATGCATGATTTATGGTTTCTTATTCCAGTGCTGTTCCATGGGGCAGGGGCAAACCCTCTTGGCTTTTGTTATTATAGTTGTTTTTAAGAGGAAAACCAATCAAAGGGTTGGTCTTCTAGTTTTGGTAAATAGAGCAATAGAGCGTAGCTTTTTGAATTCGATTAAACTAGAATCAATATAGAACTCATACTCTCTATAAGGCAATGGCTTGCAAGTGACAAGCCCTTCATGCACAACTATAAACAGCTAGTCATATAGATAACAAAACAAGGCCCTACTTGATCTGTTGATTACATTTGCAGCTATGGTGAGAAGTAATTGTTATTGCTAAATAATGCACTGCAGTTGATACAATTCTAGGAAAGCATTAATATAGTTGATAGTCATGGACCAAGCATGCTGAATTCTCATATTGAAATACGTATGAACTGGAAAAAAATTTAATTATGAACTTGATACAGATCTTCAAACATGATCCATTGAAAGTTATACAGAGAAGTATAAAACACAATGTTGTGAACAGCCCTGTCCTCTGGATGTAATTAGACTTAGTCTCGGGAGGTTTTCTTTCCACATCCTTGATCCAAACATTTTTTTATACTTGCAAATAGAATTCCAGTAGTGTTTTCTCAACTGATACGTTTCTTTGGGTTCCCACTGGTATCAACTTTTCTTATCATCCGTCTATGATGTATCTGTAAGACTACAAGTATAACTCCCTTTTGCTTTTGACAAATCTTGAGCTT

>Gm11ciRNA1318

CATTATCGGAAGACGTTACGAGATAGACAATTTTTAGCAGATAAGTCCGAAAAGAGTTTTTGTAAACAAAATTATTAAAGGTTATGGTCTTGTTTTAGGAGGGATCCTGTCTATACGTTAGTTGGTATATAACCTATTTTAGACGTTAATCCTAGGGAACCGAGAACGAACACAATTTTAATGTGTTTATAGAAACAGAACCTCGTGATATTAAACTGTCTAAATCTAAATGAAAAACGACTGATATAAATTGTTACGCTCCAAACCCCTGTACAACCGTTCACACGAAATGCACTATATTCGA

>Gm11ciRNA1319

CAGACAAACAACGTCCGTAGTGTAGACTAGAGTGTGCTAAGCCTAAGCCTTAAAAGAGCTAGGGAAAGACTAGACACTAGAGACTAGACAAACGCAATGAACTAGAATACTATGAACACAATGATATACTAAGAAAATAATGAGAGTGCGGTTATCCATGCAAGTAGAAGAAGGAAAGTGGTTTCCGCTTCTAGCTAGCCAATGATAAGCCAAGACCAAAAAACAAAAGCACGACAAAAACAAAGTCTAGACT

>Gm10ciRNA132

CAGTGAGCAGAGAAGAGGAAGAGGGGGAGAGATAAAGACAAAGAGAGGGCTAAAAGCGAAGGGTTAAAGAAGGGAGAGTATAGTAGAATCGATCTTTAATTCCATAGACTAAGACTCCCAAAAGTAAAAAAGAAAGAATGCACTCAGTGTCCCAAAAAGTGAAATCGTAATCGTTCGCTAAAAACAAACGGTTGCCCAAATCGAATTGTTTTATTTCCTCTTGGCTAAACCCCCTCTCACTTTTTTCTTGAAAAGTAAAACGTATAAGTTAGTCAACAGCAAAAAAATTTACTTAAGTAGTTGGGATTAAAAAGTAGTGAAAATCCAAAATAATTAGCAAAATGCAAAAGGATTTGTACTTGGGATTAAGATAAGTATTCAGTACTTGGGATT

>Gm11ciRNA1320

CAGACAAACAACGTCCGTAGTGTAGACTAGAGTGTGCTAAGCCTAAGCCTTAAAAGAGCTAGGGAAAGACTAGACACTAGAGACTAGACAAACGCAATGAACTAGAATACTATGAACACAATGATATACTAAGAAAATAATGAGAGTGCGGTTATCCATGCAAGTAGAAGAAGGAAAGTGGTTTCCGCTTCTAGCTAGCCAATGATAAGCCAAGACCAAAAAACAAAAGCACGACAAAAACAAAGTCTAGAC

>Gm11ciRNA1321

CAGGCAAACTACGTCCGTGGCGTTGACTAGAGTGTGCTAAGCCTAAGCCTAAAAAAGAGCTAGGAGAAGACTAGACACTAGAGACTAGATAAACGCAATGAACTAGATACTATACTATGAACACAATGATATACTAAGAAAATAATGAGTGCGGTTATCCATGCAAGTAGAAGAAGGAAAGTGGTTTCCGCTTCTAGCTAGCCAAAGATAAGCCAAGACAGCAATCAAAAGCACAAAAAAGAAAACGAAACCTAGA

>Gm11ciRNA1322

CATACAAGTGGAAGGTAACATAGTGTTAAAAATAATAGAAAGGAAGAGAACTATAATAAATCAATAAAAGGTAAGGAGTGTCATAAATCCCTAAATTTACCTATGCAAATTAAATAGTCATAATCCCCCCACTTCCTTAGATTTATTCGAACTGTTTTACTCCTCTTTAGTCTTACAGAACGGTTTTAATAAATAAATATTACGATTTGATAAGTTTGCAGTACATCAGGTTCCAAGAAAAATTATAAACGAAGGGATGAAGATTTACTATTTATAGGATAGTGTAAAAAAATAATGTGATGAATACGAACTTAGAGTTGATGGAACGAAATGCGCAAAAAAATACCATTTTTAGAAAAGTGGTTACAAATATTTCACATTCTTATATCATATTCTTTTCCCACATTTTTTAGTACTAATAAACATCTTAGCCAATTTTGAAATTAGATTATATCAAAAGTTGAGATGATAACCGTAAAACTAGCTTAATAGATCATAATCAAACTTTAATATAACACGTACCCGTAGAACCCAATCAATCCGTACCTCTATTGTAAAACTG

>Gm11ciRNA1323

GTGAGTCTTTGTTTTGTTATTCATGGCCTTTGGTTATATGAAATTTCAGATGTGTTAGGTGGATGGCGTGGCTTATAATGTTTTTCATTTGTAATGCAGTAGTAAGAGTCAATCTTGGCTGCACAAATGAAAATTCATGAAAACTTCATTTA

>Gm11ciRNA1324

GTATATAAACATCTTAGTATGAGTATAATTGCCAAATTAAACATTGTTTATTAGCAAGGGTCAATTTATAGATTGAAAATGACATTTGCTCATAACTATTTATATCAATTGTTACATTCAAAAATATAATTGGCATAAATAAGTGATGGCAAGACTTGTATTTATTTGTGTTTTTTAACTACACTTTGGAGGGTAATTCTCTAGAAGCAT

>Gm11ciRNA1325

GTATATAAACATCTTAGTATGAGTATAATTGCCAAATTAAACATTGTTTATTAGCAAGGGTCAATTTATAGATTGAAAATGACATTTGCTCATAACTATTTATATCAATTGTTACATTCAAAAATATAATTGGCATAAATAAGTGATGGCAAGACTTGTATTTATTTGTGTTTTTTAACTACACTTTGGAGGGTAATTCTCTAGAAGCATGATGTA

>Gm11ciRNA1326

GTACAACTTTGGGACTAGAGTTGGAGTGTTGGCCTGCTTTTCTGTCTTGCCTTATTTGAATGATAAAAATTTGTAATTGCCATGTTAAGATTATGCGCTGCAATATTATAGTCATGCTTTGTGGGGGTCTCAAGGGTCATACATTAAAAAACAACTTAAACAAACATTCTAAGGGATTCTTTAATTGAGATAGGAAACATTTTGGTTCTTTCTGTTTACCTTTCATCTGAAAGACT

>Gm11ciRNA1327

GTGATATTTTCAGCTCATGATATGCCATTTGCTTGTTAGTTAGTTGTTATTATGTTGGTTAATGGCTTACTATAGTTACCCCTTTCTTTCCCCTTGCATAGCAGTACATTGATTTGTGGTTAAATATTAAACACCTGTTGGTTGCTTCAACGTCTATTGTGAAAGCTAACGGTATTCAATGAATATATGTTTGGCAACTCATCAAGGTACTCATATCCAGGAAAAAAACGTGGGTATCGAAGCAAGATTTTTCCTTGATTTAGCAATTATTTGGGAATATATCATGGGATGGTTTTTAAGTGTATTGGTTTCAGAGGCTGGTGTATTTTTTAAAACATGCTTCTTTGCCATCAGTGACTTCATTATGCAATTTTTAATCTAGATTTTGCATTCAATTTATCATTTCCCTTCGCTAGTTTACCACTGCCACTCTTAGTAAATAGTCTATTGTGTCCTGAATAAGAATTTTCAGTTTCCTGAAGAAAAGGGGTACAGCAGGATCCATGACAATTTTATTTTTTTATTCTAGTAAATATTATCAGAATTTAATAAATATTTGGATTGGTTTGGAGCCCCTCTCAGTCCTTTTTATTTCCTCTGTTCTTCACATCATCTAATATGCATGATAGT

>Gm11ciRNA1328

CATGAATTAATAGTTGTACGTGAGAAGAACACCTGATTTTGTTAATATGATAAACCGAAATTAGTTGACACCAAATTAACACAAAAATACATAGGTATTTATGTCAATAATTAGTTGTCAATATCAACTGAATTAAATTAGTTAATACATAATTTATACAAAGTTGTTGGTCGGTTATTATAAATAGTCTCTTTTATCCTGTAATTACAAAAATATAACAATAGGTTAGTTTTTAATTAAAGTAATTTCAGAACATTCCCTTCACCCTTTTTCTAATCCTACAGGCTAAAAAAAAGCTTTTAAACACGTCAAAGGTGTAATTTTTGGTTTTTAACTCACCGTTTTTATCCCTCTACATGCAGTGTGAGTATCTGATCCATTTACCTTATTCGTAGTTTGA

>Gm11ciRNA1329

GTAGTTATACTTATGCTTCCTTTTCCTTTGCTGATTTTTCCGTAGTGAACGCATGTGCTTGTGCTGATCGACCGTTTGCTTTTTAGATTTCTGGATTTTGTTTTGTTATTTTTTCCTTCCTTCTGCTTGATTTTCTACTAGTTATTTGCATAGAAATATGTATTCGAGTACGGTCGGGTTTTTTTTTTTTTTTTTAATCTTTGATTTCAAAGTATTTATTATGATTAGCTTATAGCTCAGATCCTCTTCTGAGGAGCATCCGGTATGAATTGCAAGGTTTTAAGTGCCGTTTGCAGTTTTCTCATGATCTTCTTGTTGCAGGTCAAAAACCTCGATGTTGGGATCAAAATCGTGGTCACAGTGCTTTTCAGAACTTTGATGAATTTGGAGGAATGATTTTAGCTTTTTAGGTGTTATAATCAAGTGGAGAAGGACTTGGATTTATATCTTGAAATATAATTAAAAAAAAATAATTGAAATCCTAGCCTGAAGATGAGATGGATTGCTTCTTGGTTAAAAATTCAGATTATCAGTAACCAAATAATTATTTTTGAACTTAGGACTAATATTGTATGGTGGATGTTGAGTAAATTTTTTGGTCATGTTTGAAGGTTTTTTGAATTGGTTGGATGAATGGTTGTTTAATTATGAATAAGGTTTTAAATTGCTGTTTTAGTTGTGGTTTTGTTGCAATCTTTTGATATTGCTGAAAATTGTGGACAAATGCCAGTCATGCATGACCGCAGTTCATGTTTCATTTGTAGACTGAAACCTTGTTGCGGCCAAAATCATGGTTGCAGGCCATTTTTTGAAACCCTAATTATGGATCCTTGTTTGTATTACAGCATTACTA

>Gm10ciRNA133

CATTGCCAGGTATCTAATAAATTCACTAAATAATAGATAATGATACTATAACCTGGCCAAACAAATTTGAATTTTATTCGTTAAAATTTTATTCGCCTAAATTTTTTTTACGAATAAATTATTCGTCTAACCTAAACAAATAGTTTTATTCGTCTTTTGACAAAAAAATTCGTCTTCATCAAATCTGTTAATGTGATTTTTTATCTTTAGACAAATAAAATAAATTTATTCACAAAAAAAATTGCTTATTCAAATAATAAATTTGTTTGACCAGGTAACATCCTTGACTTTTAATAAACATCTATATAACCTCTGATATGGAGGAAGTGAGTTACCAACTGGAGACGTTTGTGTCTTAAAAGTCCTTTTTACGTTTTTTTTTTTTCTTTAACTTTCTATCAATACATAGATAAAAGAGTTGTTTCGATAAGCTTTGGAGTTTATAAAAGAGACCAGAAGTACATAAGTCTTCGTAGTTGCGGGAAGTGTTTATTAGTTCCAGGATAACAAGGATAATTAGTACATAAGTCTTCGATAAGGTAACATATAAGGTTGAGGAAAGATTCCTGACCTTGAGAGTTAAGCATTATCGAACCCAGGAAATTTAATCCTGGTATTAAAGGTTGGCGGAGTTCGTAATTAAACTTTCAAAAACTAACATGTAATCTGACTTAAAGTAAGAAGTTTTTCTTGAAGAAGAGCCGTTGTTCATCGGATTCGACTACTACAATCTCCAGAAAAGGTCAAACTTTGGAACTTTAAAGAACGTGGAGTAGAACTAGAAGAACGTGGGCGAGGTTCATATTCAGAAAAAGTGGAAAGAAAACGAGTATCAGACCGAAAAAAAAAAGAAGAATAGTAAGTATCCGACGGAAATTAACATGAAATTGTAGGAAACCACAAAAAGATGTTCGTAAGTGTAGAAAGTAAGGGAAAATTATTAAAAACCGAGATCATTAAGGAGATCATCTGCAAAAAGGTGTTTTTAG

>Gm11ciRNA1330

GTAAGAATATGGACCCTTCCCTCATTTGTGTAGCCGTTCAAATTTGAGGACACACTTCCACAAAATAACTAATTATTTTCTTGATTCATACTAAATAACTAATTACTACACAAAATAACTAATTATTTTCTTGATTCATACTAAATAACTAATTACTAAGGTTGGTTTCACAATTGTTTTTAAAAGCTATTTGTTTTAAAATTATTTTCAATTTAATTGTTCTTAAAACTGAAAAAATATAAATAGGTATAGGCTGTTTCTGTTTTTTACTTTTGATTTTTAAAATCATGAAAAGAAGAAATCACGACAAACATATTCTCAAAATAAATTTAACAACCAAGTATTTATTTTATTTTCTCATCTCTAAATTTGTTTTTAAATTAATATTCTTGCCAAACATTTTTTGTCATTTTCTTTTGAAAAACAGTTCTTCAATATAGAATAATTTTAAAAATAAAAAACAGAACACAATCAAACTAACCCAAATATTTTATATTCACACGATGCTTTAGTCGTTAATAACCAAATTATGCAAAGAAAGGGCATTAGAGAAGCCTCCGCTATTGTTCTTACTAAAGCGATCATTTCCCTTTCTATCCTTCAGATTGTCTAATGGCATTATTTATTCA

>Gm11ciRNA1331

CATTCATGATGAAAGTTGAGTATGACATTTAGGAACTAAGAGACGTTAAAAAGTAACAAGGTCACTTAAATATTGAGATGTGCTGCACTAAATATAGTCGTGAACATCTTTTAAGGTTAATAGACATTAGATTGGGATCGATCATTATAAGACATTGCTAAGAAAGCACAAATAAATGTCCTTTTTTTTTTAGTTTTGTCTCAGCACTTACGAAAACACACCTAACATCTGCAAACAATTAATCCGACACTAAAAGAAAAGTTAAGAAACATTGAGCTCAAGATAGTAATCAAGTTAACAGATACCTAAAATAATTTACCGATGAATTAACTTAAAAGACACGTTTTCTTAGGAACCAATACGCCAATCTAACGCTTAGGAAAAACCACGTACGTCAACGATTTGTAAACTAACCATCCGACACATATGAACTAACTTAACAAAATACAAAACCTACTATTTAAATAACTTACCTATGAACGAGTTTATTAGACACGCTTTCGAAGGAACTAATACGCTAATATATCACTTAGCAAACCACT

>Gm11ciRNA1332

GTATAATTGAAACTCATCTTCATTTATTTTAAGTTTCTTGTAAATTAAGCTGAATGAGGTGGATAGCACTTTTTATGCTCTCATGTTAGGGTGCGCTTGATTGATCGTTAATCGTTCAGTTCTATTTAACAGCAATGACCATAGAGATATGGATTGACGTTAGAAGTGCATTAGAAAGTGGGAGACGAATAAAACAATGACACTTAATTTATAACATAACTCTTCATATCATGATAACTGTTGAGAATGCTTGTCTGGCTGTAACATTTTTGTAGCTTTCGTGGGTTTTCCTTGTAGCTAATTACAACCTGTATTCCTTTGATTTCCTTGATTTTTTGTGGAACTTTTGGTTTTTGGTATTGTTTTATCAACTCTAGATGTCATATATT

>Gm11ciRNA1333

GTACTGCTCGTTCGTTTTCTTCGATTTATTTTGTTTTTTGAAAACGCGGTTTATTTATTTTATTGAGAAATGTTAGTTGTTAGTTAGTTGTCCACAATCTCACGGAGCTGGGAGCTGTATGCTTAGCGTCCATGTGATTTTCGAAGACGTGATTGATATAATTAAGAAATTGTGCGTTTCTCTGATTCTCGATTTTCGTTCTCGATGAATCGGATTTGATTTTGAGTGAACTGTTAATCTGTTAGCTAGTGCTGAGTGTTTATTGTTTAGCGTTGACTTTTTTTTTTATTTGTAAAATTGAGAAATCT

>Gm11ciRNA1334

CATTCACATCTTATATCTTTCGATAAATCCACCATAATCCACAAAGACAACTAATTACCAGTACGAATTTAAAAACACTATGTTATAAATGAAAACCAGTACTAGTAACCTTGGTATGGGTGAAAAAACCCCAACAAAGAAGGGAAATGTAAAGTCTAAAATATATAAATCAAATACATTCATCCAGTAGGAACCAGTCTAAGACTAAACGAACCATTTAATCGTATAAGAATAGTAGAAGACTTTCTGTACTAATTTATAACGAGGAGCCCCATATCCCAACTTTAAGTTAAAAATGACGATGACAACTGAACAAATAGTCATAGTGAGT

>Gm11ciRNA1335

CATTAAAAACAACACGATTAAGAATAAGTGAGTTAAGCGCTAAGTTAATCCTCAAAGTAAGTCAATTTAATCCCAAGTTAATTCTTAAATAACTATCTTAATCCCAATTGCATTGCAACTAACATAACACCACCACTTTAACAACGTTCGTACAAACAAACCACTCCGAAT

>Gm11ciRNA1336

CATTCTGAAATTCAACTTCGTTTCGTTTGTTTCTTTATAGATTTAAGTTTAATTACCTTATAGTTAGTGAAAACTAAAAAGTACTAAGTACTAAGTAGATGACACTTAAATTCTACTAAAGGGAAACATCCAAACTTATATTATCAAGCTCCGATCTAATATTCCTCATCATATACTGAAACAAACCTACTCGAAAAAAGTATTTATAAATCCTCACCTCTTTTATTCTTTTATTTTACTTAACTTAAAAGAACATTCAATTTTAGTTGAATACGTGAAGTTGAAAATCTTTTCAATCTATTTTCTCGAAGATATTTTCAATTCGCGTGTTCCACTAAAATTGAATACTCTCTTCAAGTTAAGTAAAAACAAAAGGTTGTTCACAAATATCTACGAAGGCTTGTTGCGGATCCAACACATAAAGATCAGTTTCAAGGTAACATATATTTTATAACGATACCGGACAAAACCTAAAACTCCGATCACTAAAAGTTACATAAATATAGTAAACTGAAT

>Gm11ciRNA1337

CACTCGAGAGAGAGAGAGAGAGAGATAAACATCGACGATATGGTATCAGAGAAACATCAAATTAAGTACAAGAAAAGATACAAATTAACTTAAAAATACCAAGTGAAGTTTGTTATAAATTCACAAACAATCAACAATGAATTCTCAAATTAATATTTCAAATAACTTTATTCAATTTTTCTTTTAAATAAAATTATTTAATGGAGATCTTCAAATAATCTTCACTTTTTCGAATAAGACGAAACCATTCAAGTTTTATTCGAATATTGGAATGGATATCCCTAACTTAGATTGTCTTCCCCGATTAGAAGTGTTGAACTACTGATTCTAAATTCGAGTCATCCTTCTCCACAGGTAAATATCACATACTGTCAAGGACCTTGTCCTAACTGAAATTTTTTTGCTATGTACCCCACCTGGTTTTTTTATTCAAATCTTGTAAAAAGTTTGTGTGGGTTTTAAACTCTCTCCTGATTCTAAAATAGTACAAAGTACATCTCACTATTAAACAAACTCTTTATAAACTACAAGACAAACGAGTGTTTCTCGAACAATACATACACCAACCTAACTGACT

>Gm11ciRNA1338

CACTCGAGAGAGAGAGAGAGAGAGATAAACATCGACGATATGGTATCAGAGAAACATCAAATTAAGTACAAGAAAAGATACAAATTAACTTAAAAATACCAAGTGAAGTTTGTTATAAATTCACAAACAATCAACAATGAATTCTCAAATTAATATTTCAAATAACTTTATTCAATTTTTCTTTTAAATAAAATTATTTAATGGAGATCTTCAAATAATCTTCACTTTTTCGAATAAGACGAAACCATTCAAGTTTTATTCGAATATTGGAATGGATATCCCTAACTTAGATTGTCTTCCCCGATTAGAAGTGTTGAACTACTGATTCTAAATTCGAGTCATCCTTCTCCACAGGTAAATATCACATACTGTCAAGGACCTTGTCCTAACTGAAATTTTTTTGCTATGTACCCCACCTGGTTTTTTTATTCAAATCTTGTAAAAAGTTTGTGTGGGTTTTAAACTCTCTCCTGATTCTAAAATAGTACAAAGTACATCTCACTATTAAACAAACTCTTTATAAACTACAAGACAAACGAGTGTTTCTCGAACAATACATACACCAACCTAACT

>Gm11ciRNA1339

CATTCATGGTACGGATAACAACGTATTAAACGAGAGTTAAGAGGAGGACACAACACTAAAATTGTAAAATATGAGATTACTTTGTAATTCTCAAATCCAAATAGACGGTTTGAATTCTAACGTGAGACGTTAATTATAAATATCCTTTGGAGTTGCTAAAATCCCGAGTCTTGATACCTTCAACAGTGCAATTGTTTAACCAAACGAT

>Gm10ciRNA134

GTTCATGAACCATGTATTTATTTTTTTGTTTATTTGTGTGGTTCTCAATAAAGTTTTCATCTTTTTTAGTTTTTAAGCTCCTGCTTCATGTGGGGTGCTTTGATATCTCCAGCTCTGTGTTTTGTTTGGATTTTCTCAATTGGGTTTCGTTAAAGTTCCTTCAATTTCTAAATGATCCTTGTTCACTTCAGGCCTAGAATTTGGTTTTTGGTTTGTAGATTTTATTGACTTCTGTTTGCCATGTTCATGTATTTAGCTATTCTGCCCCCTCTAGAATTTTGTTTCCGTGTGATTCTGAAATTTGAAGTTTCCTTGTGTGTCAAATGGATGTGTGTTGACTTTTTGGACTTTTGGCTTTTGGTTTTTTATATGATTCATCAAAAAACTACATATAGGGGTGGATTTAGAAAGAAATTTTGTTGCTTTGGCTAATAGAGAGAAACCTTTTGTGGGTTCTGGCCTATTAAACACATGCACTATGTTGTGTTTTTGTTAATCTTGCATGATGAGAATTATTAGCAAACATGATTGGCT

>Gm11ciRNA1340

GTAATTGGTGCTCTCAGGTTTATTTGCACAATGGAATTTGCATAATCATGCCTTAAGTGTCTGGTGGAAGGAATTAAACAGACTAGCATAAAGATTAATGAACTTGAGTCCAATATATTTGCATAAAAAAGGGCAATATATTTTAGAATTTTGCCAACATTGCCCACACTTCAAGTATAGCATAATAAAGAAAAATAGCAAGGCAAATCTTTCACCCAATATAATTTGCTAGTTTGGCTACACTTTGTTAAAGAAAGCACATTTGACTTTGAATGTATAACAAAATCATATTCATCATGGATTTAGAAGCAAGCAAATGTGTATTTGAGATAGAAATAAAAGTTATCTTTGGTTATTTTCCCTGTTTGTAATGTATCAAGCCCAATACATTTATAGGTGTAAAACCTATGTCCCTTGTCTAGGTTAAGAACTTACAATGAGCAAAATTAGGATACTTTTCACAAGAATTGACTGTGCAAAACTGAGTACCCTTAATGAAAGATAAGGGTATTGGAACAGGGATGTTTGTTGGGTAATTAATTAATAAATAAGCTTCAGCAACCTGGTTTGTAGTTTTGTACTTGTAGCTTTGAATTTATATATTCAAGAATT

>Gm11ciRNA1341

GTACTCATTAATGATCCATCATTCTTACTTTATTTTGCTGTTAAGTTTTCAAGATAATCTTGAAATGTGAATGCTGTTTAGTTTTTTATTTAATGACTTGTCCATAATACAAGTATATAATATTTCTATATAATTAACAGCTACACACACAAACAACACTTTGTATTCATGTTGGAAATTTGAAATCCAAGACTAAAATCTTCTGGTTGATGTGAGAGATTAGAGGATGTGGTTATGATATCTTAGCATTTATATTATTCTTGTTCAT

>Gm11ciRNA1342

CATATTAATATGAAACATCCTCAATTTAATTAGAACCTTAACACACAAAAAATATATCGAGACGTCACAATGTACTACACCTTTAAGTAGACCAACAGGCACAAACGGTATAGACTATAGAGGAACTATTACGTATGTTCTACCTACTTAGTTGTTTCATGGTATAGTCTAAACGTCTTAACCAACTAATTCTAAACTTGAAAGAGACTGAACAAATATCTCCTCTTTTCAACAACCTAAAAGAAAAGCTTCATCATATACTCGAGTTAAGACAAACATGGACAAAAGTAAAAAAATATATACTATAGCAACCTGAGAAT

>Gm11ciRNA1343

GTATATATCACATCATCCCTTTCAACTTCTTCTCTCCCAAACATCACAAATTCACACATGCTCAATAAAATCTCAAATTCAAGTCTTTCATATAATTAAAAATGATTGAAATAATGATGGATCAGGTTATACGGTGAAGATTGACGCACAATATTTTACATTAATAATATGGTGATAGAAAGATTTTTTTCTTTTTACATTATATCACCAAAAAATTTGAAATTAGTGACAAAATAATTTATACTTAATTTTTAAGAAATCCGTCACTAAAATTTAGTGACACCAGAGTTGATAAAGAAAAACCAATTTTTCTTCACTAAATTTAGTCACCGATTTTGATTTTTAGTAGGGAAATTATATAGGTAGTAACATTTGGATATGAGTTGAAAGTGTAAATATATGCATGCAAGAAGAAATTAATTA

>Gm11ciRNA1344

CATTCTTGGTATATGAGATATACGTTCGCTAACGTTGACACTGATTATATCGTATACCAGGTTTTGTCCCGTAATAAGGGTACTTTAAAAGGAAAATAAGAGAACGGATCGACAGATGGGGTAAAACTTTCTGATGAATCTCATTACAATAACAGAAAAACATTTCATGAATTATTCCGAAACCTTACGGTCTTCTAATGTTGTATTCAGTCCAAATTACTAACATCGACTTTAAAGTGTACAAGAAATAATCCTCGATCCCAATACACCACCAGTGACCAGGGGATGTAGGTGTAAAGGGTAAGGGGGTAAAACCACGTTGATCTGGAAGGAAGAGATCTTTGTTTTTCTTTTTATTTATTTAGTCCAGGTGAAAACGACTTGTAAACTGATACATTATAACACTAAGGTACGCTTTAAGTAATAAACCACAACACACTAGGACAAGTCGTAGAGACATCTCTACTAGTAATATATTCTTGAAAGAAATGTACGGTAACCAGGTTGTAAAGAAAAACAACCTTATAAAAAGAATAAGGTAAACTAACGTCTTATGTCATGTTATACACCACCAGAAGAACGACAACTTAAAAATCCTCTATTTAAAAACTTACAATATAAAAGAGAATTTAAATTTAAAGA

>Gm11ciRNA1345

CATTCATACTAAGTACCAAGATGAAAACATCAGTTAACACAGAAACACCAAAAACTTAAATAAATATCTTAAACTACGACCGACTTCAACTCGGATTTAGTAATCACACATTTCCCTTTAGTCTAGATGGTCGACTAACCAACCATCGTTAAAGTTTAAAAGACGTAAAATCAAAATAAGAGACAAATAAATAAATTTGTAGTTCAATTTTGAACCTCGATGTAACCACAAAAAATATGATAAAACCCAGTACGGTACCGGGAACCGTCACCCGAGTCCAAAACATACATACGACTCTAATACCAACCACAAGACTCCATATGATTCAACATTTGTTTTATTATATTATTTGAAGGACGAAAATCTAAACAAGAATTTGAACAGATGACTC

>Gm11ciRNA1346

GTTCTCTGTTTCTCTCTCACTCTCTCTCTGTGATTGTTCTATTATTTATAGTGGTTCAAGTGAAGATTATGAAATGGAACTTTGCATGCCTTGTATTGATTCAGCACAGTTTGGTTTTATATATTGTTTTTTTTTTTTGTTTAATTATTTTGTGTGTTTAAAATGTTAATTGAAATTGTTATATCTTCGTCTGGGTTGTTGCATTTGGCTGAGTTTTAGTTTATTGGTTGTGGTTATACAGACTTTGGTGATCAAAAACTCGAGAACTGTGCTATGTATGATTATTTTTTCTTCTTTTGATTGACCATTAATGATAGAAGTATTATTTTGCTGTTTTTAAAAAATATATATATACTCCTCTAGTTTTGGTATTTTTCCAGTCATCGGATCCTCTGGTTTCATAATTGTTAATGTGTTTGAAAGGTGGATTGCATTTTTTTTGGTTTCTATGATCTAACTTTTGGTCCTAGAACTTGTTTAAAGGGTATCCATAATGATGCTTAGAAGTCTTAATTATTATTATTAGTATTTTGGTTGAATTATGGTTTGGCTTATTTGTTTAGGAATTTGCCTTGTGATTGTGGGATTTGTCTGTGTCTGTGTGACCTGCTGAACAAAAAAATTCATTTTCTTGAAGTCATGGTCTATTATTCTGCATTATGTATGTTGTAAGAATTACCCAATTTACGATTTCATCCACGACGTGAGAATTCTGTAATTAAATCTATGCTATGAATTCCATTCTGATGCTTAAAAGTGCAGAACAAGTATCTAAATATTATCTTATGTAGTTTTACCTCTCTACTTCAATTTTTTCGATTGA

>Gm11ciRNA1347

CACACGAGAAGTTAAGACGTTTCTGTACGAAAATACTCACGTCGTTATTAATTTATAATCTAAAACTACAATTAAGGATCATGTAAAGTTTCAGATAAATTTGAAAACAACGTTTGGTACGAACCCACGTAAAGTCGAAAGTTAAAACGTAAGAGTATTCTGAACTAGGTCCCTATTCAAAACACTAACGTAATCAACAAAATTAGATCTTAACAGAAAACCTTTTTTACTCATAACCAACTTGAGGTAAACAGAAAGTAATTATACAGATGCTGAAACACTAGTAACAAGAAAAGGACAAAAAATAGGGACGGAGGAGGGGGAGAATTTTAATTGAAGACTTTTCTACGACAACTTTGCATTGTACGGATTTTAACCATGAAAAAGGGAACACCGATACTAGCTGTCACTTTTTCTTCTAATTCTAGTTATTACTTCCCTCGTTATTAAATTATTCCGTTCTTCTTAACACTTAAGTAATCTAACAAAGAGTAAGTAAACTAACATGACGGCTTGTAACGAAAAGGACGTTCAACAACCTCGTCTAATAAGAAACCCTATATTGTACTATTTTAAAGTCACCAACGTTTACATATAAAATTATGAGGTAAATATTTCCTATTATTATGGTACCACTTATATAATACTATTAATAGTTTATTTAACTTAACAAATTCGGAATCAGGGAATGTGTTGGGAATATATGAAAACACTGAATACGTGAACAGGAAGTATGCGTGAAAACGACGTTCAGTTATGAAACATGGACATCTATGATCTATAGGAACTAATCCACCTCAAAGATGAAAGTGACAAACAGATCTCAGATCTCTTTATACTTTTAGGTACAGGGTCGTCTCTACGGTACTAAAGTTAAAAATAGTAGTTACCCATAGTAGTAAGTCAACAAAAATAAAAAAACGAA

>Gm11ciRNA1348

GCAAGTGCCAATCTAAGTTGAAGGATATAAGATATCTTTTTGCCTCTAAACTACAACAAATGTTTCCATGTTTTATACATGATTTCATTCACAAACCTTAATTTGCCACCATTTCCTTGGGTTATTGTTGATATAAATGATGAAATCTTGGGATGAGTATTGTAAGCAGAAATCTAGAAATTGCTAACTTACATAAAAAGGTCAATGTATTGTTTCATTTTGATTATTATATACTCTATATTAAAATTCATCAATAATTGTTAATTAATATTACTAATAATTTGCATTTTTAGAGATAAGCTACTTGACAATCTTGATTTGACATTAGTTTCCCATGATAAAAAAAATTCACTTATGTCATAGGTATAGCTATAGGGGGAAGGCATTTGTTGACTCTTTGTTTTGTCATTAGGTTATTTCTCTAGTTTAAGTTGACAAAAAGGAGACGAAGGATTTTCCCAGAGTGATTAAATTTTTATTGTTGTATTCTATATTCTATATGAGGAAGATCAACTCAGCATTATTTAATAACCCAAATTAATGCTT

>Gm11ciRNA1349

CATTCGAAGATCTAGACAGAACATAAGAAAGTAGTCAAAGTACCTAGAGAAGGGGGAAGGGTTCACATGGGTTAACATACGTAAACAAATCATCTAATACTTCATAGTACTAAATGATACACACGTAAACTGACTCTTCCGTTACTGAACGAACTACAGTCTAGCACGAAAACGATAAAAGATAACAAAATAGATAATAATATTGATGGTATTGTTCATATTGGTCTCATTCCAAATATGTTATCACATCATAACATTTTTTTTCTTCGATAGGGGAGGAAGAGGAAAGAAAACGTATACTTAAGTGGAAAAGGTTCAACTACGAGTGAAACATCGTCATTCGTTCTGAAAAAAAAATAGTATTATACAAGTATTAAATACCACTTCAGCCCTCGTATGAAAATAAATAATCGTCAAGAAAACCTTAAATGTTTAATAGT

>Gm10ciRNA135

GTTGGGCACTGTTTCCTTTGTGTGTGTGTGTGTGTTCTGTGTAAATGTAATTGCTGGAATCATGCAGCATGAACTCTTCAAGATCTTTTCTTTGGTCAAAATTGAACAACCCCAGAAAGAAATTTTCAATCTTTTTGAGATTTTGGTTGTCAATTGTGGAAATTGAAGCCCTGCAAATTCAATAGAAGTTAGCTTTTAGCATGGACCATTGAAGAATCCTTTATCAGTTTAGAAATGGACAACCCCAGAAAAAAAAATCTATTGTTTAGGACTAAAATCTGTGCTAATTCAATACCAGTTTTGCTTTCCAGCTTCAGTTCTTTATTCCGCTTG

>Gm11ciRNA1350

GTATAATATGAACTGTCCTTTTTCATCAAGTTTCTTGGCTTATTTATAGCTCATGACAGCATCAATTATTTGTCCTTTTCTTTTATCCTTGCTTGTTACCAAATACCAATAATCATTTTCCAACTTTAACTTGATCATTTTTTGCAGAATGTAATTCTGCTTTTTATGGTTCTTAATTTTGATGGATGTACATTGTTAAAATCCAGTGACATAAATCTATTATTCAATAAACAAATTAATAGGTCAAGCATGTCTATGGTGGGAATATTGCCAATATATTTGTGTTACTTA

>Gm11ciRNA1351

CATTCATTGACAAGAATATTTAATATTAGAAACACAAAGTTATGATGTCTCGTTTTAAACTCCTTTTTTTATATTTTTAACTTTCAACGTAATCTGGTAGACTTAGTCAGTACAATGACTATACGTATAAGTGACAATAGGTACATCATGACTTGTAAATTCGGTATACTTGTTAAAGAATCGTAGTTTTCTACAACTTGAAATCCCTGATTACACCTCAAACGACTTTGTTACGTATCGAAACAATGAATGACACGTATACCTTCTAGTACCTTCAAACTTAATTAAGTTTAATGGTCGTTTAAGATAACTTAACCGT

>Gm11ciRNA1352

GTACTTTGTTTTTAATTCTCTGTTTTTCTTCTGAGTTTTCTATTAGAAAATTAATTACCCTCTTTTATGTCTTAGCAAATTGAAGGGCTAGATTAGCACCCTTGACTGAAACATGTTCACTGGTTATAGCTTCCAGCAATAGCAGAGAGAATGACAATTATTCTAGGCTTTAACATGTTCTTCTGGAAATGTTGTTTCTGTTTGTCTGAACAATGAAGCTTGAATTTGAATTATCACCTCTTTCTTGTTACACTTGCATGCTTTAACCTTGGTTTTGAATAACTTTCAAACTCCAACATTAAGCACTAGTGCCATTTGACATGTCATAACAAATTAATTCTTTGCTAGATTGTGGCTCGTTTGTTCATGTGACATCTTCT

>Gm11ciRNA1353

GTAATTTTCTTGTTCCCTATTTTTACTTCTCTTACCTGTTTTCATTTCTACGAAAGCCTAAAAATATGTTACTTAGTATACAAGACATTAGCTAACCATTCAAATCCTGATAGGTCTGCATGTGTATTGATGGATTATTAAATAGAGATGCATTAACATATTAAAATGAAATTTTTAAAACTTAAAAGAGTAAATTAGAAAAAAAAATATAAATAAGGAACCAAAATTCAATTTTAGCTTGAATATTTTGACTATCAAGTATTTCCTGTTGTGTTAGCATAAAGTTAGCACTTTGGCATTGCATTTTACTTTTGAAATTCTTTACAATTTTCACCTTTAAATTAAGCACTACTAGGAATGCCATACGGTAGATTTTGAAGTCAATATTACAGAGCCAACCAAACCTAGCAGAACTCTGTAGTTTTTCTATTTGGAAGTCAATGTCTATTATATTGGGATGATGAACTTGTGCATTATCAAGAACATCTTTCTATTTAATGTTTTGGCAATTGAAGTTTGAATAGGAGGTAGAATTTGGCAGAGATCAGGACTAGTTCGATATATGTTTAGACGTTTAGTCATTAGTGTATGTTGAAAACAGAATAGAACTGGAAGTCTATGTGCTCGATCTA

>Gm11ciRNA1354

GTAAGGATTAAGAAACTCCAGATTCATTTGGTCTATGATTCATTCGTTCCTTTTATTGTTGTCCCCTTTTTAACTATCTGTCCCGATACAGAAAATGGTAAGTTTTAGTCCTTACACAGTTTTTTGTTGCGGAAAGTGATTTTACACCGCCAGAAAGTTTCATATGATGTTAACGCGAGGGACTAAGGGCATGTTTGGATATAAGGTATAAGTGCTTTTTTTGAGAGCTTTTCTACTATAAATAGAGTTTTTTTTTCCAGTAGAGAAACTCTCAAAAGCTGTCAAAAGGACTTTTAGTCTTATATCCAAACAGGTTCTAAAACTTGCAATTTTCTCCTATTGGGACTAAAATATAGACAGCGGACAATTATAGAGACTAACCAAGTGTTCAATTGATTAAGTTATCTCTATGCTTGTCTTTGGGTTTTGAGATACATTTTCAAATGGGTACTGA

>Gm11ciRNA1355

GTCTCAATTTCCTCTTATAACATAATTGTATGTTCTTTTTTTAATTCGAACCGACTATAACTTGCATGTTTGGATTATATTCAATTTTTCCATAATCTATTCCAAAATTACGATGAAAGTCAAAGTAATAAATAGTTATTTTCACTTTTTCAATTTTCCATTATAATTTTGGGGTCAGAATGTGTTTAAACCCTCACACCATGTTAGTTAGAATGTCTAGAGCACGCTCAATAGAAAACTTTGGTTTGTACTAGATGTGTATCTAACATATGAGATGTAGTATTATAAAAAAATTAACCCCTCAATTTTATATATGCTTT

>Gm11ciRNA1356

GTATGCTTAATGGTTGGTGAATAAGTACTATTATTACTTACTGCACTTTAAGCACCCGATTGTCCCTAAATACTACCTCTGGTCCTATTTATAAGGAACAAGTTGTAGTGTATTTACATTACAACTTGTTTCTTATAAATAGGACTGGAGGTATTAGCATATAACATTTGTTTCCATTATAAGCTCAAACCCTATTTCAAATAGCCTGAAAAATGCTATGATATAGTGGCATAAGTTTGTTTATCAAGCACCTCTATTATGGTCAAGCAGATAGATATGCTGGTTAAATAAACTATGGACTTACTAAGATGACTTCCCAAACACATTTTTATGTTTTATTTACATTATTCATATATTTATGCAGGAAATGGGGAAAATAACAATGTTATTTATTTTGAAAACAAAAACTAAAGTGAAAATCTGACATTTTTGTAATTAGTTGTAAAACTTGAAATGAAAGCATATAATGTGTTATCAAACCGTACAGGATTCCATGGGGATACTAGTCACATATTGCTTGATTTGGTAGACCTTGTTTAACAACAGCTAACTGCTGCATGAATGTCCCTTTCCAGTCT

>Gm11ciRNA1357

GTGAGTTCTCCGCACTCTTCCTTTTTCTCTCTTAATTCCTTAGCTCAATTCGCAACTTCACTTCGCTACTTGACTACAATAAATCCTGCTGCTATCTTGTGATGATAAGTATTAGTAATAATAACTATCGAAGTTTTGCAAGTTACTCTCATGCTTTACTATATTCTGTTTTTTCCGCGATAGATTCGATCGGTGCCGTTACGAAGTTCATTCACCGCACTACTTTACTTTCTTGCTTTTTCAGTTTGCATAGATCTAGATGTTTCTTGTGTTTTTTCCTAAACTGTAGAGTTAGCGTGTGTTGTGAATTGAGAGTGATGTGATA

>Gm11ciRNA1358

GTGAGTACTTTATTTTGGGATATCTTTTATAATTAGTGCTTCCTTTCATATTGTTTGTTATCATATGATACTATGATTTAGGTTTCCATCATCCGATCATAGTTCTTTTATGTCCTTGTGTAGTATGTATTTTTATAATTTGTTAACTTTGAAATTGAAAATCACTGACAATTGTTGGAAATCTGGGAAATGTTGAATTTAAAACAGCAAGGAATGTGATTGAATTTGTTGGATTTTCCTTTTCATCTATCTTGTAGAAACTTCATAATATTATCAAATGATATTATGGTCCACATTCCATTAGTGTGGTATATCCTCTGATATTGGCTTTCTTTTATTTACTATTATCATGTTAGTTTATCTTGCTGGCATCCTATCTTTGTCTCTCTTTTTCTGTGAATTAATTTACCAGTTGAAAGAGTTATATAATTTTTTTTTCTTATTTTTCCTTTTCCAGCTATTTTCTTCAACTACCATGTCCCTCAATGATGTGAAGACATTTATTGTTCTTTACTCATGTCCCTGTATTCTTGTCGATTTGAGTTTTAATTGTGAAATTAAAAGCTCAAAGACAAAATGTTTTTTTTCAAGCTATAGATGTTGCATATACATGGCAGGAAAGTTGATAGATTTCACAAAATTGCAGCACAGATCTAAGCTAGCAAGCTCTCGGCTCTAATAAACGAGATAATGAGTCTTTTTCTTATGTCTTCAATTCTTTTTTGTTCAAAATGGAGAAAACTTGACAGAAATTCAATCTCTTATTCCCCTGCATTGATATATATATCTAATGGAACCTGAAAATACGAATGGTGGCAAGGCAAAAGTTGAAAACAAAGGGCAATATGAAGGTGATATGTCTTTTTAAACAAAAGAAAACTATTATTTA

>Gm11ciRNA1359

CATAGCGAAGATGAGTAAAAGGAAAAAGAGAAAAGCTCACAAGTTAAAGTAAAATAACTAATAAATATATAAAAGGTTACAAGACATAGGTTGGCTCCTCTAATCCCCAAAAAGAAAATGCAGGCCGATTCCCGTGGCCAATTTTTCACAAGCTATGCGTGGAATAGTCTTTAAAATAAATGAATTATTTCTAACTCACTAAAAATACAAAAACCTATATGCCACACAAAATCTTCTCGAATCAAGTCGAATACTTTCACGAATACTGTAAGCATATTTGAATAAAATCAAATAAAAACATTCAAAGTGTGTCTTCAAATTGTTTTACTCAAGTACAAATCCCATAAAGTTCTTCCAAGTATCAGTTCGAATACTTACTCGAGAATACACTATTCTAATATAGTCTATTCCCAAACAAACCTTCTCCAATAAATGTTGAATTGATCTGAATAAAATACTGTTTGTGAACACGATCGCAAACCCTCTCGAATAATTTTATCGAATACTATGCAGAAATTTTACAAAAATCGACTAAAGTTATTCGATAGATCCTACTGAATCCTTTTATCGAATGTTGAAAACACTTTTGTTAAATTAGGTTAAAGGAGAAGTTATCATCTTTGTTGAATATGTATCAACGAATATACTATTCGTGAATGAGGTATACGGGAACTAAATCTACAAATGGATGTACACGGGATTCATGTATTATAAGTATTTGAGAACAAACTATGTTCAAATAGGTTATTCATGAACTAATTTAACAAATAGGTTTGTACAAACATTATTTTATTTAATAAAAAAAGTTATTGATGAAAAATTGAAAACAAAAGAAACGCGTCCAGAAAAAAGAAATGTGAAAGGTAAAGGGGAGGTATAATGTCTTAAAACAACCTCGTCACTCAAATAACTACATTTGA

>Gm10ciRNA136

GTATGTCTTTTTTTCTTTCTCTATCTTATCCTTCTATACTCTTTTCTTTTCTTTGTTTTTTCAAGCCTTATCATTCATAAGCAAAAGCTTAAAAAATGAGTTAGTATGTGCCTGTGTCTATCTAGCTGTGAGCAACTGAGCATATTATGTTCCCTAAAGCCACACATGAACATTGCATTGGTGAAGAATTCTGAGAAGAAAAGTATGTCTTGTTGACAGTTTCATTCAATTTTGTGCCCTTACAATAACTTGGGAAGGAATGGAACACCCTAGGAGTTGTGATGTCATTTTTATATTGGGGGCTTGGTGAGAGGAATTGATGCAATTTATCTGACACAGTGGGCTTGTATTCTTCTAACCTGCTTTCCATATTTCTT

>Gm11ciRNA1360

CACCACGGAGTATAAATTATTTGCTTTCATCAATTTTTACAAAAGTATCTTTATATCTTACACATATAGATTTAAGTCAGTGACGGAAACTCCTGAAAGAGTAATAATAACTTAAAAATAATTATTGAAGAAAATGTCAGAAGAACTCTCTATATTACCCCCTTTAAACTTTGACGTAAGTAAATAATGTCAAAAATATGAAATCCGTTCTAGATGACTTTACGAGAATTCTAAATCAGTACTGAAGAACGGAACCAGTTCTTATCGTAGAGGTGTGGACTTAAACTTTAAAATATTTTTACCTAAATAACTCTACGAACTACTTAAACTCTAATAAAGATAACGGTATAAGTTGATATAGTACGACATTCGAACATTACATGATACCACGAACGA

>Gm11ciRNA1361

GTTGGTCTGTAGGGATATCATGATATGTGCTCTTAGTGGTAGTGTAAATTCTAATTACTACAAATTTTCTTTTGGGCATGCTGGTGTTTGTGTTGTTAATCCCTTGTTTTTGTTTTAATCTCGATGTAAATTCTGTATTTCGTAGAATGTCTTGTAAATTATGGCAATGTAGAAAGCCTCGGGTCTGAATTATG

>Gm11ciRNA1362

GTAAATAGTGGTAATTATGCTTCAATTAATTTATAAGGACTATTTGATGATGATTACAAAATGCATTTTGCAACGTATTATGCTTTATGATGGATTTGATAATTTTTGTATTTATGTCTACTTATTTATCATGTATTATTGACAGTAAAAAGGATGGAAGAAAATGTGGAGAAACCATAGCTAAATCCTGTCTCTTAGGAAAGAAAAGTCATATCTTTTTTATGGATGTAGATGCTATTTGAGGTGCTTAGAGCATTTTGTGTTTCTGGACTTTCTTGTGTGTTGTACTAGTTGAACTTGAATGCAACAATAGCTGATTGCTGTTGCAGAGCAGAATGCATCCAGATTGCTATGCATTGTGACTGCTGATTTAAGTTTCACCTAATTTTTTTCCCTTCACTCGGATTATGTATTGATTTGTTCTTTTAGTCT

>Gm11ciRNA1363

CAAACACGGTCGTATGACTAAAAGAGTACGGAATTCTTAAGAAAGTGAACATTCACGACTACTCAAAACCCTTCACACTGAAACTTCGTGCAACACTCTCAAGACTATTAGTTCACATTATAATGGTACGTCGCGTTAAAAAGGTTTGTTACCTTCCGTAAATTGGATTGTAAAAACGGTACTTGGTACTACAAACTATCACTGTAAAAAAGTTGAAAATTCAAAAAATTTGTTAAAACACATAAGAACCTACATTACGTGTCTATTCTGTAAAAAATGTCTACAAAAAGAGCAATTTATATTTACTCTAGTTTTCTTCGTTCAGAATTGTAATGGTCACCTCCTGAAGTCCCTAGTAAGAACACAGAGAAAAGAATTAAAGTAAAGTACTTTATGGAATATAAAATGGAATGTAATTGTCTGATTGATCTTAGTAGAAGAATAGGTGTTCAGTGGAAGGGAGTCTCTTCCAAATGTCCGAATAGACTCATCTATAAGGTTTAATGTAAACAAAAGGACATTGACCGTTGTGAACCGATGTTCTGGACATTTCGATATAAAGTTACATCAATTATTTACACAGTGAAGGTAGGTAGACCCACTAATATACTACCGCGGTCGGCAGTACTAACAGTTAATAAAATAGACGAATTCTCTCTTAGACCATTATAAAGTTTAAACTTAGAACTTAAAAAACACTAACTGGAATAATACCCATATGTTCTTTCAAGAACACATATAATACGTCACCGGTACGTACTTTTATCTCAAGCATAAACCATTACAAGAGTCTTACAACGAAACTTTTTGTTAAACCTAGATTGATTAATGTGAGTAAGTCAGAACACGA

>Gm11ciRNA1364

GTATGGTGTAGTCTCTCTTCTTCAGGCTCTGCAAAAGTGCATGTAGCCGGTCTGTAGTTTTTGCAGTTTTTCATGGGTGTATACAATTCAAAGATAATCTTGCTTGTGCACGCCACTCCTTTTATTCCAATCTTAATATCATATGCCTTGGCCAAATTTTTTTATATGAAGTAACCTTTAGATATGCATTTTTTTTTATATATATGTTTTGTGTATTCTGTTAGAAGTT

>Gm11ciRNA1365

GTACCAAAGTAGTCCTTCGAGGTTTAGTGATCTGTTTTGCGCTTTTGAAAATTTGAATATATGATTCTGCTTCTAGTTCGAAACTTGTCTTAAGCATGGTTTAGGGTTTGAGCATAGTTTAGGGTTTGAGCATGATTAAATCGATTTTCACTTATCTTCTTTAGTTTCTGCTGATTTCCAAATCTTGTATCTTAGATAGGCCAGTTGAATTTTTATTCTTCCCTAGTCATAACGTTCTGATTTCCAAATCGATGTTTTGTTATGGAGAATTAGTCGATAGTTTCATCTGGCCAACGTATCATATGTCTTTAATTTATTAATTAAACTGAGATTATGGATAAATATAAATATATAAAGTTTTGAAGATTAGGTTATGCAATAGAAAGGGGCAAGGAAGGTTCTATTGTTGTTTGTTTGTAATTGATGTTTCT

>Gm10ciRNA1366

GTAAGTAACTGAGTACTATATTTCATCTTCCTTGGTTATTCATAGATTTATCTGTCCAATGTTAATTATTGACTTCAATTTTTAAACTTATGGTAAGTGATGTTAATTGGTGCTACATGATCAAAATGGTATAAAAAATGTTATTTTCAAGTAGTAGCTGCATCACACCCAAGCAATGGGAGAAGATGGTTATAAAAAATGATTTTATTACATTATTATCTGGATTTTTTA

>Gm10ciRNA1367

GTGATTATTATTATTATTATTATTTGATTTGATAGAAGATGAAACATTGTAGGAGCATCTCTGTTAAGGATAAATGGAGCATGCTCTTTACACTGAACAATTAGGGACAGATGAACTAATTCTGAACTAGCACCTCACTGTAACACATCAAGGTAATGAAAACCTTGTGGCTTGTATGGCATCCAAACCTGCAAGTTTTTATGTAGAAATTAGATTACGTGATGGGTCTCCTGATTGATTTGTTACAAAAAAAGATTTCCTTTTAGGTTGGGATTTTAAGGAAAGCTGAGTAAACCATTGGAAGACTCTTTTTTTCTATAATAGCACATATATTCCTTGCTAGTTGTTACAGATTGCATCACATTGCAGTGTCAAAAGTGTATCTGTACCTTAGTTCTTATTTTGTCAAATGTTTAA

>Gm10ciRNA1368

CATGGGAGGAAAGGAAGTTTAAAAAGTAAACCCACAGTAACAAGTGAAGAAAGTCAACCACCCAAAGTTTAAAACGAAGACGTAAGACGAAAAGACAAAAGAGAGAACAAACAAAACACAGAAACAAGACTTTTGATTTAGTGTTTTCCCTTAGAAGAAACGATAAGAATAGCAATAATAAAATTAAATTAATCCAAAAAGTCAATAACGGTCGAATACTAGTTTCAAAGTCTTAACCTAAAAAAAATTACTTTCCAAACGTAACAGTACACACAACAAACCCAACCTCAATCTAGCTATATTGCGAATTCCGAATTCTACCACGTGAATTTATCCTTTTTTTTTTAGGGAAAAGTAGTTCAAACTAAGACGATTCACACTTCGGCACATCTAATCATCCGACCTTATATGACTTGAAATACCAGAAAAACTATACCACCTCTCTTTTTTTCACTATTCTATTTTCAAACTTTAAACTTAACTTCCCTTTCACCTTCCTTTACTTTCAAATTGATCGCTACTTGATTCACCGGTTCGAACATTTATCTTGTGTAACGATTTTATAGGACGGTGTGTATATATCAAAGAGAATGACGGAAACTTCAAAACTTGTGTGTCATTAATTCTTAAGCCTAATAACCGATCACTCTTTTATTAAGACATCTACGAATAGTACTATCTGGCGAGTCCTATAACACCATAGAAACTTACCAATTTATAAAGTACCATAGGTAAAAATACGAATTAAAAGTAAACTATAGACATAAACAATTAACAGTATGTGAAGTCAACTCGAAGACTAGTATAGAGTACAACCTACCGTGTAACGATCTTCATTAATGATTACATCCAGTAAGTTCTTTACACAACACACCGTGAATCTTCAATAACTGAACAAGAAAGAATTCGTTTAATTCGACAAACCTAGTTACAAAGTGTACACTCTAAACGACGTACGTGAGTTACATATTTGTAGAACAGGA

>Gm10ciRNA1369

GTAAGTGACCCCTTTCACAGATCTATCAGATTCGTACAAAGGACTTCACTTTCCACTTTGTTTTCGATTTTTGGATGTGGGTTTTCGTTTTCTCGCTTTTGGGGTTCAAGGATTGAATGTAAAGGGTGTGTGGCTTTTTAGGATTTGGAGTCACAAGTTTGAGTACATTATGATATGCTTGATAAATTGATTGGATTTGCTTTTTAGTTTAATATGCATCTACTAATAAATGGAATTGTTAATTTGCTAGAATGTATGTGATATTGTTGTAGATTGTTAATTGTTAGTATTTGTTAATTATCTTTTGAGCAAGGTTTTGAAAAATGTTCCTCCACTGTGATTTTGACCGCAACATCAAGGTTTTATGGGTCTCCGCGCCCGCAATTGTGGCCACATCAGCTGTATTTTTCCACAAATTAAAGATCATGACGAAATTGCGGCCACAATTTAAAACCTTGCTTTTGAGTATGCTGTCATGTTGAATGTTAAGTGGTTTTGAATATTAGTGAGAATCAATGTCCTATGAAATCTTGAGTTACATCGTTTTTAAGTTGCTTTTCCTTTTTAAGTTACTTCATTGAGGATTTTGAAGTTTGCACTAAGTACTCATCAGACTTTGATATCAAATAAGGTATGGAGCCTAAGCTGATAGATCTAATTGGGGGGCCAATTTTATGTGTCAAGAAACTTCTGGTTTAGGTTAAAAGTAAATCTAGTAATGTAAACAAGATAAGTCCCATTGTTTCCTTTACTAGCCAAAAACCCCATTAATGATCAAGCCACAAACATGAGATTCACCGACCGCAAATCTTGATGTTGATAAATTCTATGACTATCTCCGTCTCTCTGCTTCAATTCCTCTGCCTCGTACACCAAACACTAATAGTAACTTGTTAATCGGAATCTAATTTGATTTGATATTGCAACTTCCCTAGAGGTGCATGTGCATCACTGGGTTATGATTTATTAAAACTTTTTACGTATGTCATATACCTTATTTGGGAAAAAAATAACAAACCAAATGTTAGGTACCTTGAGAAATTTTGGTTTGCTAATGTATGTACTGCTTTGTGTGGCAGAAATGCTTACATGCGAAGCTGTTAAAGTAAAATAGTTCTGTTAGAGCAAGTATTAGTACTTTTGTATTGA

>Gm10ciRNA137

CAGTCATATACTTGATAAAACATTAGATAAACGATAGATTATAACAATAAAACACGAAAACCGATGAATAAAAGGGGAATGTTATCATTTACATTTTAGGACCCTTCCCCTAAGAGAGATACCCCCATTACCCTCTTAGACCTCCATCCAGACAGTCCTAAACCGTGTATTGATCGTCTTGGGGTCTAGATTACCGGACGAACACTGAACACAAGTAACCCAGTTTCCGGGGGGGTAGTTTGTACTAACTCAACTCGATAATCGTCATACGATAACACTTAAACCAAAAGAAGACGACCACATAAACACATACCGATAATAAAGGAACAATAATTAGTTTACGTGTTCGATAAT

>Gm10ciRNA1370

GTAAAATTTCTGTTACTTTAAATTATATCGCTCTGAGAATAGACTTCTTTTACTACAGAGATTTTCTTCCCAGTTTTATTTGGATCTTCATCACTGGATTTAATTCTTGCACATTTTGTTTCCGAATCGTGCATTTAGCTGTTACAGTATTTGAACTTTTGTCACCATTGCATACTTCCTTTTTAAAATTTGTTTGGGTAACATTAATCTGTGCTGTTCTAGACTAACAATCCATATAGCTGGTTGACCTTCCAAACTACACTTGCCGCCAGTATTGTGTGACTTTGAATGAGCTGAAACCTGGATAGAAAGTGATGATTTATTTTTTTATTTTATGGCTTGTTGGAAATCTGGAGCTTCATGTGTTCAGGTTAGTGCTACATTAGTA

>Gm10ciRNA1371

CAGTCAAAAAACTTCTGGGGGAAAAAGCAAAAAGAACACTAAGACACACAAACACAACAACCTACTGTATCTACCAGTATCTGGCACTACCCCAAGAAACTAAGAAAGATAAGCGAACGAGTTAGTACACTTAAGAAATTAAAGAGAAAGACCCTAGTTCCAGGGACGTTTTTTTCTTTTTTTTACATTCTGAAAACCTAGTCCCCAGTAGCCCATACAACTCGAATCCACTTATATGGGCTTAAGACAACTAAAACAAATCCAATGAATGAAGCAACATACACGAATCAAATCGCCGAATAAATACTACTAGGGTGAACAAACAATCGCTGTCATTCCCTTAGAGAGAAACAAGTAAATTTAAACTTAAAGAACTTCCTCTAACTACACACACACCCTACCTAAAAAAACCAACAATCATAGATATTCCCATTGTAACAACGACCAGTTGTGCACAACGTCTAGTTTAGGAGGAACCTCTACAAGCATTAAAACGTTATCTAAAAGCTTTAACACTCACACTTAACTAACAATCGAACACCAGAAAAACACCAAACTTCTTAGTATAACAAAGACACTACTTGTGCAATACGTTTTATCATGTAGATCAGGAACTTATATAACTAAGAACAAAACGACT

>Gm10ciRNA1372

GTATTGTTTTTCCCCTCTCCATCTTCTTGTCTAGCTACTGACTGACATTATAGAACTTATGGTTATGTATGGTTTAGCAAACTGTTCGCAATGCTTTTGTTGATTTGATAACATGGTAATTGGCTGAAACTTTGCTTGAACTACTATTATGTATATGATATGATATGGTTTTGTTTCCAAATTTGAGTTGTGACCAGTAAATTTTGATGGTGAATCAATGAATGTATTTGATGCCATGACTGATGTTATCCTGTCCCATTAAGATACAGATGGGATTCAAAATACGTGTGTTGTTTGGAGATGCATGTGCTTAATTGTGCAATCTGCATGCAAATTTCAGTGTTTATGGCTATGCAATGCTTGTTATCGATCTCATAAACCAAATGGCAATGGAAACGTAGTCTTTTCGTATGTCTATTACTTGATTTCAAGTCTGCGGTTGTACTCGGTGTGAATAGTGTGAATAATTACTCAATCATGCAAACTAACTTTACAGGGTTTGGGTATGTTATAACCCTGGTTCAAATTGTCTTACCTTTGACCTCTTGTTATTCATGTATCTCTTTGAATTGTTTTAATTATGCCTCATTTAAAAGCAATATTGCTAAGAAAATGAAAGATAACCCACAAGGGACCAGCTATAACATCTCGTGCCACACATACATCACCACACACTCTACCCCCTGAACAACCGCACAATCATCTTCTCAATATAGTAGTA

>Gm10ciRNA1373

CACCCCTTTGAATTAACTCATGTATGAGAACGAAAAACGTGATATTAACATGAACAACCAACGTCGAACGTTCAAACGACATTAAACGCTTTTAAACACTCGAATTTATACTATTTTAGTAATAGTACCACCAAGAGTTGTAAATAAACGCAACAAATCGACTACCATAAAATCTCACAGACATAAAGTGACGATTAAAAAATACATATAAAAGACCTCACGAGAAAGAGT

>Gm10ciRNA1374

GTATAAGATTATGTTTGAACTCATAATACAAATAAATTGGGTGGATTTTTTTACGTTCGAAATGAATTGTTTGAGCTCATAATACTATGTATAAGTTTGTTTGAGCTACGACAAAAATTAAGAATCTTCCCCTTTACTAGTTGGAAGAAAATTGACCATACTGGAATAAAAGAAACAGTTGGCATAATTTTTGGTGGGTTATGTTTATTTTTCAACTGTGGTGCTTGGGTAGAAGATGACAACTACTGTGTGGGGGTAGGCTGGGTGTGGGTTTTTGTCCCAAACCAAAGGGTAGGTTGGGACAAGTTGGAAGTTCATTTGAGGGGTGTGCGGTTGCTAAGACCAGTGTCTCTGATTCTTTCCTTACTCCTTAGTTGAAACATATCATCTTTCCAGTTTTCA

>Gm10ciRNA1375

CATACAAGTGGACGAAAAGAAGGTAAAGTAATGGAAGAGTTGGTCGATTTTATAAAGTCCGTTTTCAGATTACATATGAAGAAGCGTAGAACAAAAAGCATCATTAATTTAGGAACAAAACATCGACACACACACACATTCGATACGTTGACTATCAGTAACGAAATTACAAAGA

>Gm10ciRNA1376

CATTCAAAACAAGTGTTTGTTCTCTTTCTCCAAAGTAAGTAAATACGGATAGTTAAGTGTATAGTAGCGACAAGTAAGTGTAGGTGGATAAAACGTGAACGCACAAAAAAGAAAAACAAAGGAAAACATCGAGAAAAAACATAGTATCATTAGTAGTAAAGGTTGGACAGTTAATGACCGAGTTTCCCATACTCAATAATATTCAATATTGAATATTGGGAAAACGCAGAGGAATTGAAAAAGG

>Gm10ciRNA1377

CATCTCAAAGGAGAAATTAGAAAACTTTGAACTTTACAGTCGATTTATTGGTACCCAGGTAGAATAAAGTCTAAACAATATTGTCTTTTTAGTAAACGGTTCGTTTTTTGTTTAGATAAAATCTACAAAACACTTTCTATAATTACTTTTCTTACTTTAAAATAGAGTAATAGCATCTACGAAGGGTTTTACAAGGACTGGCTTCTTTTAACGACATTTTGTAAAGACAAAAAGATCTAATGA

>Gm10ciRNA1378

CATGTAGTTATTACTTAATTTTGACAAATCCGGATACGACAACGACAACACCAACCACAAACTCGAATCATTCCATGTCCTCCGATATATTTCCGAACTCGAGCTAACGGTAAGTAAAATGTGAAACCGACTCGATGAACTGTGGTTAAGACTATTTCTCTAGCCTTGGAACAACATACAACGTATCCTATCAATGTGATAAAAAGCATAGTAATATTATAAAATACCTGAACACGTATTAAATATACGTACAATGCATCATTCTATAGCCACCTGAACAATAGTAATTGGCACCCTTCCTTATTTTAAGCATAAATATAAGATCACGTAGGACTAAACAACAAACGTTTTGTTACAACTGCGTAGACT

>Gm10ciRNA1379

CAATCAATTAATGGAAAAGAGGAGAAACGTAATGGGAACCGGACCAATATGATCGTTATAATAACAACGAAAATACACAACCATATGTAGTAACGTAGTGTAACATAGTAAGTTATATATAAGAGAAATAATACTACAACTACAAATAAAGAAAAAGACCCATACTGATTACAATGAATTCTCAGACGAACAAATTTATCCATTTTTTTTGTCGTATATACCTTTACTATTAAATAGACCTGACACAAGTTTATGTAATAAATTGGGGAGTAAAAGTAAAAGGTGTATGAGACTGTTCGACTGGGAACCATGTATTTCATACAAAGAAGACGTTGTAATTACACGACTTGATCGTAAAAAGTAATAAAATAAGAAGTTGTAAAGACTTAGACTATTATTTCTCCAAAGAAGTGCAACTACAAATACCTTGAGTCCAATAATACAAACTTAAAACGATGATTAAAATAGATCTTTTTATGTACATCGTTCATGGTTGGTATATAATCAAAGAAACGCAATTAAAAATCAATCGATAGAATGGTATTTTACTTTACGGAGCAAACAACCAGATGGAACAGGCTATTTGTGTAACCAGTAGTCGATACTTCGTACTTGTGGACTATTCTCCTTAGTACAAAGTACAAGTACAGACTGCGAATTACCTGTTATGCCGTATTGTATTGAATAGTAAAGTTTAAGATTGCTCTGTGAAGTTTAAAACAACCTACAAACACAGAGCACATTCACAGGTGTGAATAATCCAGTAGTCACTAGAACT

>Gm10ciRNA138

CATTTGTAAATAAAATATACATCTGACATCACGTTGAAAGAGAAAAACATAGGCGACTGGTGTACGTGGTTCGACCCTCTTTTCCGAAAAATAACAAACAGCCACTAATGTAAAAACCACAAAATCTAAAGTAATATACTTTGAAGTACTAATACAGAACTGGAAACCTTTCTTTATACACTTGTATACAGTATAAACTGATACTTGA

>Gm10ciRNA1380

GTCTGATACAATTAACAGTGTCTTGTCTTATTCTAATCTGTTGTGGGGCTTATTCTTTGCTATGATTACCTTTTGTATTTACTTGAATATCAGAGTTCATGGGGCCTCATCTCACTACATAGAAATAATTAATTTCATATTTCATGTGTCTGTCTGCATCTCTCTTTTCATAGTGGAGGTGGGACAGTATTCCTTACATATTCCAAGTTGACCTTTGTTTGGCCATACAGTGGGCTTATTTAACATTAAAAATGGAGAATTGTTTCAATTCTTCATGAAGTAAATAAATTATGCCAATTCATAATTTTCCTGAATTGAAATTGGTTCAAATTCTT

>Gm10ciRNA1381

GTGAGTTCATTTTATTTTTAATTTGAACACTCCAAGCTATATAAGAAATGATGTTTTCAAGCTTGGGTTATATTTGGGGATAGTTGAATGGGATAGGTTAGTCATAGGGTGGGATCGAAAGCTTCATAGGCTAGGATACCTGCCAATTGCATATGTATGCCTGACTTTCATCATTTGTCTTTGTATTTTGAGAAGGGTCTCAATGTGTTTTCAAGTGTGATAACTTTTTTCACATGCTTTGGGCTGAAGGGAAGTTGTATGAATCATCCTTATTACAATTCATTCTGTGT

>Gm10ciRNA1382

CAAATGAAGAATAAAACAAACACAAAATGGAATGAACACTTACCTCGTATACTAGGGATATCCGTATAAGGGTAAGGTAGAAACGGTGGACTCGACCCCCCCGAGTTGGGTCGAGTCTAGATTAACCCTATAGAACCCGAGATATTTAATCCACATCACTAAACCCAATTTAAGTTCTGTAAAACCATGCGGGTTGAACCGTACTACGGTGTCATAATATTTTTCGTAAAAAACATTAATCACTACTTATTATTTTACGAACTGATCAACTGAGTTTAATTATTGAAAACGTATTGTACTATTATAACGTATTGTAGGGAATCAAAATTAAAAGATGAAAATTTAAAATTCTGAATTGATACATTGTCGTTGTACGGACTAATAATTAACCCGACGAGGTACAGGTCCAGGTTTTTAACTGGAGTGGTTATATAACCCGCCCAAGTACAATTATTTGGGTCAAGTCAGACAGGGTGTATGTGGACAGTCGGATTGAGTACATCGATGAATGTAAAGAAAGTAACGTGAAACTAAGAAAACTTTACATAACCTCGAGTTCAAATACTATATCAATACCATTCTGTCACTCTAAGATATACCGTTACGGTCTAAAACGTACTAGACAAACACGAACTAATAATTCGGGAATATAGGAACTTGGAAGAAAGAAAAATGA

>Gm10ciRNA1383

GTAGGTTTGTTACTGTCGTTTTTTTTTGTGTCTTCTAATTGAATGAATTAAATGGTTACGCGGTTTTTGTCGATTCAAAGAGCCGTGTCGAAACTTCCTCTGGACGCGTTGATTGGAATATTAACTGTTTTGTCTTCTTTTTTGCTTTTTTCGATGCTATTTAAGTGTTTTCCGTTGTGCATGGCCGCGAATTGTTCGAAAAGTGTGTTTCT

>Gm10ciRNA1384

CATTCAAGAATTAAAACTTAAAAACGTAGAATAAATAAAACAAGTTACTATTTTTTTCCACCAAATCAAAGCTATAGGATACGTTTCTAACAGTCGTTACGCCAAAAAAAAAAACATACTCCTTTGCGTATTTTAATTATCGCCTTAACTGAAGTAAACCTCCATAACTACAAATTTGACTTAACAACGAACCCACAAGGTCTGATCACGTAGTCAACCATATTATACGAGACACGTAGATGTGTTTGTTAAGAACAATAAACCGTGGATTTTTAACACATATGTCCCAGACATTTTAGGTTGGAACACCTCACACCGTACATGAAAAGTAACGTTATTAACCGATCTTACAATGCTAACGAAACATACAACGTACGACATCTGACATCAAGTAGAGTTTAAATTACAAGGATAGGTTTTATCTTCGTCCCCTGACAAGAGGAGCGCATACTAAACCAAAAGAGACAACCTTTTTTTTTCTTCGTTTGCAACCGTCCACATCAAATGTCCCAAAACACAACGTTGTCACAGGGTCTCGTAAAGGGTAATAACAACCCTGTTGTCTTCGCTCCAAATTCCCCTCACCTTTAAACCTAGTATTATACAAAATGGTCTATACATAATTGACTACACTATCTTAGTGTGCGACTTTACGTTGAACCAGAAATTCCCGTATATAATAACCGTCGATAAAAATCATCGTAAACGATAGAGAACGAA

>Gm10ciRNA1385

CAGGAAGAAGAAGAAGAAGAAGAAGAAAGTTAAAGTCAGTACCAAAATTTAACACCGGGCGTTGGTGTTAACGCCACCGTCGCAGTTCCATAAAACTGTGAGGCGTTAACGTAGTGCTAGCGTTAACGCCGGGATAAAAGGGCGTTAACAGGTGTCACATTTTCAAAAGACCGAGTGTGGTTGCGCTAGCGTTGACGTTAAATTTTGGTAATAAAAGTGAGGCACTGGTACTCGATACTCTATCTAGGTAGAA

>Gm10ciRNA1386

GTTTACATTTTACATTTGCTGTCCTATAATTGTTAGTTTATTTCTATTGAAACTTGGTGATCCATTTATTAAGTCTGTGGATGAATTGGAATGCAAATTTATTTTGCCTTCTCTCTTTTCAATGAAATTAACATATAAAGTTTGAGTTGAAAAAAAAATGTAATCACTTCTGAACTTGTGTGCAGAATTACTGTCATCTTGATGGTCACCCTATTGGTTTCTATTAAAAAATCTTTTTGGGTTTGCTCCGGGTAATGTGCTAATACAGTAATACTACAAGATCAAATCTTTTCAATAGCTGATCTGTTTCATTAGAGAATGTTGCAATGTGTTTGATGAGAATGGAAGCTTCTTTTCTTATGTACAAATGCCCATTTGATGACAATTCCATCTCCTTTGAGAGCTTACTAA

>Gm10ciRNA1387

CACCCATAGACTAGGCCGGATCTTTCGATGACTAAATAATAAACGGAAATATACCAACTATAAATAAAGTCTATCCATAAATATTCATAAATATAGCTCCACAGAAATTAATCTACAAAAGTACGTAACCTTTATACTTTGATCACTGGACCTTACCTC

>Gm10ciRNA1388

CATGAAAGAGAGAGAGAGAGAGAAAGAGACACGTAGCAACAAAGATAACTTAACGCCAATACCTACGAACTACATAGACACGCATTACACCTTAGCGAATAGCAGTAAGCACACACTGTTCACTCTTAAACAACAATAGAAGATGAACAAGCAACGAACACCGAAAAAAGACACAAGTCAAATCCAACGTTATACCTAAAGTCCCTTTAATTTATATACAAAACAAGGAATGCCTTAACTTCGGTTCCTTGATTAGCAAACTAAGCTTAGTGTTAAAAGAGTTGACAAAACGAAAAAAATTTCTCACAAATATACAAAACTTTAACTTTAAATTCCTCACATCTTAAAAAGGCTTTGACCAACCAACTTGATACTCAAATCCTCAAAACCACCATATTATCCCCGATAGAATACTTTAACCTTATCTTGAATAACGAACTGACATGTTACTTAAAACTTCGAAACAAACAAAGTTAGGACTTCCTGATCGTTAAAAAACCAAAAACCCGTGAAAGAGACGTAAAGAAATCCTGAGTAGTTGATTGATGATGAGATAAAAAACACAATGAAACATAACAAAATAAACGATATAAAAATACTTTATTTGACTTAAAAGGGTACTACTCCATTTCACAACATTTAAACAAGTTAAACCTAAATACGTCATATTTTCAAATCAAAGGATGCCTTCGTAAACAGGAAGGTAAAATACATACGAATAAAACGTAAACTAAAAACAAACCAAGAAAATTAAAAATTGCTCAGTTATAGAAGAAAATTTCTAGGATAACTAACATCAACACCACAGATAAATTAACGTATTACAAAGGGTTTAATTCTGGTACTAGCATACAACACCAGACGGTAAACAAAAGTTCCTTCATCACACAAACAGTGACGAAAAATCTTAATTAAAACGAAGTAAGATATGAGGTAATACGAGAGAATCCGAGAACTACTAGTAACAGACAAGGGGAAAATGTACTGTACTTACACCTAGAGAAGTCCGAACCAACACATAA

>Gm10ciRNA1389

GTAGAAAATATTTAGAAACTATGAAATGAATGTTGTGTAATATTTCCACTTGAATGAATAGGGTGAAAGATTTTCTATTGGACAGCATATATAAAACACAAATTCAGTTTAAAATGGCAGTTCGGAGGCTTAACAACAGCTATGCTCTCGACTTTGCAGTTGATAGATACACATGCAACATTTGTTAATGCAAATACCATTCAACTACCTGTGTATATGATCATACTTGATACTACAGAGAAATTATTTCTTTTGGATAGGCAATAATGGTTGTTATGACTGCCATTAAGAGTATATTGTT

>Gm10ciRNA139

GTATATACTCTGTTTGGTAGTTAGATGCTACATCTCTGTTTACTAACAATCTCTTAAGTTTTTAATACGGAAACTTATGGTACAAAGTCAACATGGTATCCTGGACACATGCTACTGGTTGCCCTTCTCTATAGCATATATATAATAACTAGAAGAAATTAGTGGTTACAGAGTATGTTGTGTTTTCTTATATAATATTCCATGTAGTA

>Gm10ciRNA1390

GTAAGTAGCTTACCTAAATTTGTCAGCTCATGCACAATATACATATTTATCATTGCCCCGGCATGCTCTCTCTAGCTATTCAGTAGATATCTGCTTAAACGTTTTTTTTTTCTGGTCCTCCAAATCGAAGTTTCAAGTAAAGAGCCAATGCTTGCAGTGCTGTTTCTCA

>Gm10ciRNA1391

CATTCACAAAGAAAAGGTGGGGACGGAGTGTTACCTGTCTTAGTCTTAACTAAAATGAAGGTCAGATTACAATAACATCATCTTCAAAATATACAAACCAATAACAATCCTTTTTAACTAAAACGAAGGTTAAAAACCAAACTGATCTCTCCTATTTTATATCGCAAACGAAATTCAAGTTTTTAAATGTAAATTCAAAATGTAGTTTGAACTAAAGTTACATTTTTGTAGGTTTATATTTAGTTAAGTGATTTTTTGGTTAAAATGTTTACGACTAGGTTTGTGTGATATTTACCAAAATTTGAACACCAACCAATTAGATAAGATTAACTGAACGTATAAGACACTAACTTTACCAAAACTAGTTTCGTATTTTACCAGTTACGTTAAACGTATAACGGAAAGTCATGCTTCATCGAAACACATTATTGATCGAAAGTCAACCACGTTTAAAGTTCGAACATACATAAACCAATTAGACTTCAAAAAGACTAACAAAT

>Gm10ciRNA1392

GTGTGTGCCCTTTTTATTTTGGGGGATTATTCATCCATTTTTACCATAGTTAATCTATTTTGTGCCTTTTTTCATTTTTTTCCTTTCCGTGGCTCTACAATTATTGATTTTGAAGCTTCTCTCTTGTTGTTGGGTGCCCTATTTCAATTCCTATGTAGAATGAATGCTTCAGAATGATTCATATCTTCTAATGAAAAAGATTGTTACTTGATATATTATCTTTCATGTGTGGTAAGCTTTTTCTTTAATGTATTTTTTTAATCTTCCATGTATGGTAAGCTTTTTCTTTACTGTATTTTTTTAATCTTCCATGTATGGCTTTGATGGTTTAATCCTGTTAGTAGAATCTTAGGATTAATACTAAATTTCTCCCCACTCAAAGACCAATTTTAGTTCTTATGCATTATAGTTTTAATCATCATTTCAATGCCTGTCAGCAATTTGGATATGACAGCAAATTGT

>Gm10ciRNA1393

CATTCAGGATTAAATCTCTTGACACACTACCTAAACTTTAATCCCAATTACTTTCACGTAAAGAAGACAGGTTTAATCCCAATAGTACCTTGGATTAAAATGCCAGATTTACGCAATTGCAAGTTCCTGCTGATGCTTACACTAATAACATTATATATTAGTAGTTACGTTAATGAAAATAGAAAGTACACGAAATTATTATCTTAATACTAACACAGAACACTGAGACTTTAAAACAACCCAACTTGACAACTTCGATAAGAA

>Gm10ciRNA1394

CATGTAAAATTATGTAAAAAGTGCACAGAAAGTGTTTTGTTTCTAAAAATCACAATAAAAATGGAGATAAAAGAGTATCCCATCATATAGAAACCAAGTTAACCAAGGATATATAATTTTACCGAAGATCGAAAGTCGTAAGAGTACTTTTCAAACTTGAACTTCAGATTATTTACGTAACTATATTAACACCTGTTCCACGATATGATTGGTGGATGTTTTATCTTTATTCTTGAAAACTTTATCAAACTACCTATACAAAGACAGAGATAGGCAGACACACACACGCAACAGACCTATAAACATATACCGATCCCCGGTACAAGTGACAACTTTTACAAGAACAATAAAGGTATATATATATGTATATATACATATATAACATTCAAAGACTAGTGTTTTGTACCGAAACCGACGATTACCCACTCTTGTAAACTCAACAAAATATACCAACTAATACGTTGTAATATGAAATCAT

>Gm10ciRNA1395

CATTACATGTAAAACGTATAAAATTATGATTCTAGAAGAACAAGGTACGGACGTAGATGGAGACGTATCCAAGTTTTGAAGTTTAATTGGAAGACTAGCTATTATCTCGTACTCAAACTACACCGGGACACATAAACCCGTCCGTTCCTACTTCGAATGAAATCGTTAGTAACGTTGGTTATCTTGTACTAGTAGCCTAAGTCAGAACATGTTTCAAAGTAAAGTACACTGTAGTTACATGTTCTAATTGGGAAGAAAAGTCAATGGTCTCTTTATTTAATCATGATTATGTGGATGGAACCATGAGTTTAGAGAACTCCGAAATATCGTACCAGAATGGCTCTTTCTTCTCACACCTCATACTCATCAATCTGTGATTAAAGAATCTTTTTTATAATTACAAAACCACGTAACTCGAATGAAGACTACTACACAAAACAAAACTCAATCACTACCTAGTGTGAA

>Gm10ciRNA1396

CATGATGAACTATAAAATAAGGATTTTAAAGTACGAATCCTCCACGTGTAAGTGATAGATAGGGCAACATAGTCTATAAAAGAGTCAATACGTAAAGATACTACTGATCTACATCGACTTTAAATCAAAACTTTTACCGTTGATTATATGTGTTAATATTCAAATACATGACATAATGTATAATAATTGTCCTTTACTATCAACCTTGGAACTTCTTGTTCACTAAATACGAAGTTTGCTTTAGTTCTGTTTTACTAAATCCAACTCCGTATCTGGGTATCAACCTAACCTATATCAAATAAAACTACAAACCTTACAAAACGAACATATTGTTCTCTCAACTTTCGAAATCATTAACCAAGTAGTACATCACTGTCATCACATCATTCTACTACTAAAAAACCACTTAAAGTTGATGTTGAACAGATAAAGACATAATGACACATTGAAGTTTACGAGTGCAAGTCCTACCGTACAATTTCGTACGAAAAGAGACAAGAAGTAGGAAATTTTACACGATTTTATAAACCCCACTTCCTGAACGTCAAAAAGAAAAAGACAAATATAATACGAATATTTGTACGAACAAGCTAAAACCTTCAATGAGTGGGTTAGGTGTAATTCATCATTGTCATTGTCGTAAATAGTACAGTTTACGAAATAAACCGAGAAGAAAGAACTCATCATACAACATAGA

>Gm10ciRNA1397

CACGTAAGTGACATACGTTTAAAATTCACATACTAACACTCCAATGTCATTTCTTCGGTCATGTTTGGTAATAGATACCCGAGACAGGACGAAAATAAACGAGGGAATTGTTTATAGGAGACTTCACATACCATTGAAGGTTCTCATCAGACACCAACTATTGGTACCTACCTTTTATAGTGGCAAGAATTAACTATTTTATGTTATTGTACCATTATTTGATAACTAAACCCTCTAAGTTGAAGGCAACCACAAGAGAAATCAAGAATACTGAACGTCAACTGAGATTTCGTTTTGGTAGTGAAGTTCCTCGGTCTAGGAAAAAAAAAATTCAAAATTGACATGGTGATTGAGAAACGAGGAAGAATAATTGTTAACGACGTTTTTGTCGAATTTATGCGGATACTTTCAGTACAACCGATAACAACTTGATGACTTGAGAATTAGGGGGTTGTTTTGATTGACTAGACTTCAATAAAAACAGAA

>Gm10ciRNA1398

GTACTTTTACATCCCATTGTGTCTAATATACCACCAATATGTTGTTACATGATTAAATATAGTGCATGATGCCTAGGCTGCCATGATTTTTTTACTTTCTTTCTTCTTTTCGGGGCATACATAGAAGTTAGAACCAAGAACCTGATTAGTTTATTCCCTAGAGCTTGTTAACTGATTGTGTTTTAATTGCCCTCTTTATTTTATCAACATGATAGATATACCTCTAATGAGATTTTCTATCAGATGCATATAGTATAATCCTTCATCACATGTATAATGGTACT

>Gm10ciRNA1399

GTATATTTTACTTACTGTTATTTTTACCAGAATATATTTGTAGAATATCTATTGCTGCATGTGACCTGCATATAAAGGCTGACTGCATTAGTGTTTCAGCCTATGCATGTTAGTAGTGCTTCCAGATCATTCTGAGTTACAATATCTGTGATTAATTGGATGACATGGCAGTCAACACTGACATCTATGTCATGATGTGCATGGATTTTCCTTCCATCAACTTTCTTGAATAGTCATAATTTCAAAATGGGAAAGACAATTGCATGTAATGCTGTTTTTTCCCTTTTCGCATGGAAGGATAGAAATATCTTCAGTTATCTATTACTTGTGTGTGTGTGTGTGTGTCATTCCATGTTTACACTGTTAGTTTTCTCTTTTCCTT

>Gm06ciRNA14

CATATACTTGAACACAACAAACCAATGAAGCCACTCTTTTAATATGTACGGTTACAGCAGGCAATAGTCAAATAATTCTTACCCAAATTGTCAACCAACGTTGAACGTTAAAAGGCTAAATTTGCCTCGTGGTAAAATATTTTGTGGAGAGTATTATTTTCACCTTTACTTTCAGGGAACTTAAACTGTACGCGAAAGGTTTCAGACGGTTTGTACACTCACGTACATATAACAACTGAAACAGAACAGTCGAACTGAAATCTAAGTTATTTGAATTGTAGACTTAGAGTTCATTACCGTGGCAATATCCACGTCGGAGAATTCAACCTACAGTAATGCTCTTGAGTTTCTCCACAAACCAACCCGTAAGACAAATCCCATACAAACCTTCATTCAAACCTCCCTTTCCTTTCTCTTTACAAAATCAAAATATAATTCCCCCAAATTACTAACCAACGAAAAAAAACGGGTTCAAATACT

>Gm10ciRNA140

GTTTGTACATTCCTTACCTTCAAACTCTTGTAGTAAAACCTTTGTTTCAATGGTGGTAATTTTCATCCATGGGTTTCCAAGTGGTCTGGTTTGCTTTATCTGTTTCAGCTGAAGATTTTGTGGAGCATGCATAAGTTAACTGAGATACTTAAATTCATTATTCATTTATTTATCACCATAGAATTAATATATTTATTTGATCAAATGAAATTCTGTCATTATGTGTTGTTTTAGATATTAGCTGACTTGCATGACGTAACGTTATATTCCTTGCATGGTTAAATATTTTATGCAAATTGCAAAATCAAATCAGAAGATACATAATCCCAGCTAGAATTTGTTAAAGTAG

>Gm10ciRNA1400

GTAGTAATTTAAGCTCTTTTTCATTTGATATTTGCCCCCAACAGAATAAATTAAAAAATGGGTTTGTACAAATCTTATCATTAATATCAAGTTGCCAGCTAGGTTTTTGATCTAGTGCCCTGGCTTTGTCACTGCCAAGGAGGTTTAAGTCACTTGGTTAGCTGACAAATCCTATACATTTGAATTCAACAAACAAATGGATGTTAACAGAATGGTGATTCATAATGTCATTAATGTGTATTGGAATTTTTTGTGGTTCATCTTCAGAATTTTTTTTCACTTTAAATCTCACGAGTACTTTAGAAATGATTCGCAGCTATTGATTAGTGATGAAAGTAGCTTATTTATTTAAGTTTCTTCCATTTTCATGTTAATAGTCTTTTACACCCTCCGAACAGAAATATAAACAAAAAAAACTAATTCACACCCATTAAAAGGTACCCTTTACATTTAGTTACATAGGACAAAAAAAGGTTGTTGGAAATAAAACTCTAATTAAATGAAGAGGTATTTTAGAGATGGTATCATTAAATAGGAGAGTTAGTTAAAATTTGCTTATATTTTAGTACATCACAGAATTTTTTTGCTTATATTTTAGTACATCACAATTTTTTTTGCTTATATTTCAGTCTGGAGGTAGTATATGTTGTAATTGATGAAATCACTGAAATTATCAATGATGTTA

>Gm10ciRNA1401

GTACACTCTTTTTAAACCTTTCAGTAATGTTCATCTTACTATTCATTGTACACTTGTTTATTTGTTATGAAACAATTAATGTGATGAAATTGATTATTGAGGAGAATATTGGCCACTATGAAACAGGCAAAATCAGAGTAATATCTATTGATGTGCTACAGAGCTAGTAAACATCACTAGTTAATTACTCTGACTGAATCAATTTCATTGCTATCAAATTTATTCTATTGTAATTTTATCATGTTTATTATATTCAATAGATAGAAGGTTTGGAGGTACAGAGGATAATAAGGCTTGGAAATTGATAAAAAATCTATTTCCGGAGATATAGAGGTTTCTTTAGGTTTTGTAAGATTATTTAGATGATTTGTTGGGTATCATTAATCAATATTTTATTTATCTGTGATATGGTATATGAGAGGGCATTGTTATTTGATTTCCCTTGTATTTTGTTCATTCTTCATTTCTGTTTTAATTAATCTCTGGCTAATATTGGAATGTCAGTCACAGTAGTGGATACAGTCACAATTTAAATATTCTCTTTTGTAATGACTTTTTTTTCTCCTCAACCAAATCAAGATCACACAGTTCCAACGTCAGTGTTTATGGCTTAGCAATATAATGCTGTGTAAAGCTGTCCTTGTACCTTGTTTAGTTGGTAAAGGTTTCGTTAACAGTGTTTATGGTTTTGGGATGCCTGTGTGAAAGTGTCAGTATATCTCTACCACACACTGATGTATTTATGTTCTTATATTTTAATATCCCCATCTTGTCACTGATTTAAAATTTGTAAGGAAAGCATAACTTGCTTGTATGTTGATCCAACTTTCCCAAAATGTCT

>Gm10ciRNA1402

CAAGCAAAGTGGAGGAAAAAGACAAAAATTAAAAGTAATAATACTGCAAAAGTAGCAATGGGAAAAGCAGATTAGAGAGAATTGTCGCACAAACTGGAAACCCCTTTGATGGGAAAACGGTTAAAACGTTCTTATTAGAGGGAAGTAACTAACAATCTAGTATTAAAACATTTTCAAAAACAGAACTTGCACATATATAAGTAAGTTAACCAACAAAGTGAACAAACCGTACACTCTCTTCATGGGAAAACTGTGTATATACTCTTAAACAAAAGAACTAATTAATAATCGGGTCTTAAATACGATCAAAACTAGAACTTATACTAAAATATACAATGTACGGGCTATCCCACAAACTCTTTTTAATGTGTACACTGAGAAAACGATGTGTATCAAAGGAACTAACTAACAATCCGAAATCGAAATAAATAAATAAAAGTATTTAGTTACACATATACCTTCGCAAGTACACGACAGTTAACT

>Gm10ciRNA1403

CACACACACACACACACAAAAAGAAAGACACAATAACAAAAATTGGTAAAACTTAACACCGAAAGACACATTATATGTACACACAGAAACTCGACTAAGTAATTCTAAGACCCTTAGCACAAACCTTAAAACCTTATACTGAAATTCAAGTAACCCAGATCAGGTTCAAACTTAAAACTCCAAAACAATATATATTTCCAATGGTTAAAAAAACGACCGAATATGTTTCAATGTTTACCAGGCTGCCCTCGTTATTAAAAGTTCTTGTAGACTTGATAAAGTAAAGACTCATCTTATCAGTAAAAGTTCATCATAAGTTAGATAACGTATCATTAGTTATTTATAAGTTAACTAACCATACTCCAACAGCTGCAAGCTCAACACTTAAACT

>Gm10ciRNA1404

GTATTCATTTTCACTTGTTGATGGCCATATAACCTATTTATATAATACCGGTTAGGAAGTTGTACATACTTCAAAGGGTTTACTATCTTTTTAGTCCTTAAACTTTTTAAAATTTCTGTTTAGTACATGAACTTTTTTCCATTTGTTTTTAGTCCTTTAACTTTTGTTTTTCCTGACTTTTAGTCTCTTAACTTTGTTCCGTCCTTACTTTTAGTCCCTTAATTTTAGTCACTTAAAAGGATTAAAAGTAAAGATGGAAAAAAGTTAAAGGATTAAAAGTAAGGATGAAAAAAAAGATAAGGGACTAAAAGCAGAAATTCTAATAACTTTAGGGACTAAAAAGATAGTTTACTCTCATCTCAAAAGCCCTTCATCTA

>Gm10ciRNA1405

GTATTTAGAGATTCTACTTGACTTGATGGAAATAAACAATTTAACTACCAAAGGCAAGGAGAATAGTAGTGCCATCAATGGCAGTTTCTTGATTTACATTTGATTTAATTGTTATAATAATTATAAATTTATAATCTCATCCCTTTAATGTACTAATTTTTATTGTAAACAGCTATATGAAGATTTAAGATTATGGCAAAACTTTGCTTCAGATTATGGCTGAGAAAAGTATTAAATCATGATCTAATGTACTGTCATTTACTGGATAACTTGTGACAGTTATTCAACAATTAAACCATTATAATTTAATTATTTATACTATAAGAATAGTTGCAATTAATAATCACTAGATTTGGCTTGAAGTTTCAGCACAGAAACCATTTATATTAATCTACAGTCTACACCTGGACAATAGTCTACCTCCCTCCTTGAAATGCACATTAGGTGTTAATAGAGATTCCACCCTGTGATACATTTCACGTGCTGTGCTTATCTTTAAACACCCTGTGGTCAGCTGGCATATATATGGATTCTTTTTAACCTGTCATGCTTGTGAGTGCAATGCCAAAACCTTGGTGTTTACATTACTATCTAAATATTTTTTTTTTATTTATTTTGATCGGCAAAATATTAATTGTTAGATTGTTGGTTTTGGTTAGAAGGAGGGATTGAACCCGCAACCTTTTCCTCCTTCCCTTCCTTCTTAACCACCCAACCAATCTTATATCTCCACTATCTAATTATTGTAGCCAATAGCAAAATCTTACAATTCTTTGTTATATCTAGAGAGAACAAAGAATTAAACTAAAGAATATGTCACATCTTCAAATTTCTGAGAAGTAAATTCTGTTGGTATATCAAGGAACAAGTAA

>Gm10ciRNA1406

GTAGAATGCTCTGTTTTACCTCTACTCTTGGTGTTGTATTCCTCTTGCATTTTGGCCTAGTTCCAATTTCGTTTATGAAAAACTTGAATTCTATGTGGCTTGTATGTTTTGTGCTATCATCTGAGCAGGAATCCTTTCCCAACTCTGCTACTCTGGTTGGATATGGCATTGTTGATGTGCGATATTCTCCCAGCCCTTTTTCTTGATTATGTTGCATTTGTTGCTTCCTTTTTGGGTATTGCATTTCTCACTTTCTTGTTAGAATCCAATTTGATGTTATATTGAGAACTGCTCAGGTTGATAGGATCTGTCATTAATGTCACCAGCTGAATGTGAGTGTGTTAA

>Gm10ciRNA1407

GTAAATAGATGACACTAAGAGCAAGGTGAATCTAAAATATGTGGACAGCATCACTTTGACAGAAAGGTCTTGCTTGATTGCAAATAAACTTGCATACACTGGATTTGAAATTTTGATACTTCTCGTGCTCTGTCAACTCCAGGAAGCTCTGGCTATAGTTGTACACTTTTTATTGATAATCTTCTGGCTGTAGCTTTTTTCGGTTGACTTTATGAGTGATGATAGTGACACTGTTGTATTTAAGCATTATATTTTGTGGTAGAAAATTTA

>Gm10ciRNA1408

GTACGTGTCTTGTCTCCCTTTTTCATTGAAAATTGAAATAGTTTTTTTTTTATTTTTAAATCAGCATGAAAATGGAAATATTAACCTCATTCAACTCTTTTGTTTACCACCATTGCAGCTGTTTTAGCTAATGAATCTTTTTTTCTCCAGTATGACCTCTTAGAAAAAAAATTTGTTGTGGAAATCTAGCATTTGTAGTGCCATAATGCCATTATTTGGACATTGGAAGGAGAAAAATAATTTCCTTTTTCTGAAGAGAAAAAAGGGAAACTTCTTGTTACTTCTGTGCATTGCTATGGGTTTATTGTTTGGTCAAAACTTATATACAGTTCCTTAGGTTCTTTTGGTGCTTTTCTGCAATTAGAATTGAAGTTTCACCGCCATAATCTACGTAATAAACTTCTGCATTAAAGTCAACATAAATTACTCAGTTGAAACTCCAATTTTGATTGAAGAAAAGCAACTAAGGAACTGTATCTAAGTTTCATCCTTATTGTTTTCAGTTATCCCTTGTTCTCAATGGAAAGAAAAATTCTGATTCTCGTAAACCGAGCAAGCTTGTTGGTGAATCAATATACTTGATTATATGGCTATATCTTTTCACTTTACACTCATTTGTACATGAAGTAGAACCCCATGCCCAGTTTTCA

>Gm10ciRNA1409

CATGAAATGAAATAAGTTTAAGTTTGATAAGATATTTAAATTCTAGACCCAACCATGTCTAGCGTTTAGAAAGATTAAAAGACGACGAACAAAACTGGAATGTTAAGAAAATATAAATTTAAATTAATGTACTGTAAATAGTTGTTTCGGATAGACTTTTAAAATCAAGTGCGATTAAACAGACAGAGAAATAATATAGATTTCGGGATTTAAAGTAGGAGATTTGTAATAGGAGAAAGGATTTACCAGGAGTTTCATTTTTCAATATATTCATTAGGGAGGATCATAATCTTTAGTACGATTCATTAGAGAGTTTTAACTTTTTGGCCAGGGGTTTTGTAATCATTTTATATAGTTGAATCTCCGATTTAAACTATCTATAAGTTATGAACTTACTAATGAACATATCAATATATCAGAACAAAAGAATAATGAAGTGCCTGATAAGTTCTCACACTGACTTACTCATTATGTACTCTCCCATTATGTATTCCCTGGTTTAACTGCCAAATAAGGAAAAGTAAAGAATGGAATCTAACAGTACAAACTAACAACTTTTGGAGCTTTAATGGGGCAGACCATTAAGTTCACAGACAAATACGA

>Gm10ciRNA141

CAAAACATAAAAGAAAGATCTATTGAAATACCCCCGAAATGATTAAACACTTAACCTGTCAACCAACTATGTTCAAGTTTTATTTATATTAAAGATTTAGGTCGTCATTTAACCTTGAGAAGATTGACCGACTGGGATTTTGGTAAAACAAAAGAAGGGCACCTTGTAAAATAGTAAATACCTTTATGTTCTACAATATTTAAGGCAAAGGAACACATCTCCTAATCGCAACGTAGTCGACGGTAACATGATATAATAGAGGAAAATCGGAAACGAATA

>Gm10ciRNA1410

GTAAGAAATTAGTGAGTCTATTCTATTGTGATTGAAATGTAGATAGCGTGCTAACCTCCTCAATGTTTCTCCACTATTTGCTGTTGCTAAATTGTGCTTATAGTTTGATAAAAACATTTGTTTCTTTTCTGATAGTTCTTTGTCATCTTAATTTCCAACATAATTATTTTGATAGATCAAGGAAACTCATTTCTATAGATTGATATCATAGCTTGTATTTCATAAGTACCAACTATCTGAATATGTACGAGTTTTTTTTAAAACCTTCCACTA

>Gm10ciRNA1411

GTGAGTCCACCCTCTTTTCCAATTTCATCTTCTTTTTCACTCTTTGTTTCTTTCAAATGCACCATCCTAGTTAGCTGGCTATGCATTTTCTCAGATACCCAATTGTGAGACAACTGTATAAGATTTATTTTTTCCTTAAAACAATCATGGGTTGGGCCTGTTATTGTAATCGTTAATTTGTTGGGAATTTGATTTCTAATGTACTCATTTCACAGAGTCATTTTGTTGTGATATTGTTAAATGCGAAGAAATTTTGGTCTATTTGGCTTTTCTGAAGTAAGGCTTTGCCATTTTATTGAAAAATGCAAATTATCTATTATCTGTTCTAGACTTCTAGATTTTTTGCTCCATGTACATACTCAATAGGGGATATTCAAATTAAAGGCATCTTACTTTGAAAGTAAACAGTGTCATGTTATGGTATATTGAGTTTGATGTCTCTATGGTCACCTCTTATGTGTTGAATTGTGCTGCTAATAATTTTGACACCCTAAACATGTAGATCTGCTTGAGGCAGTGGAGAGTCTTTTGGATTTCTATTCATTATTGAACATGAATCTTA

>Gm10ciRNA1412

CAAGAACTGATCTTAAAGGTACTAAGTAACATTAGTTATGACCAAAAAACACAAATATAGGGAGAAACGAAGGACTTTCACACCTACGACATCCTTGATTTAACCCTTCTAACCTACATCCTACTTACTTTAAATTTTATAAACTGATCTCAACACTTCGTACAGAAGTACATGACAATTAAAACAGAAGAGAAGGAAACTAGGAGGGGTAGAATTATTAGTACCACGGAACGGATGTCAATATAGAACGAAGTATAGGATTTTAAATATTGGAACATAAGTCATCACATATGTAAAGTTATTTTGGTGACCTTGCCGAACTACACTTTGAACGTATACTGAATGTCCTTAGGACAAAAGTCTAGTGAATAAGATTTTTTAATACTTCAAGTCCCGACGGAACCCTGAACCAAAT

>Gm10ciRNA1413

GTAAGCACTGCAGTTATTCTATCTTTTCATTCCCATTATATTTGTATTACAATATTATATATTGTCATTAACATTTCTTTTCAGATACTCTGTCGTCTAAAGTCATTATTGTTCAATGGTTACATTAGCTTAACCAATACTTTTCTTGACTGTGGCTCTGAAAATCATTAAAGAGACACATCAGTGAAGTAAATTTTAAAGTGGTCGCCTGGGGTTAGCCTTTTCTTGATTAATACTAATTGAAATTCTCACTTAGCAACCAAATATTGGTCATTGTGGCTTATTTATTCTACACTTGTACTCTGCTGTGCAATGTCTTAAATACCAGAATTTCTTTCCCAGTGCAGTGCAAGCACTTGGTATATTAATAGAAGAAAGTTTTGGAGTAATTGGTAACCTGCTGACAATAGGAACTACTGTCTTTACTGAATGGGTCAATGAGTTGAAAAGTTTGCATTATGAGTATTTATTCACTTTATTTTATTTACT

>Gm10ciRNA1414

GTAGCATTTTGCGTCAGTGCTATTCTTCAGTTGTTGCCTTCTGAAATTGCGATTTGTGTGAATGCTGATAAAAAAATGACAAGTCAAACGTGCGTGAGTTTTGATCGTTGAGGAAAATTTAGTGCCGCAAAAACTTGCCAATGCTCGGTTGTCTTTATGTAAATAACTATTATTGTTGCTGAGATACTGTCCCAAATCTCACGGAATGAACCGTTTATTGCGTTTTTTTGTGTTTTGCCTCTAACATTTTAATTGCATTTATTCTGGATTATTGAGTGAAGTTAAGAACTTAATCCACATGGCTATGTTTCAAATTTTTTTAGTACGCGATTCTAATGGTTTTGTATGGGCGAAGTAAAAGAGGCATGGAATGTTCTGACTAACAATAGCGATTTGTGTAACTCCAAAAAATTAAAGAGATATTGGTTTGTTTATTTTATTTTTCATGATTGCAGACCAGAATTAATTTGAGACTGGTTTGTTTGATGATGTAGTTAAACAAATACTAATAGGCCTTGTAAGTGTGGATATGGTAGAACTGGAATATAGAAAAAGGCATTATATACGTGTTAGTTTTTAGAAGCATTCATTGTGGTCTGGTGCTACCAAATAATTTACGGATCCCAATTTGTAGCGATAGAATTGTATTTTGATCAACACTATGCTTTGGCAAACTTCATAATTGCTTTTCAGCTGTAAACTTATCACTTATGAACAAAGGAGTCAGGGTTATTCA

>Gm10ciRNA1415

CACTCAAGAAATTAAGGAAGAGTATGAACGACCCTTATAATAATAACATCTAACTTAATCCTTTAAAATATAAAAGTAAGACTCACTACCATTACGACATGAAGATAGACGTACTAAGTTTACATTTTTACTGTTACTACTACTATTACTATTATATTTAAAGGACAAGTCTAGTAAATCTATTGGAAGAAGTACTTACCAATCTACAAGACGAAAGAAGGATTAACTCAGTTAAATTGAAGACAATTCTTGACTCTTCGTGTTCTAGTTGTGAGACCTTCTGGGAAAAAATACAAAAGATCGGTAATACTTTCGGAAGATATAATGACCTATTAGATGAGAATAATACCCGGATACTGTAATATTACCGACTAAGTTAAACATGTTTCGGATGATTAATTTCTTAAACGCCTTGCATAAATGATGTGGGAACGATTTAATGGTCTTTTAGACTTCTTTTCCTTTCCCCTTTTTCTACTTTTACGAGACTATTAGAGAATCAGAACGATCCGATCATTTCAGAGTTAGTAAAGGGTCAAAAATTAAAGTTTACAAAAGGGGTTTTGTATTCCTAAAGATCAGAGACTAAAAGACTGACT

>Gm10ciRNA1416

GTAATTCTTTCCCCTTTCTTCCTCCTCCAATGCTTCCGATTTGGATCTCTTTTTTGCCTCCTCGTTTTTCTATTTGCATTCCGGGATCTCCTTATGGAACTTTTGTAAAGTGTGCTTTATGTTTCAATTAAAACCTAAATCAATATTCTTAAAGTCAATTCGCCATTTTAATTCCATATGCATAAAGTTTGTACACAATATATGTTATATAACATGTTCATGCGCAACTATTCCTTAGTTAATGCTCCTAGAACTTCAAGTATCAGAAAACATGATAAGTCGTCTCTTAAATATTAAAAAATCCTTCAATTTAGTCCTCAAACTTCAATGAAATACATTAACTCATAGGGGTTAAACTGAAGAATATTCAATATTTTAGTAATTACTGTAAGTAGTTTTGTTACTTTAGAGACTATTTAGGAGAGAGAGTAATAGTTTAAGGATGAATTTGGTGGTTTATTTATTATGTAGTAATTCAGAGTGATACTGATTGAATACT

>Gm10ciRNA1417

GTAACAATATTCTCAAAATTACCTGACTTTGTTTAGAAAATGCCAGTGCTTCTTGTATACTCAGGAGTTAAATGTACTATCATGTATTTGTGTTCGGAGTGATTTAGAAGTTTGTTTTACTCAGTAGGATTTGCCAGCTCCTGCTAAGTGAACTTAGTGATTTGCATGCCAGAAAAGTTGTATCCAGTATCACATTAGATCTTTGAAGTGAGAGTAATGCTGCTTACTCTATTATTATTTTTTTCCAACAAATTGTGCGTAATCTGTTGGGGTTGTTGCATTTTTCTTAAAGGGATTATGAATGATGAATCTTCTAATTTATGCAGTAAGTTGAACAAATTTTATACTGGTGGCAAGTTCAACTGGGGGACAAATTATGCTTCGGTTAATTCAATGCCTA

>Gm10ciRNA1418

GTAAGCAATATTTTTTGGTTTAGAACATTTCTTTTAGACTTGCTCTTATAGTGATCTTAATGCCACACAATAGTTTACATTTTACATTTTCTACAAGATTTTGATAATTTATTCTTCTTGTGAGAAAAAAAAGAACTTAGTTTATAGTGCCAAATTGTCTATTGTGAATTTGCTCTTTGTTTGCAAACATATATAACCTTTTCATTGTATATATTGGTTGGTGAGTAGAAATGCTTCCTATTACAAAGCCTTCAGTGATCACAGCAGAGCATTTTATTTGTTTTTCCCGCACTGCATGATTACCCCATGCAATGTTAATCAAGAAATGGGGCATGTTTTCGTAATGCAGTGACTTCACTTCTTTGTTCCGTTTACTGTTGCTGGTAATGCGTAATGACTACCATACAGCTACTAGAATGATTTCAGGCCCGGCAATTTGCTAATTTTTCTTTAGGTTTTAAAAGTCCCATCTTGTTGTTAATTGATTCAAATTTATAGCAGCATCATG

>Gm10ciRNA1419

GTTCACCCATATGTCCTCTTCATGTTCTCAACAATAAGTTATGTAGTTCTCATTATATCATTATAGCAGTCTTCACTATTTCAATAAGTACTTCTTTAGAATTAGGCCCTGCTATTATATGTCAAGAAGGAAATCCTCTGGAGGTTGATTGATTATATATCAATTAAGGTTTGCTTATGGTTTAAACTTTAATGTAGTTAACCAAACAGTATACTCTACACCTAATCTGAGAAAGACATTTTGAGTTCAAACCCCCTCAATATGTGGTAATGAACATTTATTGAAAAAACTAAAAGAGAACTTGTGTTTCCCTTCTAATGGTGTTGTAACTAGATAAGTGTGCTTTA

>Gm17ciRNA142

GTAAGTAATATCCCTTTCCACTCTTTACTTGCTCCATATGGTAGAGTGTTTTAAAGTTTGTCCAACAGAAACGAAAGTTGAGGGTGAGGAAATATTTTTTTCTTCCCTTCTCCCATCATCTGCAACAGGTCTTATTCCAAGCTGTATATATGTCTTGTGATTGTTGTTAAAACCATTAATTAATTAATTATAAACATTGGAGCTTCATTCTTTACATTTGATGGAAGAAGCTTCTCACTGAGCT

>Gm10ciRNA1420

CACTCATAGATACTCATCCTCATCACACTCGACCACTTCGAAATTATGCAGTCTTTATTACGGACAACAAACAATTCCGTTATACTGTAACAATTTAAATTCGTTAAATTGTACGTAGCCAGACAATTTAACGAACACAATACAGTCTAATAACAGATGAAAGGAAGGATGAACAATACTTAAGATAAAACCAAACCATCTATACCTATACCTAGTGATTTCTAACCCAAGGATTAACATAGGACTTATCTGTATATTTGATAAAAGGAGGAAACTAAAATTAAAAACAGAAAGAATAGATTTAAGAAGGAACGACGAGAGAAACGGAT

>Gm10ciRNA1421

GTCAGTGAACCTTTTCTTTCATTTCCGCTTTGCAACTAAATCATTTCCATGAGCAGTGGAAGTTAGGTATTTGACAGATTCAGGCCAAAATTAGGCAATCATTTGAACAATTTTGTATTTACTGCAAATGCTTAATTTACAAAAAAAAATAAAAATTGCACATGCCAAAGCTCTAGATCAAACAATGATTTGTGAGTTAACTTTGGTTTGGGGAATGAATAGTGGCTGGTTCTATACCTGTTGGCTGTTTTTATGTT

>Gm10ciRNA1422

CATGAAAAAAGTAGAATGATATTACCGTCAGAATGTATACATACGGTTTTTACTCACTTAACAATATCACCCCGAACATAAATAATACAATGCCCTAAACGGACTATACGTTGAATAGGGTTTTTCAGTGAACACGTTGGGTTGTTCCTTAATTCTTAAAGTTTATCTAGATACCACCTTTATTCGTTGATGATGAACATTAGAAGTAACTAGTAAGTCACCTAAACCCATATGACCCCCGTCCCAAACTCGAAACTAACGTGATATCACGTAATACGAATTGTTCATTGAAGACTTCACCCTTCGATTTTGTCAAGTACTTACACATAAAACATTTAACTCAATCTGTCGCAAATTAAAAGACGATTACCTACAGATCAGAAAAAAATGTGACATAGAGT

>Gm10ciRNA1423

GTATGATATTGCGGCTGAATACATGTTTGTTGATGCTTTAGATTTAGTTTTTTGTGCTGGCTGGTCTGTCCAAGTTGCCTTGTGAACTCCCTGCTAATAAAGGATTTCATACAGATGTTTTGCAATAGTGATGAACTATGAGAATGTGATAGGTGATTTTG

>Gm10ciRNA1424

GTAAAACAATCTTCTCCTACGCTTCCCCTTTTATGTTAGAATATGTGGGTCTTCAACCTAGTCACTGATCTGCTAAAATGGCTGATAATTTCAAATGATATAAGAAGCAAATGCAAAAATTAGCACAGGAGATGGTTCGGAAACTGATCTTTGCTTCACTATTTTGTTTTTGAGAAACACAGAATATTTTCTGAGATCAGTTCTGATCTTGTTTTGTTTATCATATTTCTTCAACTATTTTTTGTGTTTATATTGTGTTAA

>Gm10ciRNA1425

GTTAGTAACACAATAAAGCTTCCTACATATCATATTTCCCTCTCTTTAAACTACATTTTTAGCATGTGACTGAAAGGATAACCATACACACACAAAAAAAAATAAAAATACCAACTTAAACATCCAACCTTAATTTTTTCATCATTATCTATTTTCTGCACCTATTTTACACCCATTTCTTTCTTTCTCCCTCTTTCTTTTTATATATTACCATTTTTTCTCTTCTCTTCTTTATTTCTTTCTTCTCTAACCCACTTGCACAATGGTTTCGAAACATCATTATCAATTTTATATGTATGTACATGTATACCAACAGTACCATATAAAGAAGAGATAAAAACAAGTGACATGAAATGAATACTGTTTATCATATGTATGTACATGTATGTCAATTAAAGTTTTAGATCCTTGATGCATGAGAATTGTTAATGATGCGTGTACGGGTTGGATTAATCACATTGGGTTTGTTACATTCAGCTATATGATTGTGGGTTATTAAACTATATTTTAGTGTTGTTTATTTA

>Gm10ciRNA1426

CATTGAATACGAATAGATGAATAGAATATTGCAAGTAATCGGTCACGAACACGTTACAAAGACAGTCTACGGACCGTTTAAAGCTCGATCAAAAGATGGAAACACTAAAATGACCGATAAAAGATTTATAACACTCTCACAATGACGAAACCAAT

>Gm10ciRNA1427

CATTACGAAGAGAGAGACACAGAGAAGGAAGTATAACCTTACTGTGAAAATTGTAACTGATTGTGATTGTTTCATAAAATGAATATTACTTTCATCACAGTGAAGTTTAACGTTACTTTATCATATATTCAACTGGTGACGTACATGGTTGTCTGGTCCGTTTATTTTAAAACTACATGACGATTATGTGATACGAATCACCGGTGATTAAACCCGTCACACCTGGTTGGTTCGATT

>Gm10ciRNA1428

GTATTCTATGCTTAATTTTTGTCATTTAGGGATATTTCAAATATTCACCAGTCATCTTGCGTACAGCTGTCTTAATAATTAAGGATGTTGTCCATATAATTTTCAACTTCATTTTGTTTACTTTTGTAGAAAGAATGGACAACAGAACATATAAATGTTGTTGGGTGGTCATCTTCTCTTCTATTTTTAAATATACTGAATTTTGTCTGTACTTTTTCCATTTTTCGCTTTGTGTGGCTGTCAAATGTGGTAATCTTGTTAATTTGTTTGCTTTTTTCCTTGAAATTTATATTTGGATGTGTGA

>Gm10ciRNA1429

CATCAAAGGAGAGAAGTTAAACTGAAAAAGACACATAAATGGTTTTTATTAACAAAATATTAAAATAAGAATTTAAAACAAGTGTATAACCGATATAAAATACAGAAGGGTATAGTATTACGAAAGGGATATAAAATAAAACAGATTTTAAATAGACAACCGTCTGTTTTTTCAATAGCTAACATTTAAGTATTCTAACCGTTTGTGACTTATTACGTATAGTATTAATTCAATAATGGAACACTAATTAAACGAAATGAGGACATCGGTATAAATTCAATCGAAAATTAATAAAGATGAGACATTTTCTCGAGTATACGTTTCACTCCCCTCTCTATAAATGAGTGTGTGTGTACGAACATAATAGTGAAGAGACGATAACCGAAGAGAATTTTTAAGAAGCTACCAAAGTCGTCCTAAAAATCACCAACACTTCTTAGTACCATGTTTGACCTTTTCGAATCGAAAATTCTTAGAATTTGAACAGAAGACAAGAATAAAACAACTTTTTAAAATAACCGTAATTCACAAAGGAACTGGATCTCATTCTTTGTGAAATTGTTGTAAAAGGGTACTAGTTAACTCTGAGAACTCTTATCTTACGTAAGTTTACACCTATCCACGTTGAATAAATACGGGTATACCAGACCAGACT

>Gm17ciRNA143

CATTCACGTTTCGGTGTGCAAGGTTAACGCAGCTATAATATAAATCCTAATTATAAATGTTGTTTGTTACAATATAGCTAAAAGAATCTACCAAATTAGTAACATAAGATACAAAATTGTAAAAGCTTACAATATAAACGCTAACCTAACTACGATTCATCCTATTATCCTCATTTGTCATCCTATTATCGTCATCTTCGAAGTGCTTCTACGTGGGAAATAACAACAGTTAAAAGTCACGAAAAAATTCCAAGAAAATTTGTTATAAAAAAAAAACCGTCAATTTCCTTAACCAAGATAAGCTTCGATTAACAAGTGAATAATTATCAACGATAACGTATACTATCCCTAAATTATGTACAACTAAGACAACATAAGAAGCTTAGGAATCCAATAAATTGACTACTCTAAAGACGAACGCCACCAAATTAAACAACTATATTCATCCTGGATTGTTATACATAGAGTTAAATAGTAGACCCCCAAAAGAAGTTACTACAACATACGCATGGAAT

>Gm10ciRNA1430

CATACACGACATTCGATATAAAGGTTAAAAAAACTAACCGACACCTTCTAACCGAAAGAATAAAAACAAAAACAGTCCTTCTAACTAAATGTACGAAAACGGTTACTAACAGGGACAATAGGAGCTACTACTAAACATATATATGTGAGGGACAGAATACTATCAAACACTACAGTAAAAAGTATACCTAAGTTTTCGCTTTTATCGGAGAAACGTATACATTTACATTCCGACACATGTTACCGAGGGGAGCATGGAAACGTATCTCTCCTAGGAAAACCGTGAATCAAAAATCAAATCACACAATGTATATCACCTAAGATACCGACTACCGAC

>Gm10ciRNA1431

GTGAGTTGTTGAATTGAATTTATGTGTTGCTCATGGTTATTTTGTAGTTGTCACTTTCATTTGTCGAGAGGCATAAGATGTTTGCCATAACATCTACTAAGTGTGTTTGGGGGTGCAGTTACAAGCTGTGGAGCAGTGTTGTCTATATGTAACTCCAATAACCATTCACAATTTAAAAGTTTAATACAAAGATAATGAAAAAATGATGGGCAGTGTTGTAGCATAAGTAAACCAAAACAAATCGATTCTAGAGCTATGTCCTGCAACAAAAGTTAATTAATCTTTCAATAAAAGTTGATTAATCTTCGACTGAAAACACTGATAGCAATATAAAGCCTAAATTGCTTTGGAGAAACAATTCTAGTTTATGTGGATCATAATTGTATGTCTTCTATGGTGCTTATTATTTTCTTTTCAATCTTCTCATTGCTCTGTATCT

>Gm10ciRNA1432

GTAATTGTCTTGTTCCATTTATAAGCTTACCAAAATTTATATTCAAGTAGTTCATAAGCAGTTTGCAGATATATTATAACCCATTCATAGTCCTTCATCTTCTTTAATATTAGATGGAACTGTCTGAAATAAAATAAAAAATGGCAACAAAATCACCACAAACATTCAACTTTATCTACTGATTTATAAAGTAACCTGCATATTTGGGTATGCTTTGAACAAATCATTTTGATTGTTGAGAAGTTTCACAACTTAAAAGATTTTAATAATTGAACTGTATGGCTAGGCATGCTTCTGCTCAATGTTAATAAATTATACAAGCTGCTTAGCTGCAAGCTGCAATACATTCTCTTCT

>Gm10ciRNA1433

GTAAGTTTCAAGTTTCTCTTGATAACAATTGTTGATCATATAATGAGCTTCTATTCTGTCTAATTTCATTGTTAACTTGGAGCTGCTTCTTACTATTGAAATATGACTTTATCATTCCCCACCTTTTCTTGATCATATGATGAGCTTCTATTCTGTCTATTGTGTTTGTGATGTTGGTTACAAGGTTGTCAAGCTCTCGAGTTA

>Gm10ciRNA1434

GTACAAGCCCTTGCTTTGCTCCAGTCTGATACTTTTCTGTCACTATCCCAATTAACTTTTCTATACTCAAGCCCCAAAGAGGGAACTCTAAGGGGCAGTTTGGTTTGAGAGAATATTTTCTTCTTTTATTTTTAATAATTATATTTACACTTTATTTTCTGTTTTGAAAAGTTTGTGTAACAAATAATGGAGACAGAAAAAAAATTCACTGTTTCCTTTGCAAACTTCTGAATACAGGAAACAAAATAAAAAATTCACCATTTTTATTTTTATAATTAAAAGTAATCAAGTAAGAACAGAAAACGTTTTCTGAAACCAAATGGTCTCATGTGCTTTGTATCACCTTTTATTTTAGTGAGGAAATTTCCAGCAGTATGCTGCTTTTGTAAAGAATCTATTCGTTAAATTGCATGCAAATACGTTATGAGCGACTTGTGTTTGAAGATATT

>Gm10ciRNA1435

CATACACAATCATATATACTTTTATAGTATCAGAAACTAATAGGAAGAAAGGAGTAGTAGAGAATCCTAACAAATAGTATAAAGCAATAGAATAGTACAAAACAAAAGGATTAATTAATCGATTCTAATACAAAGTATATTTATCCCCAAACACGAAATCCAAAATACTTGTGTGGTGATGTAACATTATGTAGAGTCAATTTATAGTTAATCACAAGGTAAGAGAAAGTAGAGAGAATGAGGGAGAAAAAAGGATTTTGAGATAAAAGTACCATAAACTCACTATGGTAGAAGGCACTGGATAGAACAGTGACGTGACAACGATTCGAGGGATCTTAAACCTCAAGATGTGAATATGAAATTAAAATACCAAGAAGGCAGTTTAATTAGAAGAGTACACAGTTGGAAAACCGTAGAATTCCGTAAAAGAGAACGAAATCGACATAAGGAAGTACGTTCACACTGGGAGAAAACGAACATGAGAAGTAAGACTGACGGAGCAAAAAGTAGAATCGGAGACGGCACGTAAATAAGGAGATTAATCAATCTTAATACTAATCATATTTATTCCCAATCAGGAAATCTAAAATATTTGTCCGGATTTTAGTATATTAAAGTTGTTATACGAAACGAATTGGACAAGACACCAAACTGAGTCATCACAAAGGATACAATTTTCAAGTAGTTAAAGTAAAATATTTTGACCCTTTTACTTATTTTTCCTTAGCATCCGGAATAGGAAAAAGTACACTTGGTACCACATTTTGTTTAAAATGTCATTACGAAAGTTCTACTCATACATTACGTTTACCTCGTAAACAATTTTGCTGGAAAATTC

>Gm10ciRNA1436

GTGAGTGAGATGGTTAGAGTTTTTAGTATACCAAGCATCTGTTTGACAAGCTGTGTTTGAAAGCTTCGGTTTGTATTTTAGAAAAGGAAAAAAGTTGATATGGTAGTTAAAGTGTAGAACATTGAAGCTATTCTTTTTTCACTTTTCTTTATGATTAACTGAATGTTGACTAGTTCCAAAACCAACACTGTGATCTTTATAGTTGAAAATTGGAATAGGAGCCTTGGTATTATTCTCATGTCAAGATTCTGAATTTAGTTTCTTGAGCAAATTATAGTTACTGACACACTGAGAAATTCAGTATTGAACTTTCTATGGCAATCCTGAAAAAAAAAAAAGAGTAAACATCAAAAGTAATTGATACAATACAAGTGTCTTCAATTTTTTCCTTGAATGACATATACACCAAAATAGTTCACCAATGTCTAATTTTGATGTCAACCCTATTTGATGAAAAAATGACATTGGAAGACCATTTTGATGACCAAAATGTTTACTCAAAATAGAAAAATAAAATACCTTGATTTAGGCTTTAAATAAATGTATGCTTTTCATATTCTAATTATGGCACAAAAATCTTATTTATATGCCCCATTTATAATCCAAAGCCAAAATCTAAGGTTATTTCTGAAAATGAAACTAAGATACAAGAGTTAATTTTTCAGTTTGAGCATGGCTCAATTCCAAAGATAATTTTTTTTTTGTATTGTTACTTGTTTCTTTCCTCTCCTTTTCAAATCTCACTCATGATATTGTGACCAGTGACTGCCAGATCAAAGTTACCTATACTTTTTACATTCTTATTTATCTAAAGTAACCAAGAATTAAACTACCAGTAGTTGTTTAATTCTTTTTCTGTTAAATGTTCCTGGCTATTTCTG

>Gm10ciRNA1437

GTGGGTAACACAATTGCTTCCTCTTCTTTGATTTACATGTTAGTACTAAATGTTGCTGACTTGTCAGAGTAAAATTAACTCTGACAGAAGCTCTCATGTGGGAGCCCTTTGAGCAAGAATCATGGTCAAAATGCAGGCTTTTTGTTCATATAAATTTGGATACCATGTTAATAAATATCTCAATCTAAAAAGTTTGTTGTTAAGTGAAGAGTTAAGATGGATTTTGATTGTCTTGAACAAATTGTTTATACCTATTACTTTTGGTGTTGCTTCCCAGAATGCTTTCTTCTGAAAAAAATAAACATCAACATGTTATATGAGGTTGAAGTAACCAGTTTATGTGATGGCAGCACATGATTAAATGTGTGAAACATTTTCTCTCAATATGAATAGGCATGTGGATGATTTGACCATTTGCCTATCCAACTCTGTAACTCTTCCTTTTTTTAGTGGAATATATTTTTAATGAGAAATTTTAAGCATGTATTCCGCTTGTTAGTTTAGCTAGAATAAATTTATGTCTTGACATTTATGGAGAATGGGCAAAGTCTTGAAGGAACATAGGCATATTCAAAATTTTAA

>Gm10ciRNA1438

CGTCCAAGGGGGGAAGGAACAAAAAATGACTTAAACTATAATCAGAACATAAACTTTCCGATGTCCAATTTCCTAAATTAGTGACCTAAATGTCTTAGAATCTTAGATGTCGTATGAAAAAAGATTATCAGGGTGTAATGGTACCCGAACGGGAAAAACCACAAAAAGGACCACTTCAAAAATCAAGGGT

>Gm10ciRNA1439

GTACTTTGCTTAGCCACCTGTTTCTTTTCTCTGTGTTCTCCTCTCCCTTGGTTTTCACTTTTCACTTTATTGTTCTTTGAAAAAAAAACAAAATCCACATAAAGGTGCTTGTGTTTTTAAGTTGAGAGGTTAATTTGTATCTCTGAGTAAGAATATGTAATAGGAATGGTTTCTTCAAATCTCACCCCACGTGCAGTTTGCATTTTGGTTTAAAGTGAAAAGTTCAAGATTAGGATACTCAGGATTTGGAGTTGCAAAATTACCTTAAAATTTATTATAAAGGCTTCTCATCCTTTATGCTCTTGTGATGTGTTTTGA

>Gm17ciRNA144

GTGAAAACTTTACTCCATCTATTGTGTTTTGATCAGGTTTATACTGTTCTGCTCATGGAAGAAGACAAGAAGACATAGGTTCAAGTTCAATCCCATTAATATGTGTATGTATTAGATGAGTAAGAGGAGCAAATCGTGGCCACACAATTAAAATGAATGGTGAAGATTAGGTTTTATTTGAAATGATCTTGGTTGTTCATGTTTAGTTATGTAGCCAAAACTAACTCCTCTAGCACTCATACTAGAAATGTCCCATAAAGAAAGAAAGAATGAATTGAAATAGGTTGTATGGTTAAAAAACTTCCACCTTTATGTCATCTTATATTTTAGTTTTCTATTTTTGTGTTAGGTTTATGGTAAAGAAGTCATCCATA

>Gm10ciRNA1440

GTATATTGTTTAACCTTTGGGCTTTCTATACTTTTCATTTGATCTACGGCCATAATTCTATTATAAGAAAAAAAATGTTCAACCTTATCTTCGAAGTGAATAAATAGGACAAAAGAAGGAGGGAAAAATATTAAATAATTGGTTAAGGTAAAAGAAGGAAATGATACAAATTAATTGGTGTACTATATAAGCTTATTTTTTATATCTTAAAGTATACCATGTTGCCACATTTTTATAAGCAAATTTACACTTATATATTTGAAGGAATTTTCTAGACGCAAGCAAAATTTGGTGAGAAAAAAGAGGGGATGTAAATTTCTTCATATACGTTATCTTTCTTTTGTATCCATACATAATCATGATCTGAGTTGTAAAAAAGGAATGTTTCTTTGTGCTTGAAAATTGAGACAATGCACGGGTTGCATTAAATAGAAGGTAAACAAGCAAGATAACACTCCAAAAAACAGCGTCCATAAGAGTTACATGACAAAAGAATCAAATAAAAAACATGGCGGGTACCCTTCCTGTCCCACTTGATTGATTAGGTTTGGAGTTAGGATCAGATCTTACACAACTTATAAATTGTGAGGTGGAGATAGGTTTGAGATATAGACACTCATTTAAACCACTTCAAACTCAAATATGTCTCCATAATATTTAAATTATAATAAAGTAAACCATAGAGTTAAGCACAAGTGTTTGTATTAATAGTACTAGTTGGTTCAATCTTTTACTTCATTTTATGAATTATTTAATACAAAATTACTTGACATATGTTTTATTTGAAGATAAAGGGTTGGGTATCTATTTGTCATCCTTACTAGCGAGAGTGGCAGGGGTTCGGAACATTATGTGTTTTCTGCAAGCGGCAATTAAGATGTCCAACAGACACATTCAATCAGTCCTCTGATTGCGGGGACCATATTAGCCAATGATATAACTTTTCCTAGCTTATCAGGCCTACATAAATTAAAAGAGTGACAGTTCAAACCACTTTGTCAGCATCACTTGTTCAAAATGGTTCATAATTTCATTTTCTCATATCTAAGTTGACCATGATCTTATATATACTAATTTAGATTGAATTTAACTCATTAGGGTAACAAAGTGAAAACGGCCCAAAATAAATTGCTTGTTCTTCGAACCTCCGCCATAGAATATATTAATACATCTTCTGAGCGAAAGATTATACTCATTACTCTCCTTTTTTTGAATTTAGTATTATAATTGGGGAATAAAAAACCCCACAATTTTACTTTGTTTTCTGTATCTAAAAAATCTTATTTTTTTTAACATGAAAAAATAAAGAAACCATTGCAATTGCCGGAAATACTAGGCCTACTAGAGGAACAAAAATGGAAGGAAAGTTTATCATAAAATGGGTACCTCGATTTACTATTTGGGTCCAGTGGTAAGGATTGGAGTAGATGGACGATGACTGAAGTTTGATTCTCATTGTCATCATTGTAAAAAAGAAAAAAAATGGTTAAGGTAATTTTGACATGTGTAAAGAAGGTTATTGGAAGCATTGAAGAACAAAGTAAATTGCATGATAGCCCTATGAACAAGGATAGAGGCATTCCAGGAATGATAATAGAGGAAATTATTAAGAGGGATCTTAAGGTGAATAATATCTATGAGAATTTGGTCTTTAACCGAACCGAATGACATTGTGATCCATGTAGCTGACATTACCTAATGGGATAAGGCTTTTGTTGATGATATTGATTTGAAACTATTTGAATTGCTAGTGATGCTGTTCCCTT

>Gm10ciRNA1441

CAAGAAAGAGTTAGTTGTGAAATGAAAATAAATATTTAGTACAAAGAAGAGCACGGAAATAGACGGGTTAAGCAAAGCACTATTACCCATATTAAACCTAAATCTGTATTTGTATATGTACCCTAGTTCAAACAGAAAAAGGAGGTAAAAAAGAGACCCAAAATATTAATTCATACGTGAACATAACCCAAACAAGTTACCCTATTAAATTAAGTGCGACTTTCAACGATCGATTAGAATGAACACGTTAAACCGAATTAAACACTTGGGGTGGACTAATAAATAAAAAAACAAAACTAGTTACTTATTCATAAACGGTGGGGTTAAAAGAAACACTGTACAGTAA

>Gm10ciRNA1442

CACGAACGGATACTTTAGTAGAGAAATAGAAGTAACTTGACGAACGTAACACCCTATTTTTGTGAACCCTCCAACTTCTCTTTATGAAATATCCTATCAAAACTAACTTAATAACTTTACACACGATTTGACGATCATTCCCTTATACTGTAAATACTTACCAACCGTTACAAACATTAACGAAGACTACGTTGAACAGGAGTTTCTTA

>Gm10ciRNA1443

CACTTATTTTAACCTTTAGAAAGAAAAAGAGACAAACTAAGCAGTTTACTTTAGAAAGTTTTTAAACTTGAAAAAGAATGGGTACATCTTTATTTTTTCTAAAAAAAAAGCCAAAAGCTAATAAAAAACGCACTAGTCCAAAACCTAAGAAGCACCGTTCGCTTAATCTCAAGTCTTTAAAACTAGGATATAAACTACTACTGGCAAATAAGTAAAAATTTTTTAAAATTAAAATAAAAGACTATAACTCAAAAAGCAACCTTAACAAGAAC

>Gm10ciRNA1444

CATAGACTGAAAATCTAGGAATAGAGGGACGTGAACTTTCAGTGTAAATCACGAACAAACTTACTCACATACGTTTCAACTAAAACCTATTTTAACTAAAACTTCACTACACTAAATATAAACCTACAGTACAATCCTCATTTGAATCTTCATATTTAAAAAAACTAGTTTATGTTTTTGATGAGTTTCAACAAAGTTGAATCTTAGTTAAGAGGTTGTACTTTGGTTTGTGCGAGAACCAATAATAACTAACAAAAAATCAATTAAA

>Gm10ciRNA1445

GTAAGTTTTTCACATTCCCCTTTCCTTGGCCATTGGCGAGTTGCATGCATCTTAGTTCATTTGCAATGCTCTCTATAGAACCTGGGAATTATAGTTCATTTTGGTTTTTGCTTTCCTCTTTATTTTCTAGACTCTTGTGTACTAACTTTTCAAGATCCTAACACAATATAAATGTGGCTCTAAAAATATAAGAACTAGTTTATTTTTTTTGGTTTGCTCATTCATGCAGAAAGTAAATTTTATTTATTTATTTATCTTTATGTTTCAGTGGATGTTGCTCTGGCTTA

>Gm10ciRNA1446

CATTCAGACGTAATGTTCGTTATACGGATATTCGTGTTGAAAAGAGTGACTTTACATAAAGAGACCTGGTTAAATACACGATTTCTACCCCATATATGAACCATCCTACCTTTTCTTCCTATATTATATTTTCCTTCCCCGACTTTTATTTTAACTGTTAAATAAATTGATATAAAAGTTTATACTCTTTAACAACATGGAAGGGAAGTAAAAAGGTTAAAACGTAATGATTTTGTGAACATTAGGAGTGGTATTGACCATAAACAAAGATTTAAAATTATATGTACTACAACGGGATCACAAGTATAAAAAGAACTTAAACAAGAACAAAACCTGAATAAAAGGACGAGTAGAAT

>Gm10ciRNA1447

GTCCAAAATTCGTCTTATCATGAATGGTTTCATAAAATTACATTCGGAACTAACATTGAACTCCTATTCTGTTTAGCAAGGATACTTTCCTTCTTTTACCCAAAAAAATATATCATTTCTTAAACAGACTTCCAAAATTTATGAATTATCATTTTTAATACCTCTGAGAATTGGCTTTTACTGTCTAGAAAGTTCAATAGCCGATGCTGAAAATCTCATCTATCCAATGTTTATCTGTTGTATATTTTTTTATTAAATATTATTATTTCCCACATTTCACATGTTTAGTCA

>Gm10ciRNA1448

GTCCAAAATTCGTCTTATCATGAATGGTTTCATAAAATTACATTCGGAACTAACATTGAACTCCTATTCTGTTTAGCAAGGATACTTTCCTTCTTTTACCCAAAAAAATATATCATTTCTTAAACAGACTTCCAAAATTTATGAATTATCATTTTTAATACCTCTGAGAATTGGCTTTTACTGTCTAGAAAGTTCAATAGCCGATGCTGAAAATCTCATCTATCCAATGTTTATCTGTTGTATATTTTTTTATTAAATATTATTATTTCCCACATTTCACATGTTTA

>Gm10ciRNA1449

CAAGAGGGTAGAAGTGAGAGAGATCGTTAACGAGTACGAGGAAAAGAGAAGAAGCAAACGTAAAGCTCTAAGCATCCTTTTTACTACAACAACTGCTACGTCAAAGTACAGACAAAAGCAACAGTGTAAAGGCTTAGGCTTGCGCTATCATCACTGTTATAATTGTTGCTAAAGAAAGGAGTAAAACAGCACAATTTTAAATAGTGGACAGAGAGCAAAAGTAATGA

>Gm17ciRNA145

GTAAGTAGCATAATCTTTCTGCATTAAAGTCTTAATTAGATAATTAGAAATATTTCTGTATTGCTTCTAGTAGTTTGGTTGGCCAACCAGATTGTGCCTTCAACTCAGATAATTCCCTTGACACGATTCTTAGATTCCGTTCCGTGGAATCTTAATGATAAGGGTATAGCTGAACCTTTGATTGCCAGACTCTGGGCACAATGGTCAGTGCCCATACCATTAGGCTTGGGGTTATACTGGTTTCTGTACAAATCCTAAAAGAATTTAAAATGTGGTTTTATCCTCCCCCTTTTGTAGAGACCTCTGCCCAGACTGTAAATACCCAAAAAAAATTTGGATCTTTACTCA

>Gm10ciRNA1450

GTTTGCTATTTATTTATCATTATGATTTAAATGTTAAGGCATACATAAATTATTGCTCTATTTGACAATTGACAGTTAACTTTTGTTTGTAGCTTCAATATGTCTGACAAATAAAACTTGGGTATAGGATTTTTTTTTCAACAGAAATAATCATGCTACCTCAGTGAATGAATGCATGCTTATGAGAGCATATTTGCAGTAAAGGTTTAGTTTGAAGTGTTGGACCTATATTCATGTTATGGGAGTGTGTAAGTTGAAGTTCATAACTAGGATCGATCACTTTGTATCT

>Gm10ciRNA1451

GTAAATTATATTATTATTTATTAAAAATTAGGACAAATTGCATTCATACTCCCTAAACATAAGTGACATTGCACCCAATATCCTTCCCTTTTCCATACAGGGACTTTAATTAACAAATTAGGATAGGCTTATGTTAATAGAAAGAGACATTGTTATATCAAGTAATTGTGGAAAGTTTGCGTTGGGTCAAAAGAGGGAGAGACATCTGGTGCAATTTCACTTTAGTTTAAATAATTTTCCCTAATAATTATTTTATCCTTTTTGTTTTTTTGGTAAACATTTAATTTAGATGGCCTGATTA

>Gm10ciRNA1452

GTAAACAAACCACTGTATAAGACTAAACTAAACTATCCTATAAATGTAGTATACCTTGAAGACATGATATGATTATGGTTAAGTTTATAATTTCAGTTTAAGCACCAGATATAGAGTCTATGGTGCAAGCTATAGATTGAAAATGTGGACTTTGCTTATCTCCAAATGAAATTCAATTATCTACTA

>Gm10ciRNA1453

CACGACAATATGATAATAAACAAAGTACTCTTTATTGTTTACAATCCAATGTTATGAAGACAATCTTTCATGTGAATATAAAAACACCTTGGTCTGGACTGTACACTAACGAAGAGACCACGGCCTTCCCTTCTAATATCAAATATAGTAATAAAAATTAAAAATTATACAACGTGTACTAAAAGAAAATCGATAGTAATCGAATATTTAAGGACGTTCCCCATCATGGAATCTGATCATAAATAGAGAGAATAGAATCTTTATAAAATACAAACACATACTACTTTGTTCGTTAAAGCCGATAAAGAACAAAAT

>Gm10ciRNA1454

GTAACCACAATTCAAAATCCTTACGTACCTAATTTTGGTAGTCTACCATGTACTGTTTACTTACTTTACTAAGTGCCAGTTAAACTCCAACACTGTTCCACCACATTTTAGTTTGGTCCTCGTTTGTTCTTCTGTGTGTGTTGTTGGGTATATAATATGCACCCTTTCTGGCATCATCATATGCATCAGATCATGCATGTTCTACTTTTGTTTGACCTCTACCTGTAATTGGACCACTTCCCCTGATTTTTTGGTGGTCCATCCACTACCATGCCATACCATATCCAAACCAATTCCATATAACATGTACCCCATGCACAAAAAGTACCCCTTTCCCGTTACTGTTCAACATTATTATCATAAATTCAAATGTAACCATAGTGTCCTATCTTATTGAAATTGAAAAATAAAAGCCTATTTTATTTGTCTCGGTTAGATTCATAGAGTACGAGGGAACACTTTATATTGTTTTGGCAAGTATTGAAAAAGACGTATACGACTTATTTGTACCCATTTTAATTAAAGAATAGAGCGATTATTATACTGTGTGTTGTGCTA

>Gm10ciRNA1455

GTAAGACGACGAAGTCAGCAACCTCAACCAAAATCGTGATTGATTTCAATAACTGAACTTGATCCAAGTTAAATTAGACCCAAATGTCTCGGATAAAATTTTGGATTCTAATTTTATAATAAAATAATGGTGATTAAGGGAAAACTCTATTAAAATTAGTCAACCAAATTTTCCTACAAAAATTATCATAAAATTAATCGTGCACAAAATCAATCCTATGAGTATGGTTATAAAAAATAGTCAACAAACGAAACGGGTCGTCACTTCCTTGTTTCGGCGATGTTGTTTTTTGACAGTGAAACCCAAATTAGATTATTATTTAAGGAAATGTTAGAAAGGATCGTTTTTAATTTTTTATAAGAAATCATTGTGATGGATAAT

>Gm10ciRNA1456

CATTCTGTATAAATGTTACTAGATAGAAAGTAAATATATCAATAGAATGGACTAAATAAGTAATATAATCCTGTGATATCACGGGATTTATAACTAAGATTAATATACGGTTTGGAACCGACAAGGGGATAGGAGGTAAGTAACTACCAATATTTGAGAGACAAATATACGTTCTAAGAACGGGACGAGGTAAAAATAAAGAGTATCACCCAAGCGACCCAACTTAGAATAAAAGTTGTTATCATCCTATAGACAAAAGGATTTAAACATCAAACAGTAAATTTATCCGCCTTTTCAAGAGAATCTTACTATCTGTACAGATTTTTTAACATTGAATACTAAAACCGAATTTTAAAATCATAGTATTTTCTCTTTTGAATGAAA

>Gm10ciRNA1457

CATTCCTTTTGAATTATTTAAACACAAAAGGAGAACCATACTTACAACTTATGATAAACATAAAAGATCAAGTATAATGACGAGTAAAATTATCGACTGTACTAGACAGTACCACCCTTGTATACGATTCAACTACGGAGCCTACCAATCAAACTGATAAATACTTGGAGATAAACACAACTTGTTACGACCGACATCACTACCGACAGAACATTCCAGATGA

>Gm10ciRNA1458

CAAGCATCTAAAGAAGACAAAAAAAAAGAAGAAGAAATAACAAACAAGATGTAGTCGTACTACAACTAAACTAACACAAAAGATAGCAAAGTAGCTAATATTTAAAAGTATTAGTCTTCTAAGTCGAAAATAATTACGTTCTTGCAGGAATTAACTACTAAAATATTGGCATTTAATCCAGATTAATCTCAAAAAAAGTATTTCTAAAAGTCTAGGCAAATGTTGTTCGGAATTAACAACTAAGACATCAGCATCTAATTCCAAAAAAAGTACTTGATGAAGTCTAGGCAATTTGTTGTCGGAATAAACAACTATGAAGTCAGCAAAAAGTTCTTTAACAAGTCTAGGCAACTATTTTCGGAATAAGCAACTAAGACATACCATAAAGTTCTCTATAACGAGTCCAGGAAATCGTTGATGGAATAAACAACTAAGACACCGGTATCTAATCCTAAAAAAAAGTGCTTTAACGAAGAACTTTAATGCACTACCTAAAACTAAGA

>Gm10ciRNA1459

CATACAGTGAGAAAGTGAAGGTAGCAATTCAGAAAACGAAAAATAATAATTACAAAAGAAAACTAGGGTTTACATTAAGGGAACTCAGCAACGAGAATTACTAAAGGGTACAAAAAATAACACATTAGTACAACCAATCTAAACTCCTAATCGTAGTATCGTTCACAAAAAAATCCAAAAAAAACAACGTAAACAAAAATTAAATTTAATCTAATGCATAAACGACGGAAATAAAACTGTATTCATACAAACCAACGTGGTATATTGAATTCACTATCAAAATATCGAATACCCATCCACCTAAAAACGACTGGTATAGTTTACAATGGGAAAACTAAAATGACCCAGATATGTAAAACCGCATCAAAATCAAGCATAACCATACGACTAGGTTGGGTAACCCTTACACAAAACTACAACAACAACGCAACGATGACTTATTTGTATTCTCAAAACCTAATAATCTAAAATCCAACCAAATCTCCACAACCCACTTAAAAAGATATCAAACAATGTAAATGAAAACACATCAAGTTAAAAAAAATACTCGAAACGACTAACAC

>Gm17ciRNA146

GTATGCATGCCCATTTTCCCATTCATGCATGTAGCTTTATTTTCCTTAGCCAAAATATGTTTTGGCCACATTACCATAGTGTGGATAAATTTTGTATGGTTCGGGATCATCATGTTATCTGTCTATAATTTTGGTTTTAAGATTTTGAACGTCAAACATGATCAATTTTTTTAATTTATGAGTTAACTGTGTGTCACATAAAAATCAATCAAGACCATAAATATGCAATAGTTCCTTAATCATATAATAATTTCTCCGGTGACACCAAAGTAAAAAGGGAAACAAATCTCTTGATGCTCCTCGTAAAGATCAACTTGTGTCTTTCAAAATAAAATCCATCAAATAAAAACAATCAAAATTTATTCCATTTTGGTCGGCTCGGCTACTTGAATCAATAAACATCATGTATATTATCACCGCACCACCACTAGGCCGACCGTAGTGGGTCAAACATCATTTGAATTCTATCGTTACTTCATAAAGTAATATTAAAATTTAAAGCTTGGAAGAGATATTGTTGACA

>Gm10ciRNA1460

CATACAGTGAGAAAGTGAAGGTAGCAATTCAGAAAACGAAAAATAATAATTACAAAAGAAAACTAGGGTTTACATTAAGGGAACTCAGCAACGAGAATTACTAAAGGGTACAAAAAATAACACATTAGTACAACCAATCTAAACTCCTAATCGTAGTATCGTTCACAAAAAAATCCAAAAAAAACAACGTAAACAAAAATTAAATTTAATCTAATGCATAAACGACGGAAATAAAACTGTATTCATACAAACCAACGTGGTATATTGAATTCACTATCAAAATATCGAATACCCATCCACCTAAAAACGACTGGTATAGTTTACAATGGGAAAACTAAAATGACCCAGATATGTAAAACCGCATCAAAATCAAGCATAACCATACGACTAGGTTGGGTAACCCTTACACAAAACTACAACAACAACGCAACGATGACTTATTTGTATTCTCAAAACCTAATAATCTAAAATCCAACCAAATCTCCACAACCCACTTAAAAAGATATCAAACAATGTAAATGAAAACACATCAAGTTAAAAAAAATACTCGAAACGA

>Gm10ciRNA1461

CATACTCAAAAAAGGGAACGGACAATGTCGACTCTTCAAGTATATACTCGAAGACACAAATTAGACAAGAAATTTGTACACGGAAGACTGTAATAAGTTTGTCACTTTAACTTGTACTATTAAAAACCTAAAATAAAACAAACATGAACATGTGTAATAAAAACGGGGTCCGATCTCAATCCTCTGATGGTTTAATACTTGATAAGGGTGTTAAATTAGGATAGAGTGTGTACGAACCTCTAATAAATACTCCTTTATTAGTGAATAAAAAGACTTCAAGAGAGATCATAGGCTATTTTTACTTCGGTAATACAGAAATTTAATTCGACTTGGTTTACGCGTGTGTCCTCGTTTATCTACAATATAAACACGGATTAGTTTATACATAAATTTTAAAATAAAAACCCCACCCTCAATCAAAACGTTTTTACCTAATACCTATTCTCT

>Gm10ciRNA1462

GTATAATGAACTGATATGTTGTCCCTTTTGGTATCTTCTTGACTTATCGTTTATCATTTTCTTCTTAACAAATTTGCGCTTAATTTTAAACTTCATGTTGGAAATAGATAAATGCATTGTTAATATATCCATGACGCAGTCTGTGATCCCTGTTGCTAAATGTGTCTGGTTGTAGCATAATCTCTTCAGGTGTTAGTGCTGTTATTTACAATTTCAATATCTGAGTAAAAATGTAAAATCAGTTAACCTAATTTTTGTGTTTCTTCTTCCAACCTTTCTTTACCTAATTTATGGTTATTTTGAACTTGATGTATTTTTCCTACACAAAGATGACATAGAATTTGAGGTTTACTGTAATTTGTTAGAATTAAGGACTTGGATGCTCTAAGCTGTGATCTCCATGCTTTGCCTTTTCATGTGTGTGCTTCCCTTGTATAAGAGAAAGGTGCAGTGAGGTTACACCTAGTTTATCTCACTTCTGAAAAATGGGGTCAAGATAGCT

>Gm10ciRNA1463

GTATGCGTTCCTTCTTTGTTTCTCCACAGACTAATCTGGTATCCTAAATGCCATTTTTCATATATTTGAATCACGTTTACAATTTTGCAGTATGAGTTTTCTGATGCCCGCAGCTACATATTGGGCCTTTTGTAATAAGAAAATAAGGAAAATTGCATTAATATGTTTATTTATGCTTTATTCA

>Gm10ciRNA1464

GTTCGTGTCTCTCATTCGTTGTTATTTCTGAATATGCTGATGGGTTATGTGTGGATCTTGGGAAAGGTTAGGACTTGGAATGAAAGATCAAAACTTGGTGTCACGGCTTCTGCTTCATTCCATCTTGTTGGATTTCCCAATTTTTGGTATTGAGGATTGGAGAAATGGAAATTTGGGATGCCTTGTGTTTGCATATTGGCTTGTGGATGGGTTTGGTTTCATTTTGTTTTGCTTTGCTTACAAGTGTGGTTATTTAGTACTTTTTCTT

>Gm10ciRNA1465

GTAATGAAATGAAAAATTACCTTTGGCTATGTAGTGGTAATTTAAGCTAGTAATTTGTTGAAGCATTATTAATAGGTTTTTTCTATGGCTTGATATTCTTGCACGGAGGATTCTTTTCATGATAGAAGCTGGTAACTTCAAGAAACATTACAATCTATTCTACCCTGTCCCCAAGTACCAGGAATTTTTTAAATGGGTTTTAGTCAACATCTTAGAGGCTGCTGGTTTA

>Gm10ciRNA1466

CATTTAGTGTAAACCAACAAAAGATCTAACTTAGACCAAAAATATTGAACGGAAGTACAACTGCTCCAATTCAGACAAAAATCTAATACAAATTATGACGTGTATTTTAAAATACTGATTGGTCATTTTCTTTTAAAAGAGTTAACAACGTAATTACATGTACTTTAAAGTCAAAAACTAAAAAGGTAAGTAAAACACGACTTACGAGGTTAAACTCACGTGGGACATAGTATCAACTGGAC

>Gm10ciRNA1467

CATTTAACCTCCTAGTAGAAAAGACCTTAAAATCATGAGATGGTATTAGATTACAGAAGTTGTGAACAAAACTAGATTTACTTAGGGTAAGAACAAAATATTTTTTGATCGTATAACGTAAACATCAAGTTTGAAATTTGGTGGTTTAGTGGCGACGTAAACCTACCTAATACAAGATCATAGTTAAAACAGGGAAAATAACGGACATGAAAAACTTGTCTATAAGGAAAGATGTAATCTCGTTGAGTAAAACTTAGTCTTGTTACAACTGTAACACAATAATCACTAACTGAATGAGATGGACGTAGGTACAAC

>Gm10ciRNA1468

CAACACGTGTAAAGTGAGAAATATCCTTAAAAGTTTACAGTTTAAGCTTAGGATCGACGAACGAAAAAAAGTGGAGGAAACTTAGGAGTTGACCTTTGGTGACGAGGGTGTCACTATAACGTATGCAGCTATATGAAGAATTTTCACAATCGGAATAAAAACAAAAACTTTTTTATTAAATCGGAATTGTTGTTTTACGTTACCTTTTTACCTTAACGAGTGTGATAAAAAAAGGAAAGAGAAATGGAGAGGAAAAACACACAATTGCACCTCTCCCGGTTCTTGCTTTCTTTGTTTTCTTTTGTAAAATCTCTAGTACTTTAATGTTTTCCATTAGTAGAGACCAAAGTAGACCTCGATCTGAAGAAGAAAACGTAACGAAGTATAAAGTTGTAACGTATCCTACAATCACATAAAGATATGCACAAGTCACGAGGCTATCTTTCTAATACGTAACCCACTTCTTTTACTAAAATCCAATGGTATATTAATGGTGGGGCGGGAATGGAGGTCAAAAAATGTTAAAGAT

>Gm10ciRNA1469

CATATAGGAGAAAGGAACACACACAATCCTCGTCCTTCTCAAACACCAATGACGTTTAGTATAATTACCAAGAAAAATGAACAAATAAAACAATTAAAATTCTTACTACGTTAAACTAAGAAAAACAGGTTTTAAACTAACCTAACTCCCTAGTATAACCTAAAAAAAGTAGTAATTGTCGTTTTATTTGAGTGACAAAGTTAAATACATACTCTCTTACAACTCCTTTTCATGAAAAGGAACCGTCTCGATCGAATACATGAATTTGA

>Gm17ciRNA147

GTAACGGAAAATGGTTTGGGATTTAGGGTTTCAATTTGTTCAATTTGAGTCTGGTAGTTTTGCTAATGAAAAAACAAAGGACTTAAATACATTTGATCCATTTAATTCAGAGTCTTTTTTTCGTCCCTGTAATTTTTTTTATTAGTTCTCGTAAAATGTATTTGTATTGTTATTTTTGTGTTTAAATTGCTTTAGGTAATAAAAAAAATAACTGTTTTAAACAGTGAAAAAAATACTATTTAAAATACTTTAAGAACTAAAAACAAAATATGTTTATTTTGTAACGAAACAAAAATTTGCAGGATGAAAATGAAAAAAAAAAAAACTCTACCCGAAGTTTCATGCACAAAAAATGTATTTAAACCTAAAAAAAAGAGCTTCTTTTATAAGTTTTTTACGCAGCTAATATAAAGTGTGTTGTGTATATTTTGGGTTTGATTTTGCAAACTGAAATTTACTTGGAGTTGGTTTTCTTAACATGATTTCACTCATAGTAATAAGTGAGTTTTTTTTTTTTTTTTGTGAAATAGTGATAAGTGAGTAACTTTGATTTATTTTTTCAATTTTTTTATTTGGAAAGTAAATTTGGTTAGTGTAATTTTCATACACTATTGTCAACCAAATTGACTTTTAAAATAATTTTTATTGAATTTACCACACATGCCTATTTTGCGTTTGGTTTTAGGATGATTTTTGTTAATGCACCTCAGAAGCAACAAAATATTGGTTTTGCGGTTGGGTAACAAAAGCATGCAATTTAGGTTTTCAAATTGTTGAATTTAAATTTGGTATGTTGCAATACAAGTAATGATGAAGTTCTTGCCTATATGCTTATGAATTTTGATAACTCAAATATGTAGTTTTTGTCCAAAATTATTCCGTTTCTTTTTTAA

>Gm10ciRNA1470

CATATATCGAAAGAAACCTGGGCGTGTCTACTAAGTAAAATCACTAACAAACTTTTCCACGTTTTAGGTTTATGTTCGGTTTGTTAGCCCAAACTCACCTATGAAACGTATAAAAATTAGCCCACAAATACCTAAAATTCATACAACGTCCTAACTTTAATAACAAGTACAAACCAAAAGACGGTAAAACTAAAGGAGTTATGTTACTACTGGAAATGACGAAGATACCAACTTACGGAATTCGATTCTCAATCGATGAACATACTG

>Gm10ciRNA1471

GTAGGATACTTAATCAAACCCTACTTGACACACAACATTACTCTCCTTGTATGGTTTGGAACTACTACATGCTCATTGGTCCTAGTCAATCTCCAAGTATCCAGGGTCCGGAACTACTATGTGTCTGTGGTCCTGGTCAATCTCTATAACACGCATAGAAGAGTCTTATCACTTCTTTCTCAAAATTGAAAAAACTCAGTTAAATATCATAGAGATTGGACAGAACAATGAACATGTAGAGATTAATTGGAATGATGAATGTGATAGTCCTGAACTATATAATAATTTCTTGTCTTATTTTTGCTGTATAACT

>Gm10ciRNA1472

GTAACAATTTCTACTCCTTTTTATTATCAGTGCTTGTTGTCCATCTGTTTTTTCCCCCCTTGTCTTTTAAATTTTTAATTCAATATTCTGTATCTGAGAAAGTCCTCATGCATCGAGTTTAAATAATTCAAAATTAGTTTCAGACTACAAAAAAAAAATGGGAGTTATGAAAACAGAATATATAAGAATTAATGGAACAGGTGATGGAATAATGAAGAGGATGCAATATGTGAATAAAAATACAACATTATAGCCATGAGTCCTTAGAACACTTTTTGATGTAGTGTGCAATTCTGATTTACTACCATATTACTGTAATATAGCATTAAGCTGAGCCTGTATGTTATCTAA

>Gm10ciRNA1473

CATGTCCTGTGCGAAGAGAAGAAGGAAAGCGAAGATGCTTATGCTTAGGAAAACAAGCACCCCTATTTAATCTAAACACCCGGTAACGACACAGACAATGGAAAAGACGGTGGTAGGTGACAAAAATAAAACTCTTATAATACAACGAAAGACGAATAAAGAAAATTAAAACTTATAGGTAAAAGGTAGAAAATGTTTTTGTGAGTTGAATGAATGGCAAGTACACTTAGTACTGTACTGAACTTCGAACAAAGAAGGTAAAGACAAAACACCCCTCCTCCCTGATGGGTCTCACATATGGAAACAATGCGTTAATACGAGTAAAAGGTATATTACTTGTTAACTAAACTCTAGTAAACGTTTTAGGGAAACTATGAGACGATCCGAGTAGTAATATAGTATGTACCTTACTTAAGTTCGGAATCGGAATGTGAAAAGAGAGAAATAAAAACGATAGTTAAGCTACGGTACAAATTCCACGCACTTTACCATATGGGAACTTCCCTGACGTAATTTTAAGAAATGAAGACTTAGACTTTACGACTAACGACTATGTCAAGGCAAGAAATTATAAAACAAAAACGTAAAAAACCAATAGTTTGAGTTAAACGACACTTGGTTTCCCCCTAGAAAAGGGTAACGCCCGATGGTATACGGTGTGTACACGTATCGTCACAACCACCCCACCAATGGATCTAAATGAATAACGTAGACAAAAGATCGTTGAACCACCGTAACTTTAAAATAACGCCTAGAATACAATAAGAGAAAAAGAAAGTGAGGTAGTTAGA

>Gm10ciRNA1474

GTTGATACTTTATCCTGACTTCTGATTATGTTTACTTATATGGGATTGAAGGAGAATATCAATTATTCCAAACAAAAATACTTTCACTTTCAGGAAGAAGCCTGCAAAATTAAATAACTAGAACTATTTGACTTCATTCCTTAAGATGATAAGAAAATGAATAAAAGTTTTAGTTAAGTCTTTCTTGCCAACTACTGGTCATATGTTTAAATCTTTCTTTATTGAAGGTGATTGGTTTCAAAATAATTATGATGCTAGCTGAACTGTGACATT

>Gm10ciRNA1475

GTATTTGTCTCAATTTGGTTGTTGAATTGTCGATTCTTTTCTTTTTAATAGAAAATATATCAATTGTTATTTGTCTCAACCTTGAATCCTTGATAGATGGTGACATGGGATGAGATTAATTCCAAATATTTGCAAGTCCCATTGTATCTAGTGATGAAAATGGAAAGGATACCAACTTATATAGATTTAATATTTGACATTTGATTTATATTAGCAAATATATAAACTATTCGTCTGCATAAATCATAATATGTGATTGATGATTCATTTGAAATTGTTTTATTTTTTCTACTTGGTGCTG

>Gm10ciRNA1476

GTAAGCAGCTTGCATGCAAGTAAACTTAACTGCTATGATTTTAACCAGTTCTATTTGATGTTACTCTCCGTGGTGTTAATGTTTCATATGTTATAAAGGCTATTTTAACTTGTATGATTATGATATGATACTTTTATAATGAATTGCTCTTTTGTCTATGAATTGTTTGTTTTACATGCTTCCACCTTTATTTATAATTGACTATCACCTGGGGCTCCCTTCGGATCTGACATTTGAAAAGGTTTCTTCAAGTTACAAGCCCCACTAGGAAACAAGGCCTAGCTATTTAATTGGCAATACTTTGTATAATCACAGTATAAAACTGGCCATGCTCTGACATACACTTGATTTATTATGATTTATCACTATCATTGAATCATTGTTAATAATACTCTCTTTGACACACACACACACACACACACTATTATTCATGTGGGTCTTTATTAAATAGAGAGCCCAAAATTTTAGTGCATTCACATAAAATTTATATAACGATAGAGATTATCTTAAAAAGAGTGTGTTACTTAGATTTCTTTCTCTATGTAATTGTTTAGCTAAATTGAGCCCCCCACAGGGGCTGCTCCTTTCCACCATGTCTTAATTGGAACTTGTCTTTTATGCATTGTAAGACATTTATTTTAACTCAATGGAGCTGTGAATGTCAGTTTTCT

>Gm10ciRNA1477

GTCCAATACAATACTTTTAGTTGCAAATCAACCATGCTTTTTATAAGCTAATATTGTTTGCTATTTTCATCACCTGAGATGCATGACCCCCTCCCCTCATCATTCTCATTGCTAAAGAAAAAAGAATCTGGCTTTTAATTAAAATTTCAATATTAATAAATCTGATCTTAGAATGGTGGCCAAATATTTATCTGCCTGTAGCTTCTTCACATCCTTACTGGTTTCAACATGTGTCGATGGCAATTGAAGTCAGAGCTGAGATTTTGTTTTCATTGTCAACCAGGACTTATGCATTTTTGGAACTTCCTCAACTTTGTAATTGAAAACTACATGTTTGATACTAAATCATACATAAATAATATCAAAAGTGGCTGGATTTACAAATGATTAAATATGACACTCTGATTTGTAAATAAATTGATTTCTTAAACAAATATAGTTCCAAATAAGTTTCAGACTAGAATTTCTTTATGAATTAGATGTTTACATTAGAGATTTTCAGAGTTAAATTATATTTTGGTAAATGTCACATGTTTAAATTGATGCTTGTCAGTTGGCAATCTTA

>Gm10ciRNA1478

CAAGCAAGCATATAGAGAAGAGCAAGCAAGTTACAAAATGACCATCACGATCGATCGCCTTGTAGTTTGTCGTTGTGTTAGGAGTTGACATTATAAACAAGAAAGTTAAGGTTAAAGACATACGCGATGAACGAAAGTTAAAAGCTTTATCTCAAGCTAACTAAT

>Gm10ciRNA1479

CATATAACTATTAGTAAGCTATAGTTAAAAAACCCTACTTATGTATTACTCATTGACATACTCTTAAAAGAAAGGTTTAAACTACAGTAAATTGTGAAATAAAACTTTTTGTACAAACTTCCTAACTGTAAGTATATTTGGTACAATATCGTTGACATAAACTCAAGTGTTCGTGGTCGAATAGTTGGTGTTACTGTTTTTGACCAAGAAAGTATAAAATCTTCCGAAATAAAACATGAAGAGATAAGTGTTTTTTTTTTACATGAAGAGAGTAAAAGACCTTATAAAAAACGGTAGTGAGGATAAGAGAAATAATTATAAATTATAATAATTTCCAAGTCGAGTAGGTCGCGACGAA

>Gm16ciRNA148

CATACGAAAGGAGGGAGGCTTTGGGATTAAAGGATTAAGGAGAAAAAACCAAATCCGGTTCGTCTAGAACCAAGAACGTCCTTAATATAGAGATGAACACAACACGGTAAAAGATACGGTAGGGTTTACTTAGAATCAGATTAAACGACAGTTGAGACGACGGTAACCTAAGATTAACGAATAATAAAACCCCCTCTAAAATGACACAACTGGATGAAAGTGAATTAAGACTAAAACCTAGCACCCCAGCCAATTTCAGAAATAGAAACTTGAAAAATAACTAAAACTTTAAATCCTTCGAATCCTAAACGAGTCCTTTTACTTTCCCTATTTCTAAAGCTATCCAAAAAAAAAAACCTACTGTTCTCAAACTCCAACGTTCAAAACCCAAAACCCCAAACACAATCGATGCACCATTAACAAGTGTTTTAGTACTACATCTACGTGTTTCAAAAAAGATCACGGTTGTAATAACACCTCCATGTCGAGCGCACCGATACATCTAATTAATCCAAACCAATCCTCAACGACCCAACGATGACTACACAGACCGATTCATACTTCATGACTACTACACTTCAGAGAAATGAGACATTATATGTTTAGACTCATTACTAATACCAAGTGTTCTACAAAACGAGTAAATAATTAGTAACCAAAGGGGGACAGTCGAACACAGGAATATCAAATACCCGTCTTTTACAAACAGACATTAGTCACCAAAATATTGGTAATCATATAAATATTAATATTTATTAATATTTGTCAGAAAAT

>Gm10ciRNA1480

CATTACAATGGGAAAGGAGGAAGGAATAGGGAAAATTCTAAGCATTAGAACAAAACAGATTTAAAATTCCCCCGTATTAACGTTGAAGTTTGAATGGACCTTTTGGATCCATAGTAACCAGTAGCACTAATATTAACAACAAATGTGAGTCACAATAGTTACGGCCTTCCGGTTTTAAGGCGCGGTATGTACATATTTCACATTATCATTGGTACTAATTTAGTTTATGTACATCTTTTTTGAGAGTTCAATCAACGTTAAACTAAAAGGATTCTACTCGAACGGTCAATCACTTCAGGAAACATCCACTAATGTCAAAAGACGTAACTAAATTTTAATTTAAGCTTACTATGTGTGTTCATT

>Gm10ciRNA1481

CATTAGGTACTTCGTACTTACTAAGACACAGAATTAATTCATCATATTTACGAATTTAAGTACCACTTCAAACACTTAGATCGTCACGATAAATAACATTATACTCGAAGTTTTACACAACATGACTAATAATAACTAGTAAGTTAGAAGTAGAAAATTTTCACACTAGGTAAACAATGACTCTCGGACGATGTTAGTAACAAAACTATAAAACTTACCAAGTATTGAGATTCGTAAAAGAACTCTCAAAAAACTCGGACAAGATACTTTGTGAATAGAAAATCCCCCTTTTTTTCATATGATTTACAGCTTAAGAGAAGGCTATCTGACATCCACATTCACCTATGGATAAATTAAAGATAACAAAGATGTGGACTTAAAAAGACAAAACCTATAAAAAAGGACTTACGAAAGTCGAAAGAGTAGGGACAGTGAGTTACGTGAGTACCAAGTTAACACATATGAAACTAGTACATTTCG

>Gm10ciRNA1482

CATAGGAAAAAAATTTTACGAACGTATGTATATTAACTTTGTGGGGGAGGTGGGAAAGGTTATTTAGAATTTTGGTGAACCTGAAAGGAATAGGAAAACTCCCTGAATTACCTTATGTTGGCAAACAGATAATTAAACTTAAGGAAACTAACCTCACCAAGTGAAAAATACGTCCATAAAATTTTGGGAGATCTAAATTTTCAAGTTTCGGTAAGTAACATACTTTAAAGTATAAACAGTCACTATTAGAGGACGGAACTCGGAAATACATCGTTAAGAAGTATTAGTATTTAATAAAACTGCATGTAATATCGATAGTAAAAAGAGACCGTTGATCCATGTGAACTTAACTGAAAATATACCATTAAGAGATCTAGACAATAATGGTAAGGGTATTAAGTAAGTTTCATACCAAGGACACCGAACACGAAGAGTAAAAATTGAGAGATTAAAAGACACCTACTAAAATTTCGAGTATGAATATTATTATTTTGGAGAAATGAAAACGGTGTCTATTGAGATAACTCTATATAAGGAA

>Gm10ciRNA1483

CATTAACAAAAGAAACGTAAGAATCTTACGGGATAATAGTAATCTATAAAATACTTTTTCTCTTTAATCCGCTTTTTGAGTAGAGATTTACCGAACTATAAAATGATTCGAAAACGATTATAAACAGAGATTTGAAATCTATACATTCATCATTATGTATTAGCATACACAGACCTTTTCAAAGAAGCCCACGCTAAAACAATCTTCAACATACAGTAAAAGTCAAATACATTATTCGGAAGTAAAAACAGTGGAGTTTTAAAGCTTAGGCTCGACAGTAAAAGACT

>Gm10ciRNA1484

GTCGATTTTGTCTTTCCTTTTGACCCATCTCACTTTTTATCTGATTGTTATATAGTTGATGGTGCATTTTTTGCTATCTTTTCACATATATAACATTCTTCAAATTACTCAGACTGAAGTAAACAATATCCATGTTTTATAAAGTGAGTAATTAGTTTTGGCTACTCACTAATGATTGTGTTAAAATAGGAGGAAACAAAGGGAAATTGAATGAAAACCTCGTGTTTGAGAACACCACGGTTGAGGAACACAGTTGAGCAACTAATTACAAAATGCCATTGTTCTTA

>Gm10ciRNA1485

GTATATTTAAGTTTTTGAAATATCCCTGTAGTCTTTACAGCATGTGTGCACCTACAAATTTTTTTCCGGTAGGGTTTTCGATTTAATATTTTTATTTTCCAAGTCTTGCAACGAACATCTGTTGTGATTTTGACATACATACCATCAACATTCAGTTTCTGGTGGTGATCCTTGAAGCACCACTGTAATAGTTGTGAACCTCCCCCAACGGCCCAAACTTTAGTGGAAAGGAAATGAAAATTAACTGCTTGACTT

>Gm10ciRNA1486

GTTTGTTTTTTCTTTTCTTTTCTATTCAATGAAACAAGGTTGTTTGTTTGTATACTTATATTGACACAAAATATAATTATGCTTGAGCTTCGTATTAGCAATGCGGGGGAAGGGGTTCATAACTATATTCTATTTCCTATTCATTAAAATGCATTGTTTAATGGTAATTTGTTAACAACAACCATGATATGATGTGTTTCATTAACTATCACGGTTGTTCCTAGTGGTTCCAGTCTCTGGATAAATAATGATTCTATTCATATGCTTTTTTGTAGGTATAGATACGTGTTTAATAATTTCTATAGTGTTTTGCCATCATTTTTTTCAAGTTGCAACTCA

>Gm10ciRNA1487

GTGATTATCATCTCTAAATTCAATATTAATGGAAATGTCAGTTAATAGCTATGAATCTCTGACCACTAGACATGATACAGACATCCTGCACATTTGTGATGTGTTTGAGGCTTCAGAGTCTGAGCTACTTTTTTTGTATATTAGTGTGTGTACTGCTGAGTGACAGACAGTACTTTGTTTCCCTCCATGTGGGAAACGAAATTTCTCTTGCATCCACTTGTTTATTGATTTGGAATTATCTTTTGTTTTCCTTGGTTTAATGATTTGGAAAACTAATTAATTGGGTTGGGCAATCTAGAAGTGTATATTTTTTTGTGCATTTGACAAGGATGGTTTTCCTTAGTCAAAGTATTATCTTGCATGTTAATCTATTGCCGTGTCCTTTCTTGCATTATGTCTAGTTGGCAAAACGAATTTGCAAATTTATTATAGGGTGCAAGACTCAAAAGCTTTAGGAGAGGATTGAGCTGAAAGAAATTGGCTCCTGCTTTGGCATTGTGAATAGTAACTTAGGGATGTAATGATGATTGTGATGTGAAGCATGTTATAGTCCTTAAAACAATTGCGTCATTTGCATATTTCATTTCTTTGACTTGCAGTTCTACTTTTCAAAACTAACAGATCTAATAGTCTGATATAAATTAGAGAGTTCACTGCTGTTAATTTGCTAACTCATGCAACCTCTATGCTTGTTGTTTAGTGACT

>Gm10ciRNA1488

CAAACAGAAATGAGAAGTGAGAAAAAGTCGATCGATAGAGAAATGATAAAAGAATGTACGATAGTCGATAATGACAAGGTTAGACGAAAATGAAAAGATACAATGAAAGGGGAGCAGTTGACAATGAAGATTCGAGGGTTAGTTATCTGGTTCTTTTTGCGATCCGGATATAAAGTACAAGAGAAGATGGTTAATCTGTGTGACAAAGTTATAAAGTGAAAATCTGTTATTATAGAATTTTAAATGCAAGACAGTAATAGAAAAAATAGAGCACGTTGAACGAGACTTTTTTACCCGAATTACAACCAGTTTATTATTGTTCTTTACCAACAACCTAGAATAGGTTCAACTTAGTATTTTTCTAGTTACTTTAAAATTGA

>Gm10ciRNA1489

CAAAGTAGAGAAAAGCGGATGGGTCTAAATCAAAACGAAGTTAAATAAGTGAAAAGCAAAGGACCTAGCTTACGTAGAGAAATTGAAGGACTAAGATAGAGTTAAGAATCATAACGTACAGTTCGAAGAAGACCGAAACTTAACACTGAGGAAGACTAGAAGCAAAACTACGAATCAAAGATCATATTAAACAAGGAACGAAGAAGAAAAGACAGTTATAAATATTTCTAAAATAGAAAATAGTAAAGATCCTTAATATATAATCGAACCCTAAATGCAAATCAATACCACAGTTATAAAAGAACATGTTAAAGCCTAACAGACTTGAAACTAACGATGCTCTTTGCTACCCCACTTTAAGTACTCTATGCACGGTGACGAATTGTGACCATAGACCGTCAAGTAAACGTACAAAATCTCAAACCTAACTAGAGTCTTTCAAGTACATTCTGAATAAAAGTCTAACACTACAATCAAGAAAGAGGCACTATCTTAAAGCGTAAGTTGAGATCAAGTCAAGACTACAACGAGGGTTCTTCATTTCTGTATTAAAAAATGTTATATGTGTAAATTACGAGTAACTACTTATTCAGTTCAGAAATTGGCTAACTAAACCATTTGAAATTTCAAATTTGACGACAGAATCATGTTCATTTCTTATAACGAACCTTTTTTAGATAAGTCTTAAGTTCATCGACAGTACAACGACTCTTTAGAAAAAGGAACTTGATCAGACACTGTTTTCTGA

>Gm16ciRNA149

CATACGACGACTAAATGACCAGAACTATTCAAGTTAAGTAGTCAAACTCAGAAGTTTGTCAATATTTTGTCACGGACTAATAACTAATGGTAACGGTTGTCAGCCAATATATACAGAAAACTATATCGACGTTCCCAAACTGATCCCGTAATAGTCCTAAACTAATACCTTGACAACCAACGGAAACAAATTGAACTTGGGGAGATTTCCCATAAAATAATACCGAACTGATAACTATTTATAAGAATTCGAAATTCTAGTTTACTTGGTCTCATAAACAGTAGACCCAGTATTCTAGAGAAAGAGACGAGATTCGTAACGAAAAATGAAACACTCTATGACTCTACGTCCGTGGTTATTAGGAATTAACATTTAGACCAGTCGATGGGTCAAAAACTTTTTATTATTACATGTCTTACTCAACTATTTCCGTTATCTCATCACTATTTCCCACTGTAACATTAAACATTCCAGGTTGATTTAGTAGAAGAGATAAAAGTATAAGAGAAACAAGAGAATATTTTACATTAAAAGTTATTTATGTCTTACTAGAAGTTCTAATTAGGTCAGACAATATTCTTCCTTTTCTTCATTAACCCTCTAGTATATGGATTACCTTGTTAATAAAGAACAGCGCCTTGACAATCAGAAATATAGTAACACTCTTGACCGATGAACACGACGGCGGATGTAAGTGTAGAGGACCCCACATTTATATGTATGTATCTGTATGTGTGTACGT

>Gm17ciRNA1490

GTATGAAACCTTGTAGTTACAGTGGATGTAAGGGTGTAAAAGTTATGTTTCAAAAAGAGTCAGTAATAAAGGCCAGAGGGGTTGGCGGAGGAGAATGGGTTAAGGCTTGTGAAAATGGTTCTTCAATTTGTAGTATTAATATAAATACTGAATGGAGGGCGGGCCCTGGCGCAGTGAAAAAGTTGTGCCTTGGTGACTGATCAGTCATGGATTCATCTAGAAATAGTCTCTTTGCATATGCAAGAATAAGGTTTCATATAATGATCCTCCCTGTACCTTTACATAGTGAGGAGGCTTTTGGCATTGGGGTATGTTAGTTACTGAATGGAAGAGAATAGTGTCTAGAATCCTCTGTAAAAAAATTCACAGGGTAGAATTTTGTTTTTGTTCCTCCAGTGGAGTAGTGGCAGTGTTATCAATGATGGATAGCGGAATATGCCGGAAGGCCAAAATTCTGCCATAGAAACATGGCATTGCAGCCTATGGCTTTGTCTAGTATCTGTGAAAGAAGTTTAGATTGCTTAATTTGAGTTAAAGCTTTTAAAAAACTTCCAAGGAAACTAATCTTAAGTATACAAGAGGTATGCCAATAGGGTCTCTCCGAAAATTGCTAAAATAAAAACAAAAAAGAGAAAAATTGAAGAAAAACTAAATATACAACTGAAATGTGCATTCCTTTTTATGATATTAGTCTGACTTCTGTCCTTTGAAGACACATCAATGGTAATGAGAATTTTTCCAAAGATGAAAGGCATGTGTTCAGGGCCTTGAGGATGGTCTAAAGTTTCCAGTACCATCTTATTAACCATTGGATAATAATGTTATCATATGTATGGTTTTCCT

>Gm17ciRNA1491

CACACGGGACTGACGTTATTAAACTCTTTATAAATAAAAAGACAGACTTTATACCAGACCAATACAGAAAATACAAAACAAAACAAAAAAGTGTCATACCAAATACTCAATCACGCACCTATTGACATTGAACTCTACACTATAATTACGTGACGATTATAGATTTGGTCATATTAAGTAATAGAAAACAATATATATATATATATATACATAAGAACTACCCGAAGGACGTTTTTTTTTTTTTTTGTTACTCTTTCTTTTATTTTGTAATAAAACGTCCTAGAACGTTCCTATTCCAGTACGTTAGTGTCCTTGGTGGTCGTAGGATTTGGGGATGGGAGTAGGGAGGTAATTTTGTTTTGAAGGTTAACTCTTTGTTTTTACTGATCTTTGGAAATTTCAAACTTATAAAACGAAAGATTTTATGTCGCGTACTGTAAAAAATGATAACTTAAATTAGTTAAACAGATACCACGTCGAATACAACCGCAATACTCTTGTACGATTCGATAGTGTTACTGTCTAGAACGA

>Gm17ciRNA1492

CATTTTAATGGTCATCGACTATCTATACAATAACCCCCATGAATAAACCGTTTAGTACCAATGTCTGACATACACGTAGTTAGAAACTTCTATACTCTTGATTTTGAAACTTGAACTACGAGTTAAGGACACTATATATAGTGAACAATTGAACGGTACTTAAACCCACTGGTAAAAGAGGTACTTATACAAACTAAAGTTAAGAAAAGACCGTGACAGTAATTAACGATGGGAACTAACATGACGGTTATCTGATACTGGAAATAAACAAAAATGAAAAAACCGATAGTTAGACTAATTAATAAGTAAGTGAGACGGACACCCATGATCGGTACTAGTTGTATCATTAGATCACGATTGGACGTAATGTATTACGTATAACAAAAATTGGATTAGAAGAAATTTGTGTTTGTCAAATTACCCTAAGTCTTAAACTACAACTATGCAGACAGATACATAGAAGAAAGGACTTCGGTAATACTTGTAAAGAATATTCACTTGACGTTTGTAAGTTATGGTATTAAGGTTCTATAAATGAAAATCCGCGGATACCATTCCTCGTTGTAGTGTTCAAAGTTGTAAATTTTCTATTAGAGTAACAATACTAAGACTCTTTGTAACAGAATAAAAAATAAGAGAGCATCTCTTTCTTTCTTCGACATCGAACATCAAACAAGAAATGA

>Gm17ciRNA1493

CACACATCAGTATACAGGTGATGCAAAACGTACGGGTGTTTGAGTCTCTACGAGGTTGGAAGTCAAAACTGTTAAGGTTGTGAATGTATTCTTAATTCTAGTACCAAAAAAGAGCACTTCAAATTTGTCAACAAGTTTGTTAATTGAACTTTAGTTTAGTACTAACTTTCGGTTTTTATTTAGTTTTATTTCTTTTCAGAAAAAGAAAACTTATTACCTATTTCTAATCCCTTATAAACAAGAAGAGTAAATCCTTCAAGAAAAGGACCGCAATAACCACAGGTATTAACAAA

>Gm17ciRNA1494

GTGAATGAAGCTATGCAGTCAGCCTTCTCTGTGCATTTATTATTATGTGATTAATAATGGGAATATCCTAGTATATAATCAGAGAGCTTTTGAGAGAATCAAGTGAATTGCATTTTCTCCAGTAATGCAGTCAACATATTTCTTATAATATTATTTCTTTAACCTTGGATTTTGTACATAATTTGTTTTTGTCGTATGTTCCAATTCCATTGATGCTGCTTTTCCTTTTTCTTGTATCATATGATGTGCCATCTTCTTGGTTTTTTAGTATAAATAGCCTTATGATATTTTGAAACCCCATTCCCATAATGCTCTAAATAATGATTTGAAATGCATATTCTTCAGCTTTCCCCTTTTGGATTATATGTTGCATGCCTGCATAGTCT

>Gm17ciRNA1495

GTACTATACACTCTCCCCTAACCCATCCATCATCCATGTACATACATTCGTGAAATGACATCCTCTCATACACCACAAATTATTATATGATATGATCAATCATCTCAGCTGATCTCCACATAACATATAATTAAGTTACAGCATACGTAATAATAATTTGTCTACATATATGTATTCATAAAGCATACATAAACTTTATGCATCACAATTCTTTTAATAAATATATGTGGGCCCCGCATGATTAATCAGAGAGATAAAGCCAGATGCAACTAGACAACATATATGAAAGATGCAACACCCTTATGTATGCATGGGTACATGACATGCTAAAAGAAAGTACACGTGGCATTGTTGCTTGTGATTTTATTTAAGTTTGAATAATGA

>Gm17ciRNA1496

CAAACAAAGGAATTATAAACAATGCACGAACGGTAAAGGTACGTTTATAGACGAACTATACGCCGAATTTCTATAAAAAAAAATAGACAAAAGGAAATCTACGGATGAAAGACAGACAACACAATTACTATGTAACTATGACCAACCTATATCAAGAAGTCAAGAGATCAATAGAAAACGCACGCATAGGGCCTTCAATAACAAATTAAATGTTAAGAAAAGTCAATGTGAGAACTTCTACATGTCAGCTTTGAGTAAAGAAGGACATTAAAGGAAATTTTTAAACCCGGTTCATTTACTATAATTTATTATTTTAGAA

>Gm17ciRNA1497

CACCCAATTGAAGATATAGTACACAAATAATATAATGTTAAGGGTTATCACGAATATCACTAAGTCTGGCCTAGTAAATTGAAAGGTTCTACCTTTATCATACTGTTCCAAATCTTATGTTCTCCCCCTTTACTTGGTATATTATACACGACGCCCAACATATCCCGTCTTATTCTAAAATGTTATTGTAGCAGAGTCAAAACAAAGAAAAGAGGATTTAGTAACAA

>Gm17ciRNA1498

GTACGTCTGCGCGCATATGAATAAATACGCTAGATAGCTATCTGGTCCGTGAAATTAAAAAAAAAATCCTTGTCTATCAGTGTGCTTAATTTACATGAACATACTAGATATGCTTGTCCCCTGCCTTAATGTATGTGCTAATTGTTTGTCCAGGAAATTAAACTCTAGCATTAATTTTCTCCATTTCATAAACATGGCATTGCTTGCAGATTAATCTCTCAATAGTGTTTTGTTTTTTGTTTGGCATTTGATCCATTCCCCCGGTCGCTAGATTCTTTCCA

>Gm17ciRNA1499

GTACTTGTAACTCCCAGTACAAAGGTACTGAGAGAGATTTTCAAAGTTGTATCTTTTCATGAAAACATGTGTCTATTTATGAAAAAATATAATCCACCATAAAATTTGATTATGGGTTTGCAAATTATATCAATGGACTTGATTATATCTTGAAGGAGATTCGTCACTACATCCTGTTGCATTCAATTTGTATAGAGATTATCTAAAACTCACGTACATACGCCTTGAAGACTTGCGCGTTTTTTTTCTTACTGTTTCTTCTTTGTTACAAGACTATTGCCATTAATATTTTTGTCTTTGATATTTC

>Gm06ciRNA15

GTTAGTACTTGGACTATCTTGAAAGTTTTGACCTCCCTTTCCCCCCTCCTCATGCGAGTCTGTGACTAATTACTTTTGACTTGAATGGGGCATATCAAGTGAGAACTTCTCTTATTGTGAGAAATGATAACTAAAAAGTTGAGCCATATATGATACATGGATGATCAAGATTATAAGTTAGTCGGTGGTCACAATGTCACATGATTACTCAAAAAGTGATGTGTTTTTCTTTTAATCTTGACCATTCAAATATGATTTAACAGCCTATATTTATAGTTCTCATTTCTCACAATAATAAGAGTTTTCATTTGATATGCTCCATATATATATATATATATATTAATTATGGTCTAACTTGCATTTACTGGAATGTTTTAAGTTATATAGATTTCTTTTATGGCTTGCATATTAAACTTTCCT

>Gm16ciRNA150

CATTGCCTTAAGACATAGATAATCACAAAGAATAGCAAAAATGTCTTCGAGAGAGCAAGCGGAATAGGCTTTTCGACTTCACGTAACTAAAATCAAAATATTTTCTTTGAGGTAAGTAAAATGGAAGAGAACCTTTTTTTAATAGGGCATTTAACTAAAATTGAATAGCCTATTTGAATTAAGTAAAATATAGAAGAACAAAAAAAAACAGAAAGATATTCTCCAATATCTTTCAAATAACAACATATTCTTATCCCATCATTTTAAGATATAGACTTACTAAGAAGATATTAAAAAAAATTATCTTTGAGAGAACAAGTTGAATAGGTTTTTGAGTTCACATATTTAACAAAAATCAAATATTCTATTTAAGTAAAAAAAAGAATAGGAGACTCACAGATAACCTCTTCAAATAGGACATTCAACTAAAATTGAATACCCTATTTGAATTAAGTAAAATAGAAGAATTAAAAAAAGGAAGATGTTCACGGATACCTTTGAAATAGGTTTGTATAAGTATATATTTGTGAAAGAATCTTGAACAATTTTATCCTGTCCACCAAATTAATTGAA

>Gm17ciRNA1500

GTACTTTTAACTTGTATGGTATCTACCTCTACAACATTGCATTTCTGTTGATTCTTTGTGCACTATGTATATTGCATTTTCTTAATTCTTATAAGTTTTGTTTGATTTATTTGTTGTTCTTGTAAAATTATTTTTTTGTTTTTAGTCCTTACAAATTACGTTTTTTTTTTCAGTCCCTATAACGCTTTAGATAACATTTTTTTAAGGACTAAAAATAATAATAAAAAATTTACAAGGATGATTTTCTTTTTTTTAAATTACAAGGACTAAAAAGATATTTAAACCTTTATTTTATTCCTGCAATAAGTTTTAGTTCTATTTGGCAGTATAGACTTACAGGCAATGTAGCAACTTCAAATAATTGCAAGCTCATTTCCTTATCGTAAGGACTATATACATACACTTCAGAAAACTGCTGGCCAAAGACTCTGAAATATCGTTAAATACAACACTTTTATCCTTTGAATATGTAGTCTGCATCTTATTATTTGGATGTCTGCACTGGCACATATTGTGTTTCCAGTGGTTTAAACTCTTAATAATGGCAATGGTTATCGATTAACTGTTAAGATTTGGATCCTCTCCTTTTCCTTGGTGACCATGATAGTGCCATGTTTTGAGGGAAAACAACACAGTTTTTCCTGTACAACATTTCCTATGCAGGTGGTCACAAATCTTGCATAGGAGTTGTTTGCATCTTTCCCTATTTGGACAACACATTATTGTCACGCTGCTAAAGAAATTGAGAGGATCAATAAAGAGTGAAAATGGTTTATTGTGTTGTTAGTCTGGTTCTGTCATTAATGTTGTCTGACAATCCAATGCATAAGCTGTGTAAGTAAACCATTTGATGATTTTAATCAATCTGACCTCAATAAATTGTTTAAATTTGATGTACAGAACACGAACGGAAACAATTCAGGTTCTATTTGGAAGAGGGGGGAGAGAGTGATAGGGTCATAGGGAGATGAATGGAAGGAAACCTTTCTATTTTTAGTACGGACTGTAGCTTTCACCTTTCATAACCCTCCCCCTCCTGGTCTTGCACATAGACCCTCACAGTTGAGGTCCTTCAGCAGCACTTTTCTCATTTCCACCTTTGA

>Gm17ciRNA1501

CATGAAGGAATGGAAGATGTGTTACTCTAATCTCCAGCATACATTAGTTTTATTTCTGTCTAATGGTAAAATAAGGTTACGTAGGTAATTATGGAGGTAGGTAACCAGGATCATTCCTTAAACTACTGTCTTTAAGGTTACGTAGGTAATTATGGAGGTAGGTAACCAGGATCATTCCTTAAACCTCTTAAAAGTTGGTATTCAACAACCTTTTCGGCGGAATTAACGTCAGAGATACCCCAACGGACTTCACGTTGGAACTCCCACCACCAAATCACCAAATCAAGGTGTAGTCGATCACTATACTGGTTTTATCACATTTATTCACTCCCCGTTGGGAGTGGAATGTTCGGCCAAAACATCTCAACTCAACCCGAGTTTAGGTGTAAGATTCTACCATAGTATTGGATAGAATCTAGGTAACAACCCGGTGGAAGTAAACTGTGTGCAGTCCAACCTTCAGAACCCACACTCCCTCACAACCTTTTCGACGGAATTAACACCTGTGGGGAATCGTGCGGAATCAACACTGGTGAAACCACGAACAGACTTCATGTTGGAACTCCCACTACCAAATCACCAAATCAGGGTGTAGCTCATCACTATACTGTTTTTATCACATATATTTACCCCCTGTTGGGAGTGGATGTTTAGCCAAAACATCCCAACTCAATCTGGGTTTGGGCGTAAAATTCTATTCAGTACTATACTGAACTAGGTTGATACGAGGTACCAAACACTTGACGTTTGATAAACCTAATTAAAATGATTTTACGGTTACGGATTATAAACTCACAACTTCAAACTGTTGAGGACCCAAACTCTTGCCGTTTGTTAAACTTTATAAATATGATTTTACGATAACTGATTATAAACTGGCCACTTCAAACTGTTTTCTGAAAGATCTTCTCGAATACACATTTATATTAGTAACCTTACCTTTTTAAATAAACACATCTCCAAAAGAAACTGTAATCAAAAATATAGTCAGTGTCGACAATGATTTAATAAAAACTCATATGAAACGACAACCTGTGAAGTGTTACTAATCGAGTAATAACTCTCTTGCTGACTCCTTTATAGTTAAAATGAATCTTAATCTACATCAGTTTCGTTTTTGTTGATATTTAAACTTAAAGAACAACTGTCAATAACT

>Gm17ciRNA1502

CATCCGTAAAAGTGGTTCCGCCGGTGATAACATCGAATAAATAACAAAGAAACCGTAAAGGGGGCGGGATATTTTACTAACATACGTATAATTACCGTCTGAGTTTCAAAAATAAAACTCTTCATGTATTCAACTACTGTACTATGATCTGTCTAGATGGAGTAAAACCATATCCGATTTCAGCTGTTGAAACCGATCAGTTATAGTTCCTTGTGTAAAATTTAGGTAGGGGACTATATATGATAGTAAATTTAAAAAATCTAAAAAGTCAAAATTAACGAGACATTTTAATTTAAAGTAAAACCAAGTCACTAACACTTCGTGTACAAGAATGACGAGTAGGCGAGTATACTCTAATCTCAAACTTGTCTCATGGGAGAGATTCTTATCAAACAGGACGTAAGAAGATACCACCTAATAAGCTCTGCATACTTAGATCTCCGACTAGACTATTGTACAGTGTAATCAATGACTTACCTAAAGAAAAAAATAAAGAAAACCACTGTTGATTGTTTACTTTATTTAGCAAGTTATGAAAAAAACACGTTCCTAAAAAT

>Gm17ciRNA1503

GTAAGAATCCATGAATCACCTTTCCTTTGTTTAAAGTCAATTTTATTTTCTGAGATTGTTTCATTTTGTTAGGGTCCTGTTGGTTCTGGTAAATTGCACTCTGATCAAACAAGCTGATTTATTTTGATTGCATCCAATTTTCCATTTAATCAATTTCTTTTAATGCGAACACTCTTCCTTTTTGAAACCCTTTTAATAGAAGTCCCAGTCATCTGATAGCTTGTAATTGTTTCTTGAGTTGTTCCCTGGAAGAAGAAATTCTTTGAAACTAAATAAAGAGGCATGCATTTTTCTGGTTATGAATCATTGTTTTATACAAATAGTATACTTTTGGCCAAAGACTTAGTTGTGTAATGTGTGCATTCCTTTTGTTTTTATTCTATTCTTTCAATGCTTGAAGTAAAGTTGACTATGATTTTTTGACCACTGACCA

>Gm17ciRNA1504

GTACGATATTATAGTTGTTCCATTTCTGTAAGAAAGCATGCCTTTGTTTTCTGTTCATTTTATTGCATATGGAAATATTTTCCTTTTTCTGAAGTCATTAACTTGGTCAGTCATTCACCTGTTTGATACTTTCTTACTGCCAATATAGATTGCGTTGCTGCCAAGAATTTTTAATTATATAACATTTCAACATTCAAATCTTGTAATTTTTTTATGTTGTGTATGGTGTGACCAATGTTTCCTTGCTCAATGGGACATTCTAAAAAGATCCAATGGATCTCATTTTAAACCTCGTCAAATTGGCTGAGGAAGCCTCGTTTTAATGACTAATTTTTCATATGGTTTCTG

>Gm17ciRNA1505

GTAGGTATTTCTTTCATGTGTTGGTGTATTTGTCCTTCAGGCATTACTTTACCTTTGGTATGAATGCTTTGTATTTGTGATATGGACCTTAACTAGAAGATTGTGTTTTTAGGTAGAGTTGGTTTCAAGATATGTAATTACTTTCCAGTAATGCTGTTCCTTGGTAAACTAAAAGCATGTTTGGTGACAATTTAATATTCCAAGTAATGTTTTCCAAAATTTGAATGATAGAAAAAATAAAAGCTGGAATGGAAATCATTAGTTGGAATATATGATAACTTCCAGCATTCCTATGGGAATGCTTCATTCCCACCTTTCATCACGGGAATTAAAGACTAGTATTGCAGAGTATGAAGATTATTCATTCCTGGAATCAATTATGCTTGGGAATTTTTTTTGAAGTTTGTAACAATTCTATTCCTAATTTCATATTCCCAGGAATAATTTTGCAAAACCTGAAACAACCACTCTTACTGTTTGCATCTAGCAGTATTTTTTATCATAGTATTACACAGTCCACTAAGAACATAACTTGTGGACAGCAGCATAATCATACATGCTACTGAATTGAGTTTAAATTATTAATCTATAAAATTTATATCAAACCTCAACCGTCAAGAATATTTAATTTAGAATTTTCCTAAGCAACATAAACTTCATTTTGTCTTGTTTACTCAGTGCTATCAACTTGCATGTTATATTCTTGCTTGAATGCGTGATTTGTTAATACAGATTGCTG

>Gm17ciRNA1506

GTAACATCAACATCAATTCAATTACGCTATTTGATTTTGTTTTTGTGTGCGTATTTTTTTCTTTTTCTTTCTAGTTACTGGTTACCGTGGTGGAGGTGTTGAATTTAGTTAATTCGCAGTGAAGTAGCAGCATTGGACCCTTTTAGTTAGTGAATGGATGAATGAATTTAATGAATAGATAGGATCTTTGATTAGTCAATGTAGATGAATCATGAATGCTGTTTGGCTTTTGTAATTTACGACTTGCTTTCAGGTAACCCCTACATTGAGCTGTTGCAAACAACTTAGTATAGTGTTAATGTTGTTTTGTTTTAAGGTAGCATTGGTTTTGTTTGTTTCGAAGGACGCTCAGCTT

>Gm17ciRNA1507

GTTCTTTTCATTGCTTTCCTTTCCTCTATTATTATTCTTAGGCTTTTCATTCTTCAATTTCACTAACTGTTTCGTTTAGCCACGGGTACCTTCTCTTATTTTGCTTCTGTTGCAATTAAAACGTTGTATTCCTCTTTAACTGTTAGCAATTACGCCTGTTGTCTTGGATTTCTAGCTAATCGTGTTTCTTTGAAGATAAAAAAAATAACATACAAAATTGAAGGGTCTATTTTTTGAGAAAGAAAAAGAAATATTGGTTGGAGCTACGATTACGGTGGTCATTATG

>Gm17ciRNA1508

CAGTCTTTGAGAAGTACAAAAAGACACGAACATAGACCATAATAGTCCCTAAATAGAAGTTGTTTTTACGTATAACATTAAGGACATAGAAGACAGAAATAAACCAACCACCTATTTATTTTAATATGAGAGTCACATAAGATACTTAAAAAGAAAAGTTTTGAAGTTGAAAAAAAAGTAGATACGGCCTTTAAACGTACGGGTCAAAGTACAACAACCCCTACTAAGAGTAACGTATAACAGAAACTCAGGAATCTGAACTCAAAATACACAAGGGAGAGAGATTAATAACTGAACGTAAAATACCCGATATATATCATTAAAATATATTACTTACACATAGATAAG

>Gm17ciRNA1509

CATCAATAATGTTCAAGACAAACGAATAACAAGTAAAACAAAGTATCCGAAGGGAACTATTATTGTCTGTAAATAATAAATACATCAAATTAAACGGGCATTAAACCGGTTTTACATCTTTGACGACGAACCTATACTACTAATTCTCAGTATGTAGAAGCCTTATAGTATAAAAGGACAACAGATTTCAACTTCTACACTGGATGTATTAACTCTACGGTACAGAAACTGGTTTATACTTACCGGTATCCTATAAAATCTAGGTATACGTAAATGAACCCGTAATAAAACTTTTAGAAT

>Gm16ciRNA151

CATTAAAGAACAAACTGAGAGACTAAGTGAAAATCCATACGTCAATTTCAATCAAACTACAGTAGCATCTCACTTCAACAAACTCTACATGTTGTACACAACTCAAACACTAGACAATACAGGAACTAGACGGGTTTCATAAGACTTATAAACTATTCTTTTTCCACTTTGATAACCTGAGTCATTATTGTTTTTTCGTCCTTCGTAAATGATAGTTATAACTTAAATCTCTTTTAATCTGAACCAGAAAAACTCCCGCTCGGGACCACGTCGCCATTTCAACACGGAACCACTGAACAACCAGTACCCAAGCTTAGGCCTTTGTCGGAGAAACGTATACGTTCCCATTCCGACGCATGTTATAGGGAGGGGGTATGGAAGCGTATCGCTTCTCGGAGACCCGTTACCCCATGCCATCAAATAGACAAGAGTCACAAGACACTTCTTGTCAATAATATGAAATACATCAATAGTATTGTATAGACTACATCCATAATTACAGACTGACGATAACCAAGTTTCATTTACTGTCTATTAGTAAAAACTTTACTGATTTACGTCAGTTCCTAATATTAGTTCTAAAATATATACAAGTTAGTAACAACCAGAGAAGAACGAAAATGA

>Gm17ciRNA1510

CATTCACAGTCAGAAGAACGAGTGGACAATATAGTTGTATTCAACGTCATTGAATAGTTTTGTATGTAGTGAAATCCGACTGTGTTTAGGTAGAAGATTTTATTGAGAGTAAATACGTAATTTTCAAACACGTACTTAGTAGATGATAAGTAACTAACTGACAACGTCGTGTCGGAAGTTTAACTATATACTATACATATGTGATAATTGTGAGTACGTACGTACGAGTGTGTGTAATTATCGAATCATCTACAGTAAACAGTTTTTGACCTCAT

>Gm17ciRNA1511

GTACGTCTCTGGTTTCCTCTCTCTCTTGTTCATTCTTTTCGTAATAGCAACCTTATTAGCCTTCGTTTATTGATTTTTCTCCTAATTACACTTCAATTTTTTCGATTTGTGTTTGTTACGTCAATCTGTATATCTTTTTTGTTGTCGTCGAATCCGAAACTTTTCATTCGCCTTTCTGATTCCGTAAATCGTTTGTTAAGTGCAACATCTTTCCCTGTTCTTAGGTATCTGATTGATTTCTCTCTTTCTTTTTTTTTTTATCAGTTTTGAGGAATTAGGGTTCGTGATCTGATTTGTTTCTTTAAAATTTCCCCGGTTCTTAATTAGGCTCTGCGATTTAATTTCTTTTATCATATTTGAAGAATCATGGTTCTTTCTTGGTTTGATTTATTGTGCACTCAGTTTCTGATATGCAAAATCTTTCCCTGTTTTAATTAAGGTTTAGGGTTTGCGAGTTGTCATTTTGGAAAATAGGGTTTGTGGTTTATTGATTGTGTAATCCGTTTGGTTGTAAGCAGAAACTTCTCTGTTTTAAATCATTGTTTGTGATTTAGCTCTATTTTTT

>Gm17ciRNA1512

GTACGTCTCTGGTTTCCTCTCTCTCTTGTTCATTCTTTTCGTAATAGCAACCTTATTAGCCTTCGTTTATTGATTTTTCTCCTAATTACACTTCAATTTTTTCGATTTGTGTTTGTTACGTCAATCTGTATATCTTTTTTGTTGTCGTCGAATCCGAAACTTTTCATTCGCCTTTCTGATTCCGTAAATCGTTTGTTAAGTGCAACATCTTTCCCTGTTCTTAGGTATCTGATTGATTTCTCTCTTTCTTTTTTTTTTTATCAGTTTTGAGGAATTAGGGTTCGTGATCTGATTTGTTTCTTTAAAATTTCCCCGGTTCTTAATTAGGCTCTGCGATTTAATTTCTTTTATCATATTTGAAGAATCATGGTTCTTTCTTGGTTTGATTTATTGTGCACTCAGTTTCTGATATGCAAAATCTTTCCCTGTTTTAATTAAGGTTTAGGGTTTGCGAGTTGTCATTTTGGAAAATAGGGTTTGTGGTTTATTGATTGTGTAATCCGTTTGGTTGTAAGCAGAAACTTCTCTGTTTTAAATCATTGTTTGTGATTTAGCTCTATTTTTTA

>Gm17ciRNA1513

CATTAACAATACCACAATAATACAATTCTCACAAAACCCCAAGTCGAGCACAATCAACACGGTGTGAACACTAACTGGATCTAAAAGTCATATTACTCCGGTAATCATCCAGACAAAGTATAGTGTTGTATTACTAATAAGTTAAAGTCTCAGAATAATAACTATACATCTTGAGAAAAAGGTAGGTTAAGGTTATTAAAACTGTCAAAAGTTACACCATGTTCTAAAATACTTCCAAGTATTTTTTTAAACCGGAGCAATTTAACTCTGTTTTCTCACTGTTCCATTGTGTACTTCAAGGAGTATTAGTTCTACCCATTGCAGTTTGCACACTGTGGACCACCTTCCTCACTACTAACACTTTAAGGTTTTTCAATCCCATCAAACTTCAAACTTGTCTATTTTTACGTTGTGAAACAGTGTTCAAATAGTGTCATTATTAGCATATTTTTATTTTGTTCACACATCCTTTACAGATAATATTAATATTTATTTACTTTAATAATCCTTATCCTTAAGTTCAAATTTATACTAATTTTTTTTATTATCTTAGAACTTCACAATAGTCAATAAACTAATCGAAAACTCTACAGAA

>Gm17ciRNA1514

GTAAGTTTTTCATTTGTTTCAAATCTTCAATTACCTTTTTCAAGGTTTCTTAGTTTAGAACAATGATATCAGCTTTTCCAAAGAATAAAGCAGACAGTGTTATATAAAAAATCGGTTAATGCTTGATGGACAGAGGACATACCTAAAGTTTTTCCTCAGACATCTTTTGCTTACTTAATGTTAAGTCTTACATGCAGCAATGTTGATAAGGGAACCTTTTCACCTA

>Gm17ciRNA1515

GTATTTGAATATAATATATGTAATATAACACCTTTTTTCCCATAACCTTCTTTGAATTGGTCCTTGCTTGAAGATCTTGCATTGGGGTTTTTCTCTGTCTGGAACTTACACAGTCCACCTTCCCCTTCCATATTTTATATTGATCTATGAAACATACGCCTTGGTCCTAGTAGACACTTTGATAAAACAATCTAAAATAAAATTTGATATTTAATAATCATTTGGGCTTGTTGCAAATATGATGATAACTTGAAACTTGTACATTGAAAATGCCTGTCTACGCAACTAA

>Gm17ciRNA1516

GTACTAAAACTAAAAGAAATTGGGAATAGTTCGTCAATGTACTGTAGTTGAACAGAATGCATGTGTTGCCTAAAACAACATAAAACTAGAATTTGCAGTTCTGTATATGTTTTATTCTCTATTTTTTTCATGATTTTGATAGCCTAGATACGTGATATTCATCCTTTATTATCTACAGGATATGTTTCAGCACCCATTGTAATTCCATATTTGGTACAGTTTTTGCTGTGTAATGCTCATCAGATTTACTTGTTAACTGTGAATTGGTTGAGACTCTTAATGTCCTGTGCCAATCACTATAGTGGAGCAAGAAATATTTAGACAATTTATGTAGATCATTGTCAAAGAAATACTTCTCATCTGCATTTTGTGCTTTAGACTATATCATTTTCTGAGTTGCTTCATGTGATGTGACTAGGCACTAAGATTTAGTCATGGTTTAAGTAAATATATGTGGGCATGTGCTAGTCAATCAAAAGCACAAGATCTATTAAAATAATAATAAAGCTTGTGCTGAAGTTATTTAAATATTATTCTGAGTTTTTTTTATTTGTTTTGCCTATTGGCATTGTGCTCATGTAAGAGTTGTTTTAGGGAGATATTGCATAGATAATCGAGTCCAAGAAGCTTCAGACAACTATGGCTCACAGATATGTTAATTAGAAAGATTGTGATTATTAGAAATGTTCATATGTGGCAGCCATTTTCATTTCATGTCTTAATCTCGTTTTTGATTTATGGGCAATATTTGCCTGTAAAATATTCCTCTGGACCTAAAAGCAAGAATCTTGTCATATAGGCAAGTTCTGGGACAGGTTAATATTGCATGGTTTACTGTATCCTATTCATGAACAGACTAATTTTTGTTGGCTTCTTTGTTACTCGAGTGGAGAATTGAATTGGTCCTCTCTGTTACGTTTAATGTGCACAAGAAGTTAATAGT

>Gm17ciRNA1517

GTAATAACCTTCTCTTATCTCTCTTTTCTTTTTCTAATTTGTTTATTTTCTCTGGTCACTGTCTGAACAATTGTTAAATGGTTTTTTCTTCAATCATACACATTAACACGAATTCATGTATAGCTGCCGTACTGTTCTTTATTTTGGCATTTTTTTTCTTCCGTTTTTTTTTTTATGTGATCAGCGACGTTTAGTAATGCAACAATAAGAGTACGCGTGGTTATTAGCTTGTACTTTGTGCTTGGGTTTTATGTAGCATCATCTGA

>Gm17ciRNA1518

CACTCTAACTCAAATAAATAATACTAAACGTCAAAGTCAACCGAAATCTCTACTAACCTAAGATTAAAAGTAGAGGACAAATGTATACTTCACATCAAAATTTGGGCAACGATAGCCTGTCCTTTCAAACAAAGTTTATCATAAAATTAAAATTGCTCTGTGAAGGTTAAAGGTTTATAATCGAAAAGATAGTCGACTATATTATAAAACTAAATAGAAGATATACAAGTAGTACTAACCGTAAAAAATTTATAATAGAAAACGTCAATATAACTATAAAAAGGTAGTTAACTAGTAATACCGTACTAACATCTAAAACTAACCACTTACTAGAAACGAAT

>Gm17ciRNA1519

CGTTCATAGAAGATGACTTATGATTTTAAATAAAGTATAGAATTACATACCAGATATTAAACGACTAATCAATATACGGTTAACAATTCCTTTATCTAAAACCGCTAATCCACTCCTATCGAAGTAGAACTATCGATCGTTACTGTTTCTTTGGT

>Gm16ciRNA152

CATATTAAGAGAGAGCAAACGGTTCGAAGTGAACTTGTATAGATAATAATTGAACGAAATGGTAATACTACGAACAGTAACTTGAAATAAACCAGTAATCGTGTAATACGGTTTACCGTAACGAACAGGGAACATCGGTTCATCAGTTGGTTCTGATCACCCTCTAAGTCTCTTCCGAACCACC

>Gm17ciRNA1520

CATTTTAAATTTTTAATGGAACGTTCTTACTTAACCTTTTTTATAACGAAACGATAGACAAAAAGTGAGAAAGGGAACCAGTAATAGATTGTATACGGTACATGGTTTGACAGAAAGTAATGGTTTGTATAAACAAGTTATCGGGACGGAAACTTAACTAGTAAGAGTGTGCTATTTTACACGGTAACACGAGGTAAAGTAAAAATAAGAACAGGAGGGTAGGAAGAATTATGACATCAAGGAATTATGGAAGCCACTAAAAACCACTTAACACTAAAATAGATATCAAAAGTAATACAGAAAAAGACTAATATAGAAGAAATCTCACTGTAAGTTTTTACCTCTAGCTATACACTACGGTACCACTTAGATGTACAGGACCAGAAATACTGTTAAATCAAAGACAATGTGTATGATGATATACGACTTCAAACAGGTTGAAAGGGACTCGAACACCGAGTAAGAAT

>Gm17ciRNA1521

CATCAAACAAGTTCAAGAGAACAGCTCACTATCCGGAGAGTATAAAAATATACGTACAAGGAAATAGTGTAACATCAATTCATTAAAACCACCGCTTCACAAGTAAAAGACAAAAGAAAGTAAAAGACTTTTATTAAAAGTAAAAGTCTAAAAGTTCTAAATCTTTTGTACATAACTCTTCTTTCATGAGATTCCACAGTGGCGTAAAAGTGAAAAAGTCTTTTCTAACTTTTGCAACTAAACAACAAAAATCAAAACCCGGACAAAAACTTTTATAAAAGTCTCTTTTACAAAAGAATTAATATTTGTTTGTATAAAAGTGAGAATAAAAGACAAAAGAAACTTTTACTTTTGTCTTTTGTGAATTTGGTTCGCAGTGGAAATAAAGAGTTCACATACAAAAGGAAATATAAGTACATTGGTACGAAAAGATTATCACAGTCTTTACTAGTTAAAAAAGAACTAAATTGAGTAATAAAAATAAGTAAGATGTCTTTAATAACTAAAAATCGGTAGAAGATAAGAAGGTACAACGAAAGAATAATGAAAGAGAAACAAAAAGTTACTAGGTAACTAAATCCAAAACTTATCGTACGGAGAACCTTAAACAATGAACTCAAAAATTTACCAAAGATTAACGGAAGA

>Gm17ciRNA1522

CATTCAGGTGCGCGGTGATACGAATGAATGATGTATTCTTTCAATATTACATGTAATCCGTGATTAGTGATATATTCGAATATTAAATGAAACGTTAACTTTTACTAACTAAACTTAAATCTCTGGTCACTAACCATTTGATGCTGAAACTTAATATTGCTTTGCTGTTTATAGTCACCTTTAAACTTGACTAAAAAAAAAAAAAAAACAGCTTGTACAAAACCTCACACTTTAAGGTTTCATCCTTCTGACTTGAATCAAAAGAAAACGCTAGCCCACTCCAAACAAGACTCTCTCCATCACCTTTAATTTACTATTTTTACCAACAATATTATATGACACTTCATCAGCACTCCGTATAATCCACCAC

>Gm17ciRNA1523

GTAAAGACTTATGTTTCATAGTTTAATTTATTCTCCATAAGTGTCAGTTATATTTATTATTTAACCACATTTAGTTCTGCTTAAAATTGTTTTACTCATCCAGCCAAGCAAATATTTCATGAGGTTCAAGATGCCGAGTTTCTTATTTTTAAGCAATACATGGATGCTGCATTTTGCTACTTGTATTTTCTTAATAATTTTTCTCCAATATATGTTTGCGGTAAACTGTATGCAAGACGTTATACAATCTTCTTTTTCTTCCCTTGAAAAAAGAAAAGCATTAGTGCACTTTAGAGTACCTATACTTGCACGTACAGGTATGAGGTGCCACACAAAAAAAATTTAAAAGATGGCAATGTGGAATGTCAAAAAGTGTTAGCACCCTTGATATTGTTGGTCTTATCTTGTATGCCAGTGTGCTTATGTGATTAAGGTGATCAGATCATATATGTTCTAATAAATTAGAATTTTAAGTCCCATACATGTACTGCTGTGCAAACCAGAAAAGAGGCACCTTATTGACCAAGGGAACATAGAGATGATATGCAAATGACTTATATCAGGCATCTTGTGTGGCAGTGTTGTGGCTTACTTGATTAATATGCTGACAGCTGTTTGGTCTAGAGGGGAGGGACACCTTGGTTGGTTCACAGGGGGTGGGGAGGAGAAATAAACAATTTTATTTTTTATATACCTAGAATGGTAAAAAAAGTTATTAAGGATAATTTAGTCCAGTTTCTGATGTGGGAGCTAATCTTCCTTTTCAAAATTTTGAATCAATCAAATTTCTCCCCGGCCCCCCTCTTCATTTTCCTCCAACCAAACACCATGTAAGGTGATTGGACAAGATTTTTAAATCCCCAATATGTATTGCTTGTTGTCCAAACTACTTAACTGTCAACATAGAGCTGATATTCAAATTATCTACATATAGTGATTTCTGTGGAACTACGGATTCAATTGATAT

>Gm17ciRNA1524

CAAACAAAAGTTATATTTACTGATTTAGTTTGATTTTATCTAATAATGTCTTACTCGGGGTACAATTTACACTTTTTATTTTCTTGACTTAAACCCGTCTTGCGTATGACCCACTTACTTTACTATTCCCCCTAATAATATACACTTATTCATAAGAAGAGATGGTTCAAACTATCCCTCGTAGAGTAAAAGTAATAATTAAGACACCTGTAAATATACT

>Gm17ciRNA1525

CATGCAAGGGGCAAAGCAGAGAAACTAAGTGCCAAGGACAAAACCTGGGGAAAGTCAACAAGAACTAACCAAATTAAATTAGTGTTCTAATCCACTTAAAGTACAATTTCATACGTAGAACCCCTAACCCCACTACGACCCATACACAGCTAACAAAACCAAGACCACACAACGAAAAGGATAACGATTCAATTGTAAAATAAACTAGAATAATACGTACATTTACTATACGTACGGTTGAATGTCATCTGACGGTAGAATATAGACCCAAGTTTTACTTACTTCGATAAACCTACTAGACACGTGACGGTACAGGAAACAACTCAATAAGGACTAAATCCTCCATCGAACCAGTACATAACTTACGTGATATCACATATTCATATAACCCGACAGCATAGACAGAAT

>Gm17ciRNA1526

CATTCGTAATGGTAAAGGAACTTGAGACTAAACTACCTAGAGAGAAGGGGGCGAGTGGGGGTAGTGAGAAACGACAACCCGACGCCAAGAGTCTAAGTCAATGAAAACACAGAGTTTTATACTAAAGACTCATGTCTAAAGTTCTATTTTTTGCTATAAAGTTTTTTACTTAGGGGGGTGGGGGAGTTCCCTTGATGACGTATGTGTACTTACTTAAACAAACGAACGTTCGAAACACTACTAGAACTCAACGTTTACATACGA

>Gm17ciRNA1527

CATTACTGTGTATAACAGTGATACCACACACTACTATGAAGAGTGTTTACTGTAAAGAGAAAAAACAATGATAGTGAGACTAAATTACGTATAACAACTTAAAACCTCGTTTGCTCTCACAGAGTCTTTTATCGTGACACAGGAAATAGTTAATAATATGTAATGATTTGACAACATTCGGATACTGTTTAAATAAGGATTCTGTCAAACCCTAAAGTCTGGGAACAACCATTCTATCTAACGTAAACACAGTACTTTCTTCACTAAGCCACAAAAACTTAATTATTACAGTCAAAATTGTAAATATACCAATAACGTAATCAAGTTGAGTTGGGGAGGGGGGTTCATACCTAAGTAAAACCACACAACAGTTCTTTGATAAAAAACTTTCACGTGCACTATTTCATATAGGTTATCTTTTAATAATACAAAACTCCGTTAAGCTACGGATTGTTCATTGTTGTTACTAAACACATTTGTTCCCAGAGAGTGTACAGTAAAACGCATTCTCACCTACTATTTTTCATACTTAGTCAATGTCTTTTTTTCTTCTCGTCTAGTACCTATCCATCTCACCGTTGAATGTTTTAAGTTTGGATGTCCCACCGTGTAACTTTAACCCGGTGTGACGACCATCCTTTAACTAAATTGGGAACAATAATACTCTAATACTTTTATAATCCCAAAAAAAATCGTCAATGAACACAACGTGGGTTACATTTTCCATATTTATACAAAAACACCATTATGAAATTTTCTTTGTCATAAAACATGGTGTCAGGTGTCGATACGTTTCAGAGAAAAAAAACACACACACCCACCCACACAACAGAGACAAAGGAAAAGGTAGGTTAGGGATCTTAAAGTAGGAAAACAGAAGTTTATTTGTTTTTGTTTTACTACCTGGATCAAAACATCGAAAGTAAACTAGA

>Gm17ciRNA1528

GTATGCTTTTCTTTCAAACTCCTTTTCCTTTACTCTCCCTTCTCACTTGTTTTTTCTTTTTATAGTTAAAACCCTTTTCCATTTTCCATATTTTTAACTACTGGATCTATCTCTCTCCTTTCTATTTTTTCACAATATCTTCAAAGAACCAACTTCCCTCCTAGCCAGGGACATATTTGGTCATGTAACAATCATAGGCACCACATTTCTCAACATGACTTACAAACATCTAGTGTAGATTCATGTGTCCTTTGCTCTTCAAACCGTGTATACACCTTTATAAATGCAAAAAGCCATAAGCTTTTCTCTA

>Gm17ciRNA1529

CATTACCTTAAACGATTTCAAGATCATCTCGTCCTCTCTTATAATTGGTCACGTGGCAAGGTAGACTATAGATTGACACTCCGTGACAGACAAACACTTACCTTCTGTGTTTCATAGACAGACTTATCAGAGGTCAGGTCAAAATTATTCGACTATTACTTTAAGGTAAAAACGCCGTCATAAACTTAGGTCGATTTTAGGAAAGTAAATTGGGTCAAAACCGAAAGTTATATATGAAGAGTAAGAAACTTAAGTCAATAATCCCTTCAAGGAATTAGTTAATCTCTCAAACTACCTGTTACCTATTTTGGACCGAGCCCAAGGTGTACATAGTGAACTATGACAATAGTTAATATCAAAAGGGAAAAGTATTAGGGACTTTTCTCGTATTTGCACAGGGATTTCCGTGGCCGTCTTGGTCTCGTTACTGAATGCCCCACGTCTATCAAATCTAAATACTATGTTTTGTACATCTCTACAAAAAGTTACTGAGGTCAACGGGTGCAACAGTAGTTACATCAAAAAACGGGTAGAATTTCAACAAATTAGAACCGTTCGCCTAATACCGTTTTCATCTCTTTATGTACCTTGTAAAAACAACGGAAATAGGAGATGCGGACAATACACTCTTTATCTCCTCTTTATCTCTCCGTGATTTCTAGTGGGATCCGTCCAGGTCCCGAACGAACGTTACATCCTTATATTAGAATGATTACGACCACTACTAAAATTAGATTCTCAATAAAAATATATCGGACAAACAGAAGTTACGGAACGAAAACGACAGTAAATACGCAATTAGAAAATGAAAATTAACTAAGCTCCCAACCATGAG

>Gm16ciRNA153

CATTGGTGGGTTGGTATTTGGTAAGAAAAAAGAAGCAATTTGTAACGTAGCAGCCTTTCTTTTCTTTTCTTTTCTATTTAAACTTATTAGACGGCTGCGAACAAGAACAAGAACAAGACACAGCGGCGCAAGCATGCGTGCGGAATAAAACAGCAAAGGCAAAAACTGGGACACCGCAAAGACATTTATTCAATGTAAAGAACCCCCACACGAAAGAAGACAACCTTAGCCATGA

>Gm17ciRNA1530

GTAATACATGATGTTGCTGTTTTCACGGCTTACTACTGTTATTTAAGTCATGCTATCTATCCTCTTATACTGATGCAAATTTATAAGAACAATTCAAGACTATTTAATTAGTTTATGGTTGATCAGTTGCATTAGTTTTTACTCCTAAAAACCATTTGTTTTGTATCATTTTTGTCAATATAACACTCAATGAGCTCAATAGGTGTTTAAAATTTATTAATTTTGGCTGGCAATACACTTCTCCAACT

>Gm17ciRNA1531

GTGTGTAGTCAATACCTGTAAAATCCTTTACAATTTAGAAATTATTGTACTAGCATGTGTAACACAGCGGAAGCCTTGGTGCAAAGGCATGCAATGTAAAAATAGGTTTCATGTGATGGGGAATTAGTGTATCCAGTCATTCCTAGAGTTGTTCAATGAGGGTAATGATGACCTTCATGTGGCAGCATAGTGTGTGAAACTGTGAATGAGAAAAGAAAAAGATGAGATAAAACTTAAAAGTATTTAATTTGGTCAAAGAGAAAGCCCTTGTAGTT

>Gm17ciRNA1532

CAAGAAGGAAGTTAGGCAAGAGACGAGGTGAGCTAAGCAAAGCAAAGGCTAAAGAGAGAGAGAGGAGGGCGAGCGGCTAAACTAAAAGTGACTTACTGATGTATTAATACAACAAACAGCTACGATAGAAAAAAGAGGCCACGAACTAAACTGCTAACGGCAACCAAGACCTCGACACATGAAGCGACTCTCTCAATAGATCGACACAAAAAAAACACTTACAAAGATAAAGGCACAGAAGACCTAAAGTTCCTCTGAATCCCGAAAAACAAAAAAGAAAACTTAACACACTCGAGACATTAAGCCACGTCTTTGTTTTAAAGTTTGCACGAACGCATGACATGTACACA

>Gm17ciRNA1533

GTATGTTATATTAGTAGAACTTGTATGTTCAGTTTCTGCCCATAATTTGTTTATCTCCAAAAGGAGACAATTAATTTTAAGTAATATGACAAATATCTTCCTTTTTCCTCATGGTATACACAGCTTCTTGTCCTTTGCAAGCTCTTATTCTTGAGTATTAGATCCTAAATCTAATTTATTGCAGAAAATATTAATTAGGATTACCTTTTGTTACTTTCACTACATAATGTGATCAGGTTGCCTTCATTAAATCAATTCAATCTTGTATGACTCATGTATTAACTAGATATCGTATGAAGTGTAATTTTGGACTCAAATGATTTTTGTGTTTATAATAGTTTACCTAGTTCCTCTGCCGAGTGCTACAGATT

>Gm17ciRNA1534

CAGTACGTAAGGGAAACAAAGGTGAAAAACTAAATTAAACATAACAAAAAGTAAACTTATTAAGGAACTTAACGTTACACCGTAACATGGACAGACAGAATCCTCTTCTTGTATGAGACGTGAAAGTAGACGTCACGTATGAAATATATGATGTATAGAGGTCTAACCACGTCTTTGTTGAGGCTATCAACAGGAACAACTCCGAAACGACGTAAAAATCCTAATTAGAGACAGTTCAAAATTTTTTCCAGCGTGCACTTAAACGAACGTTGTTGTACCAAAAAGTTCACAGACGATGACGTTAACCCCGACGTAAACAGGTCAGTAAAGGGCGTTATAGTTATTTGCACGCCGTTTTGGAACTGACTATAAATTTTGGAACTTAAAAGACATTTATCGTGATCTAAAATTGAAACACTGAATAGACACCGATACACAACTACCACCTCTTTCATTCTTGTTATTATTTCCCCTTTCCTTCCAAACTCAAATTGTCAGAAGAAACGATCAAATAATAATTTCCTCTACTACTTAACTACTACTACTTAAAAATACACTAGGAGGAGGTTTGGGCAGAATCAATAGAAACGTTTGTGAAACTTCAAAGTAGTCAAAACATACTAATATTGGGGTGAGGGGAGGTTAAAAAAAGAAGATAAGGTGACAGAAGGGAAATATTGTAGGTTTGTATACGGTGAGTTGTTTTCTGGTAGTCCTGTGGTAATTCATTGGTTAAGTAAAACCAAAATTAAAGAAAAAACGAATAATAAGT

>Gm17ciRNA1535

CAGTTTGAAATCAACGAAAGACTATTCGGTAATGAAAGTAACGGAAAGTGACCCAACGGTTTTTAAATGACATATTTAACATAAAACATTATTTGACAACTCGTATAAATGAACAGTTAATAATTCCTCTCACTCATTAGGTCGTACAACGGCACGACTAGTAAAAGTCTGGAAAATTTAAGATGTATTAGGGAACAACCATTAATAAAAACATTGTATGGACAAATACCTAAATCACAATTAGGGTGACAGTCACATATTTGAAGATGTGGTAGTAGGTTAGTACACAACGGTCTATACACCTTCAAAAAAAGAGGAGAGTGCATCAATAACAGTGTTTGGAAACAGTAGAGTTGAATACAGAGATCCCTACATTGATATAGTATACAACACAACATGATAT

>Gm17ciRNA1536

CATTACACGAAGGAATCGCAAAAAAGGGTAAACTGATGAGCCCAATCTACTACTATTAACTAAATCAAGACATAATAACCTTGTAGTTCAATTCTTCATTTGGAACAAAGACAACTAGGAGTTTACATTACCTTTTTTGAAAACTTCACTTCAGAGACGGAATAAGACTTTAAAATTAAAATTAAAACAACCAACCACTTCTAAGGGTTAAGTAATAAAACTTTAGCATACGTTATTATACGATTAAAAAAAAAAAATAGTCGTTATTATACGATTAACCAACTTCTTAACTCGAAGTCATAAGTACATTAAACGACGGTAAAAGTACAACAGACCAATTAATGTGAAAAT

>Gm17ciRNA1537

GTGAGCGCCTCCAATCATAACATATTTTCCCCGAAACTGCTTTTGCCGTTTTCATTGCATTTTTTTTATTGTTGAATTTAATTGATTGCTGCAATTCGTTTGGCGATCTCTGTTTCGCAGGTTTACACATCATGTTCCTTAATTGCACCATTACTGATTGACTGTTTTTTTTTTAATAGAAAACTGTTTAAAATTGGTTTATGTTTTCAAAATTGACTTGTAGTAATTGCTTTTGTGCTTATGCATATTGATTTAATTGAGGATCACTAGTGAAGAATTCTTATGCTTTGCTGATTGCATGTCATGAATATTTTTACTCATGTTTATGATGTGTCTGGGGTGTTATACTGCTGCAAATTACCATTTTCTAGTTGGA

>Gm17ciRNA1538

GTAATTAATCTCCTCTTTATCTACTTATTAATATTCCTTACATGTTGAGTTGAAAGAGAAGAAATTGTTTTTATTCAATCTTATTCGGGGTTACAACATGTGTGCTCCTATATATACAATGTGGGAGTTTTGATTGCATCAATGTTTGCAATGAAATTCTGATGCTATGCCGAGACACGGTCAGTACAAACAAAGTGAGAAATAGGATGCTTGACACAACCCCAAAATACTATATTCTATATGAAGTTTAGGTTCATAGTGTTTCATAACCGTAAAAAGTTGGTGTGCAATGGCATTAAAGTCTTTACATTCTATATTTCTATTATTCTTAGTAACTTTGTTTCTCTTGTTAGAAAAACTATACAAGTGGAGAGAGAAAAAGTTGTCATAAGAGAAAATAATGCTTTTTCACGGGATACAAATTTTATTATTGTGTGTTTCACAATAACAGTTTCTTTACGTTTCATTTTCAGTGATTGCTGATAAACAAACAGAAATGAAGGAAAAATATCCTAAATACAAGCAAAGTTGTCCTGTTGAAACAGGGTACCAATATAAGCAGCAAACATTTATTTTTACAAAAAAGTAAAGGCTTTGACTTCTCTACATTATGACTCAAGTGCCAGTTTGTTAGGAATGGTGGAGAGCAGCATTCTGGTGTTTCTAGGTCTTAAGAATGTTAGATAGTGTTACTTAGAGCTGTATCCATTTCGGTTGAATAGTCACTGCCAATTATGTATGCTTGTCAACTGCCACATTTTGTTGTGTATTTGGTCTTTTGAAAATTTTCTCTGTTTCCTTTTATGGTATTTTTCGTA

>Gm17ciRNA1539

GTAATTCTGTATATATTTTCTCAATGATGATGTCTTTCATTATTTCATCTAGGTAGGAATAGAGATGTGAGACATTAGTGGCTTGAAATTTTTCTCCTAGATCTGTAGGTTCTAGAATTTAGTAATCTGTATTTTTCTTTAACCGTGTATTTTTCTTTTGTTATTTGATCCAATTTAGTGGACTTA

>Gm16ciRNA154

CATTGGTGGGTTGGTATTTGGTAAGAAAAAAGAAGCAATTTGTAACGTAGCAGCCTTTCTTTTCTTTTCTTTTCTATTTAAACTTATTAGACGGCTGCGAACAAGAACAAGAACAAGACACAGCGGCGCAAGCATGCGTGCGGAATAAAACAGCAAAGGCAAAAACTGGGACACCGCAAAGACATTTATTCAATGTAAAGAACCCCCACACGAAAGAAGACAACCTTAGCCATGAC

>Gm17ciRNA1540

GTACGTATGAACATTGAAGAACTATGTTTTTTGTTATGGTGGTCTCTGTGGCTTTATTTTAAGGATGCAATCATAAAGAGCGAGTGTATTACTTCATACCCATCTAGTTGACACAGAAGGCACAAATGGAACCATGGAATTGTTGGCTTAATATGCCATATAAATATATTTTACCA

>Gm17ciRNA1541

GTAACCCCACACTTCAATCTATTCTTCTTCTTTTAAATTCAATAATTATATTATCTATTATTTCTTACTTGTTCCTATAATGCTGAAATTCTCTGCATATTCTGAATCTTTATCCCCTATTATATTCCAACTTTCCAAGGGCTAAATAAAAAGTCCCTTCCTCTCTCTCTCTATATATGTGTACGCATTGCTATTAGCTTTACAGTTGTTTTCATTTTATCTTTGGTTATGTGCTTCTAGTATTTTCCTGTGTCTAATCGGTTAAAACATTGTCTCA

>Gm17ciRNA1542

CATTCGAAAATACCAATACCTTCAATAAACGAAAATCGGTTACCGGTTTAAAGAGGACATATAAACAGACCCTGGTCTAGCAGGTTCTATGTACATTAGACGAAACTATAAACGGAGTAGGAATTATAAATTACAATTGGAACTTCTTATAACGTTTCTCTAATAACAAGTAGTATATAGACTCGCCTTAAACCTAGGTGAGTACAATCTGCAGTACAACCTCTCTTTTTTCTGTGTTTTGGTCTGTTCAATTCAAACTAAACTTAATCTGATTTTTTATTCATTGTTCTCTCTTAGGTTTAGATAATCAATTTTTAGTAATTATACTTTTTCATCTTTAGGTAGGCACAAATAACTAAATCTTCCCCCCTTTTCCTTCGTGTGTGACAATTTTTCAATGAAAATGGCGCTCATTGAATAAGAGAGAACTCAATAAAACAAACGTACAAAGAATTTGTCAACTAACATTATAGATTAGTTTAAAGTAAGTAACCTAGATAAAATTTTAAATCAATTAATTAGTAACCACAATTAAAAAGAAACTTTTCCTTTTTATTTAAAGTAATTGTATTATTTACAGTTCCGTTGTTTGACATTGGTTCTGTAAATTCCGAGATCGAGAACGAAGGACAAGTAGAGAGAGAGAGAC

>Gm17ciRNA1543

GTATGTCATTTTTTTTCAATGCTAAGCTTACTCACTGTCCTATGTGTAATTGGTCTTGCTACTTGGCAATTTAGCTCACACTGACCTATCGCCTGCCTTTGCTGAAAGAGACTGTGAAGTTGATTCAATGGGCAATGGGATTCATCCCATTTATTTGAGTTCATACTACTGGCTTTACTTGTAAACTTGTTTCCTTGCTTGTCTGTGCTCTCTCGCACACTTTTTTTGTTGGCCTCGGTCATGGTAGCTTAGTATTTACATTTGGCTCATGACCATAATTTATGGCAGTTTTGCTGAAGTATTCTTGTGTTGATCAGTTTTCAGTAGTGTTGGCAGATAGATTTTCCTGGCAACGTATTTGACGTTTGCATTAGCTTTTTTTTCAGTAGTACATGAGAGTCACTCTTTTCTTTGCCTTTGTTTCAGCATTGCTTGTTACAACATACAAGTAGCGTGATCTATTTAGATCCTCCTGCAATTAATGTAGTGCATTGTATTGTCAATGTTATAATTCATTGATTACAAGCTTA

>Gm17ciRNA1544

CATAAGTTTCGATCCAATGAATAACGTAATATTGAAATACTTTATGGCCATCTAACACTTTAAGTATAATGTCGGTACTCAGCTCACCCTTAGTTGATCCGACAGAAATTAAATGGCTTATCAAAGTCACAACAGTCTAGACCAAAACTATATTAATACCTTAAAACTATTAAACGGTTCACGACCATATAGTTTAGAGACGTGGATTCTTTATTATAAGTGATCACAAAAAAAAGAAGTTTTAAACCATGTTACGACTTGAATTTTACCTTCTAAATACGGAAAATTCATACGGTACGTAGACCAACGTAAATGTTGAAACTTTAGGTAAGTTCTCCTCAAGTACAAGTACTTATAGACACTACGGTATATATACTGAATAACATCGGAATCGTAGTGACATAGTTATTTCAAGTACCTATATCAATAAACTTTACACTTAGACTTATTATTTGTACGGTATACCAGATGTTTAAGTTTAGTAACAACTACGAACCAATGTTTTACGTCTACCAACATAGATT

>Gm17ciRNA1545

CATTGAAAAACTAGGATTTTAGAGTCTAGACTAAAAAAAGAAGAAGAACAAAACGCCAAAATAGAAAAGACCAACATCTAGACCACCCTAAACACATCTAATCCATACAAACCAAGTCCATTGCATAAAAAACGTAGTATCCTAAACACACTGCTAAAAAAGACAAAACAAACTACCTAGACAATCCAAAAAGGGAAAATTAATTATAATGCCTTGACACATGGTCATTTATTCAACTAGACCCAAGAAAATAAGGTAAAAAAACACTAATCAGACACAATATAAAACCATAACACGATCTAACGACACAAAACACTCGTACCACAAACACTAAGAGGAATGTCAAAAAAACAGGAACGATATACTGCAAATAACTGTACTTAGTGACAAAAACAAATGTATACATAGACCTAAAACAATATTTGTATGTAAGACACCAAATATAGAAGCCTAGAAAGTAAAAGTTTATTATTATATTATTTCAAAGACCTACTATAGTAAACATTTCTCCCCTTGACAATACATGAATACATTCTAGATCAGAAACTTTGGAATATAATCACCTTAAATCTTTTACTTAACAGACAGATAATTGTGTAAGATTATACTTAATACCTTTAAACAAAGTTCTGAGTTAAAAATAATACGAGGGCTATTAAATAACACACATGACCTAGTGGGAACAAATTAAGTAACAGTCAAAAAAAACCCAATATAAGACTCCACCACCACTCTCGTATCCAACTCAATTACTGTCACCCTACTGCAAAACAAAAATTGATACTCACCATGTACT

>Gm17ciRNA1546

GTGAGCTGACATCTGCCAAATCTCAGTAATTAAGTGATTTGTGAAGTTTTCACTCTTCATTGGAGACACTTTATGCTGTATGCAATTTTTTTTCTGGTGATATAATTTGACAATTTGTGGAAATCATCCAATGTACAATAGGAACTTCAATGTGCTGGCACTTAATTCATTCTTATCTGTTTGGTTGGTTCTGGGCTA

>Gm17ciRNA1547

CATGTAAAATTCTGATATATTCAAATGAAGTACTTCGAAACCTAGTAACTAATCTGAGAGACACACCAATCCGAGAAAAAAATACTAGTTTTCATACGTGAACCATCACCTAGTAAAAAGGACCAAAGATGATACGTTTCAAAAAGCATATGTCATTAGGAATATTGATAAAAGGACCTTAACACACGAACCTACCGAAAAAGACCAGAAAATGAAAATCAATAAACTCCACTTATGCCATCATATACATACACGTCAAACTAA

>Gm17ciRNA1548

GTAGTGTGTTATAACTGATTGCACTTTTTTTTTTCATCACCCCTTGGCATAAATCCCTCCCACCTTGGTGCTGATTGGAGTTTAATCCTAGTCTTCGCACTCTAGAAATGACTTTATAGCTAAACTGCTAGTGTTGCAATTGATTTCCTTTATTAACTGTGTTTGGAAATCCATTAGATCCACGTCGAACAGAAGTTTTACACTTTCCAATGCACAAACTGAGAAACAAGGTGCATGTTGCATTGACGTTTAAAGTCGATTTGGTTTAACCCAATTGATATCCAAACACACACAAAGTTGTTGACACTTATCCCTTTATAAGGCATTGGTTTCCAATATTGTGTCATTTAAAGAACTGATCAACTGATTATTTGCATTGAATTTGAAGAGAGAATGTTATGTGGAAATGATAGACACTTTACCCAGTTTTGGAAAGTTAAAAATTTTCAGGTATTAAAAGTGGAATTTTGATCAGCAATGACAGCATTAAGAACTGCCCCACACTTGGTCCATGAAAAAAATTGCATCCCACACTTAGCATGAAAAAAATTGTATTTTATATTGTATTCTTTTCTGATTTGTATCGTGGTTATGTTACCTTGTGCAAATCCTAGCTCTGAATTCATAATTCCCTAGGGAAAGAAAGCAAACTCCTATTTCCAAGTTGCT

>Gm17ciRNA1549

GTACTGTATTATCACTATCTATTAATATTAATATTACCTATAGAAGTCAAGTCTATCCTAGTAAGGCTTTATATGATTAGAGATTTCTATTCTTAATATAATCAAACATGTCCTATGTTGTATGGACTTTAGGACCTTTTTTTTTACAGGAGAGTTGTCTCTATATCATACATGATATATTTGTGTATGATTAAAAATTATGCTTGACATCGTATACATTTGAAACAACACATTAAATCCTTATATACAATCCCCAAAAGAGAAAAATATATCAAGTGTCTTTCTCTTCTTCTGTGGCCAGCAAGAGCATGTCATAACCTTGTCTTTCATGAATGACCATATCATCAGTAATATCAATTATTGTACTGTATGTTACAATTGAAAAACTGATAAGTCTTGTAATGAACATTGAAATACTTTTTCAGCTATTAATTCCATCATAATTTTGATTCCTAAGCTTCCTTTTTTTGCTCCATTCTGTTGTGTTGCAACATCCAAGTCGAATAGATTTTATGCAGTTTGTGCCTTTAGCTCATTGTATTGAGTCCGTTGACCTCTCTAGTTTCATGAAATAGTTCT

>Gm16ciRNA155

GTTAGTTAGTTAGTTACTCATCACTTCCTTTTAAGCCATTTTAAATTGCCATTGTTGTGGTGCTATTTATTGACTTTGGTAGAATCTAGAATTAGTCTGACTAAGCTGGGTATTAGTTGCAACCAAAGTTTTAAATTGCGGTCACCGTTGCAGTTTCGTTTTTGTGATCCTTGATGTTGTGCGAAATTGTGGACAAATGTGGCCAATGCGAGGGCAACCGCTTAGTTTTGTGGATGAAATCACGGTCGTATTTTTAAAACCTTGGCTGCAACAGAGGCCCCTTTCATTGTTTTATGTTGTGACGATGTTGTTCTTTTTCTTTTGAGTTTCATTTTTTTTCT

>Gm17ciRNA1550

CATTCAGGTACTATAAACTGAAAGTATTTACAATTAACACAACACAATATGAACCTGAGTTTAACTAAAACCAAGACTACCTTTACAAAAAACTCTTATGTACGGTCAAACAAAAGATGTAAATTTAATGTACAAAACTTTAACAGAGATTGAGAGATAATGAACATATTTCTAGTTAAACTTACACGGATCTTTTTTTTACGAGTTGTACTTCAATAGTTAGTGTATGAGTTCTTACAAAGAAATACAAATTACATTATTAGTACACCAAACACATGATCCTCCGAGTTAGGTTCGACAAAAGACAGTGGTTAAAACAGGAACCAAGCAAGTTTGAATTTCCAATGGGTACTTTTTTTACCTTTAATAAACAAGGTTGTGGGACAGGGTTAATAGACCCTAAAAATGAAATGGCATAAAACATTAACAAGT

>Gm17ciRNA1551

GTTGGTTTATTTCTTGAGTATCACTTCTTTTAAAATATGCAGTGTCAACAAATTTGTTTATAGTTTGCTATTGTATTTTTCTAGTCCAAGGAAGTGAAGGAACCAAAGTGAAAATTGAAATGACTAGTCTCCACATGTTTTTTTATAACTAGAAGATAGCTACGAAAATACAGGGATCCTATGTCATTGTTACCATCTTTCTTTTGTGTTCTTATTTTTCTGTCCTGCTGTGGTCTTGTTTGTTTCCTGTTGTTTATCTGACACTTGCTCGTTATTTTACTTCCTACAATATCTTCGCTACATGTTCTTCCTCAATTAAATTCACAAACTCATTCTGAATTTGTTGGGTGTTTCTTAAATTGACATCGTGCTCTTGAATCTTGCTTTTGTTCATTTCCATTTTCTTTATCATCCTCCCTGTTTGAATTATTCTGGTTCTCCAGGGTATTATAAAATCACAAAAATCAATGAAATGAAAGAAGTAAAAGTGTGGGGGAGGGGTGGGGGAGAAAGAATATTAATCCTTTTTTATGTTCAAAAGAAGCATAACTTTTTTGTCTTTAAGATGCTGTAAGGTTTTATATGACACAGTATATTCTTAACTTAGACCAAGTAGCAATTAGCGAAGAAGTTATCCATGTACATTGTAAGATTTGACATGGAAAAGATGTGCATTTTATGTGTAACTAACCTTATGGCCACAATTGGACTCTCTGTCCCCTGCCCTTTGGAAGTTGTGAAAGGGAAATGAAAAATTCTAGATGATGTTTAATTTGAAGATTCTGGACATTTAACATTTGCTGCCCTTCTCTGGTTAAAAAGGGGAAGTTTGCTT

>Gm17ciRNA1552

GTATATATATATACATACTCTCACTCGTTTCAAATATAAACAAATAAAATAAAAACTAAGAAAGTTAGTTAATCAGCTTAGTTGTATCAATCTTAATTAAAAATTAAGTTTTTTTCCACTTATCCTTGATTGAAACTTGATATTAAGAATAAAAAGGAAATAAAAAGGAGTTATTAGAACCAACACCTCAATAAAATAAATGATATTTTAGAGATAATAATGAAGTAAAGTTAGTTAATATTTTCTTATATTTGAGAGAAAAAAAAAGAGTGCTTTTTTTTTTTCTTTTTATATTTGAGATGGGAGGGAGTATGATATACTTGCTTTGCTTCTTTTTATCCTTCTTCGATTTCTCTTATTGTTTGCTGTTGTGATTGCTTTGGGATCCATTACTGATAGTAGTTACTTAACAAGTTAACATGTTAAGACTTAAGTTGCTAACAATAATTTACAGTTCTGTTCAATGAGTATAGGAAATGTTGGAAGTTATTTGCATCTACATTGTTGTATTTGGGCCATCAAGGATAAGATGATTCTTAATTTCTGCATATGACTTAAATGGTCCTGAAGCATATCAAATTCTCGGCATTGTTATTACTTGTTTGTCACTGAGCTGTCAAAATATTGTATTATGCTTTAAAAATATACCTGCAATCTTA

>Gm17ciRNA1553

CATGAAGTAAAACTTTATTTACATTGGAGAGTTTGTGACTTACGTATAAAAACGGTTGATTAATTTAAAGGTAAGTTCTCATAGTAACAAACAAAACCTACACGATACAGAAAAGTTACAAAAATTGAAATACAACTCAAACCGTGTGGAATTTTTTATCCCTAATGTATGAGAACCTGATCAAAATAAACGTTTTAACAGACTTTTTTAGTAGAGGTTAAAAAAATGGACAATGGTTTGACTGAGAACTTCTTATCATAAAAAGAACTAGTACAATACAGATAACATGTACCCGAAAGACATTCAAACTACGA

>Gm17ciRNA1554

GTAATAATGTTCCCAACCCTAATTTTGTTTCTGGAGCCTTCTGTTATGCTCAGATTAGACACAGTTAACAATCAATAGATTGTATATTTCGCGTTTTAGGTTAAGAACTGTGAGTATGTGCTAGTTTTTTAGGTGTTGTTTTTGTAATTTTACTCGTTCTTGATTTTGAATACCTAGAGGTTCAACAATTTTTGTTACATTCCTACCAAATTATGCTGAATGATGCTGTTTATGTTATCATTATATCCAAGATTTTAAATATCGGTCGTGGTCCTGTTGCGGTCATGGTCGTGGTCTTGTTGTGGTTCCGTCACGTTTGTTGATATCACGGCAAATCGCAGAGAAATGCAGCTGATGCGGTCCCAATTGCGATCATGGACAGTTAAAAAAGCTTGATGTTGTGGCCCAAATCACGGTTGCCGACCGTTTTTTAAAACCTTGATTATATCCTTTTGATTGAATCTCTTGTCCTTGCTATGTTCCGTCTCTGTGGTATCTATGAACTCTGAGAAGCTGATTGAGCATGAAGAATCACCTTGTGATTTGAAATTGGTTGATGCTGTTGTTTTCTGGATTCTATTGTTAAATACTTATAAACATTGATGTGTATTAGGACTGATTCTTGTGAGGATTTAGATTTTTCCTTCCAATTATGTTATTT

>Gm17ciRNA1555

GTGAGATTTTGTTTTTTTACATTGTTGTTGTTTTCGACCTTTGGGATCCGGAGAGTGAATAAAGCCAGCCAGTGAAGCTTCATTTGAACTATTCTCCTAAAATTTGCATAATAAACAGTAATTTACTCACAAATTTCTCAGTATTTTCTTGTGAACTATTGAATAACAACTGGACCACTCTGGCACAATCTCCCTAACACAGTTATGCAATAGTTATTTCGAAAACAGAAAAAAAAAAACTTAAAATTCATCGTAAGAAGAGTAGAAGACATTATATAATCATTTTGTTAACATAAATATATTGAAGAATAACAATATCAGCCATAAAAAGTTTTATCAATAAATATAGGAGTTCACAAGAAAAGAAAAATATGGACATATATTATGATTCGTAAGAATGATTTCATAATTCCTGATATAATTTTATTACATAGCTCTGGTACCATCATATTAGACTACACTTGATGAAAAAAATCTTTGGAAGTTATGCTTTGAAATAAGACGAGGGGCTTCAAAGTTCAAACTAGATGACTTTTTGTTGATGAAAGATGTGTAGTTCTGTGTCCCCTTAAAATATTGGAGGTGAGCTTGAATCATATGTATAAACTTCTCCTTTTGAAACATTAACAACAACAAATCCTTATCTTGCTAGATGAGATCAGCTACATAGATAACTTGACACTATTTGACTCAGTCAAAAATCAATGTTTTAGCGATGTTATTTACCATGAGATCCTTCTTGACCACTTCCTCTAACGTTCTTCTTGGCCTCCCTCTCCTCCTCTTCACATGACTAAAAACCATACAATTTATTGTTCTCACTAGTGTCTCTCAGGGTGTTCTTTGTACATGTCTAAACCACCATTGATTTTCTGTTATCTTTTTGTCAATTGATTGTACACCAATCTCTCCTTGTATACAAGCATTTTTATTTTGTATTTTTGGCCACATTCACCACCATCAAGTGTAGTTGGACATATAGAAGTACCATGGAGTGTGGTCACAAATAATCCCTGATGCCTTTATCCATTTTAGCCACCCTGCATATATTCTATGTGTGGCATCCTTGTTAATTTCCACTTCATTGTGTAGTATTGATCCCAGATATTTAAACTTAGAAAAATGTGGCAAGACTTCTTTTCCCATTTTTAATTCCATGTCATATTCTTCTTTTCTCCTGCTAAAATTACATGGCGTATACTTTGCACTACTCCTACGTAAGTTGAAACCGTTTGTTTCCAAAGTCTGTCTCCAAAGTTCAACTTGCGAGTAAGCTACTTTCCTTGACTCCTAAATCAAAATTATATCATTTGTGAAAAACATACAATAAGGGATAGACTTTTGTATGTCTTCAGTAAGCATATCTCTAATTACTAAACAAGGACTTAAGGCTGGGGGGTCCACATTAAGAGCAGGACAGTGTGGTTCTTGGTGCACTGCAGCAACCTGTTTCTGCAAATTCTAGTTTGGAAATTGTTATTGTTATTGGTAGCCAAACCTTAATTCTAATCGGTGATGTAAATCATTTAGGGTATGAACAAATAGAACTTTGATTCCAATTTTTTGAGTAGGAACTACAAAGAGCTCCTCATACTTTTGGACTTTTGTGATATATCTTCTATTTTTTTTTAAAAAAAAAAAACTCTCACAATTAGACTAACTGGTTGAAGTCTTGTGTTAATTTGAATTTATTTTTTGGTTGTTTGTTGTACTAACTTCAGCTTTCAATTCTTGTGAGATTTTTGTCATAACTTCTGAGA

>Gm17ciRNA1556

GTATCATCTTCTATTTGATTTTTTGGATATTGCATAGCAAGTTTGATTTAGTGAATCCACAACTTTCTGTCACTGTCTTCCATGGCAATTTAGTACCTCATCTCACTCTCAGCTCTCTTAGTCTATGTTTGGTTTCTCGTTTGGGGAGCTCCAAACTCAGTTTGGAAAGTAAACTCATTTTGGTAACATGTTTGGTTACTCCCAAAAGTATATATGCACATGAAAATTGTCTGAAAACTCAATTTTGTAGAAACAAGACTTGGGTTGCTTTTGTAAACACAGTTTACAAACTTAAGTTTGACCCAAACTTTAATTTGTAAATTTTAATCCAAACATGGACTTAGAAATTTACTGCTTTTATTTTGACTTTATCTACTGATGATAGATATATTCAGTATAACTATTTTTACACTTTTTTT

>Gm17ciRNA1557

CATTAAAAGACAAGCCCCCTTTACCGAATGCTATAGAATACATCTTATACAAGAAAGAACGTACAAACCATCAATCGACGTAAAAAAATATAATTTTAAACAGGATTTACAGCAGTTTATTAAGGTTACAAGTAAAAGGAAACAATAGGGAATAAACGACAAGTACAAATGTACAAAGATAGTAACGAACGAGGAAGATTTTAAATATTATATAAACGAACTTATAATACCAAACCAATACTTCGTCCTCACCAAATGACTGATTACTGTTTACTGATTTTATTATTGGGGAACTATAACATCAAACGAACGTTTATACTAAGAATATTTTATTAAAATACGTCAAAAAAATTTAACGAATAGTATCAACGTTCACAAATGGGGTGATATCAGGGAACGGGGTTTTAAGACAATTATCCCCCATAAAGTCACATACACAGTCACAATGTGATTTTATGTCTGATCACCAGACAAAGCCAATATAATAAGAAGGAAGT

>Gm17ciRNA1558

GTATATTGTTATCTACTCATCTTTTTTCTGGGAAGGGCAGAAGTGGTAATGCTTTAAGCATTTTCATAGACCTATCCAGCTAGAGAAGCAGGCTACATAGCCAAATCTTTTAACTCCTTTGCCCTGTTTACCCCCCACCTCCCCTTCCTCCCAAATAAAACACATTGAGCCTTGCTCATATGATATGCTCCTGTCTCATTCTCAACTCCACCTGAACCTAA

>Gm17ciRNA1559

CACGCTTAGTATACGCTCAATAGACATAAACAAAAAAAAAAGACTTCAGTAAGATAACTTCATCTGTAGTGTTCTAACCTATAATCGTGAACCTTAGAAAAACAAAGTAAAACTTGTCGCATAGTAACTTGAAGATTCTTTTTATTTTATATTTTTTAGTACAAAACAAGAGAGTTTTATTGTACTATGTAATATACGTCCAACGTAGAACCCACGGATTCAGTATAGTTAAACCAATGA

>Gm16ciRNA156

GTAATTATGTTGTGAAATTTTAGCATTGAATTCAACTTGATAACCATAAACAAGAAACAAGCACAATGCTTATGGGGCCACATAATTACTATTTATACGTTATATGTGAATAAAACTTTTCGTGTAATCTAAAGACCCTAGTTTTTTATGTGGAATAAAGAATTTTTCTTTTCCTGGTATGAATTTCAAGTCGAATTATAAATAGTAATTCTGTGGCTCCATAAGCAGCTTGTTGTGGCACTGGGACTTTTTCAATCTTACAGGGAGCATATAGATGTACTAAAATAGTAATATATGCCTCTTTGGGTAAATGATTGCTTTCAATA

>Gm17ciRNA1560

GTAACCATTTCCATCAACATTCAGCTTGTTACTAGTACTATTTTATTTCCTATGGCTTTGAATCTAAATTACCACCAACGTTACCAACAACTGTTGACACAAGTAATAATAACTTGTGTCTCTTAACCAAATGATTCAGGATTTGAATTTTAATTGATCCTTAGGTATAAAATAAATCATATTAGAAAAGAAATAATCCACTTTGTGTGTGCTCAAAATCTTCCGTGAAAATTAATCACCATGGTCAATAGAGACTGCTTTATATTAATAATATGGTCACAAAAAAAATTACCACCAATGGTTATTAA

>Gm17ciRNA1561

CATACTTAACGATCTGATCAATGAAATAGTCTTTATAGTCGTAGAGACGTCAGACGTGAGATCCGTCCTTGCTACTGTACAAACTTCCCTCAAGGAAAAAAAAGGGGAGTTAAAACACAGAGTTAGAAAAAAAAAATGTTATTTACAATTAAACCGATGGGTTATTATTATAAAGAGTGAACCGGTGCTATTGATTCGATATGATCGTCGACATGGGGACTAAAAACTCTTAAAACACTTTTACCTTTACTTTCGATATAAAGAAGGATACTAAGGATGAAAATGGAAGAGACAAAACACAACCTGAATTACTACTGAAAATATCATTCATCTAGAACGTACATCAAAAAGAAATATCTAACTGATCCGAAACATATTTTAAGTCTGACTAA

>Gm17ciRNA1562

CATTTCCTATATCCTTTCACACAACACTAATCGACAAAAGAACTAGTACAAACATACTCAACTAAAAAGTAAGTCAACAAATACTCTAAACGTTCTTCGATACTGTTAACACCATTACCTATGAAGTAGTACTTAAAAAAGGATAGACTTTTTCTACTACAAAACTTCCTAGGTACACTATGGAGTGGAGATCTAAGATCTCACTGTGTTTGGGAGAAGTAGGAGCAAGTTTTAAATTCATTATATTCGAGACCTGATCTCTTAAACGAACGAAGAAAAGCGTATAGTTGGTACTGGTTCGTTATACATCAATACACCGGTACTGACAATTTTAAACTCACAATATTTATTTCCTAACTTATGAACAAACAAGTCAACCTATATTTATACTTTTAGTATATAACTTATTCTTAAGAAGTGGTGTACGTAATCCACTGTCAAAACGGTCATCCAAATTACGAATTACGGATGTTGTAAATTTAGAGACCGACAAAATGATGTGACGTCGACAAGGGTAAATTGTACATGAACAATACAGTATTTACCGTGTACGTTTAAAGAGTACAAGGACGCGACGTAGAACACTATTTCTTTTTTCTTTTGTTGATGGTTCGTAAAGTACTAAAGAT

>Gm17ciRNA1563

CATATACATGTATTAAGAGAGCAGAGGTACAGGAACATAGGTACGATGAATGCAATTCTTTTCTACGATTCTACTACCAACATCAACATGTAATTAACCTTTGTGCTGTTTGATAACGGAAAACTGGATTATTTTTCACTAACAAACATAGGCATAAACAATAACGTACAATCAAACAAAGATAGGGACCTAAAAGGGAAAGATGCAATAACCCTTTAATTAATAAGGTCCTCTGTATTATCACGGTATACACTTACGAAGATGTATATAACACCTTACAAACTACTAAGACTAACGGATATCATGACAAAATACGTGCGGTACTTCCTTTAGAGAGACCTCCGAAGAGTAGATTCATTAGACAGAAACTCGTATTAAGGGTCTCCGAAATTGGTAATAGAACATGAAAGACAAAGATCATAAGAAATAACACTGGTTCTTGGAAGTACTTAAGGAATATCCTCGTTTATAACTTACGATCCACAAAGAAATATATCGTAAAGTAAAGATAGTAAATTCGTACATCCTCACTAAACGATAACCGATGTATATCGGATATGTGTTATGACTAGAAAAAGTCGATTTTTACAATTTCATCGACTTTGAAA

>Gm17ciRNA1564

GTAAGTCTACTCACTTGTTGTCTTCAGTACATCAACAGTTTCCTTTCTAATGTTGTGATTTTGTTCTATTTAATAAGTTCCTGGAATTATGTAATCTGAAAATTTTCAGATAACTCCTTAATGACTTCACCATTCTGGTTTGTGCTAAAATTTTAAAACAAACATTAATATTTTTTGCCCCCATGTCTTGTTTCATGTCATATTATGATTGTGATCACATTTTAACTTACAATGTTTTTACTTACTCACAAAGTCACAATTTAGTCTATTCCTTGAGTAATTTTGTGAAGAAAGAATCCAGAATGTCCATGAAATTCTCCCTGAATAATGTTATGTTCAGGCTTGACCTAGTCTACATCTACATGCTCATTAAAGCAATTAAAAAGTGTAAAATACAATAGTACTTTATGAAGTGAAACTGCTTGTCTCTGGAGCTGGTAAATTACCTATTATTTTTTCTGTTAACCATTCTTGAAATAAAGATCAACCAATCTTCAGATTCCAAGAGGGGTTGGGGGTGACAATGAGTCTAGCATTAATCTTATTTTGGATAACTTGGCTTCTTGCAGGTCTTTGAACTTGCACCTTCTTTCTATATGATCGTTAAATATTTAATAAATTGCTTTTATTAGACTATGAAATATGGATGAATATGCACACTGTTTTTTTTCCTTGTGCAAATAGACACGCGGTCATTATATCTCTTCCTGGTTCCATCTCTGTCATGTTGATTTGGTGTTGGAACTTTTGTTGCTTGTATCCATGGGAAGATGAAGATCTCATTATACAAGACTTGCTTTGATTGTGAGTGAAAGTACTTAGTAGTTTTTTTGTGCACTGA

>Gm17ciRNA1565

GTCTCTGATTTATTTGATTGCTTTCTTCCTTGCTTGCTCTGGCCCCTCTTTTGTATATGTTGAAATTTGTGGAATGTTTATTCAAAGGAAACAAAGTGTTTGAGTGGCTTGACTTGTGGTATTAGGCATTTACCATTTTGTCTGGCCATTTAAAGCTCT

>Gm17ciRNA1566

GTCTCTGATTTATTTGATTGCTTTCTTCCTTGCTTGCTCTGGCCCCTCTTTTGTATATGTTGAAATTTGTGGAATGTTTATTCAAAGGAAACAAAGTGTTTGAGTGGCTTGACTTGTGGTATTAGGCATTTACCATTTTGTCTGGCCATTTAAAGCTCTT

>Gm17ciRNA1567

GTATGTAATTAACTCTTCTTTTACCCAAAAAAACAAAATAAAATAACCCTGTAGTTAAAAGGGACTAGTTGTTAGTGCTGTATATACACTCTTTTTTTTTAAAAAAAACTGACCTTTTAACAGCTACTGTACTCTAAAATTAGTCTCAGTTTAAAATTTTAAATAGTACTGTTTTAGTATATTCTAAAGGACTAGTTTAATTTAATTTAATGTATGAGTGGTTATAAAGACATGGGAAGAAAGAATGACAAAACTTGCATTATTTTCTTTGAATACTTTTTGTTTGTATTTCAATCAAAATCAGAGTTTTGCCTGAGTAATTTACTTAAAAAAAGGTTTGCATTAAAGGCCATTAGACAACATGGTATGCTATGATCGGGGGATTCTCCAACGTCTGGCCAGAGATAGAATCCGTAGATCATGTGTATGAAAGAGCATGTTGAGATGAGAAAGATCAATGTAAAATTAAAATTTTACCTTCAATAAAAACTTTTATCATGCAAATTAGGATCGTGAAACTCTAAACAACACCAAGTACATTTAAGTTTTATACTAAAATAAATCAAAGATGGGGTTGGTTTGTAACTTTGTATGTTTTGAT

>Gm17ciRNA1568

GTATGTAATTAACTCTTCTTTTACCCAAAAAAACAAAATAAAATAACCCTGTAGTTAAAAGGGACTAGTTGTTAGTGCTGTATATACACTCTTTTTTTTTAAAAAAAACTGACCTTTTAACAGCTACTGTACTCTAAAATTAGTCTCAGTTTAAAATTTTAAATAGTACTGTTTTAGTATATTCTAAAGGACTAGTTTAATTTAATTTAATGTATGAGTGGTTATAAAGACATGGGAAGAAAGAATGACAAAACTTGCATTATTTTCTTTGAATACTTTTTGTTTGTATTTCAATCAAAATCAGAGTTTTGCCTGAGTAATTTACTTAAAAAAAGGTTTGCATTAAAGGCCATTAGACAACATGGTATGCTATGATCGGGGGATTCTCCAACGTCTGGCCAGAGATAGAATCCGTAGATCATGTGTATGAAAGAGCATGTTGAGATGAGAAAGATCAATGTAAAATTAAAATTTTACCTTCAATAAAAACTTTTATCATGCAAATTAGGATCGTGAAACTCTAAACAACACCAAGTACATTTAAGTTTTATACTAAAATAAATCAAAGATGGGGTTGGTTTGTAACTTTGTATGTTTTGATGAC

>Gm17ciRNA1569

GTATGCCTTTTTATTTTCTTTTTAAATATGAATTTGACATCTTGCGTTGATGAAATGATAAAGAATTAAATGAGAAGTATATGTCTTATGCAAAAATACTTTTTTTTTATATGAAACTAATGGATTATTACTTTAATACTCGTGTTTTTTTTATGTTGTATTTATTAAATGTTTAATTCTGGGGCTTCATATATATCATGTCGGTTGGTTTCAGAATTGTGATATTAACCATTCCCGACTTAAAATAAGAGCTATGAATTCTAAAAATATTGTTTTTCATTTTATTTATTTTTGAAGTATTTTCTCA

>Gm16ciRNA157

GTATCCATTTTTCACTTTGTGCATGTGTGAGTTTATTATTCACATTTTTGCGAGGAAAATATTCATCTTATATGAGCTCACGGTGCTGAGGAACATTAGTCACTGCTTCTGGCCGGGCTACTGGTACCGATACCATAATCAGAAAAAAAAAAGAAAAAAAAGCTTTCACTAATCACTGGGAGAGGAGGAATTTGA

>Gm17ciRNA1570

GTAAGTTTTTTCTTTATCGATAAATTTTAGTTTTGTTAGCAGAAAATCGAACCAACCATCTTTTCCTTTCTTTCCTTCTCTCTTAACCATCCAACTCACCTTATATCTCTCCACTCAGAAAGTTGAATATTCTAAATCTCAAGTTGTTTGTTCTTTCTAAAACTCACTTCTTACTTCCAACACCAAATCAAATTCTCTCAGATAACTCTTGCTAATGAAAATCTCACCATACATTGGATATAAATACAATGGCTCAATTTATCTCTACAAACATTGATTAATCTTATGGATTGGGATGAACTGACTTCCCAGATACAGACTTTTGCTTACTTGCTATAGATA

>Gm17ciRNA1571

CATTTAAATACAAAGATACTACGTTAGTACAACTTCATCATATTCCGATAAACAGTCTAACTACCTGTGTATCCACTATCCAGTTATCACTTCATCGATTTCATCGGGAACTAGTACAGAACACATGACAGTTAGTGGTACGAGAGTAACTTTCGTAACCCGTGTGATTCCTACACGATACCACTTCCTCAAAACTCCCTTTCTCTTCCCCTCCCATATCAAAAGTAAAGTTCATTTATAAGAGGACAAACTCTTGAAAAAATTCACTCTCTAATATGAACTTACATTGATCTCTACTAACTTACCTTTAGGAGTTCACACAAGAGGTGTGAATCCGAGATAATATAATCTTTTGAATCGAATGAGTCATTCGACTTCTCTCCATTCATGTTTTATGTCTCGAGACTTTTCTCGTCCATATGTTTTCTGTTTTTTATTTTACATTCGATGATGAGATCCATGTAGTACTGGATAAAAGGTTGATAATAGTACATTGGAAAGTTAGTAGGGAAACCTTGTGTATATAGGGAGGGATAAGTTTTTACTTTTAAATTAACAGCGAGGTAAAGGGAAGTTTTTGTTGGAGAGGTAAATTTGGTACGGGAGTTACTTTTGAATTTAATACAACAGTAAATTTTTTTTAAGGTCACAACTACAAAATAAGTCACTAACATAACAATATAGTTGTGACAGAAGATAGATCTATCGGGAAGGATTACGTACATGTAAATATACCACTGTTGTGGGTAAAACTGTAATTAAAGGTATAACGGTACGACACACATTAAAATATTTAGTTTTCTAAAAAAAAACCACGTTCATGGGACAATACATAGTTAAGACTGACTATAACCCCTCTAATCGAAGTCTAATTAAAGGAAAGACGTGTAGTAACAGAAGTACACAACACACTAATCAAAGA

>Gm17ciRNA1572

CACTCATTTAACCAAATCTTTACAGATAAATCAAAAATTGGGCTAACAATTAGGTACGTAGGTAAGAAAATTACCGGTCTTTATAGATCCCAGATCTGGATCTATACTTTTTATTCTTGGTAAAATACCTTCTCTAAATTCACGTCGTCATGTGATTTGCAATGACAAGACGATCTGACGAGGTATACAATCATGTACCTTTCCTGTACGCGTGAATGTATAACCGTAATTCTAAATACTGGAAAAACAACAAATTTTGACAATGTTCTCTTTATGAG

>Gm17ciRNA1573

GTATACATCATATTCCTCTTATTACTCCTTTTCTAGTTTGCCGTTGTATGTCTTCAAAACATCACTATGCATAAGCAACATCTTAAAGAAATTTTTTTTGAAGGTAAAGTCAGTAAGAGTTCAATAATACTATCGTTGCTTCCTCCCTAACAATCTCCCTCCTTTCCCATGCTTCTGGTGGATGCTAATAAATAAGTTTTTTTTTTGTTCATGGGCTATTGACTAGTGTTTATTAATAATCCAAAATATGTCCAGTGTCTAAACGAATTGATTCCTTGTATTAAGAAATTCTAAATGACTTCCATTCTCTGTAATGCCCAAAATTGCAAGTTTGTGCTTGCTACCTCATTAATAAATGGCCTATGGTGACAAGACTAGTTTGTAGCAGAAATTGTCAAGTTTCTTTTTTTCCAAGTCAAAGTTTGCTT

>Gm17ciRNA1574

CATGTGCGTAGACGGAGATTTAGAGACTCGAAAAAAAAGAAATTACTTGTTTACAATTAACAATCAAAAACAATCACCTCCTAAGTTTGAGTGCAGGATAGAGAAAAGAAGAGGGAAGTGGCGATCTGGTTGGAATATAGAGCGACTTAGAAACTCGAAAAGTAAGTAAAGAAATTTTAGAGTTGTATCCTTAGGCAAAAAAACCTAGTTCCGAAACTTAGAACTTTGTGTTGAACAGAAAGTTCAATTTAATGGAGGGTTGTGACGCAGTATTGTATACACAGAGTAAGGTAGTTACAATCATTAATTAATTATGAGGTATCGAAGTGCAAGTGGTGAAAACAGTAGTTATTTATTTAGGTGTATTGAGTACGTTTCTACAATGATGATAACAACATGAAAGTGTTAACGATATGAAACTACAGA

>Gm17ciRNA1575

GTAACTTAATTGTTCTCTGAACAAAAAGACTTGGTTCCGTAACTTACTTGCCCCCATTCCCCCCTACTAAAAGAAATTATAGACATATCTGTTTGATCTTTCCTTTTTTCTTAAAAAAAAAAAGGTACAAGACACGGATTTAATGGCAAAATATAGAAATACT

>Gm17ciRNA1576

CATTCAAACGATTAACAGAATTAACCGACGACAATGAAGCCTGAGAGAAAAAATCCCACAAAACTATAAAAAGACTACGAAACACCTATACGTAAGGTACACCAACCTAACCTACCGGTAACTTTAATACTCCCAACAACTGTACCATAACCTCAAGCCTCGGATAGGATTTACCAAGGGACACAGACAGTGCGAAATCCGTCACACTCCCTCTACAACCTATTTGATGGATTTAACACCAGTGGGGATGTATCTCCCCCACACAACTTTCAGGGTGTACCGCCCATCGGTACCTGTTTGCCTTGTACAACTTCAGGGTGTAGCTGATTTCCATCACAACTCTATTTGATATATTTACCCCTCATTAAGAGTGGAACGTTCTGGAACGTTCGGCCAAAACATCCTAATTCAATCCACGTTTGGGTACAAGACGGTTTACAGTTTATCACTTTAGATAAAATCCTTGTTAGACTCACTATAAAAGATAAATAGCTTTTACTTCAATACAGCATACACGAAAGAAGAACCCTCAAAAAAGTTAAATGTTCAGTACACTCACCACGATGTTCGTAACATTCACGACTTTTTCCCTATATTTTACTACTACTAAAAACGTTCGTACCTGAAATTTTATGTAAACGACCAAATTAACACGTAAACAAAGACAGGCAATATACCAACTAAGAAAATGGAACTTTCAATGGACACGACTTTTTAATAGAACCCGAAACTCCCTATCTTTATACACCGAACATCTTACTAACATTAAAAAGAACCTAACGACACCGTCAGTTAGATCAATGTACGACTCTGGATTCAAGACACTAAAGGA

>Gm17ciRNA1577

GTAACAATTTCGTCTTCCTTCCCACGATTTCACTTCTTCTTCACACACAACAGCACCTTAACCTAATTTTGGAGATATAATGTTGGTTTAGTGATGAAAGGAGAAGGAAAGGGGGAGAGGTCGCGGGTTCCAATCCCCTGCTAACAAACAAAAAACTAACAGTATTAACAACTAACATTTGCCGATAAAAAATAAACGCCTTAACCTAA

>Gm16ciRNA1578

CATACATCGAACGGTCGGGGCGTGGGGATTAAAAACCTGACAAATAGTACACATGGATAATTTCTCACACCTACACCAGCAACGTATAAAGATGTCTAACTTATACGACTCATTCGTATGACGCAAACCTTATACGTTGTTAAAAAAGTTTCATTTTGTTTAAAATCCTGAACGCACAATACGGTTCGTACGAGCTATCTAAAAAAAACATCTCAACATTAGCCTTTCCCTAATCAAAAGAAAAATACTATAGTTAGAACATACGATACATATTAATACATAGAGAAAAAGTATAACGATCAACTGGATATCCATGAATTGAAAAGGAATGACTGTCTTATCTCGAAGTGAATCCAAATTAAAAAACCTCATTTAATGTGATTGGTAGGGATTCCAAAACACTTTAATATGTCTGAAGGGTAAAAAAGATGAGGATGTGATTGGGAGGAATTACGACACAAACCAACTACCCACTCCCTCTCTTTATCTCTTCTTTAACACACCTAGGGTGTGGAGGATGTGAGATGAAACTAATTTTTCAAAGAAAAGATAAAGGTAGGAGAGTTGGTTTGTCGTATTAACAAACTTTTTGTAACGTGGTACAAAGGGAAGTTCCCTCCAACGACATTTTCAATTTTTTCGTCCCACTCAAACACATTAAAGTACTTTGGAGTTCCCACCAATTACATTAAATGAGTACAAAAAACCCCACACATCAAGTGAAATGGTAATCGAAAAGTCAACCAGACAATTAAAAAGAAAGTCAACTTACTTACAATGAGTAGTCATCGAAAAAGTCTTTTCCAAGAGACTACATTTAGGACTGTAGTAAATATTGAAACACCCAAGCGTTCACTTACGGGACGACAATACAATAACTTAACATATTATACTCTACCCTACTACTCTAATCTAAACTACAAGTATTACAGTCGCTCAAGGA

>Gm16ciRNA1579

GTGGTTACTTTACTTTGCAACCGGCATGGTTAATTTGCAAGTTATTGGAGTTCAATTTGTTATATATTGTTTTTGCTAGTCTAGAAGGCATTGTTTTAGAAGTCATTATTTCCTTGTCACCATTGCTATCATTGCTTGCCACCACTTGCATGCCTGTTTCCACCACTATTTTCATTATCACAGTTCACACCGCCTCACTTTCGTGACTAGTTGCTCCCACTGTCGCTCATAGTCATATCACTAAATGAGCTGGAATTAGGAACTAATGAGTGGTTTGTATATTGTAAAAATAAATGTGGTTTGGACTTGTGATTTAGTATTAGAATTTGAAATAGGAAAATCACAAATTTGAAATTAAAAGTTATAAAACTAAAAAATGGTTTGTTTTTATAAATTTCAAAATTTAAAACTGGAAACCATATCGAACAAGGCATAAAATGATAGCTTGAGGGGTTTTTTCCCAATAGTCTGTCATTTTCATCGCTCCTTTCTTAATAATTCTTTAAAAAAAATGAAAAGTTAAGGACAAACATTTTTGGAAACTTTATCTTTTTTTCCTTTTAATTACAAAAAATTCCACCTTGCTTTCAAATGTTTGTATAAAACATCTTAAATATTTTTGAAGACAGGATTGTGAAAATTGGAAATAAAGTGAAAACATGTAGTATGAAAATAAAAAACATTTTCTAGATCCAAACAGGCAAATGCAATAATGAGGTCATGAATTTAAACTAATCACACCAAAGTGGGTAGAGGAGGAAACTTCAGTTGATAATTATGTCTGGATTTTTTTCAAATGCTCCAAAATGCTGCGTAGACAACATAATTTGTTATCAGGCTGTTGTATTTCTTCTTTTACCTTTAAAATTATCATTGAGATCTGGTTGATACATGATGAAGGCTGAATCCTTTGCCATCTTTTATGGCCGCCCAGTTAACCAAAGATCATAAGATATGAATTTTATGGGGAAAGTGGTGTGCACATGTTGGCAGAGTATTAAATGAGTTGATTTTGTGTCTATTAGCCTGTCCATTCCTCTGTTGTCACTA

>Gm16ciRNA158

GTATGTGCTGTTTCTAGCTTTCTCCTCTTGTATGGTGCCAATTCTTTCACTATGACAACTCAAATAGGGAAGAACAGGAGAATAAATTAATAGAATTTAAGGATTCTGGTGAATTTGACATCTTCTGTACAATGCAAATTATTTTTTTAATACATTTGTTAGGTTGATTCATAAAAGAAAGAATTACAAAGGGTCAAACTTTACCTAGGGGCACCTGGAATTCCTGCCCTTTTCCCTGTGCAATAGTGCCATAATTGCTTTATTTTCCATTTGTATCCATAGCTGAAATTTGATTCAGATTCTCT

>Gm16ciRNA1580

CAATATATATAGTCTGTAACCGATGAGGGAAATGACAAGTTAAAAACGTTCATGAAACAGAACTAACACTGAAGACAAAACAAATTTGTAAACAACAATCTATGGGTAGAGGGCGACCTAAACGTGACCGACAACTGATTAATGATAAAGACTCATATGAACCACT

>Gm16ciRNA1581

CATTGGTTTTATAGAAAAAAAAAACAATGACAAATGTTAAACTGAAAATTTACCTTCTTGGGATATAATTGAATAATCCGGTTATCATGCCAGCCACAGGAAACATATGACAAATTCTTCTAAAATAATTTTGCACAATAATTACACTAAGAATTATTATCGAAAGATGAGATATACTTAAGAAATAATCCTATGACCAATAGTTCTGAAGTTGATTCAAATGTTTTAACTAAAACTAATTTTAAATAAAAGTTCACTGTACCAAATACACACCTACGAGAATGTAACTTTCGTTTACACGATACTTTGGATTACATTTGAAAAGTTGGGTTGAATTTTGAAGAAAAATAATAAAGATCAGTTTGACTTCAAAACACCGTCTTAGTTAAAAATGATTGTTATTGGTTCGTAGTTCAAACTATCCTCCGTGCAAACCTGTACTTTTTAATGGAGTTATCACTTCGATAAGGGAAATATCGATGGTGTAGTGCACTTTAATATGTAAAGTTTGTGAGATTTTTCCAGAATCTAACAATCAATGGTTACACCGGAAAATGAAAAACCTTTCACCGGTTTGTAAAAGGAATAGTACGATACCACAAACGAAATAAACCACATACGTTTAACGTTTGTTTAGATT

>Gm16ciRNA1582

CATTATCTGGACCCAGGACGTAAGGAATGAAATAGATAGAACAATGGTATGTATAATGACATTATGTATGAAGAAACTGAACCTTTTTTACAAGTATTCGATTAGTGTTTAACTAGAAGGAGTTACCAGAAAGAAAATGGGATACAAAACATTACCAACTTGAGAAAATCGGTGTAGATAAACACACAAAATCGAAAATCCTGTTCACAAAAACAGAGGTTGAAGAAGTTAAAAATACGACCACCATAAAACACTTGAAATATATGAATG

>Gm16ciRNA1583

CACCTACAATGAACTTTAAGTTTGACGTTCATCATAATCATGACTACTATAACTATAAAAGACACTAAATATAGAGACGTACATACACTTGAGTCAACAAAAATCAAACGACTTGTAAATTTTAAAATACTAAAGTAAGATCTACACATAAAGATAGATCGTTCCAAAGTTTTTGACTTAGTCAAGTTCCTAAGTTCCAGGTTGGTCCGAACAAATCTACGTATGGCTATTTATATACTGTTATATATTAGTTATATTACATACAATTCATATGTTCATCCTTATTAACTTTATATGGACATCGTGACCAAGTGACCAAAGATTCGAAGACGAAAATCTCCCAACTGGTCTGATCAGTTTTGTGGTTAAGGACTAGAGGGAACGATTAGACCAACCAAAATTTTTGTTCGTTCCGATATATCTAATATTTGTGGTTGAGGACAAGAGTTACAATAAAATCCCTGGTTCAAACTTAGTAAAAATTGTATCAACTGACCCTACCTAAAGTAAAGTATATTAGTAGCCTATTACGTAATCTCACTACGATAGTTACAAATAAAAAGTTCAAATAGAGAGGTAGAAACATAAGGATTGATT

>Gm16ciRNA1584

CAACCAACTAAAATATTACTTTCGTACAGAGATTGTCAGTTGCACGGTCAAAACGGTATTAATACTACGTAAGTTTTACGGGAAAATTTAAGTTAAAAGCAACATTCTTTATCTTAATGTTTAGATCCAACGTAACGTTGAAGGAAACGTTTACAATGTACCAAAACTCACAAAAAGAAGTTAAACCTTCAAAATGACGATGA

>Gm16ciRNA1585

GTAAGTGACCTCTAAATTCTGTATGCATGCTATTGTTAGTATTGATGGTTGGTCTGATTTAATATATTAGTTGATTATCTCTTTTCCCCCTGATCCAGTGATCCCTAACATTTTTACATGTGTATAATGGATGAGGCTATTAACAATTGCTCATTCACTTTTTCACTAACTTCAATTGCTTGGCAATTTACAGTTATATGATTTTTTATGGTCTTTATTTCTGAATAATGATGTATCCCAGTCCTTACCTATTTGTTAGCAATTTCTCTGAGGTATTTTCGCGTACTTGGCACTAGTTTTATGTAATAGTTGATG

>Gm16ciRNA1586

CAAATGTAGAAAATTGTCTATAAAAAAGAATGTAATTTATGTATGCCTACATACCTTAGTTTGGTACTGTTGTATCATTGTATGATCCAAATAACCAGAGGGTGAGAGATACAAAAAAAAAACCCCCTAAAAACTTAATCATTATTTAAAAACAAAAAAAGAATTTATATCCATCTAGACTCAGCACCCCAAAACAAAAACCTACTTAATTTCTACAACCATCAAACGTCTAACCACATTACTACTTCGTAAAACTGGACCCCCCAAAGTGAACACGCGTTTTCCAAAAGTAGTAATGAAGGAAAAGCAAAGAAAGAAAAAGAGA

>Gm16ciRNA1587

GTGCCCAAAATACTTACATTCATTTATCCCTATAGGCTTTCTGCTATACTCTACTTTTAAGGCTTGCTTGGCACATGTTCCTATACCATGTATAAATTGCTCTCCTTGATTTTCACTGTGAAACAAGTGATTACTACCAATCTTTTTTAAAATACTAGATTTTTGTTGAATTCTTTTATAAATAAGATACAAAACCTTAGTGTCCAAATGTGCAGAAGATAAACATGTTACAGGTATAAAGCATTTAGTTCCTGATGACAAAAAGTGGAAGATTTTATGGACCTGATTGTGGTAGCATATGTCTAGATGACATTATTTGAATTTGGTTCTTGCTAGATTCGTGCTTATAAGGACTAGGGAGTCTACACTCATGAATATGGATTCTGATAGCAGTTAGATTGGTCTTGTTTAGCTTATGTGATCTACTGAGAACAGTTACTTGTGTTTCTTATTATCATGATATGTATATTTTTCTTTCACCACCCCAAGAAGGAGCAAGGCTGAAATTGGGCTGGCCTTATCTAATTAATATTTTTCCTTTCTCAGCCTAGAGAAGGGCTGCCGCTGAAATTGGGCCTGTCTGATTACGTTTGGTGTGGTTGGCCTGATCTATTTTTGTACCTCCGAGATGGAGGTGCCCAAAATACTTGCATAATTCAATTTATCCATCGTGACATTTTGTTATATTCCGCTTTTACTAGTGACTTGCTTCATATGTGATCCTAACTCTA

>Gm16ciRNA1588

CACTCATTGTACTAAAAACAACGGTTGAGGTGAAAGTTAAAGAACAAAGTAACTTGGTGTTTAATTCAAATTAAACGTCCGTGATAGTTATATTTTTATAAAATGTGACAGTTCACCATTTTTTATTAACGTCTATACCTAAAATTCCATCAATAACATTTTTTATTATTTAAATAGTATGTACAATTAAATACTAGTACCCTCTTACATTAAAAAAAATTACCAGTATCCCCTTTGATAAAAGAGGATAAAT

>Gm16ciRNA1589

CATTTCAACTAAAAACTGAACTTGATCTTATGATCTAAGAACTTTATCCCATTACATACATATAAAAAACAGTAAACTAAAGGCCGTTTAACTAAACATGGACAACGACCGTACCGAAAGAAAAATCGATGAATACACGAGCAATAGATGACACTGATATATAAAACAAACTGAAAATCTTGGCACCAAACCATTGAATCAATATCATAAATGACTAACCTCTTACAAGGACT

>Gm16ciRNA159

GTAGTTTATCTCATCAATTTTATGTATTCACAATGGTCAATTGCTGCTGAAGTCTAGAAGAGCTTTTCCACTATGATTGAAGGTTTTTGAATTAGCATTGATTATGTGACTCACGATAAAGATGGGGTCATGAGTCACCATAAATCTTAACAAGGGGAGAGTTTTGAAAATATGATTAAATGGTATTTTACTAGTCTACTAGTATAATCACATGGCAGCTACAAAGATCAGATAATGCTGTTGGGTTAGACATGCCCAAATTGAAGAACCTTTTGCCTGGAATTTTCAAAAACCAAAAGTATTTGTAGTTCTCCCGAAAATTGATCATTATGTTTGAAAATAATTTGTAATTGAGTTATACATGCCTCCATCTAGCAGTGCCTCTGCTGTCTAGCTATAAAAATTCTGTACACACTACTAAAATTAGATGATAAGTATTCTTTCTATCTATTTA

>Gm16ciRNA1590

CATACTGAGGATAAGTGACAACGTAGACCTACTTGTTTTCTCAACACAACCTAATGTCAATATGAAAACCACAGTACTGACACGTTACAACAGTTGATCTAGTAACTCATACCATGATACCAGAACTATTCAACTATAATAGGATAATGAATACGTGTCAATTACTTTAATGGGTTCAAAATTGAAAGACTAATCAAACTGTTTACCCTATGAACTTACCCGAAAAATGGACCAAAAGTCTGAATTATGTCAGTTCGAAAACCACAAGAAACATACGTTAATAGTTCTTTTACAGTATGAACGTACGTCTTACACTATAAATTCATTGAAGTCGATAATTAAAATCCTTAATTTCAACAGTTGTATTATACACAAAACT

>Gm16ciRNA1591

CATGCACTTCAAAGCCACGAAACAACTAAGTTTGCGACTTTCTAAACTAAAACACAAAACCCAAGTACCTTCAAACTTCAAAATACTAATTCCACTTTTGTACCATCTTTATATATCTTTGTTGCTTTTGGCTTAGTACCACATTAAAAAAAAAAATTAAATTTATAACCTACCAAGATATACAATCAATTACCTTCTTTCCCTAATCTCAACCCTCACGGGACCTAGAGCACAACATACACAATGTTATAAACTTAAACGTACGAAAGACACAAAACTAAGAAACAAAAAATGCGAATAAACGATTTAAAAAAATATCTTTTAAATCAAAGGGTATACTATATCGACTTGTTTTTACTACTTCTCATATCTTTCCCCATAAAACCCAGTCCCCATCTCGGACATTTAACATCTGATGCCTCCAAGTCCATCGTTTATATTCTCAGAGTTATCAAGAGAGAGAGTGGTACTCTCACCTTTTCTTAAAAAATAAACTTCCCGAAAATACAGTAATACAAGAGCCTAACAGGACAACCCAAACTAAAACAACATAATAGCAACAACCAAACACAAGTAGAGAAAGATAGGTAACACCCCATCGTTTTTCTGTATCAACTGGGAGAAGATAAGACAGTAACTGACTGATAAGCAAAACAAGCATACTACGGTAGAGATATTAATATTACAGTTTGACGTCTCCAATTAAAACATTATTATACAACAGAATTACGATTGATCAGTAATAATTACCTCATTTGGTTTAAAGAGTGACAACAATATGACATAACGACATAAACTAAGGACGTAGGTTAAAATTACAATACGAATACTTAATTATTAAAACTTTGCAACCTTAACGAGGAGGAAAGACGAAAATTACCCCAATATCAAGTGTCAAATTCTCACTTTCTAATAAAAAAATAACCGTTTACAACCTCATCGCGGATAACATCTCTTCTACCACCTTTTATCTGAATCCACCAAACCCGTACATCTCTCTTCTGGCCATCTGAGACATCACTCCTCTCATCTGGTCTACCTCTCTTCTGTTTGTTAAGCTCCGTCTCCTTCTGGGTTTTTCTGATATTCTCTCCAATAGTTTTTCCTAGAGCTTTAATTACCAAACCTATCTTCATACCATGAACTATCTTGTAATACCGCCTTCAACTAGGTACATCGGCTGGGGTGGATCACTCTATTCCGCAACAACAACAACAACAACTCTCTGGTTTGGGCGTTGGAAAAGGGGGAAGGAAAGAAAGAATTGCTGGGTCGGTTGGAATATAGAGGTTCTCACTTTCTAATACAACATCCCAAACTTAACATATTTGTGACTAAATTCTATAAAAACCAGAAACGTTGTAAAACGAAA

>Gm16ciRNA1592

GTAAGATTCTTTCTTTCTCTAAACACCATATATGGATCACCTGAATGTGCTCATTACTACTTTATGCTAGTTAGGTTACCAGTGCATTACACAGGCGTTTTAAATTTTTAATAAACAATATTTAGTACATACTTCAATTAGTAGCATTAAAAAAACTTATAATCAAAATATTTAAAAGAGAAACATTAGTTAAAATTAATTTATTGCAAAATAAATCAATGTAGACATAATAAATCTTAATCAAGTATAACCTTTCGAATTATTTGTGTTAAATTTATATACACATAAGTTATTATATTTATTTTAACATGCAATAATTTTTTTAAAGATTTAGAAAGCCTTTAGAATTGTTACTCTCTATCTTTTCCAATATAGTTTTGACTCAGTCCTAATTTGATAAATTTCCTGTTAAATTTTCAAACAAAACTATTTTGGCAATAAACACCTTTCTTCTTTTAGTTTGTTTTTAAGTTTTCAAATTTGTAGTTTCATTAATATTTATTCCTAATTAGTCATCTAAGTTGTTAGATTAGTAAAATATTTTCATGACGGCTAACTCTATTAGTGTCATCAACTTGTGTGGCAAAATGTCGTTGGTACAATGTAGGAACTAGGTAGAATTAATCCCTTAGTGGTGTTGCAGAAGTAGCTTTTAGGAAAACAGTTAATTTGATAACTTGGAGAATTTTTGGAATGCAAAAAAGTTAAAAGAAAAATTTCATTACTCAAAATCTTTATTCATTTGATCTTATCTCACATTGTTCCCGAAATTTTTGCTCAAGTATGTTGGTCTAATAAGGATAAGAAATCAACTTCTTGGCATTCCTGATTTAGTAAGCTGTAGATTGA

>Gm16ciRNA1593

GTACGAGACTCTCTCACCGGCTTTCTCTCCCTCTCTCTCTCTCTCTCTCACTTCCTCCTTCCTTTGTTTTCTTAAGTTTCACGATCCGTTCCACAATTCTCATTTTCCCTTACTCTTCTTTCTTCTTTCAACACTTCAATTTCACTCCGTAATTTTCCTCAGATCTACACTTAAAACGTAATGCTCAACTTCTGTGAATGATTGTGACGGTTCTAGATCTGTTGCTATTTGTCGTTGTAATATGTAGATCTGCTCTGGATCTGAATCGATTTTGCTCTTATTTCGCTCTTTTTCTATAACACTATTCAAATCTAGTATTTCTTGATATGAGAATCATTCTTCTACTAAATCGTGTGAACTTATCTTAGTAGCTAAAACTGCTATGTAGTCATTAGTTTATACTCTCTGTGTGTGTGTGTGTTTTCACGTGCTTCTGCTCAACGGATTGGAAATGACAAGTGTGACCTTTCATGCAACATAGGGAATACGAATCTCGCTATGTAGTCTTTCTTGATATATGTGTTGTTGTTAATTTGTCATTATCTCTGAATATTGTTGCACGTGTTATTATGGAAATGAGAAATGTTAGCATCTTACTCTCTGTTCTCTGATGTTGTTTGAAATTTATTGGAATCTTATTTAATCAATGAGTTTCACTTATGATTTTTGTATTTTTCAATGAATTTTAAACTAATGGTGGAGAGTGGAGAGTGAAGAGTGTTTTAGTAGGAGTTAGCTAGAGAGTGAGCTGCTTGCACTCCTGGATATGAGAGATTATTGTAATGATTATTGAATATTGTTGCATGTGTTAATGCGGATATTATTGCCATGATCTATGAATTTTGTTGCATGTGTTATTCGTTGTGAACT

>Gm16ciRNA1594

GTAACATGCTGAAATTCATCCTCCTCTTTTTCTGTTTTATTTTTTTATTTGTTAACGACTAATATGTGTAGTCTGATTGATCATATTTAATAAAATTCTCTCCTTTTAATCAGTTTTTTTTTTTTTTTTTTCATTTCGAATTTGTGACCATAGAGACGAGTTCATTGTCACATTTAGTGACCATACCTAAGAAGGGCTTGTTGGTCCCATATGGAGTATTTGTTGAAAGATTTCTTTATGTATACGTTAATAGTAATGATCCAGTTTCTATACCTGTGCCACAAATTCCTCTTTCCATGCTTTCACAATAAATGTCTTTTCAGTG

>Gm16ciRNA1595

CATAAATAAAAGAATTTTAGTAACCTTTTATTGTTTCTTTCATTTTTCTAGACAAACACCACGTAGTTTGTTTGTGAACAATTGTAGTAAGACAGAAGACTAAAAGGTCAGAAACTAAGTCCTGTCATAAGGACATCCTTTATATAAAGAACGGACTCATTAGTATAAGTTTACCTTTGTTAAGGTCACTTTATATCATCTTACGGGGGATAAAATAAAAACTTATAAGATTCTATGTTACATCGTAACGGCGGTGTTTCCTTTGATGGATCATCCGAATTTAATTTTCTCTATGTCTATGAAAAAGAGTCAACAACGAGTTGTACGAGAACCCTAAAGAACTAAAACAATTGAGTACAAGATAACAAACCAATAATTAACAAATATACACGTATAACAATAACTAATAGGTACACCTTACGGAAAT

>Gm16ciRNA1596

GTGATTTTCCTAATTTAACTACAAAATTCTTTTGTTCTCAACTCAGGCAAATAAGGGACACCTCAAAATATTTGATTATTTTAACAAACTACATGATGGCACAGTACCATCATCATTATAGCTTACTTTGAAAGCAAAAATAAAATTGAACCACCCCTTTTTCTTTGTTAGCTTAGTAATTGTCTCCTGATATAAGCAATATTCTAAAGTTGAATTATATAATAAGGAAAATGTCTAAAGCTAATTTGTTCTTTTATAATTTTATT

>Gm16ciRNA1597

CATACGGGTTTAACGTGTACTGAAGAGAACCTAACGTAATAACGGAAAGGGTTTGTATTGACGTTAAAGTTATTAATACTAACGATAGAAGCAATTAAAAGTTATTCATTGCTATTAATACTGACGATAGAAGAAATTAAAAGTTTTTCATACGACCCCAACCCATATTATCAATATGATAAATAACGTGAATAACAGAAAAAACAAAGACAATTACACAACCCAAACCGACAAGTCCTTTAAACTCAAACACTACCTCTTACAAAATTAAGTATTAAAATCTTATTGCAAATAATACAAGGTGTGAATAAGGTCTGACGTAACATCAACAACAAAAATCAATTATATCTATCTAAACCGTCATGGAAT

>Gm16ciRNA1598

CAAAGTCTCATAAATGTAACTAGCCACAACTTCAAAACTTAAACTAGCGTCGAAGTACTTACGAAAGACCCCAAGCTACTAAAACTGAACAATTGTATATACTTCTTTTTTTAACAAAAAAAGAAAGACCCAAGGTCAGAAACATGCTCTAAGACATTAAGAAAAAAAAAATTAGAAAATCACT

>Gm16ciRNA1599

CATCCAAGTTAAAGGGAGATCCAACAAATTCATAACCAATATCAGGTAAGAGGTAGTACCTTACAAATTTGTATGGTTAATCTTACTGTGTTGTTTCCCTTCTTAGTTTATAATAAGGTATTAGACATATACTTTACCACTAGTACTAAGGTAAGTTTGTAAAAGCGATACGGTATTTAAAACGAAGCAAAATACGAAGGGAAATATTCAAAAGATGTATATATTTTTAAATTGGCAGATCCCGACTATATTGTGGATTGAAGACGAGAAACCTACGTCACTTTGAAAAGATATTACGTTCCGACAATCATAATTGTAAATAGTTTTGTTTGGAAATCACCGACGTTTGAAGTAACGTTACCCTCCTATTTCTAATTTAAAGATGAATTTGTAGTTTTTAGTTCATACTTTAGACGAAGGTACAGTATTTCGTGATCGACATCCGTATTGATTAACTTTTAGTTTAATTACCGAGTAAATGGACATGCAGTAAAAGAAGACAAATGACCCTTTTTTTTTTGACTAACTTTTACCATTACTAAGAAAGTACATTGTTAACCCTATTACGACTCTACTACAAGAAAGAACGTTATACCAGATATACCACAACTCAAATTATTTAAAAGAAAACGATT

>Gm06ciRNA16

GTATGTATATTTATACTTTCTTCAGTTAATGAATATATATGGCATATGTTCTTGTTTATGATATTCTTAGAATAGAAAAACGTTTTAGTGGCAATAGTTTTCCTACAATTTTAAACATTGGGATGGAATTTGGAAATAGTGAAATAAGCTTATTATGTAAAGTTGAAATGCATGTTAACCTTTTATTCTTTGAGCATAAGGCGGCAATGGTGTCTCAGTTAACTTGCTAGTGTTTTTCTTGCTTTGGCTTACTATTGTTTGATCATGCTCTTTAGAACACTAAGTTTGTATCCATTGTTCAATTTCCACCTCTTAATTGTTATTTCTAGTCACTTTCATTGCCATTTGAAAATTTATTATCCACAATTTCAAATATCTGTGCTGTTTTTGGCTTGTTTTTCTTTACAGATATATTGCAATATTACAACATTTCAAATGTCAGTCAGTGTTTTTGATAGTGTACTTTGCATTCTG

>Gm15ciRNA160

CAAAGTAAATTGGACAAAGAAAGAAAGTATGTATAAATACGCGAAATAAAGTAAAAAAAGCTCGTTTTTCCAAAGGGATTAATTATGAACAGATCGACGAGATTCTTCAACTGATAGTAAGACTAATTATTAAACAAGAAACAACAGGTTTACAAACACTTACGAATATAGAAAAACTCTTACCCCCACCGTTTGAAAATAACTGTACACAGTACTGTACGACTTGTTCAAAACCAGTGTCAATGTCAATTGGTAAACAACAAAAATACCGTATACTATAAGAATTGAAACTTTTTACGGAATACGTTAAACGTCTAACGTAAAGAGTGAGAATCGTATACTAAAGCTACGAAGGGAATATTCGACACACACGGTATGAATACAAACATTATTGGTTACTCTATATAAACACAGAAAATTACACCATTAAATTTAGAAAGACCCCGAATCAACAATT

>Gm16ciRNA1600

GTAACTCACTTTATTCTATTTGTTGTTCCTTCCATTCCATTCTGCCTTTATCTTATTTATTTAATTTTTTAGATTTCAATTATGTGTAGAGCAGATACGATGTTATTTAGCAAGATCCAATTTTATCAGCCTGCATGACATGCATTTTGCTGGGTTTCCTAAATTTTTTCCAATTTATGCTGCTGACATTAGCTTGGCACTAATTATCAATTTCACAAGCAACCCTATAGGATATAATCTTCTTTTAGTCCAGCATTATCATTATATTCAATTTTTTTTAGTTTTTCCACTTGCATTCTTAACTGCATGCCTTCTGCTAAGTCCTGTCCTTTGCAATCTTGTGTCAACATTCCATTTTGTTTGAACTCTAAGTCACTTTACGGTTCTTAAGTTTTTTGCATTCTTAACTGTGTCTATCTCCTTTCTTTTGAAGCACATTATATTCACACTTCACAGATTGTG

>Gm16ciRNA1601

CAGCATTTCACGAAGAGAGAGAGACGTAACAAGAACGACGATGATGATATAAAGAACAATAGAATAGAAGAAAGACCTACTCCTAGTCACGGAAATATATTATCATAGTGAACATTATCTACGAATAGACTACTTAAAGAAGTACATTCTAACTGTTAATAGACCTTCAACTTACAATAACGAAGACCATTAGATCTAAACACATTTAGGACTAACAACCGTCGAAGGTAAGTTGACTTCTAAATTTTAAGAGTAACATATACGAGACACGATGTATTTTAAAACCATACCATACGAGTAATGAGACCTTAATGTTAGTAGTTTCATCATGTTGGAGTTATCTTCAATTCCAGTACTTTGTTGATAGAGTTTTGGAATTTCAATAATCCATTTCTCATTCCTGAGTACTTACCAAAATATAATATAAACATTTTACAGGGAATTGAACTCTCGGGAAACCGCAAACCGAAACATAATAGTTATTACGTGTCCAGTTGGATGGAACGACTTTAAGTTGAAAAATAATCTTCTTGCTCCTGTCACTTCTAACTTGAGATCTAGTGCACTTGAAACTCTGAACCTATGAAACAGGACTTGATTGATAGGATTTTTGAACAATTCACTGACTTGATAGAATTTTTGACTTCAACAATCCACTTCAGTGTACTTACTAAAATAATAATGTAGAGATTGGTAATTGTTACTTGTAAAGGTTCTATCATCAGTTGGACCTAACTCCAAAAACCAGAAGGAATTCGAATTCATATACTCGTACCTCTTAAGATAACACTATTTTAGAAATTGAATTAATATATATCTTTAAAATCATGTAGATGACTAAACATAAACTTCGTGTACTACAAACAAAGAGTGGTTTTTACGATACGAGTACTAGATACAACGTATTTAAAAACTAACACACTACTAGACCAGTGAGT

>Gm16ciRNA1602

CATTTGTAATCAGAAGTAGTGTCGAGCACAACAAGAGACGTAAGGAGTGAATACTACATAAGTTTCGAAAACTTTACCAACACGTAAAACAAAACAATACAAGGGGTCAAGGCGCATATACAGTTGTTTACAAATCCAGCGTTCAATTTGTAAGTGTGTGTGTGTATAAAACAAATGAACAGTATACATTACAGAGGTTGTTAAATTCTTTGTTCAAACGTAAAGGTCAACGGTATCAGGTACCAACCGACCGGAAGACGTGATAGGAATAATCTATATGTATAAGCAAAGTAAAAGTAAACATTAGCACAAATCAAAGAGACATTGTCTTAATCGAAAAGACAATAATAAATCAAAGAACGGCTGAGTCGTATATTATAAGTAGAGACATTAAAAGTTAGATTACTAGTCTATTGTAGAAGGGGAAAGAAGAGAGAATGGATTTAAGATCATTGGGTATTTCAGATCGTAACGGCAAGTAAGGGTTTTATCCCTTTACCGAGTATTACGACTTGAACGTGTAAAAGGAACCCGTACGGAATAAGAATAACTGTAACAAACAACAACACTGATGGAAAGTTCATAACACTCTATCGTCGTAGTTCCATAAACTTTTATAGTCAGAGAAATGACTAGTAACTAAAAAAAATAAAAAAAATTTTAAACAGATCAATCAAAAAAAAACCTACTCTATATGAAGGAACTTTTATAATTTCAGTCA

>Gm16ciRNA1603

GTATTGTATAAGATTGTCAAACTTGCCAGTTTTTGTTGAATTTTAGTTGGTAGCTCATAATCCAAAAGATTTAGACTTGGCTAGAGTTTGGTGAAATTTTACATCTTGCATCAGATAATGTTTGCTTGTTACTTGCTCCTGATTGTTGATCTTGATTAAAGCTTTCTAAAAAAGTAAATAGTGGCGTTTTTGATTTATTGGCTTGTATGTGTCTGTACATAAAATAAGGGATACAAATCTTTGTTATCATTACTGATTGCTCTCAATTTTCTGGTCATGTATCTTATGCGGTGGTTTCAACCCCAGAACTAGAAAATATCTGAAAATGTAATGCTACTGGTATTTGGATATTGTTCTAACCTATTAGTGGATATAGTTTGGATAGCAACTCAGGCAATTTATGTGCTTATAATTATATACTTGAAGATTCTATGAGTGCCAGATGTTTGGGATAGCAATGTTGTGATAAAAGAAATATATAAAATCCCTTCATTCTTTTGTAAGGTGCAATGTTTCTGGGATAGACATCATAAACCATTGGGGTGATGAATATCTGTTGTGAAAACAGCTTCTAATTGCATAATTGATAATATCTATCTATTTCTTTTCTTTTCATCAATGATCAAGTGTAATTCTTA

>Gm16ciRNA1604

GTGTGTATTGCATGTGTTCTGGGCATGTTTTTATATACACATTAATTTAATTACCTATCTAGTATGGGTTTGGATAATTGCTGATATAAAGTGATGTGTAATTTATATTTAAATATTTTTATGATAAAATTAAGTTAAGAGTAAAATTTAGTATAATTTTTTTATCCTAGTTAGAATTGCTTAACTCAGGATCAATTTTAGATCCAAAATCGATTATGAATCTTTTTTAACATAAAATCAAACATATAATTTTTGTTCTAAAATCAATTATGAACTTGAAAATAATTCTTTTTTTTTTTTTTGCTTTTAACTCATAATCAATTCTAACATGAAACAAAGCACACATACTTTTTTCTTGATAATTATCGGAAACCTTGAGCACACACAAAATGAACATTTCCCTTTTAATACGATTTATAACCTAAGGACAAATTATAATTCGAACCCTAAACCACTTTTATTGTGCCAAACTCTTATTGATCCAAACACATAATTAATACAACTGAATGGATATAATG

>Gm16ciRNA1605

CACTAAAGATTAACTTGAATCAATAATATGGAATATTAAATCAGGTTTTTAACAGTAAAATAGCAGTTATGTACCTTTTGTCATTAACTTAATATACCCACCCCTTCGAGGTACAGGTTTTCATTTACTGTACCAATTACGGTTAAACCGTGGCAAGTAAGTCAAGAACCTCACTCGTTAACGTTTTAACTAAAACTTACTTTAAACTAACACATTTTAATTAAAACTTATTTTAACCAAAACCTCACTTCACTATACAAACCTACAAAAATAAACTTTTCGTTCAATCATCATTTTAAAACATATTGAAAAAAAAAAGATTGTGTTCGATCGAAGTTGAGTTATTTAAAACCTGAGTTAGTTTATAGAATTACTCTAGTTTGTACAGTTATAGAATTACTCTGGTTTGTACACTTGTAAAGTTATACGTTTATTTACCGAATTGCGGTTACGTGTTGGTTAAATAAAAAT

>Gm16ciRNA1606

CATACGAAAACAAGAAAAGTAATAACAACAACCTCTGTTCACTGAACCAATAGAGATAAGAGACCAATCAATCCGTTACACCCTTAGACACTCAATCCCTAACTCTTTAAAAGATACTATTTATGTGTACAGTTTAAGTGATCAAAACCAAACGGACCATACAAAGGATTTCTGTTTACTATGATAATTTGGTTTGTCTATCCTCGGAACGAATTCATATAAAGCGAATGTTGTAGAGATATACGGGACGAATCAAGGAAAACCTGATACCACCACATTTAAGTTAAGACTAACCTGGCAGGTTAATCCGGTTCCCAAAATAAATACGAAATGTCGTATTAAACATAGACATGTTACACTTCAACATTTGAT

>Gm16ciRNA1607

GTAATTAGGTTTTGAAATTTTCAAATTGAATTTGAAAACTAAACCAAGAAACATACACAGTGCATTAATTCATACCTGGAAAGGCAAAACACATGACTTTCGATGAAAGGAAAATTCTTTTATCCAGGTAAAAATTAAAACATTAGCATCTTTAGATGACCCACAACGATTTAGTCACTAGTATATGTGGATGTAGCATATACATAGTAATTATGCAGCGCACAAAAGAATATTATTCAGGCACTGAGACTCTTTCTAATTTTCTAGAGAACAAATAGATGTATTAAATTAATAAGACACA

>Gm16ciRNA1608

GTAATTATGTTGTGAAATTTTAGCATTGAATTCAACTTGATAACCATAAACAAGAAACAAGCACAATGCTTATGGGGCCACATAATTACTATTTATACGTTATATGTGAATAAAACTTTTCGTGTAATCTAAAGACCCTAGTTTTTTATGTGGAATAAAGAATTTTTCTTTTCCTGGTATGAATTTCAAGTCGAATTATAAATAGTAATTCTGTGGCTCCATAAGCAGCTTGTTGTGGCACTGGGACTTTTTCAATCTTACAGGGAGCATATAGATGTACTAAAATAGTAATATATGCCTCTTTGGGTAAAT

>Gm16ciRNA1609

CAATCAATAAGAAAGTAAACAGTAAGCGGAAAACAACTAAGGTGCAACGAACCACAAAACCACTAGCGAAAAAATATCATAAGAGTTAAATTAACGTCACCTGGAAAAATCAAAACCCACTAAACTCTAATGGAAACAATTACTTTAAAGTTCATCCTTAAACCCCGATGACGAAGCAAAAATTCAATAGCCAGAATAACACTAAATTGTAGACCAATACACAGGGGTGACAAAAAGGTGAAGGCTTTCTCAGTTAACTTTTCGTCTAGTTTTAACGACAACGACTATGAAACTAACTCCAGAACAAAGGAGAGTTCACAAAGAAATCAGCCCTAATTCGATATTATCATTATCTCGTACACTACTAAGGGGGAACATAAATATTACAAAATTCATCTATATGATAACATTTTACTTTGAAGCACTTAGTTTTCATTTACTTCAACTCCCTACAACCCTTTGACTTGAGGGAAAAGTCGAAACGTTTGACTTGAGTTAAGGGGAACATATGATTCAAAATTAAATCACCTTGTTCCTAGAGAATCCAAGAAT

>Gm15ciRNA161

GTAAATTATCTTCCCTGCCTCTGCTTTTCTCATTGGTCCATGATTTTCCTTTTATTACGCGAATGAAGAAATTAATGGGATTATTAGATGCTTATACTTCTAATTTGAAACGTGTTTTGATATAGTTTGGGGGAATTTGTTTCCTTCTCAGCTGGCTGGTTGATTTAGGGTTTGGATCCGATCAATTGTTTTGTTC

>Gm16ciRNA1610

CATACACTGTTCAATGGAATATTAAGACCAATTTTAAAAATAAAACTACAATCGACACATTGACACAGACACACACCGGATACATACACTAATACAGTGTAGTGTACAAGTAAACGGGAGTTGTACAATATCGGTTTACGTTATAAGTAAAACTTGAGGTGACCTACACTGAAAAATTAGATTGAACACTAAATTATCTATAAAAACACCTCATGAAAACAATAATAAAACAATAATAGTTACATTCTTTTAGAGTTGATCACCCTATTCCGAAAACAACAACAACAAACAACGTAATAACAATATTCGTTTAT

>Gm16ciRNA1611

CATTCAGTAGGTAAGACGAGGATAGATTTCACTACGATCCGACGAAGTGCGTCCACGCGAAGCGAGCCGGTGTACTACTTGTACAAAATTCGATTTACAGCTCGCGAATTCGCCTCGAACACTAGTGAGCAAGGAGCGTACAAGGTTAGGATCATTTCTCGAAGTCTA

>Gm16ciRNA1612

CATGGCAAAGCAAGGAGGAGTAATGTACTCAAAAATGTATAATGTGAATAAAAACAATAAGTTCAACAAACCATCTCGAACTATGAAAAACAACGGCGGAGAACGTTATTCAAGGTCATAAGGTTTAAGGGGGAAATGTCCCTATACAACCATAAAACTAGGATCTGGGTCTATCATAAAAGGAGATCAAATATAAATATACACACACATAAAAAATAAGAAATCAGTCAAAGCTGGAGTTCTTAACCTACCCTATGGGGACCTTTAAACCATAAAAGAGCTGTATAGACGGCTTCCTAAGAT

>Gm16ciRNA1613

CATACATAGGTGTACGAAGTAATAATTGGTATTGTGGTTAACCAACCGGTCGTTGATAGTAAGTTTAAAACATTTCTTTTCCCTTTTCTAATATAGGTTTTATGATAAAAAAAAAATTTGCATTTATGTTTTGGTAACTGATTCCCTTAGACTGAACACTCTACTGTGAATTTGATTTGATAGTAAGAGGTTTTCTTTTTTCTTTTTTTTTTACCGCGTACGAAAGCAGAACAAATGA

>Gm16ciRNA1614

CATGTATTTTATCAACAACACTCCTAAACAAACTATAAAAATGTAAATTAAGGATAACATTAAAACTGTCTTTTTTTTAATCTTCGGAAAATGAGTCAGTTAGTAATTCAACAAGAAAAGATACCTTTTACAAGGTACCTGTGGGATTATTTTCAGATATGAGCCAGTCTTTTTCTTTATCGTTTCTCTCTTTACTATTTACATTATCCACTATAGTACGCTGTTCTTCTCTCTCTATCCTTCCTTACCCCTATAATTAACTCACAAACGTAACAACCCTACAGTTTGATAATGAGAAAAAGTTTGATGAAGCGTACAACTTTGATT

>Gm16ciRNA1615

GTACTTATTCTAACTCTTCTCAGACCTCAGGGTGTTTGCATGTGATTTTTCATTAGACTTGTAAGTTGTAACATCTCCATTTTTCAATGCTTAACATTTTGTGGGCAATTACTTTCCGTGCTGGCAAATTAAATTGAGAAAACATAGCACAGATCATTTGAACATGGAATAATTTCCTTTAGACTTGTCTAGTTATATACACAACATTGTTTGGACTTCAAGGAATTAAATCCCTTATAGTGGCGGTTCTCGTGCCTGCATTTGACACTATACTACTATCAAATCTAGTGATAGCTTCTTGTTCAAGGAATGTGCAAAAAACAAAAGCAAGTTCCCAGGGGACAAAACATGTGAATTTATTTAGACACAAACAGATACACATTACATAACCATTTTTTTTCATGCTTAATTAACTCTAGTGAGTTGCTT

>Gm16ciRNA1616

CATCATCATCATCATCATCATATACGTATAAGTACGTATAGTTGTAGTGAGAAAGTTAAAAGAAGAGAGAATAAAAGGTAACTTTTGGAAATAAATAAAGGACGTTCGTAAATAACTCTATCTACCGAGGGGTTGGCGTAGCAAGTGTTCTGAATTAAAGGTAAATCAGGTGTTTCAGTTGGTGTATGAATATGTGAAAAAAAGGTTAAAGTGGTCTTTTTTTCTATCTATGAAGTACCATGATGATTGAAATACTTCTATATTCTAACCAACCCATCAATACTTTTTTTCTCTTGCTCTTCTGGTCAAACTAGGGAGTACTACTGTTGTTTGATTGGTTTTTTTTTTCATGA

>Gm16ciRNA1617

GTAATTAAACTAGGCAGGGTTTATGTTTTACTCAAAAGACATGCTTATTATTATTATTATTATTATTAATGTTGTTAACCATAAAGTAGAATAAATAGTTTTTACGTAGTTAATTAACCATCATTTAGTATAATATTATGAATAATCATCGATACTTTATAGTGTTATTCAAGGTTTTAAATTCTGGTCATGCGGTCGCAGTTTTGTCGCAATTCATGATATTATAGGAAATTGCCAACAAATGCAACTGAAACTCCAAAAAATTTGATTTTAAAACCTTTTGTATATTATTCTATCTTTGTGATTCAATATTGATATTTCTAACATCTGCCTTGTACCTTGTTCGGTTCATCCCTGACCTCTTGGTGTGTA

>Gm16ciRNA1618

GTAATTAAACTAGGCAGGGTTTATGTTTTACTCAAAAGACATGCTTATTATTATTATTATTATTATTAATGTTGTTAACCATAAAGTAGAATAAATAGTTTTTACGTAGTTAATTAACCATCATTTAGTATAATATTATGAATAATCATCGATACTTTATAGTGTTATTCAAGGTTTTAAATTCTGGTCATGCGGTCGCAGTTTTGTCGCAATTCATGATATTATAGGAAATTGCCAACAAATGCAACTGAAACTCCAAAAAATTTGATTTTAAAACCTTTTGTATATTATTCTATCTTTGTGATTCAATATTGATATTTCTAACATCTGCCTTGTACCTTGTTCGGTTCATCCCTGACCTCTTGGTGTGTAACTTGCT

>Gm16ciRNA1619

GTATTATTCATTTATATGTCACTTCATTTAATTTGTAGAATATATGCCCAGAATATTACAATGCAGAATTTACAGATAGCAGGAAGAAACCAAGCAGATGATGAAAAGAATAATGGTCAATTACTTGACTATCTGCATCCTGTATTTCTTATATGTTGATTTGTTTTCCTTGACTAACCACTGCTGGCATGTTTGAGCGGCCTTTAGTTTTTCAATTTAAATTTCTTGTGTTTGTTGACTTTGACAATTTTGTTGCTCTCACTATCATAAAATGTGAATAAGTTCTGTGATCTCTTGTGCTTGCCCTGTTATCGTAAATGCTGTGACTCTTCT

>Gm15ciRNA162

GTGCTGTTTTATTTGTCCAGTACATGTTATTTTCTTATTAGGCACTGTAAGAAGTGGCTTGAATTTATAGTTATCCTAACAAACTGGTTATATTTGGACTTCAAAAACAGCCACTTAGTTCTTACAAGTTTGACATGATTTTCTGTTATTTGCTTATTTGTCTGTTAAACTGGTTCTTTAACCTTGCATGTAAAACCTTCAAAGTTCTGTTATTTTTGTCTGTTAGAGCTACTAGGCACTCTTGATTATTGTTGAAAAAATAGAGTCAAGAATAAGGGTACATTGTTGTTGATGAAAATATAGAAAGGCTGTAGGTTCAAATCTCCTCTAGCTCACACCTGTATTAGACTAGAGGGAAGAGTAATACAATTGTGCTCCTTTCTTTCAAGTTTCAACCACCCCTCAACTGTGATAGAAACAATCAATACCTAGAAACAATCCCCCCCCCCCAATTAATCAGTTTTGAATCTAGTGGTAGCACAACCTTAACTCATTAGACTTTTTTTAATACTATTGTTTGATTGGATTTTTTCACAAATTGATACAATTTGTTTGTGCTTTTCTGGCTCTTCTTAATTTATGGTCCTTA

>Gm16ciRNA1620

GTGAGTGGCTTGTTTCTGTTTAGTGTATGGCTTAAAATAGATATACTTAAGTAATTTTTATACCCGCAAAAGCACAGACATGATTGGGGTCAAGATCCTTCTCTTCTCTTCTTTTTTTTGGGTCACCATGACATTGGTGCGGTGATGACTAATCTCGCCGGGAAACTTGGCTGGTTACCTTTAGTGAGGTCTTCCTCCCA

>Gm16ciRNA1621

GTAACATACATGTGCTATCCTATCTTCATAGAGTAGAAGTGCCAAAACGGGCCACCCTTCCTTATGTGGGCTGGGCAAGAAGAAAACCCGATTCAATTCAAGTCTCTAAGACGAAGCTCAAGCATATTGTTAAAGCCCCATTTTTTAAAGCTTTTGGTGGGTTAAGCCTTATCCGGGCCATAATCAAATCAGGCTAAAAAGACTTGCGATTAATTAGTGGTGCTCAAAATTTAAAGCTCAAGTCTTATATAATTATATTTCATCAAGCTGGGACAGCTAGCAGAACACATTTTGCCACCTCTTATCTCAGATTGACCATATATGTCATAATTTCTT

>Gm16ciRNA1622

GTATGTGCTGTTTCTAGCTTTCTCCTCTTGTATGGTGCCAATTCTTTCACTATGACAACTCAAATAGGGAAGAACAGGAGAATAAATTAATAGAATTTAAGGATTCTGGTGAATTTGACATCTTCTGTACAATGCAAATTATTTTTTTAATACATTTGTTAGGTTGATTCATAAAAGAAAGAATTACAAAGGGTCAAACTTTACCTAGGGGCACCTGGAATTCCTGCCCTTTTCCCTGTGCAATAGTGCCATAATTGCTTTATTTTCCATTTGTATCCATAGCTGAAATTTGATTCAGATTCTCTTA

>Gm16ciRNA1623

CATTATTTTGTAGGAGGTGTACTGGTCCAATAGACATAGACCAGGATGAACGTATAAACCGGCGAATTAACATCTACCGGTGTCATTATAAACTGATCACTATTAATAATCACCACTACCCTGTATCTCTTAAATTTTTATACTTGAAATTGGTACAAAAAGTAGAAATGTACTGAACCAGAAATTGAGCAGGAGTACGTACTAACAATAACGAATTTTGACTCTACATTAACAACCTTAGTAACAAAAATATACATGAAATTGGTACAAGAAGTAGAAATGTAAAGAACCTTAGCAACAAAAATATACATGAGAGAAAC

>Gm16ciRNA1624

GTAGGGTGAATTTAAAATTTCACTCAAATGGCTATGGAGTACTTGATTTGGGCATTTGTACAATACTGCATGAAGTATTTAAAAGTGGATATTTGTTAAAATCGAGGAATTAAAAAATGCTGGTTCAATTGAATACGGTGATGGATATATGGCACTAGAGTCATCTGATCCATTTTTATTTCCACTTAAAATGGTAACATGTACATGTAAGTTGTAACCAAGGATTTCATACTAACAATGTCACACAAGTGGAAGAAGGAAAGTGTGTATTTCCAATAGAATATCTTTTCCCATGTACATGAACTT

>Gm16ciRNA1625

GTAGTTTATCTCATCAATTTTATGTATTCACAATGGTCAATTGCTGCTGAAGTCTAGAAGAGCTTTTCCACTATGATTGAAGGTTTTTGAATTAGCATTGATTATGTGACTCACGATAAAGATGGGGTCATGAGTCACCATAAATCTTAACAAGGGGAGAGTTTTGAAAATATGATTAAATGGTATTTTACTAGTCTACTAGTATAATCACATGGCAGCTACAAAGATCAGATAATGCTGTTGGGTTAGACATGCCCAAATTGAAGAACCTTTTGCCTGGAATTTTCAAAAACCAAAAGTATTTGTAGTTCTCCCGAAAATTGATCATTATGTTTGAAAATAATTTGTAATTGAGTTATACATGCCTCCATCTAGCAGTGCCTCTGCTGTCTAGCTATAAAAATTCTGTACACACTACTAAAATTAGATGATAAGTATTCTTTCTATCTATTTATTCATTTTCTTA

>Gm16ciRNA1626

CATTAGAAAATAGAAATTTAAAAACAAGGGAAAGTGTGTAGAATGTTAGTCTTGAAGTTTTACGAAAACAATTGTTACGACTTTTTTCTACCTTCTGTGTAAAGGAAAAGCTTAACAAATTCTGGGATAAACCTATTTGAAGAGGTATTCATGAATTCCTTCTTCTTTTATTCCAGTTTACTTAAGTCAAAGAAGTATTCTATTTTAGTTGAATACGAATAGAAACTATTTTCAAGAGAGTAGACTGAAGAGGTTTCAGTTCCACTCATTCAACTAAAAGTAAGTTTGTCCTGGAATGAGTTTTACTCCAGATATCTTTACCACGACGTAACAATAACACCGCACTCGA

>Gm16ciRNA1627

GTATTTTCATCATTTTATAACTAGCTTTTGTTGCATTAGGAGGACGTCCTTTTTGTACTTTGCTAATTACTGTTTCCTAGTTTAATTTTGATCTTTTGGTATATACTGTATTCGTGATATACAAAGAGGATATGCGGGGCTCTTATTATAAGTTTTTTTTTTATCAAGTTGTGATGATTTCTTTTTAACACATTGATGCCAAATGGAGATGTAGATTATAAGAATGCTTCATTTTTAGATTGCTA

>Gm16ciRNA1628

GTAAATGATCGCTGTTTGTTTCATAGAGAAAAGAAAATGTGTTTTTATTTTCATTGTTGTAGTGCGTTGTTTGTGTTATGCTGAGTGAAGCGTCATTATCATGCAAAACAGATTGTCGTGTTTGAAGTGTGGTAGTGTAAGTGGAAATGTGGAATGCGTAGTTTATATTGATGTTGTATTGATGTTTATTGGCTTCAAAGTTTCATTGCATCTGGTTATGGCATGGGTTGGTAGTTCTGAAAGTGGCGTGTCCTTA

>Gm16ciRNA1629

CAAAGTGAATCATGGAGATTTAAATCTAATGAGGATGAGTGGGAGAAAAGGTAAGAACAAAAACATTCTTTGCTCCTAAACTCAATAACGATAAACTAGGAAAAATTTAATTTTTGTAAAAAGTGTTTTATATATCAAAAAAGACAAACACCAAAAGTCAATACACGATCTAAAATAAGAAAAAACCTTTGCCAATTTAAAATTGTAAGCCGTTGACACTCCAGTACCACATGATTTTTGTACGAAATAGAAAATAATAACAAGAGCCACAGAGTCAAAGTAAGAGGAAGAGCAAACGCCTAAAAGCTAAGACATTACTCAATTCAACTCAAGACACACCGTTAGGACCATTTAACGGACACATGTGTTAATACAGAAGTACAAGTTAAAAACCGTAAGGTTTTATTGCTACAAAAAATTAAACTGGGCCCTGACCTAATAGGTACCACTAAAACCACTACAACAATCGAACTAAATATATCTCTATAGAA

>Gm15ciRNA163

GTAAGTTTCTTATGCTCTTCCATTTGGCTGATGTATTTAATTTGAGTCTTGCTGCTAAAATTTCTGTTTACAGCTAACGTGCCACGTCTCCCTTGTTAAGATCAATCATGCTAGTTTTTCCTCTTCCCTTTGCTTGTCTCAAATTCTGTGTCCAACCCAGACTCACTTGTTCTGATTTTCCTCCTCTAATGTAAGGGATTCACTTTAGAGAGTAAAGTGAAAGTTATACCCATTGATTAGAAAATCAATGGTTGAGATTTAAATAGCTAAATATTTTATTAAACATTTGTATGCATTTAATCACAATCCTGACAGTTGATTTTTCAATCAATGGTTATAAGTTATAACTCATCATCAGTTTACTCTTTATACTAAATCCCTTAGAGCATCTAAAGTACACAATTGTTTAGATGGGTTGTTTATCTTAATTTTATAGACATTACAATTTCATGTAGATTTAACCACTCTACACAAAATTTACTCAAATGTTAAGTGGCTAAAATGAATGTTTAACTTAATTTTACAAGTCCTATAATGCTAAAAAATGTGTTATTTGTAATATAAATTTGTTAAGCAACCGTGACTATATAAGCAACTTGATGTCATCTGCTCCAGTAAAAAAAAATAAACAATGTTAACTTAAAGTTAAGCATCTCATGTATGCACCCTTATTGGAGATGCTTTTGTGTGTCAAATCTCTTTTGACGACACAGCTCTAAAGCCTTGATTTTGTTGTACTTCTTTTTTTGTTTCTCTCTACCAATTTACTGAATAGTTCTTCTGCATTTGATGCCTGTAATATCTTGGTACTTA

>Gm16ciRNA1630

GTAAGTTTTTTAACACAACTAGTTGGGCTTGGCATTTGAATTATCTTGGAAGTTCTAGCAAATCTGTATGCACACAAGTCTGTTGGTTTTGGTATCACAATATAGAAATAGTGACTGTCAAATCCCAAGAACACACTGTCACACTGTTTTCAATTTAAAAAGTAACTTTGAAGCTAAAATAATTATATAATTGCCAATCTGTTTTAATTTTAATTTTAAAGCTTTTAGTTTTTACTTTATAGTAATTTTTTTTAAGATCCTAAAAATGAGCCAAATGCTACTCTAGTGTTTCCCCAGTGTGAATCAGGTGCTATTTTTTTTCAAGCACTGAAAAATGGAATAGAAAAAGGAAACTGTTCTGGACCTCAGGATACCATATTTGCCGATATTTATATTATATGTAAAGGCATGGATAAATGGATACATAATGAATTTAA

>Gm16ciRNA1631

CAAAGAAAAGATAAGACCTAGAGACGGTGAGTGAAGTGAAGGTTGAAAAATTTTGGGGTCTAACGGTGACATCTCGTATTGCACCAAAACTGAAACAAGTTCATAAACCGTACCACAGACAACCCTTTTATACAAAAAATACTAACCTACTCTCCATTTTCTCTCCCCCTTCCCTTTTCACTGGTTTTAGTATTGGGCTATTCCTCCATCTAAACAAAGAGGGGAGGATTTGTCCCTCTAATCAAGGAAGTAAGGTGTTATAGACGAGAAAGGAGAACAAATTTTTGTTATGTTTATAAATAAATAGCACCAAGAAAAGGTAAATCATCATGTATACC

>Gm15ciRNA1632

CACTCGAAGGAGAAACTAGACGAGAAGGCGTTTGCGAAGACGTAAGAATTGTTGGAATCTAGACCTGGCTTAAGCGAAGCTAAAGTACAAAAGAGAAAGAAAGCAAGCGAGAAAGTCAAAGCTTACGAAACGACGGAGAATGAACTTTACGCTCGGAGCGTTAAGCGAATAGGTCGAGAAGGATAAAGTTAAAGTTACACTAGATGACACCTTGGCACGTGA

>Gm15ciRNA1633

CATACATTTAAGTGAAAGATCTTAATAAATATACAATGACTTAAAAGAAAGGCAAAGTTAATCGTAACGTACATTGAGAGTACAATATCTGTAAGTAATCACAACGTAAAGGAGAACTGTATCAATGCCCCCGAACTATACAAAGTTGATAGTTAAGAACTACACAAAACCGAACTGATCAATTTATAGAATGAACTTCCTGACTCCACAACGTAATTTGTTGAACTCCCGAATCTTTGAGATCTGTTAAGCCGTTTCGTCGTCTTTTACACCTTAAAACAGACTCAATATGAGATTGGTTACGGTTTTCACTAAGTAAGTTATGATGTTTTTGAAGAGTTAAATACCAAAACCAATGTTTTTCATGACACTATTGTAAAACTGTTCGCAACAACGGTATCATTCACATCGTAGGTCAACCTTTTATAAACCAACCAAAGTCTGTTGAAAACTATTAGGAATGTCGTCACACCTTAATCAAAAGAGATAAATTTAAAAAATTATATAATAAGATAATCCGATAATCGTAATCCCCATAGACCATTCGTTATTGAACAAGGACTATTCCTTTAAGT

>Gm15ciRNA1634

GTGAGTATTTATCTTCCTTTACAGCTTATTTTTAATGTCTTGCTTATTCATGTTCCCTTTGAGACATTTAGTTTTGACTTTTGATAGTTATGCATTAGTTTTGCTTATTTATTTATTTTGACTTTAGCTTATGTGTAGAATCAATCAAACTAACCAGCTAGGTACAAGTAGGTCTCGCATGATTTGATTTTCATGTGATTCGTATGAATGAATGGTTTCTGGTAAAATTGTAAGACTTTCTTAATAAATATGTGGACTTTGA

>Gm15ciRNA1635

CACAGAGAGAAGAAACCTGGAAAACAAGAAGGAACGAAAGAAAAAAGTAAATAGAAATGGAAAAAAAGAAGGGAACATTAAATTACCGTACGGTCACTTATATATTTTCCCATCGTTAACCTCGAATTCTACTACCTTGAGTAGCAACTACGAAATAAAGAAAAAATCGTTCCTAAAAGAAAAAACTGGCACATATAACACCTTTCACCCTTAATTTGTAAATATCTTTCGAAAGGTCGTAATCTGAAGTTTCTCCTTGCAAAATGTACATAAGATGAGCATAAGATAACACAACTAAGTTTAACAGAAAAGAGAAAACAAATATATGGGTTTTTATAAATCGGTCTAGGTCCAAAGA

>Gm15ciRNA1636

CATAGATAGGAGGAGAGAAGAGAGTTGAATCAACGAAAATTAAAAAGGAGTGTTAAAAGACTTTTTAAGCACTAGAGTTCTTAAAGTTACGGACTAAGCGACTAGAAGTGAAGGATTACAAAAGTAGGCAGCCTAAATCTTTTGGGTAAATTTTTTTAAACTTAAAAAAACCGTAATTCTAGTGCTTAAAAGCTTCCCACTTTAAGATTCTTTACCAACCTTTACCAAATACACACAGACTAACTTAGGTTACGACTACACTGACATAAGGCAAGTCAGTTTAACCCTTGGAAGAAAAGCATCACAATACCTAATAACAACGAAACTTTTACGTAAAGTACCTTCGTGTACGGAATATGTTAAATACCCTTAAAT

>Gm15ciRNA1637

GTGAGTTAGTGTTCTTTTCATCACTGAAAGACGGTATATGTGGTTATGTTTATTCTGTTAATAGTTACTTTATGCTTGATAGATAATAGATTAGACAGGCACTCACTCATTGCTATTGATTGTCTATCAAAACCATCATACAAGAGTTTGAATTTTGCACCTAATAGACTTTCCCATGGTATACCTCTACACAAATTACAGTAAAATTGCTTTAAGCTAAGCATTCTCAAATATAAAACTGTTGATGTTTATGCCTATTGTCTATTGATTGCAATTTTAGTAGATGTTTGAGTTTATGTATTAAGATGCATGGAAAATATTTTGACAACATCTTCTGTTTGGCGGATATGTTTGTGTTAGTCCATGCGAGACCTTGTATGTGAAGAGTAGCTTGAGCAAAATGTGAAGATCATCTTGTTGAATTTTCAGTTTTTCCCATGCTT

>Gm15ciRNA1638

CATATGTCAAATCCTAACCTTATAGTGAAAGAGAAAAACGACCAAGTAAACAGTGATCAGATACACATTCGATCTTTCTTATAGAAGTAAATCAATATACAAAAGAATTTCGTTACAGATACATCGTTCAACAAATATGTTAACTACTGTACCTGACTTTAAATTTAACACATCCGTTCACCAAACAACAGTAACGAACTTAATGTGACAGAAGAACCACACTGGTGGTTTTCAAAGTTATAAAGAAAACCCCATTATAACGTTCGTCTGTTAGTTCATTGTTCATCTTAAAATTGGACGTATAAGTTTCTTTATGTGA

>Gm15ciRNA1639

CATTCAAGTTACTGTTTTAAACGACATTTAGGGACGTAAAATGAAATCACGTGTGTCTTTTTAGAGTTAAACTAGGACACTTCATAGTTCATTACGTACAGTAGTGTAATCAGGAGGTTTATAAAAACTGTGGTTTAATCAGATAGTTTCTAAGAAAAAACAAAGGTAAAATCAGTAAAGTTCTAAAACTACGGTTTAATCAAAGATTTCTAAATTAAACAGCGGTTTAATCAGGGAGTCTAAGAAAAAAAAAGTGGTGAAATCAGGAATTCTTCCTGTTTATGCTACTCTAACATCTAAAAACTCTGATGATTAAATCACTATCCAATTTGGTAACTCCCTAATTATACCACTGTTTTATAAACCTCATGATTATACCACTACTAAATTTAGACTCCTTAATTAAACCACTGTTTTTAAATTTCCTGATGGTGTGTAGATTAAAACCTCCTAGTTTATCTTCTAAAAAAGAAAAATTAGACGAAATATTAAATACGTATAATAAT

>Gm15ciRNA164

CAGTCATGATAATGATAGTATGGTGAGAGCAAAAGGAAGGAAGGTGTCAAGAACGTAAGTAATAAGGTACCAACAGTAGTTCCAAAATTTATAGGTAGCAAACGTAACACTAAAGCAGCGCAAACAACTATAGTGTGGTGTAGCGTCTGTTTACGTCGGCTACGCCAGGGACAATACCAGCGCTTGTTAATTTTTTACAACTACAATCCCAGGTTTAATGCCATCACCTAACAAAAAATTTTGGAACTAACAACATTTACCCGGTACGTAAATTTTTCCGTTGAAGGTCTCCAAATCTTAAAAAAAGTTAATACGAAATAAAATAACAAAATTACCCATCCTAAGTCTTTTAGGTGCGAACCAATATTTACACGATGACTGACTCACACACCTACGCACTTTTTAACTAACGTGACTTAGACGAAGAGGAACAAAAAGAGGAGAAGGGAACAACAGTCGATGATGGTACCACAAACAATCTGTCAACAGAATACAATTCACTACTGAAAGCTTTTCACGTACT

>Gm15ciRNA1640

GTAATTGGAGTTAGTTCTTATGATTATGATTTTTTCATACAACATGGGATGGTTTTGGGCCTCTGGGGGGAGCTGCAAAACAGCCATAGAAACTTCTAATCTCTAATTTTGATTATTTTCATTTATATTGATGCCTGGTTTGTTTATAAAACCATATTTCTCTTCTACATCAATAGGAATCATTTGCCAGTCATATCCATTAGATCCTCAATTCAATTTTAATCTATAAATGAAACTCA

>Gm15ciRNA1641

GTTAGTTATTTTTTATTGTTACCTGAATTGTTGTAGCATTATTACAATTGGAGGGGATGACTTTTTTTTTAATTGCCAAGTGCCAACAACTATATTCCCACCTCATGTCATTTTTGTGTGGTGAGTAAGGATGCATATGATTTTTTTTTTTTCAACGAAATGTTGCGTTGAAGAAATTTTTGTTTGTGGTTTATTTTCCTCTTTAAATTCCCAATATTCCTTAGCAATTTTTTGGCCGCAGTAATCAGTAGCTTGGGTTAGTTATATCGTACCTCCTTGTGTCAATTTAACAACCGCTATAAGTGTAAATTGTTGTTAACTGAGAATATAGTGATGGCATCTACATTAGTGTTCTATTAGATAGTGAAGCTTCAATGCTA

>Gm15ciRNA1642

GTAAGAAAATTTCGCTGATGATTCCTCATTCCTTTTCCTGGATTTAGCTCCTCTAAAGTGAGTTGTTCACTTGAGGGTAAATTAAGCAATTTTAGTCATTGATTAAAAAATCAATTATTGTTATTACATGCACATACATAACAAAATTCATGGATATGTGTTGGAATATCAATTGTTGATTTTCTCATTAACGATAGAGATTACTCAGGTTATCCTTGCCCACTTTAGAGGAGCCAGATTCCCTTTTCTTCTTTCTCTCTG

>Gm15ciRNA1643

GTAAAGGAATTTCAATCATTGAATTGATTACTGTGATTATTAATATTAGTTTCATATTATCTTCAATTTGTTCCTATTCTGCTGGTATGTTTTCTACTCAACGTTAGCCTACCTTGAAAGACGACCCAGGGTTGCTAAATGCAGATCCTATTAATTTTCTATCTGGAATTGTCATAATATAGATGTATGTGTTTATTAGAAGCTTTAACTACATATTTTTTAGGTGTCTGAGTCATGTTAAAAGCATTTCTACGGCAGATGGAATTAAAAGGAATGATCTACCGGCAACATAGTTTCAGCTTCTGCCCTTGATACAAGGGGGTAGGCGAACTTGGACAGCCTGTGAACAATTCATCCCAAAACTCAAATATATGCCACTAAATCACAAAAGTAAAATATAAATGAAATGTGTAGAAAAATTCATTAGTTTGACATTTAGAATAAAAAAAGCTTGTGATATTTCACAGGCTAGAATGTCAAGTGAAATAAAATGGTTACCCAGTTATTCATCTGCTTAAAGTAGTTAAATCGACAGCAAAAATCATAAAGCAACTGA

>Gm15ciRNA1644

GTGTGTGTTTTGTTGCTGAAATTAGTGACCACTGTCCTGTATCCTGTATTCTAATGTCATGGTTAGCTACAGGTGTTTTGTTTCAGTTGCTGTGTCATTATGATTTATGAATAATACCTGAGTGGTGTGGCGTGTGAACGACAGTAACATAGTGAACT

>Gm15ciRNA1645

GTTATTTCTTAGTCACTTATTTTAGATAGAGCTTCTTTACTATTGTTTTATGGCGGTGCTTTTGAGAAAGTTATGTCATTTGTGTTGGAATTAGGAAATAATATTTACATGATTGTTTCACATTTGGATCTATCTTGATTGTATTAATAGCCCTGCCCTGTCTTTTAATTAATATATTTTGTGAGTGCTGGGTATACCCATTAGTGTGAAACAATTTTTATTCAATTTTCATTTCCGGGGGTAGGAAGGAGTTAGGAGGGAAACAACTTACTGTTGCTAGGTGATAAAAAAATTTCATGTGTATACTTCAATGCAGCTTATTTACTATTGCAATGAGTCAGTGTGACTCAAGTGCAAGTGGTTCCATACTGAATTTTTTCTTCAATTCTATAATTTTTGTGACAGGATCATAATCTTTCTATTTGTGAATTTTGTACAGCAAATAGGATGTCTATATGTTGAGCTGATAGTTTATGTATATTAATTATATTGAAT

>Gm15ciRNA1646

GTACATTCCCCTGTGAAGCTTCTGTTCTATTAGTTTATGCTTTTGCACATTTGAATTTAGTATAATGGATTTATCTTAGGCATTTATTAGGGCATATTTATTTGGCTGCTTAGGGTCACATTCACCAAGAGCTAGGCAATTCCAGTCATGATAAACTGTTAATTTTAGCAGTTTTTTATTGTCTACAAATCATGATCTTTTACTTTTATCATTCACAAGATCACAATTCATGTTGGATGTAATAGAAAATTGAGTTCTACTGTGATTCCCTTGACTTCTTTCTCCT

>Gm15ciRNA1647

CATATAATAACAAACAACGATGCAAGAAAAGGGGTACATATTGATTAGTTGTTGAATTAAACGTACGTAGTTCACAACTCTTTGATACTTAAAATAAAATCCTAAAATAGAGTGTGAGGATCGGATCCTATGGACCCTATGTATTATTGTACAACTAAGTAGAAAAATTGATCTTAAAATTCTTAAATTCAAATTCAAATACTTAAAAAAAATTCTGAAATAGAGTGTGAGGAAAGGATCCTATGTATTATTGTACAACTAAGTAGAAAAATTGATTTTAAAATTCTTAAATTCAAATTCAAATACTTAAAAAAAAATAAAATAAAATTTAATACACTAAAAAGAAACGATTCAACATTTTTTTCCTAACCCCACTACACTCGGCAATCTAGTACACTCTTTAGTTGCCAACTATATATACATATGACACCATATTA

>Gm15ciRNA1648

GTATTTGTCTCTCTTTTCAATTTCATCTTAGTTTTAACTTCCTTTGCTTTCATTTAGGTTTCTGATTAGTAAACATTTCAATCGAATTGCACTTGCACGAATCCGTTTCTTATATTCTGAGCGAGGGGTAGTGCTGTCATGTTAACACTCAGCTGATTAACCACCCTCCTGCCCACGAGTTCCAACCTTGATGGGCTTGGGGTTCTAATTCAGGATTCTTAATAACATAAGTTATGTTCAGCAATTTCAGAGTATGCTAGTATTTTCCTATCTTTCTAGTATCTCACTGTCTTTATAGTATTTAGGTTAAAGCTTATGAGATATTTTCAATTACAAAGCTCTA

>Gm15ciRNA1649

GTACTTCTCTTGTTTTGTTCTATGTATATGAATTCCTTACATTGGGTTACTAGAATAGTATTTACTATTTATATACCGGGGGAGGAATATGGAATGGCTTGCATTATTCTACATTGATTTTGATCGTTGTTCATAATTCTTCTTCTTGTGATATGAATTTTGCTGCTTCTTTTGTGTATAAGAAGGAGTTCTGCTTGCTACCTTGTTTGTTTGCTGGAGTTAGTGAAAGCACATGCCACTATCAATTGACTTGATTCTTCTGGAAATTAAATGTCAAAATTCTTAGTCTTGGCTGTTCTTAGAGCATCTCAAATGGGAGTTGTTTATGGAATTATTTTGTGCAGTTTCATCATTGGATTACTTGACGCAACAGTTTTTTATATAAGAACCAGCTGTTATTAGTTCTTAACAATTGTGAGACAATTATAGGGGACTCATTTTATGCTATTAAGGATAGCATCATTAAAGAGAAAATTTGTTGAAATTCTTAAATTCAATGTAACATTATAGGGCCCACCAAAAGTGAGTTAAGCAACTCCACCATGGTTGTACCATTAGAGATGCTGTTAGATCTTTTATGTGTATGCCTTGTGTATTTACACTTAATCAGATTCAGAAATTTCTCTGTTTTCCTCATTGACTGGGCTGGATTTCAGTGCTTGGCTTTTGAACTTTGAAGTGTCCAATTTTTCCTATTGGTATTGATTTTGTTATAGGTTGCTGTTTAATTTTCGACTTCCATTTTTGGGCTTATATTGTTCAGAACTTTAAATGCAACCTAAAAATGAAGAATTTTTGGAAATTTCCTGGTGGGCAAGAAGCAAGATGATGAATTTAGATGTTCATAATATTGGAGTTGAGTCACTCACATGGTGCATCCTGTCAATTCTGATGCCTTAGCGTCGTGCATGCAAGTTTAAACTAAATTATGTGGAATTTGCACCTTTGCTGTTTTTACAATGGTCCACACTCACATTGTGCCCTTTTACTATTACCTTGGATTTCTATATTTGTGTATTATATCTAAGGTGAAATTTCTCTTTTA

>Gm15ciRNA165

CATGCAGACCTAGCAATTTATGACAATATTGAGACCAAAAAGAGAAGGCGCAGATAACCCCTAGAAGACACATAAGTCCCCGACGGTTATTATAACTTTTATATTAAAAGAAACTAGTGAAAAGACTAGGCACTGACACACATCTCATAACTATACAGTAACTTAAGTAAAGCCATGAAAGTCAAAAGAGAGAAAAAGTAGACCCATAAAAGATTTACGACACTCGTGACCCAGAACCATCGAAAACAAAAAAAAGATAAAGAGAATAAAAAAAAGACAAGCTAATAACTAACTAA

>Gm15ciRNA1650

CATACTTTAAAATAATTTCGGTTGTTTTTTACATTTTAGGGTGTCTTAGGCTTATTTAACCTGAAAAACACGGTAATTCGACAAAACCCCAAACAGGAATTTTACGAACACATTGATAACAACCAAGGATTTGTTCATCTTAAAATAAGAAATTACGCGTAATATTTCTGATTAGTTTGAATTAATCCTCTTTCATCACAATCTCATACGACAGAAAAGAACCAAACGA

>Gm15ciRNA1651

GTATGTCTCTTTCTTAATGTTGTTTGTTCTTTTTGCTGCAAATATCTCTTTCATATGCCGCATAAGGGAAAATTGAGCTGTCTTCGTTAGATGAGTGATACTGGCTGAATATAATGCTGTATGTCTCAGAATATTGTTTTTGGTGTATGCTAAGTGTTATTAAGTTATGATTCTTTTCAGTTACGCACTTGAAGATTTGCAGTCCAAACATTTCTTCCTGCAATTTCCTTTAAAAATTTGGGCTAAATAAATTATATATAATAATAAAATCTTA

>Gm15ciRNA1652

CATGAATGAATAAGTGTGCGGATTAAAGAAAAAAACTTCTTTATAGAGTTACATTGCCCGGGAAGTACTAGGAATCAGGAATGATGAGAAAACACACAGATACAAACACAATGTTTTATATAGACCAATACTCAACCGAAACTGATTGAGTTGAGGAAGGAATTTTTTTTTTTTGTCTTTATTGGGTATTCCTAACTTTTTATGAAATTCAGATTTTTATTTCAATTTAATTTTATATATTTACTCCTCGTTGGGAATGAGTCCTCAATTGAAAACCCCAAATTAATCCGGAGAGTATAATAAGTAAAATTCTTATTGAGAACTTCTCTGTAAAGTTACTATAAGTTGAGCACAGATATAAGACGGGTGATATATTAACGAAGTTTAAATACATATGAGACGGCTTATCATATACGTGGCCATTTCTAAAATGTAATTGTTGATTAATGTCCGATTGTACATGATCATACCATAACTCAATGTGGATGGTACTAAGTGGCACATATTCGAAAATGTGATTTATTACGTGTCTTTAAATTGAGATTAAATAAAACACTTTTAACGACACGGTTTAACTAGAAAGTTGTGGAATGAGACT

>Gm15ciRNA1653

GTGAGTCGTGTTCTTATAGTTCGCATAAGCTTAAGAGAAATTATTACATGATTTATGATAATCATAGTCTTTATCAATTTCTATGTCACATATAATAGGTTTATTGAAAGGCTCTACATGACATGTGACATGTAGGTGAGTATAATTCATTTTGAAATATTTGATTATGTCATGTGAAGATTAGTCAAGATCATAAACATGTGAGGGTCTTATACCATGTAATAATTTTTCTCTAGCAAGATTAATTGGTTACTTGTTGGTGCTATTCAAGGAATGAATTACAAGGTTGTTACCATTTTGATTAATATAAATACTGAAGTCAACGGGAGAATGAAGTGGTTGTTTCCATTTTATTTAATGAAGCCATTGATTTAGCTGACTTTCTGTGCTT

>Gm15ciRNA1654

GTGAGTCGTGTTCTTATAGTTCGCATAAGCTTAAGAGAAATTATTACATGATTTATGATAATCATAGTCTTTATCAATTTCTATGTCACATATAATAGGTTTATTGAAAGGCTCTACATGACATGTGACATGTAGGTGAGTATAATTCATTTTGAAATATTTGATTATGTCATGTGAAGATTAGTCAAGATCATAAACATGTGAGGGTCTTATACCATGTAATAATTTTTCTCTAGCAAGATTAATTGGTTACTTGTTGGTGCTATTCAAGGAATGAATTACAAGGTTGTTACCATTTTGATTAATATAAATACTGAAGTCAACGGGAGAATGAAGTGGTTGTTTCCATTTTATTTAATGAAGCCATTGATTTAGCTGACTTTCTGTGCTTG

>Gm15ciRNA1655

GTATGATTGTCATTCCTCTCTGTGGATCAATTATGTTCTCATCCCTGTCACCTTAGTATATGCTCTGTCCAATGTGTTCTGTGGTGTCTTTCACTTTTCCCTTTCCACAGAGCAGGGGTCCACTTGGAATTTTAATATGTATGAAGGATGAACCAATACCATGGTCATATTTTACCAACTTAGGTTATATTGTTGATACATACTGTTGAGGAACTGCCTGTGCAGAATTACAATTAGAAGAATTGTTTATGGACATATTTGCAATTAGGGAGCAAATTAGCCTAATTTTGGAAATTTGGATTACCAAGTTAATTTATGTTTAACATATTAACTACTCTTTGAATTTGAACATTGATTACCGATTCTTTTTTCTTGGCAAATTACTCCCTAAATTAGAAAAAAGTTTGCCAGAACTTCAGTAATGCCAACTAAGCTTTTATGTTATAATCTTGGCTTCAACT

>Gm15ciRNA1656

CAAAGGGACGGGGGGAGAGAGATAAAGTTAGAGAGAAACAAAAGACAAAGCTCCAAAATCTACGAAAAGAAAGTGAACTTGCTTTGCGTTTTATGGAAACAAGATACAAAGCTCCGATAACGAAACGAGCGAACGCGTAATACGCTTTCACTCTTTATTGACTCGCTCTTATTAGGCGCACAAAAACAAAAACCGACACCTCACACTAAAACACCGCGTCCCACGACATTATCTACAATAGA

>Gm15ciRNA1657

CATACCACAGTACCTACTATGAGTAAATGAACGAACACAAGATATTTAACAGTAGTACCAAGGTCGTAGATCCAAAATTTTACGAAACGAAAAAACACGGACCCTCACCTTTAAAGGTTTTAAACACAGTGAAACATGTTCTTTAACATTTCTATAACAATGATTATCAATGTAAGAAGAAGAAGAAGAAGAGAGAGAGAGAGAGAGAGAGAGAGATATATATATATATATATATATATATATATATAAAGGTGAGCACACAGTAGTACGAAAACGAGTTTCAAGGAAAGGGAAAACTCCCTAAAAAAACACACGTTTTGTGGTTTTCCTAGTACGTCACTTAAAAACCCCAGACAGGAACTGAGGACACTAACCTGAAGTCCTAACTGGTCAACTAGATGGTCGAAATAACAAAAAATGACAAATTGAAAACACAATGTTTGAGATTCATTACATTACATCCTGTCCATCTGTAATCTGTTAAGTTACTTTACAACCGACTGAATTCATTACATTAAACTCAACCTTCAGCCTTTACCTTTCTAAAAGTTTATAGGTTTAGTTCCCAAATCCCAAGATTACAAAGAAAACAAAACCTAAAACTTTATTCCTCAAGAAAAATTAAGAAACCGTTTACTTAATTCTGACCTTTGTTTCCGGATAATAGACAATTTCGTAAATAATATGTGAGATCTAAAATCACAAAATACAAAACAATTAGGTTTTTTCTTTCATCGTACGTTACGTGGTACTGAACTCTAACCTGTCAATGGTACCTTTTTCTTCACCAGTTTAGTTTTGTAACCTGTACATCTTCTTTACGTACATAATCTACTCATAATACCGGAACTTATCCGATACACCTAATCTAAGAAGCATGGGGTAACGGGTCTCCGAGAAGCGATACGCTTCCATACCCCTCCCTATAACATGCGTCGGAATGGGAACGTATACGTTTCTCCGATACACCTAATCTAATTACCTTTTTTTAAGTTAACCGCTTGGTATAAGACACAACATAGAATATGGTTAATTAACACCAAATGTATCGACCCCGACTCACTACATCAAAGAATTAGATAACTACACCTACAAAAGAAAAAAGGTCAATATTCTGTTCGACATTATTTTACTTTTAAAAACTATTCAAAATTTTCGTCTTTGTGGCTCTGGTGGAAGAATACCGACTTTCCAACCTCTCGGTTACAAATAGTTTTAGAAAAGTCAAACTTTGGACAGAATAAACTACAACTAATAGATTGAGAGAATTCTCTAGACTACACGTCGAGGAAACTCTTGCAACAAATTGACCAAATGACATAAGTACTCTATAAAATACGAGTTTGGTAACT

>Gm15ciRNA1658

CATTTTTGAGACACATACTACTGTGTTTTCTAACGGTGTATTGTACAAAGTAAATTGATTTACTAGTCGGTAACATGATTAGAAGACGATTTTTAAAAGAATGTAGTTTGAAGGGTCATGTATGAACTATGATGTATGTTCGGAGGTATATGGAATCGTTCACGATCACGAGGGTACATTTACGAAAGGAAAAGAGCTTGTATTAGTAATGTGAACACAAACTCGATCGAAAATGTAAACACCTAGGGTTTCCTCATACTTTAACTTGAGATATTATATACAACGTAACACATCAGTGTACGGAACGAGGATCGTGGAACACGGAACCCTAATTCGTAATGTGAGTCGAGGTGTCATAGAAAATTTAATAACTCATGTACTTGAAGTGAACACGATTACGAACCCTTGAAGTGAACTAGTCCGAATTCCTAGTTCCATGATT

>Gm15ciRNA1659

CATAGAAGACGGTGGTCCAAAAACGATCAAAAGTTAGGACCGATGACGACGTCGAAGAACAAATGGAAAAAAGAAAGAAAAAAAAAACAGAAGTTGTATGGTACGTTTAAAATGGTTCTTGAAACTACCAAAACACAAACTAAGTTGAATAAAACCTGTTTATTATTGAAAGAAAAATGTTCGAAGGATTATCTTGAAAACTTCTCTTTTTTTTATGTTAATGAAACACCCTCTATTCGAAAGAGTTTGAACACGTTTTTGAACTATACAAAAAGATACCTCTTATAGAACAATACTCGTATCATCAGGAACGGGAGAAAAAAGAAAATAGTGGAAAAAGATAATTTTTTTACGATGTGATTATTGCACTTTTAAGGGGAAAATGAATAAAACATACCGGTTGATAAAACAATCACTAACT

>Gm15ciRNA166

CATTTTTGAGACACATACTACTGTGTTTTCTAACGGTGTATTGTACAAAGTAAATTGATTTACTAGTCGGTAACATGATTAGAAGACGATTTTTAAAAGAATGTAGTTTGAAGGGTCATGTATGAACTATGATGTATGTTCGGAGGTATATGGAATCGTTCACGATCACGAGGGTACATTTACGAAAGGAAAAGAGCTTGTATTAGTAATGTGAACACAAACTCGATCGAAAATGTAAACACCTAGGGTTTCCTCATACTTTAACTTGAGATATTATATACAACGTAACACATCAGTGTACGGAACGAGGATCGTGGAACACGGAACCCTAATTCGTAATGTGAGTCGAGGTGTCATAGAAAATTTAATAACTCATGTACTTGAAGTGAACACGATTACGAACCCTTGAAGTGAACTAGTCCGAATTCCTAGTTCCATGAT

>Gm15ciRNA1660

CATGAACGGAAGAAGGAGAGAATGGAGAGATAAACCAAACACGAGTATAAACCAACTTGGAGTTAAAATTGATTCAAAGTTAAAACTCTTCAAGGGAGAACAAAAAAAAAAAAAAAAAAACCCTCCCCCCACTAGTTCAAACTTAACACACACATTCAACAACAAACCAGAGTACACATCGACCAAAAACCGAGGATCTCTACTAACACACACTAAAAGTTACGTTCCAACCTCACTTCTTTCTTTCACACCAACCAAAACCAACACCTAACAAAGAGGAACTAGTTCACATTTGAAGAGAAACACAAAAGAACCTCTTTTATTCCCTTAAACCCCAAAATTTCTAACAAGACCATAACCAACAACAAATTTGAACTAATTCAAGTTTGACAAAACCCTCTTCGTTGATCTTTAGTAAGAAACTCTTGAATTAATTCCAATTCCCATAACCATGACTCCAGTTTTCGTCCCATGGTACTGTTGAGGGGTTAAAATAACACAACAAAGGAATATAATCTATCTGACACACACCTATTTTAAAGAGCTATTTACGAATATATCCTCTTCGTTTATTTTGTGTTTTACTTAGTTCGAAGAAGGTATTCAATTTTAGATGAATTCGTAGAATCAATTTTGGATGATCATTTTAGTAAAGGACATAATACGTATTCTAAATCATTTACAATGAACAATCATAGAATCTGTGAACACTAACGTTTTATATAACTAAAACACTTGAGACGTTATACAAGTTTGTTTTACAGCGTCGTACATCTATCTTGAATCTTCCAATCACTGAAATGTTCCTAACTAGGGTTCCGAGTCATTTGGTCGACTCTACAGGTTGAATGTATATTTATAGCCGAAAACAATCCTACACTTTATCAAGTTCCTTCGTTTTCTAATTTTAGAGTTCTACACTTTCACACCGAAATTGAAAACCAAGTTATACCTTATCTTTTAACATGAAAAGTTATACCCCAAATATAAACGATACATAAAGAAACGATAAACGAAGGTTGACGTCCATAATAATGATGATGGTTAATACACCAGTTACCAGTTACGTATAATTAGTATTGACGACTAAACAAAAAGAT

>Gm15ciRNA1661

GTAAAACGCCCCTTCTTCTTTCAATCTTGCTTTCCCGATCTCTCTCTGCTCCTCTTTCCCACCGCTGCGACGTGGTTACGATTCGTCACCCGCGGATTTCGCAATTCGGATGCTGAAGATTGATGGATTGCGTTGTTTCGTGAAATAGGATTGGGCAAATCTGCGGATGATTTCGAAATTAACCTCGTTTTGGAACAGTTTTAGCTTTTAGTTCGGGGGGAATTGTGCGCTGTTTACTGTGATTTCCTTTTATGAGAGCTGAAATTGTTTGGTTTGAATTTGGAATTTTTTAGTTTTTGGTTGAGGTTTGGGGAATTTTTTTTTCTGA

>Gm15ciRNA1662

GTATCTGTCTGTCTTTCGGTTCTGTCTTCGTTGACTTTGATCCTTTCATTGATCTTAATTTAATGTTACCTGATCCTGTTTTTATGGTATGTTCTGAAATTAGTCTTGTTATGTTCGTTGGTACGTTGATATTCTATTATAGAATAGGTTCATCCATTGATTGATTCTTTTTATTTTTTTTCGTTATTAATTTTTCTATTTCTCTTGAATTTCTTATATTTTTTGAAGTGAAATATATAGGGTACTATAACCATCCGCAAACACAAGGAAGAAAAATAAAGTCTTTACCCTTTCCTTTTTTGACTGTTAGTCCTCATTTCTTCATAATTTGGCTCTGTGTTTTCTTTTTCCGTTTGAGTAAACAGCTTAATTGAGTGTTTACTGAATAACAATCAGCTTATTAAGGGCTTATTAAATTAGTACTTAATACTTACCATGTAAGTATTTATGTATAAGTTGTTTTTATAATCAAAGAAGAAATAAATTTAAAATATTTTCATATATGTTGTAAGCTATTTTTATAAACTATCCTTAAAACTTATTTGAAATAAGCTGAGAATAGTCTATGTATGTCAGAGATAAGTCTAAATAAGCTCTTTCAAACGGGTTGTTATATGATCCTTTTAATGAATCCTAAAGGCTACAAAAGGACTAGTATTAGAAGCATTTTCACAGGATCTTCAAGCATCCTTTTGTATTATAATTCTACAAATCCAGGTTTTGTGATGCTTA

>Gm15ciRNA1663

CATAGAAAAAGCAGTAAGGTGGAGGAGAATTGACATGTGAACAAAGTTGAAATAAAGGAAGAGAAAGGGAAAGTAGAATACCTGTGTGAAACTTAAATGCATGGAGACATAGAAAAAAATAAAAATACAAAAGATGATGTTGAGGTAGTTCTTGAGTTGGTCTAGATACGTGATTAATGAAAGGGAATGTTAAAACTTTGTAAAGTAAAGATTAAGATAAACGGGTTTTATGAAT

>Gm15ciRNA1664

CAGCAATTGTGAGAAGCGAATTTGTTGAGGAGTGCAAAAAAGCGTGCACGGCGTTACGTACAACGCTCCCTCCGAACTAGAGCGAGACGCTCGCGAAAAATAGAAACTCAAAATGACAAGATGACAAAGTATAAAACAAAAAAATAACTACCGAAGGATAGGA

>Gm15ciRNA1665

GTGAGTTGTTCTTCCACGTTTCCAGACACTTATAATATTGTTTTAACCCAGAAATTATGGAACATTGCACACAAGCCTCACTTCACTTTCAAAATCATCTTCTGTCTTGTTCGATTTCCCCAGACCTAATTTCTCGTTCAATGCCATGGTTTTGAAATTTCAATTCGAAAGTGATTTTTTTTCTCTGTTGCTATATCTATATATACTGTTTGTTAACTACTTTCGGTTCCTATTTTGTTTTTCGCTCACT

>Gm15ciRNA1666

CATTCAAAAGACGAGAGACCCAGATGAATCAGACTATTAATAAGTCTTATAATCGAATTCCATCTGGACATTCTACCAGAATTATTGTTATTATAAATTCCGAAATTTTACCTTTTTCTTTACTCTCACTAAAAAATAAAGAAGATCGACAACCGATATTTTTCACCTGTTACGTACAAAATTCCGTCTAAGAAATACAACTTTCAAAAACCATAGTCTTTTTTCTTTACTTACCTTCCTATGTTTTAATTGACAAATTTATGTCACTTATCCAAACTTGGTCTTCGGACAACCTGTTAACCATAAGGACATTCGACATTGTCAGGTAGAACACTCTAATAATTAAAATACCTATATACGTATCAACCGGAAGACAAAAATGAGTCAGAAACTTCGTATCAACTAAAAAAAAACCAATTTAATGAGCCAGGGATTTGATAAATCATTAAAAGTTCATCCAGGGATTTGACTAAAAAAATTAACCTAGGACTTGATTTTTTCATCCAGGGACCCCAATCAAATATCACCGGACTTTGGCAATCTTTTACTGTTAAATACCGCCAAAATTTTTGGGATCCTTGAGTTAATTTTTTTCATGTACTAGTTCCTGGATTAATCTTTTTTTAATTTCAATCTGTGAATGAACATTTAAAGGTTTATTAAGTTGACTCATCAAACTGGAAGAAAAAGGTTATTACAAAACCGACCCTTCCTCTATAGTCTTTTAAGACGGAAACAAAAGATGTAGAGATT

>Gm15ciRNA1667

CATTGACGAATGTTTAATGTTTTACTCTAAAAAAAACAACCAGTGAATAACTTAACGACAATGACCATAACTAGAATTAGTTACGATCATTCATTGAACTAAAAACGACCTCCTACATTTCGTCCAACAGTCCCGAAACACTAACGAAAACAATAAACCTCTAAAACTATAGACACGCAGACAAAAGATACAATAAACACAGCTTAACTGTACAAAACTCTCCGGTTCATTTAATGTCACGTCACTAGGATTTTACCCTAAGGAACTCAAATGTTTCTTCGAGCTCTTCTCAGTTAATACTATAAAAATGATTTACTCAAAGAAAACCACGGACGCACACCTTTAATACAACGAGATAAACACCTCAGACGGTATTTGAAGGGGGTGAAATGTTTTACTCAGAACTTGAAGAACAACCGAAAACACCTCAACTTTTGTAAGGACCAAAGTAGGTGAATTAACTTTTTCAAACGACACTTTACACTACTAAAACTTTTCGGCAAGAACTAACTCAAACAAACATTTTTACTCGATGGAAAATGCAATAAACGAAAAACATACATCACTCTTCACATCACACAGCTCCAGTACATCCTAACAAGACCCTGAGTCGACAAATGATTTGGCAAGAACAGTTTATCAATAACTAGGTCGGATGTCGTTATAGTAGTTTTGAGTGTCAAGATACGATTTTCTGAGCCAAAATAAACGACTTTATTGATTTGAAACATGTCTATAACACGAATTACAGTATAACTGAATATCTTTGTATTTTAAATGTACAAAAAATGTACTAAGATTAACGTCGAATTTTGAGTAAAACCACCTTTGGAATCACCCTAGTACGATTTCACAAGATTAGAACGAAACGGTCTTAGGAAATCTTCTTTTAGATCAACAAAACGAGTTCGATGAACGTTTTTTGTGGGATATGTTTAAATGAAGTAGGTGGGTATATCAACTTAATATAAGATTTAAAATTAAACTTTTGTACACTAAAATCTTAACGAAAACGGACAGTAAAATATACAAGTAAAAGGGATAACCTCTAAATATAAGAGAAAGAAAATAACTAATGGAAGAACGAGACAGAATCAGGGATTTAAAACTAAAACTACAACGACGACCGACAGAAGGGAAACAGTATACAGGTTCGTTTCAACCTCATATAGGAA

>Gm15ciRNA1668

GTACCAACTGTTGCTGAAGCCAAGCTATAATGTTCTGACTTCTAATCATCTAAATGTTCTTTCCCCAATGTTTTTTTTCCATCTGTTATGTAACTTCTCTGAAAATTTGCATGTTGCTTTCCAAGTATTATGTAAATATACAGATCAGGTTAAGATCCAGATATCTGATTTATAGATAAATATAGAACCTAATGAAGTTATTTATTTTATCTAACATAATT

>Gm15ciRNA1669

GTTTGTTCATCATCGCGTCCTCCGTCCAAAGAAAAAACGATTTTTTTTAAAGAAAATCTGTGAATTTTCAAAATGGGGTTGCTCAATTTTGATTGTAGAGTTATCACCCGTCATCAATTTTTTTTTTTTTTTTACATTTTCTTTAATTTATGTACAATTCGACATAATCTGATCTACCGCAAAACCTCTTCGAGCGTTTATGCAAAAATTAAATTATTTTGATAATTATATTGTCCCCTTCGCTTTTCTTTCTTGTGGTTGGGGTTTTGCAGATTTAACTAATGATTTGTTTTATTTTATGTTCTTGTTAGCTTTTGTCTTCTTGTTCTGATAAGATTTAGAAATTACCTTAAGATTTATATTTTATATCCCTTTTCTTTTCAATTTCCATTATGCTTTGCAACTATGAGAATCATAATTTTGATAAACTACTTGCTGGCTCGTTAAATGGGGGTGTCTTGGTGAGGGTTATTGCAATTACGAATTACTGCGTATTGATTTCTTAGTACAT

>Gm15ciRNA167

GTAAGTATATGTTCCTCCATTTTTCATGAACGTTGTGCATCTAGGCCAGGGGAGTTCTTTTTTTTTACTCACATAATCTGTTTGGCTCATGCTTATCAGGTTTTTTTCTTTATAGTTCTTGGATTATTTAAAAATTTATATTCTGACATATTAGCCTATATAACAGTTAAGATGCAAATCATATATTCTGCAAAAGTTTAATAACGAATTATGTAGTATTCAGTATTCTAAAATATTGTTTGAAGAACCTAATTGGTTTTCAATATAATAAAAAGTGTTTACTGTGTGAAAAATGATGGAAGTTTGCTGATGTGTGACATTTTATTTTCAAAAGAAAAATAAATGGGATTCCATGCATTGAGAAGTATGCTGTTCCATTTGTCTTTTCTTTCATTTTGATTCTTGCTTGTATTAAGATATTTTAATGTTATTTCTTGAAGTCACATGCATTATAAATTTGTGCCATTGTAGAAAGGAAAGCCATAGGTTATGAAATTGAAGCGTGAACTGTGTCTTGCAGAATGCTAAGTTTTCAAATAGCTGTTTTTGAAGACTG

>Gm15ciRNA1670

CATAAATACTATATAAAACGAAGGTAATTATAATACGTAATACGTTTGAGAGAAAAAATTATACTTTAATAAAACGGAAGGGGAAAATTAATAAACTGGTAATCATTGTCTGACAGACTTCAGTATATAGAGGAACAGTTAGACCAATGAAACGTAGATGTTGAAGTAACATATGTCTGGGTCACATAATCTACTGGTTAAATATGCTACTTCAACCTCCCACAGGTTCAAAAATTTGTGACTACGA

>Gm15ciRNA1671

GTACGTTGTTTTTTGGCCTCATGATTTTACCACAATTGGTTTGCTGGGGTATACAATGGGCGCACATTCTATATAAAACAGCTAGACATGCTCGGGACGATAGTACTTGAACATTGTGACATACTGGAAGAGCTGCTATGTGATTGTAAGGAGATTAACTAACCTATTTTGAGCTTATCATTAGCCTGTAAAACATAATTACTTTGAAGTACAGCAAATTTTCCTTATTTTCTTGCTCAAAATCCTTCTCTTCTCTTGTCAGTAAATGCGCATGTCTTTTGGGTTTTCTATCGTCAGTTTAATTGCTGCGTGCTAATTCTTTGGTAGATTGTTTTACT

>Gm15ciRNA1672

GTATCACAATTATAACTGTTCTGCAACTTTTTTTGACATATTATCAGGTTTGACAGCATAGAAATCAGAGTTTAGATTGTGTATAATACTATAACACTTCTTTGATGGAAACTAAATCAAGATAATTGGTGGTGAACTTCAGGTATTTCTAATTAGAACAGTGTGATGTATTCAGGGACATCCAAATTTATGTCGTTTTGATGAAATTTTATGTCCTCTAGCTGATTATCTGGTGATTTTCATCTCTAATGTTCTTAGAAATGTGAAGGGATTTGATGTTTTTTCATGGGGGTCTTCCTGAGCAACTGCACATATCTAACTACATTTACATTTTTTTTCATCGATCACTTATTTTTTCACCTAATCAAAAGATTCTGTTATCAGAAGATAATTCTAATTTTTATTCCAAAATAAAATGGCTGTTCTATTCAGTTTTTTTTAAGTATATTGCTTATATTTCAGAATTTTCTATATTCACTGCATCATCTTGGCTTAGGCCATATATCTGGTTATCTGAGGATGGTTGCTTTTCAAAGTTACTTCATTCTTTCTATTGGGAGCATTCCATAAATGTTTAGTGTGTATATAGGCTAACTGCACTCTGAAAGCATTTGAGTGTTTTTTCTAATTATGCACATAACTAAATTAACCACATACACCATGGGAAAGAGAAAATTATTGTGTGAAAGATTTTGCACATAACATTTTTATATGATA

>Gm15ciRNA1673

CATATATGAAAAAAAAAACAGGAAAAACTTCACATACGAACCCTAATAGTTAACAGGTTTAATCATAGATATCTGTAATGCATGATGTACTTAAATTAGGTGTACCATGATCATGATGGTGGGTATAAATAACTGTTAAACATTAATAACGAAATCTTCCGTTTAAGAAGTAACAAAGTACTGAACTTATAGGTCACCACAAAGGCACCTGGTACACGTTTAGATTCTTTAGACATAGAAAAGATATATTTTATACTATACGTTACTAGTAAGGAAACGTTACAACAAAACTGTTCAAACGAAATAAACATT

>Gm15ciRNA1674

CAATTAACAACAAAGAGTTCCAAAGCAGACAGCCTATATCGAAAGATTTTATATTAAATTACCTAGAATTTTTAATAAAAATTAGTTACTTTCCCGGATCTACCTCCGAAAACGATTTCTATAAACACTGAAGACGAAACGGTAATATAAAAGGTCAACTGACCAAATACATAAAATCAGGTTAACAATCCGGATATTGTCATCAAACAAATGAAACAATAACAAGGTTAACAAGACAAAAAGTGCCTTCACTGCCTTGTATACTTTTATCGGAGGTGTAACATAACCGAACTAGAAAATCGTTAAAGTACGACATTACTGTACTGGGAACAATCAAGGAAAAAAGAAAAACTTATTACTTTGGAATAGAAAACTAAT

>Gm15ciRNA1675

GTAATGCTTCTGAACAATAGCTTTCTTGTTGCATTCTTTGTTTGATTGATGCCTCAATTAGTACTTTTAGAATAAATAAAAATAAGAAAGTAAAATAAATTAAATGTCTTATACAAGTTAAAATTAAATGATACATTGAGGTTCCCTTTTGAGGTTTCAATGCCATATTATGAAATCGTGCTACCATCTTATTA

>Gm15ciRNA1676

CATGACAGAAAGTGACACGTGATGAGTTGAAATACGTACGCTTCGAAATTGGGAAAAGTTAAAAATAAAAAATAAAAACCAGAAAAACCCGTCTACCCATGGAAAAGTAAACTCCCCTTATACGTCGAACGATCAGAAAAAGTAAGTTATTTCTAATCTAAAATGACACATAGACACTTACTAAATTACTAACTAACACAAAACCCCTCAATTAAGTCAACCAACTACTTAACAATTACCATCACTTTGA

>Gm15ciRNA1677

CATGTATTAGCCAGTGTGAGATAACAATCAAGTAGATAAAGAAAATCAAGGGTTAAAAATTCGTCAAAATTTAAAGACAATTCACAACTTAAGAGTGGTGTTTACAAAAAAACTTAGACACCCATTAATTTTAATAAAAACGAAAAGTTTAACGATATACATAATCTTCTAAAAGGTTTTCCGTATATATTTTGATCCATATAATTTTCAAAGTATTGTCAATAGAACCCCTTAGGACGTCGTTCGTGATCTTCTCGTGATCAGAGATTGAAAATGGTTCTGTTATATTATCCTCTTTGAACTCTAAGAAATGTGCTCAGTCAGTTACTATTGGATTATTAGTGTTCAAACCTACTTTCAATTTCTCTCCCTTTCTTTCTCTTTAACTGAGTCTGGAACAAGCCTTCAAAAATTTATTCCCCTTCCCTCCCCCTTTACAAGGAAGAAAGACGGAGTTTTAATCCGACCCCAGTTCCGAAATAAAATTTCTCCTTTTATTGGGAGTAAGAGAGGAAACGAACGAATTTGTAAGTTTGTTCTCCTTTATTGAGAGAAGGTAAAGGAAAAGAGAGGAAAGGTAAAGAAAGGGGTAAATTTTTGAAGATTACCGGTGACAGACCGGGACCTACCTCCTCCCCTCTTTTATAAAAGAAGTAACTTATAAAAAA

>Gm15ciRNA1678

CATTCAAATCTGGAGTTGTGGATACTAACGAAAACATGAATACAAAAGTCTTTCTTACACAGCATTCAAATAATTGAATCTAAATAATTTTATAATTTTTTTTATGTTGAAATAGCTAGTGCTATGGATAAAAATGTTTTATACTCTTATATATTTGTTTGAATCCCATTTTAGGCAAAAGTTCATAGATATAAATACTACTTTAAACTAAAATCACAAGTTTAAAATTTAAACTAATTAAACTAGAGAACTGAAATTTTTTATCATTAAAGCAAGGAATATCATTTTTTAGTAACCACTACTTCCTACTTTTAATGATAAAAAGATTACAAATCTTAATTTAACTAGTTTCAATTTAATGCTTTTTATTATGTTTAAAAACGGTTTTTATGTTCCTCAGTTATGTATAAAATGTATATTCGAATATAATGAAATAAACACAGCACTTCAGTATACACCCAGTAGTTATAAAAGTTTAAATAATAATTTTCAAAATCCCGGTTACTACTATTTTTTCTGTTGATAATAAGTTCAGTATATTTAACTCACCTTGTCCTATTCTAATAACAGGGTTACATGGATTGATTCGTTATTCCTAACTGGATGTATATACAACTAAACGAAGCGTAAACAGAGAAGCTACTAACGA

>Gm15ciRNA1679

CATAAGAGAGAGAGAAAGTTAAAGACCCCACAAATCAAGACCAACGTCTTTAAATTTTGGTTTTTTTTTTCTTCTTTTTTGAAATGAAAAAACCCTAAAATTAACTACTTGTTTTCTTGGGTATCAAGGTTTATGACATTAGACCTATTTGTTAAATCTAGGTATAAACAAAGCTAGT

>Gm15ciRNA168

GTAATGCTTCTGAACAATAGCTTTCTTGTTGCATTCTTTGTTTGATTGATGCCTCAATTAGTACTTTTAGAATAAATAAAAATAAGAAAGTAAAATAAATTAAATGTCTTATACAAGTTAAAATTAAATGATACATTGAGGTTCCCTTTTGAGGTTTCAATGCCATATTATGAAATCGTGCTACCATCTTATTAATTA

>Gm15ciRNA1680

CAAGCGAAGAATAGTGAAGAGTAAAAATAAGAAGAAAGTTCAATTCAAACATCTCTAATCACTTTCGAAGTCTCTGCTTTTCTCTCCTCTCCTCACAGTCATTATAAGTAACATGTCGTTTATATTCTACCAACCTTCTCTCCTACTGACTCTTCGAACCGTAAACGTATTAACGAACGCCTTTTAAACTCTAAAACTTACAAAATGTGTTCAGTTATTCTATTACAATAATATGAAC

>Gm15ciRNA1681

CATTTTAGAGTAGGAATGACTACGACATAAAGAATACATGAAAATACTATGTACCTTGTACAAAAACAATAAAAAAACGTTTGACTTCGGAGATAACGGGAGAGTTTTTAGGAAAGAGGACGACCTTTCAATCAATGACTACTAGACTGAACATATTTACCTAACAATCACATACATAATTATTAACCATTACTTGATACAACGTAATGTACCTAACCCATACCCAAAACGTATACTATGTTAAGTCTTTATACTGTTAAAAGATTGATACCCATGTTGTTATATTATACACACACATGTGATCAGACATAGAGACAACTAGCGTATACGAATCAACAGAAAAAAGTACATAGGCACGTAATCTCGATTATTCGTTACAAACCTATATTGTATAACGACCGTACCAAGAATTAATGAAGAAATTAACACTAATGTATTAAAATCAACTTGAGAAGTCTCAAGTCTTCACACATAAACATCAT

>Gm15ciRNA1682

CAGGTAAAAGACTAGAGAGAAGTAGTTGGGTTGTAAAACAAAATCGTTTAAAGTCACCGTGTGGACTCGAAAAAGTTTAATGTCTATTATGGTGAAAAAAAGGAAGGGATGTGACTGGAAAAAATACAAATTTTAGTAATGTCATTCTAGGGAATATACAATTTGATTGAATGTGGGAGAATTACAAACTTTTGTAATGTAATACATAATGTGATTCTGAGGATGTTCCCTTTACCAATTTTTTTTCCACAAGCGCATTAAAGGATTTTAGAGTCCCTATAAATGACATTAAATCAGAGAGAGAAAGAGAGAGAGAGAGAGAGAGAGAGAGAGAGAGAGAGAGAGAGAGAGAGAGAGAGAGAGAGAGAGAGAGAGAGAGAGAGAGAGAGAGAGAGAGAGAGAGAGAGAGAGAGAGAGACAACAATAAACAAAATTTTAGAACAAACGACGAAAAGTCCAAAAGAATAAACACCTATAACGGTTGATACAAGAAAATTACAAGTTAGAACTACCAAACTTTCATGGAACAAGGGTCGTAATCAAATGAACTACCTTAAACCTTTACCAAAGAACTCACTTGATCAATCAACGAAACTGAATAAGAGTAAAACGA

>Gm15ciRNA1683

CAATAAACTAAAGGAATTTCCGTTTAATTCGTCGTATAATAAAGTACAGAAGAAGTTTTGATTTAGTAATTGGGAAAACTACAGTTAAAAGTAAATCAATAACCTGATATAACAAACTACCCGAGTCTAACAAAAGGAGAGTTGTAAAGTACTATTACACTTAGGAATATTTACGTAAGACAATCACAACTTGTAAACGAAATTTGAACTCCATCTCCAACGTTATAAACTTCCGGGAAACAACATCCTACTTCTATTAACACCCACCATTAAGAATATCAATCTTTGTGAAGGACAATACTTTCTGTTGACCAGTTTACAGA

>Gm15ciRNA1684

CATATTTGATATATAAATTACACTACGATCGGATTGAGAAAGTTATTTCAATAATCTTAAATTTTAGTTCTTATCGCACCTTGTTGTCTTTTCTTTGTTTTTACTTAATGTGACCGAGTCCTTTCGAGTATTTAGCTATACCGTGATATTTCACTTCGACTTATACTTTGGTCCATGAT

>Gm15ciRNA1685

CATTCACTTAACTCTTACCGTACTTATTTTAAAATAGAACTAAATCACACAAATCGTAAGGTATATTCCGTATAGTTACTCAAAATGCAACCGACAACTCCCAGATCGTGTTAGGAGGAGGAAATGGGCCCGAACCCTGAGTGATACTAAACCAGAAATTGGTTCAACTTACCGCAGTACACTTAACACTAGGTATATCGGCTAGAGTGGATTACACTAAACCAGAAAACAACAACAACATCAAACAAATCACAAGACAAGGTTAGTATCTATTGTCTTAAT

>Gm15ciRNA1686

CACTAAAAGACAAAACTCTTAGTAATCAAAAAATTAAAAGAAGGACTAATTGATTCGAAATCCCAAACAGATTATACTACTGTACAAATATAACTTAAAGTACAAACCCCACCCCACTATCTTCGTAATCGGATAGAAAAAAAGGTCAAACCTACTTACAGAAAGATCATTACACAACTTCAATTCCAACTTAGCAAGCCCATCCTAGGCTCAAACTAGGAACCACCTTTGTTAATAACTGGTCTGAACTAAAATGAAGGACTGGCTTAAGGTCTAATCAGCCACAGGGAAAAAGTATTACTTGACTTCCCAATTCTTTTATTTTTATTATAGAAATAGATGTTACTGAATTCATCCGGAAAAAAAAGGTGTTCCTTCGTACCAGTGAAGGTTTCCAAAACATTATAAAAGCTTGTAACTAAACAAAAAAAATGGTTTAAAAACACAATCGAAAAAAAAAATAAACAAAATCTTGTAACTTA

>Gm15ciRNA1687

CAGAAAGGTAGAGAGAGAAAAAGAGAGGTGACTTTACAAACGTGTACGTTTGCACCGTACCCGTCCAAAGTACTTCTATAAAGTGTCTTTTGTACTGACTGATTACTATGTGAAATTGTACCCTCTTTATAAAACATGACAAATTTTTACTAACCATATGTCCGTATCGATCTTTTAAAATATAAATAGGTAAACACATACGTTTCTACGTGTACTCATCAGGTTCCTCTTTAAGACATGACTACCCTCTCATACATTTTCGAAGTAAGAAGAGGTACCAAGAT

>Gm15ciRNA1688

GTGAGTCACCGAGTGAGTGTGTGTGCAAGTGTCTGTATGTGTTTCGATTGTTAAATGTAGGTACAAAACTTAGATACAATTCCTTAGGTTAGGTGTTGTTTGGACTGTTCTCTGGTCAGAATTCGAGTTTCACCTTGCTAATATGCATAATTGTTTGTTGCAAATCTTTATAGTGCAAATTATTGTGGTGAAACTCCAATCAGAATTGGAGAACAGCACAAACAACACCTATTGAAATGTATCCAAGTTTTGTCCTATTTGTTTGGGTGTGTTCGTGTTTTAGTTATTGA

>Gm15ciRNA1689

CATTTAGTTCTATAGAATTTTAAACATTGGAATCACATTTAATACGTATACGTACGAAAACAAGCTCTGTTTAATAATGTACTGTAAATTCATGTCACCTCAAACATTTTTATAGAGTCACTGTATTTAAACACAAAGATTTCCTATGAACATCTAAAGGTAATATCAAGGAGAAAATAAAACAGATTCTGTAATATTAGTTTATAACAGTGACATATACAACAGATCATGAGATCACACGTAATCCATCACGGTTGACCAGGAAGGGAACGTTAATACCAGGTTGTATCATCCCGTACGAAGTAAAGAATAGTGAAAACATAAGAAAAACTTTCTCAATGACTTCGAAGTCGACGTTCTGA

>Gm15ciRNA169

GTGCTACCGATTTTACATTCATCTGGGATGTTAATAACATTTGCTAGCACACTTTCAATGTCCTTTTTCATTAATGGGTGAAATTCTTACGAGTTTTCCTCAAGGTTTTAAATTACATTTAGTGACGGTTATACAGAACTCCAAAATCTTAATATCCCAGAAAATTGTAGGCAAATGCAGTTGCAGACTGTAATTTAAAACTGTCACCCCCAGTAAATGACGTGGGCCCCACTTAAGTTACTGGGTTTCATCTCTTATCTTGTGGGTCCTATGTAAATTCCACTCAATAAGGGTGTTGGAAACAATTTGTAGATAGTGTATTGGTAACATTTATGGAGGATTTGCTTTCATTTTCTTAGCAGTTTTTGTC

>Gm15ciRNA1690

GTAGGTCTTTTCGTCAATTTCCCATGTATCATGCTTGATTTGCACTGACGTCACTCACAGATATTAATATCCCCAATAATGTATGGAATGCATAATTTTTAAAATGTTTAATCCATGCTACAATTTCAAAGTGCTTTTACAAGTAATGACAAGTTGTCCACTATAAATATGGATGCTCCATGAATGAGAAACAAGCGCCCTATTAATTTGTTGCATTGCATTGTCTTTTTTGTTTGGAATCTTTTTATGGTTGCAATTTTCCAAAATACGTTGAAAGACTGCCATTTTATTTCCAAACCAAAATAAAGGAATGCAATTGTGTGGTGCTTTTGCACAACCAATTGAATTTGTTATTATGAACTTTCAAGTGGTTTAAAGACCACAGACATCAGACTCATCT

>Gm15ciRNA1691

CATAAAGAAAGTAGTTCGTTAGAGACGAGAACTCAGGGTTGCAACTTGATGGACAATTAATTAACAAAAGAAACATCCCGAAACTAAATCCAGAGACCTAACTTTAACTTAAAATAACGACAAAAGAAATTAAGAAACACAAACAAATTGACACCCCTAAATAAAGTTTTATTTTAAAGAAAACTTGAATAGTTAAAAAAGAATGGAGAGAACAT

>Gm15ciRNA1692

CAAGCAAGTTAAAAGAAAAGAAAAAAGACCCATAAAAGACAAGACAAAACAAAGGGCCCTTATTTTTATTTTTTTATCAAATAAAAACAGGGTGTTGCTTTTGAGCGCTTAGGACCCTTAGCCTTTAGCCTTTAGGATTACAAAAAAAATTACTTTTTGATTGCTAACTTAGCTTAGTAACTTAAAACATCACAAAGAAAACAAAAAGGAAAAAAGTATAAAAACAAAACAAACAACAGCTCCAAACACACAATACTAAAAAAAAAGAAAAATGGGGTCACCCCACAAGACAAAAATTAAAAAAATAAAACTAATAAATATTTACTTTATCTAAATACCCAAGTAACCCCATGGGATAAACCCTGAAAACGCTAAACGAAAGTACCAAACGGTAACATACAACGCTTTCACATCTTATTTCTCTTAACAAAAAAAAAAAATTTATTTCACAATTGTAAGTTTCAAAAAACTAGCCACTAACAATTTGAAAACGGAAAATTACATTGTCGCCCGGACGTTAATGAGTTAAAAGCAAGGAAACATGGAAACATAAATAAAGAGTATTACTTCTCAAATATCCTAAGGCCAAAGACCATAGGTTCGAAATAGTCTATACCTACGATACTAGAGTAAAGACTCAAAGAGAGTTACCTAACTACAGGAAGTATTGGTTAAAATAAGAAAACATAGTGAGGGTTACTTGAACAAAACAAAAAACATAGAACATAGTTAAGTAAGTGGACTCAATTAAAACAATCAATAACTAGAGTATTAACCAAAATGTTTAGGGGTACAAATTATTACTAAGACTAGTATTGTTCTTATTAAAAGAGTACCATTTTACACAGAACGAAAGGAACCAAAAGTAATTTAAACCAAAATCATATTAACCATCAAAATATGATAGTGATTATAAATGGCCTCTGTTAGAATTAGTATAAGCTCGCACTACAAGATTTAAAATATTTTCCCTCTGGCATAAATGGTTAATATTTATTTTGTCCTGATAGTTTTGTAATATGGTTAAAAAAAAGTAATTTAATACTACCTACACAACAC

>Gm15ciRNA1693

GTTGGTAATAGAACTCTAGCATGACACTATTTATGAATTTAGGTCCTCTTTGGATAAATTTCTCAGTAAGTATTTGAGAAAAAAACAATATGGTAAATTGAATTCAGTTTCTCCCATAAGTTAAAATTAGCTTATGCATAAGTTAATTTATAGAAGTCCTCTCGTTTAACTTCTCCAAAAGCCGATTTTAACTTTTGTATAAGCTAATTTTAACTTATGGAAGAAATTTAATTTGCTTTACCCTTTTATTCTCTTCTCCTACAAGTATTTATTGAGAAATTTTACCCATAGTTTTTGTGCCATTTTCCCCCAA

>Gm15ciRNA1694

GTCTTTTCTTTATGGTTTGCTTTTATGTAACTTCTTTCCTTCAATTTTCCTTTTTGTTTTTATGCCTGAGCAGATTACTAGTGGTACTGGATTCCTTCACTGACTGACTGTTTTAAATGACTCATTTATGAATGGATCATAGTTGGGGCATATTATATATTATATAACGTAAATGGCTTGGACATTTTCAAGTGGGCTGGTCTGATTTGCTGTTTTCTGAAATGGCAAAATGTGTCTGAACATTTATTGCTCTTATTGTGGACTAGACTTTTTCTTGCTTTATCTGGAGTGAAAACTTGAATGTTCCTTTCA

>Gm15ciRNA1695

CATTCAGAGAACTCGACTTAACACCAAACCAGTAAGACAACTACGATAACAACCCAGAAAAAGATACACCTGAAACCAACCGAACAAAGTTTAGACCAGTTAAATCCGGTACGTACACATGTATATGAACTTAAACTATGATACTATTAATACGGAAGTTTATGAATAAAAGTAAATAGTGGTAAAGGTGAATTCTAAAACTCTTTATTCGTCAGAAAGGTTCTTCGTATAATAACATTCTTTCCCACCTTACCTTTTATTATTTTACATAGACATAATCGTTGAACTAAAAAATTAAAAAAAATAAAAAGACCCCAAGGTAAACCCTAAGTTTGAACTTGGTCACATGTACAACGCCAAACTATACAAAAGACGAACTTACATTAGTGGTCAATTTGTTAAAGTGTATATTTTTTAGTTTACTAATAATAAAATTTGATTAGGTATGTACTGTAGAAAAAGATTTGATTCGGTCATTGGCACCAAAGGACTTAGTCAACTTAAAATGATACATGAGAGAAACGTAAGAATCGAAATTCAGTTCGTTCGTATATTAACCGGAAAAATTGTAAGAAACTATATATTATGTACAGAGT

>Gm15ciRNA1696

CATGTAAAAAATAAATAAATAAAGGTAAAATATATAATAGAGAACTATAACCTGTAAGACCGTACTTTTATACGGGTCGTGAATTGTTTGTGATGGTATTACGTGGTAATTATATAATTGTGGTGACAAAACACGTCTGGTGTGATCAATTAATATTTAGTTCTCTTAGAGACTGTGATTGTAAAGTAATATGCACTTACGACGTAATAAAAAATCCCAGGTACTTAAATAATTATACCTTGTTACGGTAGACT

>Gm15ciRNA1697

CATTGACGTAACGGGGAAAGAGAGAGATCTCAAGAAAGGATAAAGTTAGACAACGTAAACAAAAGTACAAGTAAAAAAGGACACACTAAGACAACGTAACGTGCACCAGTACGATTTTTAGACAAAAACTAACCTTAAGACTCCCGGGGTCTTCGAGT

>Gm15ciRNA1698

GTGACTTCTGATTTCATTTGTTAAGAATTTCTGCAGTAATTTTCCTTGCTTAATATTTTCAATCCGTTTAAGATCATTCCCCTACCTTCACTAGTAAATGCCACAATTATGAGAGTTGAAAAAGCTTGTTTAATGACTTCTAATTTAGTATATTAATGGACCAGCGAAAAACCAAAGAACAAACACTCTTCCTATTTTAGAGTAGCATTTTTGGGATACATTTCTTTGCAAATTTGAAG

>Gm15ciRNA1699

GTTTCCTCATTTCCTCTAGATATTTGTCAAAGAACTGCTTGACGTAAAAGCTTACGTTGTTGTATCAAGATAGAGCACAAAAATGGTTGCTATAACTGTAGCATGCCACCATGCAAGAGTCCTTGGTGGTTGAAATGTAGTAAAAGTTTATTGTACTGAAACCATGACCAAATATATAAAATTTGGTGGGGACATAACCCCATTACCAAAAGCTTGGTCCATCCTTGCT

>Gm06ciRNA17

CATTTAATAATTGGCTTTGTAAGGAAAGTAATTAGCGTTTGGTTTTTGGCGATTAATTTAAGTAGTATAGAACAAGGCTAAAAATTAAAGAAAAAGTTTCTTTTATTTATTTATTAAGTGACTTTTAGCAAAATTAAACCTGCTCTCTTAAAAAGTACACACCAACAAAAGTTAAAAGTTAATTACTCCAAGCAGGTTTTGCGCAGCAAAAGTAATCCGACCGCTCTTTCGCTTTCTTTTATATACACTAGGAGTCTCTTAGAAACAACTCTTACCTTTCTATAAAAATGGTAAAAAAAAACAAAAAATGCCCTTAATATCCTCAGGCACGAAAGACTTTTTGTATTTTCAAAACAGCTACATTTGCTACTAAACAAATTCTCGGAGTGTTTAAGACTTATCTACCTAACAAGCATGAACACTGGACGAAACAAAAGATCAAAGCAGGGACTAAAATACCAAGTTACTACAACACAACAACAAAAATGAACATTGACACTGGCACAAGGTCATGAGCCAAACAATGA

>Gm15ciRNA170

GTTTGTGATAATAGTAATACTACTTTTATTATTATCAGCACTTGCACATGTTAGGACTTAATAATGGTGGATAACTAATTTCTTGGGACAGTATCTAATTTGTGGTGAGATGATATCATTACATTGAAATTTTTGTTAAGGGAACATAGTATATCTGTTACTATTTTTCTAAAATATCTTCCACAACTGAAATTTTTTATTATGTTGGAGAGTCTCGCATCATCTACTTCAATACTTCAGATGATACTTATATACCTTGTAGGCAACTTTAACTAATGCTAATTAGTTTTAAGATGAAATCTAACATAATCTTAAAACTACTAGCTCACCATTGTGCATTGCCTATTCTTCTATTCTAAGCCAATTGATTTTAGGATGAAATGTAACACATTGGATTTAGTCTTTCAGATGTAATGTCTTTATTTCCAATTTTAGTTGAGTTCTCTTGCAGAAAATGAGGACCCTCTAATCT

>Gm15ciRNA1700

GTACTGTTCATAGACCATTCCTGGAATTAACAAAAGTCAATAGATTGAAATTTGATGAATGTCCTGCACATTATTATATTGTAAAGCCTAGTTTTGAAAGCTCAATACCAAGTAAAAAACCAAAATTCATATAGACACACGAGTGACAGACAATTTCTATGACAAAAGAATCAGGCACATCTTGAGGCATCTTTCCTGTTGGAATGGAAAAAATGATTTTTCCTCTATTTTTGTAATTAGATATTGTGTAAAGTAATGAAACAAATTCTAGATGGATTGATTCTTCTCAAATATATCCTGCACTATGATATATTCTTTACAGTTGCAACTTGCAACAGTTATCAATTTGTTTA

>Gm15ciRNA1701

GTGAGAATTCACATTATTATATTCTGTATCATTAATATTTGGTGTATTGTTTGTTCAGGGAATGCTCCTGAGTGTTGATTGCCTGCACATGTAATAGGTGAAAAGATGTTTTGCTTTGAACACCATTAATGAGTTTTCAGCATTACTTGAAAAAATAACAAATAAATAAAAAGTAAAATAAAACCAAGATTCACCTATATAGAAGTTTTAACTGCATTACAAAAATAAATTGAGAAACTTTTACTATTGTGACCTTGTGTTTGGTTTAGTTAAATATTATTTTTTGAAAAAATAGAGTATTAAAAGCTCATATCCACATAATATGCCATCTTTTCTTTTAACTTGTGAGGTCATTCTTGTTTTTAATCATGACTAGACATATTTATGTGCATCTTTTCTTACTGCATAATGGTTAACAAAATATTGCTGATGTACAAGCAACAGATGAAGAGTCATGATCTCCACACTCCTTTTCTGTTACACCCCTCA

>Gm15ciRNA1702

GTGTGTGATGTTTGTGATGAGATTAACTTCAATTCATTTAGTTTTTGCCTGATGAGCTTTGTTGGTGGCAAGTTATTTATTTTTTGCGTTTATAATAGTTCATCAAGTTAATCTGATTGCTCTAGTATATGTTCTTATTTTAATCTGGTTGCCAAAAGCTCCCTCTTATGTTTGTTGGCTGTTCTTCAAAATGCCTAGTTCTGTTTTGTAGAACATAATAATGTCTAGGAAACAATTCTTCCTTCTGCTCCCACCAAACTGATACTTGATATTGCCAAGATAGTTTTTCTTTGTGAAAATTCTAGTGATTATGTCTTTGAGTGTGCTTTAGAATGTATTTCTATGGAGTTTTAGATGTGCATTGATGAAGGTAGTTACTAATTTCGAAATGTTACTCATGTGCTATGATGTGATTTGGATTTGTACCTTAACCGAAATGTGGTATTTCTTCCAGTATT

>Gm15ciRNA1703

GTAATTTCCTGATCACTTACAAAATCTTATGACATCTTTTGCTTTTCCTATCCAATTAATCCGTGATCTAATCACAACTAAAGCGCAGAGAGTTTTAACTTTGGGTTACAATTATATGCTGCCTAGTTGATGAGGGACGATCCATTTCCTTTCCTCCTTCTCTATATTGACCGGCGCAGCTGATGCCAAGCCATATAGCATATATTTCACAACAAGAAAAAGAAAAACCTCAGCTGAGTGAAATAGTGAATGGCTATCTCTTTTTCT

>Gm14ciRNA1704

GTAATCCTTCATAATAATCAAACCGCTTTTACTTTATTTACTTCTGCATATCCTTTTGTTACTTAATGTACTCCACTAGCTATGAGTAGCCCCTGTCACCAGAAGAAGATATTTTATGCACTCAACTTTTTGGTTGTGTCACCCTTCTCCAGCCCTTCCAAGCAATTATTTGGAACAAGAATTAGAATTGTGAAACTCAAATAATAACCGTCGTATCCATGCAATTTCCCTTCCCTTTTAGTTTTAGGATGAAGGCAAAGCGTAGCCGAATGAGCATACTGCTTTTTACTTAGATCACATTGACTTCTTTACTAAACAGAATTCTAAGATCTCAAATCTGGAAATATCCCCATGTCACCGTATGGGTAGCATTATGCACAGAAAAAAGTAGTTGAATTGCTGAATTGAGGGAGTATCTGAATAGTGTAGCTGGAAGTTTCATCATGTGCAAATTGTGTAATATATTATGGTATTGTAATAAAATGAAGGCATTAGTTTGTGCCCCCAGCCCCCTCCTTTCATTTTGTTACTTGCTTCATCAAACTGTTTTATCGTACACTTATAAATTGCTATTAAACTA

>Gm14ciRNA1705

CATTGAGTTGTAATGGGAAGGGTGAGGTGATCAAGTTGAATCATCAATGACGAAAATTATATTATGAGCGAACTGTTGTATTTTATTATGATCATTAAGGTGTTTTGAATCTACGTTTTGGAAAACAAAAACCATAACACAAGATGAAGAAGGTAGAGTAATATTAACAGGGCCTTCTCTTTTTTTTTTTTTCTGAAACAAAGTATTATTAATAGTAAAATCAAAAAGTAAATAAATAATAAAATAGAACCATATAGATTTTATTAGAAATCTGCTAATAATAAAACCCTACCTCCCTTATTAATTTTAACCTCAAAATTGATTGATTAAGTGCATTGATTTTCAAGCGCAATTTACAATTACTAATAGTGACTCCATTTTGAGGTTTTTCGTAGATATCTTGATATAGATACAAAACGTGAATTGTTAAGAGTCGTCCTACCCCGATTGATCTTTCGGTCACTAATACGTTCGTATTGTCTAATTATTATCAATAACAGAACAGAACATTTCTATTTTTTTTAGTTGATTCATTGTCTATACTTCGATCCAAAATTCTTAAACAGAC

>Gm14ciRNA1706

GTGTGTTCCCCGAGCTTTATTCTGTACACACCTATTCTGTATTACTGTTATTATGTTCCCTTAAATAAGTTGTGCTTTTGTTTCTTCACGTGAACTTTTGTTTCCCTGAAGTGTTGCTGTTATATGAATAGGAAAACTTGAGCTCGTCATTTAGATGGTTCTATATTTCTTGTTTTGTGATTTTATGAAGTGCTCTTTGTTATGGATAAGCATGGACATCTTTTTAATTTTACTCAACTGAAAATAGTATGTAAAGGTCCCATGGGGAAAACACGGTTTCCATTAGTATTAAGTGCAACAAATGCTAAAATTAATGGTCATTTTCAAATAAACCATAGGGGCTTCCCAATCATAGCCATTATATGCCCCTACTTTTGCTGCAGAAAAAATTTGCAAATGGATGAGAATGTGGGATATAGCATGTTTGTTGCCACCACCTTTGGAGGGTAGGGGGTAGAAAAGGCAAGTGAGCAGTGGCAAGAACTTGTTGTATTGTAGATGTTGAAGAAGATCATTGCCGACAAATTAACATTGTATGCACTTCTTGGGGGGTCGGGGTATTCAACTCCCAATTAGTTATATTTATGCATGCCTTTGACCTCTGTTATTAA

>Gm14ciRNA1707

GTGACCTTTCTTCACAGATTTATCTAATCTGCATTTCTGATCATTTTGCTTTGTTTTTGCTCATGAAAAATGGAATCTTTCTGGTGATTTAGCTCAATTATGCATTATTCAATCAGCTATGCATTTAGATTGGTTCTGGGACTGGAACTTTTCCCATCACTGAATAATCTAGAACCTAGATTGTGTGTATTGGAATTTCTAATTTTTTTTGTTATATTGTTTTTTTGGTTTGGCATGAGAATTTTGATTCAATAAGAGTGGTTGGATTGGATTTGTTTGATGAAATGAGTGATTA

>Gm14ciRNA1708

GTAATCCGTTGCTACTATTTCTTTGGCTATGATGATCACTCCCCTCGGTTTCTCTACCTCGTTGCATTTCTTCTGTAACACACCCTCTATACTTTTTAGCCCCCAAAATGAGCTTATGGTGAAGGTGGAATATGGAGACCAAGAATTCCATTAGCTGGAAAAGGGCTTTGAGTTTGTAAATTTATCTGTCCTTAGTTTTCTTAAAGGCTCTTTCTTTCTGTATCTTATATATTGACATCACTCATAATATGATGAGGACATCTATCAAAAAGAGCCTTGGGGAAGGTCTTTATTTCAATGTTCAACTTGATTCCAACCATTGCTTGA

>Gm14ciRNA1709

GTGAGTAAATGAATCTTCTTTTCATTTTCAATTGATTCTAGAATTTTTGGAGGATAAAGGAACACATGGTGACTCTTTTAACACATTTGCAGTTGACCAAACCTGTTTAGCTTATGATACTAGATTAGTGTTGGCAACCAGAATATGATCCTTGTTCATTCATTCAATCATGATTATCAATTTATCATCATCATTCATTGGCATTGTAGTGGTCCTGGTGGATAGGATTCCTTTGGATCTTAGTACATGTTTTCCATCTTTATTACATGGAAAAAGTGTTGTATACGTTCTAA

>Gm15ciRNA171

CATACTTATACAAAATTTGTTATAAAGGAAAGATTTAGTACGTATTCACGACCCCGATAAATAAGAAAGGAAGTAATTATACTAGGATACACAGTCCAATATGAAATCTTTTGACGATAAATTTGTCGCTAATGGGTTCAATGTTTGTGGATAATTTACTCTTACTAAATAGAACTTTTGATTTCTATGTCCCCGACGATTGGAATCAACCTTCATTAATACCAATCAGTTCCCAAGAAAAGAAAAGTAGTCTCAATCTATATGTACCTATCATTCAATATTATCTACCGTATGCCTCTACAGATGTAACCAAAGTGGTAGTCAGTCAGTAAAGGAATCACATTTTCGAGATTTAAATATACTCGAGACTGTTTATAATTGGTATCGTTCTATTCTAAGAGAAATTACTTTCTTTATTACTTGTTATTACGAGACTGAATAGACACATTCACCCACTACTAACCAGGGATATACTGCTGTATACGTTATAAACATTTACTTCTGAAACGTTAAATTTACTTATCAGTAACTTGTATTTTTAAATGTAGACAAATGTGAAGGATGACAATGACACTTGAACCGACGTGTTACTCTCACATAAATCTACTGTTCCCTTACGAAATTCATT

>Gm14ciRNA1710

CATGAACAACGAAAGAGTGAGAAAACTAGGCACCTCTTTGGGGGAGAAAGAGACAATGAGATAAATAACCGAAAATATACTAGCTAAATTTATCAAAGGATTCTCAAGTCCCTCTGTGAAGTACGCAACAAACTGACGGTCTCTCTGTCTCTCCCTTATTGACTAACTGCTTAACCAGTATTTCTCAAGATCCAAAAGGACTGTTTTTCACTAAAGAACAATCTAACGTAAAACCTCTCCAATACACAAAAGTTGCAGAAATAACTAACTAGCGTAGTAACAAAAAGAGAGAGAAAGGAACGTTGACTCATCTCCCAGATCAAGTAAATAACAGGGGGGGGGACCAAAAGTCGACTAAATTCCGTACTATGAA

>Gm14ciRNA1711

GTATTTTCTCCGAAGATTTTTCTAATGCTTTACTGCGTGTTCTTCTTGATTGCTGTGTGCCTCTATGGTTTTGTTTTGTTTTATCGTTGAATATCTATTAGTGATGATGCCTCTGCCAGTTCTATAGTGCTGCTACTGCGTGTGATTGACTTCCCTCATGATAATGGTCACTTGGTCAGGAATTGTATGCACTAGTAACAAGATTTGTGGATCAATAATCGTTTGTCTCAGTATACATGGGAACTGATATTTAGGTGCAATTATGACAGTTATGTGTAGAAAATAACTGATAGTAGTTATGTATTAAGAGTATATATCGGACAGTAGGTAGTGTCAAGATATTTTCCCTTTGTTGAAAGAGAATACTGTGACTTCTGTCTTTGGGAAAGCAAGGGTGTTGCTGTATTTGTGTAACAGTGGACTTTTGTCTCTGGGGCAGCAAGTGTATTGCTGTGTTGTGTGTGATTTCAGTGTTCAATATTATAATTTATTACTTTATCAATTGGTGTTCTATTGCCTTCTTAGGCACCAGAGTAATACAAATAGAATTCATAAGCTATTTTTGGTTCAGCTTTATTGAATTGCACCCTCAAAGTTCTCTCTTTAGCAGCTAAAAAATTTCACACTAAAACAATTTGAACTGAACACGCACACTTCAAGCTTTCCATAGCAATGAAATTCACTAAGCACTTTCAGGAGACCTTCATTAGTAACATTCCAGAAATTTGAAATCACTGGGCTGCCATGGAAGTTATTTTTTCTCCTTATTCTTGCTTCCAACAGAGGGCGAGCCCTGGTGCAGCGGTAAAGTTGTGCCTTGGTGACTTGTTGGTCATGGGTTCGAATCCGGAAACAGCCTCTTTGCATATGCAAGGGTAAGGCTGCGTACAACATCCCTCCCCCATACCTTCGCATAGCGAAGAGCCTCTGGGCAATGGGGTACGAAGTTTTTATTCTTGCTTCCAACAGCTCTTTTGATTTTGAATTCTACCATATGATTTTTGTATTTTGTCATAATACTTTATGCAACCTAAGCATTTCAAAGAAATTAGTCTG

>Gm14ciRNA1712

GTATTCATGTAAATTGATTATATTAATTGTTGGAAAGTCGCCTTAATTGCAATCACCCCTGGCCCACCTTAGTTGTGTTGGGTAGCCTGGTAAATGAGGTATGCTAGAGATTTCACATATGAATAGTGATATGACCAAAATAGAATATATGAGGGACAACCTGTTGAGTTAGGACCAAATTCGTATTCTAAGACTACGATTGTGAGATAATAAGATCCAATGTGGCTTTTTTCGTTGTAATGTGTTTA

>Gm14ciRNA1713

GTAAGTTCAGCTTCTACTTGATTGAGGCCAATGAGTTTAATAGACATGTGGTTAGTTTAAAAGTTTTTACACAGTCAATCATGAAAAATAATCCAAGATACAACTTTTAAAATAATTATTATAAAACTCAATAGACTTATCATACATGATAGTTTGTGATTGTATGGCAATGCAAAAAATCAGAACATGTATTGTTTATTCTTAAACAATTTTGTTTGTATTGTCCTCCCTAAAATCCTAAGAAGGTAATTTTATAGTTCATATTGGTTACCATGTATGGTTTTTTAAGTATTTAGATTCCT

>Gm14ciRNA1714

CATAATAAACTAATAACTACTGAAACAAAGAACCGAAGAACCCTGTTATAGAAAAAAAGGAAGTAAATACAATTAATAAAAATTTGTGACGGGAACCTAACAATAAAATACAGTCTCGAAGTTAATCTGTCACAGAATTTTACTGAATTATATATACATCTAAGAAGTTAGACTAAAAGAGAAAGGCTAATAAGAAATGTTGTAACATTAAACAAACCTGTTCCGAGGAGATTTCTCGAATAGAAGTAAATTAAATTTCATTTTGAGTTAAATTGAATTATTTTAATTTCCTACTTATGTGGGAACGAATGAAATCTCTGTAAGAGTAAAATCTCCTCGAAATAGGGTAAACAAAGTGGGTCTTAAAGTGACATATAAGTACACCTAGTTTAACGATGATGTCCTTTTGTATTGACTTACCTAATAATGTATACTAACTGGTCATACAAAACAAGATATTTTTTACTAAAATTTACGGGACTAGACTATAGAAAAGGAAATGACAGAAGAAGTACAATACGTTAAAATTTTTATAACATAATCTTGACATAGACTACTCGAAAAAGAAGTGTTCGTAAGAGTGAACAATACAAAGAA

>Gm14ciRNA1715

GTAAGTATTGACAATTTCATTTCTACTCTAAGTAAACACCTGGGAAATGGTAATTAAAATACTCAATTTCTTCCTAATCCTAACAGCAGCTCCAATGACAATTCTAAATCCCTTGTATTCGAGTCAGTCACATAAAATCTGACATGGCCATGCGGTTTTAACAAATTTTAAAAGCAACCAAGGTACTAACAGGATTTTTTTGAGATACTAAAATATTTATACAAAATATAGTGGTGTCAGCCAAATTACTCATCATATGCCACCAATGTCCTTGCAATCATGTTGTTTATTGCAATTTTAATAATAGTTTGAGTAAAAGAACAAGAACGGTTGAATTTCGTTAGATAAGGGGATTACTCAGGTTATGACAACTATCTAACATTTGTTACATGTTGATCATATTTAGCAAATTTTCCAACACTTAAACCAACAATCAACAGTTGTTTCACATTATTTTACTCTTTATCAGACTCA

>Gm14ciRNA1716

CATACAAACGTCGAACAGTTAAAAATCTTATTGACGGACAAAGTACAATACGTTGGTTTTATACAATGTCATACTCCCCCATTATTATACGATTTCAAGAATAACAAATAACTCGAACTGGTTAAGACTTAGACTATGTAAATCATTTGAGAGAACTTCAGATACATCGACCTATAACATTATATATACATATAGGTCTCCAAGCTTACCTTTCAATCCACAATTGACTAAGAGTCGATAAGTACAAGATTCTTACATTTACAATTGGGAATAAAGATTGTAAGAGGTCACCGAACACTTATCTGATACCAATTTACACTCGAACTTAAATGGATTGGTTTAATCAACGTCTTTGTCAAGGACAGGCTTGAGACCTAATCAGTCCAGAAAAGTAATGGGACTACATGACCTCCCAATTCTGTTTTTTATTATCAACGTCTTCGAAGGTGGTCCGAGAGAGGGGGCGGTTTTTGTGGAAAACAGACAGGGTTATACCAAGATATTAATATAACTAAATCGAGCTCCTTA

>Gm14ciRNA1717

CACTCAATAACAAGAGGAAGCTAAAAGATAAGGGCGCTTGAAAACAAATAAGTCTAGTTAAGTGACAATATCGAGACCTTCGTTCTTGATCTTGTGAACGAACACTACTAATAACTACGAAACGACATATTAGCCAGTTAAAGCCAGAGGTTCAGTTCATACGACGTTTAAAATGAAATGATCAAACGAATGACACTTCATACGCGTCTAGACTAAAAAAAAAGCAAGCGAAAGACTTGACCTCTCGCTCTTCGTATTAACAAGCAACAACGACTACATAAGAAAACGATCTATCACTAAACT

>Gm14ciRNA1718

CAAGGAAAACTCGAAATAATCACGGAATCAAGTAAAATAAAGAATTATGTTTATAAACTTTTGTCTTTTATTTTACTTTTATCTTATACGAAAGTGATTGGTTTGTTCATTTATCAAATGACTAACAATATAAATTTGAATTTTCAATTTTAGTTAATTATAATTCATTTCAACAAGTTGTCTATATATTTAATGTAGTACTCTTAACTCTGAACTCTGCCCCTTTACCTGAAAGTGAAGCGTTGTGGTTATTGATAAATTTGTAACAGATGTACAATATCATATTGTACAATATGATAGAAAATCGAGT

>Gm14ciRNA1719

CATTCAACTACTCAACAAATGTGTCTTAATACATTAGAAAGTACTGGGGTGTCGATTCCAAAACAGAGTAAGTCCTAAACGTAAAACAGATTAACTAAGTAATTTGTTGTAAAATTTTATCCAAAGTAAAGATACTGACAGTTACATTTTTTGAAATGTGGTAGTTGATCAATTTTTAGTAGTAACTATACTGAAAAATCCAACAATACATTTTCAGTTGTTCGAATAGTATATATTAACAAATATTAACATATTGTAATATTTTAAAATGTAATACTTAAGTATCTGATAAATGAGAAACTTATAATGTTAATTGTTGTGTCGGGAAATAGTATTCCACCCCAACCGTTGTACTTAGTTTGTTACGCTAACATAAGACGACATTTTAGTATAGTTCTAAACTTTTAAGATAGGGGAAAAGTAAAT

>Gm14ciRNA172

CATTAATGGTGAAGTAGGAATTGAAGATTAAATATAAACGTTAGTTGAGTATTATATTTCGTTCATTATAACTCACGTACTAACCAAAGTGCAACTCAGTAGGTTTTAGTGTAATTTTATCATAGTTAATTGTACGTAAAACTCATTTACAAGTGCACAAGCTAGACCGCAATTTCTTAACTATCGTACAATACTAGATCAACCAAATTTTCAACGTAGTTAAAGTGTGTGTGAATTGTATTTTCGACTTTATCTATCAAAGGTTTATAGTACCCGTACAAGAAACACGCTCAAAAGAGAGCTCCTTACAGTTTAGTTTGCGATGTACTAAGATACAGATCTTTGCTAAAATCAATTTCATTAGAATAAAGCTTTAAAATATGTTTTAAAATAATGATTGAAACAAAATTCTTATTTTCGTGGGTTTGAATTTAACTAGCTGATTAGTTAATATAAATCTTAGTTAAATTAAACTTTAGTTAAAAAGTTTGCAACCGGATTAGTGTTTGA

>Gm14ciRNA1720

CATACTTGTCGATATATATGAATTACGTGTATACGTTCCGTACGTGCATACGTCAGAAAGAAAACCAAGGTAACCTACCTCATTTGTTAAATGTACAGAGTTTTAAAGCGTTAGTAGGGACTCCTCTTATTTATAAAAACTGTTTACTTTTAACGTTGTAACCACATCGAAATAAAGAAGGTTATACTGGTTATAACTGACTACTGATATTAGACCATACACTCCAAGTAGAGACGATAAAATGTCGA

>Gm14ciRNA1721

CACTCAGTCTTAGCGTAAACGAAGACAAATAACTTAGCTTCAACCAATGACCTACAACTTCGGAGGCCTCAATCCTTCCAAAAATTAATACGTTTACGATTTGTCACCGGAATCCCGTAACCAATTCTTTGATTTTCTTCTTTACAAAAATAAACCTTCGAGTTTTAACACGACGGGTATTGAAAAAAATACAAGAGAACACTATAGGTGTTATATATAAAAGAGAAAAAAAAAGGGAATTGTTTACAGGATTCCCGTGGCTAAATGTTCTGGGAAATTTAATGACAGCGGCAAAACAACGCAAGGAA

>Gm14ciRNA1722

CATACATTTAAGAAGTAGAAATAGGAGGGAAACAAGTTAAAACCAAGAAAACAAAAAACAATGTACACAAGTTACGAGACCTTTTAGAATTGAGAATCCCTTTACTGTGATCGATCCAAACAACCACCATGGGAAAACCAATAAAAATCAGTTTCAACAAAGAAAACACAACATACTACTAAGTTGGTGACCTTTCAAATCTAAAAACTTCAGAAAACACACAAAAAGGAGTTGTACAGAATACCCCATCAACACCCCAACTGTATCATTCAATTCAGGATCTAAAACACACACTACAAAAAAA

>Gm14ciRNA1723

GTAAGTTTCTTAATTAATTTCTTTCATAAACTAGGAAGTGTGCCTATTGCATAGAAGAAGTAGAATGGGAAGATTCAAAATTACTGAAGCATAATTATCTAACAACGAACAATTCATTGTGAATTTTTCATATTTCATGTTTGAGGGGGTTTTCACCACTTAAACGTGAGTTCAGAGCAGCAGCTAAATTGGATTGTGCAAATGCATCAACTCAAAATCAAGTTTTGTTCTACAAGCAAGTTTGATTTGAAGTTAGTAAATAAATTCTCTGTTTAATTTTATCACAAACTTGCTTTTGATCAAAGTTTCTAAACATGAATCACTTTCTTTTATAACCCAATTTTAACAAAATCAATTTTAAAAAATTAATATATCGCCTTCCCAAATGTGCTGTTATTATTCTTTTCTTCTCTTTTCCATTTATTCTCATTGTTACCTATACACCTGATAGAAAACCAATTTATCATGTGTGGTAAGTAGTTTTGCTCTTGCTTCTTGCTAATTGGCTTATCCGCCAAGTCATTCTCCACAGCATATTTACTCTATATTGATAAAATTTTCTCA

>Gm14ciRNA1724

GTATGACTAAGACCATCTAATATTTTCTAGCTGCTTACCGAATTTTCATGTGGAACTAACCATTGGCTATTATTTTAGATATTGGTGGATGATTAAGTTCATTTGCATAGTTCTTAGTTCTTTCTGAATGAGCATATTACACTTGCACCTCTTGAAACAACTCTCTTACAAGAATACACGATGTAATATGATTCTTGGATAACAAAGGGTATATCAGTGTAATGTATAAGGAGTGTCGATATAACATAATTTCAAAGAAACAAGGAGAGTTAGTATAGGGATTAAATAATGGAGGGATGTCAGAGAAAAATTTGGCAAGACCTTAAGGGGTGTTGGTATAATTTACTCTTGATTGTAAAAGTTGTTTTCACT

>Gm14ciRNA1725

CAACCAAACCACTAAGTTCTACGGTGAATACCAAAACCGTTGATAACTATGACTTTAATATAGACGATTTCATAACAGTATATTAAGACCTCTATCTAAGTCACGAAGAAATAATTCACAAATAACGATAATCGAAAAATTAAAGAAATTCTTACAGTAACGGGACATTATACGACGAGAGAACGGGTACGAAAAACTGATTGTTAGAAGTTTATCTATACATGGTATGAGTAAAATAATCTGGAGTGA

>Gm14ciRNA1726

CATCTGTAGAGAACGTATTCAAGTACAAGAGGGGTAACGTATGTAAAGATCGTGACACGTGGGATCGAACTCGTGAATTTGAACATACCATATACATCTATATTATTCGAAAAATTGTATAGTTAAATTGGATATAAGTAACTATGTACAGGACTCGATAACACCAGAACAACTAATGAAATACACTAACAACTAAACTACGGTGAATTTCATTACATTGACGTTAACACACCTGAACCCCTACGTCTACCTTATACAGTAATAGTTAACTTCCCATTTTTTAATCTCTTTGAACACTTATTCAACCTAGAGAAAGAAACCTATTAACGGATTTAAGATTTTGAGTTTATTGAACCTGTATTACGTGTGTGAAGATTACACAACGAAAAAATATCTACGAAGACACAGTGATAAGTCATCCATGGAACCAAGGAATCGAAAGTATGGTTTAGACAAACACGACCTCGTTTAACCCATTGACCTAAAACCAAAAATGTAGTCCTACTATGGGGATGGTTTGTAGTTATTTTAGTGTCAACAAATTGTACACGAATGATCAATGATGAAGGTAACCACCACCACTTGACTATTAAAGTATACTCTTAGATTTTGACCCACCGTATAGGTTCTATTAACGAATTTACCCGATAAGTAATCGTCATGGAATTAATACAAACTAAGGAGAACTACAGTGTAGGGGAAAGTATGAAAAAACAATGTAAAATATACGAAGAAAGGACTGACGTATATGACCGAACATGAAAAGGAATAGTAGAAACATTAACATA

>Gm14ciRNA1727

CATTCTATTAGAGACCTACAAGAAAATCGTCATACGAGTACTTACTTAAATACGTCTCTTTAGATATGTCAAATTGAAAACGGTTTTCGTTAGAGTTGAGTTGATATTAAAACACGTGCATAAAATCCGACACGAACCTATTTGAAGAGTTATTCATAAATATTCTCTTCTTTATTTTACTTAGTTTGACAAAAGTATTCAATTTTAGTTGAATGGTATGCAAACTATTGTGTAAACAATAACCACCAGTCTTCGATGTTTCTCATCGAAGACGGAGATAACGACGGAACCTGCACTTTTGTCATTCTATACACGAAATCTTTACGTCTATGTTAGAACGTTATTATGTGTCTAGTCAAAAACCTCTTCAATCTACTTTCTCGAAGATATTATCAATAACATGTGTTCAACTGTTCAAATAGGTTTGTCCTGGTTGAGTTCTAAAACGATCAGAGGATTTGATCATAACTAGGA

>Gm14ciRNA1728

CAAACAAGGGTAAAATAAAATTAGAGGAAAAAAAAGCAAACAAGAAAACAAAAAACACGTTAAGCTTTGTGTTAAAACAATAAGTGATTAGCAACGTCTACTTTTTCGCTGCTAGTTTAATAGTAAACCACAATAACCCAAGGTTAGTTATAAAAAAACCTAGCTACATAGCGACGTTTAAAGTAAAAAAACTTTAACAAAATTTTAAACTAAATTCAAAAACAAGACGAACTAACACGACCACAAAGTTTTCCCTAGTGACTTTTCAGTTTAAAATTTCGAAACAAAAACCAAAATGATCACAAAGAACTAGAAAAGTCTTTTTAAGAACTAGACTACAAAAATAAATAAACAAATAAAACGGTATACTCACGAGGTAGTTCCTCACTATGATTATATATACTAACTTGGATCAATGAATGCGTACGTCAACAATTTTCCCAATGTTCTTTCAAACTACTAACTTAGTAGTAAATATAGAACACAAAACCGAGACAAGAAATGACTAAGTCTCAAACAAGTTAGTATGAGATGAGAGAGACAGTTAGAAAAAAAAAAAATCAAAAACGTAAACATCTAAGAACCCCAAACTTCGACACTTAAGGAACATAAAAAATCGAAGGGGTTCTTTCCTTAAATTAAAAATTAACTGAAATCGTAGTACAACT

>Gm14ciRNA1729

GTAAGTAAATAATTCTGGAACAAATGATCTTTTTTCTTTTTCAGTTATTTTATATTTTTTATTCTATTTTTGATATGATTGCTGGTATAATTATTTGAGTTTAGCATAATAAATGAGTGAAAATTCTGTTTAATTTCTAACTGTACCATGAGATTAATGACGGAATGCAATGATTGCAAGAGAACTATGTATTA

>Gm14ciRNA173

CATTCTTACGTTGATCCTAGTGTGTGAGTCTGAGTTACAAGCTTTCAGAAAATAAAGAATTTGGAACCACTTTGAAGAAACCTTTTCCTTTGTTGATACATGGTTGAAAAAGTTTCCTACCAATATAGTCAATGAAATAAACCACAAAAGAATACGTGGAATCGTACAGAATTTAAGAGTTTAAATTCTATTGCACAACATACTTCATGAT

>Gm14ciRNA1730

CATTTTTAAGTAGGGACATAGGAGTGTTCGTACCTTTGTTGTGGAAAAAGTAAAAAAACCATCAATGTAATGTCGGTGACGAAAAACGAAGTGACAGACGGACGAAAAGATGAACCTACCATAAGCGTATCTCCTCGTAGGGGAATAGAACTAAAAAACTTTGTATCACATCATATAATATTAACTTAGAAGCAGGTATAAATGAAAGGTAGACTTTATACTCGTTAGAAACCCAACTTAACCTATTTATACCCAAAATAGACTTACCCGCCCTGTGGTTAAAACATTATGATTAAGCCCTATGAGCACTAAAAGGAATCTTCAAATCCCTTCAAAACAAATTTTTTCGTAAAAGATAAAAGTAAAGGAAGTGTTTACTAATGTTTTTATAGTGACAAATGACAAAAACATCTAAGTATAACCTTTACCACTTTGGTTTGTACGAACCAAAAGAAAAAAAATAAAAATAAAAATATTATTAATTCGTTTTCGTTTGGACGTTACATTCAGGGATACAAACACCGAACTTCTAGGATCACTAATTATTGGTCTCACTGACAGAACGAT

>Gm14ciRNA1731

GTAGGAGTTAAGACACCATTACACCACAGTAACTGTTTTTTGTGAGATTTTCTTCAAAATCTCATTGGTTGCTTGCTGTTACAGTAATTAGTAGTATAATGAAAGTAAGTGTGCACATAGTTTTTCAAATGGTTCAAGAACGATTGATATGTTCTCTATGTGGAATAGGTGCCAACTTATCAATTTTCACATTTAATGTAAAATAACTACAAGTCTACAAGATATATATACCTTTTATGGTAAGGCCCTGGGGGGAGAATTTCAATTATGTCACGTCCAAAAATCATCAAAATAAATAGCTGGTAATATTTTGTATACATTTGAAGAACATGAAGCCCAATTGTTAAATCAACCTTACTGGTGATAGTAAATTGTAAAATGTTTATCGATAAATTAAAGCCACAGCCGTTCAATCAAAATGTTTTATTTTTATTTTTATCTT

>Gm14ciRNA1732

CATATAAGGGGAAAGAGAGAAGTTATGCTTCCTGTATTACCTGTAAAGCACCGTGTTTATAATGGAAAGCTTTAGTGGCAAAATGAAAATGGGAATTTATTTACCGTGGACCCGGGTGGGGAACCGTTCGCTTGTTCCTTCGAAGGCAAATGCTATTCAACTATTACTGGTTAGTGTTTTTATTATCATTATAATTTTTTACAAAAACGGTTGGTTAATAATGAAAGATGAATACCTTTAATAAGCAAGAATAGAATCCCTATAGCCTTTAATTTTTAACTTCTAATTTTATACACACGTGTACTAACGA

>Gm14ciRNA1733

GTATAGTAATGAAATATCCTTCAATTTACCCTAAATATTATTTTCTCCTAGTAAATTTAATTTTTCAATTTCCTATTTTAATTTTGACAGGGACCTATATTCTAGTTATGTGGTTTTCCTTTTCTATTTTCTTGTTTTCCTCTTTGTTGTTTACTCTCTTCTAGACCTTGCATTACCACAACCTGTTTTGTGATAAGTAACCAATTCAGCTTGTAAGAAACTCTCAGAGTTGTAAACTTCAGTATTCACACATGATGCGAACATTTCACTTTCAAGAAAGATCTTCTCTACTTAGAAGGTTGGACACATTTAATGATCTTGAAGTTACTCTGAGAAATTCTGGTGACAATCATTTGGAGGAGCCTATTAGCATCACTAGCCTTGAAACATTTTCACTAAGTTACAATGTCTTTTCTTCTCCATAAGTTTTTGAGAGTGTCTTGCATTTGCATCGTTA

>Gm14ciRNA1734

CATAGTACAAAGGAAAAATGTGAATGGGTACACCAAACAAAATCCAAGACGACAAACAAATAAAAACCCCTTCCTTTTCCACCTGATTCCTTTTTAACTTAATAGTTAACGTAAAGTCGACATACCTGATACCTGTATCGACGTCATCACCGTAAGAGAATGTGCAACGAACACAAAAGACATTAAAATCTACTGTACCTGTCTACTACTCCGTTACGACTTAAACCACTTGAATTAAAGATTATTCCTCATCGTACTTACTTAAGCACTATTCCCGTATTCAACAAAATAAAACACCTAAGTAGATCTGTGACATTAAAATTGTCGAACGATGAAAAAATAAAAGGACACTAACTAAACTTTCAAGAAAGAATTAAGAATTTATAACGTGTGTATGGGGACACTAAACAAAGTCTAATTGAAGACAGTTACACCTAAATTGAAAATATCAAATCGAGACTAGACTCCATATAAGGATTAACTTTCAAACATAAAAAGTTAAGATTATCCGTTATCACGTTAGACAGTGTACCAATACTTTCACCGTGTGTTACACAGGAAACACTAAAACACGGTATTTTTATCTTAGTTATACTGTAGGACAAAAATTTCGTACCTGTCAGGTAAAACCACAACATAGATACACAACATGAACAGCCTGTACTGTGTGACTGTTGTGTACAGGTTTTGTCCAGACACACATAGGTTTAAAATTTTGTTATTTTTAACCGAGGTGTTAATTACTGGGAGTAATTTTGAATGTTCGTGAGTTCTCTGACAATTATCATTTCTTCTATTTATTCCATCCACGAAAACAGTAAATAGTTTAAAGTAAAAGGTACTGATGTTATGTTAAACTGTGTATACTGTATATAGTTCTATAACATTATAACTTTGAATATATACTAAATAATTAACACTTAGGAAAAGGAATTTAAAAGTTAATATATATCCATATATAAGGTACAAGAATAGCACGTTAAAAATTTTAAACCAACAGGGGTACAGTACAACATATACGGAGACAGTCACGGACACGATGTATCAAT

>Gm14ciRNA1735

CAGACATATAACTATTCCACAGATAAATACATCACTATTTATAGAAGTAAAGAAATATTCAAATAAAATTTATTACGACATCACGTAAAAGGGAAATAAAACAACCTTAACTAATTAACTAACAGAAAATTTTGAGTAAAGACCCTTTACTATTTTTTTTAAATACGTACTAGAAAAAGAGATACTTTTTTTTCCAAATCATGATGTTCACACACCAAGATAACTTTTTAAAACAATCCTGGCTTTAGGAAAACATATAGACTTGTACATATACATCATACGACAGTACTCGCGATATATTGAGGGCTGACAAGAGCCCACACATGAAT

>Gm14ciRNA1736

CAGACATATAACTATTCCACAGATAAATACATCACTATTTATAGAAGTAAAGAAATATTCAAATAAAATTTATTACGACATCACGTAAAAGGGAAATAAAACAACCTTAACTAATTAACTAACAGAAAATTTTGAGTAAAGACCCTTTACTATTTTTTTTAAATACGTACTAGAAAAAGAGATACTTTTTTTTCCAAATCATGATGTTCACACACCAAGATAACTTTTTAAAACAATCCTGGCTTTAGGAAAACATATAGACTTGTACATATACATCATACGACAGTACTCGCGATATATTGAGGGCTGACAAGAGCCCACACATGAA

>Gm14ciRNA1737

CACACATGGTCGTAGTGGTTATAAATTAAATTTCTATTTTATACAAAATTCATTAGATAAATCCTATTTTAAACTAAAATTAAAAACTTGAAGTTGAAATTTATTAAATGATGGATTTAAAATATTTTTTTCACATTGAAAACAAAACAAGGAGGTGCGTCACTTTTGGCACACTACTCACTGTTTTAGTGTGATAAAAGATCTTTAAATCTTTAGTTTAATGAGTTTTAATTTTATACCTCTGATTTATAATTAAAAATAACTTTCATGTCCTTTTTTTTGTATAAAATGGCAATTAAACTAATTAGTAACTAGTATAATAATATTCAAAGTGTTTATAATCCTACAA

>Gm14ciRNA1738

CACAAGATTAAAGTAGGAAAAAAAGAAGAAGTTTAATAATTAAGAGCCTTCTGTATTAAGTTAACAAAATAATCCAATTGAACACCTAATACTCTTGTACCGAAACACCCACAACTTCAACTACAACACACAAAAAGGATAAATACCTTTCAGTTTAGCTTTCGCTCAAGAGAAAAAAACTCCTCTTTCCCACATTGATAAACACAAGCACTACACTTGTGTACAGTGACAAGAAATGGTTACCAAGGCTCCAAAATAACGCCCAGAACACAAACAACGACAATGACAAACGAAAACAACATATACCCAGAACTTATCAAGTCAGTTACGTTTTACACGGTACCACTAAACAAACAAACTAAAAAAACCCCTCCAACTTTTCAAATTCACACAACCTATAGGCCAGAAACGGCCCCGTCCCATCCGGAGATCCGAACGAGCGTATGTCATGAATGTATAATGGGTGTGATTGTTCCAGAAGTGTAGTAGTTATCATCCTAAGTTTGAGTGTTGGAACACTCTATAATAAGACTAGGAATACTTGACCTGAGCAACGAACACCAACAAACAAAATGGACGGTGAGACTAGTTGAGAGGAAAAAAACCATTTAAACGTTAAATCTAATTCTTAAATCCACTTTACCAAACTTTCAAATTAAGAGAGAAAAAAAACACACAAACACCATTTACACTTTTTTTACCTAAGAGCACACT

>Gm14ciRNA1739

GTATGAGTGGATTTCCCTTCTTACTCCTCCCCCTCCTAGTTTGTATTTCAATGTTATGAGTTAAAAATCATGATGCTTATTTTGTTGTAAAATGGCCATATGATCAATATTTATTTACTTTTAGTCGGAGAAGAGTAGTTCAAGTTGCAGATCAAATTTCTGTTATAGCCACATAATATGATGTTTCACTGCATATAATTGATAGGTTGTTAGGTGCCAACATAGTTGGGATCTAATCTTCCTATAGTGATACTTTTCAACATCTTTCCAAAAAAAAAAAACAATGTGATGTTTCTATCAACACATTTGTGATTTCCTAATTTGGTAGTTGACATTATTCATGGCACGCACCTTTATACTG

>Gm14ciRNA174

CATTATACAACTAAAGTCAAAAAACTACTTGACACACACGGGTACGTGTGCGCACGCACATATATATCCTTGCAAAAAGAAGTAAAGACTACGTCTGGCTCAAGTTAAATCTCTCAAATTCACTAGACGCAAAGTCATACACTACCACTTCGTTAAAGGGATTTTCATACGACACTTGTACACTTACCCGTACTAAATACTAACCTTCCCACAACACGAACAATATACTTGAATTACAACTAAACGATGATCACTAGAATAACTTAAAGA

>Gm14ciRNA1740

GTGAGTGCTTCCATTTTCCTCTTTCTAGAGATTCTCTGGTGCCACCCACATGATAAAAATTCGTTCTATACTTTTTCTTAGGTCCCCATGTTGTTTTCGACGATGATGATGGTGTTTTGTTTTTTTGGAAAATTATTAAGTTGTGTTAGTTTGTTACTGATTACTTGAAAATCCCATGTGATCTGTGTGTCCAATTGATTTTTCTTTCATGGGGTTGTGTATATCTGCATTGTAGTTGGAATTTGAATTTACTTGTTGGTGATGAGTGTGGACATGCTTGCT

>Gm14ciRNA1741

GTAATTAATTTTTTCAATCAGTTAGGGTGTGGAAGTGTGCAGCAAATATTTGGATGATGGGGTCCAGTGATTTTAAATTGCGGTTGTGGTTGCACTGTAATTGGCAAAATTGCAGACAAATGTAGCAGCAATTGCATGCCCTATGTGTTATAGAGACCCCAAAAACCACAATAATTAGGCTGCAATTGCAGTTGTGAATACCAATCTTTTCAATTGGGTCTCTTCTCAGTTAGGACCCTTACAATCTTGGTTA

>Gm14ciRNA1742

GTATTTATTACTTATTTGTTAACTGGTATGCCTGGCTTGTGTAATGTGATATTAATATGCAATTAGGATGAGAGATTTGGAGCATAGGTTTATGACTGTGTATAGGCATTTGCTTGAAAAGGGAAGACCTAATGTGCTGCACGAAATGCATTTTGCCGTGTTTGTGATCAGTGACATGCTGGCAACATTTCTTAATAATTCTTATATGTCCTCATTGCCATTTCTTATCTTAGTGTTTTTGTTATTTTTCATTTG

>Gm14ciRNA1743

GTATGTCATCTTCTGTGTTGGGTTGGATCTCCAAGTGAAAATTTGCCATCATAATTCATCATAATACTGTCAGGATTATTTTTCAGCTTTGAATTCTATTAAATTACAAAATTAGCGTATGTTCAGTGAATGTGATGCTAATGCAAACCTTGTGATGGCTGAGAAACACTGATGATTATCATTTACATGGATCCAATCCCCATGTCAATTCAATCATTAGGTTTATTAAGATTACAATTTTTTTTCTTAGTTTTATGTCAAAGCAATTCTGTCCCACCAATATATTTTTAATGGTACAAAAAATATTGAGCAGCATGTATTG

>Gm14ciRNA1744

GTAAGTGTTATGGAATTTTTAATTGAGTTGTTATGGGTTACAGTGTTATACTTATGCATGTTTCAGATGAATCGTGGCTTCATTATTATAATCTGGGCACGTGTGCGTGTGTGCGTAATTAATAATACACATTATTATTAGCTGGTTTATGCAGAAGTAAGGTTTTTATAGTTGTCGAGAAAATAAGGTTAAACCATTTTCATATAATTTGTAGGGTGTTTCCATAAGTAATATTGAACACCTTATTGAAATAAAGTGTTTAAGTTACAAGATCAATTTAACTAAATGATAAATAAATCCTTCCAAACGTTTGAAAGAAAAAGAAAGTGAGAAAAATGAGTACTAATTAACTTTCCTCACTGTGGTACGTGGTTAAGTAAAAAGAGGAAAAATGTCCCGAAAGAAAAGCAAACAAGGAAGCTGCTTTTACCCCTCATCGTTATTCCTTAGTGATCAAATTAAGGACACTTTGCTTGACTACGTTTTGTTAGGGAATTCTTGCTTTGCAGGATGTTGAATTGATATATTTTAGAGCAACAAAACCTTATTGAGATTAAAACTAAGAAAAATGTTGTTAATTTCAGCACGTATGATGAGAATATGGTATCAAACATCAAACACAAAAGATCAAAATGCGAGTTAATTTGATTGTTAAGCTATTAATACTATGAAACGGAAAATTCATTTTAAATCTCAATACAAACGAAGGATATCTTGACACATCAGCGGTAGGCTCTTGCATTTTATAGAACTCAGCTAGCCTTGATGGAGGAGAATATATATCAAGGGATTTTCTCCCTCAATTACGTGACGCAAGTCACTTCAGCAAACATTCTTAGATGCTTTCCTAAAGAAGCCAATTCAAGAAAAATGAAGCCCCTCCAACTTGTTCATACTTGGCATTGAAGAGAGGAAGGGAATTAAACTTCCATTGTTGATTGCAAGATACTATTTTAACTTGCCATGGAATTAGTATGAATATATAAGTGATCCATTATGACTAATTATTCATAGGGCACATGCATGCAGTCACAAGGTCGCTATTCCTGATTGAATTAGTATGAATAATAACCTGCATCTTTATCATTTCCTTACTTTGCTAACAATGGAAGGATGAACTTCCAAGTTTTATTCTTTCATTATATTTAACAACAATACATATTTTTATACTTCTTCACTCATCTATCTTTGATTTTTCCTATCTCTCATCAACATTTTCATTCTTTAAAATTGCAACTCCAACTCA

>Gm14ciRNA1745

CATAGACTATTCTATTCCTACATTTATAAAAAAAAAACTTCCCACAAATTTCATTGAAAAGGAGAATTTTAATTCACATCAAATAATTTACTTAAAGACATTCCTTAGTTTTGTACTTACACGTTATAGAAAATACGATTATACAGGGATACCTTAAGAATCAATTACGTTTCATCACTTAAATTTACAAGATACAATATCTTAGTTTCCTTCTCACCCTTTTTGACGAACGAATACGAGAAAGTAGAGAACGAGAGATAGGAACGGTAGATACTACGGTTTTAAGATATATTAGAATCCCTTTATCAAAGCCATTCTATCAAGTTAAGATGATGTACAAGACGTGTTATTCGAGTTCGTTGTATACGTATTAACGTATTGACTTGTTCGTTTTACTCTAACGTTCGAACTGTGACATCCGTAAACCGTAGAACATTTATGTTCTTTCTGTTGTTGCTCTACCCACTATAATTAGGTTACCACGTAAACCGTAAACTCTTAGAAATAGTTCACGACATTTATGGGCGTACAGGAAACTGCTCTTACTATACTAAATACAACTTTTTCGTTTTAACCGATTGATCGATTACTAGGACTTTTTCTGCGTATAACATCACCTACTTTTAACACACCCAAACATAGTTATATAATATCCTACGTCAATTTACCAGATTTATAGATGTCGTGACATACAACTAATACAAACAGGGACCCAGTAACTACAAT

>Gm14ciRNA1746

CATTCATAAGAAGAATTAGAGTGTATTACATAAACGTGACAGGTTTTGAAAACAAAATAAGGGATATTTACTTCTTGGATTAAGTCATTTATAAAGTTTTCAAATGAAAGAATTAGTTCTAGAGGAAAGTAATTTGGTTCATCGTTTTTAAATATGTAACATCCTTACTCTACTCGTGAAACTTTGAATTTTAGTCAGATCTAAAGACTTAACGAACTAGTGGAGTCGCGACTTATGTGTGTACGTATATTAATGTAAAATAATTGCTTACGTGAACGTATATTAATGTAAAATGATTACTTTGTACTGCGTTAGTCTTTTATTTATAGATTAATGTCCATTATTTAGTTGATTGATTTATGTCTCTAAGATATTATGTTAGGGAACAACATCCCTAATTTAGTCGTATAATAGAGTTTATTTAGTCGTACAATACATCTGTCACCGTGTATAAGGAAAACAATACATCACCTGCCTTCCGACTATCTTTCCCGATTACATTTCTAACCGTACTAATTACGTTATGTAAAAAGTTTATTACTTTGTCAAACAATGCTTAGATAACAACTTTCCCCGCTGTACATCAAAAATTTTTGCAAAGGAGTTTGAATAAAAAGTCAACTTTTCGTATTTAAATTTGAGGTACGAGTCACCGTTTTAAACTTGAAGTCACTCAATT

>Gm14ciRNA1747

GTAGACTCGCTCTTGGTTCCCTTCATATTTGAGCACACACTAAACCAGATTATGGACCATTTCTTACAGGCTTACAGCTGCTTTTATAAAAGAATCATTTTAAAAATATGAATAAATTCTTTAGTTTTTGCATGTGTTTTTCAAAACAACAGATTTTTATGATTGTAAATAAATATAAATTAACTTTTTTTTTAATACAAAAAAACTGGTCATGTATATTCTTTTGTTGTGGGACACAAATAGATAAGGGCAGAATGTGAATGGAAATACTAACCTTAAAAAAAGGTCTTGTCGTTTTCCAGAAGCTATTATTGTTTTTTTTTTCTTTTTCTGAGTATGCCTTTATCTAACTTCATTGTTATATTTTTTTCAAATATATCTTTTAATTACAATAAATACTTTTATTAAGTAAAGTCAAAACGTCCGGGCTCATGTCATATTCCATCACTAA

>Gm14ciRNA1748

CATGGTTATAGAAGCTGAGGCTAAGGAAAGAAATTAATGAAAAGGGAGAGAAAAGAGAGGTTTAGGGATTAGAGTCTAAAGCTAAAGAAAATAAGGTAAGAATTGCGCGGAGACAAGTGGGTGGAAAGAAAAAGTAAAGGTAGGCGCTCAAGGTACCTAAAACAAGTAAGTTAAATAACGACATTGAGCGAGTGCGTCCGTGGTTTAAAGCAAATAGGGACTCGCCTAGATAAAATTAAATTAAACACTTTAAAACCGTTTTTTTTACTTTTTGGGCGATGGTGAACTTTGTTACTCGCAAAACAAACTTTAAATTTATATTCATCGAAACAGGATGAAAACTAGCCACAGGAGCACGAACTTTACTTTAACATTTGAAAAAGTACAAACAATCAAGTAGCAATATTAACAAAACTTATAAGT

>Gm14ciRNA1749

CATGCGTAAAACAATACAAACTCGAATTACTTAAGTTTCCTAACTTCAACTTCAATGAGTTTATTCGTACTACATGATTAACATAAGTAGCGTAGAAAAAAATACTATAAAACTTTAAAAGTTGACTAACCTGAAATTGAAGTAAGTTCCGTATACTAAGAGGTTATTAAACGAGTTAGTACAGATAGTGAATAAGAACTTATCGACCACCTCGTTCAACTTAGTAAGT

>Gm14ciRNA175

GTACTTTGGTTTTAGAATTTGATTGCATAATATAAAACATGAGTGGTTTGTGTTTAAGTGGCATGGCATCATATCATATCATAATCTGAGATTGTGGATGAAATATTTTTCATGGTTAGAGTTTATTTGCGGAAAAAGAGGGATTTTAATTTGTTTGTGGTTGTGCACCATCTTACAAATACACACATTCATTCAGATTTGGCTCCTAGTGACAAACTATGACGTGTATGTTAGTTAAAGAGATATATAAGCTCTTTAGTTTCAGACTTCATCGCCCTCTTTCTTCCTCTTTTTGTTGTAACTCAAGGGTTGCTGTTTTTTTCCTCGTGACATGATTTGATTTCCACCTTCACTCAAGTTCTCTTTCTTCTTTCCCTTCCTTTCATTTATTTATTTTGCCTATGTTTCTTTAAATTTTAATTTATAGTTTTATACCATGTTATGTTAATGTCATATGTGCGATTTTTCTTATATTTTGTACAAGATTTGTTGATTCAATTATCT

>Gm14ciRNA1750

GTAATTCACATCAATATTCAGAATCCCTTTTTTATTTTATTTTTTCTCTCCAAAATTATTAAACCTTGGAACAATTTTCCTAGTAAAGTTGGCAGATTTCACACTGATGTAATTGATCTGTGCATTTTCCCTGACTTGGGTTTCTTGTTTTGGTCACCCAGCTTAAAATTTTAGTACTTTTGGAAACAAAAGGGTTGGAAAAAAAAATAGGGTTTTGGAGCAATTCAAGCAAACAGGAAAAAAAGGGTGCCCCTTGTTGGTCTACAATGAGTTTTACCCGAATTCATGAACATGGGCTATGATTTTTACTGCACCCCAGCTCTGTTACTGAAAAAGACTTAATTCATAATTTGTGAGACTT

>Gm14ciRNA1751

GTGTGTATGATGTCAAACTCCTTAGAGTGTGTTTGGTTTGCAGTTGGCAAGTAACAAACGTCTGTTCAACAGAGAAGTAACAAATTGTTACTTCTCATTTTTGTGCATTGGAGAGTGGGAATTTCCGTTTGCAATGATGCAAATGGGTTCCCAAACAAACACTTAATAACTAAATTTCATTGTAGTTTTTAGTGAGGAGTGAGGACAAAGATATCTGATGCATTGGTGATTCTATATAACTGACAATGTGGTACGCAAAAGAATAATTTCAGGGTGTTCTTAAACACTCAATTGACAATCCTATTTAATGGAAACTATATCAACGTGTGTCTGTATAACATGCTAGCACAGCATCCATGTCATACCTGCCTGCATCCATCTTTGCATTTACGTTCTT

>Gm20ciRNA1752

CATTCTGGCTCAGAGCAAGTTAAATCAAAAAAAATCCTTTAATTTATTACGAAAAACAAAATTTATCCAATCAAAAACAAGTTAAGATCATAAATTAAATTTATTGACATGTATAATTTCCTCATAAATAAAGTACTTATAAATCTAAATAGAACTTCGATACTATTTATGAATCCCATTTCACGAAGAAAAAGCCAGTTTTAATTAGAATAAGAGGAAATTTTCGTTGCTAAAACTAGAACAAAAGATATTAACCACCTAAAGCCGGGAGTGGGATCCTAGATTCAAAGTATTTCTCGCCTACTTAAGTAGTTAATAATTATTATACTTATGGTTTTAGTAACAAAAATTTCGTATACCTGCTTTCCAGTTAAAACTAGCTTTTATCTAACTTGATTTTTAGATTTTGTGTGAAATTGGTTTATTTATTAATCTAAAAGCCTTATTGTCGACTAATTAGATTTTTCACGAATCCCAAAACCTCTCAATTAATACCTTTTTTCTAAAAAAAGAAGAAGGAAAATTTCGACGATTCGAGATAATTTTCTTTTGTTAAGAGAAAAAAAGGGTATTATTTGACTACAGTTTTAATAAAATTTATTAATAAAAATTCAAAAACTTTCTTGAGCACGTTTTTTTTATTTTTTTTTATTTCGTTAATTACCAGCTTTTATTACCGAACAAATATTCCTTTATTCAGTTTTGGTCGTATGGCAACAATTAATACCAAAAAGGAAAAGATCACATTTTGTTCGTACGTGGTTCAAACAAACACAACAC

>Gm20ciRNA1753

GTAGTGACCCTAATCTCCTGGAGCCTGTGTGAAGGAAATCTTTTCTTGTGTGATTTATGTTGCTACATTTCCCCTTCATTTCAAAATCTTTTCATGGTTTTGGAGTTTGTGGTTCACAAGTGTGTGTTCATAATCATATTCTGGTGGTATACTATCATGTACGACTTTGATGTTATATTTTTCATGATGACCTTCATGTGTTGCGAACTTGCAAATCCCTCATTTGA

>Gm20ciRNA1754
[truncated: 6,550,565 more chars]
